# Comparative Genomic Analysis of Two Monokaryons of *Auricularia heimuer* Hei29

**DOI:** 10.3390/jof11020122

**Published:** 2025-02-06

**Authors:** Fengli Wang, Chuang Han, Jiechi Zhang, Piqi Zhang, Xiaojia Zhang, Xin Yue, Yanshu Zhao, Xiaodong Dai

**Affiliations:** 1 Institute of Microbiology, Heilongjiang Academy of Sciences, Harbin 150010, China; wang7877153@163.com (F.W.); hanchuang419@163.com (C.H.);; 2 College of Plant Protection, Northeast Agricultural University/Key Laboratory of Agricultural Microbiology of Heilongjiang Province, Harbin 150030, China

**Keywords:** *Auricularia heimuer*, dikaryon, monokaryons, comparative genomes, phytoene synthase

## Abstract

*Auricularia heimuer* is a valuable traditional Chinese fungus used as food and medicine. Hei29 is a strain derived from wild *A. heimuer* through systematic domestication and selection. It has been the primary *A. heimuer* variety in Northeast China for 30 years and offers the advantages of high yield, good commercial property, and stable traits. This study used protoplast nucleation on Hei29 to produce two amiable and paired monokaryons, Hei29-D1 and Hei29-D2. The genome of Hei29 was sequenced utilizing the Illumina PE150 and PacBio Sequel sequencing platforms. Hei29-D1 and Hei29-D2 had genomic sizes of 47.54 Mb and 47.49 Mb, GC contents of 56.95% and 56.99%, and an N50 of 2.37 Mb and 4.28 Mb, respectively. Hei29’s genome possessed two phytoene synthase (PSY) protein genes, one of which—PSY encoded by g894—has a transmembrane domain. The phylogenetic tree showed that Hei29 shared the closest evolutionary relationship with *Auricularia subglabra* TFB-10046 SS5. Collinearity analysis showed that the correlation between the two monokaryons was as high as 90.81%. Cluster analysis revealed that Hei29 contains 12,362 core genes, 223 unique genes in Hei29-D1, and 228 unique genes in Hei29-D2. This study is the first to sequence two related and paired monokaryons from *A. heimuer*, which is critical for fully understanding the genetic composition and information of the characteristic strain of *A. heimuer* in Northeast China. It establishes the data and theoretical foundation for gene mining, usage, and molecular breeding. It further promotes the genetic breeding and active substance utilization of *A. heimuer.*

## 1. Introduction

*Auricularia auricula-judae* was first described in Europe in 1789; subsequently, this name was applied to the Chinese counterpart, which was widely accepted in China. However, Wu et al. conducted a comprehensive study, selecting both wild and cultivated samples of *A. auricula-judae* from Asia, Europe, and North America. Through a combination of phylogenetic and taxonomic analyses, they discovered that the species widely distributed and cultivated in China is a previously undescribed new species, distinct from the European *A. auricula-judae*. This new species has been named *Auricularia heimuer* F. Wu, B.K. Cui and Y.C. Dai [1] *A. heimuer* is China’s second-largest edible fungal species, with a production of 7.1447 million tons in 2023 [2], or more than 90% of the world’s total. Northeast China is the main producing area of *A. heimuer*, accounting for more than 70% of the whole country’s production, and the northeast *A. heimuer* is famous for its excellent quality [3]. *A. heimuer* is a valuable traditional Chinese fungus used as food and medicine [4]. It is delicious, nutritious, and popular whether eaten directly or as a deeply processed food [4]. *A. heimuer* is well-known for its therapeutic potential, owing to the numerous bioactivities of its polysaccharide extracts, which include anticoagulant, antitumor, lipid-lowering, and immune-boosting effects [5]. The melanin in *A. heimuer* has strong antioxidant properties, effectively neutralizing DPPH radicals and superoxide anions. Additionally, the peptides present within may have antimicrobial potential, further enhancing its therapeutic profile [6]. In 1994, we collected the fruiting body of wild *A. heimuer* in the Shangzhi area of Heilongjiang Province, extracted mycelium using tissue separation, and cultivated it using systematic domestication. The Hei29 strain is a late-growing variety with high yields, excellent commercial properties, and stable characteristics [7]. It has been the primary production variety of *A. heimuer* in Northeast China for 30 years and was designated as a national edible fungus variety in 2006.

According to NCBI data, *A. heimuer* is the most sequenced species in Auricularia, including the strains A14-8, Dai13782, *Auricularia* sp. (M12), and *Auricularia* sp. (M13). In a previous study, strain Dai13782, with a genome size of 49.76 Mb, was de novo sequenced using Illumina HiSeq 4000 and PacBio RSII [8]. De novo sequencing of wild *A. heimuer* mononuclear strain A14-8 from Jilin province revealed that its genome size was 43.6 Mb [9]. The two strains of *A. heimuer* were sequenced using single-spore isolation. Although this approach clarifies the genetic background, it may not capture all the genetic variation present in the parental strains. Comparative genomics leverages genome sequencing to compare known genes and genomic structures, thereby elucidating gene function, expression mechanisms, and evolutionary relationships among species [10,11]. Most edible and medicinal fungi are heterokaryotic. In genomic studies of *Lentinus edodes* [12] and *Taiwanofungus Camphoratus* [13], two homologous and paired monokaryons derived from their parents have been utilized. However, similar reports for *A. heimuer* and other species are lacking. Sequencing and comparing the genomes of two monokaryons of the same strain can help understand the coordination of heterokaryons in regulating fruiting body growth and development. Comprehending the biology, evolution, and biosynthesis of secondary metabolites is the first step.

To comprehensively understand the gene function of *A. heimuer* and enrich its genomic data resources, we nucleated protoplasts of the representative strain Hei29 in Northeast China to obtain two related and paired monokaryotic strains, Hei29-D1 and Hei29-D2. We then conducted de novo genome sequencing, assembly, and annotation of these strains, followed by comparative genomic analysis. This work can fully understand the genetic composition and information of the characteristic strain Hei29 in Northeast China, laying a solid foundation for gene mining, utilization, and molecular breeding. It will advance the genetic improvement and exploitation of bioactive compounds in *A. heimuer*.

## 2. Materials and Methods

### 2.1. Strain and DNA Preparation

*A. heimuer* Hei29 was provided by the National Edible Fungus Germplasm Bank (Haerbin, Heilongjiang, China), No. HMCC50060. For the nucleation of mycelial protoplasts, the treatment conditions were as follows [14]: 31 °C, 1.0% enzyme concentration, 5-day-old mycelium, 4 h of enzymatic hydrolysis, and 0.5 mol/L sucrose as the osmotic stabilizer. The candidate strains were stained with the fluorescent nuclear dye Hoechst 33258, and the monokaryons and dikaryon were distinguished under a fluorescence microscope. Two monokaryons, Hei29-D1 and Hei29-D2, were identified through confrontation assays. The locking association of the Hei29-D1 and Hei29-D2 fusion strains was observed under a light microscope to confirm the compatibility and pairing of these two monokaryotic strains. High-quality DNA of Hei29-D1 and Hei29-D2 were extracted using the Magen RaPure Plant DNA Mini Kit (Invitrogen, Guangzhou, China) and analyzed using agarose gel electrophoresis.

### 2.2. Genome Sequencing, Assembly, and Annotation

Whole genome sequencing was performed on the Illumina PE150 platform and PacBio Sequel system [15]. For the PacBio sequencing library, 5–10 μg of genomic DNA was sheared into 10–15 Kb fragments using a g-TUBE device. The g-TUBE utilizes centrifugal force to propel the sample through minute apertures in a ruby, thereby shearing the DNA. The size of the DNA fragments can be controlled by adjusting the centrifuge speed. This adjustment alters the flow rate through the pores, which in turn modifies the shear force exerted on the DNA. Typically, a higher rotation speed results in smaller DNA fragments. The library was constructed using the SMRTbell^®^ Express Template Preparation Kit 2.0. Briefly, the sheared fragments were processed through single-strand overhang removal, DNA damage repair, end-repair, A-tailing, and barcoded overhang adapter ligation. The library was quantified using a Qubit 3.0 Fluorometer (Invitrogen, Carlsbad, ON, Canada), and the size of the library was checked using an Agilent 2100 Bioanalyzer System (Agilent Technologies, Waldbronn, ON, Germany). Subsequent steps were followed as per the manufacturer’s instructions to prepare the SMRTbell library. The library was sequenced using the PacBio Sequel platform.

PacBio reads were assembled using Hifiasm [16]/Canu [17]. Then, we recorrected the genome with the software Pilon using previous Illumina data. The Prodigal [18]/Augustus [19] gene-finding software were used for finding coding genes. Transfer RNAs (tRNAs) were detected in the genome using the program tRNAscan-SE [20] with the default parameter settings. rRNA was identified by using Barrnap. Other RNAs were identified by the rfam database. The coding genes were annotated with the National Center for Biotechnology Information (NCBI) nr database by Diamond. Then, the functions of the genes were annotated by the GO [21] database, and the pathways were annotated using the KEGG [22] database. The proteins encoded by genes were classified on a phylogenetic classification by the database of COG. Diamond was used to search the protein sequences within the CAZy database, Swiss_Prot database, Pfam database, CARD database, VFDB database, or DFVF database with E < 1 × 10^−5^.

### 2.3. PSY Gene Family Analysis

AntiSMASH was employed to identify and characterize the secondary metabolite biosynthetic gene clusters (BGCs) in Hei29. Tertiary structure prediction of the PSY proteins was then performed using SWISS-MODEL (https://swissmodel.expasy.org, accessed on 13 November 2024) to predict the tertiary structure. The subcellular localization of PSY proteins and the presence of signal peptides and their cleavage sites were predicted using TargetP (https://services.healthtech.dtu.dk/services/TargetP-2.0/, accessed on 5 January 2025) and SignalP-6.0 (https://services.healthtech.dtu.dk/services/SignalP-6.0/, accessed on 5 January 2025). Initially, a protein sequence alignment of the PSY protein was conducted using the BLASTp tool from NCBI [23]. The non-redundant protein sequence database (nr) was selected as the target database, and the default parameter settings were used to ensure the efficiency and reliability of the alignment. The top 10 proteins with the highest sequence similarity to the PSY protein were identified and selected based on the alignment scores. Afterward, the sequences were aligned using MAFFT [24] with the following parameters: mafft --maxiterate 1000 --localpair input.fasta > output.fasta. Then, the phylogenetic tree was constructed using IQtree [25] with the following parameters: -m MFP -bb 1000 -alrt 1000 -abayes -nt AUTO.

### 2.4. Comparative Genome Analysis

Cd-hit (v4.6) was used to cluster the CDS sequences from the Hei29-D1 and Hei29-D2 genomes. The clustering parameters were as follows: 70% sequence similarity, 60% shortest sequence length, and 60% alignment length. We used the one-step MCScanX-super fast program of TB tools (v2.112) [26] to gather the collinearity relations of Hei29-D1 and Hei29-D2, which were then visualized using the dual synteny plot.

## 3. Results

### 3.1. Acquisition and Morphological Description of Two Monokaryons

**Figure 1:**
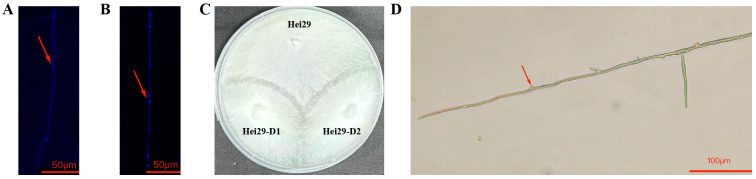
Identification of Hei29-D1 and Hei29-D2: (**A**) fluorescence status of Hei29 monocytic strain; (**B**) fluorescence status of Hei29 binuclear strain; (**C**) Hei29 antagonism diagram; (**D**) lock-like association of Hei29-D1-D2.

Hei29 mycelia were cultured in the dark for 5d, and the protoplasts were prepared and regenerated following the method described by Han et al. [14] to obtain the test strain. The mycelial protoplast nucleation method was preferred because it could completely replicate its parents’ genetic characteristics while ensuring accurate genetic information transmission [27]. The monocytic hypha cells of *A. heimuer* have one nucleus between their two diaphragms (Figure 1A), whereas the binuclear hypha cells have two nuclei (Figure 1B). Fluorescence labeling allowed for unambiguous separation of the monocytic and binuclear states of the tested strains. The two different karyotypes were further validated using the confrontation method. The two monokaryons were congenial and paired with each other. They both were antagonistic to Hei29 (Figure 1C) and were subsequently identified as Hei29-D1 and Hei29-D2. The affinity of the two monokaryons was verified by observing their lock-like association when engaged in forming the dikaryon (Figure 1D).

### 3.2. Genome Sequence Assembly

**Figure 2:**
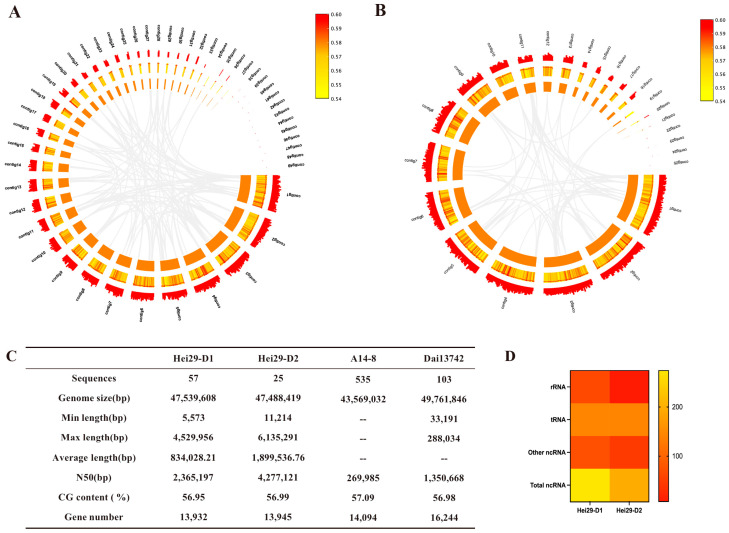
Genomic information of Hei29: (**A**) genomic circles of Hei29-D1; (**B**) genomic circles of Hei29-D2. The first circle represents the gene length, the second circle is the GC content, and the third circle is the gene density (from inside to outside); (**C**) essential genomic features of *A. heimuer*; (**D**) differential ncRNA numbers in Hei29-D1 and Hei29-D2.

The genome size of Hei29-D1 (PRJNA1181138) was 47.54 Mb, with 57 contigs assembled. The GC content was 56.95%, whereas the N50 was 2.37 Mb. The genome size of Hei29-D2 (PRJNA1181141) was 47.49 Mb, assembled into 25 contigs with 56.99% GC content and 4.28 Mb N50 (Figure 2C). Additionally, correlation analysis between GC content and sequencing depth revealed no significant GC bias in the genomes of Hei29-D1 and Hei29-D2. The average sequencing depth was 13.15× for Hei29-D1 and 31.35× for Hei29-D2, with a genome coverage rate of 100% for both strains (Figure S3). Genome Circos plots are presented for the Hei29-D1 and Hei29-D2 genomes, displaying the genome length, GC content, and gene density in order from the innermost to the outermost circle (Figure 2A,B).

Hei29-D2 had 193 ncRNA, while the number for Hei29-D1 was much higher at 274 ncRNA (Figure 2D). The primary reason for this phenomenon was the variation in rRNA content. rRNAs in fungi are essential in protein synthesis and play a key role in regulating gene expression and cell growth [28]. Hei29-D1 possesses 63 rRNAs, whereas Hei29-D2 contains only 8. Within Hei29-D1, the rRNAs included 17 18S rRNAs, 16 5.8S rRNAs, 16 28S rRNAs, and 14 5S rRNAs. These rRNAs are distributed across eight contigs, including contig 40, 42, 43, 45, 46, 48, and 50. The rRNAs in Hei29-D2 are predominantly found in contigs 16 and 25, with each contig containing two copies of the 5S, 5.8S, 18S, and 28S rRNAs.

The total length of Hei29-D1 (4,623,761 bp, 9.73%) was higher than Hei29-D2 (4,248,365 bp, 8.95%) (Table 1). Transposable elements (TEs) affect the genetic expression, genetic diversity, and adaptive evolution of *A. heimuer*, including short interspersed elements (SINEs), long interspersed nuclear elements (LINEs), and long terminal repeat (LTR) and DNA elements [29,30,31,32,33]. Hei29-D1 (4,001,419 bp, 8.42%) had a slightly higher TE content than Hei29-D2 (3,736,397 bp, 7.87%). The TE results of Hei29-D1 and Hei29-D2 showed that LTR accounted for the highest proportion in the two monokaryons, while Hei29-D1 (1031) had a higher number than Hei29-D2 (918). SINEs in Hei29-D1 (22) were slightly higher than in Hei29-D2 (16). Hei29-D1 contains 612 LINEs, while Hei29-D2 possesses just 397. DNA elements are the only TEs with a higher count in Hei29-D2 (217) than in Hei29-D1 (67).

### 3.3. Genome Sequence Annotation

**Figure 3:**
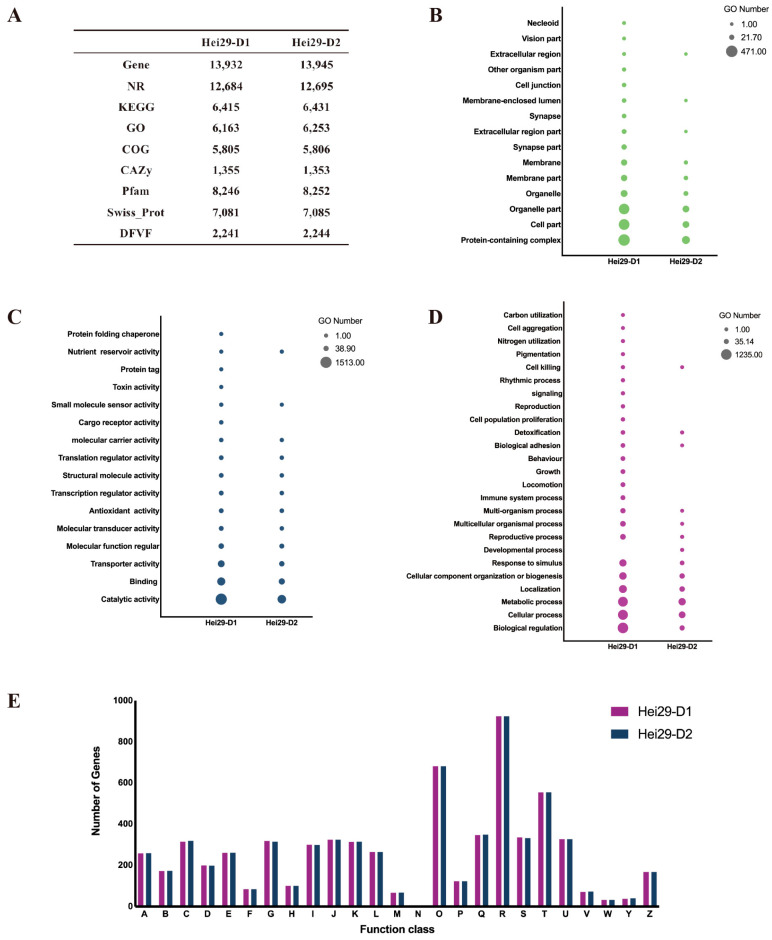
Functional annotation of the Hei29 genome: (**A**) annotation of the Hei29 database; (**B**) molecular function (GO) of Hei29; (**C**) cellular component (GO) of Hei29; (**D**) biological process (GO) of Hei29; (**E**) COG of Hei29 (A: RNA processing and modification, B: chromatin structure and dynamics, C: energy production and conversion, D: cell cycle control, cell division, and chromosome partitioning, E: amino acid transport and metabolism, F: nucleotide transport and metabolism, G: carbohydrate transport and metabolism, H: coenzyme transport and metabolism, I: lipid transport and metabolism, J: translation, ribosomal structure, and biogenesis, K: transcription, L: replication, recombination, and repair, M: cell wall/membrane/envelope biogenesis, N: cell motility, O: posttranslational modification, protein turnover, and chaperones, P: inorganic ion transport and metabolism, Q: secondary metabolites biosynthesis, transport, and catabolism, R: general function prediction only, S: function unknown, T: signal transduction mechanisms, U: intracellular trafficking, secretion, and vesicular transport, V: defense mechanisms, W: extracellular structures, Y: nuclear structure, Z: cytoskeleton).

As shown in Figure 3A, the two monokaryons had approximately identical gene counts and comparable annotation findings. The terms and numbers of gene annotations obtained from the KEGG, COG, NR, and CAZy databases were similar. However, there were significant discrepancies between Hei29-D1 and Hei29-D2 in the GO database, with Hei29-D1 containing several unique terms (Figure 3A).

The highest proportion of genes was annotated for general function prediction using the COG database annotation. The results were consistent with the annotation of the Dai13742 genome. Figure 3E illustrates that both Hei29-D1 and Hei29-D2 were predominantly annotated for these three categories: signal transduction mechanisms (R); posttranslational modification, protein turnover, and chaperones (O); and secondary metabolites biosynthesis, transport, and catabolism (T). There was little difference in the two strains, with Hei29-D1 having counts of 924 for R, 682 for O, and 554 for T, whereas Hei29-D2 had 924 for R, 682 for O, and 555 for T (Figure 3E).

The terms and gene number of the annotation for the two Hei29 strains in the GO database differed significantly. In the biological processes (Figure 3D), Hei29-D1 featured 25 terms and Hei29-D2 had 13. Most genes were annotated as biological regulation, with 1235 genes in Hei29-D1 and 46 in Hei29-D2. Hei29-D1 outperformed Hei29-D2 for the number of genes annotated for cellular and metabolic processes, with metabolic processes being the most dominant category for Hei29-D2. Hei29-D1 annotation contained 12 unique terms, including cell growth and proliferation, cell aggregation, immune system, and pigment deposition. In the molecular function, Hei29-D1 annotated 16 terms and strain Hei29-D2 annotated 12 terms (Figure 3C). The two monokaryons showed maximum annotations for catalytic activity, with Hei29-D1 having 2.5 times more genes than Hei29-D2. In the cellular component, Hei29-D1 was annotated for 15 terms compared to 9 terms for strain Hei29-D2 (Figure 3B). Maximum genes were annotated as protein-containing complexes, with more genes in Hei29-D1 than in Hei29-D2.

**Figure 4:**
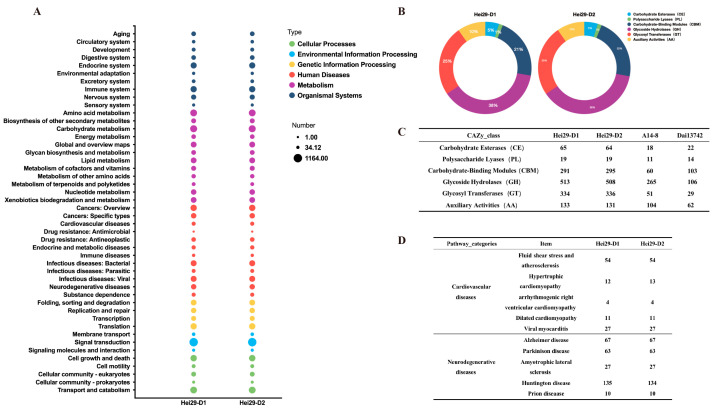
Functional annotation of Hei29 genome: (**A**) KEGG annotation of Hei29; (**B**) CAZy annotation distribution maps of Hei29-D1 and Hei29-D2; (**C**) CAZy annotation of *A. heimuer*; (**D**) annotation of genes in Hei29-D1 and Hei29-D2 related to cardiovascular disease and neurodegenerative disease pathways.

The annotation of the two strains in the CAZy database yielded the following categories: Glycoside Hydrolases (GH), Glycosyl Transferases (GT), Carbohydrate-Binding Modules (CBMs), Auxiliary Activities (AA), Carbohydrate Esterases (CE), and Polysaccharide Lyases (PL) (from maximum genes to minimum) (Figure 4B). Through comparative analysis of the CAZy annotations of A14-8, Dai13742, Hei29-D1, and Hei29-D2, we discovered that the content of GH was the highest across strains but Hei29 had higher representation than A14-8 and Dai13742 (Figure 4C).

KEGG annotation primarily provides information on the main biochemical metabolic pathways in which proteins participate [34], which is essential for understanding the processes of transformation, synthesis, and decomposition of substances in *A. heimuer*. The annotation results of the two strains were similar, with most genes annotated as metabolism-related, including carbohydrate, amino-acid, lipid, and nucleotide metabolism (Figure 4A). Hei29 ranks second in the human diseases annotation results (Figure 4D), with 12 two-level classifications. Many Hei29 genes were annotated related to cardiovascular and cerebrovascular diseases, including 17 proteins such as NOX1, HSP90, AMPK, GST, and p38 in fluid shear stress and atherosclerosis (Figure S3A). Gene annotation identified multiple key nodes in the AD KEGG pathway in the Hei29 genome, including APP-BP1, GAPD, IRE1, CYC, and others (Figure S3B).

### 3.4. Identification of the PSY Gene

**Figure 5:**
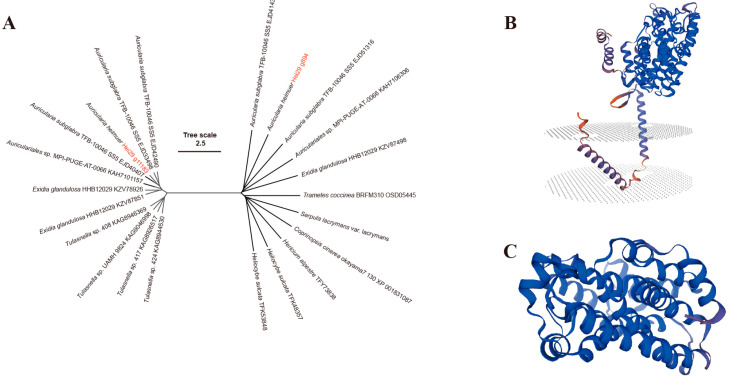
Identification of the PSY gene: (**A**) an evolutionary tree of phytoene synthase protein family (the genes g894 and g11183 are numbered within the Hei29-D1 genome); (**B**) prediction of phytoene synthase protein tertiary structure (g894); (**C**) prediction of phytoene synthase protein tertiary structure (g11183).

The terpenoids of *A. heimuer* have received less attention. A study by Zou et al. identified 14 candidate genes involved in triterpene biosynthesis [35]. The Hei29 genome was analyzed using antiSMASH, and both Hei29-D1 and Hei29-D2 contained 20 BGCs. Blast alignment of the core biosynthetic gene of BGCs showed that the BGCs of the two monokaryons were corresponding (Table S4), described below as Hei29-D1 gene ID. The Hei29 genome comprised two PSY genes, one terpene cyclase gene (lanosterol synthase, LSS), two Tri5 trichodiene synthase genes, and three terpene synthase C-terminal genes (Table S5). The PSY protein is the first rate-limiting enzyme in the carotenoid biosynthesis pathway. Its activity determines the fungi’s ability to synthesize carotenoids. Multiple genes encode PSY proteins, and their number and function vary by species, with only one PSY gene in *Arabidopsis thaliana L.* [36], three genes each in *Nicotiana tabacum L.* [37], *Oryza sativa L.* [38], *Zea mays L*. [39], *Solanum Lycopersicum L.* [40], and *Triticum aestivum L* [41], and four PSY genes in *Spinacia oleracea L.* [42]. Ten mushroom protein genes from the NCBI BLASTp alignment were chosen for gene family phylogenetic tree analysis using g894 and g11183. The results showed that g894 and g11183 were located on different branches of the phylogenetic tree, indicating that they are distantly related to each other (Figure 5A). This suggests that they may have undergone a long period of independent evolution and differentiation in the early stages, leading to significant genetic differences. From their positions on the tree, g894 and g11183 were found to be located in the same branch and closest to EJD41437 and EJD33498 of *Auricularia subglabra* TFB-10046 SS5, respectively, in the phylogenetic tree. This indicates that Hei29 is most closely related to *Auricularia subglabra* TFB-10046 SS5 [43]. BGCs of two PSY protein genes (g894 and g11183) were identified, and their encoded protein tertiary structures were predicted using the Swiss-Model software. The analysis revealed that the PSY protein encoded by g894 contains a transmembrane domain (Figure 5B), while the protein encoded by g11183 does not (Figure 5C). The predicted tertiary structures of these two proteins were distinct, consistent with the phylogenetic tree results. However, according to a bioinformatics analysis by Liu et al. [44], PSY proteins generally lack transmembrane domains. Additionally, signal peptide prediction using TargetP and SignalP-6.0 did not identify any signal peptides in these proteins.

### 3.5. Comparative Genomics

**Figure 6:**
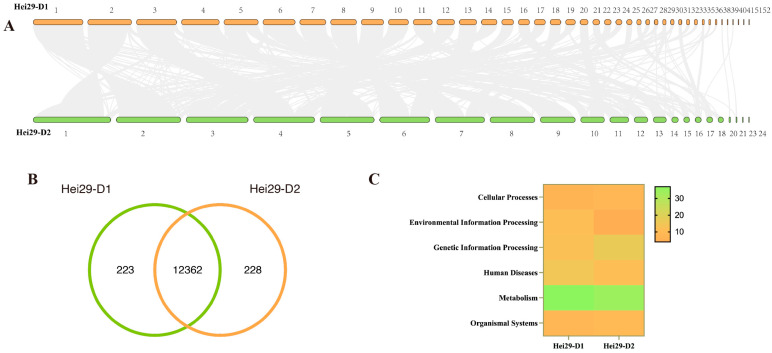
Comparative genomes of Hei29-D1 and Hei29-D2: (**A**) collinearity analysis of the Hei29-D1 and Hei29-D2 genomes; (**B**) a Venn diagram for co-unique genes in Hei29; (**C**) unique genes in Hei29-D1 and Hei29-D2 based on KEGG annotation.

The strain Hei29 was self-selected and has been the primary production variety of *A. heimuer* in Northeast China for 30 years. There were 27,877 genes in the two monokaryons, and 25,315 showed collinearity, with a correlation as high as 90.81% (Figure 6A). The high collinearity between the two monokaryotic strains could explain Hei29’s stability and non-degradation. No collinearity between two species indicates that the genes have been rearranged during evolution. There were 2562 rearranged genes between the two mononuclear strains, accounting for approximately 9.19%.

Understanding the co-unique genes of Hei29 allows us to understand the similarities and differences between the two monokaryons and the whole system of Hei29 [45]. There were 12,362 core genes, 223 unique Hei29-D1 genes, and 228 unique Hei29-D2 genes (Figure 6B). Hei29’s unique genes accounted for only 1.8%. The gene difference between the two monokaryons was minimal, consistent with the collinearity analysis results. The two strains shared a vast number of genes, which covered almost every direction and field. Several unique genes were annotated in metabolism, human diseases, and genetic information processing (Figure 6C). However, most unique genes were not annotated due to limitations in databases and research methodologies. There are two distinct monokaryons in *A. heimuer*, and the unique genes discovered through comparative analysis may shed more light on whether monokaryons can perform specific functions in *A. heimuer*. Hei29-D1 comprises 13 genes that regulate gene expression, including the large subunit ribosomal protein, RNA polymerase-associated protein RTF1, ATP-dependent RNA helicase DDX3X, and superkiller protein 3 (SKI3). Even though *A. heimuer* does not contain aflatoxin, our study identified an aflatoxin B synthase protein gene. According to studies, *A. heimuer* has a high detoxifying effect on aflatoxin B1 and may eliminate some of it [46]. Terpenoids have long been the focus of attention for edible and medicinal fungi. Although the terpenoids of *A. heimuer* are unknown, our study discovered two STE24 endopeptidase and hexaprenyl-diphosphate synthase protein genes involved in the metabolic pathway of terpenoid backbone biosynthesis in Hei29-D1. The unique genes of Hei29-D2 included protein genes from the cytochrome P450 family, solute carrier family 25, p24 family, and DnaJ homolog subfamily. Furthermore, we identified four genes encoding proteins involved in the cell cycle: cyclin-dependent kinase 8/11, cell division cycle 14 cell, division cycle protein 37, and poly ADP-ribose polymerase.

## 4. Discussion

For the first time, we sequenced and compared two monokaryon strains of Hei29, Hei29-D1 and Hei29-D2. Genome sequencing of *A. heimuer* has been previously reported for strains A14-8 [9] and Dai13742 [8] using monokaryon strains isolated via single-spore techniques. In contrast, this study employs monokaryon strains derived from mycelial protoplast regeneration. This method offers the advantage of more complete and accurate retention of genetic information compared to single-spore isolation. The genome sizes of Hei29-D1 and Hei29-D2 are relatively close, falling between A14-8 and Dai13742. The GC content of Hei29-D1, Hei29-D2, and Dai13742 was similar, while A14-8 had slightly higher GC content. Although Hei29-D1 had more than twice as many contigs as Hei29-D2, the number was much less than A14-8 and Dai13742. This phenomenon could be due to different methods used for obtaining monokaryons. Both A14-8 and Dai13742 were obtained by single-spore isolation of the fruiting body, while the monokaryons in this study were obtained using the mycelial protoplast regeneration approach. It is also possible that the sequencing techniques or the strains themselves differ significantly.

Hei29 has been the primary *A. heimuer* cultivar in Northeast China for 30 years, largely due to its stable traits and resistance to degradation since its initial breeding. Most of the other *A. heimuer* strains developed over the same period, including 8808 and 9809, were eliminated due to degradation. The monokaryon strains, Hei29-D1 and Hei29-D2, have similar genome sizes and GC content, with good collinearity and a 1.8% unique gene content. The annotation results acquired from the COG and KEGG databases revealed that the genome structure and function of the two strains were quite similar, which helped to comprehend the stability and consistency of Hei29’s genetic information. Strain degradation is a serious problem caused by many factors, such as the changes in genetic material, the influence of the external environment, and the influence of human factors [47]. This can lead to economic losses and the discontinuation of experimental research. Although Hei29’s genomic information is insufficient to solve the strain degradation problem, it is an excellent experimental material for studying strain degradation because of its low degeneracy and stable traits.

*A. heimuer* is a binucleate heteronuclear basidiomycete; however, there is no consensus on the primary and accessory nuclei [48]. A comparative analysis of the genome annotation of Hei29-D1 and Hei29-D2 revealed that the gene annotation results from the COG, KEGG, and CAZy databases were similar; however, there were significant discrepancies in the GO database, ncRNA, and repetitive sequences. In fungi, the amount of 5.8S, 18S, and 28S rRNA is usually consistent, although the amount of 5S rRNA might vary. Hei29-D1 contains 63 rRNAs, which is significantly higher than the 8 rRNAs found in Hei29-D2. This indicates that Hei29-D1’s rRNA is more stable and actively involved in protein synthesis, suggesting a higher metabolic demand and a more essential role in the growth and metabolism of *A. heimuer*. In the genome of *A. heimuer*, TEs such as SINEs and LINEs are crucial for gene regulation and genome stability. In the *A. heimuer* genome, transposable elements like SINEs [49] and LINEs [50] are crucial for gene regulation and genome stability. SINEs primarily influence gene expression, chromatin conformation, and cell proliferation, with Hei29-D1 showing significant advantages in these areas. Meanwhile, LINEs play key roles in regulating gene expression, maintaining genome stability, and influencing disease mechanisms, where Hei29-D1 may be more important. In contrast, DNA elements dominate in expression regulation, cell signaling, and chromosome structure, with Hei29-D2 likely having an edge in these functions. Despite limitations in research and technology that leave many TEs unclassified, it is speculated that Hei29-D1 primarily drives growth, development, and metabolic regulation, whereas Hei29-D2 focuses on cell signaling. The two strains jointly regulate gene expression and maintain chromosome structure, highlighting a division of labor and cooperation. In the GO annotation, Hei29-D1 exhibits higher diversity and complexity in key mechanisms—such as biological regulation, cellular processes, and metabolic processes—which are key mechanisms for organisms to retain physiological functioning and adapt to environmental changes, and Hei29-D1 probably dictates Hei29’s adaptability. Hei29-D1 may be more valuable in investigating certain specific functions, such as the synthesis of melanin, which may be related to pigment deposition and needs further investigation. Catalytic activity is essential for understanding metabolic pathways, signal transduction, and cellular responses to the external environment in organisms. According to the analysis, the Hei29-D1 genome is more diverse and complex in cellular structure, molecular function, and biological processes, and it may have advantages in growth and development, metabolic regulation, and protein function, which are more in line with the popular concept of the primary nucleus.

In the CAZy database, Hei29 had higher representation than A14-8 and Dai13742, which could be due to sequencing technology and database limitations or strain differences (Figure 4C). According to reports, *A. heimuer* GH is more abundant than 11 other types of mushrooms, including *L. edodes*, *Wolfiporia cocos*, and *Ganoderma lucidum* [9]. The significance of GH primarily lies in its ability to efficiently transform various carbon sources in the environment while promoting growth and reproduction [51], indicating that Hei29 is more environmentally adaptable. Yuan et al. compared Dai13742 to eight common edible fungi, revealing that only CBMs had more than the average number of genes. *A. heimuer* is a significant saprophytic fungus that obtains nutrients for growth primarily through the breakdown of cellulose. Yuan et al. suggested that this could explain the abundance of GH [6] but this paper suggests that CBMs are the primary reason. The fruiting body of Hei29 belongs to the multi-stringed type, with a more wrinkled structure on the back, hypertrophic ear lobes, and a high amount of colloid and polysaccharides. Hei29 has a 61.70% total sugar content, 6.72 % protein content, and 2.65 % ash content [52]. *A. heimuer* polysaccharide (AHP) is a bioactive molecule with therapeutic properties; however, the molecular mechanism of AHP synthesis remains unknown. GT regulates biological reactions [53], enhances and activates the immune system, and is essential for polysaccharide biosynthesis [54]. Fan et al. investigated the gene transcription profile of IPS synthesis from *A. heimuer* under deep fermentation conditions for the first time and found that optimizing the fermentation process significantly increased the transcription levels of pgm-1, ugp, and pgi genes involved in IPS synthesis [55]. Uridine diphosphate-glucose pyrophosphorylase (UGPase) is a vital enzyme in the polysaccharide biosynthesis pathway. Sue et al. identified two AhUGP genes in the genome of *A. heimuer* [56]. Their promoter region contained more light-responsive and hormone-responsive elements, and AhUGP1 expression significantly correlated with mycelia growth rate, biomass, and polysaccharide content [56]. The UGP gene influences polysaccharide synthesis in *Grifola frondosa* [57], *Coprinopsis cinerea* [58], and *G. lucidum* [59]. Our study identified 11 and 10 UGPase-related genes in Hei29-D1 and Hei29-D2, respectively (Table S5).

*A. heimuer* is a traditional Chinese edible and medicinal fungus. The Hei29 genome contains several genes involved in carbohydrate, amino acid, lipid, terpenoids, and polyketides metabolism, it is an excellent genetic explanation for why Hei29 is rich in nutrients and capable of producing a wide range of bioactive secondary metabolites. *A. heimuer* research mostly focuses on nutrition, health, and the development and application of health foods. Studies have shown that it contains genes that can produce bioactive molecules with antioxidant, anti-proliferation, and antitumor therapeutic properties [60]. The anticancer effect of *A. heimuer* has been demonstrated by using its extract to enhance the immune response of tumor tissue, stimulating tumor cell apoptosis and inhibiting angiogenesis [61,62,63]. A polysaccharide extract from *A. heimuer* was administered to a mouse model with a specific disease. Identifying relevant indicators and the omics analysis demonstrated that it could achieve the treatment goal of up- or down-regulating a disease’s anabolic pathways in vivo [64]. Mushrooms have been shown to inhibit the growth and reproduction of tumor cells in various malignancies [65]. *A. heimuer* mainly plays a role in breast cancer, liver cancer, and cervical cancer [66,67,68,69,70]. According to the results of the Hei29 genome, there is a broader research scope in the field of cancer, such as melanoma, non-small cell cancer, and bladder cancer. Identifying these genes provides molecular-level evidence for *A. heimuer*’s potential in anticancer therapy and possible targets for developing new anticancer strategies and medications. The Hei29 genome identified numerous genes associated with cardiovascular and cerebrovascular diseases and neurodegenerative diseases. *A. heimuer* may achieve prevention and improvement in these diseases because of its anti-inflammatory, anti-thrombotic, and free radical scavenging activity. Mushrooms, as functional foods, protect against and improve cardiovascular and cerebrovascular disorders, as well as neurodegenerative diseases [71,72,73]. Natural ACE inhibitors are popular in hypertension research, including *L. edodes* [74], *G. lucidum* [75], and *Agaricus bisporus* [76]. *A. heimuer*, a mushroom recognized for its blood-pressure-lowering properties, may inhibit hypertension by 61.23% under appropriate conditions [77]. Some studies have found that mushrooms such as *G. lucidum* [78], *Hericium erinaceus* [79,80], *Grifola frondose* [81], and *L. edodes* [82] can help alleviate Alzheimer’s disease (AD). In addition, studies have uncovered the molecular interactions between compounds derived from *Sanghuangporus* and Alzheimer’s disease; within *Sanghuangporus*, 374 disease-related targets have been identified, including crucial ones such as JAK1, LCK, MAPK1, STAT3, and STAT1 [83].

Phylogenetic analysis revealed that the PSY proteins encoded by g894 and g11183 belong to distinct branches and are evolutionarily distant, indicating that the PSY proteins may have mutated at the early stage of evolution. Structural prediction indicated that the PSY protein from g894 contains transmembrane domains, supporting the phylogenetic findings. According to the bioinformatics analysis of the PSY protein in *Chlorella vulgaris* by Liu et al. [44], the PSY protein does not possess a transmembrane domain but the first 45 amino acid residues are predicted to be chloroplast transport peptides [44]. Although the PSY protein encoded by g894 was predicted to transmembrane, no signal peptide was found when signal peptide prediction was performed using TargetP and SignalP-6.0. Further experimental validation and detailed structural analysis are needed to accurately elucidate the membrane-binding properties and functions of PSY proteins. *A. heimuer* contains carotenoids. This term describes triterpene compounds and their derivatives with antioxidant, immune-regulating, anticancer, and anti-aging properties. The discovery of carotenoids provides potential candidates for the development of novel nutritious foods or drugs using *A. heimuer* and aids in the understanding of related metabolic pathways and biosynthetic mechanisms. It provides a scientific basis for future biotechnology applications and genetic improvements.

*A. heimuer* has a complex genetic background and is prone to continuous variation due to environmental influences. There is no clear relationship between phenotype and genotype, and conventional breeding methods are ineffective for genetic improvement. We obtained the complete genome information of Hei29 through whole genome sequencing of two related and paired monokaryons. The results revealed numerous genes and metabolic pathways that respond to and adapt to their environment and a high collinearity between the two monokaryons. This helps to study the gene expression regulation and stability during genetic transformation. Understanding co-unique genes is significant for gene editing, natural metabolite research, and genetic breeding. Based on the results of co-unique genes, targeted procedures such as gene editing and genetic modification can be performed to create the foundation for genetic breeding. The genetic transformation system of *Auricularia* mycelium is already established, and *Agrobacterium tumefaciens* is responsible for the overexpression of a gene. However, it is unclear whether the experimental chassis cells should select mono-A, mono-B, or dikaryon based on these needs. For example, a single monocyte can be chosen for gene editing to obtain high-yielding strains for secondary metabolites, which can be quickly obtained by fermentation and other means. Monokaryons A or B can be selected or mutated simultaneously to identify the function of pigment-related genes, and the depth of the fruiting body color can be used to evaluate the gene’s function. The functional determination of the unique gene can be used to silence, knock out, overexpress, and perform other monokaryon operations with the gene, allowing for the speedier identification of new genes.

In this study, we sequenced and analyzed the genomes of two related and paired monokaryons of Hei29 for the first time, thereby enriching the genomic data resources of *A. heimuer*. It reveals the genomic structure and genetic information of these monokaryons and highlights their genetic differences. Based on Hei29’s characteristics, this study provides an ideal experimental model for exploring the mechanism of strain degradation, which is crucial for promoting genetic breeding and maximizing the use of active substances in *A. heimuer*. In addition, these findings provide theoretical support for developing the medicinal value of *A. heimuer*, especially as an antitumor and anti-aging agent. Genome annotation aids in understanding the differences between the two karyotypes and is critical for identifying and confirming the major and minor karyotypes. It also provides a deeper understanding of the role of gene expression and function in various karyotypes, resulting in a comprehensive and multidimensional standard for determining major and minor karyotypes. In this study, we conducted genome sequencing and comparative analysis of two strains of the same species; however, the genome analysis did not extend to the chromosomal level, which constrained our ability to conduct an in-depth investigation of the genome structure and chromosome localization. Furthermore, analyzing only two single nuclei from a single variety is insufficient to establish a definitive pattern or draw conclusive results. Therefore, the findings presented here are specific to this particular variety and cannot be generalized to *A. heimuer*. Genomic data are vast and contain a wealth of information and potential. Limited by the sequencing level and research methodologies, the in-depth analysis of genomic data in *A. heimuer* is still insufficient, and how to effectively mine and use these data is still a challenge. Future research will require more advanced bioinformatics tools and methods to analyze genomic data in greater depth, revealing other biological features and potential applications of *A. heimuer*. Future genomic data research should focus on gene mining and functional identification of *A. heimuer*, such as biosynthetic pathway analysis of the active components of functional genes, disease-resistance genes, and the development of new technical means combined with multi-omics data to fully exploit its potential for practical applications.

## 5. Conclusions

This experiment fully elucidates the genome structure, genetic information, and differences of Hei29-D1 and Hei29-D2, enriching the genomic data for this species. The genome sizes of Hei29-D1 and Hei29-D2 are 47.54 Mb and 47.49 Mb, the GC contents are 56.95% and 56.99%, and the N50 values are 2.37 Mb and 4.28 Mb, respectively. The two strains share 12,362 common genes, while Hei29-D1 has 223 unique genes and Hei29-D2 has 228. The genome annotation results effectively elucidate the food and medicinal value of *A. heimuer* and partially explain why its traits are stable and less prone to degradation. These findings will promote research into the mechanisms of strain degradation, support the development and utilization of genetic breeding and active substances, and provide a strong theoretical foundation for the development of its medicinal value. The investigation of co-unique genes offers precise molecular tools and strategies for gene editing, genetic modification, and breeding, thereby enhancing the efficiency of improving the *A. heimuer* species.

## Figures and Tables

**Table 1:** Repeated sequences of Hei29.

Sample	Type	Number of Elements	Length Occupied	Percentage of Sequence
Hei29-D1	SINEs	22	7987	0.02
	LINEs	612	786,037	1.65
	LTR elements	1031	1,777,874	3.74
	DNA elements	67	20,390	0.04
	Unclassified	1843	1,409,131	2.96
Hei29-D2	SINEs	16	7460	0.02
	LINEs	397	494,891	1.04
	LTR elements	918	1,798,866	3.97
	DNA elements	217	166,481	0.35
	Unclassified	1667	1,268,699	2.67

## Data Availability

The data presented in this study are openly available at NCBI at http://www.ncbi.nlm.nih.gov/bioproject/1181141, accessed on 2 November 2024 and http://www.ncbi.nlm.nih.gov/bioproject/1181138, accessed on 2 November 2024, reference numbers PRJNA1181138 and PRJNA1181141.

## Supplementary Materials

The following are available online at https://www.mdpi.com/article/10.3390/jof11020122/s1, Figure S1. Quality control of Hei29D1: A. sequencing data length distribution (PacBio data); B. reads sequencing quality distribution map (Illumina data); C. reads base type percentage statistical map (Illumina data); D. reads average quality distribution map (Illumina data). Figure S2. Quality control of Hei29D1: A. sequencing data length distribution (PacBio data); B. reads sequencing quality distribution map (Illumina data); C. reads base type percentage statistical map (Illumina data); D. reads average quality distribution map (Illumina data). Figure S3. Statistical plot of correlation analysis between GC content and depth: A. Hei29-D1; B. Hei29-D2. Figure S4. KEGG pathway of Hei29: A. fluid shear stress and atherosclerosis KEGG pathway of Hei29; B. Alzheimer’s disease KEGG pathway of Hei29 (genes present in Hei29 and the pathway are highlighted in red). Figure S5. Phytoene synthase gene of Hei29: A. terpene gene clusters in Hei29-D1 genomes (g894); B. biosynthetic gene clusters in Hei29 genomes (911183). Table S1. Clean data of Hei29. Table S2. GO and gene distribution of Hei29-D1. Table S3. GO and gene distribution of Hei29-D2. Table S4. The biosynthetic gene cluster of Hei29. Table S5. The UGPase-related genes of Hei29.
