# Supplementary material for: Comparative Genomic Analysis of Two Monokaryons of Auricularia heimuer Hei29
Source: J Fungi (Basel). 2025 Feb 6;11(2):122. doi: 10.3390/jof11020122 (PMC11856363; doi:10.3390/jof11020122)
Supplement: Supplementary file 1 [file jof-11-00122-s001.zip › Hei29-D1.Repeat.html]

 


 


 

 [ close all hsps ] 
 [ open all hsps ] 
 
 
  
                                          ----position in query----                                    --position in repeat--
            %      %      %      query                                       C   matching            repeat           (left)     end      begin    linkage 
  +    score     div.   del.   ins.    sequence           begin      end      (left)     +   repeat              class/family      begin     end     (left)    id/graphic  
 
 
 
  +      24   12.3  5.7  0.0  contig10_pilon     1817    1869 (1813010) +  (AAGCTTG)n         Simple_repeat        1     56     (0)     1     
 
 ANNOTATION EVIDENCE: 
    24  12.31 5.66 0.00  contig10_pilon     1817    1869   1813010 +  (AAGCTTG)n         Simple_repeat        1     56       0      
24 12.31 5.66 0.00 contig10_pilon 1817 1869 (1813010) (AAGCTTG)n#Simple_repeat 1 56 (0) m_b495s252i0

  contig10_pilo       1817 AAGCATAAATCCTGAAGCTTGAAGCTTG-AGCTTGCA-CTCGAAGCTTGA 1864
                               v i  v i                -      v -  i         
  (AAGCTTG)n#Si          1 AAGCTTGAAGCTTGAAGCTTGAAGCTTGAAGCTTGAAGCTTGAAGCTTGA 50

  contig10_pilo       1865 A-CTTG 1869
                            -    
  (AAGCTTG)n#Si         51 AGCTTG 56

Matrix = Unknown
Transitions / transversions = 1.00 (3/3)
Gap_init rate = 0.06 (3 / 52), avg. gap size = 1.00 (3 / 3)

 
 

 
 
   +      12    5.5  0.0  0.0  contig10_pilon    21822   21840 (1793039) +  (CTG)n             Simple_repeat        1     19     (0)     2     
 
 ANNOTATION EVIDENCE: 
    12   5.48 0.00 0.00  contig10_pilon    21822   21840   1793039 +  (CTG)n             Simple_repeat        1     19       0      
12 5.48 0.00 0.00 contig10_pilon 21822 21840 (1793039) (CTG)n#Simple_repeat 1 19 (0) m_b495s252i1

  contig10_pilo      21822 CTGCTGCTGCTGCTTCTGC 21840
                                         v    
  (CTG)n#Simple          1 CTGCTGCTGCTGCTGCTGC 19

Matrix = Unknown
Transitions / transversions = 0.00 (0/1)
Gap_init rate = 0.00 (0 / 18), avg. gap size = 0.0 (0 / 0)

 
 
 
 
 
  +      14   17.4  1.9  8.0  contig10_pilon    25324   25376 (1789503) +  (GCGCTG)n          Simple_repeat        1     50     (0)     3     
 
 ANNOTATION EVIDENCE: 
    14  17.37 1.89 8.00  contig10_pilon    25324   25376   1789503 +  (GCGCTG)n          Simple_repeat        1     50       0      
14 17.37 1.89 8.00 contig10_pilon 25324 25376 (1789503) (GCGCTG)n#Simple_repeat 1 50 (0) m_b495s252i2

  contig10_pilo      25324 GCGTCTTGCGCGTGCGCGCTGGCGCTGGTGCTGCCATTCGGCTCCGGCGC 25373
                              -  v    -  -             i    v ii -   v i     
  (GCGCTG)n#Sim          1 GCG-CTGGCGC-TG-GCGCTGGCGCTGGCGCTGGCGCT-GGCGCTGGCGC 46

  contig10_pilo      25374 -GGC 25376
                           -   
  (GCGCTG)n#Sim         47 TGGC 50

Matrix = Unknown
Transitions / transversions = 1.33 (4/3)
Gap_init rate = 0.10 (5 / 52), avg. gap size = 1.00 (5 / 5)

 
 

 
 
   +   10632    9.1  0.3  0.2  contig10_pilon    27070   28852 (1786027) C  rnd-4_family-763   LTR/Gypsy          (0)   3908    2316     4     
 
 ANNOTATION EVIDENCE: 
  2071  13.18 0.00 0.55  contig10_pilon    27070   27435   1787444 C  rnd-4_family-763   LTR/Gypsy            3    366    3542      
2071 13.18 0.00 0.55 contig10_pilon 27070 27435 (1787444) C rnd-4_family-763#LTR/Gypsy (3542) 366 3 m_b495s001i0

  contig10_pilo      27070 TGTTGCGGTTTTCGTCGGTTCCCTCCTCCCCCGCTACCTCATTTCGCCGT 27119
                                    i        v                iii            
C rnd-4_family-        366 TGTTGCGGTCTTCGTCGGGTCCCTCCTCCCCCGCTGTTTCATTTCGCCGT 317

  contig10_pilo      27120 CCCATGGTCCTCGCACTTCATTTGGGCGCCCCAGATTCCTTTTGCCCGAT 27169
                             ?i   i      i i  ?iii        - i i     vi     ii
C rnd-4_family-        316 CCNGTGGCCCTCGCGCCTCNCCCGGGCGCCC-AAACTCCTTGCGCCCGGC 268

  contig10_pilo      27170 CCCGCAGCCCCTCCGTCGGCGCGTCCTATCGGTCTCCCCGCCTGGCGGGC 27219
                                i      i             v v       i    ii       
C rnd-4_family-        267 CCCGCGGCCCCTTCGTCGGCGCGTCCAAGCGGTCTCTCCGCTCGGCGGGC 218

  contig10_pilo      27220 GTTTCGGCCTCTTCCCAGGAGAAGGGTAATACGACGGGATGTTTCTGGTT 27269
                           iii    i                       i                  
C rnd-4_family-        217 ACCTCGGTCTCTTCCCAGGAGAAGGGTAATATGACGGGATGTTTCTGGTT 168

  contig10_pilo      27270 TGGTTGGGCTCTATTTATCCCGAGTTTCTTGTCTCGCCGAGGGCCTCCCC 27319
                                                 i     i           v i- v    
C rnd-4_family-        167 TGGTTGGGCTCTATTTATCCCGGGTTTCCTGTCTCGCCGACGA-CGCCCC 119

  contig10_pilo      27320 GGCGGTCTCCCTCGGCTTGATCTGTGGCGCCCGCATGACACTCGGCCGGT 27369
                                    ii     i                 i     vi   i    
C rnd-4_family-        118 GGCGGTCTCTTTCGGCCTGATCTGTGGCGCCCGCGTGACAACCGGTCGGT 69

  contig10_pilo      27370 GGTGCAATTGGTCCGCGGGCCCCCTCGGCCTTGATTCCGTAGTATATATA 27419
                             i                v             v                
C rnd-4_family-         68 GGCGCAATTGGTCCGCGGGGCCCCTCGGCCTTGCTTCCGTAGTATATATA 19

  contig10_pilo      27420 CCTGGGCCGGAGCCCC 27435
                           i        vv     
C rnd-4_family-         18 TCTGGGCCGTCGCCCC 3

Matrix = 20p53g.matrix
Kimura (with divCpGMod) = 12.72
Transitions / transversions = 3.36 (37/11)
Gap_init rate = 0.01 (2 / 365), avg. gap size = 1.00 (2 / 2)

 10632   8.57 0.38 0.13  contig10_pilon    27264   28852   1786027 C  rnd-4_family-763   LTR/Gypsy         2316   3908       0      
10632 8.57 0.38 0.13 contig10_pilon 27264 28852 (1786027) C rnd-4_family-763#LTR/Gypsy (0) 3908 2316 m_b495s001i1

  contig10_pilo      27264 CTGGTTTGGTTGGGCTCTATTTATCCCGAGTTTCTTGTCTCGCCGAGGGC 27313
                            i                          i     i           v i 
C rnd-4_family-       3908 CCGGTTTGGTTGGGCTCTATTTATCCCGGGTTTCCTGTCTCGCCGACGAC 3859

  contig10_pilo      27314 CTCCCCGGCGGTCTCCCTCGGCTTGATCTGTGGCGCCCGCATGACACTCG 27363
                           v-             ii     i                 i     vi  
C rnd-4_family-       3858 G-CCCCGGCGGTCTCTTTCGGCCTGATCTGTGGCGCCCGCGTGACAACCG 3810

  contig10_pilo      27364 GCCGGTGGTGCAATTGGTCCGCGGGCCCCCTCGGCCTTGATTCCGTAGTA 27413
                            i      i                v             v          
C rnd-4_family-       3809 GTCGGTGGCGCAATTGGTCCGCGGGGCCCCTCGGCCTTGCTTCCGTAGTA 3760

  contig10_pilo      27414 TATATACCTGGGCCGGAGCCCCCTCTTGTACCTTAGGTTTGGTCTGGTCT 27463
                                 i        vv     -                           
C rnd-4_family-       3759 TATATATCTGGGCCGTCGCCCC-TCTTGTACCTTAGGTTTGGTCTGGTCT 3711

  contig10_pilo      27464 ATCATACCACTTATCTGCTCCTCCCTCTCCTCACTCCTCCCTACCCCTCG 27513
                                   i      i  v           i     v     v       
C rnd-4_family-       3710 ATCATACCGCTTATCCGCGCCTCCCTCTCCCCACTCATCCCTTCCCCTCG 3661

  contig10_pilo      27514 GGTGTCGTCTCGGAGAGTGATATAGCAGTCACCGTGACATAAGACCAAAC 27563
                             i                                               
C rnd-4_family-       3660 GGCGTCGTCTCGGAGAGTGATATAGCAGTCACCGTGACATAAGACCAAAC 3611

  contig10_pilo      27564 CTACCCTCCACCCCGACGCTCGAGATCCCCACACCCCTTACCCACCACCC 27613
                                    vi                    i         i        
C rnd-4_family-       3610 CTACCCTCCCTCCCGACGCTCGAGATCCCCATACCCCTTACTCACCACCC 3561

  contig10_pilo      27614 TTCCAACCCGGGCGAACCGTTCGGTCGGAGTCGCTCAGTCCAGGAAGGAA 27663
                                 i                     v  i    i             
C rnd-4_family-       3560 TTCCAATCCGGGCGAACCGTTCGGTCGGCGTTGCTCGGTCCAGGAAGGAA 3511

  contig10_pilo      27664 TCCGACCCCTCAGACCGGCTAGGAGACCCCTCGCGTCAACGAGGACCCGT 27713
                                      i                         i            
C rnd-4_family-       3510 TCCGACCCCTCGGACCGGCTAGGAGACCCCTCGCGTCGACGAGGACCCGT 3461

  contig10_pilo      27714 CGCGACCGCCGCGCCCAATCATCGGCCCACCAGGACCCTGCAGGACCCAC 27763
                                            v                                
C rnd-4_family-       3460 CGCGACCGCCGCGCCCACTCATCGGCCCACCAGGACCCTGCAGGACCCAC 3411

  contig10_pilo      27764 CCTCCGGACGCCGAGCCTTGCCCACTGGCCTTCTGGTCAGGCACTTAGCC 27813
                                                                        i    
C rnd-4_family-       3410 CCTCCGGACGCCGAGCCTTGCCCACTGGCCTTCTGGTCAGGCACTCAGCC 3361

  contig10_pilo      27814 AACGAGGACCCCTCCAGACCCATACGGTCGTCAAATCCGAAAACTCATTC 27863
                                                                     i       
C rnd-4_family-       3360 AACGAGGACCCCTCCAGACCCATACGGTCGTCAAATCCGAAAGCTCATTC 3311

  contig10_pilo      27864 CATCACCGGCTCCTGATCTCTCCCCTCCATCAGAGTCCGGTCATTCTCGG 27913
                                                  i  i        i        i v   
C rnd-4_family-       3310 CATCACCGGCTCCTGATCTCTCCTCTTCATCAGAGCCCGGTCATCCGCGG 3261

  contig10_pilo      27914 CAAGACCACCTCCCC---AAGCCCTACTTGCCAAACGCGCCCGTTGCGCG 27960
                            i        i    ---          i   i          i      
C rnd-4_family-       3260 CGAGACCACCCCCCCCACAAGCCCTACTCGCCGAACGCGCCCGCTGCGCG 3211

  contig10_pilo      27961 TCCCT-CCCCTCGGTCAACCGCTAGACCTCCCACAGCCAACCCCTCTCTT 28009
                               i-              v   i         i   i          i
C rnd-4_family-       3210 TCCCCACCCCTCGGTCAACCTCTAAACCTCCCACGGCCGACCCCTCTCTC 3161

  contig10_pilo      28010 CGGAACCTCACCCTCCGCGCACCCCGAGAGCAGGTTTCCCCCCCC-ATGG 28058
                            i                   i          i   i        -i   
C rnd-4_family-       3160 CAGAACCTCACCCTCCGCGCATCCCGAGAGCAAGTTCCCCCCCCCCGTGG 3111

  contig10_pilo      28059 TGCGCGCGGCGCATCCACACCATCTTCCTCGGATCGTGACACCCATCACC 28108
                                         i    i                    v       i 
C rnd-4_family-       3110 TGCGCGCGGCGCATTCACATCATCTTCCTCGGATCGTGACCCCCATCATC 3061

  contig10_pilo      28109 CCCACGCATCAACGCTTACGACACATACACCATATAATCCGGACTGGCCG 28158
                                                                      ii     
C rnd-4_family-       3060 CCCACGCATCAACGCTTACGACACATACACCATATAATCCGGATCGGCCG 3011

  contig10_pilo      28159 CCAGACCGACGCCTCTTTCTTCCCCTTTCCCCGACACGCCCCTT-GTTCG 28207
                                              ii   i                   -     
C rnd-4_family-       3010 CCAGACCGACGCCTCTTTCCCCCCTTTTCCCCGACACGCCCCTTTGTTCG 2961

  contig10_pilo      28208 GACCGAACGCCCACAAAATGTCCCAGTACGACGAGACCATGGTTAACCCT 28257
                                     i                             i         
C rnd-4_family-       2960 GACCGAACGCTCACAAAATGTCCCAGTACGACGAGACCATAGTTAACCCT 2911

  contig10_pilo      28258 CGCTACATACCGGCGGCGGTCCTGGAACACTGCATACACCGAGGAGTTCC 28307
                            iv              i        v   i  i                
C rnd-4_family-       2910 CAATACATACCGGCGGCAGTCCTGGACCACCGCGTACACCGAGGAGTTCC 2861

  contig10_pilo      28308 CCAGTACCTCATTCAATGGATGGGCTTCCCTCCCGAGGACGCTAGTTGGC 28357
                           i     i   i i              i  i              i    
C rnd-4_family-       2860 TCAGTATCTCGTCCAATGGATGGGCTTTCCCCCCGAGGACGCTAGCTGGC 2811

  contig10_pilo      28358 AACTGGCCGCCGATTTGGACGGAACCGGACTCGTGGAGGAGTACCACGCC 28407
                                        ii                                   
C rnd-4_family-       2810 AACTGGCCGCCGACCTGGACGGAACCGGACTCGTGGAGGAGTACCACGCC 2761

  contig10_pilo      28408 GAACCACCGGAGCCGAGCGAGGACCAAGGCGCCGCACAGGACCCGGACCA 28457
                                         i ii                         i      
C rnd-4_family-       2760 GAACCACCGGAGCCAAATGAGGACCAAGGCGCCGCACAGGACCTGGACCA 2711

  contig10_pilo      28458 GGATGCGGACATGGAGCTCGGTGAGGACTTTGGCGACGGACCCCAGGGGC 28507
                                          v     i                  i    i i  
C rnd-4_family-       2710 GGATGCGGACATGGACCTCGGCGAGGACTTTGGCGACGGATCCCAAGAGC 2661

  contig10_pilo      28508 TGTTCGGCGAATCACCCATCAGCACCGGCGAGTCATCCCTTGCCGCCGAC 28557
                                                              i i  i         
C rnd-4_family-       2660 TGTTCGGCGAATCACCCATCAGCACCGGCGAGTCACCTCTCGCCGCCGAC 2611

  contig10_pilo      28558 GAGTCGCCCGAACTTGGAGACGCGCGCCCGGCCACACCCCCGCTGGAGCT 28607
                                      i    ii                    i       i   
C rnd-4_family-       2610 GAGTCGCCCGAGCTTGAGGACGCGCGCCCGGCCACACCTCCGCTGGGGCT 2561

  contig10_pilo      28608 CATGCCCCTTCGGGCTCGTATTCGATCACCGGACCCGGGCGACATCCAGC 28657
                              v        v        i  i  i          i     i  i  
C rnd-4_family-       2560 CATCCCCCTTCGCGCTCGTATCCGGTCGCCGGACCCGGACGACACCCGGC 2511

  contig10_pilo      28658 TCAACATGTCCATGCTCGTGGCCCCGGCCCAACCGTTCTTCTGGATGCTA 28707
                             i    vv                vv                       
C rnd-4_family-       2510 TCGACATCGCCATGCTCGTGGCCCCTCCCCAACCGTTCTTCTGGATGCTA 2461

  contig10_pilo      28708 GACCGCTTCACTCACTACGGCCGCTTTGCTGCCCCGAGACATCTCCCGCT 28757
                                                     i                       
C rnd-4_family-       2460 GACCGCTTCACTCACTACGGCCGCTTCGCTGCCCCGAGACATCTCCCGCT 2411

  contig10_pilo      28758 CGATGTCTCGCGTTACTGGGTACTCCCAAATGCAGTCTGTGACGACTGGG 28807
                               i                      i vi iv  vi ii i   i i 
C rnd-4_family-       2410 CGATATCTCGCGTTACTGGGTACTCCCGACCGTCGTGCGCAATGACCGAG 2361

  contig10_pilo      28808 GACGACGGGCTCGCATGGAGCTGGTGGTCTTCTCGTTCCCGCCCC 28852
                            v             iv     i                 i  i 
C rnd-4_family-       2360 GCCGACGGGCTCGCACCGAGCTAGTGGTCTTCTCGTTCCCACCTC 2316

Matrix = 20p53g.matrix
Kimura (with divCpGMod) = 7.33
Transitions / transversions = 3.53 (106/30)
Gap_init rate = 0.00 (6 / 1588), avg. gap size = 1.33 (8 / 6)

 
 
 
 
 
  +    2717    1.3  0.0  0.0  contig10_pilon    31225   31704 (1783175) C  rnd-4_family-763   LTR/Gypsy       (3542)    366       1     5     
 
 ANNOTATION EVIDENCE: 
  2717   2.19 0.00 0.00  contig10_pilon    31225   31590   1783289 C  rnd-4_family-763   LTR/Gypsy            1    366    3542      
2717 2.19 0.00 0.00 contig10_pilon 31225 31590 (1783289) C rnd-4_family-763#LTR/Gypsy (3542) 366 1 m_b495s001i2

  contig10_pilo      31225 TGTTGCGGTCTTCGTCGGGCCCCTCCTCCCCCGCTGTTTCATTTCGCCGT 31274
                                              i                              
C rnd-4_family-        366 TGTTGCGGTCTTCGTCGGGTCCCTCCTCCCCCGCTGTTTCATTTCGCCGT 317

  contig10_pilo      31275 CCCGTGGCCCTCGCACCTTGTCCGGGCGCCCAAACTCCTTGCGCCCGGCC 31324
                             ?           i   i?i                             
C rnd-4_family-        316 CCNGTGGCCCTCGCGCCTCNCCCGGGCGCCCAAACTCCTTGCGCCCGGCC 267

  contig10_pilo      31325 CCGCGGCCCTTTCGTCGGCGCGTCCAAGCGGTCTCTCCGCTCGGCGAGCA 31374
                                    i                                    i   
C rnd-4_family-        266 CCGCGGCCCCTTCGTCGGCGCGTCCAAGCGGTCTCTCCGCTCGGCGGGCA 217

  contig10_pilo      31375 CCTCGGTCTCTTCCCAGGAGAATGGTAATATGACGGGATGTTTCTGGTTT 31424
                                                 v                           
C rnd-4_family-        216 CCTCGGTCTCTTCCCAGGAGAAGGGTAATATGACGGGATGTTTCTGGTTT 167

  contig10_pilo      31425 GGTTGGGCTCTATTTATCCCGGGTTTCCTGTCTCGCCGACGACGCCCCGG 31474
                                                                             
C rnd-4_family-        166 GGTTGGGCTCTATTTATCCCGGGTTTCCTGTCTCGCCGACGACGCCCCGG 117

  contig10_pilo      31475 CGGTCTCTTTCGGCCTGATCTGTGGCGCCCGCGTGACAACCGGTCGGTGG 31524
                                                                             
C rnd-4_family-        116 CGGTCTCTTTCGGCCTGATCTGTGGCGCCCGCGTGACAACCGGTCGGTGG 67

  contig10_pilo      31525 CGCAATTGGTCCGCGGGGCCCCTCGGCCTTGCTTCCGTAGTATATATATA 31574
                                                                            v
C rnd-4_family-         66 CGCAATTGGTCCGCGGGGCCCCTCGGCCTTGCTTCCGTAGTATATATATC 17

  contig10_pilo      31575 TGGGCCGTCGCCCCTC 31590
                                           
C rnd-4_family-         16 TGGGCCGTCGCCCCTC 1

Matrix = 20p53g.matrix
Kimura (with divCpGMod) = 1.98
Transitions / transversions = 3.00 (6/2)
Gap_init rate = 0.00 (0 / 365), avg. gap size = 0.0 (0 / 0)

  2265   0.70 0.00 0.00  contig10_pilon    31418   31704   1783175 C  rnd-4_family-763   LTR/Gypsy         3622   3908       0      
2265 0.70 0.00 0.00 contig10_pilon 31418 31704 (1783175) C rnd-4_family-763#LTR/Gypsy (0) 3908 3622 m_b495s001i3

  contig10_pilo      31418 CTGGTTTGGTTGGGCTCTATTTATCCCGGGTTTCCTGTCTCGCCGACGAC 31467
                            i                                                
C rnd-4_family-       3908 CCGGTTTGGTTGGGCTCTATTTATCCCGGGTTTCCTGTCTCGCCGACGAC 3859

  contig10_pilo      31468 GCCCCGGCGGTCTCTTTCGGCCTGATCTGTGGCGCCCGCGTGACAACCGG 31517
                                                                             
C rnd-4_family-       3858 GCCCCGGCGGTCTCTTTCGGCCTGATCTGTGGCGCCCGCGTGACAACCGG 3809

  contig10_pilo      31518 TCGGTGGCGCAATTGGTCCGCGGGGCCCCTCGGCCTTGCTTCCGTAGTAT 31567
                                                                             
C rnd-4_family-       3808 TCGGTGGCGCAATTGGTCCGCGGGGCCCCTCGGCCTTGCTTCCGTAGTAT 3759

  contig10_pilo      31568 ATATATATGGGCCGTCGCCCCTCTTGTACCTTAGGTTTGGTCTGGTCTAT 31617
                                 v                                           
C rnd-4_family-       3758 ATATATCTGGGCCGTCGCCCCTCTTGTACCTTAGGTTTGGTCTGGTCTAT 3709

  contig10_pilo      31618 CATACCGCTTATCCGCGCCTCCCTCTCCCCACTCATCCCTTCCCCTCGGG 31667
                                                                             
C rnd-4_family-       3708 CATACCGCTTATCCGCGCCTCCCTCTCCCCACTCATCCCTTCCCCTCGGG 3659

  contig10_pilo      31668 CGTCGTCTCGGAGAGTGATATAGCAGTCACCGTGACA 31704
                                                                
C rnd-4_family-       3658 CGTCGTCTCGGAGAGTGATATAGCAGTCACCGTGACA 3622

Matrix = 20p53g.matrix
Kimura (with divCpGMod) = 0.38
Transitions / transversions = 1.00 (1/1)
Gap_init rate = 0.00 (0 / 286), avg. gap size = 0.0 (0 / 0)

 
 

 
 
   +      15   17.0  0.0  0.0  contig10_pilon    33609   33641 (1781238) +  (CAGCTG)n          Simple_repeat        1     33     (0)     6     
 
 ANNOTATION EVIDENCE: 
    15  16.97 0.00 0.00  contig10_pilon    33609   33641   1781238 +  (CAGCTG)n          Simple_repeat        1     33       0      
15 16.97 0.00 0.00 contig10_pilon 33609 33641 (1781238) (CAGCTG)n#Simple_repeat 1 33 (0) m_b495s252i3

  contig10_pilo      33609 CAGGTGCGGCTGGATCTGCAGCTGCAGCTGGAG 33641
                              v   i    v v               v  
  (CAGCTG)n#Sim          1 CAGCTGCAGCTGCAGCTGCAGCTGCAGCTGCAG 33

Matrix = Unknown
Transitions / transversions = 0.25 (1/4)
Gap_init rate = 0.00 (0 / 32), avg. gap size = 0.0 (0 / 0)

 
 
 
 
 
  +   36459    1.7  1.1  0.6  contig10_pilon    34885   36679 (1778200) +  rnd-4_family-738   LTR/Gypsy            1   1820  (4634)     7     
 
 ANNOTATION EVIDENCE: 
 36459   1.71 1.10 0.62  contig10_pilon    34885   36679   1778200 +  rnd-4_family-738   LTR/Gypsy            1   1820    4634      
36459 1.71 1.10 0.62 contig10_pilon 34885 36679 (1778200) rnd-4_family-738#LTR/Gypsy 1 1820 (4634) m_b495s001i4

  contig10_pilo      34885 CACCTCAGGTAGCTGCAACCGGGTGACGCTAACCCTATTTTACCCCCCCG 34934
                                                    i                        
  rnd-4_family-          1 CACCTCAGGTAGCTGCAACCGGGTGGCGCTAACCCTATTTTACCCCCCCG 50

  contig10_pilo      34935 GAAAATAGGGTAGCGTCAGGCCCGCGCCCGCGCCCGCGCCTGTTGCGGGT 34984
                                          i                                  
  rnd-4_family-         51 GAAAATAGGGTAGCGCCAGGCCCGCGCCCGCGCCCGCGCCTGTTGCGGGT 100

  contig10_pilo      34985 GGGTCGGTGGGACTGCAGGTGGTGGTGCGGGTTTCTTTGCGGCTGCCTGT 35034
                                                                             
  rnd-4_family-        101 GGGTCGGTGGGACTGCAGGTGGTGGTGCGGGTTTCTTTGCGGCTGCCTGT 150

  contig10_pilo      35035 ATGCTAGTGCGGTTCGCTCTGCGGGTCCTGGAATGCTGCTGCGGGTTGCT 35084
                                                                             
  rnd-4_family-        151 ATGCTAGTGCGGTTCGCTCTGCGGGTCCTGGAATGCTGCTGCGGGTTGCT 200

  contig10_pilo      35085 CTGCGGGTGTGCGAATGCTGCTGCGGTCCACGCTGCGGGTTGCTCTGCGG 35134
                                                        i          ----------
  rnd-4_family-        201 CTGCGGGTGTGCGAATGCTGCTGCGGTCCGCGCTGCGGGT---------- 240

  contig10_pilo      35135 GTGTGCGAATGCTGCTGCGGTCCACGCTGCGGGTGTGCAGGTG------- 35177
                           --    iii     v     v ii ii        i  v    -------
  rnd-4_family-        241 --GTGCAGGTGCTGGTGCGGGCTGCATTGCGGGTGCGCTGGTGGTGCTGC 288

  contig10_pilo      35178 --CTGG---TGCGGGCTGCATTGCGGGTGCGCTGGTGGTGCTGCGGTTGG 35222
                           --i   ---     v i  ii           v    v         iv 
  rnd-4_family-        289 GGTTGGCATTGCGGTCCGCGCTGCGGGTGCGCGGGTGCTGCTGCGGTCCG 338

  contig10_pilo      35223 CATTGCAGGCCCACGGATGCTGCCACGGGTTGGCCTGCGGGTGTGCGGGT 35272
                                 i     i           i                  i      
  rnd-4_family-        339 CATTGCGGGCCCGCGGATGCTGCCGCGGGTTGGCCTGCGGGTGCGCGGGT 388

  contig10_pilo      35273 GCTGCCGCGGTTTGCAATGCGGTCCGCACTGCGGGTGCAACGGTGCTGGT 35322
                                                      i          i           
  rnd-4_family-        389 GCTGCCGCGGTTTGCAATGCGGTCCGCGCTGCGGGTGCGACGGTGCTGGT 438

  contig10_pilo      35323 GCGGTTCCCATTGCGGTCCGCGCTGCGGGTGCGCGTGCACAGGTGCTGCT 35372
                                   -                             i           
  rnd-4_family-        439 GCGGTTCC-ATTGCGGTCCGCGCTGCGGGTGCGCGTGCGCAGGTGCTGCT 487

  contig10_pilo      35373 GCGGGTCGCATTGCGGGTGCGCTGGTGGCGCTGCGGATTGCATTGCGGTC 35422
                                                                             
  rnd-4_family-        488 GCGGGTCGCATTGCGGGTGCGCTGGTGGCGCTGCGGATTGCATTGCGGTC 537

  contig10_pilo      35423 CGCTCTACAGGTGCACAGGTACCCCCGCAGGTACTGGTGCGCATTTCATT 35472
                                         i                           i       
  rnd-4_family-        538 CGCTCTACAGGTGCGCAGGTACCCCCGCAGGTACTGGTGCGCGTTTCATT 587

  contig10_pilo      35473 GCGGGTGCGCTGGTGACGCTGCGGGTGGCATTGCGGTCCACGCTGCGGGT 35522
                                                        i         i          
  rnd-4_family-        588 GCGGGTGCGCTGGTGACGCTGCGGGTGGCGTTGCGGTCCGCGCTGCGGGT 637

  contig10_pilo      35523 GCGCTGCCTTCCGGCACATTCCGGTACTGCCCGGAATGGTGCCGCCGCCT 35572
                               i   v                  i        i             
  rnd-4_family-        638 GCGCCGCCGTCCGGCACATTCCGGTACCGCCCGGAACGGTGCCGCCGCCT 687

  contig10_pilo      35573 CCGCCACCGCCGGAAACGGCCGGAAATCGCCGACCGACGTCGCCGCCGCC 35622
                                                                             
  rnd-4_family-        688 CCGCCACCGCCGGAAACGGCCGGAAATCGCCGACCGACGTCGCCGCCGCC 737

  contig10_pilo      35623 GCCGCTGCGGTCGGAAATCGCCGGAACCGTCGCCACTGCCGCCACCGGTC 35672
                                                               i             
  rnd-4_family-        738 GCCGCTGCGGTCGGAAATCGCCGGAACCGTCGCCACCGCCGCCACCGGTC 787

  contig10_pilo      35673 GTCACCGGCGCTTA-TTTATGAAAAAAAAACTTTTTTACCCCAAAACACA 35721
                                         -                                   
  rnd-4_family-        788 GTCACCGGCGCTTAATTTATGAAAAAAAAACTTTTTTACCCCAAAACACA 837

  contig10_pilo      35722 AAATGTTCCAATTGATGTCCTTCATGTTATTATACATATAAAAGTCTTGC 35771
                                                                             
  rnd-4_family-        838 AAATGTTCCAATTGATGTCCTTCATGTTATTATACATATAAAAGTCTTGC 887

  contig10_pilo      35772 TAACTATAATACACAGGAATTGAATTCTGAATGGATTAAAGCTAAATGCA 35821
                                                                             
  rnd-4_family-        888 TAACTATAATACACAGGAATTGAATTCTGAATGGATTAAAGCTAAATGCA 937

  contig10_pilo      35822 GCTGCATTCACTTCTGCAGCAACCGCACCTGCAGCCGCACCTGCAACCGC 35871
                                                                             
  rnd-4_family-        938 GCTGCATTCACTTCTGCAGCAACCGCACCTGCAGCCGCACCTGCAACCGC 987

  contig10_pilo      35872 ACCTGCGCTTGCGCCTGCAGCACATGCGGTCGCACCTGCAGGGATATCTC 35921
                                                                             
  rnd-4_family-        988 ACCTGCGCTTGCGCCTGCAGCACATGCGGTCGCACCTGCAGGGATATCTC 1037

  contig10_pilo      35922 AAAAGAGAACAGACTTGAGACCTTTGTGACGAATTACTAAGCTTAGAGCA 35971
                                                                             
  rnd-4_family-       1038 AAAAGAGAACAGACTTGAGACCTTTGTGACGAATTACTAAGCTTAGAGCA 1087

  contig10_pilo      35972 AAGTGTGCATTAACAGAGTGACCCAGAATCTTCCAGTTGGCCAAGTTTCA 36021
                                                                             
  rnd-4_family-       1088 AAGTGTGCATTAACAGAGTGACCCAGAATCTTCCAGTTGGCCAAGTTTCA 1137

  contig10_pilo      36022 AAAAGCCCGGACCCTTGTAGCATGCTCTAACTTGCTCCATGTGCTGGATG 36071
                                                                             
  rnd-4_family-       1138 AAAAGCCCGGACCCTTGTAGCATGCTCTAACTTGCTCCATGTGCTGGATG 1187

  contig10_pilo      36072 TAGCAGCAGCCGCAGCCGCAACCGCACCCGCGCCCGCGCCTGCACCCGCA 36121
                                                                             
  rnd-4_family-       1188 TAGCAGCAGCCGCAGCCGCAACCGCACCCGCGCCCGCGCCTGCACCCGCA 1237

  contig10_pilo      36122 CCCGCAGCTGCGACTTGCAATCCGCCCGGTTACCGCACACAGGCATCCAA 36171
                                                                             
  rnd-4_family-       1238 CCCGCAGCTGCGACTTGCAATCCGCCCGGTTACCGCACACAGGCATCCAA 1287

  contig10_pilo      36172 TTGTAAGCTTCAGATACACCACAGATCAATGAGGAACGACCATTGGAGAC 36221
                                                               i      i      
  rnd-4_family-       1288 TTGTAAGCTTCAGATACACCACAGATCAATGAGGAATGACCATCGGAGAC 1337

  contig10_pilo      36222 ATGTGCCAAGTTTTCAGCGTTTCCGGAGCTTTGTGGAATATTCTGCTGCA 36271
                                                                             
  rnd-4_family-       1338 ATGTGCCAAGTTTTCAGCGTTTCCGGAGCTTTGTGGAATATTCTGCTGCA 1387

  contig10_pilo      36272 CTCTGTGCTGTTGGTGGAAGAATACTTCCGAACCTCGGACATCGGACTTT 36321
                                                                             
  rnd-4_family-       1388 CTCTGTGCTGTTGGTGGAAGAATACTTCCGAACCTCGGACATCGGACTTT 1437

  contig10_pilo      36322 GCCGCAGTGCAAGCCGTCCAATGATTCACATGCTATACATCGCATCCGCA 36371
                                                                         v   
  rnd-4_family-       1438 GCCGCAGTGCAAGCCGTCCAATGATTCACATGCTATACATCGCATCGGCA 1487

  contig10_pilo      36372 ATACCATCAGCCTGTATACTGTCCAAGTAGAACCGCGGTGGCTGATGGTT 36421
                                             ?                       i       
  rnd-4_family-       1488 ATACCATCAGCCTGTATANTGTCCAAGTAGAACCGCGGTGGCCGATGGTT 1537

  contig10_pilo      36422 ACAAGCACCCACACCCGCACCCGCGCCCGCGCCTGCACCCACGCCCGCGC 36471
                               ------ ???????????????????????????????????????
  rnd-4_family-       1538 ACAA------ANNNNNNNNNNNNNNNNNNNNNNNNNNNNNNNNNNNNNNN 1581

  contig10_pilo      36472 CCGCGCCTGCAACCGCGCCCGCAACCGCACCTGCAAGCATTCACTACTAT 36521
                           ????         i                                    
  rnd-4_family-       1582 NNNNGCCTGCAACTGCGCCCGCAACCGCACCTGCAAGCATTCACTACTAT 1631

  contig10_pilo      36522 GATTTTATTTTTCATCATCCATGGACCCGCACCCGCACCCGCACCCGCAC 36571
                                                                     i     i 
  rnd-4_family-       1632 GATTTTATTTTTCATCATCCATGGACCCGCACCCGCACCCGCGCCCGCGC 1681

  contig10_pilo      36572 CCACGCCCGCACCTGCAACCGCGCCCGCGCCTGCAACCGCACCTGCAAGC 36621
                            ii  ?    i     i-------                          
  rnd-4_family-       1682 CTGCGNCCGCGCCTGCG-------CCGCGCCTGCAACCGCACCTGCAAGC 1724

  contig10_pilo      36622 A--------------TTTACTACTAAG--------------CATTTAC-- 36641
                            --------------           i--------------   ii i--
  rnd-4_family-       1725 ATTTACTACTAAGCATTTACTACTAAAATTTAGTTTTTCATCATCCATGA 1774

  contig10_pilo      36642 -----TAC---TAAAATTTAGTTTTTCATCATCCATGAACCCACAT 36679
                           -----   ---???????????????????????????????????
  rnd-4_family-       1775 ACCTGTACACNNNNNNNNNNNNNNNNNNNNNNNNNNNNNNNNNNNN 1820

Matrix = 20p53g.matrix
Kimura (with divCpGMod) = 0.99
Transitions / transversions = 5.00 (45/9)
Gap_init rate = 0.02 (34 / 1794), avg. gap size = 2.26 (77 / 34)

 
 

 
   +      47    8.1  0.0  0.0  contig10_pilon    36680   36744 (1778135) +  (CCGCAC)n          Simple_repeat        1     65     (0)     8     
 
 ANNOTATION EVIDENCE: 
    47   8.12 0.00 0.00  contig10_pilon    36680   36744   1778135 +  (CCGCAC)n          Simple_repeat        1     65       0      
47 8.12 0.00 0.00 contig10_pilon 36680 36744 (1778135) (CCGCAC)n#Simple_repeat 1 65 (0) c_b495s251i0

  contig10_pilo      36680 CCGCACCCGCACCCGCACCCATACCCGCACCCGCAACCGCAACCTCACCC 36729
                                               ii             v     v  v     
  (CCGCAC)n#Sim          1 CCGCACCCGCACCCGCACCCGCACCCGCACCCGCACCCGCACCCGCACCC 50

  contig10_pilo      36730 GCACCCGCACCCGCA 36744
                                          
  (CCGCAC)n#Sim         51 GCACCCGCACCCGCA 65

Matrix = Unknown
Transitions / transversions = 0.67 (2/3)
Gap_init rate = 0.00 (0 / 64), avg. gap size = 0.0 (0 / 0)

 
 
 
 
  +   36459    1.7  1.1  0.6  contig10_pilon    36745   39576 (1775303) +  rnd-4_family-738   LTR/Gypsy         1821   4651  (1803)     7     
 
 ANNOTATION EVIDENCE: 
 36459   1.71 1.10 0.62  contig10_pilon    36745   39576   1775303 +  rnd-4_family-738   LTR/Gypsy         1821   4651    1803      
36459 1.71 1.10 0.62 contig10_pilon 36745 39576 (1775303) rnd-4_family-738#LTR/Gypsy 1821 4651 (1803) m_b495s001i4

  contig10_pilo      36745 ATCACATCTGCAGACGCAAATACAGCTGTAGTCACTCACAGGATTGCATA 36794
                                                          v            i     
  rnd-4_family-       1821 ATCACATCTGCAGACGCAAATACAGCTGTAGACACTCACAGGATCGCATA 1870

  contig10_pilo      36795 CTCTACATGGGAACACCAGCAGTGCAGTGGAACACACCTTGTGAGCCGCC 36844
                                                                             
  rnd-4_family-       1871 CTCTACATGGGAACACCAGCAGTGCAGTGGAACACACCTTGTGAGCCGCC 1920

  contig10_pilo      36845 AGGCTCAACGGTTAGTAACAAATCACAAATTTGAGAAATCACTGGAGAAT 36894
                                                          i                  
  rnd-4_family-       1921 AGGCTCAACGGTTAGTAACAAATCACAAATTCGAGAAATCACTGGAGAAT 1970

  contig10_pilo      36895 CTACATTAGAACTCAAACACAAGCTGCCCTGTAAGCGAAAGTGACACAGG 36944
                                                                    i        
  rnd-4_family-       1971 CTACATTAGAACTCAAACACAAGCTGCCCTGTAAGCGAAAGCGACACAGG 2020

  contig10_pilo      36945 CACAAGTCTCAAAATCAGACAATGAACATCACAATTTACGATTAAATGCG 36994
                                                                             
  rnd-4_family-       2021 CACAAGTCTCAAAATCAGACAATGAACATCACAATTTACGATTAAATGCG 2070

  contig10_pilo      36995 AAGCATGTACATCAGAATAATTAAAGTCAACCCAGTTGCAATTACAGGCC 37044
                                                                             
  rnd-4_family-       2071 AAGCATGTACATCAGAATAATTAAAGTCAACCCAGTTGCAATTACAGGCC 2120

  contig10_pilo      37045 GTAGTGAAATGTGTAGTGAAATGCGGCTCAATCCGCATTAGAAATTGCAA 37094
                                      i                                      
  rnd-4_family-       2121 GTAGTGAAATGCGTAGTGAAATGCGGCTCAATCCGCATTAGAAATTGCAA 2170

  contig10_pilo      37095 GTGCAATGGATTACAAACAGCCTTACACACGGCGGTTAAATCCGGCTTGA 37144
                                                                             
  rnd-4_family-       2171 GTGCAATGGATTACAAACAGCCTTACACACGGCGGTTAAATCCGGCTTGA 2220

  contig10_pilo      37145 ACCGCATTAGAAGTTGCAAGATTAGCGCATTAGACGAGACATCACTAATG 37194
                                                              v              
  rnd-4_family-       2221 ACCGCATTAGAAGTTGCAAGATTAGCGCATTAGACCAGACATCACTAATG 2270

  contig10_pilo      37195 CTCATTACAACACAAGTGTAATAGCTTTGAGACAGCATTACAACTCACGC 37244
                                  i                                          
  rnd-4_family-       2271 CTCATTATAACACAAGTGTAATAGCTTTGAGACAGCATTACAACTCACGC 2320

  contig10_pilo      37245 CTGAAGTGCATTAGCACTCGCACTCGAAGCAACATCACAAATGCGCATTA 37294
                                 i                                           
  rnd-4_family-       2321 CTGAAGCGCATTAGCACTCGCACTCGAAGCAACATCACAAATGCGCATTA 2370

  contig10_pilo      37295 GTACAAAAATGTAATGGATGAGAGACATCATTACAAATTGCACCTGAATC 37344
                                                                             
  rnd-4_family-       2371 GTACAAAAATGTAATGGATGAGAGACATCATTACAAATTGCACCTGAATC 2420

  contig10_pilo      37345 GCATTGGCAGTTGCATTGGAACTGACCTAGTGACGTGCATTAGCTCACAG 37394
                                                                             
  rnd-4_family-       2421 GCATTGGCAGTTGCATTGGAACTGACCTAGTGACGTGCATTAGCTCACAG 2470

  contig10_pilo      37395 ATGTAATGGATTAGAAATGGGATTACAACTTCCTCAGTAATCGCATTAGA 37444
                                                                             
  rnd-4_family-       2471 ATGTAATGGATTAGAAATGGGATTACAACTTCCTCAGTAATCGCATTAGA 2520

  contig10_pilo      37445 AGACAAGTGTAATTAGATTACAACCGTCATTAGAACTCGCAGTGTACTGC 37494
                                                                             
  rnd-4_family-       2521 AGACAAGTGTAATTAGATTACAACCGTCATTAGAACTCGCAGTGTACTGC 2570

  contig10_pilo      37495 GCATTAGGCCAGAAATGTAGTACATGCCATATGTGAGAAACACTCAGGCG 37544
                                                                             
  rnd-4_family-       2571 GCATTAGGCCAGAAATGTAGTACATGCCATATGTGAGAAACACTCAGGCG 2620

  contig10_pilo      37545 CGGTCATGGATGATTCCATGCTCTTATGTAGCTGATCTCAGACTGTGAGC 37594
                           i                                                 
  rnd-4_family-       2621 TGGTCATGGATGATTCCATGCTCTTATGTAGCTGATCTCAGACTGTGAGC 2670

  contig10_pilo      37595 AAGTTTTGGTGCCTTGGGATGCAGTGGGGGGTAAGGTTTGTTTGCCTCAA 37644
                                                                             
  rnd-4_family-       2671 AAGTTTTGGTGCCTTGGGATGCAGTGGGGGGTAAGGTTTGTTTGCCTCAA 2720

  contig10_pilo      37645 CTAAGGGAAATGGGTAGAACACTACCCCATGAAGCCATGCTTCAACCGTT 37694
                                                                             
  rnd-4_family-       2721 CTAAGGGAAATGGGTAGAACACTACCCCATGAAGCCATGCTTCAACCGTT 2770

  contig10_pilo      37695 AAGCTTCAATGGTGAAAGTTGCTTGTATTGGACCTATTTAGCTTATCCCA 37744
                                                                             
  rnd-4_family-       2771 AAGCTTCAATGGTGAAAGTTGCTTGTATTGGACCTATTTAGCTTATCCCA 2820

  contig10_pilo      37745 CTCAAGAATGGTCAATAGGGTGTACTGGCGAGGCTCGGTGCAAATGCCCC 37794
                                                                             
  rnd-4_family-       2821 CTCAAGAATGGTCAATAGGGTGTACTGGCGAGGCTCGGTGCAAATGCCCC 2870

  contig10_pilo      37795 TTTTTGATGGGGAAAGAACATTGCACCCAGAGAGACCCCTCAACCCGTAT 37844
                                                                    v        
  rnd-4_family-       2871 TTTTTGATGGGGAAAGAACATTGCACCCAGAGAGACCCCTCCACCCGTAT 2920

  contig10_pilo      37845 CTTTCGCAAACCTGCACGTACAATCTGGGGAATGAATAAAGACTTGAGGC 37894
                                                         v                   
  rnd-4_family-       2921 CTTTCGCAAACCTGCACGTACAATCTGGGGTATGAATAAAGACTTGAGGC 2970

  contig10_pilo      37895 GCTTGGTGCCTTGACGAATGTCTCGCAACACTTTCTCAGTGCAACTACTT 37944
                                                                            i
  rnd-4_family-       2971 GCTTGGTGCCTTGACGAATGTCTCGCAACACTTTCTCAGTGCAACTACTC 3020

  contig10_pilo      37945 GCATTTTCGCCCCCATGAGCTCACAGACTTCGCTCCTCTCCCTCTATTCT 37994
                                                                 v           
  rnd-4_family-       3021 GCATTTTCGCCCCCATGAGCTCACAGACTTCGCTCCTCGCCCTCTATTCT 3070

  contig10_pilo      37995 CAGTCTAATGCTTTCAGGGTAGTGTATAGTGGTCACACAAAAGGCTCAGG 38044
                                   i                                         
  rnd-4_family-       3071 CAGTCTAACGCTTTCAGGGTAGTGTATAGTGGTCACACAAAAGGCTCAGG 3120

  contig10_pilo      38045 AAGTTTGAAGCTGAACGGAGCATGGGTGGTTGAGATATGACCTGCGCATG 38094
                                                                             
  rnd-4_family-       3121 AAGTTTGAAGCTGAACGGAGCATGGGTGGTTGAGATATGACCTGCGCATG 3170

  contig10_pilo      38095 TCTGGACACTGAGTTGCAGCCAGTTTCACCAAGAACTGCACATGCAACCT 38144
                                                                             
  rnd-4_family-       3171 TCTGGACACTGAGTTGCAGCCAGTTTCACCAAGAACTGCACATGCAACCT 3220

  contig10_pilo      38145 ACCCCACCTTTTGGAAACATGCCTCTCTCACCCAAGAAGATGCTCAGAGT 38194
                                      i                                      
  rnd-4_family-       3221 ACCCCACCTTTCGGAAACATGCCTCTCTCACCCAAGAAGATGCTCAGAGT 3270

  contig10_pilo      38195 TTCATATAAAATGGATCGGAGCACTTGTGGATGTGGGAGATGCATGTTAC 38244
                                      i                                      
  rnd-4_family-       3271 TTCATATAAAACGGATCGGAGCACTTGTGGATGTGGGAGATGCATGTTAC 3320

  contig10_pilo      38245 CGAGAGGGGGGGTAGGTTCATGTGCAATTCTTGGTGAAACTGGCTGCAAC 38294
                                -                                            
  rnd-4_family-       3321 CGAGA-GGGGGGTAGGTTCATGTGCAATTCTTGGTGAAACTGGCTGCAAC 3369

  contig10_pilo      38295 TCAGTGTCCAGACATGAACAGGTCATATCTCAACCACCCATGCTCCGTCC 38344
                                                                             
  rnd-4_family-       3370 TCAGTGTCCAGACATGAACAGGTCATATCTCAACCACCCATGCTCCGTCC 3419

  contig10_pilo      38345 CACTTCAAACTTCCTGAGCCTTTTGTGTGACCACTATACACTACCCTGAA 38394
                                                                             
  rnd-4_family-       3420 CACTTCAAACTTCCTGAGCCTTTTGTGTGACCACTATACACTACCCTGAA 3469

  contig10_pilo      38395 AGTGTTAGACTGAGAATAGAGGGCGAGGAGCGAAGTCTGTGAGCTCATGG 38444
                             i                                               
  rnd-4_family-       3470 AGCGTTAGACTGAGAATAGAGGGCGAGGAGCGAAGTCTGTGAGCTCATGG 3519

  contig10_pilo      38445 GGGCGAAAATGCGAGCAGTTGCACTGAGAAAGGATTAACTCATATAGTGC 38494
                                                                             
  rnd-4_family-       3520 GGGCGAAAATGCGAGCAGTTGCACTGAGAAAGGATTAACTCATATAGTGC 3569

  contig10_pilo      38495 AAGGCACAGCACAGCACAGTACTCGCATTATGTATCATATAGTGTATGCG 38544
                                                                             
  rnd-4_family-       3570 AAGGCACAGCACAGCACAGTACTCGCATTATGTATCATATAGTGTATGCG 3619

  contig10_pilo      38545 AGAGTTCAAGAGAGTTGGGCGACAGTGTATCTCTGGGTGCAATGTCCTTC 38594
                                                                            i
  rnd-4_family-       3620 AGAGTTCAAGAGAGTTGGGCGACAGTGTATCTCTGGGTGCAATGTCCTTT 3669

  contig10_pilo      38595 CTCCATCAAAAATGCGCATTTGCACCCAGCCCCCCCACCTCACTGGTTTA 38644
                                                                             
  rnd-4_family-       3670 CTCCATCAAAAATGCGCATTTGCACCCAGCCCCCCCACCTCACTGGTTTA 3719

  contig10_pilo      38645 ACATAACCCAAGCTGGACAGGCTAGAGAGGCATAATACGAGTAATTTTCA 38694
                                                                             
  rnd-4_family-       3720 ACATAACCCAAGCTGGACAGGCTAGAGAGGCATAATACGAGTAATTTTCA 3769

  contig10_pilo      38695 TTATTGAAGCTTCATGGTATATACAGTTATTGAAGGGGAATGGCTGATCC 38744
                            i                    i                           
  rnd-4_family-       3770 TCATTGAAGCTTCATGGTATATGCAGTTATTGAAGGGGAATGGCTGATCC 3819

  contig10_pilo      38745 TCCTCTCTCAGTTGAGGCAAGCAAAACTCACTTCCCCCAGCATCTGGAAT 38794
                                                                             
  rnd-4_family-       3820 TCCTCTCTCAGTTGAGGCAAGCAAAACTCACTTCCCCCAGCATCTGGAAT 3869

  contig10_pilo      38795 CATGAACACTCGTCAGGAGGATGGGATTGATATAATGAACACATGGCAGG 38844
                                                                             
  rnd-4_family-       3870 CATGAACACTCGTCAGGAGGATGGGATTGATATAATGAACACATGGCAGG 3919

  contig10_pilo      38845 ATGCAGAAGCCAAACCATGAGTTCCACATGTATCTCATGTATTACATTTT 38894
                                                                             
  rnd-4_family-       3920 ATGCAGAAGCCAAACCATGAGTTCCACATGTATCTCATGTATTACATTTT 3969

  contig10_pilo      38895 CGGCCAAATGCGCAATACACCGCGAGTTCTAATGGCATTTCTAATCAGTT 38944
                                                                             
  rnd-4_family-       3970 CGGCCAAATGCGCAATACACCGCGAGTTCTAATGGCATTTCTAATCAGTT 4019

  contig10_pilo      38945 ACACTTACTACTTCTAATGTGATTACGCCACCAATCCTAATCCATTACAT 38994
                                              i                              
  rnd-4_family-       4020 ACACTTACTACTTCTAATGCGATTACGCCACCAATCCTAATCCATTACAT 4069

  contig10_pilo      38995 TTGCTGCTAATCGACATCACTGAGTGTTCCTAATTAAATTTACTAATCCA 39044
                                                                             
  rnd-4_family-       4070 TTGCTGCTAATCGACATCACTGAGTGTTCCTAATTAAATTTACTAATCCA 4119

  contig10_pilo      39045 ATCTCGGTGCGACTTCTAATGCTAGCTCTGTTCAATTACTCCGGCGACTG 39094
                                                                             
  rnd-4_family-       4120 ATCTCGGTGCGACTTCTAATGCTAGCTCTGTTCAATTACTCCGGCGACTG 4169

  contig10_pilo      39095 TACACATTAGTGATGTTGGTACTAATGGCGACTTTGCGTATTAGTGATGT 39144
                                                                             
  rnd-4_family-       4170 TACACATTAGTGATGTTGGTACTAATGGCGACTTTGCGTATTAGTGATGT 4219

  contig10_pilo      39145 TTGTTCTAATACGACTACTAATGTGATTTGGATGCGAGGTCTAATGTCTT 39194
                                                                             
  rnd-4_family-       4220 TTGTTCTAATACGACTACTAATGTGATTTGGATGCGAGGTCTAATGTCTT 4269

  contig10_pilo      39195 CTCTGATCCATTATAGTGATGTCCTAATGTGCAGTAGTGATGCAGCTTCT 39244
                                                                             
  rnd-4_family-       4270 CTCTGATCCATTATAGTGATGTCCTAATGTGCAGTAGTGATGCAGCTTCT 4319

  contig10_pilo      39245 AATCGGACTGCCGTTGTGGTTAAGGCGCAAGTTCTAATGGTGTCACTGGT 39294
                                                                             
  rnd-4_family-       4320 AATCGGACTGCCGTTGTGGTTAAGGCGCAAGTTCTAATGGTGTCACTGGT 4369

  contig10_pilo      39295 TATTGAACTTGTGTTCTAATGCATCTTACTCCTAATGCAGTACTTGTAAG 39344
                                                                             
  rnd-4_family-       4370 TATTGAACTTGTGTTCTAATGCATCTTACTCCTAATGCAGTACTTGTAAG 4419

  contig10_pilo      39345 TTCTATTTCGGTTTAAGCCGGAATTGACCGCAGCTTGTAATGCTCTTTCT 39394
                                                                             
  rnd-4_family-       4420 TTCTATTTCGGTTTAAGCCGGAATTGACCGCAGCTTGTAATGCTCTTTCT 4469

  contig10_pilo      39395 GGCGCATCTAACTTGTGTTTCCTGACACATTTGTGAGCCGTGGTTCACTG 39444
                                                                             
  rnd-4_family-       4470 GGCGCATCTAACTTGTGTTTCCTGACACATTTGTGAGCCGTGGTTCACTG 4519

  contig10_pilo      39445 CGACTTCGAATGTTGTTCCAATGAATTATCTTTTGGAATGCCGATTCACT 39494
                                                                             
  rnd-4_family-       4520 CGACTTCGAATGTTGTTCCAATGAATTATCTTTTGGAATGCCGATTCACT 4569

  contig10_pilo      39495 TTATTATGCGCTCTGCTGATGTTCCCATGTAGGGTATGCGATTCTGTGAG 39544
                                                                             
  rnd-4_family-       4570 TTATTATGCGCTCTGCTGATGTTCCCATGTAGGGTATGCGATTCTGTGAG 4619

  contig10_pilo      39545 TGTACAGCTGTATTTGCGTCTGCAGATGTGAT 39576
                                      i                    
  rnd-4_family-       4620 TGTACAGCTGTGTTTGCGTCTGCAGATGTGAT 4651

Matrix = 20p53g.matrix
Kimura (with divCpGMod) = 0.99
Transitions / transversions = 3.40 (17/5)
Gap_init rate = 0.00 (1 / 2831), avg. gap size = 1.00 (1 / 1)

 
 

 
   +      47    8.1  0.0  0.0  contig10_pilon    39577   39641 (1775238) +  (TGCGGG)n          Simple_repeat        1     65     (0)     9     
 
 ANNOTATION EVIDENCE: 
    47   8.12 0.00 0.00  contig10_pilon    39577   39641   1775238 +  (TGCGGG)n          Simple_repeat        1     65       0      
47 8.12 0.00 0.00 contig10_pilon 39577 39641 (1775238) (TGCGGG)n#Simple_repeat 1 65 (0) c_b495s251i1

  contig10_pilo      39577 TGCGGGTGCGGGTGCGGGTGAGGTTGCGGTTGCGGGTGCGGGTATGGGTG 39626
                                               v  v     v             ii     
  (TGCGGG)n#Sim          1 TGCGGGTGCGGGTGCGGGTGCGGGTGCGGGTGCGGGTGCGGGTGCGGGTG 50

  contig10_pilo      39627 CGGGTGCGGGTGCGG 39641
                                          
  (TGCGGG)n#Sim         51 CGGGTGCGGGTGCGG 65

Matrix = Unknown
Transitions / transversions = 0.67 (2/3)
Gap_init rate = 0.00 (0 / 64), avg. gap size = 0.0 (0 / 0)

 
 
 
 
  +   36459    8.5  5.6  1.8  contig10_pilon    39642   39700 (1775179) +  rnd-4_family-738   LTR/Gypsy         4652   4758  (1696)     7     
 
 ANNOTATION EVIDENCE: 
   448  10.10 6.59 2.11  contig10_pilon    39642   39700   1775179 +  rnd-4_family-738   LTR/Gypsy         4694   4758    1696      
448 10.10 6.59 2.11 contig10_pilon 39642 39700 (1775179) rnd-4_family-738#LTR/Gypsy 4694 4758 (1696) m_b495s001i5

  contig10_pilo      39642 ATGTGGGT------TCATGGATGATGAAAAACTAAATTTTAGTAGTAAAT 39685
                                   ------                                    
  rnd-4_family-       4694 ATGTGGGTCTNNNNTCATGGATGATGAAAAACTAAATTTTAGTAGTAAAT 4743

  contig10_pilo      39686 GCTTAGTAGTAAATG 39700
                                          
  rnd-4_family-       4744 GCTTAGTAGTAAATG 4758

Matrix = 20p53g.matrix
Kimura (with divCpGMod) = 9.73
Transitions / transversions = 1.00 (0/0)
Gap_init rate = 0.02 (1 / 58), avg. gap size = 6.00 (6 / 1)

 36459   1.71 1.10 0.62  contig10_pilon    39642   39652   1775227 +  rnd-4_family-738   LTR/Gypsy         4652   4661    1793      
36459 1.71 1.10 0.62 contig10_pilon 39642 39652 (1775227) rnd-4_family-738#LTR/Gypsy 4652 4661 (1793) m_b495s001i4

  contig10_pilo      39642 ATGTGGGTTCA 39652
                           -  i    v  
  rnd-4_family-       4652 -TGCGGGTGCA 4661

Matrix = 20p53g.matrix
Kimura (with divCpGMod) = 0.99
Transitions / transversions = 1.00 (1/1)
Gap_init rate = 0.10 (1 / 10), avg. gap size = 1.00 (1 / 1)

 
 

 
   +    4431    2.5  0.3  0.0  contig10_pilon    39701   40266 (1774613) C  rnd-4_family-108   Unknown            (0)   1698    1131    10     
 
 ANNOTATION EVIDENCE: 
  4431   2.47 0.35 0.00  contig10_pilon    39701   40266   1774613 C  rnd-4_family-108   Unknown           1131   1698       0      
4431 2.47 0.35 0.00 contig10_pilon 39701 40266 (1774613) C rnd-4_family-108#Unknown (0) 1698 1131 m_b495s001i6

  contig10_pilo      39701 GACCCCTAGAAAACCGTGCGCAGGGTCCGTGCGCATCCGTGCGCAAGAAT 39750
                                                                             
C rnd-4_family-       1698 GACCCCTAGAAAACCGTGCGCAGGGTCCGTGCGCATCCGTGCGCAAGAAT 1649

  contig10_pilo      39751 ATCCGTGCGCAGAACTTCCGTGCGCAGGGTTAGAACTTTCGTGCGCATGA 39800
                                                                             
C rnd-4_family-       1648 ATCCGTGCGCAGAACTTCCGTGCGCAGGGTTAGAACTTTCGTGCGCATGA 1599

  contig10_pilo      39801 GTTTAACTTTCGTGCGCAGGGTCCAAATGTCCGTGCGCACTTTTCAAAAT 39850
                                                                             
C rnd-4_family-       1598 GTTTAACTTTCGTGCGCAGGGTCCAAATGTCCGTGCGCACTTTTCAAAAT 1549

  contig10_pilo      39851 CCGTGCGCAGGGTCCTGGTCATCCGTGCGCATGAAATGTGCTTTCGTGCG 39900
                                                                             
C rnd-4_family-       1548 CCGTGCGCAGGGTCCTGGTCATCCGTGCGCATGAAATGTGCTTTCGTGCG 1499

  contig10_pilo      39901 CAATTTTCACAATCCGTGCGCAGGCTCCTGATAT-CCGTGCGCAGTTTTC 39949
                             ?        i v              ?  vi -         ?     
C rnd-4_family-       1498 CANTTTTCACAGTGCGTGCGCAGGCTCCNGAAGTGCCGTGCGCANTTTTC 1449

  contig10_pilo      39950 ACAATCCGTGCGCAAAGTACAAAATC-CGTGCGCAGTGTGAAAAACATCA 39998
                             i  v            v   i?v?-                       
C rnd-4_family-       1448 ACGATGCGTGCGCAAAGTCCAAGNGNGCGTGCGCAGTGTGAAAAACATCA 1399

  contig10_pilo      39999 AAAATCTTTTGAAGATGTATATCATAGATGTATTTATAGCACTGTGTGCA 40048
                                                                i            
C rnd-4_family-       1398 AAAATCTTTTGAAGATGTATATCATAGATGTATTTATGGCACTGTGTGCA 1349

  contig10_pilo      40049 TTGATAGAGGTGCACATCTCTAGTGCCAAATATTTTAGATAAACTACTGT 40098
                                                    i   v                    
C rnd-4_family-       1348 TTGATAGAGGTGCACATCTCTAGTGTCAACTATTTTAGATAAACTACTGT 1299

  contig10_pilo      40099 GCTACATGATTTGTTCGAAATTTCACTTCCACGTTAGTACAGGATCAGAA 40148
                                                                             
C rnd-4_family-       1298 GCTACATGATTTGTTCGAAATTTCACTTCCACGTTAGTACAGGATCAGAA 1249

  contig10_pilo      40149 ATATTCAGCCAGCATGTCGGTTTGTGTTCTCAGCTGATTTCCTGAAGAGG 40198
                                            i            v                   
C rnd-4_family-       1248 ATATTCAGCCAGCATGTTGGTTTGTGTTCTAAGCTGATTTCCTGAAGAGG 1199

  contig10_pilo      40199 CTCTAAGTAGGTACTCATAACTCAAAAGATGTTACCCTCATACAGTTATG 40248
                                                                             
C rnd-4_family-       1198 CTCTAAGTAGGTACTCATAACTCAAAAGATGTTACCCTCATACAGTTATG 1149

  contig10_pilo      40249 TTATGTTAGGTTAGATTA 40266
                                             
C rnd-4_family-       1148 TTATGTTAGGTTAGATTA 1131

Matrix = 20p53g.matrix
Kimura (with divCpGMod) = 2.37
Transitions / transversions = 1.00 (7/7)
Gap_init rate = 0.00 (2 / 565), avg. gap size = 1.00 (2 / 2)

 
 
 
 
  +      13   27.3  2.8  4.3  contig10_pilon    40429   40499 (1774380) +  (CAATTG)n          Simple_repeat        1     70     (0)    11     
 
 ANNOTATION EVIDENCE: 
    13  27.29 2.82 4.29  contig10_pilon    40429   40499   1774380 +  (CAATTG)n          Simple_repeat        1     70       0      
13 27.29 2.82 4.29 contig10_pilon 40429 40499 (1774380) (CAATTG)n#Simple_repeat 1 70 (0) m_b495s252i4

  contig10_pilo      40429 CAAGTGC-ATTGCCAATGGCGATGGGAATTGATAATTGCAA-TCCTGTTG 40476
                              v   -    -    v  i  v v     -i        - v vi   
  (CAATTG)n#Sim          1 CAATTGCAATTG-CAATTGCAATTGCAATTG-CAATTGCAATTGCAATTG 48

  contig10_pilo      40477 CAATCTCATGTTCCACTGGCAAT 40499
                               iv  -i  v  v v     
  (CAATTG)n#Sim         49 CAATTGCA-ATTGCAATTGCAAT 70

Matrix = Unknown
Transitions / transversions = 0.50 (5/10)
Gap_init rate = 0.07 (5 / 70), avg. gap size = 1.00 (5 / 5)

 
 

 
   +    7995    2.9  4.6  0.1  contig10_pilon    40671   41547 (1773332) C  rnd-4_family-108   Unknown          (561)   1137     227    10     
 
 ANNOTATION EVIDENCE: 
  7995   2.94 4.60 0.09  contig10_pilon    40671   41547   1773332 C  rnd-4_family-108   Unknown            227   1137     561      
7995 2.94 4.60 0.09 contig10_pilon 40671 41547 (1773332) C rnd-4_family-108#Unknown (561) 1137 227 m_b495s001i7

  contig10_pilo      40671 TACAGTAAGCTTGCTAAGGTTAGAACTTATGTTATTTTGGTTCATGAGTT 40720
                             v v                                          i  
C rnd-4_family-       1137 TAGATTAAGCTTGCTAAGGTTAGAACTTATGTTATTTTGGTTCATGAATT 1088

  contig10_pilo      40721 CAGGAGTAATATCCTTTGCAGCCTGAGTAACCATGATTCACTCATCTACT 40770
                                                                             
C rnd-4_family-       1087 CAGGAGTAATATCCTTTGCAGCCTGAGTAACCATGATTCACTCATCTACT 1038

  contig10_pilo      40771 AATAGATTCTAAGCCCTGATCCCAGTTTGGTCAAAAAATCTCAAATGAAT 40820
                                                                             
C rnd-4_family-       1037 AATAGATTCTAAGCCCTGATCCCAGTTTGGTCAAAAAATCTCAAATGAAT 988

  contig10_pilo      40821 TGTCTGGGTCAAGGAGAGACGAGACACGTGTTTTGGGCCGTGGAGTGGAC 40870
                                                                             
C rnd-4_family-        987 TGTCTGGGTCAAGGAGAGACGAGACACGTGTTTTGGGCCGTGGAGTGGAC 938

  contig10_pilo      40871 CTGGAACAAGCCAAAGTTTGCTGTGTCTGCTCCACCACCTCTGTACCACA 40920
                                                                             
C rnd-4_family-        937 CTGGAACAAGCCAAAGTTTGCTGTGTCTGCTCCACCACCTCTGTACCACA 888

  contig10_pilo      40921 GAGGTGCTCACTCTCCTGCATCACACCATCATCCCGTCCCTTCAAATCTT 40970
                                                 i     i               i     
C rnd-4_family-        887 GAGGTGCTCACTCTCCTGCATCGCACCACCATCCCGTCCCTTCAGATCTT 838

  contig10_pilo      40971 CTTGTTGATTAGATGGGTTCCAACCTCATCCTCACCATGCCATCTCACAG 41020
                                                                             
C rnd-4_family-        837 CTTGTTGATTAGATGGGTTCCAACCTCATCCTCACCATGCCATCTCACAG 788

  contig10_pilo      41021 AGGGACTTGCACGCCCACACCATATTATACTCAAGTTACCAGGATGTATT 41070
                                                                             
C rnd-4_family-        787 AGGGACTTGCACGCCCACACCATATTATACTCAAGTTACCAGGATGTATT 738

  contig10_pilo      41071 TTCTGTAGTTTGGACTAGTGGTTAACATGTACATGTTCTTAATGGATTCG 41120
                                                                             
C rnd-4_family-        737 TTCTGTAGTTTGGACTAGTGGTTAACATGTACATGTTCTTAATGGATTCG 688

  contig10_pilo      41121 AAACACCCTCAGTTCAAATCCAGTCATAGACATCCATTTTGTGGCACTTC 41170
                                                                          -  
C rnd-4_family-        687 AAACACCCTCAGTTCAAATCCAGTCATAGACATCCATTTTGTGGCAC-TC 639

  contig10_pilo      41171 TACCTGTGTTTACTACATTCTGTAGTGCATTAACAAACTGTACTTGAGCA 41220
                                                      i                      
C rnd-4_family-        638 TACCTGTGTTTACTACATTCTGTAGTGTATTAACAAACTGTACTTGAGCA 589

  contig10_pilo      41221 GTCAATTGCAACAAGACTTATGGAAATTGTTGGTGTTTGTTAATGAACTT 41270
                                                                             
C rnd-4_family-        588 GTCAATTGCAACAAGACTTATGGAAATTGTTGGTGTTTGTTAATGAACTT 539

  contig10_pilo      41271 CAATGAGTTCAATCAGGTCGCCTAGATGTGCCAATGTGAAAAAAATAATG 41320
                                          i                  v               
C rnd-4_family-        538 CAATGAGTTCAATCAAGTCGCCTAGATGTGCCAAAGTGAAAAAAATAATG 489

  contig10_pilo      41321 CAGTTGTGCTTTATACAGAATCCGTGCGCAACACACAAAATCCGTGCGC- 41369
                                                                            -
C rnd-4_family-        488 CAGTTGTGCTTTATACAGAATCCGTGCGCAACACACAAAATCCGTGCGCA 439

  contig10_pilo      41370 -AGTGTCAGCATGTCCGTGCGC-ATGTCTACCA-TGTCCGTGCGCATGAT 41416
                           -    iv      ??       - i ?    v -                
C rnd-4_family-        438 AAGTGCAAGCATGNNCGTGCGCAACGNCTACGACTGTCCGTGCGCATGAT 389

  contig10_pilo      41417 TCTAACA-TCCGTGCGCAGGGTCCAAAATGTCGTGCGCACAAAATCACAA 41465
                             i    -                   ?           ? i        
C rnd-4_family-        388 TCCAACAGTCCGTGCGCAGGGTCCAAANTGTCGTGCGCANAGAATCACAA 339

  contig10_pilo      41466 TCCGTGCGCAGTCCTCCTTCATCCGTGCGCACAGATTTACAATCCGTGTG 41515
                                                                           i 
C rnd-4_family-        338 TCCGTGCGCAGTCCTCCTTCATCCGTGCGCACAGATTTACAATCCGTGCG 289

  contig10_pilo      41516 CATGATTTCAACTTTCGTGCGCAGTGTGAAAA------------------ 41547
                               ?         ?                 ------------------
C rnd-4_family-        288 CATGNTTTCAACTTNCGTGCGCAGTGTGAAAAATCCGTGCGCAGGCGCAC 239

  contig10_pilo      41547 ------------ 41547
                           ------------
C rnd-4_family-        238 GTTAGTTTTGCG 227

Matrix = 20p53g.matrix
Kimura (with divCpGMod) = 2.19
Transitions / transversions = 2.20 (11/5)
Gap_init rate = 0.01 (7 / 876), avg. gap size = 5.14 (36 / 7)

 
 
 
 
  +      29    2.6  2.4  2.4  contig10_pilon    41548   41588 (1773291) +  (ATCCGTGCGC)n      Simple_repeat        1     41     (0)    12     
 
 ANNOTATION EVIDENCE: 
    29   2.61 2.44 2.44  contig10_pilon    41548   41588   1773291 +  (ATCCGTGCGC)n      Simple_repeat        1     41       0      
29 2.61 2.44 2.44 contig10_pilon 41548 41588 (1773291) (ATCCGTGCGC)n#Simple_repeat 1 41 (0) c_b495s251i2

  contig10_pilo      41548 ATCCGTGCGCAGTCCGTGCGCATCCGTGCGCA-ACGTGCGCA 41588
                                      -                    -v        
  (ATCCGTGCGC)n          1 ATCCGTGCGCA-TCCGTGCGCATCCGTGCGCATCCGTGCGCA 41

Matrix = Unknown
Transitions / transversions = 0.00 (0/1)
Gap_init rate = 0.05 (2 / 40), avg. gap size = 1.00 (2 / 2)

 
 

 
   +    7995    2.9  4.6  0.1  contig10_pilon    41589   41798 (1773081) C  rnd-4_family-108   Unknown         (1472)    226       2    10     
 
 ANNOTATION EVIDENCE: 
  7995   2.94 4.60 0.09  contig10_pilon    41589   41798   1773081 C  rnd-4_family-108   Unknown              2    226    1472      
7995 2.94 4.60 0.09 contig10_pilon 41589 41798 (1773081) C rnd-4_family-108#Unknown (1472) 226 2 m_b495s001i7

  contig10_pilo      41589 ACATGCGC--------GCGGAACTTGAAACCAGTAATTTGACAATATCTT 41630
                             i     --------              v?   v? i          i
C rnd-4_family-        226 ACGTGCGCACGTGCGCGCGGAACTTGAAACGNGTATNTCGACAATATCTC 177

  contig10_pilo      41631 GGCCAGTTTTTAAGGTTTGATAG--GTTTGTGCACACAGACATGTGCGGG 41678
                              vi                i --   ?       ??      i ?   
C rnd-4_family-        176 GGCGGGTTTTTAAGGTTTGATGGCCGTTNGTGCACANNGACATGCGNGGG 127

  contig10_pilo      41679 TAGGTGTA-AGCTACTGGAATGACCCTCAACAATCAT----TCTAGGATG 41723
                                 i -      i     ?   i     i    i----         
C rnd-4_family-        126 TAGGTGCATAGCTACCGGAATNACCTTCAACGATCACAATATCTAGGATG 77

  contig10_pilo      41724 TCTGAACAGTCTTCTAGGGCAGGATTACTGCTGCGCACGGACCTCGCGCA 41773
                                  i ?      ?   ?         i                   
C rnd-4_family-         76 TCTGAACGGNCTTCTANGGCNGGATTACTGTTGCGCACGGACCTCGCGCA 27

  contig10_pilo      41774 CGTGCGCACGGTTTTCTCGTGACCC 41798
                                                    
C rnd-4_family-         26 CGTGCGCACGGTTTTCTCGTGACCC 2

Matrix = 20p53g.matrix
Kimura (with divCpGMod) = 2.19
Transitions / transversions = 4.33 (13/3)
Gap_init rate = 0.02 (4 / 209), avg. gap size = 3.75 (15 / 4)

 
 
 
 
  +   12988    3.1  0.2  1.1  contig10_pilon    41799   43512 (1771367) +  rnd-4_family-738   LTR/Gypsy         4749   6447     (7)     7     
 
 ANNOTATION EVIDENCE: 
 12988   3.12 0.23 1.12  contig10_pilon    41799   43512   1771367 +  rnd-4_family-738   LTR/Gypsy         4749   6447       7      
12988 3.12 0.23 1.12 contig10_pilon 41799 43512 (1771367) rnd-4_family-738#LTR/Gypsy 4749 6447 (7) m_b495s001i8

  contig10_pilo      41799 GTAGTAAATGCTTGCAGGTGCGGTTGCAGGCGCGGGCGCGGTTGCAGGTG 41848
                                                                           i 
  rnd-4_family-       4749 GTAGTAAATGCTTGCAGGTGCGGTTGCAGGCGCGGGCGCGGTTGCAGGCG 4798

  contig10_pilo      41849 CGGGCGTGGGTGCGGGTGCGGGTGCGGGTGCGGGTCCATGGATGATGAAA 41898
                                 ------------                                
  rnd-4_family-       4799 CGGGCG------------CGGGTGCGGGTGCGGGTCCATGGATGATGAAA 4836

  contig10_pilo      41899 AATAAAATCATAGTAGTGAATGCTTGCAGGTGCGGTTGCGGGCGCGGTTG 41948
                                               i            v  ???v  i       
  rnd-4_family-       4837 AATAAAATCATAGTAGTGAACGCTTGCAGGTGCCGTNNNTGGTGCGGTTG 4886

  contig10_pilo      41949 CAGGCGCGGGCGCGGGCGTGGGTGCAGGCGCGGGCGCGGGTGCGGGTGTG 41998
                                      i------   v        i   i             i 
  rnd-4_family-       4887 CAGGCGCGGGCA------TGGCTGCAGGCGTGGGTGCGGGTGCGGGTGCG 4930

  contig10_pilo      41999 GGTGCTTGTAACCATCAGCCACCGCGGTTCTACTTGGACAGTATACAGGC 42048
                                                                   v         
  rnd-4_family-       4931 GGTGCTTGTAACCATCAGCCACCGCGGTTCTACTTGGACATTATACAGGC 4980

  contig10_pilo      42049 TGATGGTATTGCGGATGCGATGTATAGCATGTGAATCATTGGACGGCTTG 42098
                                                                             
  rnd-4_family-       4981 TGATGGTATTGCGGATGCGATGTATAGCATGTGAATCATTGGACGGCTTG 5030

  contig10_pilo      42099 CACTGCGGCAAAGTCCGATGTCCGAGGTTCGGAAGTATTCTTCCACCAAC 42148
                                                                             
  rnd-4_family-       5031 CACTGCGGCAAAGTCCGATGTCCGAGGTTCGGAAGTATTCTTCCACCAAC 5080

  contig10_pilo      42149 AGCACAGAGTGCAGCAGAATATTCCACAAAGCTCCGGAAACGCTGAAAAC 42198
                                                                             
  rnd-4_family-       5081 AGCACAGAGTGCAGCAGAATATTCCACAAAGCTCCGGAAACGCTGAAAAC 5130

  contig10_pilo      42199 TTGGCACATGTCTCCAATGGTCGTTCCTCATTGATCTGTGGTGTATCTGA 42248
                                          i      i                           
  rnd-4_family-       5131 TTGGCACATGTCTCCGATGGTCATTCCTCATTGATCTGTGGTGTATCTGA 5180

  contig10_pilo      42249 AGCTTACAATTGGATGCCTGTGTGCGGTAACCGGGCGGATTGCAAGTCGC 42298
                                                                             
  rnd-4_family-       5181 AGCTTACAATTGGATGCCTGTGTGCGGTAACCGGGCGGATTGCAAGTCGC 5230

  contig10_pilo      42299 AGCTGCGGGTGCGGGTGCAGGCGCGGGCGCGGGTGCGGTTGCGGCTGCGG 42348
                                                                             
  rnd-4_family-       5231 AGCTGCGGGTGCGGGTGCAGGCGCGGGCGCGGGTGCGGTTGCGGCTGCGG 5280

  contig10_pilo      42349 CTGCTGCTACATCCAGCACATGGAGCAAGTTAGAGCATGCTACAAGGGTC 42398
                                                                             
  rnd-4_family-       5281 CTGCTGCTACATCCAGCACATGGAGCAAGTTAGAGCATGCTACAAGGGTC 5330

  contig10_pilo      42399 CGGGCTTTTTGAAACTTGGCCAACTGGAAGATTCTGGGTCACTCTGTTAA 42448
                                                                             
  rnd-4_family-       5331 CGGGCTTTTTGAAACTTGGCCAACTGGAAGATTCTGGGTCACTCTGTTAA 5380

  contig10_pilo      42449 TGCACACTTTGCTCTAAGCTTAGTAATTCGTCACAAAGGTCTCAAGTCTG 42498
                                                                             
  rnd-4_family-       5381 TGCACACTTTGCTCTAAGCTTAGTAATTCGTCACAAAGGTCTCAAGTCTG 5430

  contig10_pilo      42499 TTCTCTTTTGAGATATCCCTGCAGGTGCGACCGCATGTGCTGCAGGCGCA 42548
                                                                             
  rnd-4_family-       5431 TTCTCTTTTGAGATATCCCTGCAGGTGCGACCGCATGTGCTGCAGGCGCA 5480

  contig10_pilo      42549 AGCGCAGGTGCGGTTGCAGGTGCGGCTGCAGGTGCGGTTGCTGCAGAAGT 42598
                                                                             
  rnd-4_family-       5481 AGCGCAGGTGCGGTTGCAGGTGCGGCTGCAGGTGCGGTTGCTGCAGAAGT 5530

  contig10_pilo      42599 GAATGCAGCTGCATTTAGCTTTAATCCATTCAGAATTCAATTCCTGTGTA 42648
                                                                             
  rnd-4_family-       5531 GAATGCAGCTGCATTTAGCTTTAATCCATTCAGAATTCAATTCCTGTGTA 5580

  contig10_pilo      42649 TTATAGTTAGCAAGACTTTTATATGTATAATAACATGAAGGACATCAATT 42698
                                                                             
  rnd-4_family-       5581 TTATAGTTAGCAAGACTTTTATATGTATAATAACATGAAGGACATCAATT 5630

  contig10_pilo      42699 GGAACATTTTGTGTTTTGGGGTAAAAAAGTTTTTTTTTCATAAA-TAAGC 42747
                                                                       -     
  rnd-4_family-       5631 GGAACATTTTGTGTTTTGGGGTAAAAAAGTTTTTTTTTCATAAATTAAGC 5680

  contig10_pilo      42748 GCCGGTGACGACCGGTGGCGGCAGTGGCGACGGTTCCGGCGATTTCCGAC 42797
                                                 i                           
  rnd-4_family-       5681 GCCGGTGACGACCGGTGGCGGCGGTGGCGACGGTTCCGGCGATTTCCGAC 5730

  contig10_pilo      42798 CGCAGCGGCGGCGGCGGCGACGTCGGTCGGCGATTTCCGGCCGTTTCCGG 42847
                                                                             
  rnd-4_family-       5731 CGCAGCGGCGGCGGCGGCGACGTCGGTCGGCGATTTCCGGCCGTTTCCGG 5780

  contig10_pilo      42848 CGGTGGCGGAGGCGGCGGCACCATTCCGGGCAGTACCGGAATGTGCCGGA 42897
                                                 i                           
  rnd-4_family-       5781 CGGTGGCGGAGGCGGCGGCACCGTTCCGGGCAGTACCGGAATGTGCCGGA 5830

  contig10_pilo      42898 AGGCAGCGCACCCGCAGCGTGGACCGCAATGCCACCCGCAGCGTCACCAG 42947
                           v                                          i      
  rnd-4_family-       5831 CGGCAGCGCACCCGCAGCGTGGACCGCAATGCCACCCGCAGCGCCACCAG 5880

  contig10_pilo      42948 CGCACCCGCAATGAAATGCGCACCAGTACCTGCGGGGGTACCTGTGCACC 42997
                                      i    i                           i     
  rnd-4_family-       5881 CGCACCCGCAACGAAACGCGCACCAGTACCTGCGGGGGTACCTGCGCACC 5930

  contig10_pilo      42998 TGTAGAGCGGACCGCAATGCAATCCGCAGCGCCACCAGCGCACCCGCAAT 43047
                                                                             
  rnd-4_family-       5931 TGTAGAGCGGACCGCAATGCAATCCGCAGCGCCACCAGCGCACCCGCAAT 5980

  contig10_pilo      43048 GCGACCCGCAGCAGCACCTGTGCACGCGCACCCGCAGCGCGGACCGCAAT 43097
                                               i                             
  rnd-4_family-       5981 GCGACCCGCAGCAGCACCTGCGCACGCGCACCCGCAGCGCGGACCGCAAT 6030

  contig10_pilo      43098 GGGAACCGCACCAGCACCGTTGCACCCGCAGTGCGGACCGCATTGCAAGC 43147
                                              ?i  i       i                i 
  rnd-4_family-       6031 GGGAACCGCACCAGCACCGNCGCGCCCGCAGCGCGGACCGCATTGCAAAC 6080

  contig10_pilo      43148 CGCGGCAGCACCCGCACACCCGCAGGCCAACCCGTGGCAGCATCCGTGGG 43197
                                                             i           i   
  rnd-4_family-       6081 CGCGGCAGCACCCGCACACCCGCAGGCCAACCCGCGGCAGCATCCGCGGG 6130

  contig10_pilo      43198 CCTGCAATGCCAACCGCAGCACCACCAGCGCACCCGCAATGCAGCCCGCA 43247
                             i                  v         i       i  i       
  rnd-4_family-       6131 CCCGCAATGCCAACCGCAGCAGCACCAGCGCGCCCGCAACGCGGCCCGCA 6180

  contig10_pilo      43248 CCAGCACCTGCACACCCGCAGCGTGGACCGCAGCAGCATTCGCACACCCG 43297
                                   i  i           i              ii   i      
  rnd-4_family-       6181 CCAGCACCCGCGCACCCGCAGCGCGGACCGCAGCAGCACCCGCGCACCCG 6230

  contig10_pilo      43298 CAGAGCAACCCGCAGCAGCATTCCAGGACCCGCAGAGCGAACCGCACTAG 43347
                                  i                  v        ?           i  
  rnd-4_family-       6231 CAGAGCAGCCCGCAGCAGCATTCCAGCACCCGCAGNGCGAACCGCACCAG 6280

  contig10_pilo      43348 CAT--ACAGGCAGCCGCAAAGAAACCCGCACCACCACCTGCAGTCCCACC 43395
                              --i      v     i  vi               i    ??     
  rnd-4_family-       6281 CATTCGCAGGCACCCGCAGAGCGACCCGCACCACCACCCGCAGNNCCACC 6330

  contig10_pilo      43396 GACCCACCCGCAACAGGCGCGGGCGCGGGCGCGGGCCTGACGCT-ACCCT 43444
                                                                       -     
  rnd-4_family-       6331 GACCCACCCGCAACAGGCGCGGGCGCGGGCGCGGGCCTGACGCTAACCCT 6380

  contig10_pilo      43445 ATTTTCCGGGGGGGTAAAATAGGGTTAGCGTCACCCGGTTGCAGCTACCT 43494
                                                                  i  i?  i   
  rnd-4_family-       6381 ATTTTCCGGGGGGGTAAAATAGGGTTAGCGTCACCCGGTCGCGNCTGCCT 6430

  contig10_pilo      43495 GAGGTGCACCAGGCGGGA 43512
                                 vii -       
  rnd-4_family-       6431 GAGGTGGGTC-GGCGGGA 6447

Matrix = 20p53g.matrix
Kimura (with divCpGMod) = 1.67
Transitions / transversions = 4.30 (43/10)
Gap_init rate = 0.01 (22 / 1713), avg. gap size = 1.05 (23 / 22)

 
 

 
 
   +    4431    2.5  0.3  0.0  contig10_pilon    47100   47665 (1767214) C  rnd-4_family-108   Unknown            (0)   1698    1131    13     
 
 ANNOTATION EVIDENCE: 
  4431   2.47 0.35 0.00  contig10_pilon    47100   47665   1767214 C  rnd-4_family-108   Unknown           1131   1698       0      
4431 2.47 0.35 0.00 contig10_pilon 47100 47665 (1767214) C rnd-4_family-108#Unknown (0) 1698 1131 m_b495s001i9

  contig10_pilo      47100 GACCCCTAGAAAACCGTGCGCAGGGTCCGTGCGCATCCGTGCGCAAGAAT 47149
                                                                             
C rnd-4_family-       1698 GACCCCTAGAAAACCGTGCGCAGGGTCCGTGCGCATCCGTGCGCAAGAAT 1649

  contig10_pilo      47150 ATCCGTGCGCAGAACTTCCGTGCGCAGGGTTAGAACTTTCGTGCGCATGA 47199
                                                                             
C rnd-4_family-       1648 ATCCGTGCGCAGAACTTCCGTGCGCAGGGTTAGAACTTTCGTGCGCATGA 1599

  contig10_pilo      47200 GTTTAACTTTCGTGCGCAGGGTCCAAATGTCCGTGCGCACTTTTCAAAAT 47249
                                                                             
C rnd-4_family-       1598 GTTTAACTTTCGTGCGCAGGGTCCAAATGTCCGTGCGCACTTTTCAAAAT 1549

  contig10_pilo      47250 CCGTGCGCAGGGTCCTGGTCATCCGTGCGCATGAAATGTGCTTTCGTGCG 47299
                                                                             
C rnd-4_family-       1548 CCGTGCGCAGGGTCCTGGTCATCCGTGCGCATGAAATGTGCTTTCGTGCG 1499

  contig10_pilo      47300 CAATTTTCACAATCCGTGCGCAGGCTCCTGATAT-CCGTGCGCAGTTTTC 47348
                             ?        i v              ?  vi -         ?     
C rnd-4_family-       1498 CANTTTTCACAGTGCGTGCGCAGGCTCCNGAAGTGCCGTGCGCANTTTTC 1449

  contig10_pilo      47349 ACAATCCGTGCGCAAAGTACAAAATC-CGTGCGCAGTGTGAAAAACATCA 47397
                             i  v            v   i?v?-                       
C rnd-4_family-       1448 ACGATGCGTGCGCAAAGTCCAAGNGNGCGTGCGCAGTGTGAAAAACATCA 1399

  contig10_pilo      47398 AAAATCTTTTGAAGATGTATATCATAGATGTATTTATAGCACTGTGTGCA 47447
                                                                i            
C rnd-4_family-       1398 AAAATCTTTTGAAGATGTATATCATAGATGTATTTATGGCACTGTGTGCA 1349

  contig10_pilo      47448 TTGATAGAGGTGCACATCTCTAGTGCCAAATATTTTAGATAAACTACTGT 47497
                                                    i   v                    
C rnd-4_family-       1348 TTGATAGAGGTGCACATCTCTAGTGTCAACTATTTTAGATAAACTACTGT 1299

  contig10_pilo      47498 GCTACATGATTTGTTCGAAATTTCACTTCCACGTTAGTACAGGATCAGAA 47547
                                                                             
C rnd-4_family-       1298 GCTACATGATTTGTTCGAAATTTCACTTCCACGTTAGTACAGGATCAGAA 1249

  contig10_pilo      47548 ATATTCAGCCAGCATGTCGGTTTGTGTTCTCAGCTGATTTCCTGAAGAGG 47597
                                            i            v                   
C rnd-4_family-       1248 ATATTCAGCCAGCATGTTGGTTTGTGTTCTAAGCTGATTTCCTGAAGAGG 1199

  contig10_pilo      47598 CTCTAAGTAGGTACTCATAACTCAAAAGATGTTACCCTCATACAGTTATG 47647
                                                                             
C rnd-4_family-       1198 CTCTAAGTAGGTACTCATAACTCAAAAGATGTTACCCTCATACAGTTATG 1149

  contig10_pilo      47648 TTATGTTAGGTTAGATTA 47665
                                             
C rnd-4_family-       1148 TTATGTTAGGTTAGATTA 1131

Matrix = 20p53g.matrix
Kimura (with divCpGMod) = 2.37
Transitions / transversions = 1.00 (7/7)
Gap_init rate = 0.00 (2 / 565), avg. gap size = 1.00 (2 / 2)

 
 
 
 
  +      13   27.3  2.8  4.3  contig10_pilon    47828   47898 (1766981) +  (CAATTG)n          Simple_repeat        1     70     (0)    14     
 
 ANNOTATION EVIDENCE: 
    13  27.29 2.82 4.29  contig10_pilon    47828   47898   1766981 +  (CAATTG)n          Simple_repeat        1     70       0      
13 27.29 2.82 4.29 contig10_pilon 47828 47898 (1766981) (CAATTG)n#Simple_repeat 1 70 (0) m_b495s252i5

  contig10_pilo      47828 CAAGTGC-ATTGCCAATGGCGATGGGAATTGATAATTGCAA-TCCTGTTG 47875
                              v   -    -    v  i  v v     -i        - v vi   
  (CAATTG)n#Sim          1 CAATTGCAATTG-CAATTGCAATTGCAATTG-CAATTGCAATTGCAATTG 48

  contig10_pilo      47876 CAATCTCATGTTCCACTGGCAAT 47898
                               iv  -i  v  v v     
  (CAATTG)n#Sim         49 CAATTGCA-ATTGCAATTGCAAT 70

Matrix = Unknown
Transitions / transversions = 0.50 (5/10)
Gap_init rate = 0.07 (5 / 70), avg. gap size = 1.00 (5 / 5)

 
 

 
   +    8358    3.4  1.3  0.5  contig10_pilon    48070   49197 (1765682) C  rnd-4_family-108   Unknown          (561)   1137       1    13     
 
 ANNOTATION EVIDENCE: 
  8358   3.39 1.33 0.53  contig10_pilon    48070   49197   1765682 C  rnd-4_family-108   Unknown              1   1137     561      
8358 3.39 1.33 0.53 contig10_pilon 48070 49197 (1765682) C rnd-4_family-108#Unknown (561) 1137 1 m_b495s001i10

  contig10_pilo      48070 TACAGTAAGCTTGCTAAGGTTAGAACTTATGTTATTTTGGTTCATGAGTT 48119
                             v v                                          i  
C rnd-4_family-       1137 TAGATTAAGCTTGCTAAGGTTAGAACTTATGTTATTTTGGTTCATGAATT 1088

  contig10_pilo      48120 CAGGAGTAATATCCTTTGCAGCCTGAGTAACCATGATTCACTCATCTACT 48169
                                                                             
C rnd-4_family-       1087 CAGGAGTAATATCCTTTGCAGCCTGAGTAACCATGATTCACTCATCTACT 1038

  contig10_pilo      48170 AATAGATTCTAAGCCCTGATCCCAGTTTGGTCAAAAAATCTCAAATGAAT 48219
                                                                             
C rnd-4_family-       1037 AATAGATTCTAAGCCCTGATCCCAGTTTGGTCAAAAAATCTCAAATGAAT 988

  contig10_pilo      48220 TGTCTGGGTCAAGGAGAGACGAGACACGTGTTTTGGGCCGTGGAGTGGAC 48269
                                                                             
C rnd-4_family-        987 TGTCTGGGTCAAGGAGAGACGAGACACGTGTTTTGGGCCGTGGAGTGGAC 938

  contig10_pilo      48270 CTGGAACAAGCCAAAGTTTGCTGTGTCTGCTCCACCACCTCTGTACCACA 48319
                                                                             
C rnd-4_family-        937 CTGGAACAAGCCAAAGTTTGCTGTGTCTGCTCCACCACCTCTGTACCACA 888

  contig10_pilo      48320 GAGGTGCTCACTCTCCTGCATCACACCATCATCCCGTCCCTTCAAATCTT 48369
                                                 i     i               i     
C rnd-4_family-        887 GAGGTGCTCACTCTCCTGCATCGCACCACCATCCCGTCCCTTCAGATCTT 838

  contig10_pilo      48370 CTTGTTGATTAGATGGGTTCCAACCTCATCCTCACCATGCCATCTCACAG 48419
                                                                             
C rnd-4_family-        837 CTTGTTGATTAGATGGGTTCCAACCTCATCCTCACCATGCCATCTCACAG 788

  contig10_pilo      48420 AGGGACTTGCACGCCCACACCATATTATACTCAAGTTACCAGGATGTATT 48469
                                                                             
C rnd-4_family-        787 AGGGACTTGCACGCCCACACCATATTATACTCAAGTTACCAGGATGTATT 738

  contig10_pilo      48470 TTCTGTAGTTTGGACTAGTGGTTAACATGTACATGTTCTTAATGGATTCG 48519
                                                                             
C rnd-4_family-        737 TTCTGTAGTTTGGACTAGTGGTTAACATGTACATGTTCTTAATGGATTCG 688

  contig10_pilo      48520 AAACACCCTCAGTTCAAATCCAGTCATAGACATCCATTTTGTGGCACTTC 48569
                                                                          -  
C rnd-4_family-        687 AAACACCCTCAGTTCAAATCCAGTCATAGACATCCATTTTGTGGCAC-TC 639

  contig10_pilo      48570 TACCTGTGTTTACTACATTCTGTAGTGCATTAACAAACTGTACTTGAGCA 48619
                                                      i                      
C rnd-4_family-        638 TACCTGTGTTTACTACATTCTGTAGTGTATTAACAAACTGTACTTGAGCA 589

  contig10_pilo      48620 GTCAATTGCAACAAGACTTATGGAAATTGTTGGTGTTTGTTAATGAACTT 48669
                                                                             
C rnd-4_family-        588 GTCAATTGCAACAAGACTTATGGAAATTGTTGGTGTTTGTTAATGAACTT 539

  contig10_pilo      48670 CAATGAGTTCAATCAGGTCGCCTAGATGTGCCAATGTGAAAAAAATAATG 48719
                                          i                  v               
C rnd-4_family-        538 CAATGAGTTCAATCAAGTCGCCTAGATGTGCCAAAGTGAAAAAAATAATG 489

  contig10_pilo      48720 CAGTTGTGCTTTATACAGAATCCGTGCGCAACACACAAAATCCGTGCGC- 48768
                                                                            -
C rnd-4_family-        488 CAGTTGTGCTTTATACAGAATCCGTGCGCAACACACAAAATCCGTGCGCA 439

  contig10_pilo      48769 -AGTGTCAGCATGTCCGTGCGC-ATGTCTACCA-TGTCCGTGCGCATGAT 48815
                           -    iv      ??       - i ?    v -                
C rnd-4_family-        438 AAGTGCAAGCATGNNCGTGCGCAACGNCTACGACTGTCCGTGCGCATGAT 389

  contig10_pilo      48816 TCTAACA-TCCGTGCGCAGGGTCCAAAATGTCGTGCGCACAAAATCACAA 48864
                             i    -                   ?           ? i        
C rnd-4_family-        388 TCCAACAGTCCGTGCGCAGGGTCCAAANTGTCGTGCGCANAGAATCACAA 339

  contig10_pilo      48865 TCCGTGCGCAGTCCTCCTTCATCCGTGCGCACAGATTTACAATCCGTGTG 48914
                                                                           i 
C rnd-4_family-        338 TCCGTGCGCAGTCCTCCTTCATCCGTGCGCACAGATTTACAATCCGTGCG 289

  contig10_pilo      48915 CATGATTTCAACTTTCGTGCGCAGTGTGAAAAATCCGTGCGCAGTCCGTG 48964
                               ?         ?                             --- i 
C rnd-4_family-        288 CATGNTTTCAACTTNCGTGCGCAGTGTGAAAAATCCGTGCGCAG---GCG 242

  contig10_pilo      48965 CGCATCCG---TGCGCAACATGCGCAACATGCGCGCGGAACTTGAAACCA 49011
                            i i iv ---     -  i     -  i                   v?
C rnd-4_family-        241 CACGTTAGTTTTGCGC-ACGTGCGC-ACGTGCGCGCGGAACTTGAAACGN 194

  contig10_pilo      49012 GTAATTTGACAATATCTTGGCCAGTTTTTAAGGTTTGATAG--GTTTGTG 49059
                              v? i          i   vi                i --   ?   
C rnd-4_family-        193 GTATNTCGACAATATCTCGGCGGGTTTTTAAGGTTTGATGGCCGTTNGTG 144

  contig10_pilo      49060 CACACAGACATGTGCGGGTAGGTGTA-AGCTACTGGAATGACCCTCAACA 49108
                               ??      i ?         i -      i     ?   i     i
C rnd-4_family-        143 CACANNGACATGCGNGGGTAGGTGCATAGCTACCGGAATNACCTTCAACG 94

  contig10_pilo      49109 ATCAT----TCTAGGATGTCTGAACAGTCTTCTAGGGCAGGATTACTGCT 49154
                               i----                i ?      ?   ?         i 
C rnd-4_family-         93 ATCACAATATCTAGGATGTCTGAACGGNCTTCTANGGCNGGATTACTGTT 44

  contig10_pilo      49155 GCGCACGGACCTCGCGCACGTGCGCACGGTTTTCTCGTGACCC 49197
                                                                      
C rnd-4_family-         43 GCGCACGGACCTCGCGCACGTGCGCACGGTTTTCTCGTGACCC 1

Matrix = 20p53g.matrix
Kimura (with divCpGMod) = 2.42
Transitions / transversions = 3.22 (29/9)
Gap_init rate = 0.01 (15 / 1127), avg. gap size = 1.40 (21 / 15)

 
 
 
 
 
  +   18736    1.2  0.0  0.0  contig10_pilon    49198   51500 (1763379) C  rnd-4_family-400   LTR/Gypsy      (11071)   2303       1    15     
 
 ANNOTATION EVIDENCE: 
 18736   1.22 0.04 0.04  contig10_pilon    49198   51500   1763379 C  rnd-4_family-400   LTR/Gypsy            1   2303   11071      
18736 1.22 0.04 0.04 contig10_pilon 49198 51500 (1763379) C rnd-4_family-400#LTR/Gypsy (11071) 2303 1 m_b495s001i11

  contig10_pilo      49198 GACATAATCAACCACCACCTTCTTCCATCGGTCAGCTCCAATCGCGGCTA 49247
                                          v                 i                
C rnd-4_family-       2303 GACATAATCAACCACAACCTTCTTCCATCGGTCGGCTCCAATCGCGGCTA 2254

  contig10_pilo      49248 GTCGCGCCCGATTTTCGAACGTCTCGAGGAAGGTCCCGATTTTGTATGGG 49297
                                                                             
C rnd-4_family-       2253 GTCGCGCCCGATTTTCGAACGTCTCGAGGAAGGTCCCGATTTTGTATGGG 2204

  contig10_pilo      49298 TCACCGTCAAATCGCGGAGCGCCCTTCTCGTCTGGGTACGGGAGGGGAAC 49347
                                            v                                
C rnd-4_family-       2203 TCACCGTCAAATCGCGGCGCGCCCTTCTCGTCTGGGTACGGGAGGGGAAC 2154

  contig10_pilo      49348 GCGTGCTTGTTGAGCAGCTGGAGCTTGCTGTCCTGGGGGCACAGCGGGAA 49397
                                          i              v                   
C rnd-4_family-       2153 GCGTGCTTGTTGAGCGGCTGGAGCTTGCTGGCCTGGGGGCACAGCGGGAA 2104

  contig10_pilo      49398 GGTTCTGTACTGCACGCTGCATGAACGTGAACAGTAGGTTGCCGAAGGCC 49447
                                                                             
C rnd-4_family-       2103 GGTTCTGTACTGCACGCTGCATGAACGTGAACAGTAGGTTGCCGAAGGCC 2054

  contig10_pilo      49448 GCTTCCATTTCTGGATTGCCTGGCGGGTTGGGGGG-TTTCCGTCATTGTC 49496
                                                              -          i   
C rnd-4_family-       2053 GCTTCCATTTCTGGATTGCCTGGCGGGTTGGGGGGGTTTCCGTCATCGTC 2004

  contig10_pilo      49497 CATCTCTTGGAAGTACGGATGTTCTTCTGCGAGAGGTATGCGATTGACAG 49546
                                                                             
C rnd-4_family-       2003 CATCTCTTGGAAGTACGGATGTTCTTCTGCGAGAGGTATGCGATTGACAG 1954

  contig10_pilo      49547 GTGATGGAGTGCGTGGTGCAAGTGAAGCAAATCGAGAACGATGGGGTGCG 49596
                                                                    i        
C rnd-4_family-       1953 GTGATGGAGTGCGTGGTGCAAGTGAAGCAAATCGAGAACGACGGGGTGCG 1904

  contig10_pilo      49597 ACGTCTTGATCTGAATCTTCTCGCGAACATGAAGAATGAAACTCGGGTTC 49646
                                                       i   i                 
C rnd-4_family-       1903 ACGTCTTGATCTGAATCTTCTCGCGAACGTGAGGAATGAAACTCGGGTTC 1854

  contig10_pilo      49647 CGACTCAGGGTCTCGTTCCGAGAGGGTGAGAGAAGAGAGGGAAGAAAGGG 49696
                                                                             
C rnd-4_family-       1853 CGACTCAGGGTCTCGTTCCGAGAGGGTGAGAGAAGAGAGGGAAGAAAGGG 1804

  contig10_pilo      49697 GGGGAAATGGTAAGGAAACCGATGTAGATGTATGGGGGTTCGCTTGCGTG 49746
                              i                                              
C rnd-4_family-       1803 GGGAAAATGGTAAGGAAACCGATGTAGATGTATGGGGGTTCGCTTGCGTG 1754

  contig10_pilo      49747 AGTGATGATGTTGAGGCAACAGGAATGGTTGCTTGGCGAACACGAGAACG 49796
                                                                             
C rnd-4_family-       1753 AGTGATGATGTTGAGGCAACAGGAATGGTTGCTTGGCGAACACGAGAACG 1704

  contig10_pilo      49797 AGTTCGTGCGGCAGGACCAGGTGAAGCCGTCGCAATCTCGACGCAAGTAA 49846
                                                                             
C rnd-4_family-       1703 AGTTCGTGCGGCAGGACCAGGTGAAGCCGTCGCAATCTCGACGCAAGTAA 1654

  contig10_pilo      49847 GTGGTGGGGGTGTGTGAAAGGGATAAACAGGGAGCCTGAACCGTTTGTAA 49896
                                                                             
C rnd-4_family-       1653 GTGGTGGGGGTGTGTGAAAGGGATAAACAGGGAGCCTGAACCGTTTGTAA 1604

  contig10_pilo      49897 GACTAGTTCAAAGACTCCCTGCGGTGGTCACCACTTTGAACAGCCAGGGA 49946
                                                                             
C rnd-4_family-       1603 GACTAGTTCAAAGACTCCCTGCGGTGGTCACCACTTTGAACAGCCAGGGA 1554

  contig10_pilo      49947 AGACTAAGACAATGAATACAGGAGTAGGGAGGAGCCGCAGATCAAAAGGG 49996
                                            v                                
C rnd-4_family-       1553 AGACTAAGACAATGAATCCAGGAGTAGGGAGGAGCCGCAGATCAAAAGGG 1504

  contig10_pilo      49997 TGGGTGGGTGGAGTATACCCAACACACGAGAGAATAGATATAGAGGTAGA 50046
                                                                            i
C rnd-4_family-       1503 TGGGTGGGTGGAGTATACCCAACACACGAGAGAATAGATATAGAGGTAGG 1454

  contig10_pilo      50047 GGTGAAATGGGGATATGGTCTCGAGTGTGTATGATCAAAACCCGAGTGAG 50096
                                  i                                          
C rnd-4_family-       1453 GGTGAAACGGGGATATGGTCTCGAGTGTGTATGATCAAAACCCGAGTGAG 1404

  contig10_pilo      50097 GATCACGAAGGCGAGGGGGAACCCGAATTCGTATCCGGAGAGCTAGAGAC 50146
                                     i                                 v     
C rnd-4_family-       1403 GATCACGAAGACGAGGGGGAACCCGAATTCGTATCCGGAGAGCTCGAGAC 1354

  contig10_pilo      50147 TACTCGTGTGGAGTCGTCTACGAGAAGAGGGTACACGGGAGAGGAGGATT 50196
                                                           i                 
C rnd-4_family-       1353 TACTCGTGTGGAGTCGTCTACGAGAAGAGGGTGCACGGGAGAGGAGGATT 1304

  contig10_pilo      50197 GTGAGACGGGAGTGCGTCTTTTCTTCGTCGCTGCACTGGCGAAGTCTATG 50246
                                                                             
C rnd-4_family-       1303 GTGAGACGGGAGTGCGTCTTTTCTTCGTCGCTGCACTGGCGAAGTCTATG 1254

  contig10_pilo      50247 ACTGCGTGCGAACGCGACGAACAGAAGTAGCACGGTTCGGATGGAGAAGT 50296
                                                                             
C rnd-4_family-       1253 ACTGCGTGCGAACGCGACGAACAGAAGTAGCACGGTTCGGATGGAGAAGT 1204

  contig10_pilo      50297 CGTAGTAGGAGAGGAGGGAAGATGGTGTGCAATCACGGAAGTTGCGACTA 50346
                                              i                      i       
C rnd-4_family-       1203 CGTAGTAGGAGAGGAGGGAGGATGGTGTGCAATCACGGAAGTCGCGACTA 1154

  contig10_pilo      50347 CGCACCGAGGGAAGGGGGGAAAACGGGACTTGAGGCCAACGGAGATGGCA 50396
                                             -                               
C rnd-4_family-       1153 CGCACCGAGGGAAGGGGG-AAAACGGGACTTGAGGCCAACGGAGATGGCA 1105

  contig10_pilo      50397 GAGGGTGACTGAGACTTCGATTGCGTACGGACTAGAACGGAGGACGGGTG 50446
                                                                             
C rnd-4_family-       1104 GAGGGTGACTGAGACTTCGATTGCGTACGGACTAGAACGGAGGACGGGTG 1055

  contig10_pilo      50447 CGAGTACGAGAACTGAGCGCCGGAGTGCGCGATACTGAACTACTGGTGAG 50496
                                          i                                  
C rnd-4_family-       1054 CGAGTACGAGAACTGGGCGCCGGAGTGCGCGATACTGAACTACTGGTGAG 1005

  contig10_pilo      50497 CCGGATGCTCGTGAGCCGCTGTGCTAAGGGAAAACTACCTAACTACCTAT 50546
                                                                             
C rnd-4_family-       1004 CCGGATGCTCGTGAGCCGCTGTGCTAAGGGAAAACTACCTAACTACCTAT 955

  contig10_pilo      50547 CGCATTTTATACAGGAGAATGCCAAGCCAATCAAGACCATGTCCCGAGCT 50596
                              i                                              
C rnd-4_family-        954 CGCGTTTTATACAGGAGAATGCCAAGCCAATCAAGACCATGTCCCGAGCT 905

  contig10_pilo      50597 GTACCGATAATGCCCATGCGATAGGAAGAGTCCCTGATTGTCACATGCGG 50646
                                                          i  i               
C rnd-4_family-        904 GTACCGATAATGCCCATGCGATAGGAAGAGTTCCCGATTGTCACATGCGG 855

  contig10_pilo      50647 ACTGCGCGCGCCTGCGGGTTGCGCAATCCGTTCTCGGGAGCCAATGAGAG 50696
                                                                             
C rnd-4_family-        854 ACTGCGCGCGCCTGCGGGTTGCGCAATCCGTTCTCGGGAGCCAATGAGAG 805

  contig10_pilo      50697 GCCGATCCCGTGAGCGTGTGATTCAGAATTTGCTCCGAGGAAAGACTACC 50746
                                                                             
C rnd-4_family-        804 GCCGATCCCGTGAGCGTGTGATTCAGAATTTGCTCCGAGGAAAGACTACC 755

  contig10_pilo      50747 GATCGTCCACAAGTGTGCTGCGGCCTGCCCTGCCGTTTGGATGACGCCAT 50796
                                                                             
C rnd-4_family-        754 GATCGTCCACAAGTGTGCTGCGGCCTGCCCTGCCGTTTGGATGACGCCAT 705

  contig10_pilo      50797 AGACCGTGGGAAAGTGCGCTGCGATCACGATGACGGGATTCCCCTAGTAT 50846
                                                                             
C rnd-4_family-        704 AGACCGTGGGAAAGTGCGCTGCGATCACGATGACGGGATTCCCCTAGTAT 655

  contig10_pilo      50847 GGGTTTCGTGTTCCGCATTTCCCCGGTTTCTCAGGGTGCACATGCTCAGG 50896
                                                                             
C rnd-4_family-        654 GGGTTTCGTGTTCCGCATTTCCCCGGTTTCTCAGGGTGCACATGCTCAGG 605

  contig10_pilo      50897 GACAGTAGAGAGGAAGGATGCCCTAGACCCTGGTCTGGATATGGTCCGAG 50946
                                                                             
C rnd-4_family-        604 GACAGTAGAGAGGAAGGATGCCCTAGACCCTGGTCTGGATATGGTCCGAG 555

  contig10_pilo      50947 GTCGCAGCAGGTGACGTGTTCGCGAATGCGCAGGTGCCGATAACGATTTT 50996
                                   i                                         
C rnd-4_family-        554 GTCGCAGCGGGTGACGTGTTCGCGAATGCGCAGGTGCCGATAACGATTTT 505

  contig10_pilo      50997 GGGTGAATGAGTCAGGATCGTTCTCGGTTGCTGGTGATCCCTTGGAACCG 51046
                                                                             
C rnd-4_family-        504 GGGTGAATGAGTCAGGATCGTTCTCGGTTGCTGGTGATCCCTTGGAACCG 455

  contig10_pilo      51047 GAGACAGCGGCGTCGAGAATATTCCTTCGCAGCCAGGGCGACTGCGCACC 51096
                                                                             
C rnd-4_family-        454 GAGACAGCGGCGTCGAGAATATTCCTTCGCAGCCAGGGCGACTGCGCACC 405

  contig10_pilo      51097 TTCGTGGTCGTGGTGTCTTTGCCGGAGTAGCACGCGGTCGTGTGCTGGGA 51146
                                                                             
C rnd-4_family-        404 TTCGTGGTCGTGGTGTCTTTGCCGGAGTAGCACGCGGTCGTGTGCTGGGA 355

  contig10_pilo      51147 CACTTTCGTGAGGCGAACGCGGAACATGCTCGCCTGCTCTTGGAGACGGA 51196
                                   v                                i        
C rnd-4_family-        354 CACTTTCGGGAGGCGAACGCGGAACATGCTCGCCTGCTCTTAGAGACGGA 305

  contig10_pilo      51197 TCGTTCGTCCTTCGCGCATACTCTGGGAGTCTCGAAAGGCAAATCCGGGA 51246
                                                                             
C rnd-4_family-        304 TCGTTCGTCCTTCGCGCATACTCTGGGAGTCTCGAAAGGCAAATCCGGGA 255

  contig10_pilo      51247 GTCGTGCTTGAGGTCCAATAGAAGGGATTGTGGTTGCCGAGTCGTTGTCG 51296
                                                             i      i        
C rnd-4_family-        254 GTCGTGCTTGAGGTCCAATAGAAGGGATTGTGGTCGCCGAGCCGTTGTCG 205

  contig10_pilo      51297 CCAAGTTGTCCATGCTGGAGAGATGTCGGGTCTTGCCGGTGTGCGTCGCG 51346
                                                                             
C rnd-4_family-        204 CCAAGTTGTCCATGCTGGAGAGATGTCGGGTCTTGCCGGTGTGCGTCGCG 155

  contig10_pilo      51347 TGTTCCATTTCGGGATGCTGTTCTTGTTGATTTGCGGTCTTCGTCGTTGT 51396
                                                                             
C rnd-4_family-        154 TGTTCCATTTCGGGATGCTGTTCTTGTTGATTTGCGGTCTTCGTCGTTGT 105

  contig10_pilo      51397 CGGGATGTGTGGTAGTACTGTATTCGAGTTTGCATTGTGTCTTGCTATGT 51446
                                                           i                 
C rnd-4_family-        104 CGGGATGTGTGGTAGTACTGTATTCGAGTTTGTATTGTGTCTTGCTATGT 55

  contig10_pilo      51447 CGCCTTTATCTTGAAAGGGACGCGATTTTTCCGGCGTGTCAATTTCTGCC 51496
                                                                             
C rnd-4_family-         54 CGCCTTTATCTTGAAAGGGACGCGATTTTTCCGGCGTGTCAATTTCTGCC 5

  contig10_pilo      51497 CGAT 51500
                               
C rnd-4_family-          4 CGAT 1

Matrix = 20p53g.matrix
Kimura (with divCpGMod) = 0.79
Transitions / transversions = 3.67 (22/6)
Gap_init rate = 0.00 (2 / 2302), avg. gap size = 1.00 (2 / 2)

 
 

 
 
   +    8398   11.2  1.8  0.3  contig10_pilon    52212   53614 (1761265) +  rnd-4_family-1185  LINE/Tad1          365   1788     (0)    16     
 
 ANNOTATION EVIDENCE: 
  8398  11.22 1.78 0.28  contig10_pilon    52212   53614   1761265 +  rnd-4_family-1185  LINE/Tad1          365   1788       0      
8398 11.22 1.78 0.28 contig10_pilon 52212 53614 (1761265) rnd-4_family-1185#LINE/Tad1 365 1788 (0) m_b495s001i12

  contig10_pilo      52212 AAAAAATCCAGGAAAAGCGGTCCTATTCCCTCCACCACCTCCCAGCACCT 52261
                                i                                            
  rnd-4_family-        365 AAAAAGTCCAGGAAAAGCGGTCCTATTCCCTCCACCACCTCCCAGCACCT 414

  contig10_pilo      52262 CCCACCCGAAAGGGCTAGCTCCCAGAGTGCCTGGTTGAGCCCTGGTGAGG 52311
                                                    i                        
  rnd-4_family-        415 CCCACCCGAAAGGGCTAGCTCCCAGGGTGCCTGGTTGAGCCCTGGTGAGG 464

  contig10_pilo      52312 TGCTGCCTCAGGAGGCCGCGCATTCACCGATTTCAAACTCCGCTCCGGAT 52361
                                                i    i                       
  rnd-4_family-        465 TGCTGCCTCAGGAGGCCGCGCGTTCATCGATTTCAAACTCCGCTCCGGAT 514

  contig10_pilo      52362 ACTTCCGGACCCCCTCCTCCTGCCCCTGCACCACC--ACAGGCTCATTTC 52409
                                                       --  i  --i      vvi   
  rnd-4_family-        515 ACTTCCGGACCCCCTCCTCCTGCCCCTG--CCGCCTGGCAGGCTACCTTC 562

  contig10_pilo      52410 TCCTCC--TCTGGCCTCGATACTTCGGCTCTGGGTGCCCCTAGGGGTGCC 52457
                           vv  i --   -    -      i     v               i   i
  rnd-4_family-        563 AGCTTCAATCT-GCCT-GATACTCCGGCTGTGGGTGCCCCTAGGGATGCT 610

  contig10_pilo      52458 CTAGACACTGAGCCAGTTGCTCCTGATCAGAACATGGACTCAGACTCTGA 52507
                             ivv v  v                i   i  vv   iv i  vi vv 
  rnd-4_family-        611 CTGCCCTCTCAGCCAGTTGCTCCTGACCAGGACCAGGATACGGAGCCACA 660

  contig10_pilo      52508 GGAGGACTATGATGGAGTTGACCTTACTGCCTTTGCTTCTGATTGGGCCT 52557
                            i v    v     vvv v    i iv i vi     v    viv  viv
  rnd-4_family-        661 GAATGACTCTGATGCTCTGGACCCTGATACACTTGCTGCTGAACTGGATG 710

  contig10_pilo      52558 CTACACCCCTGACCCCCAGCCACCTCAGGAACTCTGTCAAGCTGGTCAAT 52607
                            i vi  iv     i    vv vvii   i  vi i ivvvi vv v vi
  rnd-4_family-        711 CCAAGCCTGTGACCTCCAGGAAGGCTAGGGACATTATTTCCTTCCTGACC 760

  contig10_pilo      52608 AACTTTCTAGGCATGCTAAAGGTGCTCCTTGATTACAGGGAGCGTGTTCC 52657
                           v   vi  i i    v v      v  iv    ii  i   v  iv    
  rnd-4_family-        761 TACTGCCTGGACATGGTCAAGGTGATCTATGATCGCAAGGACCGCTTTCC 810

  contig10_pilo      52658 ACAGGCTGCT---TCCATCATCAAGGACTTCTGCCACTCCGTTGTGCTGT 52704
                           vviv      ---v iiivi  i       i    i   i  v    vi 
  rnd-4_family-        811 TAGTGCTGCTGGAGCTGCAGTCGAGGACTTTTGCCGCTCTGTGGTGCAAT 860

  contig10_pilo      52705 CCTCCACCACTAGGCTGCTTCTTGCTCAGCAGGAGGAGCTTGGGAAGCTC 52754
                                   v iv             v               ii       
  rnd-4_family-        861 CCTCCACCTCCCGGCTGCTTCTTGCACAGCAGGAGGAGCTTAAGAAGCTC 910

  contig10_pilo      52755 TCTGCTATGGTAGCCTCGCTTAAGGGCGCCCCTGGAGGTGTGCCTGCTGC 52804
                                i           v   v         i   iv   ivi       
  rnd-4_family-        911 TCTGCCATGGTAGCCTCCCTTCAGGGCGCCCTTGGGTGTGCTTCTGCTGC 960

  contig10_pilo      52805 TGCATCTTCGTCTGCACCA------------------CCTGCTCCTCCTT 52836
                               v i        v   ------------------v        v  i
  rnd-4_family-        961 TGCAACCTCGTCTGCCCCAGCTGCCCAGCCTGCTGCTGCTGCTCCTGCTC 1010

  contig10_pilo      52837 GTCCTGCCAAGGACTCCTACGCTGCTCGCGCTGCCAAGTCAGCTGCACCA 52886
                                                                       v     
  rnd-4_family-       1011 GTCCTGCCAAGGACTCCTACGCTGCTCGCGCTGCCAAGTCAGCTCCACCA 1060

  contig10_pilo      52887 CCACCTGCTGTGCCCCGCGTGCCAGCCCCAGCTACTCCCCTGGCCTGCCA 52936
                                i                                i  vi ii    
  rnd-4_family-       1061 CCACCCGCTGTGCCCCGCGTGCCAGCCCCAGCTACTCCTCTTACTCGCCA 1110

  contig10_pilo      52937 CGACGCTCGCCGCCTGATTCTTATGGTCAAGCCCAGTGCCGCTGTTCGCC 52986
                                             i  i                 i          
  rnd-4_family-       1111 CGACGCTCGCCGCCTGATCCTCATGGTCAAGCCCAGTGCTGCTGTTCGCC 1160

  contig10_pilo      52987 TCGCTGTCAAGAACGCCGCCACTGCCCGCTCTGCCATCAACACCGCACAC 53036
                                              ii i                       v v 
  rnd-4_family-       1161 TCGCTGTCAAGAACGCCGCTGCCGCCCGCTCTGCCATCAACACCGCCCTC 1210

  contig10_pilo      53037 GCCGGCGTCTCGCCTCCTGCTGCACCTCATCCCAGGCTTGGGGTGAGTGG 53086
                                      v                                      
  rnd-4_family-       1211 GCCGGCGTCTCCCCTCCTGCTGCACCTCATCCCAGGCTTGGGGTGAGTGG 1260

  contig10_pilo      53087 TGTTGGCCTGACTCGCTCAGGCAACGTTGTTGTCTTTGCGCTGCCAGGTC 53136
                                                                     v       
  rnd-4_family-       1261 TGTTGGCCTGACTCGCTCAGGCAACGTTGTTGTCTTTGCGCTTCCAGGTC 1310

  contig10_pilo      53137 TCACTGCTGCCGACTTGGAGCCCCACAGCGCTCTCATCGCCTCTGCCTTC 53186
                                                                             
  rnd-4_family-       1311 TCACTGCTGCCGACTTGGAGCCCCACAGCGCTCTCATCGCCTCTGCCTTC 1360

  contig10_pilo      53187 CTGCCCCAGGAGGCCGCCTTTTCTGGTGCTGCTCGTGACAGCCTTTGGTT 53236
                                                                             
  rnd-4_family-       1361 CTGCCCCAGGAGGCCGCCTTTTCTGGTGCTGCTCGTGACAGCCTTTGGTT 1410

  contig10_pilo      53237 CAAGGCTGTTGTCTCTGATGTTGTGCCTCCTGACCTTGGCCAGCCCCTGC 53286
                                 i                                           
  rnd-4_family-       1411 CAAGGCCGTTGTCTCTGATGTTGTGCCTCCTGACCTTGGCCAGCCCCTGC 1460

  contig10_pilo      53287 CGACGTCGCAGGAGTTGCAGGCCAAGGTGGAGGAGTTTACTGCCCACCAG 53336
                                                  i                          
  rnd-4_family-       1461 CGACGTCGCAGGAGTTGCAGGCCGAGGTGGAGGAGTTTACTGCCCACCAG 1510

  contig10_pilo      53337 TTTGCCTGGGCCGCCCCTCCCATGTGGTTGGGCCCGCCTACTCGCATCAT 53386
                                                                             
  rnd-4_family-       1511 TTTGCCTGGGCCGCCCCTCCCATGTGGTTGGGCCCGCCTACTCGCATCAT 1560

  contig10_pilo      53387 CCAGCAGGGCTCTGGCTCTGTGCTACTTTCCTTCACTCGTGAAGATGACT 53436
                                                                             
  rnd-4_family-       1561 CCAGCAGGGCTCTGGCTCTGTGCTACTTTCCTTCACTCGTGAAGATGACT 1610

  contig10_pilo      53437 TGCAGTATGTCCTTCGCCCTGGTGTCTTCCTCTTTGGCCACGCTATGCAT 53486
                                  i          v                             i 
  rnd-4_family-       1611 TGCAGTACGTCCTTCGCCATGGTGTCTTCCTCTTTGGCCACGCTATGCGT 1660

  contig10_pilo      53487 GCTCGTCGCTTCCACGACTCTGGTCGTCCTTGCCCCTGCTCCAACTGCTG 53536
                                                         i                   
  rnd-4_family-       1661 GCTCGTCGCTTCCACGACTCTGGTCGTCCTCGCCCCTGCTCCAACTGCTG 1710

  contig10_pilo      53537 CAGTTTGGAGCACGCTGCTCGTGCCTGCACCCAGGCCCCTTGCTGCGGTC 53586
                                                                   i         
  rnd-4_family-       1711 CAGTTTGGAGCACGCTGCTCGTGCCTGCACCCAGGCCCCTCGCTGCGGTC 1760

  contig10_pilo      53587 TGTGTTTTCAGAGTCATTTGACTTCTGA 53614
                                 i                     
  rnd-4_family-       1761 TGTGTTCTCAGAGTCATTTGACTTCTGA 1788

Matrix = 20p53g.matrix
Kimura (with divCpGMod) = 10.97
Transitions / transversions = 1.09 (82/75)
Gap_init rate = 0.01 (8 / 1402), avg. gap size = 3.62 (29 / 8)

 
 
 
 
 
  +     699    3.3  0.0  0.0  contig10_pilon    53730   53820 (1761059) +  rnd-4_family-2951  Unknown              1     91   (266)    17     
 
 ANNOTATION EVIDENCE: 
   699   3.30 0.00 0.00  contig10_pilon    53730   53820   1761059 +  rnd-4_family-2951  Unknown              1     91     266      
699 3.30 0.00 0.00 contig10_pilon 53730 53820 (1761059) rnd-4_family-2951#Unknown 1 91 (266) m_b495s001i13

  contig10_pilo      53730 GCTTGCCGTGTCTGGACTTGCCGTGCTGGTGGTCCCAGGAGAACTAGGAG 53779
                                   i         i                               
  rnd-4_family-          1 GCTTGCCGCGTCTGGACTCGCCGTGCTGGTGGTCCCAGGAGAACTAGGAG 50

  contig10_pilo      53780 GACATCTGGTGGTGGTCAGGTGGCCACTGCCATGGATGTGA 53820
                                          i                         
  rnd-4_family-         51 GACATCTGGTGGTGGCCAGGTGGCCACTGCCATGGATGTGA 91

Matrix = 20p53g.matrix
Kimura (with divCpGMod) = 1.34
Transitions / transversions = 1.00 (3/0)
Gap_init rate = 0.00 (0 / 90), avg. gap size = 0.0 (0 / 0)

 
 

 
 
   +    6166    2.2  0.0  0.0  contig10_pilon    53825   54590 (1760289) C  rnd-4_family-2054  LINE/Tad1          (0)   1253     488    18     
 
 ANNOTATION EVIDENCE: 
  6166   2.22 0.00 0.00  contig10_pilon    53825   54590   1760289 C  rnd-4_family-2054  LINE/Tad1          488   1253       0      
6166 2.22 0.00 0.00 contig10_pilon 53825 54590 (1760289) C rnd-4_family-2054#LINE/Tad1 (0) 1253 488 m_b495s001i14

  contig10_pilo      53825 GAGAGAGAGAGAGAAATTCTTGAATTTTAGACAGCGTAAGCAGGCTACAA 53874
                                                                             
C rnd-4_family-       1253 GAGAGAGAGAGAGAAATTCTTGAATTTTAGACAGCGTAAGCAGGCTACAA 1204

  contig10_pilo      53875 ATACAGAGCCAAGACGCTGTGGTCTGTACAAAGTACATTCCAATGGGTCA 53924
                                                                             
C rnd-4_family-       1203 ATACAGAGCCAAGACGCTGTGGTCTGTACAAAGTACATTCCAATGGGTCA 1154

  contig10_pilo      53925 ACATGGGGCAAGCAAGGGGGGGTCGTAACAGGGGTGTAGAGGGATCTAGT 53974
                                     i                                       
C rnd-4_family-       1153 ACATGGGGCAGGCAAGGGGGGGTCGTAACAGGGGTGTAGAGGGATCTAGT 1104

  contig10_pilo      53975 GCAAACTGGTGTGGATTCTGGTTGTAACAAAGTTGTGACTGCAGTTCCAT 54024
                                                v                vi          
C rnd-4_family-       1103 GCAAACTGGTGTGGATTCTGGGTGTAACAAAGTTGTGAGCGCAGTTCCAT 1054

  contig10_pilo      54025 AGGGGTATAGGGGCATAGGGGCATAGAATAAATAAAAATATACAAACATA 54074
                                                                             
C rnd-4_family-       1053 AGGGGTATAGGGGCATAGGGGCATAGAATAAATAAAAATATACAAACATA 1004

  contig10_pilo      54075 CCATAAATTCCTGCGCCTCATCAGCACATAAAGTGACCAGCTCGCTAATA 54124
                                 v   v                  i               i    
C rnd-4_family-       1003 CCATAACTTCATGCGCCTCATCAGCACATGAAGTGACCAGCTCGCCAATA 954

  contig10_pilo      54125 AACAAGCAGATGTTAGCAAGTAGGCAGCTGCATGTGGCCCTTTAGGGCCA 54174
                            i                    i                           
C rnd-4_family-        953 AGCAAGCAGATGTTAGCAAGTAAGCAGCTGCATGTGGCCCTTTAGGGCCA 904

  contig10_pilo      54175 CTATGAGTGTATCTCTTGACACACTCAAGAAGAGTATCTTAGAGACTGAT 54224
                             i  i             v           i                  
C rnd-4_family-        903 CTGTGGGTGTATCTCTTGAGACACTCAAGAAAAGTATCTTAGAGACTGAT 854

  contig10_pilo      54225 AGAGGGTAGAGGGCAGAGTCTGGATGGGTGGGGCTGTAGCCTACAGACAA 54274
                                                                             
C rnd-4_family-        853 AGAGGGTAGAGGGCAGAGTCTGGATGGGTGGGGCTGTAGCCTACAGACAA 804

  contig10_pilo      54275 AAGCGCACCGCAACATGGTCAAAGCCATGTGGAGCCACGGCGCAGCTCAT 54324
                            i                                                
C rnd-4_family-        803 AGGCGCACCGCAACATGGTCAAAGCCATGTGGAGCCACGGCGCAGCTCAT 754

  contig10_pilo      54325 GGTCCTCCCAACCCTAAGGGTCCTGCGGCGGTGGCTCACCAGTTTTAGTG 54374
                                                                         i   
C rnd-4_family-        753 GGTCCTCCCAACCCTAAGGGTCCTGCGGCGGTGGCTCACCAGTTTTGGTG 704

  contig10_pilo      54375 AACGCACCCGACGAGTGGATGAATTTGGCCAAGGCAGAGATGCCCTTGCG 54424
                                                                             
C rnd-4_family-        703 AACGCACCCGACGAGTGGATGAATTTGGCCAAGGCAGAGATGCCCTTGCG 654

  contig10_pilo      54425 CGTACTGAGTAGCGCATGCGTCCCAAGCAAGTTAGGCTTGAAGGAGGCAA 54474
                                                           i                 
C rnd-4_family-        653 CGTACTGAGTAGCGCATGCGTCCCAAGCAAGTCAGGCTTGAAGGAGGCAA 604

  contig10_pilo      54475 AGTGGTGATAGTGCTCGTCATGGCGCTCGCAATCTCTAAGGATGTGATCG 54524
                                                                             
C rnd-4_family-        603 AGTGGTGATAGTGCTCGTCATGGCGCTCGCAATCTCTAAGGATGTGATCG 554

  contig10_pilo      54525 CGTGTCTGCAAGTGGTGTCCACAAGGGCAAGTCACATCTTCAGTGGGAAC 54574
                                                                             
C rnd-4_family-        553 CGTGTCTGCAAGTGGTGTCCACAAGGGCAAGTCACATCTTCAGTGGGAAC 504

  contig10_pilo      54575 ATGGCGCTGATAGTAC 54590
                                           
C rnd-4_family-        503 ATGGCGCTGATAGTAC 488

Matrix = 20p53g.matrix
Kimura (with divCpGMod) = 2.14
Transitions / transversions = 2.40 (12/5)
Gap_init rate = 0.00 (0 / 765), avg. gap size = 0.0 (0 / 0)

 
 
 
 
  +    1826    1.3  0.9  0.0  contig10_pilon    54591   54817 (1760062) C  rnd-4_family-3316  LTR/Gypsy          (0)    523     295    19     
 
 ANNOTATION EVIDENCE: 
  1826   1.30 0.87 0.00  contig10_pilon    54591   54817   1760062 C  rnd-4_family-3316  LTR/Gypsy          295    523       0      
1826 1.30 0.87 0.00 contig10_pilon 54591 54817 (1760062) C rnd-4_family-3316#LTR/Gypsy (0) 523 295 m_b495s001i15

  contig10_pilo      54591 CTGTAAGTGACTGTGCCCCCCAATTCCAGGTGGATTCCAGGTGGAAAAAT 54640
                                                                             
C rnd-4_family-        523 CTGTAAGTGACTGTGCCCCCCAATTCCAGGTGGATTCCAGGTGGAAAAAT 474

  contig10_pilo      54641 GCAACTATGCAAAAAA-GTGTCAGAATGTCAAATCCATCATATTTTGATG 54689
                                           -                                 
C rnd-4_family-        473 GCAACTATGCAAAAAAAGTGTCAGAATGTCAAATCCATCATATTTTGATG 424

  contig10_pilo      54690 GTGTACCCC-TCAGGTGGAATTTGGGTGGAATTCCACTGGACCGTTGGAT 54738
                             i      -                                        
C rnd-4_family-        423 GTATACCCCCTCAGGTGGAATTTGGGTGGAATTCCACTGGACCGTTGGAT 374

  contig10_pilo      54739 CGGATGGGCCAGAATGGCCGAATTCCGGTGGAATTCCACTGGAAACAATG 54788
                                                                             
C rnd-4_family-        373 CGGATGGGCCAGAATGGCCGAATTCCGGTGGAATTCCACTGGAAACAATG 324

  contig10_pilo      54789 TGGAAATTCATCAAAAGCTGTGATTAAGT 54817
                                           i            
C rnd-4_family-        323 TGGAAATTCATCAAAAACTGTGATTAAGT 295

Matrix = 20p53g.matrix
Kimura (with divCpGMod) = 1.33
Transitions / transversions = 1.00 (2/0)
Gap_init rate = 0.01 (2 / 226), avg. gap size = 1.00 (2 / 2)

 
 

 
   +      70    1.4  0.0  2.7  contig10_pilon    54818   54893 (1759986) +  (GTTAG)n           Simple_repeat        1     74     (0)    20     
 
 ANNOTATION EVIDENCE: 
    70   1.41 0.00 2.70  contig10_pilon    54818   54893   1759986 +  (GTTAG)n           Simple_repeat        1     74       0      
70 1.41 0.00 2.70 contig10_pilon 54818 54893 (1759986) (GTTAG)n#Simple_repeat 1 74 (0) c_b495s251i3

  contig10_pilo      54818 GTTAGGTTAGGTTAAGTTAGGTTAGGTTAGGTTAGGGGTTAGGTTAGGTT 54867
                                         i                   --              
  (GTTAG)n#Simp          1 GTTAGGTTAGGTTAGGTTAGGTTAGGTTAGGTTA--GGTTAGGTTAGGTT 48

  contig10_pilo      54868 AGGTTAGGTTAGGTTAGGTTAGGTTA 54893
                                                     
  (GTTAG)n#Simp         49 AGGTTAGGTTAGGTTAGGTTAGGTTA 74

Matrix = Unknown
Transitions / transversions = 1.00 (1/0)
Gap_init rate = 0.03 (2 / 75), avg. gap size = 1.00 (2 / 2)

 
 
 
 
  +    1826    1.8  0.0 178.5  contig10_pilon    54894   55056 (1759823) C  rnd-4_family-3316  LTR/Gypsy        (229)    294       2    19     
 
 ANNOTATION EVIDENCE: 
  1311   1.82 0.00 0.00  contig10_pilon    54894   55056   1759823 C  rnd-4_family-3316  LTR/Gypsy            2    164     359      
1311 1.82 0.00 0.00 contig10_pilon 54894 55056 (1759823) C rnd-4_family-3316#LTR/Gypsy (359) 164 2 m_b495s001i16

  contig10_pilo      54894 CTAGAACGTTTTCATGTTTCCTGAGGCGGCTGTGCAGCTCTGAACCTTAT 54943
                                                               v             
C rnd-4_family-        164 CTAGAACGTTTTCATGTTTCCTGAGGCGGCTGTGCATCTCTGAACCTTAT 115

  contig10_pilo      54944 TTGTGATGTCTAACATAATTTTTTACCACTTAGAAAATATTTTGTCTGGA 54993
                                                      v                      
C rnd-4_family-        114 TTGTGATGTCTAACATAATTTTTTACCCCTTAGAAAATATTTTGTCTGGA 65

  contig10_pilo      54994 GCAGGTTTTCCACCGGAATTCCACCTGGAATCCTCCTGGAACCAGGGGGC 55043
                                                                             
C rnd-4_family-         64 GCAGGTTTTCCACCGGAATTCCACCTGGAATCCTCCTGGAACCAGGGGGC 15

  contig10_pilo      55044 AGTGTTAGGTTCT 55056
                                i       
C rnd-4_family-         14 AGTGTCAGGTTCT 2

Matrix = 20p53g.matrix
Kimura (with divCpGMod) = 1.85
Transitions / transversions = 0.50 (1/2)
Gap_init rate = 0.00 (0 / 162), avg. gap size = 0.0 (0 / 0)

  1826   1.30 0.87 0.00  contig10_pilon    54894   54895   1759984 C  rnd-4_family-3316  LTR/Gypsy          293    294     229      
1826 1.30 0.87 0.00 contig10_pilon 54894 54895 (1759984) C rnd-4_family-3316#LTR/Gypsy (229) 294 293 m_b495s001i15

  contig10_pilo      54894 CT 54895
                           i 
C rnd-4_family-        294 TT 293

Matrix = 20p53g.matrix
Kimura (with divCpGMod) = 1.33
Transitions / transversions = 1.00 (1/0)
Gap_init rate = 0.00 (0 / 1), avg. gap size = 0.0 (0 / 0)

 
 

 
   +    2657    0.6  0.0  0.0  contig10_pilon    55058   55378 (1759501) C  rnd-4_family-2054  LINE/Tad1        (758)    495     175    18     
 
 ANNOTATION EVIDENCE: 
  2657   0.62 0.00 0.00  contig10_pilon    55058   55378   1759501 C  rnd-4_family-2054  LINE/Tad1          175    495     758      
2657 0.62 0.00 0.00 contig10_pilon 55058 55378 (1759501) C rnd-4_family-2054#LINE/Tad1 (758) 495 175 m_b495s001i17

  contig10_pilo      55058 GATAGTACTCGCCCATGAACGCATGTCCAAGGCGGCACTGCATGACCAGT 55107
                                                                             
C rnd-4_family-        495 GATAGTACTCGCCCATGAACGCATGTCCAAGGCGGCACTGCATGACCAGT 446

  contig10_pilo      55108 CCATAAAGATGGCGAGGCAGCTCACCAAAGTGAGGTGGAGGGCGCCTCTT 55157
                                    i                                        
C rnd-4_family-        445 CCATAAAGACGGCGAGGCAGCTCACCAAAGTGAGGTGGAGGGCGCCTCTT 396

  contig10_pilo      55158 GGGTGGCCCATAGGCTGCCCTGGCATACAAACCCCGAGGAGGTGACGCTG 55207
                                                                         v   
C rnd-4_family-        395 GGGTGGCCCATAGGCTGCCCTGGCATACAAACCCCGAGGAGGTGACTCTG 346

  contig10_pilo      55208 CCCACTCCTCTATCCAAGTCTCCATGACACGCTCCTTGGCACGTCTTTTG 55257
                                                                             
C rnd-4_family-        345 CCCACTCCTCTATCCAAGTCTCCATGACACGCTCCTTGGCACGTCTTTTG 296

  contig10_pilo      55258 AGGTGGGACACAGTGGCCCATGAGGGACCTTGCATGCGAGCTGCCTCCTT 55307
                                                                             
C rnd-4_family-        295 AGGTGGGACACAGTGGCCCATGAGGGACCTTGCATGCGAGCTGCCTCCTT 246

  contig10_pilo      55308 GGCTAACTCATCTGCACGCTCATTGCCCCTTATGCCAATATGGCTGGGGA 55357
                                                                             
C rnd-4_family-        245 GGCTAACTCATCTGCACGCTCATTGCCCCTTATGCCAATATGGCTGGGGA 196

  contig10_pilo      55358 CCCAAGAGATGGTCACCTCAT 55378
                                                
C rnd-4_family-        195 CCCAAGAGATGGTCACCTCAT 175

Matrix = 20p53g.matrix
Kimura (with divCpGMod) = 0.34
Transitions / transversions = 1.00 (1/1)
Gap_init rate = 0.00 (0 / 320), avg. gap size = 0.0 (0 / 0)

 
 
 
  +    1477    0.0  0.0  0.0  contig10_pilon    56285   56465 (1758414) C  rnd-4_family-2054  LINE/Tad1       (1072)    181       1    18     
 
 ANNOTATION EVIDENCE: 
  1477   0.00 0.00 0.00  contig10_pilon    56285   56465   1758414 C  rnd-4_family-2054  LINE/Tad1            1    181    1072      
1477 0.00 0.00 0.00 contig10_pilon 56285 56465 (1758414) C rnd-4_family-2054#LINE/Tad1 (1072) 181 1 m_b495s001i18

  contig10_pilo      56285 ACCTCATGAGAAGCATCAGCAGCAAGGAAGTCACGAGCATGCTCGCAGAA 56334
                                                                             
C rnd-4_family-        181 ACCTCATGAGAAGCATCAGCAGCAAGGAAGTCACGAGCATGCTCGCAGAA 132

  contig10_pilo      56335 AGAAATGGAAAACTGCTGGGCAGAAGCAGGACGCTCCTTGACAATGGCCT 56384
                                                                             
C rnd-4_family-        131 AGAAATGGAAAACTGCTGGGCAGAAGCAGGACGCTCCTTGACAATGGCCT 82

  contig10_pilo      56385 CAACAGCTGAAGAGTTGTCAGCAAAGAAGTGAAGGTGGGAGGCCGAGCCC 56434
                                                                             
C rnd-4_family-         81 CAACAGCTGAAGAGTTGTCAGCAAAGAAGTGAAGGTGGGAGGCCGAGCCC 32

  contig10_pilo      56435 ATGGGCAAGTCCATGTAATGGGCAACATGGT 56465
                                                          
C rnd-4_family-         31 ATGGGCAAGTCCATGTAATGGGCAACATGGT 1

Matrix = 20p53g.matrix
Kimura (with divCpGMod) = 0.00
Transitions / transversions = 1.00 (0/0)
Gap_init rate = 0.00 (0 / 180), avg. gap size = 0.0 (0 / 0)

 
 

 
 
   +    4457    1.5  0.0  0.0  contig10_pilon    56466   57010 (1757869) C  rnd-3_family-161   LINE/Tad1          (0)   4561    4017    21     
 
 ANNOTATION EVIDENCE: 
  4457   1.47 0.00 0.00  contig10_pilon    56466   57010   1757869 C  rnd-3_family-161   LINE/Tad1         4017   4561       0      
4457 1.47 0.00 0.00 contig10_pilon 56466 57010 (1757869) C rnd-3_family-161#LINE/Tad1 (0) 4561 4017 m_b495s001i19

  contig10_pilo      56466 CCATGGCAGCAGAGAGTGCATGCATCTCAGTATCATAGACCTCAGCCTGG 56515
                                           v             i                   
C rnd-3_family-       4561 CCATGGCAGCAGAGAGGGCATGCATCTCAGCATCATAGACCTCAGCCTGG 4512

  contig10_pilo      56516 GACCCCATAGGGATGCGACTGCAAGACTGCTCCTGACCCTCCAGGTAGGT 56565
                                              i                              
C rnd-3_family-       4511 GACCCCATAGGGATGCGACCGCAAGACTGCTCCTGACCCTCCAGGTAGGT 4462

  contig10_pilo      56566 CACAAAAGCTGCACCAGTCCTGCGACCAGTTGGAGTGTCTAAAAGGGAAC 56615
                                                                             
C rnd-3_family-       4461 CACAAAAGCTGCACCAGTCCTGCGACCAGTTGGAGTGTCTAAAAGGGAAC 4412

  contig10_pilo      56616 CATCAGTGTAGATCAGCAGGTTTGCAGAGCTGAGTTCCAGCATCTGCACA 56665
                                                                             
C rnd-3_family-       4411 CATCAGTGTAGATCAGCAGGTTTGCAGAGCTGAGTTCCAGCATCTGCACA 4362

  contig10_pilo      56666 AGTCTGACATGGGACTCTGCAGCATCAGCTTTGCTGACACCCTTTGGCAC 56715
                                                              v              
C rnd-3_family-       4361 AGTCTGACATGGGACTCTGCAGCATCAGCTTTGCTCACACCCTTTGGCAC 4312

  contig10_pilo      56716 AGGCCTGAGGTGGATGCGACCTGGAAAAGCTGCAAGCAGAGAAATGGCCC 56765
                                                                             
C rnd-3_family-       4311 AGGCCTGAGGTGGATGCGACCTGGAAAAGCTGCAAGCAGAGAAATGGCCC 4262

  contig10_pilo      56766 ATGGGGCAATGTGGAAGGGCTCAATGCGCTCATTGCCAGGTGAGGTGAGC 56815
                                                                             
C rnd-3_family-       4261 ATGGGGCAATGTGGAAGGGCTCAATGCGCTCATTGCCAGGTGAGGTGAGC 4212

  contig10_pilo      56816 TTGGCAAGGTAGCGAAGAGAGGAGCGAGTTTGACCACGCTTGTGCACCAC 56865
                                                                             
C rnd-3_family-       4211 TTGGCAAGGTAGCGAAGAGAGGAGCGAGTTTGACCACGCTTGTGCACCAC 4162

  contig10_pilo      56866 TGGTGGCTTCTCCATTGGAGACTGGTGCCAGGCAGGGTGCAGGCGCTGCA 56915
                                                                             
C rnd-3_family-       4161 TGGTGGCTTCTCCATTGGAGACTGGTGCCAGGCAGGGTGCAGGCGCTGCA 4112

  contig10_pilo      56916 AGACAGGACTGTAAGGCCCCAACTTGTGCAGGTGAGCAGCAGCAAGGTCA 56965
                                      ii               i   i                 
C rnd-3_family-       4111 AGACAGGACTGCGAGGCCCCAACTTGTGTAGGCGAGCAGCAGCAAGGTCA 4062

  contig10_pilo      56966 TCGGCCTTGTCTAATACCAAATGTATAGGTGGAATAGCTGCCTCC 57010
                                                                        
C rnd-3_family-       4061 TCGGCCTTGTCTAATACCAAATGTATAGGTGGAATAGCTGCCTCC 4017

Matrix = 20p53g.matrix
Kimura (with divCpGMod) = 0.96
Transitions / transversions = 3.00 (6/2)
Gap_init rate = 0.00 (0 / 544), avg. gap size = 0.0 (0 / 0)

 
 
 
 
 
  +     876    1.8  0.0  0.0  contig10_pilon    57010   57122 (1757757) C  rnd-4_family-2951  Unknown          (244)    113       1    22 *   
 
 ANNOTATION EVIDENCE: 
   876   1.77 0.00 0.00  contig10_pilon    57010   57122   1757757 C  rnd-4_family-2951  Unknown              1    113     244      
876 1.77 0.00 0.00 contig10_pilon 57010 57122 (1757757) C rnd-4_family-2951#Unknown (244) 113 1 m_b495s001i20

  contig10_pilo      57010 CTCAGGGGGAAAGCAAATCAAGTCACATCCATCGCAGTGGCCACCTGGCC 57059
                                                           v                 
C rnd-4_family-        113 CTCAGGGGGAAAGCAAATCAAGTCACATCCATGGCAGTGGCCACCTGGCC 64

  contig10_pilo      57060 ACCACCAGATGTCCTCCTAGTTCTCCTGGGACCACCAGCAAGGCGAGTCC 57109
                                                                   v         
C rnd-4_family-         63 ACCACCAGATGTCCTCCTAGTTCTCCTGGGACCACCAGCACGGCGAGTCC 14

  contig10_pilo      57110 AGACGCGGCAAGC 57122
                                        
C rnd-4_family-         13 AGACGCGGCAAGC 1

Matrix = 20p53g.matrix
Kimura (with divCpGMod) = 1.79
Transitions / transversions = 0.00 (0/2)
Gap_init rate = 0.00 (0 / 112), avg. gap size = 0.0 (0 / 0)

 
 

 
 
   +    9925    9.6  1.7  0.4  contig10_pilon    57238   58842 (1756037) C  rnd-4_family-1185  LINE/Tad1          (0)   1788     163    23     
 
 ANNOTATION EVIDENCE: 
  9925   9.57 1.74 0.43  contig10_pilon    57238   58842   1756037 C  rnd-4_family-1185  LINE/Tad1          163   1788       0      
9925 9.57 1.74 0.43 contig10_pilon 57238 58842 (1756037) C rnd-4_family-1185#LINE/Tad1 (0) 1788 163 m_b496s001i2

  contig10_pilo      57238 TCAGAAGTCAAATGACTCTGAAAACACAGACCGCAGCAAGGGGCCTGGGT 57287
                                                i               i            
C rnd-4_family-       1788 TCAGAAGTCAAATGACTCTGAGAACACAGACCGCAGCGAGGGGCCTGGGT 1739

  contig10_pilo      57288 GCAGGCACGAGCAGCGTGCTCCAAACTGCAGCAGTTGGAGCAGGGGCAAG 57337
                                                                          i  
C rnd-4_family-       1738 GCAGGCACGAGCAGCGTGCTCCAAACTGCAGCAGTTGGAGCAGGGGCGAG 1689

  contig10_pilo      57338 GACGACCAGAGTCGTGGAAGCGACGAGCATGCATAGCGTGGCCAAAGAGG 57387
                                                        i                    
C rnd-4_family-       1688 GACGACCAGAGTCGTGGAAGCGACGAGCACGCATAGCGTGGCCAAAGAGG 1639

  contig10_pilo      57388 AAGACACCAGGGCGAAGGACATACTGCAAGTCATCTTCACGAGTGAAGGA 57437
                                    v          i                             
C rnd-4_family-       1638 AAGACACCATGGCGAAGGACGTACTGCAAGTCATCTTCACGAGTGAAGGA 1589

  contig10_pilo      57438 AAGTAGCACAGAGCCAGAGCCCTGCTGGATGATGCGAGTAGGCGGGCCCA 57487
                                                                             
C rnd-4_family-       1588 AAGTAGCACAGAGCCAGAGCCCTGCTGGATGATGCGAGTAGGCGGGCCCA 1539

  contig10_pilo      57488 ACCACATGGGAGGGGCGGCCCAGGCAAACTGGTGGGCAGTAAACTCCTCC 57537
                                                                             
C rnd-4_family-       1538 ACCACATGGGAGGGGCGGCCCAGGCAAACTGGTGGGCAGTAAACTCCTCC 1489

  contig10_pilo      57538 ACCTCGGCCTGCAACTCCTGCAACGTCGGCAGGGGCTGGCCAAGGTCAGG 57587
                                                i                            
C rnd-4_family-       1488 ACCTCGGCCTGCAACTCCTGCGACGTCGGCAGGGGCTGGCCAAGGTCAGG 1439

  contig10_pilo      57588 AGGCACAACATCAGAGACGACGGCCTTGAACCAAAGGCTGTCACGAGCAG 57637
                                             i                               
C rnd-4_family-       1438 AGGCACAACATCAGAGACAACGGCCTTGAACCAAAGGCTGTCACGAGCAG 1389

  contig10_pilo      57638 CACCAGAAAAGGCGGCCTCCTGGGGCAGGAAGGCAGAGGCAATGAGCGCA 57687
                                                                   i     v  i
C rnd-4_family-       1388 CACCAGAAAAGGCGGCCTCCTGGGGCAGGAAGGCAGAGGCGATGAGAGCG 1339

  contig10_pilo      57688 CTGTGGGGCTCCAAGTCGGCAGCCGTGAGACCTGGAAGCGCAAAGACAAC 57737
                                                  v                          
C rnd-4_family-       1338 CTGTGGGGCTCCAAGTCGGCAGCAGTGAGACCTGGAAGCGCAAAGACAAC 1289

  contig10_pilo      57738 AACGTTGCCTGAGCGAGTCAGGCCAACACCACTCACCCCAAGCCTGGGAT 57787
                                                                             
C rnd-4_family-       1288 AACGTTGCCTGAGCGAGTCAGGCCAACACCACTCACCCCAAGCCTGGGAT 1239

  contig10_pilo      57788 GAGGCGCAGCAGGAGGGGAGATGCCGGCGAGGGCAGTGTTGATGGCAGAG 57837
                               i                i            i               
C rnd-4_family-       1238 GAGGTGCAGCAGGAGGGGAGACGCCGGCGAGGGCGGTGTTGATGGCAGAG 1189

  contig10_pilo      57838 CGGGCGGCAGCAGCGTTCTTGACTGCAAGGCGAACAGCAGTACTGGGCTT 57887
                                      i           v  i             i         
C rnd-4_family-       1188 CGGGCGGCAGCGGCGTTCTTGACAGCGAGGCGAACAGCAGCACTGGGCTT 1139

  contig10_pilo      57888 GACCATGAGGATCAGGCGGCGAGCGTCGTGGCGGGCAAGAGGAGTAGCTG 57937
                                                            i i              
C rnd-4_family-       1138 GACCATGAGGATCAGGCGGCGAGCGTCGTGGCGAGTAAGAGGAGTAGCTG 1089

  contig10_pilo      57938 GGGCTGGCACGCGGGGCACAGCAGGTGGTGGTGCAGCTGACTTGGCGGCG 57987
                                                 i          v            i   
C rnd-4_family-       1088 GGGCTGGCACGCGGGGCACAGCGGGTGGTGGTGGAGCTGACTTGGCAGCG 1039

  contig10_pilo      57988 CGAGCAGCGTAGGAGTCCTTGGCAGGACGAGGAGGAGCAGG--------- 58028
                                                          v        v---------
C rnd-4_family-       1038 CGAGCAGCGTAGGAGTCCTTGGCAGGACGAGCAGGAGCAGCAGCAGCAGG 989

  contig10_pilo      58029 ---------TGGTGCAGACGAAGATGCAGCAGCAGGCACACCTCCAGGGG 58069
                           ---------   v        i v           ivi   vi   i   
C rnd-4_family-        988 CTGGGCAGCTGGGGCAGACGAGGTTGCAGCAGCAGAAGCACACCCAAGGG 939

  contig10_pilo      58070 CGCCCTTAAGCGAGGCTACCATAGCAGAGAGCTTCCCAAGCTCCTCCTGC 58119
                                 v   v           i            ii             
C rnd-4_family-        938 CGCCCTGAAGGGAGGCTACCATGGCAGAGAGCTTCTTAAGCTCCTCCTGC 889

  contig10_pilo      58120 TGAGCAAGAAGCAGCCTAGTGGTGGAGGACAGCACAACAGAGTGGCAGAA 58169
                             v             vi v         iv    v      i    i  
C rnd-4_family-        888 TGTGCAAGAAGCAGCCGGGAGGTGGAGGATTGCACCACAGAGCGGCAAAA 839

  contig10_pilo      58170 GTCCTTGATGATGG---AAGCAGCCTGAGGAATACGCTCCCTGCAATCAA 58216
                                i  iviii ---v      viv     vi  v   i   i    v
C rnd-4_family-        838 GTCCTCGACTGCAGCTCCAGCAGCACTAGGAAAGCGGTCCTTGCGATCAT 789

  contig10_pilo      58217 GGAGCACCTTTAGCATGCCCAAAAGCTCATTGACCAGCGTGACAGAGTTC 58266
                           i  v      v v    i   iiviv viv v vv iv vi i iv  i 
C rnd-4_family-        788 AGATCACCTTGACCATGTCCAGGCAGTAGGTCAGGAAGGAAATAATGTCC 739

  contig10_pilo      58267 CTGAGGTCGCGAGGAGTCAGGGGT---GCACAGGCCCAATCAGCAGCAAA 58313
                             iivv iv vi ii    vi  i---   i---  viv          i
C rnd-4_family-        738 CTAGCCTTCCTGGAGGTCACAGGCTTGGCAT---CCAGTTCAGCAGCAAG 692

  contig10_pilo      58314 GGCAGCAAGGTCAAATCCTTCATAGTCATCCTCGGAGTCTGAGTCCATGT 58363
                           v i v  i    v ivv v   v      i  vv iv  i vi   vv  
C rnd-4_family-        691 TGTATCAGGGTCCAGAGCATCAGAGTCATTCTGTGGCTCCGTATCCTGGT 642

  contig10_pilo      58364 CCTGATCAGGAGCAACTGGCTCAGTCTCCAGGGCACCCCTAGGGGCACCC 58413
                               i                v  vvvv   i   i              
C rnd-4_family-        641 CCTGGTCAGGAGCAACTGGCTGAGAGGGCAGAGCATCCCTAGGGGCACCC 592

  contig10_pilo      58414 AGAGCCAAAGTATCGAGGCCAGA--GGAGGAGAAATGAGCCTGT--GGTG 58459
                            v    ii      -   -    -- i  vv   ivv      i--  i 
C rnd-4_family-        591 ACAGCCGGAGTATC-AGG-CAGATTGAAGCTGAAGGTAGCCTGCCAGGCG 544

  contig10_pilo      58460 GTGCAGGGGCAGGAGGAGGGGGTCCGGAAGTATCCGGAGCGGAGTTTGAA 58509
                           --                                                
C rnd-4_family-        543 --GCAGGGGCAGGAGGAGGGGGTCCGGAAGTATCCGGAGCGGAGTTTGAA 496

  contig10_pilo      58510 ATCGGTGAACGCGCGGCCTCCTGAGGCAGCACCTCACCAGGGCTCAACCA 58559
                               i                                             
C rnd-4_family-        495 ATCGATGAACGCGCGGCCTCCTGAGGCAGCACCTCACCAGGGCTCAACCA 446

  contig10_pilo      58560 GGCACTCTGGGAGCTAGCCCTTTCGGGTGGGAGGTGCTGGGAGGTGGTGG 58609
                                i                                            
C rnd-4_family-        445 GGCACCCTGGGAGCTAGCCCTTTCGGGTGGGAGGTGCTGGGAGGTGGTGG 396

  contig10_pilo      58610 AGGGCATAGGACCGCTTTTCCTGGACTTTTTAGGCCCAGAAATGCACCCA 58659
                               v                                        i    
C rnd-4_family-        395 AGGGAATAGGACCGCTTTTCCTGGACTTTTTAGGCCCAGAAATGCGCCCA 346

  contig10_pilo      58660 GGAGGTGGTGCAGACCCAGCAGAACCTGCAAGTTGCCCAGGACTAGCCAA 58709
                                                                            i
C rnd-4_family-        345 GGAGGTGGTGCAGACCCAGCAGAACCTGCAAGTTGCCCAGGACTAGCCAG 296

  contig10_pilo      58710 GGCATCTACAGGCCGCTTAAGGGGGGCAAGGGAGGGGGGAGTGGTCCTAG 58759
                           i   i                                             
C rnd-4_family-        295 AGCACCTACAGGCCGCTTAAGGGGGGCAAGGGAGGGGGGAGTGGTCCTAG 246

  contig10_pilo      58760 AGGGAGGGCCAGGGCCAGAGGTGGGGCCAGGGGCAGTGGTGGGGGCTGGA 58809
                                                                             
C rnd-4_family-        245 AGGGAGGGCCAGGGCCAGAGGTGGGGCCAGGGGCAGTGGTGGGGGCTGGA 196

  contig10_pilo      58810 GTGGCCAGGGCAGGTTGGGCTGGAGCACCCAAG 58842
                                                            
C rnd-4_family-        195 GTGGCCAGGGCAGGTTGGGCTGGAGCACCCAAG 163

Matrix = 20p53g.matrix
Kimura (with divCpGMod) = 9.04
Transitions / transversions = 1.22 (84/69)
Gap_init rate = 0.01 (13 / 1604), avg. gap size = 2.69 (35 / 13)

 
 
 
 
 
  +     295   37.1  0.0  0.0  contig10_pilon    59670   59812 (1755067) +  rnd-4_family-1458  LTR/Gypsy        11813  11955   (428)    24     
 
 ANNOTATION EVIDENCE: 
   295  37.06 0.00 0.00  contig10_pilon    59670   59812   1755067 +  rnd-4_family-1458  LTR/Gypsy        11813  11955     428      
295 37.06 0.00 0.00 contig10_pilon 59670 59812 (1755067) rnd-4_family-1458#LTR/Gypsy 11813 11955 (428) m_b496s001i3

  contig10_pilo      59670 GGCATGTGCGTCATCGCAATCTTTGGCAAGTTCAACCACGAGAAGCATGG 59719
                                v         iiiiiv  i  i  i v     vvi   vviv   
  rnd-4_family-      11813 GGCATCTGCGTCATCATGGCGTTCGGTAAATACAACCCGAAGACTTCTGG 11862

  contig10_pilo      59720 CCTCCTCATCCTTCGCAAGCTCAAGATCGCCATGCAACTTGCGCCGGGCG 59769
                             vii     v vvivi   iv  vi   i v vv ii i  v     i 
  rnd-4_family-      11863 CCATTTCATCATGAAAGAGCCGAACGTCGTCCTCGAGTTCGCCCCGGGTG 11912

  contig10_pilo      59770 ATGTTGTGTTTATTCCGTCCACGCTCATCACGCACGGCAACAC 59812
                             i  v    ii vvi   vi  i v     i         i 
  rnd-4_family-      11913 ATATTCTGTTCGTGATGTCGGCGTTGATCACACACGGCAACGC 11955

Matrix = 20p53g.matrix
Kimura (with divCpGMod) = 43.30
Transitions / transversions = 1.21 (29/24)
Gap_init rate = 0.00 (0 / 142), avg. gap size = 0.0 (0 / 0)

 
 

 
 
   +   14062    1.1  0.0  0.0  contig10_pilon    60614   62350 (1752529) +  rnd-4_family-2631  Unknown              1   1737     (0)    25     
 
 ANNOTATION EVIDENCE: 
 14062   1.15 0.00 0.00  contig10_pilon    60614   62350   1752529 +  rnd-4_family-2631  Unknown              1   1737       0      
14062 1.15 0.00 0.00 contig10_pilon 60614 62350 (1752529) rnd-4_family-2631#Unknown 1 1737 (0) m_b496s001i4

  contig10_pilo      60614 TTCTTGAACTCCGACTTCCACCAAGTCCGAGTCAGTGGAGCAATTCTCGC 60663
                                                                             
  rnd-4_family-          1 TTCTTGAACTCCGACTTCCACCAAGTCCGAGTCAGTGGAGCAATTCTCGC 50

  contig10_pilo      60664 TTTTCCAGCGCGCCCCCTCATCCGCCTCTTCGATCTAACGATCATGGCAT 60713
                                           i                                 
  rnd-4_family-         51 TTTTCCAGCGCGCCCCTTCATCCGCCTCTTCGATCTAACGATCATGGCAT 100

  contig10_pilo      60714 CTTCCTGGACCCACCAGGAGTCTATCGAGCCTGGGCGCGACCCTCCTGGG 60763
                                                          i                  
  rnd-4_family-        101 CTTCCTGGACCCACCAGGAGTCTATCGAGCCCGGGCGCGACCCTCCTGGG 150

  contig10_pilo      60764 CCGGTGGATCGCGAGAAATCGCAGGTGCGAGTTCCAGACAAAACTAGGAC 60813
                                                                        i    
  rnd-4_family-        151 CCGGTGGATCGCGAGAAATCGCAGGTGCGAGTTCCAGACAAAACTGGGAC 200

  contig10_pilo      60814 TCCTAGAGCGCACCACCACTCCCCCTCTGCTTCCCTCGCTTCCATGGAGC 60863
                                                      i                      
  rnd-4_family-        201 TCCTAGAGCGCACCACCACTCCCCCTCCGCTTCCCTCGCTTCCATGGAGC 250

  contig10_pilo      60864 TAGATAACGACCCTGCGCACTCCCCCACGACTCTGCAGCGACTTGGCAGC 60913
                                                                             
  rnd-4_family-        251 TAGATAACGACCCTGCGCACTCCCCCACGACTCTGCAGCGACTTGGCAGC 300

  contig10_pilo      60914 TTTGTGCGGTCGGGTGTACGCCGGTTGACCCCCAGGAGCACCAGGAGCAC 60963
                                                                  iv         
  rnd-4_family-        301 TTTGTGCGGTCGGGTGTACGCCGGTTGACCCCCAGGAGCGGCAGGAGCAC 350

  contig10_pilo      60964 CAGGAACGAGCTGCAGAGCAGGCAGCTCACTAGGAAGCGGTCTGACAGCC 61013
                                                                             
  rnd-4_family-        351 CAGGAACGAGCTGCAGAGCAGGCAGCTCACTAGGAAGCGGTCTGACAGCC 400

  contig10_pilo      61014 CTCCTAGACACTCAGGTGCGCCCCAGCCAGTTTCCAAGCGTTATAGACGC 61063
                                                           i                 
  rnd-4_family-        401 CTCCTAGACACTCAGGTGCGCCCCAGCCAGTTCCCAAGCGTTATAGACGC 450

  contig10_pilo      61064 TCCTCCTTTTGTTCTTCGCATCCTGGGGTCGAGACGAGCCAGGAGGCTCG 61113
                                    i                           i            
  rnd-4_family-        451 TCCTCCTTTCGTTCTTCGCATCCTGGGGTCGAGACGAACCAGGAGGCTCG 500

  contig10_pilo      61114 CTTTGCAGCCCCTGGGGACGACCCCTTCGTTTCTGGACTCGATTTTCGCG 61163
                                                                             
  rnd-4_family-        501 CTTTGCAGCCCCTGGGGACGACCCCTTCGTTTCTGGACTCGATTTTCGCG 550

  contig10_pilo      61164 CACACACATCTCCTACATCCCCTCCTGCATTCCACACCATTCCTGGCACC 61213
                                        i                                    
  rnd-4_family-        551 CACACACATCTCCCACATCCCCTCCTGCATTCCACACCATTCCTGGCACC 600

  contig10_pilo      61214 AGAGCGCACTCTGTGCGCGTCCCAGGCTCGTTTTTGCGCGAGGGTGACGT 61263
                                                                             
  rnd-4_family-        601 AGAGCGCACTCTGTGCGCGTCCCAGGCTCGTTTTTGCGCGAGGGTGACGT 650

  contig10_pilo      61264 GGCCCCATCGGCTCCGGCCGGAGCGGACCGGTACCGCCTCCAGGACGCGC 61313
                                                                        v    
  rnd-4_family-        651 GGCCCCATCGGCTCCGGCCGGAGCGGACCGGTACCGCCTCCAGGAAGCGC 700

  contig10_pilo      61314 CAGAACGCGCAGGAGGAGCCCAGCAGTCCCCTCCAGCCTCCCCAACTCGA 61363
                            i                                                
  rnd-4_family-        701 CGGAACGCGCAGGAGGAGCCCAGCAGTCCCCTCCAGCCTCCCCAACTCGA 750

  contig10_pilo      61364 GCTGCACACTCTGGTGTGCAGCAGCTGGCTGTACTGACTCCAAACCGCTC 61413
                                                                             
  rnd-4_family-        751 GCTGCACACTCTGGTGTGCAGCAGCTGGCTGTACTGACTCCAAACCGCTC 800

  contig10_pilo      61414 AGAACACGCTCCTACCGCTTCAATGCGGCCCACAGTGTCTGCTCCTCGCC 61463
                                                                             
  rnd-4_family-        801 AGAACACGCTCCTACCGCTTCAATGCGGCCCACAGTGTCTGCTCCTCGCC 850

  contig10_pilo      61464 CCCTTAGTGCACTGCACACTGGGGACGAGCCTGCTGCACTCACAACATCT 61513
                                                                             
  rnd-4_family-        851 CCCTTAGTGCACTGCACACTGGGGACGAGCCTGCTGCACTCACAACATCT 900

  contig10_pilo      61514 GCTGTGCGCTCTGGGGAGTCCAAGGATGCCCTGGGCGCAATTCCGGCTCG 61563
                                           v                                 
  rnd-4_family-        901 GCTGTGCGCTCTGGGGTGTCCAAGGATGCCCTGGGCGCAATTCCGGCTCG 950

  contig10_pilo      61564 TTCAACGGGCATTGGTATCGATGACCGGCTGGGGGCGTCAGGAAAGGTGG 61613
                                                                             
  rnd-4_family-        951 TTCAACGGGCATTGGTATCGATGACCGGCTGGGGGCGTCAGGAAAGGTGG 1000

  contig10_pilo      61614 ATGGGCTCGTCCTCGAGCAGGGCGCGGCATATCGAGCCACTAACTCACCT 61663
                                                    v                i       
  rnd-4_family-       1001 ATGGGCTCGTCCTCGAGCAGGGCGCCGCATATCGAGCCACTAGCTCACCT 1050

  contig10_pilo      61664 GCTGCGCCCAAAACCGTTGAAACGAGTTCGGCCTCACCAGAACAGGCTGA 61713
                                            i                                
  rnd-4_family-       1051 GCTGCGCCCAAAACCGTCGAAACGAGTTCGGCCTCACCAGAACAGGCTGA 1100

  contig10_pilo      61714 GGGAGCATCCAGAATGCAGGGCCTCGTTCCCACCGCTTCTGGACGCGCTC 61763
                                                                             
  rnd-4_family-       1101 GGGAGCATCCAGAATGCAGGGCCTCGTTCCCACCGCTTCTGGACGCGCTC 1150

  contig10_pilo      61764 CTCGAGTTGCTGCACATGGCAGCGGCTCAGATCGCGCCCCCTCGGTCAGA 61813
                                                                             
  rnd-4_family-       1151 CTCGAGTTGCTGCACATGGCAGCGGCTCAGATCGCGCCCCCTCGGTCAGA 1200

  contig10_pilo      61814 AATGACTGGGGTGTCGACTCAGCTCCTGGAGAGCCTCAATCGGCGCATAA 61863
                                                                             
  rnd-4_family-       1201 AATGACTGGGGTGTCGACTCAGCTCCTGGAGAGCCTCAATCGGCGCATAA 1250

  contig10_pilo      61864 CTGGGGCAGTTCTGCCCCAGGTGAACCACAGGCAACCAAGTTGTCTTCTG 61913
                                                                             
  rnd-4_family-       1251 CTGGGGCAGTTCTGCCCCAGGTGAACCACAGGCAACCAAGTTGTCTTCTG 1300

  contig10_pilo      61914 CTGCAACAAACTTGCCCTATGCTCGCCCTAGCACCTCAATCCCCCATGGG 61963
                                     v                                   v   
  rnd-4_family-       1301 CTGCAACAAAGTTGCCCTATGCTCGCCCTAGCACCTCAATCCCCCAAGGG 1350

  contig10_pilo      61964 GAGACTATGTCAGTCTGTGGGGACGAGGACATGCAGTCTGTGCACTCACT 62013
                                               v                           v 
  rnd-4_family-       1351 GAGACTATGTCAGTCTGTGGTGACGAGGACATGCAGTCTGTGCACTCAGT 1400

  contig10_pilo      62014 TCAGGACTGGAGGGCACAGAATGTGCCAGCCAACTTGGGCTGTCTGCCAG 62063
                                                                             
  rnd-4_family-       1401 TCAGGACTGGAGGGCACAGAATGTGCCAGCCAACTTGGGCTGTCTGCCAG 1450

  contig10_pilo      62064 ACGGCACACCACTTGAGGAGGCAGTCAATGACATCAGGTCTGACCTGCAG 62113
                                                                             
  rnd-4_family-       1451 ACGGCACACCACTTGAGGAGGCAGTCAATGACATCAGGTCTGACCTGCAG 1500

  contig10_pilo      62114 CACCTCATTGCACGTGCCACTGCAGTCAATGCGGCTGTCTGGAACCAGGA 62163
                                                                             
  rnd-4_family-       1501 CACCTCATTGCACGTGCCACTGCAGTCAATGCGGCTGTCTGGAACCAGGA 1550

  contig10_pilo      62164 AGACCAGTACTGGCGCTGGGCAGACCTGTGGACAGCAGAGCGTGTCACTG 62213
                                                                             
  rnd-4_family-       1551 AGACCAGTACTGGCGCTGGGCAGACCTGTGGACAGCAGAGCGTGTCACTG 1600

  contig10_pilo      62214 AGCTTGCCAGAGTCATGTGCTCTTCGGCAGCTGCAGGAGAAGCAGAGCAA 62263
                                                                             
  rnd-4_family-       1601 AGCTTGCCAGAGTCATGTGCTCTTCGGCAGCTGCAGGAGAAGCAGAGCAA 1650

  contig10_pilo      62264 CACTTTGGGTACTTGTCCTGCCCACAGCCCACACTCCTTGACACCCTTGT 62313
                                                                             
  rnd-4_family-       1651 CACTTTGGGTACTTGTCCTGCCCACAGCCCACACTCCTTGACACCCTTGT 1700

  contig10_pilo      62314 CTCGGATATGGCCGAGGTCAAGCGCAACATTGCCTTC 62350
                                                                
  rnd-4_family-       1701 CTCGGATATGGCCGAGGTCAAGCGCAACATTGCCTTC 1737

Matrix = 20p53g.matrix
Kimura (with divCpGMod) = 0.85
Transitions / transversions = 1.50 (12/8)
Gap_init rate = 0.00 (0 / 1736), avg. gap size = 0.0 (0 / 0)

 
 
 
 
 
  +   15066    2.5  0.1  0.0  contig10_pilon    62352   64229 (1750650) +  rnd-3_family-161   LINE/Tad1            1   1879  (2682)    26     
 
 ANNOTATION EVIDENCE: 
 15066   2.45 0.05 0.00  contig10_pilon    62352   64232   1750647 +  rnd-3_family-161   LINE/Tad1            1   1882    2679      
15066 2.45 0.05 0.00 contig10_pilon 62352 64232 (1750647) rnd-3_family-161#LINE/Tad1 1 1882 (2679) m_b496s001i5

  contig10_pilo      62352 TCACTGCCCAACAGGCCTCTGCGCCTTCAAGTGCCCAACAGCAGCAGCCT 62401
                                                                             
  rnd-3_family-          1 TCACTGCCCAACAGGCCTCTGCGCCTTCAAGTGCCCAACAGCAGCAGCCT 50

  contig10_pilo      62402 GCAGCAGTAGCAGCTCCTACAG-CCGCTCCTGCAAAGGCAAGCGCACCTG 62450
                                  i   v     i    - i                    i    
  rnd-3_family-         51 GCAGCAGCAGCTGCTCCCACAGGCTGCTCCTGCAAAGGCAAGCGCGCCTG 100

  contig10_pilo      62451 GCCGGTCCTATGCGTCGCGTGCTGCTCAGCCAGCTGCCAAAGGCACTGTG 62500
                               v                                             
  rnd-3_family-        101 GCCGCTCCTATGCGTCGCGTGCTGCTCAGCCAGCTGCCAAAGGCACTGTG 150

  contig10_pilo      62501 GTGTCTGCCCGCCCCAGTGCGGTGCCAAAGCCACCTCCTACCAATGGTCG 62550
                              i       i                                      
  rnd-3_family-        151 GTGCCTGCCCGTCCCAGTGCGGTGCCAAAGCCACCTCCTACCAATGGTCG 200

  contig10_pilo      62551 TGCGCGCCACCACCCAGGTCGTCTCATCCTGAGCGTCCGTGCCACCCCTG 62600
                                                      i                      
  rnd-3_family-        201 TGCGCGCCACCACCCAGGTCGTCTCATTCTGAGCGTCCGTGCCACCCCTG 250

  contig10_pilo      62601 AGCTCACTGCTCATGTGCGCAACAATGCGAGCCGCTTGCGCGCCGCAGTG 62650
                                                                             
  rnd-3_family-        251 AGCTCACTGCTCATGTGCGCAACAATGCGAGCCGCTTGCGCGCCGCAGTG 300

  contig10_pilo      62651 AACACTGCGCTTGCTCCTCACAAGGGCAAGTCGCCTGCCAAGCTCCCACT 62700
                                                   i                         
  rnd-3_family-        301 AACACTGCGCTTGCTCCTCACAAGAGCAAGTCGCCTGCCAAGCTCCCACT 350

  contig10_pilo      62701 TCGTGTCACAAGTGTGGACACCACCGCCTCTGGCAACATTGCGGTGGTTG 62750
                                                   i                i        
  rnd-3_family-        351 TCGTGTCACAAGTGTGGACACCACTGCCTCTGGCAACATTGTGGTGGTTG 400

  contig10_pilo      62751 CAGCAGATGGACTGACTTCAGCAGATCTGGTTGAACATGGAGATGCAATT 62800
                            i                       i                        
  rnd-3_family-        401 CGGCAGATGGACTGACTTCAGCAGACCTGGTTGAACATGGAGATGCAATT 450

  contig10_pilo      62801 GCGTCTGCCATCGTGCCAGACTCTGGCTCATTCATCAAGGCGTCGCGCGA 62850
                                                                       v     
  rnd-3_family-        451 GCGTCTGCCATCGTGCCAGACTCTGGCTCATTCATCAAGGCGTCTCGCGA 500

  contig10_pilo      62851 CGAACCTTGGCACCAAGTGCTAGTCAATGGTGTCTCGACTCGAGTTCACG 62900
                              i                            i            i  i 
  rnd-3_family-        501 CGAGCCTTGGCACCAAGTGCTAGTCAATGGTGCCTCGACTCGAGTCCATG 550

  contig10_pilo      62901 ACATTGAGGGACTGCCCACTGGGCAAGACCTCCTCTCAGACATTGTTGAG 62950
                                  i                                          
  rnd-3_family-        551 ACATTGAAGGACTGCCCACTGGGCAAGACCTCCTCTCAGACATTGTTGAG 600

  contig10_pilo      62951 TTCAACACTCACAAATTTGACTGGGCCTCCATGCCACGCTGGCTGGGAAG 63000
                                         i            v                      
  rnd-3_family-        601 TTCAACACTCACAAGTTTGACTGGGCCACCATGCCACGCTGGCTGGGAAG 650

  contig10_pilo      63001 TGCAGCAGACCTTGCTGGCAAGAGCTCAGGCTCTGTCATTTTGGCATTCC 63050
                                                                   i         
  rnd-3_family-        651 TGCAGCAGACCTTGCTGGCAAGAGCTCAGGCTCTGTCATTCTGGCATTCC 700

  contig10_pilo      63051 TGAGTGAGGCAGATGCTCGCTATGCAGTTGAGCATGCAGTCAGTGTCTTT 63100
                                        i                    i     i         
  rnd-3_family-        701 TGAGTGAGGCAGACGCTCGCTATGCAGTTGAGCACGCAGTTAGTGTCTTT 750

  contig10_pilo      63101 GGACGCCTCTGCTCAGTTCGCTACTTTGAGGACATGCCTCGTCTGCGCAC 63150
                                                                             
  rnd-3_family-        751 GGACGCCTCTGCTCAGTTCGCTACTTTGAGGACATGCCTCGTCTGCGCAC 800

  contig10_pilo      63151 CTGTGACAGGTGTTGCAGCCTTGACCACACTGCTCGCATTTGCGACAAGC 63200
                              i                                              
  rnd-3_family-        801 CTGCGACAGGTGTTGCAGCCTTGACCACACTGCTCGCATTTGCGACAAGC 850

  contig10_pilo      63201 CCATGCGCTGTGGAGTCTGCAGTGCCAGCACCCACACCACTGCCAACCAC 63250
                                                                             
  rnd-3_family-        851 CCATGCGCTGTGGAGTCTGCAGTGCCAGCACCCACACCACTGCCAACCAC 900

  contig10_pilo      63251 ACTTGCGAGCAGATGGAGTGCGAGTTCTACAGCTCTGTACATGCACCTGG 63300
                                                                i            
  rnd-3_family-        901 ACTTGCGAGCAGATGGAGTGCGAGTTCTACAGCTCTGCACATGCACCTGG 950

  contig10_pilo      63301 AACTCACTGTGAGCACACTCAGCTCAAGTGCCCTCACTGTGGTGGGGCAC 63350
                                    i                                        
  rnd-3_family-        951 AACTCACTGCGAGCACACTCAGCTCAAGTGCCCTCACTGTGGTGGGGCAC 1000

  contig10_pilo      63351 ATGTTGTGCGTGACTCCCAGTGCAAGATCTGGCAGCGGCGTCGCATGGCG 63400
                           i         i                                       
  rnd-3_family-       1001 GTGTTGTGCGCGACTCCCAGTGCAAGATCTGGCAGCGGCGTCGCATGGCG 1050

  contig10_pilo      63401 CTGCGCAAGCCCAGACATGATGCACCCAAGGCTGCAACTGCGCCCAAGCT 63450
                                                                             
  rnd-3_family-       1051 CTGCGCAAGCCCAGACATGATGCACCCAAGGCTGCAACTGCGCCCAAGCT 1100

  contig10_pilo      63451 GCGCCGCACCAAGAGCAGAGTTGCACTTGGCCCCAGGGCCAATGTGTCTG 63500
                                                              i      i       
  rnd-3_family-       1101 GCGCCGCACCAAGAGCAGAGTTGCACTTGGCCCCAAGGCCAACGTGTCTG 1150

  contig10_pilo      63501 GCAGCAATGCAGTCCCTCTGGGGACTAACTCTCGCTCAGAGACAGACGCC 63550
                                     v              i               i        
  rnd-3_family-       1151 GCAGCAATGCCGTCCCTCTGGGGACCAACTCTCGCTCAGAGGCAGACGCC 1200

  contig10_pilo      63551 GCGGCCAAGGCTGCAGCAACTGCAAGCAGCACTCGTGACAAGCCCAGCAC 63600
                                      v                                      
  rnd-3_family-       1201 GCGGCCAAGGCAGCAGCAACTGCAAGCAGCACTCGTGACAAGCCCAGCAC 1250

  contig10_pilo      63601 ACCTGCACCTGACACTGAGTCTGCATGAGAGACACCACTCGCCCCATTCG 63650
                                 i                                           
  rnd-3_family-       1251 ACCTGCGCCTGACACTGAGTCTGCATGAGAGACACCACTCGCCCCATTCG 1300

  contig10_pilo      63651 TGTCTCCATGACCAATGTGCGACGTCTCAATGACTCGCAGTCTGCACTCT 63700
                                                                       i     
  rnd-3_family-       1301 TGTCTCCATGACCAATGTGCGACGTCTCAATGACTCGCAGTCTGTACTCT 1350

  contig10_pilo      63701 TCACTCCTGGCTCCCATTATGGGACCCCTCTTGCATCTGATATTTATCTG 63750
                                                                             
  rnd-3_family-       1351 TCACTCCTGGCTCCCATTATGGGACCCCTCTTGCATCTGATATTTATCTG 1400

  contig10_pilo      63751 GTCACTGAACCGTGGTGGCGCGTTGGTGATGAACCAGACGAGCCACACTA 63800
                                               v                             
  rnd-3_family-       1401 GTCACTGAACCGTGGTGGCGAGTTGGTGATGAACCAGACGAGCCACACTA 1450

  contig10_pilo      63801 CTCTGTCAAGGAACCAGCAGGCATGTATCCCATCCTGCCAGTTCAAGTAG 63850
                                    i                                        
  rnd-3_family-       1451 CTCTGTCAAAGAACCAGCAGGCATGTATCCCATCCTGCCAGTTCAAGTAG 1500

  contig10_pilo      63851 TGCCTGCTCTTGAGAGGCCACGTGTCTTTGCCTATGCGTCTGCGACGCGG 63900
                                                                             
  rnd-3_family-       1501 TGCCTGCTCTTGAGAGGCCACGTGTCTTTGCCTATGCGTCTGCGACGCGG 1550

  contig10_pilo      63901 CGTGACTTTGAAGTATTTAACCGCTATGACCTTGCCCAAGATCTTGACTT 63950
                                                                             
  rnd-3_family-       1551 CGTGACTTTGAAGTATTTAACCGCTATGACCTTGCCCAAGATCTTGACTT 1600

  contig10_pilo      63951 TCTCATCCTTGAGATTCGTCAAGGGAGGCGTCCAGCGTTCTTACTTGTCC 64000
                                 i                                           
  rnd-3_family-       1601 TCTCATTCTTGAGATTCGTCAAGGGAGGCGTCCAGCGTTCTTACTTGTCC 1650

  contig10_pilo      64001 TCATGTACAATGAGGACGCTCCTGAGGGGGCTGAGCGACTTGGCCGCACT 64050
                                                                v            
  rnd-3_family-       1651 TCATGTACAATGAGGACGCTCCTGAGGGGGCTGAGCGCCTTGGCCGCACT 1700

  contig10_pilo      64051 TTTGCGCGCTTCAAGCTCATTGACCTTCCAGACATGCCAATCGCCATTGC 64100
                                                                    i        
  rnd-3_family-       1701 TTTGCGCGCTTCAAGCTCATTGACCTTCCAGACATGCCAATTGCCATTGC 1750

  contig10_pilo      64101 CATGGATGCGAATGAACACCACCATGTGTGGGATTTGTTTGCGGCCAACT 64150
                                    i                       i                
  rnd-3_family-       1751 CATGGATGCAAATGAACACCACCATGTGTGGGACTTGTTTGCGGCCAACT 1800

  contig10_pilo      64151 CCTCTCGAGGGGGTGAGCAGATGCTGGAGTGGATGGAGGAGCATGGGTTC 64200
                                                                             
  rnd-3_family-       1801 CCTCTCGAGGGGGTGAGCAGATGCTGGAGTGGATGGAGGAGCATGGGTTC 1850

  contig10_pilo      64201 TCTGTGCTCAACAGTCCTGATGTGGCCACTTG 64232
                                                           
  rnd-3_family-       1851 TCTGTGCTCAACAGTCCTGATGTGGCCACTTG 1882

Matrix = 20p53g.matrix
Kimura (with divCpGMod) = 2.00
Transitions / transversions = 4.75 (38/8)
Gap_init rate = 0.00 (1 / 1880), avg. gap size = 1.00 (1 / 1)

 
 

 
   +   30705    0.9  0.1  0.1  contig10_pilon    64230   68451 (1746428) C  rnd-4_family-763   LTR/Gypsy          (0)   3908       1    27     
 
 ANNOTATION EVIDENCE: 
  2736   2.17 0.00 0.00  contig10_pilon    64230   64598   1750281 C  rnd-4_family-763   LTR/Gypsy            1    369    3539      
2736 2.17 0.00 0.00 contig10_pilon 64230 64598 (1750281) C rnd-4_family-763#LTR/Gypsy (3539) 369 1 m_b496s001i6

  contig10_pilo      64230 TTGTGTTGCGGTCTTCGTCGGGCCCCTCCTCCCCCGCTGTTTCATTTCGC 64279
                             i                   i                           
C rnd-4_family-        369 TTATGTTGCGGTCTTCGTCGGGTCCCTCCTCCCCCGCTGTTTCATTTCGC 320

  contig10_pilo      64280 CGTCCCGTGGCCCTCGCACCTCGTCCGGGCGCCCAAACTCCTTGCGCCCG 64329
                                ?           i    ?i                          
C rnd-4_family-        319 CGTCCNGTGGCCCTCGCGCCTCNCCCGGGCGCCCAAACTCCTTGCGCCCG 270

  contig10_pilo      64330 GCCCCGCGGCCCTTTCGTCGGCGCGTCCAAGCGGTCTCTCCGCTCGGCGA 64379
                                       i                                    i
C rnd-4_family-        269 GCCCCGCGGCCCCTTCGTCGGCGCGTCCAAGCGGTCTCTCCGCTCGGCGG 220

  contig10_pilo      64380 GCACCTCGGTCTCTTCCCAGGAGAATGGTAATATGACGGGATGTTTCTGG 64429
                                                    v                        
C rnd-4_family-        219 GCACCTCGGTCTCTTCCCAGGAGAAGGGTAATATGACGGGATGTTTCTGG 170

  contig10_pilo      64430 TTTGGTTGGGCTCTATTTATCCCGGGTTTCCTGTCTCGCCGACGACGCCC 64479
                                                                             
C rnd-4_family-        169 TTTGGTTGGGCTCTATTTATCCCGGGTTTCCTGTCTCGCCGACGACGCCC 120

  contig10_pilo      64480 CGGCGGTCTCTTTCGGCCTGATCTGTGGCGCCCGCGTGACAACCGGTCGG 64529
                                                                             
C rnd-4_family-        119 CGGCGGTCTCTTTCGGCCTGATCTGTGGCGCCCGCGTGACAACCGGTCGG 70

  contig10_pilo      64530 TGGCGCAATTGGTCCGCGGGGCCCCTCGGCCTTGCTTCCGTAGTATATAT 64579
                                                                             
C rnd-4_family-         69 TGGCGCAATTGGTCCGCGGGGCCCCTCGGCCTTGCTTCCGTAGTATATAT 20

  contig10_pilo      64580 ATATGGGCCGTCGCCCCTC 64598
                             v                
C rnd-4_family-         19 ATCTGGGCCGTCGCCCCTC 1

Matrix = 20p53g.matrix
Kimura (with divCpGMod) = 1.96
Transitions / transversions = 3.00 (6/2)
Gap_init rate = 0.00 (0 / 368), avg. gap size = 0.0 (0 / 0)

 30705   0.89 0.05 0.15  contig10_pilon    64426   68337   1746542 C  rnd-4_family-763   LTR/Gypsy            1   3908       0      
30705 0.89 0.05 0.15 contig10_pilon 64426 68337 (1746542) C rnd-4_family-763#LTR/Gypsy (0) 3908 1 m_b496s001i7

  contig10_pilo      64426 CTGGTTTGGTTGGGCTCTATTTATCCCGGGTTTCCTGTCTCGCCGACGAC 64475
                            i                                                
C rnd-4_family-       3908 CCGGTTTGGTTGGGCTCTATTTATCCCGGGTTTCCTGTCTCGCCGACGAC 3859

  contig10_pilo      64476 GCCCCGGCGGTCTCTTTCGGCCTGATCTGTGGCGCCCGCGTGACAACCGG 64525
                                                                             
C rnd-4_family-       3858 GCCCCGGCGGTCTCTTTCGGCCTGATCTGTGGCGCCCGCGTGACAACCGG 3809

  contig10_pilo      64526 TCGGTGGCGCAATTGGTCCGCGGGGCCCCTCGGCCTTGCTTCCGTAGTAT 64575
                                                                             
C rnd-4_family-       3808 TCGGTGGCGCAATTGGTCCGCGGGGCCCCTCGGCCTTGCTTCCGTAGTAT 3759

  contig10_pilo      64576 ATATATATGGGCCGTCGCCCCTCTTGTACCTTAGGTTTGGTCTGGTCTAT 64625
                                 v                                           
C rnd-4_family-       3758 ATATATCTGGGCCGTCGCCCCTCTTGTACCTTAGGTTTGGTCTGGTCTAT 3709

  contig10_pilo      64626 CATACCGCTTATCCGCGCCTCCCTCTCCCCACTCATCCCTTCCCCTCGGG 64675
                                                                             
C rnd-4_family-       3708 CATACCGCTTATCCGCGCCTCCCTCTCCCCACTCATCCCTTCCCCTCGGG 3659

  contig10_pilo      64676 CGTCGTCTCGGAGAGTGATATAGCAGTCACCGTGACATAAGACCAAACCT 64725
                                                                             
C rnd-4_family-       3658 CGTCGTCTCGGAGAGTGATATAGCAGTCACCGTGACATAAGACCAAACCT 3609

  contig10_pilo      64726 ACCCTCCCTCCCGACGCTCGAGATCCCCATACCCCTTACTCACCACCCTT 64775
                                                                             
C rnd-4_family-       3608 ACCCTCCCTCCCGACGCTCGAGATCCCCATACCCCTTACTCACCACCCTT 3559

  contig10_pilo      64776 CCAATCCGGGCGAACCGTTCGGTCGGCGTTGCTCGGTCCAGGAAGGAATC 64825
                                                                             
C rnd-4_family-       3558 CCAATCCGGGCGAACCGTTCGGTCGGCGTTGCTCGGTCCAGGAAGGAATC 3509

  contig10_pilo      64826 CGACCCCTCGGACCGGCTAGGAGATCCCTCGCGTCGACGAGGACCCGTCG 64875
                                                   i                         
C rnd-4_family-       3508 CGACCCCTCGGACCGGCTAGGAGACCCCTCGCGTCGACGAGGACCCGTCG 3459

  contig10_pilo      64876 CGACCGCCGCGCCCACTCATCGGCCCACCAGGACCCTGCAGGACCCACCC 64925
                                                                             
C rnd-4_family-       3458 CGACCGCCGCGCCCACTCATCGGCCCACCAGGACCCTGCAGGACCCACCC 3409

  contig10_pilo      64926 TCCGGACGCCGAGCCTTGCCCACTGGCCTTCGGGTCAGGCACTCAGCCAA 64975
                                                          v                  
C rnd-4_family-       3408 TCCGGACGCCGAGCCTTGCCCACTGGCCTTCTGGTCAGGCACTCAGCCAA 3359

  contig10_pilo      64976 CGAGGACCCCTCCAGACCCATACGGTCGTCAAATCCGAAAGCTCATTCCA 65025
                                                                             
C rnd-4_family-       3358 CGAGGACCCCTCCAGACCCATACGGTCGTCAAATCCGAAAGCTCATTCCA 3309

  contig10_pilo      65026 TCACCGGCTCCTGATCTCTCCTCTTCATCAGAGCCAGGTCATCCGCGGCG 65075
                                                              v              
C rnd-4_family-       3308 TCACCGGCTCCTGATCTCTCCTCTTCATCAGAGCCCGGTCATCCGCGGCG 3259

  contig10_pilo      65076 AGACCACCCCCCC-ACAAGCCCTACTCGCCGAACGCGCCCGCTGCGCGTC 65124
                                        -                                    
C rnd-4_family-       3258 AGACCACCCCCCCCACAAGCCCTACTCGCCGAACGCGCCCGCTGCGCGTC 3209

  contig10_pilo      65125 CCCACCCCTCGGTCAACCTCTAAACCTCCCACGGCCGACCCCTCTCTCCA 65174
                                                                             
C rnd-4_family-       3208 CCCACCCCTCGGTCAACCTCTAAACCTCCCACGGCCGACCCCTCTCTCCA 3159

  contig10_pilo      65175 GAACCTCACCCTCCGCGCATCCCGAGAGCAAGTTCCCCCCCCCCCCCCCC 65224
                                                                       ------
C rnd-4_family-       3158 GAACCTCACCCTCCGCGCATCCCGAGAGCAAGTTCCCCCCCCCC------ 3115

  contig10_pilo      65225 GTGGTGCGCGCGGCGCATTCACATCATCTTCCTCGGATCGTGACCCCCAT 65274
                                                                             
C rnd-4_family-       3114 GTGGTGCGCGCGGCGCATTCACATCATCTTCCTCGGATCGTGACCCCCAT 3065

  contig10_pilo      65275 CATCCCCACGCATCAACGCTTACGACACATACACCATATAATCCGGATCG 65324
                                                                             
C rnd-4_family-       3064 CATCCCCACGCATCAACGCTTACGACACATACACCATATAATCCGGATCG 3015

  contig10_pilo      65325 GCCGCCAGACCGACGCCTCTTTCCCCCCTTTTCCCCGACACGCCCCTTTG 65374
                                                                             
C rnd-4_family-       3014 GCCGCCAGACCGACGCCTCTTTCCCCCCTTTTCCCCGACACGCCCCTTTG 2965

  contig10_pilo      65375 TTCGGACCGAACGCTCACAAAATGTCCCAGTACGACGAGACCATAGTTAA 65424
                                                                             
C rnd-4_family-       2964 TTCGGACCGAACGCTCACAAAATGTCCCAGTACGACGAGACCATAGTTAA 2915

  contig10_pilo      65425 CCCTCAATACATACCGGCGGCAGTCCTGGACCACCGCGTACACCGAGGAG 65474
                                                                             
C rnd-4_family-       2914 CCCTCAATACATACCGGCGGCAGTCCTGGACCACCGCGTACACCGAGGAG 2865

  contig10_pilo      65475 TTCCTCAGTATCTCGTCCAATGGATGGGCTTTCCCCCCGAGGACGCTAGC 65524
                                                                             
C rnd-4_family-       2864 TTCCTCAGTATCTCGTCCAATGGATGGGCTTTCCCCCCGAGGACGCTAGC 2815

  contig10_pilo      65525 TGGCAACTGGCCGCCGACCTGGACGGAACCGGACTCGTGGAGGAGTACCA 65574
                                                                             
C rnd-4_family-       2814 TGGCAACTGGCCGCCGACCTGGACGGAACCGGACTCGTGGAGGAGTACCA 2765

  contig10_pilo      65575 CGCCGAACCACCGGAGCCAAATGAGGACCAAGGCGCCGCACAGGACCTGG 65624
                                                                             
C rnd-4_family-       2764 CGCCGAACCACCGGAGCCAAATGAGGACCAAGGCGCCGCACAGGACCTGG 2715

  contig10_pilo      65625 ACCAGGATGCGGACATGGACCTCGGCGAGGACTTTGGCGACGGATCCCAA 65674
                                                                             
C rnd-4_family-       2714 ACCAGGATGCGGACATGGACCTCGGCGAGGACTTTGGCGACGGATCCCAA 2665

  contig10_pilo      65675 GAGCTGTTCGGCGAATCACCCATCAGCACCGGCGAGTCACCTCTCGCCGC 65724
                                                                             
C rnd-4_family-       2664 GAGCTGTTCGGCGAATCACCCATCAGCACCGGCGAGTCACCTCTCGCCGC 2615

  contig10_pilo      65725 CGACGAGTCGTCCGAGCTTGAGGACGCGCGCCCGGCCACACCTCCGCTGG 65774
                                     i                                       
C rnd-4_family-       2614 CGACGAGTCGCCCGAGCTTGAGGACGCGCGCCCGGCCACACCTCCGCTGG 2565

  contig10_pilo      65775 GGCTCATCCCCCTTCGCGCTCGTATCCGGTCGCCGGACCCGGACGACACC 65824
                                                                             
C rnd-4_family-       2564 GGCTCATCCCCCTTCGCGCTCGTATCCGGTCGCCGGACCCGGACGACACC 2515

  contig10_pilo      65825 CGGCTCGACATCGCCATGCTCGTGGCCCCTCCCCAACCTTTCTTCTGGAT 65874
                                                                 v           
C rnd-4_family-       2514 CGGCTCGACATCGCCATGCTCGTGGCCCCTCCCCAACCGTTCTTCTGGAT 2465

  contig10_pilo      65875 GCTAGACCGCTTCACTCACTACGGCCGCTTCACTGCCCCGAGACATCTCC 65924
                                                          i                  
C rnd-4_family-       2464 GCTAGACCGCTTCACTCACTACGGCCGCTTCGCTGCCCCGAGACATCTCC 2415

  contig10_pilo      65925 CGCTCGATATCTCGCGTTACTGGGTACTCCCGACCGTCGTGCGCAATGAC 65974
                                                                             
C rnd-4_family-       2414 CGCTCGATATCTCGCGTTACTGGGTACTCCCGACCGTCGTGCGCAATGAC 2365

  contig10_pilo      65975 CGAGGCCGACGGGCTCGCACCGAGCTAGTGGTCTTCTCGTTCCCACCTCG 66024
                                                                             
C rnd-4_family-       2364 CGAGGCCGACGGGCTCGCACCGAGCTAGTGGTCTTCTCGTTCCCACCTCG 2315

  contig10_pilo      66025 CCTCGGCCCTCATCTCATCACGTCGCCTACGACGGAACGAGCCCTCCAGG 66074
                                           v                                 
C rnd-4_family-       2314 CCTCGGCCCTCATCTCCTCACGTCGCCTACGACGGAACGAGCCCTCCAGG 2265

  contig10_pilo      66075 TCGTTCGGCTTACGGCGTGCCTTGTCTGGGACGTCCGCGAATATCGTGCC 66124
                                                                             
C rnd-4_family-       2264 TCGTTCGGCTTACGGCGTGCCTTGTCTGGGACGTCCGCGAATATCGTGCC 2215

  contig10_pilo      66125 ACGTACGCCGACGACGCCCTGGTCCTGCGGATCGCGTCCCCCGGCCCGGC 66174
                                                                             
C rnd-4_family-       2214 ACGTACGCCGACGACGCCCTGGTCCTGCGGATCGCGTCCCCCGGCCCGGC 2165

  contig10_pilo      66175 CCACCGTCTTTGAACGCTCCGGACGGTTGCCCCTGCCTTGTACAGCAGCA 66224
                                                      i                      
C rnd-4_family-       2164 CCACCGTCTTTGAACGCTCCGGACGGTCGCCCCTGCCTTGTACAGCAGCA 2115

  contig10_pilo      66225 CGCTTTGCGAAGCTGAAACCGCCCTTCTTTGTCGATATCTACTGGCGTAC 66274
                                                                             
C rnd-4_family-       2114 CGCTTTGCGAAGCTGAAACCGCCCTTCTTTGTCGATATCTACTGGCGTAC 2065

  contig10_pilo      66275 AGGTGACGCCACCCGTCGTTCCCTCCCCCTCGGCGACCGTGCCCAGAATC 66324
                                                                             
C rnd-4_family-       2064 AGGTGACGCCACCCGTCGTTCCCTCCCCCTCGGCGACCGTGCCCAGAATC 2015

  contig10_pilo      66325 GGACCCTACCTCGGTCGGACGCCGCTTCGGTCGGATGCTCTCCACAGTCA 66374
                                                                             
C rnd-4_family-       2014 GGACCCTACCTCGGTCGGACGCCGCTTCGGTCGGATGCTCTCCACAGTCA 1965

  contig10_pilo      66375 CGGTCCCGGTCGTTTCTCATACCCCGTATTTATGCTCGTCTTTCCGCCTC 66424
                                                                             
C rnd-4_family-       1964 CGGTCCCGGTCGTTTCTCATACCCCGTATTTATGCTCGTCTTTCCGCCTC 1915

  contig10_pilo      66425 TCTATTTACATATTTTACGAGCCTCCACACCACTACCCGAATCATACCCG 66474
                                                                 i    i      
C rnd-4_family-       1914 TCTATTTACATATTTTACGAGCCTCCACACCACTACCCAAATCGTACCCG 1865

  contig10_pilo      66475 GACACGGTCTTACTCCTCCATGGGTTGGCCCGCATCGACCGACTTCTCCC 66524
                                                                             
C rnd-4_family-       1864 GACACGGTCTTACTCCTCCATGGGTTGGCCCGCATCGACCGACTTCTCCC 1815

  contig10_pilo      66525 CTATCACGGACGCATCCCGACCCTCGGACCGTTGGACGCGTGGCAGGAAT 66574
                                                                             
C rnd-4_family-       1814 CTATCACGGACGCATCCCGACCCTCGGACCGTTGGACGCGTGGCAGGAAT 1765

  contig10_pilo      66575 CCGGACCCGGACCCAGACGCCTCCAGCGACGCAAAGCTGGGTATCGGACG 66624
                                                                     i       
C rnd-4_family-       1764 CCGGACCCGGACCCAGACGCCTCCAGCGACGCAAAGCTGGGTGTCGGACG 1715

  contig10_pilo      66625 CACATCCCCCGGCACTACCACGCCAAGAAGGGCCAGGTCGGCGCCGAAGT 66674
                                                                             
C rnd-4_family-       1714 CACATCCCCCGGCACTACCACGCCAAGAAGGGCCAGGTCGGCGCCGAAGT 1665

  contig10_pilo      66675 CAGCAAGCACGCCGACAGGACCCGCCGGCGCCTCCGCCTCCACGCCCGCG 66724
                                                 v                           
C rnd-4_family-       1664 CAGCAAGCACGCCGACAGGACCAGCCGGCGCCTCCGCCTCCACGCCCGCG 1615

  contig10_pilo      66725 GCGCCAGGGGAAGTCGGCGCGTCGGTGGGCGACCTCCTCGCCTCGTCCTC 66774
                                                                i            
C rnd-4_family-       1614 GCGCCAGGGGAAGTCGGCGCGTCGGTGGGCGACCTCCCCGCCTCGTCCTC 1565

  contig10_pilo      66775 CACCGCTCGGCCGTCATTGGAGTCGGTATCTCGCTCGCTCCCGCCCGGCG 66824
                                                         v                   
C rnd-4_family-       1564 CACCGCTCGGCCGTCATTGGAGTCGGTATCGCGCTCGCTCCCGCCCGGCG 1515

  contig10_pilo      66825 ACACGAGGCGCACCGTCTTATGGGCACGCCGGTCATCGACGAGCTCTTGC 66874
                                                                             
C rnd-4_family-       1514 ACACGAGGCGCACCGTCTTATGGGCACGCCGGTCATCGACGAGCTCTTGC 1465

  contig10_pilo      66875 GTCCACTTGCGAAGGTCGCGCCTCAACGCCGAGACGGACAGCTGGAACCC 66924
                                                                             
C rnd-4_family-       1464 GTCCACTTGCGAAGGTCGCGCCTCAACGCCGAGACGGACAGCTGGAACCC 1415

  contig10_pilo      66925 CTGGACGATGCGGTCGCCCAGGACCTCGAGGTCGGCCATGGTGGCTGTCC 66974
                                                                             
C rnd-4_family-       1414 CTGGACGATGCGGTCGCCCAGGACCTCGAGGTCGGCCATGGTGGCTGTCC 1365

  contig10_pilo      66975 CCGGCGACCGAAGCCTTCGGCGTGTGCGCGACCGCGCCACGGACGCGCCG 67024
                                                                             
C rnd-4_family-       1364 CCGGCGACCGAAGCCTTCGGCGTGTGCGCGACCGCGCCACGGACGCGCCG 1315

  contig10_pilo      67025 ACGGTGTCCACCACGTCGTCCTGGTCCGGATCGTCCCCGTCATCGCCGTC 67074
                                                                    i        
C rnd-4_family-       1314 ACGGTGTCCACCACGTCGTCCTGGTCCGGATCGTCCCCGTCGTCGCCGTC 1265

  contig10_pilo      67075 CACGTCAGCGTCCTGTCGCACCCTCTTGCGCGATGTGCCCGACCGGCCGA 67124
                                              i                              
C rnd-4_family-       1264 CACGTCAGCGTCCTGTCGCGCCCTCTTGCGCGATGTGCCCGACCGGCCGA 1215

  contig10_pilo      67125 CGCTGTGAGAACACCCCACGCGGGTCCGGTGGCACCGAGCACAGGCAGTC 67174
                                                             v               
C rnd-4_family-       1214 CGCTGTGAGAACACCCCACGCGGGTCCGGTGGCAACGAGCACAGGCAGTC 1165

  contig10_pilo      67175 GCCGACCCGGACACTTGTCTAAAACACGGCCGGCCGGAGCGCTCGCACTG 67224
                                              v                              
C rnd-4_family-       1164 GCCGACCCGGACACTTGTCGAAAACACGGCCGGCCGGAGCGCTCGCACTG 1115

  contig10_pilo      67225 CTCACAAGGCTGGACCCGGACGAAGGATGTCAGCGCAGGATTCACCCCGG 67274
                                                                    v        
C rnd-4_family-       1114 CTCACAAGGCTGGACCCGGACGAAGGATGTCAGCGCAGGATACACCCCGG 1065

  contig10_pilo      67275 AGCAAGAGGACCTTTGACCAAGACGTACCGTGTTAAAAGGAGGCCCGACC 67324
                                                                             
C rnd-4_family-       1064 AGCAAGAGGACCTTTGACCAAGACGTACCGTGTTAAAAGGAGGCCCGACC 1015

  contig10_pilo      67325 GCGTCCTTCTCCTCGTCGCCGGTCCCTCGCGCGCCGACGTCCGAAGGCCG 67374
                                                                             
C rnd-4_family-       1014 GCGTCCTTCTCCTCGTCGCCGGTCCCTCGCGCGCCGACGTCCGAAGGCCG 965

  contig10_pilo      67375 CTTCGACCCAGACGCGACCACTCCCGATGAGCCCTTGCCCTTCGTGGTCG 67424
                                              i         i              i     
C rnd-4_family-        964 CTTCGACCCAGACGCGACCGCTCCCGATGGGCCCTTGCCCTTCGCGGTCG 915

  contig10_pilo      67425 AGGAGGTCGTTCCCGTGCGGCGAACCCCTTCCACCTTCGGCACTACGGGG 67474
                                                                             
C rnd-4_family-        914 AGGAGGTCGTTCCCGTGCGGCGAACCCCTTCCACCTTCGGCACTACGGGG 865

  contig10_pilo      67475 AGCGAGGCTGCTGTCATGCCGAGGGGAGACGCCGCCGTCTCCAGATTGCG 67524
                                                   i                         
C rnd-4_family-        864 AGCGAGGCTGCTGTCATGCCGAGGAGAGACGCCGCCGTCTCCAGATTGCG 815

  contig10_pilo      67525 GGTCGCGGCGTTGCGTCGGCGCACCTCGGCGTCAATGGCCTCCAACCGCT 67574
                                                                             
C rnd-4_family-        814 GGTCGCGGCGTTGCGTCGGCGCACCTCGGCGTCAATGGCCTCCAACCGCT 765

  contig10_pilo      67575 GGCCCGCAGACGCAAGCACCGTCCGCGCCTTCTCCTCCTCCGCGTCCGCG 67624
                                                                             
C rnd-4_family-        764 GGCCCGCAGACGCAAGCACCGTCCGCGCCTTCTCCTCCTCCGCGTCCGCG 715

  contig10_pilo      67625 GCGTAGTGCCCGAAGTCCAGTTGGCGCTGGATGGAGGCATGCAGCGTGAA 67674
                                                                             
C rnd-4_family-        714 GCGTAGTGCCCGAAGTCCAGTTGGCGCTGGATGGAGGCATGCAGCGTGAA 665

  contig10_pilo      67675 CACCCGCTCCCTGAGGTCCCGAAGTTCGCCGAGAGGCTTCGCAGTCAGTC 67724
                                                                             
C rnd-4_family-        664 CACCCGCTCCCTGAGGTCCCGAAGTTCGCCGAGAGGCTTCGCAGTCAGTC 615

  contig10_pilo      67725 CGTCGGCGGCGGTGGGCGTCATAGGATATCGGACGGTGACGGGAAGCGGG 67774
                                                                             
C rnd-4_family-        614 CGTCGGCGGCGGTGGGCGTCATAGGATATCGGACGGTGACGGGAAGCGGG 565

  contig10_pilo      67775 GAGGCGAAGAGCGAGAAGTACCGCGGTTCGTGTACGGCGGCAGCCGAGCC 67824
                                                                    i        
C rnd-4_family-        564 GAGGCGAAGAGCGAGAAGTACCGCGGTTCGTGTACGGCGGCGGCCGAGCC 515

  contig10_pilo      67825 TTTCGCGGCATCTGACGCTTAGAGTTTGATCCCGCGACGGATGGAGCTCG 67874
                                                               i             
C rnd-4_family-        514 TTTCGCGGCATCTGACGCTTAGAGTTTGATCCCGCGGCGGATGGAGCTCG 465

  contig10_pilo      67875 GCCCGGCCACGCTATGGTCCGTCTCCCCACGCCCGATCTAACCCGCACCT 67924
                                                                             
C rnd-4_family-        464 GCCCGGCCACGCTATGGTCCGTCTCCCCACGCCCGATCTAACCCGCACCT 415

  contig10_pilo      67925 CCCCTGGCCATT-CCCCCTCGGCCACTGGACGGCTAGAAGGGGGGTTATG 67973
                                       -                                     
C rnd-4_family-        414 CCCCTGGCCATTCCCCCCTCGGCCACTGGACGGCTAGAAGGGGGGTTATG 365

  contig10_pilo      67974 TTGCGGTCTTCGTCGGGCCCCTCCTCCCCCGCTGTTTCATTTCACCGTCC 68023
                                            i                         i      
C rnd-4_family-        364 TTGCGGTCTTCGTCGGGTCCCTCCTCCCCCGCTGTTTCATTTCGCCGTCC 315

  contig10_pilo      68024 CGTGGCCCTCGCACCTCGTCCGGGCGCCCAAACTCCTTGCGCCCGGCCCC 68073
                           ?           i    ?i                               
C rnd-4_family-        314 NGTGGCCCTCGCGCCTCNCCCGGGCGCCCAAACTCCTTGCGCCCGGCCCC 265

  contig10_pilo      68074 GCGGCCCTTTCGTCGGCGCGTCCAAGCGGTCTCTCCGCTCGGCGAGCACC 68123
                                  i                                    i     
C rnd-4_family-        264 GCGGCCCCTTCGTCGGCGCGTCCAAGCGGTCTCTCCGCTCGGCGGGCACC 215

  contig10_pilo      68124 TCGGTCTCTTCCCAGGAGAATGGTAATATGACGGGATGTTTCTGGTTTGG 68173
                                               v                             
C rnd-4_family-        214 TCGGTCTCTTCCCAGGAGAAGGGTAATATGACGGGATGTTTCTGGTTTGG 165

  contig10_pilo      68174 TTGGGCTCTATTTATCCCGGGTTTCCTGTCTCGCCGACGACGCCCCGGCG 68223
                                                                             
C rnd-4_family-        164 TTGGGCTCTATTTATCCCGGGTTTCCTGTCTCGCCGACGACGCCCCGGCG 115

  contig10_pilo      68224 GTCTCTTTCGGCCTGATCTGTGGCGCCCGCGTGACAACCGGTCGGTGGCG 68273
                                                                             
C rnd-4_family-        114 GTCTCTTTCGGCCTGATCTGTGGCGCCCGCGTGACAACCGGTCGGTGGCG 65

  contig10_pilo      68274 CAATTGGTCCGCGGGGCCCCTCGGCCTTGCTTCCGTAGTATATATATATG 68323
                                                                          v  
C rnd-4_family-         64 CAATTGGTCCGCGGGGCCCCTCGGCCTTGCTTCCGTAGTATATATATCTG 15

  contig10_pilo      68324 GGCCGTCGCCCCTC 68337
                                         
C rnd-4_family-         14 GGCCGTCGCCCCTC 1

Matrix = 20p53g.matrix
Kimura (with divCpGMod) = 0.65
Transitions / transversions = 1.92 (23/12)
Gap_init rate = 0.00 (8 / 3911), avg. gap size = 1.00 (8 / 8)

  2265   0.70 0.00 0.00  contig10_pilon    68165   68451   1746428 C  rnd-4_family-763   LTR/Gypsy         3622   3908       0      
2265 0.70 0.00 0.00 contig10_pilon 68165 68451 (1746428) C rnd-4_family-763#LTR/Gypsy (0) 3908 3622 m_b496s001i8

  contig10_pilo      68165 CTGGTTTGGTTGGGCTCTATTTATCCCGGGTTTCCTGTCTCGCCGACGAC 68214
                            i                                                
C rnd-4_family-       3908 CCGGTTTGGTTGGGCTCTATTTATCCCGGGTTTCCTGTCTCGCCGACGAC 3859

  contig10_pilo      68215 GCCCCGGCGGTCTCTTTCGGCCTGATCTGTGGCGCCCGCGTGACAACCGG 68264
                                                                             
C rnd-4_family-       3858 GCCCCGGCGGTCTCTTTCGGCCTGATCTGTGGCGCCCGCGTGACAACCGG 3809

  contig10_pilo      68265 TCGGTGGCGCAATTGGTCCGCGGGGCCCCTCGGCCTTGCTTCCGTAGTAT 68314
                                                                             
C rnd-4_family-       3808 TCGGTGGCGCAATTGGTCCGCGGGGCCCCTCGGCCTTGCTTCCGTAGTAT 3759

  contig10_pilo      68315 ATATATATGGGCCGTCGCCCCTCTTGTACCTTAGGTTTGGTCTGGTCTAT 68364
                                 v                                           
C rnd-4_family-       3758 ATATATCTGGGCCGTCGCCCCTCTTGTACCTTAGGTTTGGTCTGGTCTAT 3709

  contig10_pilo      68365 CATACCGCTTATCCGCGCCTCCCTCTCCCCACTCATCCCTTCCCCTCGGG 68414
                                                                             
C rnd-4_family-       3708 CATACCGCTTATCCGCGCCTCCCTCTCCCCACTCATCCCTTCCCCTCGGG 3659

  contig10_pilo      68415 CGTCGTCTCGGAGAGTGATATAGCAGTCACCGTGACA 68451
                                                                
C rnd-4_family-       3658 CGTCGTCTCGGAGAGTGATATAGCAGTCACCGTGACA 3622

Matrix = 20p53g.matrix
Kimura (with divCpGMod) = 0.38
Transitions / transversions = 1.00 (1/1)
Gap_init rate = 0.00 (0 / 286), avg. gap size = 0.0 (0 / 0)

 
 
 
 
 
  +   11141    2.2  0.0  0.1  contig10_pilon    68452   69841 (1745038) C  rnd-3_family-161   LINE/Tad1       (1296)   3265    1878    28     
 
 ANNOTATION EVIDENCE: 
 11141   2.22 0.00 0.14  contig10_pilon    68452   69847   1745032 C  rnd-3_family-161   LINE/Tad1         1872   3265    1296      
11141 2.22 0.00 0.14 contig10_pilon 68452 69847 (1745032) C rnd-3_family-161#LINE/Tad1 (1296) 3265 1872 m_b496s001i9

  contig10_pilo      68452 AGATGGATGCCTCTCTGTCCTGCAAGAAGGATGCAACCCACTGCACAATG 68501
                                                                             
C rnd-3_family-       3265 AGATGGATGCCTCTCTGTCCTGCAAGAAGGATGCAACCCACTGCACAATG 3216

  contig10_pilo      68502 GGCAGAGGCAGACGCTTCTCTCGCAGGACCAGCAGGAGACGCTGGTGGTT 68551
                                     v                        v  i           
C rnd-3_family-       3215 GGCAGAGGCACACGCTTCTCTCGCAGGACCAGCAGCAGGCGCTGGTGGTT 3166

  contig10_pilo      68552 GACAAAGTCAAAGTAGCCCTTGATGTCAAAGGTCAGGGAACTAGTCACCA 68601
                                    i                          v  i          
C rnd-3_family-       3165 GACAAAGTCGAAGTAGCCCTTGATGTCAAAGGTCAGTGAGCTAGTCACCA 3116

  contig10_pilo      68602 AACCCTTGTCACGAGCAGTCTGCACATCATGAGTGTAGCACAGAGCAGCA 68651
                                                                             
C rnd-3_family-       3115 AACCCTTGTCACGAGCAGTCTGCACATCATGAGTGTAGCACAGAGCAGCA 3066

  contig10_pilo      68652 TCCACAGTGGAGGACGCAGGCATGGCCCCAAACTGGTTGGGGTGCACAAG 68701
                                                                             
C rnd-3_family-       3065 TCCACAGTGGAGGACGCAGGCATGGCCCCAAACTGGTTGGGGTGCACAAG 3016

  contig10_pilo      68702 TCCCATGGCAGATGCCATGTAGGTCAAGCGCTTTGCCTGAATGCGCTCCA 68751
                           v                                                 
C rnd-3_family-       3015 ACCCATGGCAGATGCCATGTAGGTCAAGCGCTTTGCCTGAATGCGCTCCA 2966

  contig10_pilo      68752 AGACTTTTCCCAAGCAGACAAGCAAGGTAATCAGCCTGTAAGACCTGGGA 68801
                                                                         v   
C rnd-3_family-       2965 AGACTTTTCCCAAGCAGACAAGCAAGGTAATCAGCCTGTAAGACCTTGGA 2916

  contig10_pilo      68802 TTAGACCAATCAGTCTTGCCAGCTTTCTTGAGCACATATGCAATGGCTCG 68851
                                                                             
C rnd-3_family-       2915 TTAGACCAATCAGTCTTGCCAGCTTTCTTGAGCACATATGCAATGGCTCG 2866

  contig10_pilo      68852 TCTCCAGCGGCGAGGGTGGTAGCCCCAAAGAGCACAGCGCGCAATGAGAA 68901
                                                                             
C rnd-3_family-       2865 TCTCCAGCGGCGAGGGTGGTAGCCCCAAAGAGCACAGCGCGCAATGAGAA 2816

  contig10_pilo      68902 GATAGATCTCCTCCTGTGCCTCATCCCAAGCCCAGCGCAACCCAGTGTAG 68951
                                               v                             
C rnd-3_family-       2815 GATAGATCTCCTCCTGTGCCACATCCCAAGCCCAGCGCAACCCAGTGTAG 2766

  contig10_pilo      68952 GGAACTCCCTCATCACCAGGCGCTTTCTGGGGGTTAGGCCCAAAGATGCC 69001
                                                                             
C rnd-3_family-       2765 GGAACTCCCTCATCACCAGGCGCTTTCTGGGGGTTAGGCCCAAAGATGCC 2716

  contig10_pilo      69002 TTCTCTCACCTCCTCCAGAGTGACCTCCCTGTAGGGAATGTCTTCTGGGT 69051
                                                                             
C rnd-3_family-       2715 TTCTCTCACCTCCTCCAGAGTGACCTCCCTGTAGGGAATGTCTTCTGGGT 2666

  contig10_pilo      69052 GTGGTGTGGTCAGGTCTGGCTCGTCCAGGCCCTCTATGTGAGGAGGGCGC 69101
                                                v                            
C rnd-3_family-       2665 GTGGTGTGGTCAGGTCTGGCTGGTCCAGGCCCTCTATGTGAGGAGGGCGC 2616

  contig10_pilo      69102 TGGTACAAGGTCTCGCGCAGTGCATCACACTTTTCAGCATGAGTGCGCGC 69151
                                                                             
C rnd-3_family-       2615 TGGTACAAGGTCTCGCGCAGTGCATCACACTTTTCAGCATGAGTGCGCGC 2566

  contig10_pilo      69152 AAAGGTGCCATCTGGGCGACGAATGTCAGGAGAACGGTACCCCCGACGTC 69201
                                   v               i                        -
C rnd-3_family-       2565 AAAGGTGCAATCTGGGCGACGAATATCAGGAGAACGGTACCCCCGACGT- 2517

  contig10_pilo      69202 CCTGCGACCACTTGCGCATGTCCCAGATGTCATCCACCGTGGCATCCTCC 69251
                           -   v                                v            
C rnd-3_family-       2516 -CTGGGACCACTTGCGCATGTCCCAGATGTCATCCACAGTGGCATCCTCC 2468

  contig10_pilo      69252 ACTACCTTGTTCCAGTAGTCACGCTTGGTGCGCTTTACCTGGCGACGATG 69301
                              i                                              
C rnd-3_family-       2467 ACTGCCTTGTTCCAGTAGTCACGCTTGGTGCGCTTTACCTGGCGACGATG 2418

  contig10_pilo      69302 GTAGTTGCGCTCCCTGGCTGCAGTTGCAGTCAGGACTGCATTGGAGCGGC 69351
                                 i                   v         v  v          
C rnd-3_family-       2417 GTAGTTACGCTCCCTGGCTGCAGTTGAAGTCAGGACAGCCTTGGAGCGGC 2368

  contig10_pilo      69352 GCATGCGCCTTGCAAAGTCCTGTGCAGCTCTCTCTGCATCTCGCATCTTC 69401
                                                                             
C rnd-3_family-       2367 GCATGCGCCTTGCAAAGTCCTGTGCAGCTCTCTCTGCATCTCGCATCTTC 2318

  contig10_pilo      69402 TGCAAGCTCTGGGTGAGTGCAGGAGTCCACCAGGGCTGTGAGTGCTTAGA 69451
                                                                             
C rnd-3_family-       2317 TGCAAGCTCTGGGTGAGTGCAGGAGTCCACCAGGGCTGTGAGTGCTTAGA 2268

  contig10_pilo      69452 GGGTCTCCTCTTGGGCACCTTGGCCTGAGTGGCACCATTGAGAGCGTCAT 69501
                                          v                                  
C rnd-3_family-       2267 GGGTCTCCTCTTGGGGACCTTGGCCTGAGTGGCACCATTGAGAGCGTCAT 2218

  contig10_pilo      69502 GGAGTGCTGCTGCAGTGCGCTCTAGGAGCTCAGTGTTGCGAGTGTGCTTG 69551
                                                       i         v           
C rnd-3_family-       2217 GGAGTGCTGCTGCAGTGCGCTCTAGGAGTTCAGTGTTGAGAGTGTGCTTG 2168

  contig10_pilo      69552 GGAGCACTTTGCAGCTCTGCAAGCACTGCTGCATGGCCATGCAGGCGAGA 69601
                                                 i                 v         
C rnd-3_family-       2167 GGAGCACTTTGCAGCTCTGCAAACACTGCTGCATGGCCATCCAGGCGAGA 2118

  contig10_pilo      69602 GCGAAACTCTGCCTTGAACTCAGCCTCGTCTGCATGCTTGAAGTTGTAGC 69651
                                                i                            
C rnd-3_family-       2117 GCGAAACTCTGCCTTGAACTCGGCCTCGTCTGCATGCTTGAAGTTGTAGC 2068

  contig10_pilo      69652 TGGCTCCAGAGCCAAGATCCACATCCTCAGCACCCAGGTCAAAGACCCAG 69701
                                     i     i                 ?               
C rnd-3_family-       2067 TGGCTCCAGAACCAAGGTCCACATCCTCAGCACCNAGGTCAAAGACCCAG 2018

  contig10_pilo      69702 GAGACAGTGTGATGGTCAGAGAAGTCATGGACGTCGTCTGACACCGCAAA 69751
                                                              v              
C rnd-3_family-       2017 GAGACAGTGTGATGGTCAGAGAAGTCATGGACGTCTTCTGACACCGCAAA 1968

  contig10_pilo      69752 GTCTTGCAGCAGACCAAGTGCAGCCATTTGGTCATTCTGCCATACCAAGT 69801
                                                                     i       
C rnd-3_family-       1967 GTCTTGCAGCAGACCAAGTGCAGCCATTTGGTCATTCTGCCACACCAAGT 1918

  contig10_pilo      69802 CCAGAACAGAGGACTGAGAGTGGTTGTCCTTGCGCCAAGTTGTCAC 69847
                                                                   v i   
C rnd-3_family-       1917 CCAGAACAGAGGACTGAGAGTGGTTGTCCTTGCGCCAAGTGGCCAC 1872

Matrix = 20p53g.matrix
Kimura (with divCpGMod) = 2.13
Transitions / transversions = 0.72 (13/18)
Gap_init rate = 0.00 (2 / 1395), avg. gap size = 1.00 (2 / 2)

 
 

 
 
   +   30692    0.9  0.1  0.2  contig10_pilon    69842   74064 (1740815) +  rnd-4_family-763   LTR/Gypsy            1   3908     (0)    29     
 
 ANNOTATION EVIDENCE: 
  2265   0.70 0.00 0.00  contig10_pilon    69842   70128   1744751 +  rnd-4_family-763   LTR/Gypsy         3622   3908       0      
2265 0.70 0.00 0.00 contig10_pilon 69842 70128 (1744751) rnd-4_family-763#LTR/Gypsy 3622 3908 (0) m_b496s001i10

  contig10_pilo      69842 TGTCACGGTGACTGCTATATCACTCTCCGAGACGACGCCCGAGGGGAAGG 69891
                                                                             
  rnd-4_family-       3622 TGTCACGGTGACTGCTATATCACTCTCCGAGACGACGCCCGAGGGGAAGG 3671

  contig10_pilo      69892 GATGAGTGGGGAGAGGGAGGCGCGGATAAGCGGTATGATAGACCAGACCA 69941
                                                                             
  rnd-4_family-       3672 GATGAGTGGGGAGAGGGAGGCGCGGATAAGCGGTATGATAGACCAGACCA 3721

  contig10_pilo      69942 AACCTAAGGTACAAGAGGGGCGACGGCCCATATATATATACTACGGAAGC 69991
                                                         v                   
  rnd-4_family-       3722 AACCTAAGGTACAAGAGGGGCGACGGCCCAGATATATATACTACGGAAGC 3771

  contig10_pilo      69992 AAGGCCGAGGGGCCCCGCGGACCAATTGCGCCACCGACCGGTTGTCACGC 70041
                                                                             
  rnd-4_family-       3772 AAGGCCGAGGGGCCCCGCGGACCAATTGCGCCACCGACCGGTTGTCACGC 3821

  contig10_pilo      70042 GGGCGCCACAGATCAGGCCGAAAGAGACCGCCGGGGCGTCGTCGGCGAGA 70091
                                                                             
  rnd-4_family-       3822 GGGCGCCACAGATCAGGCCGAAAGAGACCGCCGGGGCGTCGTCGGCGAGA 3871

  contig10_pilo      70092 CAGGAAACCCGGGATAAATAGAGCCCAACCAAACCAG 70128
                                                              i 
  rnd-4_family-       3872 CAGGAAACCCGGGATAAATAGAGCCCAACCAAACCGG 3908

Matrix = 20p53g.matrix
Kimura (with divCpGMod) = 0.38
Transitions / transversions = 1.00 (1/1)
Gap_init rate = 0.00 (0 / 286), avg. gap size = 0.0 (0 / 0)

 30692   0.89 0.05 0.20  contig10_pilon    69956   73869   1741010 +  rnd-4_family-763   LTR/Gypsy            1   3908       0      
30692 0.89 0.05 0.20 contig10_pilon 69956 73869 (1741010) rnd-4_family-763#LTR/Gypsy 1 3908 (0) m_b496s001i11

  contig10_pilo      69956 GAGGGGCGACGGCCCATATATATATACTACGGAAGCAAGGCCGAGGGGCC 70005
                                           v                                 
  rnd-4_family-          1 GAGGGGCGACGGCCCAGATATATATACTACGGAAGCAAGGCCGAGGGGCC 50

  contig10_pilo      70006 CCGCGGACCAATTGCGCCACCGACCGGTTGTCACGCGGGCGCCACAGATC 70055
                                                                             
  rnd-4_family-         51 CCGCGGACCAATTGCGCCACCGACCGGTTGTCACGCGGGCGCCACAGATC 100

  contig10_pilo      70056 AGGCCGAAAGAGACCGCCGGGGCGTCGTCGGCGAGACAGGAAACCCGGGA 70105
                                                                             
  rnd-4_family-        101 AGGCCGAAAGAGACCGCCGGGGCGTCGTCGGCGAGACAGGAAACCCGGGA 150

  contig10_pilo      70106 TAAATAGAGCCCAACCAAACCAGAAACATCCCGTCATATTACCATTCTCC 70155
                                                                      v      
  rnd-4_family-        151 TAAATAGAGCCCAACCAAACCAGAAACATCCCGTCATATTACCCTTCTCC 200

  contig10_pilo      70156 TGGGAAGAGACCGAGGTGCTCGCCGAGCGGAGAGACCGCTTGGACGCGCC 70205
                                              i                              
  rnd-4_family-        201 TGGGAAGAGACCGAGGTGCCCGCCGAGCGGAGAGACCGCTTGGACGCGCC 250

  contig10_pilo      70206 GACGAAAGGGCCGCGGGGCCGGGCGCAAGGAGTTTGGGCGCCCGGACGAG 70255
                                 i                                      i?   
  rnd-4_family-        251 GACGAAGGGGCCGCGGGGCCGGGCGCAAGGAGTTTGGGCGCCCGGGNGAG 300

  contig10_pilo      70256 GTGCGAGGGCCACGGGACGGTGAAATGAAACAGCGGGGGAGGAGGGGCCC 70305
                            i           ?      i                         i   
  rnd-4_family-        301 GCGCGAGGGCCACNGGACGGCGAAATGAAACAGCGGGGGAGGAGGGACCC 350

  contig10_pilo      70306 GACGAAGACCGCAACATAACCCCCCTTCTAGCCGTCCAGTGGCCGAGGGG 70355
                                                                             
  rnd-4_family-        351 GACGAAGACCGCAACATAACCCCCCTTCTAGCCGTCCAGTGGCCGAGGGG 400

  contig10_pilo      70356 G-AATGGCCAGGGGAGGTGCGGGTTAGATCGGGCGTGGGGAGACGGACCA 70404
                            -                                                
  rnd-4_family-        401 GGAATGGCCAGGGGAGGTGCGGGTTAGATCGGGCGTGGGGAGACGGACCA 450

  contig10_pilo      70405 TAGCGTGGCCGGGCCGAGCTCCATCCGTCGCGGGATCAAACTCTAAGCGT 70454
                                                      i                      
  rnd-4_family-        451 TAGCGTGGCCGGGCCGAGCTCCATCCGCCGCGGGATCAAACTCTAAGCGT 500

  contig10_pilo      70455 CAGATGCCGCGAAAGGCTCGGCTGCCGCCGTACACGAACCGCGGTACTTC 70504
                                                 i                           
  rnd-4_family-        501 CAGATGCCGCGAAAGGCTCGGCCGCCGCCGTACACGAACCGCGGTACTTC 550

  contig10_pilo      70505 TCGCTCTTCGCCTCCCCGCTTCCCGTCACCGTCCGATATCCTATGACGCC 70554
                                                                             
  rnd-4_family-        551 TCGCTCTTCGCCTCCCCGCTTCCCGTCACCGTCCGATATCCTATGACGCC 600

  contig10_pilo      70555 CACCGCCGCCGACGGACTGACTGCGAAGCCTCTCGGCGAACTTCGGGACC 70604
                                                                             
  rnd-4_family-        601 CACCGCCGCCGACGGACTGACTGCGAAGCCTCTCGGCGAACTTCGGGACC 650

  contig10_pilo      70605 TCAGGGAGCGGGTGTTCACGCTGCATGCCTCCATCCAGCGCCAACTGGAC 70654
                                                                             
  rnd-4_family-        651 TCAGGGAGCGGGTGTTCACGCTGCATGCCTCCATCCAGCGCCAACTGGAC 700

  contig10_pilo      70655 TTCGGGCACTACGCCGCGGACGCGGAGGAGGAGAAGGCGCGGACGGTGCT 70704
                                                                             
  rnd-4_family-        701 TTCGGGCACTACGCCGCGGACGCGGAGGAGGAGAAGGCGCGGACGGTGCT 750

  contig10_pilo      70705 TGCGTCTGCGGGCCAGCGGTTGGAGGCCATTGACGCCGAGGTGCGCCGAC 70754
                                                                             
  rnd-4_family-        751 TGCGTCTGCGGGCCAGCGGTTGGAGGCCATTGACGCCGAGGTGCGCCGAC 800

  contig10_pilo      70755 GCAACGCCGCGACCCGCAATCTGGAGACGGCGGCGTCTCCCCTCGGCATG 70804
                                                                  i          
  rnd-4_family-        801 GCAACGCCGCGACCCGCAATCTGGAGACGGCGGCGTCTCTCCTCGGCATG 850

  contig10_pilo      70805 ACAGCAGCCTCGCTCCCCGTAGTGCCGAAGGTGGAAGGGGTTCGCCGCAC 70854
                                                                             
  rnd-4_family-        851 ACAGCAGCCTCGCTCCCCGTAGTGCCGAAGGTGGAAGGGGTTCGCCGCAC 900

  contig10_pilo      70855 GGGAACGACCTCCTCGACCACGAAGGGCAAGGGCTCATCGGGAGTGGTCG 70904
                                              i              i         i     
  rnd-4_family-        901 GGGAACGACCTCCTCGACCGCGAAGGGCAAGGGCCCATCGGGAGCGGTCG 950

  contig10_pilo      70905 CGTCTGGGTCGAAGCGGCCTTCGGACGTCGGCGCGCGAGGGACCGGCGAC 70954
                                                                             
  rnd-4_family-        951 CGTCTGGGTCGAAGCGGCCTTCGGACGTCGGCGCGCGAGGGACCGGCGAC 1000

  contig10_pilo      70955 GAGGAGAAGGACGCGGTCGGGCCTCCTTTTAACACGGTACGTCTTGGTCA 71004
                                                                             
  rnd-4_family-       1001 GAGGAGAAGGACGCGGTCGGGCCTCCTTTTAACACGGTACGTCTTGGTCA 1050

  contig10_pilo      71005 AAGGTCCTCTTGCTCCGGGGTGAATCCTGCGCTGACATCCTTCGTCCGGG 71054
                                                 v                           
  rnd-4_family-       1051 AAGGTCCTCTTGCTCCGGGGTGTATCCTGCGCTGACATCCTTCGTCCGGG 1100

  contig10_pilo      71055 TCCAGCCTTGTGAGCAGTGCGAGCGCTCCGGCCGGCCGTGTTTTAGACAA 71104
                                                                       v     
  rnd-4_family-       1101 TCCAGCCTTGTGAGCAGTGCGAGCGCTCCGGCCGGCCGTGTTTTCGACAA 1150

  contig10_pilo      71105 GTGTCCGGGTCGGCGACTGCCTGTGCTCGGTGCCACCGGACCCGCGTGGG 71154
                                                        v                    
  rnd-4_family-       1151 GTGTCCGGGTCGGCGACTGCCTGTGCTCGTTGCCACCGGACCCGCGTGGG 1200

  contig10_pilo      71155 GTGTTCTCACAGCGTCGGCCGGTCGGGCACATCGCGCAAGAGGGTGCGAC 71204
                                                                       i     
  rnd-4_family-       1201 GTGTTCTCACAGCGTCGGCCGGTCGGGCACATCGCGCAAGAGGGCGCGAC 1250

  contig10_pilo      71205 AGGACGCTGACGTGGACGGCGATGACGGGGACGATCCGGACCAGGACGAC 71254
                                                 i                           
  rnd-4_family-       1251 AGGACGCTGACGTGGACGGCGACGACGGGGACGATCCGGACCAGGACGAC 1300

  contig10_pilo      71255 GTGGTGGACACCGTCGGCGCGTCCGTGGCGCGGTCGCGCACACGCCGAAG 71304
                                                                             
  rnd-4_family-       1301 GTGGTGGACACCGTCGGCGCGTCCGTGGCGCGGTCGCGCACACGCCGAAG 1350

  contig10_pilo      71305 GCTTCGGTCGCCGGGGACAGCCACCATGGCCGACCTCGAGGTCCTGGGCG 71354
                                                                             
  rnd-4_family-       1351 GCTTCGGTCGCCGGGGACAGCCACCATGGCCGACCTCGAGGTCCTGGGCG 1400

  contig10_pilo      71355 ACCGCATCGTCCAGGGGTTCCAGCTGTCCGTCTCGGCGTTGAGGCGCGAC 71404
                                                                             
  rnd-4_family-       1401 ACCGCATCGTCCAGGGGTTCCAGCTGTCCGTCTCGGCGTTGAGGCGCGAC 1450

  contig10_pilo      71405 CTTCGCAAGTGGACGCAAGAGCTCGTCGATGACCGGCGTGCCCATAAGAC 71454
                                                                             
  rnd-4_family-       1451 CTTCGCAAGTGGACGCAAGAGCTCGTCGATGACCGGCGTGCCCATAAGAC 1500

  contig10_pilo      71455 GGTGCGCCTCGTGTCGCCGGGCGGGAGCGAGCGAGATACCGACTCCAATG 71504
                                                            v                
  rnd-4_family-       1501 GGTGCGCCTCGTGTCGCCGGGCGGGAGCGAGCGCGATACCGACTCCAATG 1550

  contig10_pilo      71505 ACGGCCGAGCGGTGGAGGACGAGGCGAGGAGGTCGCCCACCGACGCGCCG 71554
                                                     i                       
  rnd-4_family-       1551 ACGGCCGAGCGGTGGAGGACGAGGCGGGGAGGTCGCCCACCGACGCGCCG 1600

  contig10_pilo      71555 ACTTCCCCTGGCGCCGCGGGCGTGGAGGCGGAGGCGCCGGCGGGTCCTGT 71604
                                                                    v        
  rnd-4_family-       1601 ACTTCCCCTGGCGCCGCGGGCGTGGAGGCGGAGGCGCCGGCTGGTCCTGT 1650

  contig10_pilo      71605 CGGCGTGCTTGCTGACTTCGGCGCCGACCTGGCCCTTCTTGGCGTGGTAG 71654
                                                                             
  rnd-4_family-       1651 CGGCGTGCTTGCTGACTTCGGCGCCGACCTGGCCCTTCTTGGCGTGGTAG 1700

  contig10_pilo      71655 TGCCGGGGGATGTGCGTCCGATACCCAGCTTTGCGTCGCTGGAGGCGTCT 71704
                                                i                            
  rnd-4_family-       1701 TGCCGGGGGATGTGCGTCCGACACCCAGCTTTGCGTCGCTGGAGGCGTCT 1750

  contig10_pilo      71705 GGGTCCGGGTCCGGATTCCTGCCACGCGTCCAACGGTCCGAGGGTCGGGA 71754
                                                                             
  rnd-4_family-       1751 GGGTCCGGGTCCGGATTCCTGCCACGCGTCCAACGGTCCGAGGGTCGGGA 1800

  contig10_pilo      71755 TGCGTCCGTGATAGGGGAGAAGTCGGTCGATGCGGGCCAACCCATGGAGG 71804
                                                                             
  rnd-4_family-       1801 TGCGTCCGTGATAGGGGAGAAGTCGGTCGATGCGGGCCAACCCATGGAGG 1850

  contig10_pilo      71805 AGTAAGACCGTGTCCGGGTATGATTCGGGTAGTGGTGTGGAGGCTCGTAA 71854
                                               i    i                        
  rnd-4_family-       1851 AGTAAGACCGTGTCCGGGTACGATTTGGGTAGTGGTGTGGAGGCTCGTAA 1900

  contig10_pilo      71855 AATATGTAAATAGAGAGGCGGAAAGACGAGCATAAATACGGGGTATGAGA 71904
                                                                             
  rnd-4_family-       1901 AATATGTAAATAGAGAGGCGGAAAGACGAGCATAAATACGGGGTATGAGA 1950

  contig10_pilo      71905 AACGACCGGGACCGTGACTGTGGAGAGCATCCGACCGAAGCGGCGTCCGA 71954
                                                                             
  rnd-4_family-       1951 AACGACCGGGACCGTGACTGTGGAGAGCATCCGACCGAAGCGGCGTCCGA 2000

  contig10_pilo      71955 CCGAGGTAGGGTCCGATTCTGGGCACGGTCGCCGAGGGGGAGGGAACGAC 72004
                                                                             
  rnd-4_family-       2001 CCGAGGTAGGGTCCGATTCTGGGCACGGTCGCCGAGGGGGAGGGAACGAC 2050

  contig10_pilo      72005 GGGTGGCGTCACCTGTACGCCAGTAGATATCGACAAAGAAGGGCGGTTTC 72054
                                                                             
  rnd-4_family-       2051 GGGTGGCGTCACCTGTACGCCAGTAGATATCGACAAAGAAGGGCGGTTTC 2100

  contig10_pilo      72055 AGCTTCGCAAAGCGTGCTGCTGTACAAGGCAGGGGCAACCGTCCGGAGCG 72104
                                                               i             
  rnd-4_family-       2101 AGCTTCGCAAAGCGTGCTGCTGTACAAGGCAGGGGCGACCGTCCGGAGCG 2150

  contig10_pilo      72105 TTCAAAGACGGTGGGCCGGGCCGGGGGACGCGATCCGCAGGACCAGGGCG 72154
                                                                             
  rnd-4_family-       2151 TTCAAAGACGGTGGGCCGGGCCGGGGGACGCGATCCGCAGGACCAGGGCG 2200

  contig10_pilo      72155 TCGTCGGCGTACGTGGCACGATATTCGCGGACGTCCCAGACAAGGCACGC 72204
                                                                             
  rnd-4_family-       2201 TCGTCGGCGTACGTGGCACGATATTCGCGGACGTCCCAGACAAGGCACGC 2250

  contig10_pilo      72205 CGTAAGCCGAACGACCTGGAGGGCTCGTTCCGTCGTAGGCGACGTGATGA 72254
                                                                          v  
  rnd-4_family-       2251 CGTAAGCCGAACGACCTGGAGGGCTCGTTCCGTCGTAGGCGACGTGAGGA 2300

  contig10_pilo      72255 GATGAGGGCCGAGGCGAGGTGGGAACGAGAAGACCACTAGCTCGGTGCGA 72304
                                                                             
  rnd-4_family-       2301 GATGAGGGCCGAGGCGAGGTGGGAACGAGAAGACCACTAGCTCGGTGCGA 2350

  contig10_pilo      72305 GCCCGTCGGCCTCGGTCATTGCGCACGACGGTCGGGAGTACCCAGTAACG 72354
                                                                             
  rnd-4_family-       2351 GCCCGTCGGCCTCGGTCATTGCGCACGACGGTCGGGAGTACCCAGTAACG 2400

  contig10_pilo      72355 CGAGATATCGAGCGGGAGATGTCTCGGGGCAGTGAAGCGGCCGTAGTGAG 72404
                                                           i                 
  rnd-4_family-       2401 CGAGATATCGAGCGGGAGATGTCTCGGGGCAGCGAAGCGGCCGTAGTGAG 2450

  contig10_pilo      72405 TGAAGCGGTCTAGCATCCAGAAGAAAGGTTGGGGAGGGGCCACGAGCATG 72454
                                                    v                        
  rnd-4_family-       2451 TGAAGCGGTCTAGCATCCAGAAGAACGGTTGGGGAGGGGCCACGAGCATG 2500

  contig10_pilo      72455 GCGATGTCGAGCCGGGTGTCGTCCGGGTCCGGCGACCGGATACGAGCGCG 72504
                                                                             
  rnd-4_family-       2501 GCGATGTCGAGCCGGGTGTCGTCCGGGTCCGGCGACCGGATACGAGCGCG 2550

  contig10_pilo      72505 AAGGGGGATGAGCCCCAGCGGAGGTGTGGCCGGGCGCGCGTCCTCAAGCT 72554
                                                                             
  rnd-4_family-       2551 AAGGGGGATGAGCCCCAGCGGAGGTGTGGCCGGGCGCGCGTCCTCAAGCT 2600

  contig10_pilo      72555 CGGACGACTCGTCGGCGGCGAGAGGTGACTCGCCGGTGCTGATGGGTGAT 72604
                              i                                              
  rnd-4_family-       2601 CGGGCGACTCGTCGGCGGCGAGAGGTGACTCGCCGGTGCTGATGGGTGAT 2650

  contig10_pilo      72605 TCGCCGAACAGCTCTTGGGATCCGTCGCCAAAGTCCTCGCCGAGGTCCAT 72654
                                                                             
  rnd-4_family-       2651 TCGCCGAACAGCTCTTGGGATCCGTCGCCAAAGTCCTCGCCGAGGTCCAT 2700

  contig10_pilo      72655 GTCCGCATCCTGGTCCAGGTCCTGTGCGGCGCCTTGGTCCTCATTTGGCT 72704
                                                                             
  rnd-4_family-       2701 GTCCGCATCCTGGTCCAGGTCCTGTGCGGCGCCTTGGTCCTCATTTGGCT 2750

  contig10_pilo      72705 CCGGTGGTTCGGCGTGGTACTCCTCCACGAGTCCGGTTCCGTCCAGGTCG 72754
                                                                             
  rnd-4_family-       2751 CCGGTGGTTCGGCGTGGTACTCCTCCACGAGTCCGGTTCCGTCCAGGTCG 2800

  contig10_pilo      72755 GCGGCCAGTTGCCAGCTAGCGTCCTCGGGGGGAAAGCCCATCCATTGGAC 72804
                                                                             
  rnd-4_family-       2801 GCGGCCAGTTGCCAGCTAGCGTCCTCGGGGGGAAAGCCCATCCATTGGAC 2850

  contig10_pilo      72805 GAGATACTGAGGAACTCCTCGGTGTACGCGGTGGTCCAGGACTGCCGCCG 72854
                                                                             
  rnd-4_family-       2851 GAGATACTGAGGAACTCCTCGGTGTACGCGGTGGTCCAGGACTGCCGCCG 2900

  contig10_pilo      72855 GTATGTATTGAGGGTTAACTATGGTCTCGTCGTACTGGGACATTTTGTGA 72904
                                                                             
  rnd-4_family-       2901 GTATGTATTGAGGGTTAACTATGGTCTCGTCGTACTGGGACATTTTGTGA 2950

  contig10_pilo      72905 GCGTTCGGTCCGAACAAAGGGGCGTGTCGGGGAAAAGGGGGGAAAGAGGC 72954
                                                                             
  rnd-4_family-       2951 GCGTTCGGTCCGAACAAAGGGGCGTGTCGGGGAAAAGGGGGGAAAGAGGC 3000

  contig10_pilo      72955 GTCGGTCTGGCGGCCGATCCGGATTATATGGTGTATGTGTCGTAAGCGTT 73004
                                                                             
  rnd-4_family-       3001 GTCGGTCTGGCGGCCGATCCGGATTATATGGTGTATGTGTCGTAAGCGTT 3050

  contig10_pilo      73005 GATGCGTGGGGATGATGGGGGTCACGATCCGAGGAAGATGATGTGAATGC 73054
                                                                             
  rnd-4_family-       3051 GATGCGTGGGGATGATGGGGGTCACGATCCGAGGAAGATGATGTGAATGC 3100

  contig10_pilo      73055 GCCGCGCGCACCACGGGGGGGGGGGGGGGGGGAACTTGCTCTCGGGATGC 73104
                                         --------                            
  rnd-4_family-       3101 GCCGCGCGCACCAC--------GGGGGGGGGGAACTTGCTCTCGGGATGC 3142

  contig10_pilo      73105 GCGGAGGGTGAGGTTCTGGAGAGAGGGGTCGGCCGTGGGAGGTTTAGAGG 73154
                                                                             
  rnd-4_family-       3143 GCGGAGGGTGAGGTTCTGGAGAGAGGGGTCGGCCGTGGGAGGTTTAGAGG 3192

  contig10_pilo      73155 TTGACCGAGGGGTGGGGACGCGCAGCGGGCGCGTTCGGCGAGTAGGGCTT 73204
                                                                             
  rnd-4_family-       3193 TTGACCGAGGGGTGGGGACGCGCAGCGGGCGCGTTCGGCGAGTAGGGCTT 3242

  contig10_pilo      73205 GT-GGGGGGGTGGTCTCGCCGCGGATGACCTGGCTCTGATGAAGAGGAGA 73253
                             -                           v                   
  rnd-4_family-       3243 GTGGGGGGGGTGGTCTCGCCGCGGATGACCGGGCTCTGATGAAGAGGAGA 3292

  contig10_pilo      73254 GATCAGGAGCCGGTGATGGAATGAGCTTTCGGATTTGACGACCGTATGGG 73303
                                                                             
  rnd-4_family-       3293 GATCAGGAGCCGGTGATGGAATGAGCTTTCGGATTTGACGACCGTATGGG 3342

  contig10_pilo      73304 TCTGGAGGGGTCCTCGTTGGCTGAGTGCCTGACCCGAAGGCCAGTGGGCA 73353
                                                             v               
  rnd-4_family-       3343 TCTGGAGGGGTCCTCGTTGGCTGAGTGCCTGACCAGAAGGCCAGTGGGCA 3392

  contig10_pilo      73354 AGGCTCGGCGTCCGGAGGGTGGGTCCTGCAGGGTCCTGGTGGGCCGATGA 73403
                                                                             
  rnd-4_family-       3393 AGGCTCGGCGTCCGGAGGGTGGGTCCTGCAGGGTCCTGGTGGGCCGATGA 3442

  contig10_pilo      73404 GTGGGCGCGGCGGTCGCGACGGGTCCTCGTCGACGCGAGGGGTCTCCTAG 73453
                                                                             
  rnd-4_family-       3443 GTGGGCGCGGCGGTCGCGACGGGTCCTCGTCGACGCGAGGGGTCTCCTAG 3492

  contig10_pilo      73454 CCGGTCCGAGGGGTCGGATTCCTTCCTGGACCGAGCAACGCCGACCGAAC 73503
                                                                             
  rnd-4_family-       3493 CCGGTCCGAGGGGTCGGATTCCTTCCTGGACCGAGCAACGCCGACCGAAC 3542

  contig10_pilo      73504 GGTTCGCCCGGATTGGAAGGGTGGTGAGTAAGGGGTATGGGGATCTCGAG 73553
                                                                             
  rnd-4_family-       3543 GGTTCGCCCGGATTGGAAGGGTGGTGAGTAAGGGGTATGGGGATCTCGAG 3592

  contig10_pilo      73554 CGTTGGGAGGGAGGGTAGGTTTGGTCTTATGTCACGGTGACTGCTATATC 73603
                              i                                              
  rnd-4_family-       3593 CGTCGGGAGGGAGGGTAGGTTTGGTCTTATGTCACGGTGACTGCTATATC 3642

  contig10_pilo      73604 ACTCTCCGAGACGACGCCCGAGGGGAAGGGATGAGTGGGGAGAGGGAGGC 73653
                                                                             
  rnd-4_family-       3643 ACTCTCCGAGACGACGCCCGAGGGGAAGGGATGAGTGGGGAGAGGGAGGC 3692

  contig10_pilo      73654 GCGGATAAGCGGTATGATAGACCAGACCAAACCTAAGGTACAAGAGGGGC 73703
                                                                             
  rnd-4_family-       3693 GCGGATAAGCGGTATGATAGACCAGACCAAACCTAAGGTACAAGAGGGGC 3742

  contig10_pilo      73704 GACGGCCCATATATATATACTACGGAAGCAAGGCCGAGGGGCCCCGCGGA 73753
                                    v                                        
  rnd-4_family-       3743 GACGGCCCAGATATATATACTACGGAAGCAAGGCCGAGGGGCCCCGCGGA 3792

  contig10_pilo      73754 CCAATTGCGCCACCGACCGGTTGTCACGCGGGCGCCACAGATCAGGCCGA 73803
                                                                             
  rnd-4_family-       3793 CCAATTGCGCCACCGACCGGTTGTCACGCGGGCGCCACAGATCAGGCCGA 3842

  contig10_pilo      73804 AAGAGACCGCCGGGGCGTCGTCGGCGAGACAGGAAACCCGGGATAAATAG 73853
                                                                             
  rnd-4_family-       3843 AAGAGACCGCCGGGGCGTCGTCGGCGAGACAGGAAACCCGGGATAAATAG 3892

  contig10_pilo      73854 AGCCCAACCAAACCAG 73869
                                         i 
  rnd-4_family-       3893 AGCCCAACCAAACCGG 3908

Matrix = 20p53g.matrix
Kimura (with divCpGMod) = 0.62
Transitions / transversions = 1.92 (23/12)
Gap_init rate = 0.00 (10 / 3913), avg. gap size = 1.00 (10 / 10)

  2728   2.17 0.00 0.00  contig10_pilon    73697   74064   1740815 +  rnd-4_family-763   LTR/Gypsy            1    368    3540      
2728 2.17 0.00 0.00 contig10_pilon 73697 74064 (1740815) rnd-4_family-763#LTR/Gypsy 1 368 (3540) m_b496s001i12

  contig10_pilo      73697 GAGGGGCGACGGCCCATATATATATACTACGGAAGCAAGGCCGAGGGGCC 73746
                                           v                                 
  rnd-4_family-          1 GAGGGGCGACGGCCCAGATATATATACTACGGAAGCAAGGCCGAGGGGCC 50

  contig10_pilo      73747 CCGCGGACCAATTGCGCCACCGACCGGTTGTCACGCGGGCGCCACAGATC 73796
                                                                             
  rnd-4_family-         51 CCGCGGACCAATTGCGCCACCGACCGGTTGTCACGCGGGCGCCACAGATC 100

  contig10_pilo      73797 AGGCCGAAAGAGACCGCCGGGGCGTCGTCGGCGAGACAGGAAACCCGGGA 73846
                                                                             
  rnd-4_family-        101 AGGCCGAAAGAGACCGCCGGGGCGTCGTCGGCGAGACAGGAAACCCGGGA 150

  contig10_pilo      73847 TAAATAGAGCCCAACCAAACCAGAAACATCCCGTCATATTACCATTCTCC 73896
                                                                      v      
  rnd-4_family-        151 TAAATAGAGCCCAACCAAACCAGAAACATCCCGTCATATTACCCTTCTCC 200

  contig10_pilo      73897 TGGGAAGAGACCGAGGTGCTCGCCGAGCGGAGAGACCGCTTGGACGCGCC 73946
                                              i                              
  rnd-4_family-        201 TGGGAAGAGACCGAGGTGCCCGCCGAGCGGAGAGACCGCTTGGACGCGCC 250

  contig10_pilo      73947 GACGAAAGGGCCGCGGGGCCGGGCGCAAGGAGTTTGGGCGCCCGGACGAG 73996
                                 i                                      i?   
  rnd-4_family-        251 GACGAAGGGGCCGCGGGGCCGGGCGCAAGGAGTTTGGGCGCCCGGGNGAG 300

  contig10_pilo      73997 GTGCGAGGGCCACGGGACGGCGAAATGAAACAGCGGGGGAGGAGGGGCCC 74046
                            i           ?                                i   
  rnd-4_family-        301 GCGCGAGGGCCACNGGACGGCGAAATGAAACAGCGGGGGAGGAGGGACCC 350

  contig10_pilo      74047 GACGAAGACCGCAACACA 74064
                                           i 
  rnd-4_family-        351 GACGAAGACCGCAACATA 368

Matrix = 20p53g.matrix
Kimura (with divCpGMod) = 1.97
Transitions / transversions = 3.00 (6/2)
Gap_init rate = 0.00 (0 / 367), avg. gap size = 0.0 (0 / 0)

 
 
 
 
  +     850    5.4  0.0  0.0  contig10_pilon    74065   74173 (1740706) +  rnd-3_family-161   LINE/Tad1         3263   3371  (1190)    26     
 
 ANNOTATION EVIDENCE: 
   850   5.41 0.00 0.00  contig10_pilon    74063   74173   1740706 +  rnd-3_family-161   LINE/Tad1         3261   3371    1190      
850 5.41 0.00 0.00 contig10_pilon 74063 74173 (1740706) rnd-3_family-161#LINE/Tad1 3261 3371 (1190) m_b496s001i13

  contig10_pilo      74063 CATCTGCCTGGATGGTAGAGTCAGCAGCTGCAAGCCAGTTGAGAATGGAG 74112
                                                         i                   
  rnd-3_family-       3261 CATCTGCCTGGATGGTAGAGTCAGCAGCTGTAAGCCAGTTGAGAATGGAG 3310

  contig10_pilo      74113 TGCCTCAGGGCTCTCCAGTCTCTGGCATTCTCTCTGCATTCTACTCCACT 74162
                                                       i        v        i   
  rnd-3_family-       3311 TGCCTCAGGGCTCTCCAGTCTCTGGCATCCTCTCTGCCTTCTACTCTACT 3360

  contig10_pilo      74163 GGACTCATTAA 74173
                             i      i 
  rnd-3_family-       3361 GGGCTCATTGA 3371

Matrix = 20p53g.matrix
Kimura (with divCpGMod) = 5.67
Transitions / transversions = 5.00 (5/1)
Gap_init rate = 0.00 (0 / 110), avg. gap size = 0.0 (0 / 0)

 
 

 
 
   +    9353    2.0  1.7  0.0  contig10_pilon    74176   75394 (1739485) +  rnd-4_family-1185  LINE/Tad1            1   1240   (548)    30     
 
 ANNOTATION EVIDENCE: 
  9353   1.97 1.72 0.00  contig10_pilon    74176   75394   1739485 +  rnd-4_family-1185  LINE/Tad1            1   1240     548      
9353 1.97 1.72 0.00 contig10_pilon 74176 75394 (1739485) rnd-4_family-1185#LINE/Tad1 1 1240 (548) m_b496s001i14

  contig10_pilo      74176 AGCTTCTTGGACTCTTACCACCACCTGG---------------------C 74204
                                                       --------------------- 
  rnd-4_family-          1 AGCTTCTTGGACTCTTACCACCACCTGGTCAGAGTCAGTCTACCTCTGGC 50

  contig10_pilo      74205 ACACTCTGTCCTCTACCTCTCGTCCTACTGGCCCTCCTAAGGTCCCAGGG 74254
                                                                  i  v       
  rnd-4_family-         51 ACACTCTGTCCTCTACCTCTCGTCCTACTGGCCCTCCTAGGGGCCCAGGG 100

  contig10_pilo      74255 GGTCCCCCTGGCCCTCCAAAGAGTGGCTCCAGTCTGCTCATGGCTCCCCA 74304
                                                           i                 
  rnd-4_family-        101 GGTCCCCCTGGCCCTCCAAAGAGTGGCTCCAGCCTGCTCATGGCTCCCCA 150

  contig10_pilo      74305 GTGGAGCTTGCCCTTGGGTGCTCCAGCCCAACCTGCCCTGGCCACTCCAG 74354
                               i  ?                                          
  rnd-4_family-        151 GTGGGGCNTGCCCTTGGGTGCTCCAGCCCAACCTGCCCTGGCCACTCCAG 200

  contig10_pilo      74355 CCCCCACCACTGCCCCTGGCCCCACCTCTGGCCCTGGCCCTCCCTCTAGG 74404
                                                                             
  rnd-4_family-        201 CCCCCACCACTGCCCCTGGCCCCACCTCTGGCCCTGGCCCTCCCTCTAGG 250

  contig10_pilo      74405 ACCACTCCCCCCTCCCTTGCCCCCCTTAAGCGGCCTGTAGGTGCTCTGGC 74454
                                                                             
  rnd-4_family-        251 ACCACTCCCCCCTCCCTTGCCCCCCTTAAGCGGCCTGTAGGTGCTCTGGC 300

  contig10_pilo      74455 TAGTCCTGGGCAACTTGCAGGTTCTGCTGGGTCTGCACCACCTCCTGGGC 74504
                                                                             
  rnd-4_family-        301 TAGTCCTGGGCAACTTGCAGGTTCTGCTGGGTCTGCACCACCTCCTGGGC 350

  contig10_pilo      74505 GCATTTCTGGGCCTAAAAAGTCCAGGAAAAGTGGTCCTATTCCCTCTACC 74554
                                                          i              i   
  rnd-4_family-        351 GCATTTCTGGGCCTAAAAAGTCCAGGAAAAGCGGTCCTATTCCCTCCACC 400

  contig10_pilo      74555 ACCTCCCAGCACCTCCCACCCGAAAGGGCTAGCTCCCAGGATGACTGGTT 74604
                                                                   i  v      
  rnd-4_family-        401 ACCTCCCAGCACCTCCCACCCGAAAGGGCTAGCTCCCAGGGTGCCTGGTT 450

  contig10_pilo      74605 GAGCCCTGGTGAGGTGCTGCCTCAGGAGGCCGCGCGTTCATCAATTTCAA 74654
                                                                     i       
  rnd-4_family-        451 GAGCCCTGGTGAGGTGCTGCCTCAGGAGGCCGCGCGTTCATCGATTTCAA 500

  contig10_pilo      74655 ACTCCGCTCCGGATACTTCCGGACCCCCTCCTCCTGCCCCTGCTGCCGGG 74704
                                                                      i   v  
  rnd-4_family-        501 ACTCCGCTCCGGATACTTCCGGACCCCCTCCTCCTGCCCCTGCCGCCTGG 550

  contig10_pilo      74705 CAGGCTACCTTCAGCTTCAATCTGCCTGATACTCCGGCTGTGGGTGCCCC 74754
                                                                             
  rnd-4_family-        551 CAGGCTACCTTCAGCTTCAATCTGCCTGATACTCCGGCTGTGGGTGCCCC 600

  contig10_pilo      74755 TAGGGATGCTCTGCCCTCTCAGCCAGTTGCTCCTGACCAGGACCAGGATA 74804
                                                                             
  rnd-4_family-        601 TAGGGATGCTCTGCCCTCTCAGCCAGTTGCTCCTGACCAGGACCAGGATA 650

  contig10_pilo      74805 TGGAGCCACAGAATGACTCTGAGGCTCTGGACCCTGATACACTTGCTGCT 74854
                           i                     v                           
  rnd-4_family-        651 CGGAGCCACAGAATGACTCTGATGCTCTGGACCCTGATACACTTGCTGCT 700

  contig10_pilo      74855 GAACTGGATGCCAAGCCTGTGACCTCCAGGAAGGCTAGGGACATTATTTC 74904
                                                                             
  rnd-4_family-        701 GAACTGGATGCCAAGCCTGTGACCTCCAGGAAGGCTAGGGACATTATTTC 750

  contig10_pilo      74905 CTTCCTGACCTACTGCCTGGACATGGTCAAGGTGATCTATGATCGCAAGG 74954
                                                                             
  rnd-4_family-        751 CTTCCTGACCTACTGCCTGGACATGGTCAAGGTGATCTATGATCGCAAGG 800

  contig10_pilo      74955 ACCGCTTTCCTAGTGCTGCTGGAGCTGCAGTTGAGGACTTTTGCCGCTCT 75004
                                                          i                  
  rnd-4_family-        801 ACCGCTTTCCTAGTGCTGCTGGAGCTGCAGTCGAGGACTTTTGCCGCTCT 850

  contig10_pilo      75005 GTGGTGCAATCCTCCACCTCCCAGCTGCTTCTTGCACAGCAGGAGGAGCT 75054
                                                 i                           
  rnd-4_family-        851 GTGGTGCAATCCTCCACCTCCCGGCTGCTTCTTGCACAGCAGGAGGAGCT 900

  contig10_pilo      75055 TAAGAAGCTCTCTGCCATGGTAGCCTCCCTTCAGGGCACCCTTGGGTGTG 75104
                                                                i            
  rnd-4_family-        901 TAAGAAGCTCTCTGCCATGGTAGCCTCCCTTCAGGGCGCCCTTGGGTGTG 950

  contig10_pilo      75105 CTTCTGCTGCTGCAACCTCGTCTGCCCCAGCTGCCCAGCCTGCTGCTGCT 75154
                                                                             
  rnd-4_family-        951 CTTCTGCTGCTGCAACCTCGTCTGCCCCAGCTGCCCAGCCTGCTGCTGCT 1000

  contig10_pilo      75155 GCTCCTGCTCGTCCTGCCAAGGACTCCTACGCTGCTCGCGCTGCCAATTC 75204
                                                                          v  
  rnd-4_family-       1001 GCTCCTGCTCGTCCTGCCAAGGACTCCTACGCTGCTCGCGCTGCCAAGTC 1050

  contig10_pilo      75205 AGCTCCACCACTACCCGCTGTGCCCCGCGTGCCAGCCTCAGCTACTCCTC 75254
                                      i                         i            
  rnd-4_family-       1051 AGCTCCACCACCACCCGCTGTGCCCCGCGTGCCAGCCCCAGCTACTCCTC 1100

  contig10_pilo      75255 TTACTCGCCATGACGCTCGCCGCCTGATCCTCATGGTCAAGCCCAGTGCT 75304
                                     i                                       
  rnd-4_family-       1101 TTACTCGCCACGACGCTCGCCGCCTGATCCTCATGGTCAAGCCCAGTGCT 1150

  contig10_pilo      75305 GCTGTTCGCCTCGCTGTCAAGAACGCCGCTGCCGCCCGCTCTGCCATCAA 75354
                                                                             
  rnd-4_family-       1151 GCTGTTCGCCTCGCTGTCAAGAACGCCGCTGCCGCCCGCTCTGCCATCAA 1200

  contig10_pilo      75355 CACTGCCCTCGCTGGCGTCTCACCTGCTGCTGCACCTCAT 75394
                              i        i        v   v              
  rnd-4_family-       1201 CACCGCCCTCGCCGGCGTCTCCCCTCCTGCTGCACCTCAT 1240

Matrix = 20p53g.matrix
Kimura (with divCpGMod) = 1.24
Transitions / transversions = 2.43 (17/7)
Gap_init rate = 0.00 (1 / 1218), avg. gap size = 21.00 (21 / 1)

 
 
 
 
 
  +   12948    1.9  0.0  0.0  contig10_pilon    75395   77046 (1737833) C  rnd-4_family-1185  LINE/Tad1        (135)   1653       2    31     
 
 ANNOTATION EVIDENCE: 
 12948   1.94 0.00 0.00  contig10_pilon    75395   77046   1737833 C  rnd-4_family-1185  LINE/Tad1            2   1653     135      
12948 1.94 0.00 0.00 contig10_pilon 75395 77046 (1737833) C rnd-4_family-1185#LINE/Tad1 (135) 1653 2 m_b496s001i15

  contig10_pilo      75395 GCGTGGCCAAAGAGGAAGACACCATGGCGAAGGACGTACTGCAAGTCATC 75444
                                                                             
C rnd-4_family-       1653 GCGTGGCCAAAGAGGAAGACACCATGGCGAAGGACGTACTGCAAGTCATC 1604

  contig10_pilo      75445 TTCACGAGTGAAGGAAAGTAGCACAGAGCCAGAGCCCTGCTGGATGATGC 75494
                                                                             
C rnd-4_family-       1603 TTCACGAGTGAAGGAAAGTAGCACAGAGCCAGAGCCCTGCTGGATGATGC 1554

  contig10_pilo      75495 GAATAGGGGGGCCCAACCACATGGGAGGGGCGGCCCAGGCAAACTGGTGG 75544
                             i    v                                          
C rnd-4_family-       1553 GAGTAGGCGGGCCCAACCACATGGGAGGGGCGGCCCAGGCAAACTGGTGG 1504

  contig10_pilo      75545 GCAGTAAACTCCTCCACCTCGGCCTGCAACTCCTGTGATGTCGGCAGGGG 75594
                                                              i  i           
C rnd-4_family-       1503 GCAGTAAACTCCTCCACCTCGGCCTGCAACTCCTGCGACGTCGGCAGGGG 1454

  contig10_pilo      75595 CTGGCCAAGGTCAGGAGGCACAACATCAGAGACAACGGCCTTGAACCAAA 75644
                                                                             
C rnd-4_family-       1453 CTGGCCAAGGTCAGGAGGCACAACATCAGAGACAACGGCCTTGAACCAAA 1404

  contig10_pilo      75645 GGCTGTCACGAGCAGCACCAGAAAAGGTGGCCTCCTGGGGCAGGAAGGCA 75694
                                                      i                      
C rnd-4_family-       1403 GGCTGTCACGAGCAGCACCAGAAAAGGCGGCCTCCTGGGGCAGGAAGGCA 1354

  contig10_pilo      75695 GAGGCAATGAGAGCGCTGTGGGGCTCCAAGTCGGCAGCAGTGAGACCTGG 75744
                                i                                            
C rnd-4_family-       1353 GAGGCGATGAGAGCGCTGTGGGGCTCCAAGTCGGCAGCAGTGAGACCTGG 1304

  contig10_pilo      75745 AAGCGCAAAGACAACAACGTTGCCTGAGCGAGTCAGGCCAACACCACTCA 75794
                                                                             
C rnd-4_family-       1303 AAGCGCAAAGACAACAACGTTGCCTGAGCGAGTCAGGCCAACACCACTCA 1254

  contig10_pilo      75795 CCCCAAGCCTGGGATGAGGTGCAGCAGCAGGTGAGACGCCAGCGAGGGCA 75844
                                                      v   v        i        i
C rnd-4_family-       1253 CCCCAAGCCTGGGATGAGGTGCAGCAGGAGGGGAGACGCCGGCGAGGGCG 1204

  contig10_pilo      75845 GTGTTGATGGCAGAGCGGGCGGCAGCGGCGTTCTTGACAGCGAGGCGAAC 75894
                                                                             
C rnd-4_family-       1203 GTGTTGATGGCAGAGCGGGCGGCAGCGGCGTTCTTGACAGCGAGGCGAAC 1154

  contig10_pilo      75895 AGCAGCACTGGGCTTGACCATGAGGATCAGGCGGCGAGCGTCATGGCGAG 75944
                                                                     i       
C rnd-4_family-       1153 AGCAGCACTGGGCTTGACCATGAGGATCAGGCGGCGAGCGTCGTGGCGAG 1104

  contig10_pilo      75945 TAAGAGGAGTAACTGAGGCTGGCACGCGGGGCACAGCGGGTGGTGGTGGA 75994
                                      i   i                                  
C rnd-4_family-       1103 TAAGAGGAGTAGCTGGGGCTGGCACGCGGGGCACAGCGGGTGGTGGTGGA 1054

  contig10_pilo      75995 GCTGACTTGGCAGCACAAGCAGCGTAGGAGTCCTTGGCAGGACGAGCAGG 76044
                                         i i                                 
C rnd-4_family-       1053 GCTGACTTGGCAGCGCGAGCAGCGTAGGAGTCCTTGGCAGGACGAGCAGG 1004

  contig10_pilo      76045 AGCAGCAGCAGCAGGCTGGGCAGCTGGGGCAGACGAGGTCGCAGCAGCAG 76094
                                                                  i          
C rnd-4_family-       1003 AGCAGCAGCAGCAGGCTGGGCAGCTGGGGCAGACGAGGTTGCAGCAGCAG 954

  contig10_pilo      76095 AAGCACACCCAAGGGTGCCCTGAAGGGAGGCTACCATGGCAGAGAGCTTC 76144
                                          i                                  
C rnd-4_family-        953 AAGCACACCCAAGGGCGCCCTGAAGGGAGGCTACCATGGCAGAGAGCTTC 904

  contig10_pilo      76145 TTAAGCTCCTCCTGCTGTGCAAGAAGCAGCTGGGAGGTGGAGGATTGCAC 76194
                                                         i                   
C rnd-4_family-        903 TTAAGCTCCTCCTGCTGTGCAAGAAGCAGCCGGGAGGTGGAGGATTGCAC 854

  contig10_pilo      76195 CACAGAGCGGCAAAAGTCCTCAACTGCAGCTCCAGCAGCACTAGGAAAGC 76244
                                                i                            
C rnd-4_family-        853 CACAGAGCGGCAAAAGTCCTCGACTGCAGCTCCAGCAGCACTAGGAAAGC 804

  contig10_pilo      76245 GGTCCTTGCGATCATAGATCACCTTGACCATGTCCAGGCAGTAGGTCAGG 76294
                                                                             
C rnd-4_family-        803 GGTCCTTGCGATCATAGATCACCTTGACCATGTCCAGGCAGTAGGTCAGG 754

  contig10_pilo      76295 AAGGAAATAATGTCCCTAGCCTTCCTGGAGGTCACAGGCTTGGCATCCAG 76344
                                                                             
C rnd-4_family-        753 AAGGAAATAATGTCCCTAGCCTTCCTGGAGGTCACAGGCTTGGCATCCAG 704

  contig10_pilo      76345 TTCAGCAGCAAGTGTATCAGGGTCCAGAGCCTCAGAGTCATTCTGTGGCT 76394
                                                         v                   
C rnd-4_family-        703 TTCAGCAGCAAGTGTATCAGGGTCCAGAGCATCAGAGTCATTCTGTGGCT 654

  contig10_pilo      76395 CCATATCCTGGTCCTGGTCAGGAGCAACTGGCTGAGAGGGCAGAGCATCC 76444
                             i                                               
C rnd-4_family-        653 CCGTATCCTGGTCCTGGTCAGGAGCAACTGGCTGAGAGGGCAGAGCATCC 604

  contig10_pilo      76445 CTAGGGGCACCCACAGCCGAAGTATCAGGCAGATTGAAGCTGAAGGTAGC 76494
                                              i                              
C rnd-4_family-        603 CTAGGGGCACCCACAGCCGGAGTATCAGGCAGATTGAAGCTGAAGGTAGC 554

  contig10_pilo      76495 CTGCCCGGCAGCAGGGGCAGGAGGAGGGGGTCCGGAAGTATCCGGAGCGG 76544
                                v   i                                        
C rnd-4_family-        553 CTGCCAGGCGGCAGGGGCAGGAGGAGGGGGTCCGGAAGTATCCGGAGCGG 504

  contig10_pilo      76545 AGTTTGAAATTGATGAACGCGCGGCCTCCTGAGGCAGCACCTCACCAGGG 76594
                                     i                                       
C rnd-4_family-        503 AGTTTGAAATCGATGAACGCGCGGCCTCCTGAGGCAGCACCTCACCAGGG 454

  contig10_pilo      76595 CTCAACCAGTCATCCTGGGAGCTAGCCCTTTCGGGTGGGAGGTGCTGGGA 76644
                                    v  i                                     
C rnd-4_family-        453 CTCAACCAGGCACCCTGGGAGCTAGCCCTTTCGGGTGGGAGGTGCTGGGA 404

  contig10_pilo      76645 GGTGGTAGAGGGAATAGGACCACTTTTCCTGGACTTTTTAGGCCCAGAAA 76694
                                 i              i                            
C rnd-4_family-        403 GGTGGTGGAGGGAATAGGACCGCTTTTCCTGGACTTTTTAGGCCCAGAAA 354

  contig10_pilo      76695 TGCGCCCAGGAGGTGGTGCAGACCCAGCAGAACCTGCAAGTTGCCCAGGA 76744
                                                                             
C rnd-4_family-        353 TGCGCCCAGGAGGTGGTGCAGACCCAGCAGAACCTGCAAGTTGCCCAGGA 304

  contig10_pilo      76745 CTAGCCAGAGCACCTACAGGCCGCTTAAGGGGGGCAAGGGAGGGGGGAGT 76794
                                                                             
C rnd-4_family-        303 CTAGCCAGAGCACCTACAGGCCGCTTAAGGGGGGCAAGGGAGGGGGGAGT 254

  contig10_pilo      76795 GGTCCTAGAGGGAGGGCCAGGGCCAGAGGTGGGGCCAGGGGCAGTGGTGG 76844
                                                                             
C rnd-4_family-        253 GGTCCTAGAGGGAGGGCCAGGGCCAGAGGTGGGGCCAGGGGCAGTGGTGG 204

  contig10_pilo      76845 GGGCTGGAGTGGCCAGGGCAGGTTGGGCTGGAGCACCCAAGGACATGCCC 76894
                                                                     i  ?    
C rnd-4_family-        203 GGGCTGGAGTGGCCAGGGCAGGTTGGGCTGGAGCACCCAAGGGCANGCCC 154

  contig10_pilo      76895 CACTGGGGAGCCATGAGCAGACTGGAGCCACTCTTTGGTGGGCCAGGGGG 76944
                                               i                 v           
C rnd-4_family-        153 CACTGGGGAGCCATGAGCAGGCTGGAGCCACTCTTTGGAGGGCCAGGGGG 104

  contig10_pilo      76945 ACCCCCTGGGCCCCTAGGAGGGCCAGTAGGACGAGAGGTAGAGGACAGAG 76994
                                                                             
C rnd-4_family-        103 ACCCCCTGGGCCCCTAGGAGGGCCAGTAGGACGAGAGGTAGAGGACAGAG 54

  contig10_pilo      76995 TGTGCCAGAGGTAGACTGACTCTGACCAGGTGGTGGTAAGAGTCCAAGAA 77044
                                                                             
C rnd-4_family-         53 TGTGCCAGAGGTAGACTGACTCTGACCAGGTGGTGGTAAGAGTCCAAGAA 4

  contig10_pilo      77045 GC 77046
                             
C rnd-4_family-          3 GC 2

Matrix = 20p53g.matrix
Kimura (with divCpGMod) = 1.07
Transitions / transversions = 3.57 (25/7)
Gap_init rate = 0.00 (0 / 1651), avg. gap size = 0.0 (0 / 0)

 
 

 
 
   +    8051    2.9  0.4  0.6  contig10_pilon    77418   78458 (1736421) C  rnd-3_family-40    LTR/Gypsy      (13745)   1039       1    32     
 
 ANNOTATION EVIDENCE: 
  8051   2.90 0.38 0.58  contig10_pilon    77418   78458   1736421 C  rnd-3_family-40    LTR/Gypsy            1   1039   13745      
8051 2.90 0.38 0.58 contig10_pilon 77418 78458 (1736421) C rnd-3_family-40#LTR/Gypsy (13745) 1039 1 m_b496s001i16

  contig10_pilo      77418 CGATGAGATCACTTCCTGATTGGGCGGTCGGCGAGCGCGGGTGCGGAGTT 77467
                              vi           i                      v          
C rnd-3_family-       1039 CGAGAAGATCACTTCCCGATTGGGCGGTCGGCGAGCGCGCGTGCGGAGTT 990

  contig10_pilo      77468 ATCACGCTCAATCTCGAACCAAGCAGAGGCAACTAGACACGTGAACGACG 77517
                                      i          v                           
C rnd-3_family-        989 ATCACGCTCAACCTCGAACCAACCAGAGGCAACTAGACACGTGAACGACG 940

  contig10_pilo      77518 GGTACCTAGCAGGCCCAGGGCTGCTCTCGCATTGTATTGCGCGCAAAACC 77567
                            ----i            v              i         ?      
C rnd-3_family-        939 G----TTAGCAGGCCCAGCGCTGCTCTCGCATTATATTGCGCGNAAAACC 894

  contig10_pilo      77568 GCGCATGAAGCGCGAAGTAGGACACCAATGGCTGGGGTGTGGAGGCATAT 77617
                                                      i                      
C rnd-3_family-        893 GCGCATGAAGCGCGAAGTAGGACACCAGTGGCTGGGGTGTGGAGGCATAT 844

  contig10_pilo      77618 CGGCTTCCATTGCGCGTCGCGATGACTACATGAAATATGTTGCGTGTAGC 77667
                                                 i       i                   
C rnd-3_family-        843 CGGCTTCCATTGCGCGTCGCGACGACTACACGAAATATGTTGCGTGTAGC 794

  contig10_pilo      77668 GCGTCCTCGTGAGGCGGAAAAGGCGTTATGACGCTATGGATGCACGGCGA 77717
                                                                             
C rnd-3_family-        793 GCGTCCTCGTGAGGCGGAAAAGGCGTTATGACGCTATGGATGCACGGCGA 744

  contig10_pilo      77718 CGCGCATTGGAGGCCGATGTGCAGCCAAACGACCAGCCCAGCATCATCCG 77767
                                                 i           i               
C rnd-3_family-        743 CGCGCATTGGAGGCCGATGTGCGGCCAAACGACCGGCCCAGCATCATCCG 694

  contig10_pilo      77768 CGCACACACCAGGGGGAACGGGTCTGCCGATCAGCAGGTCACGTGACCTC 77817
                              i                    i                  i      
C rnd-3_family-        693 CGCGCACACCAGGGGGAACGGGTCCGCCGATCAGCAGGTCACGCGACCTC 644

  contig10_pilo      77818 GTGCACACCTCGAAGGCTCGGTAGGAACAGTAAGACCACTGGATAATGAA 77867
                                                                             
C rnd-3_family-        643 GTGCACACCTCGAAGGCTCGGTAGGAACAGTAAGACCACTGGATAATGAA 594

  contig10_pilo      77868 GTTGATGGTCGTTTTCCATTCACAGTGGACTTCTCACCGTAATCTCATCT 77917
                                                                             
C rnd-3_family-        593 GTTGATGGTCGTTTTCCATTCACAGTGGACTTCTCACCGTAATCTCATCT 544

  contig10_pilo      77918 GTGGAACGGAGGATTTGTGGGTAAGGAACGTTTCGACCGGCTTGAACAGA 77967
                                                                             
C rnd-3_family-        543 GTGGAACGGAGGATTTGTGGGTAAGGAACGTTTCGACCGGCTTGAACAGA 494

  contig10_pilo      77968 GATGGTACTGAGTCATTGAATCTCAACCAGGGCTATCTCTTGAGGACGAG 78017
                             i                                               
C rnd-3_family-        493 GACGGTACTGAGTCATTGAATCTCAACCAGGGCTATCTCTTGAGGACGAG 444

  contig10_pilo      78018 CAACGCACAGCAATACAAAACTTACCATATGTACTGCAGGGAACGGCGAA 78067
                                                                             
C rnd-3_family-        443 CAACGCACAGCAATACAAAACTTACCATATGTACTGCAGGGAACGGCGAA 394

  contig10_pilo      78068 ACGAGAAATTGACTGCGAACAGAAAGAGGACGCGACGGAATAAATTGGTG 78117
                                    i                                        
C rnd-3_family-        393 ACGAGAAATCGACTGCGAACAGAAAGAGGACGCGACGGAATAAATTGGTG 344

  contig10_pilo      78118 AAATGCTCATACTGTTCTGAATGAGCTGCCCATGCTCACCAACACGAATG 78167
                             i                                               
C rnd-3_family-        343 AAGTGCTCATACTGTTCTGAATGAGCTGCCCATGCTCACCAACACGAATG 294

  contig10_pilo      78168 ACATTGGGATCGCAGGGCTGCCCATGCAGGGAGACGGGTCCGGAAGCATT 78217
                                                                             
C rnd-3_family-        293 ACATTGGGATCGCAGGGCTGCCCATGCAGGGAGACGGGTCCGGAAGCATT 244

  contig10_pilo      78218 TCGGCGCTGGACTTGGGCGGATTTTTGCCCAAGTCC--CTGACTGGGCAG 78265
                                                  i--          --            
C rnd-3_family-        243 TCGGCGCTGGACTTGGGCGGATTC--GCCCAAGTCCGCCTGACTGGGCAG 196

  contig10_pilo      78266 TCTCGGCTGTGGCCGACATCTTCCCGACCACCACCACTCATGGTGTCCAA 78315
                                   v       ?                       i  i      
C rnd-3_family-        195 TCTCGGCTTTGGCCGANATCTTCCCGACCACCACCACTCACGGCGTCCAA 146

  contig10_pilo      78316 CGCCATCAGCACCC-CGCA-ACCCCACCCCCTCCCTGCCTTGCTCTGCGC 78363
                                         -    -               i   i         ?
C rnd-3_family-        145 CGCCATCAGCACCCGCGCACACCCCACCCCCTCCCCGCCCTGCTCTGCGN 96

  contig10_pilo      78364 CGGCCACCCTAGACGTTAGGTGTGGCCGTAGTCGCGGCACAGGTAATATA 78413
                                                     v           i           
C rnd-3_family-         95 CGGCCACCCTAGACGTTAGGTGTGGCGGTAGTCGCGGCGCAGGTAATATA 46

  contig10_pilo      78414 TCCAACTGCTTCGCGCGCACAGACGTGGGCGCTCCCCTGCCAGTC 78458
                                  i          i                          
C rnd-3_family-         45 TCCAACTACTTCGCGCGCGCAGACGTGGGCGCTCCCCTGCCAGTC 1

Matrix = 20p53g.matrix
Kimura (with divCpGMod) = 1.53
Transitions / transversions = 4.00 (24/6)
Gap_init rate = 0.01 (9 / 1040), avg. gap size = 1.11 (10 / 9)

 
 
 
 
 
  +     686    2.3  0.0  0.0  contig10_pilon    80802   80888 (1733991) C  rnd-4_family-867   Unknown           (18)  16439   16353    33     
 
 ANNOTATION EVIDENCE: 
   686   2.30 0.00 0.00  contig10_pilon    80802   80888   1733991 C  rnd-4_family-867   Unknown          16353  16439      18      
686 2.30 0.00 0.00 contig10_pilon 80802 80888 (1733991) C rnd-4_family-867#Unknown (18) 16439 16353 m_b496s001i17

  contig10_pilo      80802 TTTTGGTTTGCTACTCCCAGCTCCCAGAACTCCCAGGTCCTGACTTTGAC 80851
                                                                             
C rnd-4_family-      16439 TTTTGGTTTGCTACTCCCAGCTCCCAGAACTCCCAGGTCCTGACTTTGAC 16390

  contig10_pilo      80852 TGCTCCTGACTCCTGAACTCGAGACTTGTACTCCCAA 80888
                                             i       i          
C rnd-4_family-      16389 TGCTCCTGACTCCTGAACCCGAGACTCGTACTCCCAA 16353

Matrix = 20p53g.matrix
Kimura (with divCpGMod) = 1.28
Transitions / transversions = 1.00 (2/0)
Gap_init rate = 0.00 (0 / 86), avg. gap size = 0.0 (0 / 0)

 
 

 
 
   +     339    4.5  0.0  0.0  contig10_pilon    81764   81807 (1733072) C  rnd-4_family-867   Unknown         (3969)  12488   12445    34     
 
 ANNOTATION EVIDENCE: 
   339   4.55 0.00 0.00  contig10_pilon    81764   81807   1733072 C  rnd-4_family-867   Unknown          12445  12488    3969      
339 4.55 0.00 0.00 contig10_pilon 81764 81807 (1733072) C rnd-4_family-867#Unknown (3969) 12488 12445 m_b496s001i18

  contig10_pilo      81764 GCAGGCGGCTCGGGCTACATCGTCAAAGGCTTTGGACCAGCAGT 81807
                                           i i                         
C rnd-4_family-      12488 GCAGGCGGCTCGGGCTGCGTCGTCAAAGGCTTTGGACCAGCAGT 12445

Matrix = 20p53g.matrix
Kimura (with divCpGMod) = 2.56
Transitions / transversions = 1.00 (2/0)
Gap_init rate = 0.00 (0 / 43), avg. gap size = 0.0 (0 / 0)

 
 
 
 
 
  +   10753    2.2  2.2  0.0  contig10_pilon    81985   83364 (1731515) +  rnd-4_family-867   Unknown             55   1464 (14993)    35     
 
 ANNOTATION EVIDENCE: 
 10753   2.17 2.17 0.00  contig10_pilon    81985   83364   1731515 +  rnd-4_family-867   Unknown             55   1464   14993      
10753 2.17 2.17 0.00 contig10_pilon 81985 83364 (1731515) rnd-4_family-867#Unknown 55 1464 (14993) m_b496s001i19

  contig10_pilo      81985 ACTGCGTGGACAAGGGCAGATAGATGATGCGCATCGCCTTGTAGTCCCAC 82034
                                                           v                 
  rnd-4_family-         55 ACTGCGTGGACAAGGGCAGATAGATGATGCGCTTCGCCTTGTAGTCCCAC 104

  contig10_pilo      82035 CACTCCGTCATGCCGCGCTTGATGTTGTGGAAGTCGCGATGCTTGCGACT 82084
                                                                             
  rnd-4_family-        105 CACTCCGTCATGCCGCGCTTGATGTTGTGGAAGTCGCGATGCTTGCGACT 154

  contig10_pilo      82085 GATCAGCCAGAACGTTGGGGCGTCGTCGTTTGCCTCATCCACGCTGAAGT 82134
                                                                             
  rnd-4_family-        155 GATCAGCCAGAACGTTGGGGCGTCGTCGTTTGCCTCATCCACGCTGAAGT 204

  contig10_pilo      82135 ACCACACAGTGTAGTACGTCAGCGGGCGCGTCTTCTTGCTCTCGGGCTCT 82184
                                                                     i       
  rnd-4_family-        205 ACCACACAGTGTAGTACGTCAGCGGGCGCGTCTTCTTGCTCTTGGGCTCT 254

  contig10_pilo      82185 GAGATATACCAGGTCTTGGGTTCGACCTGGCGCCGCTCAGCCTCGCTCCG 82234
                                                                             
  rnd-4_family-        255 GAGATATACCAGGTCTTGGGTTCGACCTGGCGCCGCTCAGCCTCGCTCCG 304

  contig10_pilo      82235 TCGAGCTACTTCGCCTTGTTCGGGTTCCTCGAAAAGGTCGCGAATGAGCG 82284
                                                      v              i       
  rnd-4_family-        305 TCGAGCTACTTCGCCTTGTTCGGGTTCGTCGAAAAGGTCGCGGATGAGCG 354

  contig10_pilo      82285 TGTCCTTGAGCTCAGAGACGGTCTTTGCCTGGACGGGGGGA--------- 82325
                                            i                       ---------
  rnd-4_family-        355 TGTCCTTGAGCTCAGAGGCGGTCTTTGCCTGGACGGGGGGAGAACGAGGA 404

  contig10_pilo      82326 CTAGGGGGACCGTACATTTCGTTCA---TTTCCTCCTCGCAAGAGTCAGA 82372
                                                    ---                   i  
  rnd-4_family-        405 CTAGGGGGACCGTACATTTCGTTCAGCCTTTCCTCCTCGCAAGAGTCGGA 454

  contig10_pilo      82373 ATCTTCGTCGATGTCGAAGACTCTCGCCGACCGTGGACGGTTCAGACGGG 82422
                                                v i     i                    
  rnd-4_family-        455 ATCTTCGTCGATGTCGAAGACGCCCGCCGGCCGTGGACGGTTCAGACGGG 504

  contig10_pilo      82423 GCGGAGGCGGCGGCGCAGGCTGGGGTGGAGGCGGCAGTGGATGCGGCGGC 82472
                                                    i        vi i v i        
  rnd-4_family-        505 GCGGAGGCGGCGGCGCAGGCTGGGGCGGAGGCGGAGGCGCACGCGGCGGC 554

  contig10_pilo      82473 GCTG---------------GAGCATGTACGGCTTGACCGGACTGTGCAGC 82507
                               ---------------     i                         
  rnd-4_family-        555 GCTGAGGGATGCGGCAATGGAGCACGTACGGCTTGACCGGACTGTGCAGC 604

  contig10_pilo      82508 AGACGGTTGGGATGCGGGCGGTAGCGGAGCAGGCTCGTCTTC---GTCCT 82554
                                                 i       i   ?       ---     
  rnd-4_family-        605 AGACGGTTGGGATGCGGGCGGTGGCGGAGCGGGCNCGTCTTCATCGTCCT 654

  contig10_pilo      82555 GCACACTATCATCCACAGGCTTGGGCAACGATGTGACCTTAGGTGCTGGT 82604
                             i i                                             
  rnd-4_family-        655 GCGCGCTATCATCCACAGGCTTGGGCAACGATGTGACCTTAGGTGCTGGT 704

  contig10_pilo      82605 TGCGGCAGGCTCGCGTCCAGACCGAGCAAGTTGACCATGCGAGCAACATG 82654
                                                                       i     
  rnd-4_family-        705 TGCGGCAGGCTCGCGTCCAGACCGAGCAAGTTGACCATGCGAGCGACATG 754

  contig10_pilo      82655 CTGCTCCGGCACGACCAACTTCAACAAATTTCGCCAATGAGGCACGGACA 82704
                                                                      i      
  rnd-4_family-        755 CTGCTCCGGCACGACCAACTTCAACAAATTTCGCCAATGAGGCGCGGACA 804

  contig10_pilo      82705 TGCCTGGATTCTGACTGGCATCCGTCGTCGCCAGTCGCCGAAGAAAATAA 82754
                                                                             
  rnd-4_family-        805 TGCCTGGATTCTGACTGGCATCCGTCGTCGCCAGTCGCCGAAGAAAATAA 854

  contig10_pilo      82755 GGACGGATCTTGAGCCAATGCAGCAAGGGTTTAGCGGTATTCAAGTTTCC 82804
                                                                             
  rnd-4_family-        855 GGACGGATCTTGAGCCAATGCAGCAAGGGTTTAGCGGTATTCAAGTTTCC 904

  contig10_pilo      82805 GCTCTTGACTTCCTGGGGCCGCGTGGCACTGACGAGGGGTACACTGCCCT 82854
                                                          i            i     
  rnd-4_family-        905 GCTCTTGACTTCCTGGGGCCGCGTGGCACTGGCGAGGGGTACACCGCCCT 954

  contig10_pilo      82855 TGCCCTTGCGTAAGTTAAAGCCGGGCTCCTTTGCGGCGTTCCAGGCGTTG 82904
                                                                v            
  rnd-4_family-        955 TGCCCTTGCGTAAGTTAAAGCCGGGCTCCTTTGCGGCCTTCCAGGCGTTG 1004

  contig10_pilo      82905 ACGAAATACGCTGCCAAGGGGACCTTGTGCAGCTGCAACTTGCCGATGTT 82954
                                     v                                       
  rnd-4_family-       1005 ACGAAATACGATGCCAAGGGGACCTTGTGCAGCTGCAACTTGCCGATGTT 1054

  contig10_pilo      82955 CGGCGGCACAGGCAGCACGTCGGCGGTCATGAGGTCCAGCTGCCGCTCCC 83004
                                                                             
  rnd-4_family-       1055 CGGCGGCACAGGCAGCACGTCGGCGGTCATGAGGTCCAGCTGCCGCTCCC 1104

  contig10_pilo      83005 GCGATACATTCACGCGCCCGGAGACGTTATGAGCGGGCATGAATACCGTT 83054
                                                                             
  rnd-4_family-       1105 GCGATACATTCACGCGCCCGGAGACGTTATGAGCGGGCATGAATACCGTT 1154

  contig10_pilo      83055 GGGAACCAGTGCGGCTGCTCCGTGCTCTTGTAAGCCGAAATCCTGTCTGG 83104
                                          i                                  
  rnd-4_family-       1155 GGGAACCAGTGCGGCCGCTCCGTGCTCTTGTAAGCCGAAATCCTGTCTGG 1204

  contig10_pilo      83105 CGCCACGAGCACAATGTTGTCCAGGCCAATTGTCGAGTTCTTCCCTTCGA 83154
                                                                             
  rnd-4_family-       1205 CGCCACGAGCACAATGTTGTCCAGGCCAATTGTCGAGTTCTTCCCTTCGA 1254

  contig10_pilo      83155 GCAGCTTCGCAGTCGTTCCGCGGTTGCGCTTCTTTGGCGGGGCATCCGCG 83204
                                                                             
  rnd-4_family-       1255 GCAGCTTCGCAGTCGTTCCGCGGTTGCGCTTCTTTGGCGGGGCATCCGCG 1304

  contig10_pilo      83205 CTGGAACCCAGGTCTTTGCAGTTCTGGTCAGTAACCTATCATGAGAAATA 83254
                                    i                                        
  rnd-4_family-       1305 CTGGAACCCGGGTCTTTGCAGTTCTGGTCAGTAACCTATCATGAGAAATA 1354

  contig10_pilo      83255 GCCAACAGATACATACCCTGTCTTTTGCGCTTGGACGACATGCCCGACCC 83304
                                                                             
  rnd-4_family-       1355 GCCAACAGATACATACCCTGTCTTTTGCGCTTGGACGACATGCCCGACCC 1404

  contig10_pilo      83305 TTTGGCGCCTGTCGATGGCCCGGCTGCTGACAGCTGCGGCACTGGAGGCA 83354
                              v                                    i         
  rnd-4_family-       1405 TTTCGCGCCTGTCGATGGCCCGGCTGCTGACAGCTGCGGCGCTGGAGGCA 1454

  contig10_pilo      83355 GATGCCCTGC 83364
                                     
  rnd-4_family-       1455 GATGCCCTGC 1464

Matrix = 20p53g.matrix
Kimura (with divCpGMod) = 1.27
Transitions / transversions = 2.75 (22/8)
Gap_init rate = 0.00 (4 / 1379), avg. gap size = 7.50 (30 / 4)

 
 

 
 
   +    1355    0.6  0.0  0.0  contig10_pilon    83442   83604 (1731275) +  rnd-4_family-3316  LTR/Gypsy            1    163   (360)    36     
 
 ANNOTATION EVIDENCE: 
  1355   0.61 0.00 0.00  contig10_pilon    83442   83604   1731275 +  rnd-4_family-3316  LTR/Gypsy            1    163     360      
1355 0.61 0.00 0.00 contig10_pilon 83442 83604 (1731275) rnd-4_family-3316#LTR/Gypsy 1 163 (360) m_b496s001i20

  contig10_pilo      83442 AGAACCTGACACTGCCCCCTGGTTCCAGGAGGATTCCAGGTGGAATTCCG 83491
                                                                             
  rnd-4_family-          1 AGAACCTGACACTGCCCCCTGGTTCCAGGAGGATTCCAGGTGGAATTCCG 50

  contig10_pilo      83492 GTGGAAAACCTGCTCCAGACAAAATATTTTCTAAGGGGTGAAAAATTATG 83541
                                                                  i          
  rnd-4_family-         51 GTGGAAAACCTGCTCCAGACAAAATATTTTCTAAGGGGTAAAAAATTATG 100

  contig10_pilo      83542 TTAGACATCACAAATAAGGTTCAGAGATGCACAGCCGCCTCAGGAAACAT 83591
                                                                             
  rnd-4_family-        101 TTAGACATCACAAATAAGGTTCAGAGATGCACAGCCGCCTCAGGAAACAT 150

  contig10_pilo      83592 GAAAACGTTCTAG 83604
                                        
  rnd-4_family-        151 GAAAACGTTCTAG 163

Matrix = 20p53g.matrix
Kimura (with divCpGMod) = 0.61
Transitions / transversions = 1.00 (1/0)
Gap_init rate = 0.00 (0 / 162), avg. gap size = 0.0 (0 / 0)

  1702   0.90 4.95 0.43  contig10_pilon    83603   83604   1731275 +  rnd-4_family-3316  LTR/Gypsy          292    293     230      
1702 0.90 4.95 0.43 contig10_pilon 83603 83604 (1731275) rnd-4_family-3316#LTR/Gypsy 292 293 (230) m_b496s001i21

  contig10_pilo      83603 AG 83604
                            i
  rnd-4_family-        292 AA 293

Matrix = 20p53g.matrix
Kimura (with divCpGMod) = 0.50
Transitions / transversions = 1.00 (1/0)
Gap_init rate = 0.00 (0 / 1), avg. gap size = 0.0 (0 / 0)

 
 
 
 
  +      52    0.0  0.0  3.7  contig10_pilon    83605   83660 (1731219) +  (TAACC)n           Simple_repeat        1     54     (0)    37     
 
 ANNOTATION EVIDENCE: 
    52   0.00 0.00 3.70  contig10_pilon    83605   83660   1731219 +  (TAACC)n           Simple_repeat        1     54       0      
52 0.00 0.00 3.70 contig10_pilon 83605 83660 (1731219) (TAACC)n#Simple_repeat 1 54 (0) c_b496s251i0

  contig10_pilo      83605 TAACCTAACCTAACCTAACCTAACCTAACCCCTAACCTAACCTAACCTAA 83654
                                                       --                    
  (TAACC)n#Simp          1 TAACCTAACCTAACCTAACCTAACCTAA--CCTAACCTAACCTAACCTAA 48

  contig10_pilo      83655 CCTAAC 83660
                                 
  (TAACC)n#Simp         49 CCTAAC 54

Matrix = Unknown
Transitions / transversions = 1.00 (0/0)
Gap_init rate = 0.04 (2 / 55), avg. gap size = 1.00 (2 / 2)

 
 

 
   +    1702    0.9  5.0  0.4  contig10_pilon    83661   83879 (1731000) +  rnd-4_family-3316  LTR/Gypsy          294    522     (1)    36     
 
 ANNOTATION EVIDENCE: 
  1702   0.90 4.95 0.43  contig10_pilon    83661   83879   1731000 +  rnd-4_family-3316  LTR/Gypsy          294    522       1      
1702 0.90 4.95 0.43 contig10_pilon 83661 83879 (1731000) rnd-4_family-3316#LTR/Gypsy 294 522 (1) m_b496s001i21

  contig10_pilo      83661 ACTTAATCACAGTTTTTGATGAATTTCCACATTGTTTCCAGTGGAATTCC 83710
                                                                             
  rnd-4_family-        294 ACTTAATCACAGTTTTTGATGAATTTCCACATTGTTTCCAGTGGAATTCC 343

  contig10_pilo      83711 ACCGGAATTCGGCCATTCTGGCCCATCTGATCCAACGGTCCAGTGGAATT 83760
                                                      i                      
  rnd-4_family-        344 ACCGGAATTCGGCCATTCTGGCCCATCCGATCCAACGGTCCAGTGGAATT 393

  contig10_pilo      83761 CCACCCAAATTCCACCTGAGGGGGTATACCATCAAAATATGATGGATTTG 83810
                                                                             
  rnd-4_family-        394 CCACCCAAATTCCACCTGAGGGGGTATACCATCAAAATATGATGGATTTG 443

  contig10_pilo      83811 ACATTCTGACACTTTTTTTTGCATAGTTGCATTTT-----------TCCA 83849
                                       -                      -----------    
  rnd-4_family-        444 ACATTCTGACAC-TTTTTTTGCATAGTTGCATTTTTCCACCTGGAATCCA 492

  contig10_pilo      83850 CCTGGAATTGGGGGGCACAGTCACTTACAG 83879
                                                         
  rnd-4_family-        493 CCTGGAATTGGGGGGCACAGTCACTTACAG 522

Matrix = 20p53g.matrix
Kimura (with divCpGMod) = 0.50
Transitions / transversions = 1.00 (1/0)
Gap_init rate = 0.01 (2 / 218), avg. gap size = 6.00 (12 / 2)

 
 
 
 
 
  +   11640    2.1  0.6  0.0  contig10_pilon    84310   85760 (1729119) +  rnd-4_family-867   Unknown           5226   6685  (9772)    38     
 
 ANNOTATION EVIDENCE: 
 11640   2.07 0.62 0.00  contig10_pilon    84310   85760   1729119 +  rnd-4_family-867   Unknown           5226   6685    9772      
11640 2.07 0.62 0.00 contig10_pilon 84310 85760 (1729119) rnd-4_family-867#Unknown 5226 6685 (9772) m_b496s001i22

  contig10_pilo      84310 ATCCAAAGCTAGTCATGTGCGTCCGCGCTGCTGCGGAAACTCCTACTCAC 84359
                                                                             
  rnd-4_family-       5226 ATCCAAAGCTAGTCATGTGCGTCCGCGCTGCTGCGGAAACTCCTACTCAC 5275

  contig10_pilo      84360 GTGCGGAATAAACTCAGGACATCCTATTCAAGAGTCTCATGGACTTCGGG 84409
                                                                             
  rnd-4_family-       5276 GTGCGGAATAAACTCAGGACATCCTATTCAAGAGTCTCATGGACTTCGGG 5325

  contig10_pilo      84410 CACAGTGTCGGTGTGAAAATTCGCGCTGGCGCAGATCAGAATGCGAGAGA 84459
                            i                                                
  rnd-4_family-       5326 CGCAGTGTCGGTGTGAAAATTCGCGCTGGCGCAGATCAGAATGCGAGAGA 5375

  contig10_pilo      84460 CGCGGAAGAATGCTATACTCGGCAGGAGCATCAAGACGAACTGCACGGCG 84509
                                                                             
  rnd-4_family-       5376 CGCGGAAGAATGCTATACTCGGCAGGAGCATCAAGACGAACTGCACGGCG 5425

  contig10_pilo      84510 TATTCCAGTTGGTATCCATCTGGAGGGCCCTCGGTCGATCGGTGAGCTCA 84559
                                                                             
  rnd-4_family-       5426 TATTCCAGTTGGTATCCATCTGGAGGGCCCTCGGTCGATCGGTGAGCTCA 5475

  contig10_pilo      84560 GATGATCAGCCTAGAGTGTGTGTGCTCACCGGGTGAAGGCAAACAATGGC 84609
                                                                   i         
  rnd-4_family-       5476 GATGATCAGCCTAGAGTGTGTGTGCTCACCGGGTGAAGGCGAACAATGGC 5525

  contig10_pilo      84610 CCTGCGGGTCCCGCTCGCGATCTCTTCAAGAAAACAGACAACCTCAACGC 84659
                                   v                                         
  rnd-4_family-       5526 CCTGCGGGGCCCGCTCGCGATCTCTTCAAGAAAACAGACAACCTCAACGC 5575

  contig10_pilo      84660 GGGGCTCGCGTTGCTTCGAGAGCTGCGCCTCGTGACCATGCGACTCATGG 84709
                                                      v                      
  rnd-4_family-       5576 GGGGCTCGCGTTGCTTCGAGAGCTGCGGCTCGTGACCATGCGACTCATGG 5625

  contig10_pilo      84710 ACATGTTCCAGCTCATCGACCCCGAGCAGTACAACCTCTTCAACGATGCC 84759
                                                                             
  rnd-4_family-       5626 ACATGTTCCAGCTCATCGACCCCGAGCAGTACAACCTCTTCAACGATGCC 5675

  contig10_pilo      84760 TCGGAGAAGTTGAAAGCCATGTACCCCGCGTATGCCGCGCTGGCTGCTGC 84809
                                                        i              i     
  rnd-4_family-       5676 TCGGAGAAGTTGAAAGCCATGTACCCCGCATATGCCGCGCTGGCCGCTGC 5725

  contig10_pilo      84810 CGACCCCTCCTTCCTCCACAGGAGAAGTCTCATCTACAATCGAGAGACCG 84859
                                              i                              
  rnd-4_family-       5726 CGACCCCTCCTTCCTCCACGGGAGAAGTCTCATCTACAATCGAGAGACCG 5775

  contig10_pilo      84860 GTGAACACACTGACCGCCGCGATATGAAGCTCGCATGGACGCCGATTCTC 84909
                            i                                                
  rnd-4_family-       5776 GCGAACACACTGACCGCCGCGATATGAAGCTCGCATGGACGCCGATTCTC 5825

  contig10_pilo      84910 ACAGTGGGTAAATACCGCGAAGGCACGCTTTGTGTCGCAGGTTTGGACAT 84959
                                                         i       i    i      
  rnd-4_family-       5826 ACAGTGGGTAAATACCGCGAAGGCACGCTTCGTGTCGCGGGTTCGGACAT 5875

  contig10_pilo      84960 TAACTATCTCCCCGGAACACTTGCGTTCATTAGAGGGGGCATCTTGAAGC 85009
                                                                             
  rnd-4_family-       5876 TAACTATCTCCCCGGAACACTTGCGTTCATTAGAGGGGGCATCTTGAAGC 5925

  contig10_pilo      85010 ACTCTGTTCAATTCGCAGGCAGTCAGCGCATCGCGATTGCGCACTTCATG 85059
                                               i                             
  rnd-4_family-       5926 ACTCTGTTCAATTCGCAGGCGGTCAGCGCATCGCGATTGCGCACTTCATG 5975

  contig10_pilo      85060 CACAAGAATGTCCTTGCGGAGGTCGGTGCAACGAAGGTCAAGCTTTTCAC 85109
                                                                             
  rnd-4_family-       5976 CACAAGAATGTCCTTGCGGAGGTCGGTGCAACGAAGGTCAAGCTTTTCAC 6025

  contig10_pilo      85110 ATACAAAGAAATGGCGGGCTATAAGCCGCCCTTCGACGAAGGCAACGGTG 85159
                                                         i                   
  rnd-4_family-       6026 ATACAAAGAAATGGCGGGCTATAAGCCGCCTTTCGACGAAGGCAACGGTG 6075

  contig10_pilo      85160 ACTGCGATGAACCCATGAATTAGTTTCTGAATAGCGAATGTATTCAACTC 85209
                                        v                 v    v             
  rnd-4_family-       6076 ACTGCGATGAACCGATGAATTAGTTTCTGAAGAGCGCATGTATTCAACTC 6125

  contig10_pilo      85210 ACCCTGGCAAGCATATATGCACTCATATCTCACCTTACTAATTTTCGGGC 85259
                                                                             
  rnd-4_family-       6126 ACCCTGGCAAGCATATATGCACTCATATCTCACCTTACTAATTTTCGGGC 6175

  contig10_pilo      85260 ACTCATTCATCATGAACAAATGAAAGCATTAAGATTGGCGGTTAAGAGGG 85309
                                                                             
  rnd-4_family-       6176 ACTCATTCATCATGAACAAATGAAAGCATTAAGATTGGCGGTTAAGAGGG 6225

  contig10_pilo      85310 TCAAGGACAGGCTCATCGAGATTACAAAGTGATAAAAATAAAGTAAGGGA 85359
                                                            v        v       
  rnd-4_family-       6226 TCAAGGACAGGCTCATCGAGATTACAAAGTGATTAAAATAAATTAAGGGA 6275

  contig10_pilo      85360 AGGGTATTCACGCCGAACGAGACTTCTTCGTCACCGGCTGGAGTTCCTGC 85409
                                     v                                       
  rnd-4_family-       6276 AGGGTATTCAAGCCGAACGAGACTTCTTCGTCACCGGCTGGAGTTCCTGC 6325

  contig10_pilo      85410 TGCTCATCGACCTCGCCCTGCATGTCTCTCTTGCGCTTGTCCCTGCTCGA 85459
                                                                             
  rnd-4_family-       6326 TGCTCATCGACCTCGCCCTGCATGTCTCTCTTGCGCTTGTCCCTGCTCGA 6375

  contig10_pilo      85460 CGGTGCCGCGGGTGCCTGCCTGGCACTGGCCACGTTGCGGCTCTTCGACG 85509
                                                         v    i              
  rnd-4_family-       6376 CGGTGCCGCGGGTGCCTGCCTGGCACTGGCGACGTCGCGGCTCTTCGACG 6425

  contig10_pilo      85510 GGCCGGGAACGGGCACCGGGGTGGCGAACGGGTTAGGTTTGGGGCGGCCA 85559
                                                                             
  rnd-4_family-       6426 GGCCGGGAACGGGCACCGGGGTGGCGAACGGGTTAGGTTTGGGGCGGCCA 6475

  contig10_pilo      85560 TTGCGGCTGGCCGAACCAGCCATTGGCCGCTGCATGATTGCCGTCCCGCT 85609
                                                                             
  rnd-4_family-       6476 TTGCGGCTGGCCGAACCAGCCATTGGCCGCTGCATGATTGCCGTCCCGCT 6525

  contig10_pilo      85610 GGCGGCGCCACCACGGAGGCCAGCGTTGGCAAGGGGAGTGGCAGCGGT-- 85657
                                       i                 i                 --
  rnd-4_family-       6526 GGCGGCGCCACCGCGGAGGCCAGCGTTGGCGAGGGGAGTGGCAGCGGTGG 6575

  contig10_pilo      85658 -------GGCGGCGGCGGCGGTGCTTCGCGGCACGGTGGCAGGGGCGACA 85700
                           -------                                 i        i
  rnd-4_family-       6576 CGGCGGCGGCGGCGGCGGCGGTGCTTCGCGGCACGGTGGCGGGGGCGACG 6625

  contig10_pilo      85701 GCGCGTTTGGTGTGTGGAGCAGGAGCAGTGGCGGTGGGCGCGTGAGCGGG 85750
                                       i      vi     i            i          
  rnd-4_family-       6626 GCGCGTTTGGTGCGTGGAGGGGGAGCGGTGGCGGTGGGCACGTGAGCGGG 6675

  contig10_pilo      85751 AGCGCCGGTA 85760
                                     
  rnd-4_family-       6676 AGCGCCGGTA 6685

Matrix = 20p53g.matrix
Kimura (with divCpGMod) = 1.08
Transitions / transversions = 2.00 (20/10)
Gap_init rate = 0.00 (2 / 1450), avg. gap size = 4.50 (9 / 2)

 
 

   +    4508    1.6  0.0  0.0  contig10_pilon    85830   86384 (1728495) +  rnd-4_family-867   Unknown           6869   7423  (9034)    38     
 
 ANNOTATION EVIDENCE: 
  4508   1.62 0.00 0.00  contig10_pilon    85830   86384   1728495 +  rnd-4_family-867   Unknown           6869   7423    9034      
4508 1.62 0.00 0.00 contig10_pilon 85830 86384 (1728495) rnd-4_family-867#Unknown 6869 7423 (9034) m_b496s001i23

  contig10_pilo      85830 TCGCCAGGTAGTATCTCCGGGATTCGGGTTCGCGGTTGGAGGATAGGATC 85879
                                                          v                  
  rnd-4_family-       6869 TCGCCAGGTAGTATCTCCGGGATTCGGGTTCTCGGTTGGAGGATAGGATC 6918

  contig10_pilo      85880 TGGCAGCTCGGAGCCGCTGTACGGCCTGCGCACAAGCCGGAGCTGTACAA 85929
                                       v                                   i 
  rnd-4_family-       6919 TGGCAGCTCGGATCCGCTGTACGGCCTGCGCACAAGCCGGAGCTGTACGA 6968

  contig10_pilo      85930 CGATGGACATGCCGTCACAGTCATGGCCATCCCGGCCCATGTCCATATCG 85979
                                                                             
  rnd-4_family-       6969 CGATGGACATGCCGTCACAGTCATGGCCATCCCGGCCCATGTCCATATCG 7018

  contig10_pilo      85980 TCGCCTTCCTTCAGGTGCGCATAGCGAGGAATATCCTCACCGTAACTTGG 86029
                                                                             
  rnd-4_family-       7019 TCGCCTTCCTTCAGGTGCGCATAGCGAGGAATATCCTCACCGTAACTTGG 7068

  contig10_pilo      86030 GTGCACCTGCAACACCTCGACGAACAAGTTTTTCTCCCAGTCCTTCAGGA 86079
                                                                     v       
  rnd-4_family-       7069 GTGCACCTGCAACACCTCGACGAACAAGTTTTTCTCCCAGTCGTTCAGGA 7118

  contig10_pilo      86080 GAGAGAGCACGGTGGGCGGCAGCTCGTCGTAGCTCAACCACCGGTTGAAG 86129
                                                                             
  rnd-4_family-       7119 GAGAGAGCACGGTGGGCGGCAGCTCGTCGTAGCTCAACCACCGGTTGAAG 7168

  contig10_pilo      86130 CGCGGCGTGTTCACGATGATCGTCTCGCCAGTATGATTGACTTTAGTCTC 86179
                                                           v                 
  rnd-4_family-       7169 CGCGGCGTGTTCACGATGATCGTCTCGCCAGTCTGATTGACTTTAGTCTC 7218

  contig10_pilo      86180 GTGCAACATAAGAAAAGCGCGGCGGATAATACGCACCACCATCTGACAAT 86229
                            i                                                
  rnd-4_family-       7219 GCGCAACATAAGAAAAGCGCGGCGGATAATACGCACCACCATCTGACAAT 7268

  contig10_pilo      86230 AAAACTGCCACTCGCCGTTGGGGCGGGTCGGTTTGTCCGGTAGAGAGTAG 86279
                                                                             
  rnd-4_family-       7269 AAAACTGCCACTCGCCGTTGGGGCGGGTCGGTTTGTCCGGTAGAGAGTAG 7318

  contig10_pilo      86280 AGGGGAAGCTTGGGGGGCATGGAAAGAGGATTAAAGAGCGGGGTAATGTC 86329
                             v                                               
  rnd-4_family-       7319 AGCGGAAGCTTGGGGGGCATGGAAAGAGGATTAAAGAGCGGGGTAATGTC 7368

  contig10_pilo      86330 AAGTCTGTAACTGCGAGACCCAGACGTTTAAGTACGTCACTGCGCGGTCC 86379
                                                     i           i           
  rnd-4_family-       7369 AAGTCTGTAACTGCGAGACCCAGACGCTTAAGTACGTCGCTGCGCGGTCC 7418

  contig10_pilo      86380 CACGA 86384
                                
  rnd-4_family-       7419 CACGA 7423

Matrix = 20p53g.matrix
Kimura (with divCpGMod) = 1.14
Transitions / transversions = 0.80 (4/5)
Gap_init rate = 0.00 (0 / 554), avg. gap size = 0.0 (0 / 0)

 
 
 
  +    4254    3.1  0.0  0.2  contig10_pilon    86384   86924 (1727955) +  rnd-4_family-867   Unknown           9564  10103  (6354)    38 *   
 
 ANNOTATION EVIDENCE: 
  4254   3.15 0.00 0.19  contig10_pilon    86384   86924   1727955 +  rnd-4_family-867   Unknown           9564  10103    6354      
4254 3.15 0.00 0.19 contig10_pilon 86384 86924 (1727955) rnd-4_family-867#Unknown 9564 10103 (6354) m_b496s001i24

  contig10_pilo      86384 AAATAGCACCTGATGCTCTTCAGGGAAGGGATCTTCCAATGCTCAAGCAT 86433
                                                                       i     
  rnd-4_family-       9564 AAATAGCACCTGATGCTCTTCAGGGAAGGGATCTTCCAATGCTCGAGCAT 9613

  contig10_pilo      86434 ATCTTCTGACTGTGGGCGCAACACTGATTTTGTCGGGGGGGATGGATGCG 86483
                                                         i        i-         
  rnd-4_family-       9614 ATCTTCTGACTGTGGGCGCAACACTGATTTCGTCGGGGGA-ATGGATGCG 9662

  contig10_pilo      86484 AGGAACGGGAATGAGATGTCCAGGGACACCAAGGAGGGTATATTGACGCA 86533
                                                      i                      
  rnd-4_family-       9663 AGGAACGGGAATGAGATGTCCAGGGACGCCAAGGAGGGTATATTGACGCA 9712

  contig10_pilo      86534 TATACCCTCCTTTGACCGCCCACGCTCACTGACGCGTGAGCGTGTATGTG 86583
                                                                           i 
  rnd-4_family-       9713 TATACCCTCCTTTGACCGCCCACGCTCACTGACGCGTGAGCGTGTATGCG 9762

  contig10_pilo      86584 GGTGGTGTTCTTGTGCGAGCCGGCGATAGCGTCGAGGGGAATGGATGCGA 86633
                                i  i                         i        v      
  rnd-4_family-       9763 GGTGGCGTCCTTGTGCGAGCCGGCGATAGCGTCGGGGGGAATGCATGCGA 9812

  contig10_pilo      86634 GAAACGGGAAGAAGATGTCCAGGGACACCAAGGAGGGTATATTGACGCAT 86683
                                           i                                 
  rnd-4_family-       9813 GAAACGGGAAGAAGATATCCAGGGACACCAAGGAGGGTATATTGACGCAT 9862

  contig10_pilo      86684 ATACCCTCCTCTAATCGCCCACGCTCACTGACGCGTGAGCTTGTATGCGG 86733
                                       i                           v         
  rnd-4_family-       9863 ATACCCTCCTCTGATCGCCCACGCTCACTGACGCGTGAGCGTGTATGCGG 9912

  contig10_pilo      86734 GTGGCGTCCTTGGGCAAGCGGGCGACTGCATCTGGGGGTCTTGGGGGAAT 86783
                                          i             i                    
  rnd-4_family-       9913 GTGGCGTCCTTGGGCGAGCGGGCGACTGCGTCTGGGGGTCTTGGGGGAAT 9962

  contig10_pilo      86784 GGCTGCGAGGAACGACAAGAAAATGTACAGGAGCACTCTGAGGGACGCCA 86833
                                                                             
  rnd-4_family-       9963 GGCTGCGAGGAACGACAAGAAAATGTACAGGAGCACTCTGAGGGACGCCA 10012

  contig10_pilo      86834 AGGAGGATACATTGACGCATGTACCCTCCTCTGACCACCCACGCTCACTG 86883
                                                               i             
  rnd-4_family-      10013 AGGAGGATACATTGACGCATGTACCCTCCTCTGACCGCCCACGCTCACTG 10062

  contig10_pilo      86884 GTGCGTGAGCGTATATGCGGGTGGCCGTGCAGGTACGCCTA 86924
                            i          i                            
  rnd-4_family-      10063 GCGCGTGAGCGTGTATGCGGGTGGCCGTGCAGGTACGCCTA 10103

Matrix = 20p53g.matrix
Kimura (with divCpGMod) = 1.67
Transitions / transversions = 7.50 (15/2)
Gap_init rate = 0.00 (1 / 540), avg. gap size = 1.00 (1 / 1)

 
 

 
   +    4314    1.7  0.0  0.0  contig10_pilon    86923   87447 (1727432) C  rnd-4_family-3862  Unknown            (0)    525       1    39     
 
 ANNOTATION EVIDENCE: 
  4314   1.71 0.00 0.00  contig10_pilon    86923   87447   1727432 C  rnd-4_family-3862  Unknown              1    525       0      
4314 1.71 0.00 0.00 contig10_pilon 86923 87447 (1727432) C rnd-4_family-3862#Unknown (0) 525 1 m_b496s001i25

  contig10_pilo      86923 TACTCCCCGCCACGGAAAATAACCGCCGTTGGGAAATTCTCGATTTCTGC 86972
                                                                             
C rnd-4_family-        525 TACTCCCCGCCACGGAAAATAACCGCCGTTGGGAAATTCTCGATTTCTGC 476

  contig10_pilo      86973 CACAATTTTGATACACTCGACCTGAAACTTTTATTTGTGCATGATGCTTA 87022
                                                                             
C rnd-4_family-        475 CACAATTTTGATACACTCGACCTGAAACTTTTATTTGTGCATGATGCTTA 426

  contig10_pilo      87023 GGCTTGATACTATTCAATGGGAAACTCGGTTTGCGTTTTCCCCCATCCAG 87072
                                               v                             
C rnd-4_family-        425 GGCTTGATACTATTCAATGGTAAACTCGGTTTGCGTTTTCCCCCATCCAG 376

  contig10_pilo      87073 TAATTCCGGAGGGCAAGTCGGCTGGTGAACTGGAGGACAAGTTTGAGCTC 87122
                                                         i                   
C rnd-4_family-        375 TAATTCCGGAGGGCAAGTCGGCTGGTGAACCGGAGGACAAGTTTGAGCTC 326

  contig10_pilo      87123 ATATAAAAGCAAATATTGCGCGTACACCTTCGCTGCTTCTGCAACACAAC 87172
                                     i     i                            i    
C rnd-4_family-        325 ATATAAAAGCGAATATCGCGCGTACACCTTCGCTGCTTCTGCAACGCAAC 276

  contig10_pilo      87173 ATCCATACAAAATGAACCTGGGTTTCATATCAGGGGGAAGCTTGATGCTT 87222
                                                                            i
C rnd-4_family-        275 ATCCATACAAAATGAACCTGGGTTTCATATCAGGGGGAAGCTTGATGCTC 226

  contig10_pilo      87223 CCTGGTTGTGGAGATAGTATTAATATCGGTCCCCCTCAAAGATGACATCA 87272
                                                                             
C rnd-4_family-        225 CCTGGTTGTGGAGATAGTATTAATATCGGTCCCCCTCAAAGATGACATCA 176

  contig10_pilo      87273 TAGTCGAGTTGTATAGCACCCGTTTCAAGATCTCAGAGGAGGATTATGTA 87322
                                                                             
C rnd-4_family-        175 TAGTCGAGTTGTATAGCACCCGTTTCAAGATCTCAGAGGAGGATTATGTA 126

  contig10_pilo      87323 CCCAAGGACGTGCCCTACAACCCTTACTATTACACTTTATTTTTCTATGC 87372
                                                                             
C rnd-4_family-        125 CCCAAGGACGTGCCCTACAACCCTTACTATTACACTTTATTTTTCTATGC 76

  contig10_pilo      87373 AGATTGTAGAAAGAAAGGTGTGTCGCAAGTGGGCGGCTTTCTCCGAACGG 87422
                               i                                 v           
C rnd-4_family-         75 AGATCGTAGAAAGAAAGGTGTGTCGCAAGTGGGCGGCTATCTCCGAACGG 26

  contig10_pilo      87423 CGGTTATTTTCCGTGGCAGGGAGTA 87447
                                            i       
C rnd-4_family-         25 CGGTTATTTTCCGTGGCGGGGAGTA 1

Matrix = 20p53g.matrix
Kimura (with divCpGMod) = 0.69
Transitions / transversions = 3.50 (7/2)
Gap_init rate = 0.00 (0 / 524), avg. gap size = 0.0 (0 / 0)

 
 
 
 
  +    4103    2.0  0.2  1.5  contig10_pilon    87446   87990 (1726889) +  rnd-4_family-867   Unknown          10102  10639  (5818)    38 *   
 
 ANNOTATION EVIDENCE: 
  4103   2.05 0.18 1.49  contig10_pilon    87446   87990   1726889 +  rnd-4_family-867   Unknown          10102  10639    5818      
4103 2.05 0.18 1.49 contig10_pilon 87446 87990 (1726889) rnd-4_family-867#Unknown 10102 10639 (5818) m_b496s001i26

  contig10_pilo      87446 TAAGCGAAGAACCTGTCCCCATCATCTCGCCTATGAGGAGAACACCGAGA 87495
                                                                      i      
  rnd-4_family-      10102 TAAGCGAAGAACCTGTCCCCATCATCTCGCCTATGAGGAGAACGCCGAGA 10151

  contig10_pilo      87496 AGCCGCTCACTGACGCGTGAGCGGCCTCGTCACAGGCCCGCAGTTAAGCG 87545
                                                                             
  rnd-4_family-      10152 AGCCGCTCACTGACGCGTGAGCGGCCTCGTCACAGGCCCGCAGTTAAGCG 10201

  contig10_pilo      87546 GGCCTGGCCGTGCTGTCGGACGGACACTGACCCGTGGCGTCGACAGCAGC 87595
                                                                             
  rnd-4_family-      10202 GGCCTGGCCGTGCTGTCGGACGGACACTGACCCGTGGCGTCGACAGCAGC 10251

  contig10_pilo      87596 GGGCGGGCGGTGAGTCTGGGTCGGAGTTCGAACGGACTTTGGTCTTTCAT 87645
                                                                             
  rnd-4_family-      10252 GGGCGGGCGGTGAGTCTGGGTCGGAGTTCGAACGGACTTTGGTCTTTCAT 10301

  contig10_pilo      87646 CCGCCCTCCCC-TCCTCCGCACACCCACATCCCCGTCACCCACCCACCTT 87694
                                      -              v                  i    
  rnd-4_family-      10302 CCGCCCTCCCCCTCCTCCGCACACCCCCATCCCCGTCACCCACCCGCCTT 10351

  contig10_pilo      87695 CGCCCGCCACCCCGCCTTCCCCCTTCCCTCCTCCCTCCCTCTGCTCCGCC 87744
                                                                    i        
  rnd-4_family-      10352 CGCCCGCCACCCCGCCTTCCCCCTTCCCTCCTCCCTCCCTCCGCTCCGCC 10401

  contig10_pilo      87745 GGTGGCAACGCGTCAGTGCGTCAGTTGCCATCCAGAGCTCAGGTCGTTGG 87794
                                            --------                         
  rnd-4_family-      10402 GGTGGCAACGCGTCAGT--------TGCCATCCAGAGCTCAGGTCGTTGG 10443

  contig10_pilo      87795 TGAGCGTGGACACGCGTCAGTGTCTGCGCTCTCTTCGGCCGTCCCTCCTG 87844
                                                                             
  rnd-4_family-      10444 TGAGCGTGGACACGCGTCAGTGTCTGCGCTCTCTTCGGCCGTCCCTCCTG 10493

  contig10_pilo      87845 CCCCACTGAATACATTGTATAACCCCCTAGTGCCAGGCATACATTTTCTT 87894
                                          i                         v        
  rnd-4_family-      10494 CCCCACTGAATACATCGTATAACCCCCTAGTGCCAGGCATAAATTTTCTT 10543

  contig10_pilo      87895 TATACTTTACCTTTGCCCATTCCCCTCCGTGGCAGTCAACACATCAGTTG 87944
                                 i                         i       i i       
  rnd-4_family-      10544 TATACTCTACCTTTGCCCATTCCCCTCCGTGGTAGTCAACGCGTCAGTTG 10593

  contig10_pilo      87945 TCTGCCCGGAGAATCGTCGAGCACGCGTCAGTGTCCGTACGTCCCA 87990
                                                                    i    
  rnd-4_family-      10594 TCTGCCCGGAGAATCGTCGAGCACGCGTCAGTGTCCGTACGCCCCA 10639

Matrix = 20p53g.matrix
Kimura (with divCpGMod) = 1.05
Transitions / transversions = 4.50 (9/2)
Gap_init rate = 0.02 (9 / 544), avg. gap size = 1.00 (9 / 9)

 
 

 
 
   +    6894    9.1  0.7  1.2  contig10_pilon    88001   89046 (1725833) +  rnd-4_family-867   Unknown          12998  14037  (2420)    40     
 
 ANNOTATION EVIDENCE: 
  6894   9.10 0.67 1.25  contig10_pilon    88001   89046   1725833 +  rnd-4_family-867   Unknown          12998  14037    2420      
6894 9.10 0.67 1.25 contig10_pilon 88001 89046 (1725833) rnd-4_family-867#Unknown 12998 14037 (2420) m_b496s001i27

  contig10_pilo      88001 CAAGGACGGCAACACGGAGGGCATCCTGCCGCTACTCTCACTCGTTCCCG 88050
                                     i  i                   v                
  rnd-4_family-      12998 CAAGGACGGCGACGCGGAGGGCATCCTGCCGCTTCTCTCACTCGTTCCCG 13047

  contig10_pilo      88051 CACTCGCTGTCGTTGCTCGGTATGAAGTGAAGACAACGACACAAATCAGC 88100
                            v     i     i                    i    v          
  rnd-4_family-      13048 CCCTCGCCGTCGTCGCTCGGTATGAAGTGAAGACGACGAAACAAATCAGC 13097

  contig10_pilo      88101 TCTACACTTCGAAGCGTCGCGGGATTCGTTCTTGACCGTTCTCAAACGGT 88150
                                     i i                         v  v        
  rnd-4_family-      13098 TCTACACTTCAAGGCGTCGCGGGATTCGTTCTTGACCGATCGCAAACGGT 13147

  contig10_pilo      88151 CTTTGCGTCGCTTTCGTACCACGACCCGGATGAACACCTGATCGAAGAGC 88200
                              i            i                                 
  rnd-4_family-      13148 CTTCGCGTCGCTTTCGCACCACGACCCGGATGAACACCTGATCGAAGAGC 13197

  contig10_pilo      88201 ACGAGAATTGGCGCGAGGTTCGTTTTTATAATGATCCCGGACAGTGACAA 88250
                            i   i                                            
  rnd-4_family-      13198 ATGAGGATTGGCGCGAGGTTCGTTTTTATAATGATCCCGGACAGTGACAA 13247

  contig10_pilo      88251 AGTTAACACGCATTTCAGACTGGCACCTTCTATGGACAGCCTCGGATTCG 88300
                                                           i    i   i  v     
  rnd-4_family-      13248 AGTTAACACGCATTTCAGACTGGCACCTTCTACGGACGGCCCCGCATTCG 13297

  contig10_pilo      88301 AAACAGATCGATGTACCCCAACATTCCCGGAGACGGCGCAACTGATCGCG 88350
                                     i                               i       
  rnd-4_family-      13298 AAACAGATCGGTGTACCCCAACATTCCCGGAGACGGCGCAACCGATCGCG 13347

  contig10_pilo      88351 CGACGGTCACAGCGTCAGGTGGGAAGATGGCATGCTCCAAGTTTTATCAG 88400
                                     i     i  i           i                  
  rnd-4_family-      13348 CGACGGTCACGGCGTCGGGCGGGAAGATGGCGTGCTCCAAGTTTTATCAG 13397

  contig10_pilo      88401 ACCTATGGCAAGAAAGGCCTAACGGGCGGCTTGATGGCCATCTGGTGCCT 88450
                                             i       i     i                i
  rnd-4_family-      13398 ACCTATGGCAAGAAAGGCTTAACGGGTGGCTTAATGGCCATCTGGTGCCC 13447

  contig10_pilo      88451 GCACAGCGTCTGCTATGGGTTTCACTGCATCCCGAAAGGCGAGGGGCGGA 88500
                                 i        i                                  
  rnd-4_family-      13448 GCACAGTGTCTGCTACGGGTTTCACTGCATCCCGAAAGGCGAGGGGCGGA 13497

  contig10_pilo      88501 ACAACGTCTTTTCCGCGCTGTACACTCACTGGGTTCGGCCACCCAAAGTC 88550
                             i                               i               
  rnd-4_family-      13498 ACGACGTCTTTTCCGCGCTGTACACTCACTGGGTCCGGCCACCCAAAGTC 13547

  contig10_pilo      88551 GTTGTGTACGACTTCGCCTGCACGCTGTCCCCGTACTGCATGATTCGGGA 88600
                             i  i               i                            
  rnd-4_family-      13548 GTCGTATACGACTTCGCCTGCGCGCTGTCCCCGTACTGCATGATTCGGGA 13597

  contig10_pilo      88601 ACCCATCTACTTCGGCGACACCCTTTTCGTCATCGATGGCTTCCATGAGC 88650
                                                i     i        i             
  rnd-4_family-      13598 ACCCATCTACTTCGGCGACACTCTTTTTGTCATCGACGGCTTCCATGAGC 13647

  contig10_pilo      88651 ACGACCACTCCCGCTGCTCGCCGGCCTGTTTCCTCAGCGCGTATTTGGAA 88700
                                   v v  v        v  v                 ivii   
  rnd-4_family-      13648 ACGACCACACGCGGTGCTCGCCTGCGTGTTTCCTCAGCGCGTACACAGAA 13697

  contig10_pilo      88701 TGGTCTGAAAAGTATCGGAACATCAACTCGAGCGCCAGCGACTGCGGGAA 88750
                               v            v           v     vi    v  i     
  rnd-4_family-      13698 TGGTGTGAAAAGTATCGCAACATCAACTCCAGCGCGGGCGAATGTGGGAA 13747

  contig10_pilo      88751 CTCCAGCCTGCTCAAGATCCGCAAGTCGCTCAGTTACATGGGCCAACGCC 88800
                                 i                                         v 
  rnd-4_family-      13748 CTCCAGTCTGCTCAAGATCCGCAAGTCGCTCAGTTACATGGGCCAACGGC 13797

  contig10_pilo      88801 ACGGTATAGTCCTTGTCTATACATTCCTTGCCATTTGGAACCGCATGCGG 88850
                               i  v  i  i  v  i  i                           
  rnd-4_family-      13798 ACGGCATCGTTCTCGTGTACACGTTCCTTGCCATTTGGAACCGCATGCGG 13847

  contig10_pilo      88851 CAGAAATCGCGAGAGGCAGATGCTTAATCACGTGGCTTGAGACCCTGTCG 88900
                                              v  i                           
  rnd-4_family-      13848 CAGAAATCGCGAGAGGCAGCTGTTTAATCACGTGGCTTGAGACCCTGTCG 13897

  contig10_pilo      88901 ATCCGGCGATCTTAGATAATCCTG-ATAATCCCAAGTG-CTGAACCCCAA 88948
                              v       v            -             -      ii --
  rnd-4_family-      13898 ATCGGGCGATCATAGATAATCCTGTATAATCCCAAGTGCCTGAACTTC-- 13945

  contig10_pilo      88949 AGGGGACGGACTTGCATTACTGGTGTGTCGTGATCGTCCAAT-TCTCTGT 88997
                           ------  --ii             v   ---  iv viv  -   v   
  rnd-4_family-      13946 ------CG--TCTGCATTACTGGTGAGTC---ATTTTATTATGTCTGTGT 13984

  contig10_pilo      88998 G--TAATATCTCATGCAAATATACC-TCGCAGTAAACGTCATGATA-ACC 89043
                            --i     v v iiv  vii   v-             iv i   -   
  rnd-4_family-      13985 GCTCAATATATAACAAAACCGTACAGTCGCAGTAAACGTTTTAATAGACC 14034

  contig10_pilo      89044 ATG 89046
                           i  
  rnd-4_family-      14035 GTG 14037

Matrix = 20p53g.matrix
Kimura (with divCpGMod) = 7.46
Transitions / transversions = 1.76 (60/34)
Gap_init rate = 0.02 (19 / 1045), avg. gap size = 1.05 (20 / 19)

 
 
 
 
  +      13   18.4  6.6  4.8  contig10_pilon    90059   90119 (1724760) +  (CGTCC)n           Simple_repeat        1     62     (0)    41     
 
 ANNOTATION EVIDENCE: 
    13  18.41 6.56 4.84  contig10_pilon    90059   90119   1724760 +  (CGTCC)n           Simple_repeat        1     62       0      
13 18.41 6.56 4.84 contig10_pilon 90059 90119 (1724760) (CGTCC)n#Simple_repeat 1 62 (0) m_b496s252i0

  contig10_pilo      90059 CGTCTCG-CCCGTCTCACGCACGCGCGCCGTCCCG-CCCGTTCC-ACCCG 90105
                               i  -      - -  -v v  iv        -     i  -v    
  (CGTCC)n#Simp          1 CGTCCCGTCCCGTC-C-CG-TCCCGTCCCGTCCCGTCCCGTCCCGTCCCG 47

  contig10_pilo      90106 TTCCG-CCCGTCTCG 90119
                            i   -      i  
  (CGTCC)n#Simp         48 TCCCGTCCCGTCCCG 62

Matrix = Unknown
Transitions / transversions = 1.25 (5/4)
Gap_init rate = 0.12 (7 / 60), avg. gap size = 1.00 (7 / 7)

 
 

 
 
   +      12    8.6 13.5  0.0  contig10_pilon    90161   90197 (1724682) +  (CGTCT)n           Simple_repeat        1     42     (0)    42     
 
 ANNOTATION EVIDENCE: 
    12   8.58 13.51 0.00  contig10_pilon    90161   90197   1724682 +  (CGTCT)n           Simple_repeat        1     42       0      
12 8.58 13.51 0.00 contig10_pilon 90161 90197 (1724682) (CGTCT)n#Simple_repeat 1 42 (0) m_b496s252i1

  contig10_pilo      90161 CGTCTCG-CTCG-C-CGTCTCGCCT-GTC-CTGCTCGTCTCG 90197
                                  -    - -       i  -   - vv         
  (CGTCT)n#Simp          1 CGTCTCGTCTCGTCTCGTCTCGTCTCGTCTCGTCTCGTCTCG 42

Matrix = Unknown
Transitions / transversions = 0.50 (1/2)
Gap_init rate = 0.14 (5 / 36), avg. gap size = 1.00 (5 / 5)

 
 
 
 
  +     998   11.4  5.1  2.2  contig10_pilon    91016   91231 (1723648) +  rnd-4_family-867   Unknown          16236  16457     (0)    40     
 
 ANNOTATION EVIDENCE: 
   998  11.37 5.09 2.25  contig10_pilon    91016   91231   1723648 +  rnd-4_family-867   Unknown          16236  16457       0      
998 11.37 5.09 2.25 contig10_pilon 91016 91231 (1723648) rnd-4_family-867#Unknown 16236 16457 (0) m_b496s001i28

  contig10_pilo      91016 ACTGACTTAGGTAGACTTTTGGGGAGTT--GGGAGTACTGACTTAGATAG 91063
                                             vvv       --                    
  rnd-4_family-      16236 ACTGACTTAGGTAGACTTAGTGGGAGTTTGGGGAGTACTGACTTAGATAG 16285

  contig10_pilo      91064 ACTTACAGGG-TGGAGAAGCCCAGGAGTGGGTAAAAAGAGTCAATTGGAC 91112
                            i        -                    i    ?             
  rnd-4_family-      16286 ATTTACAGGGGTGGAGAAGCCCAGGAGTGGGCAAAANGAGTCAATTGGAC 16335

  contig10_pilo      91113 TCTGGGAGTTGTGGGAGTTGGGAGTGGGAATAT---GTACATAGGGAGT- 91158
                                                    iv  i v ---  v  ---     -
  rnd-4_family-      16336 TCTGGGAGTTGTGGGAGTTGGGAGTACGAGTCTCGGGTTCA---GGAGTC 16382

  contig10_pilo      91159 AGGAGTAGAAAAATTCAGGA----GTATTCCTGAGAAAACCGGGAGCAGC 91204
                                i  vv   v      ---- v v i   i -- i i     i   
  rnd-4_family-      16383 AGGAGCAGTCAAAGTCAGGACCTGGGAGTTCTGGG--AGCTGGGAGTAGC 16430

  contig10_pilo      91205 AAACCAGAAAACCACTCAAGGCCATAA 91231
                                 i    vv              
  rnd-4_family-      16431 AAACCAAAAAAGAACTCAAGGCCATAA 16457

Matrix = 20p53g.matrix
Kimura (with divCpGMod) = 12.42
Transitions / transversions = 0.85 (11/13)
Gap_init rate = 0.05 (10 / 215), avg. gap size = 1.60 (16 / 10)

 
 

 
 
   +    6631    1.8  0.0  0.0  contig10_pilon    91349   92185 (1722694) +  rnd-4_family-1185  LINE/Tad1            1    837   (951)    43     
 
 ANNOTATION EVIDENCE: 
  6631   1.79 0.00 0.00  contig10_pilon    91349   92185   1722694 +  rnd-4_family-1185  LINE/Tad1            1    837     951      
6631 1.79 0.00 0.00 contig10_pilon 91349 92185 (1722694) rnd-4_family-1185#LINE/Tad1 1 837 (951) m_b496s001i29

  contig10_pilo      91349 AGCTTCTTGGACTCTTACCACCACCTGGTCAGAGTCAGTCTACCTCTGGC 91398
                                                                             
  rnd-4_family-          1 AGCTTCTTGGACTCTTACCACCACCTGGTCAGAGTCAGTCTACCTCTGGC 50

  contig10_pilo      91399 ACACTCTGTCCTCTACCTCTCGTCCTACTGACCCTCCTAGGGGCCCAGGG 91448
                                                         i                   
  rnd-4_family-         51 ACACTCTGTCCTCTACCTCTCGTCCTACTGGCCCTCCTAGGGGCCCAGGG 100

  contig10_pilo      91449 GGTCCCCCTGGCCCTCCAAAGAGTGGCTCCAGCCTGCTCATGGCTCCCCA 91498
                                                                             
  rnd-4_family-        101 GGTCCCCCTGGCCCTCCAAAGAGTGGCTCCAGCCTGCTCATGGCTCCCCA 150

  contig10_pilo      91499 GTGGGGCATGTCCTTGGATGCTCCAGCCCAACCTGCCCTGGCCACTCCAG 91548
                                  ?  i      i                                
  rnd-4_family-        151 GTGGGGCNTGCCCTTGGGTGCTCCAGCCCAACCTGCCCTGGCCACTCCAG 200

  contig10_pilo      91549 CCCCCACCACTGCCCCTGGCCCCACCTCTGGCCTTGGCCCTCCCTCTAGG 91598
                                                            i                
  rnd-4_family-        201 CCCCCACCACTGCCCCTGGCCCCACCTCTGGCCCTGGCCCTCCCTCTAGG 250

  contig10_pilo      91599 ACCACTCCCCCCTCCCTTGCCCCCCTTAAGCGGCCTGTAGGTGCTCTGGC 91648
                                                                             
  rnd-4_family-        251 ACCACTCCCCCCTCCCTTGCCCCCCTTAAGCGGCCTGTAGGTGCTCTGGC 300

  contig10_pilo      91649 TAGTCCTGGGCAACTTGCAGGTTCTGCTGGGTCTGCACCATCTCCTGGGC 91698
                                                                   i         
  rnd-4_family-        301 TAGTCCTGGGCAACTTGCAGGTTCTGCTGGGTCTGCACCACCTCCTGGGC 350

  contig10_pilo      91699 GCATTTCTGGGCCTAAAAAGTCCAGGAAAAGCAGTCCTATTCCCTCCACC 91748
                                                           i                 
  rnd-4_family-        351 GCATTTCTGGGCCTAAAAAGTCCAGGAAAAGCGGTCCTATTCCCTCCACC 400

  contig10_pilo      91749 ACCTCCCAGCACCTCCCACCCGAAAGGGCTAGCTCCCAGAGTGCCTGGTT 91798
                                                                  i          
  rnd-4_family-        401 ACCTCCCAGCACCTCCCACCCGAAAGGGCTAGCTCCCAGGGTGCCTGGTT 450

  contig10_pilo      91799 GAGCCCTGGTGAGGTGCTTCCTCAGGGGGCCGCGCGTTCACCGATTTCAA 91848
                                             v       i             i         
  rnd-4_family-        451 GAGCCCTGGTGAGGTGCTGCCTCAGGAGGCCGCGCGTTCATCGATTTCAA 500

  contig10_pilo      91849 ACTCCGCTCCGGATACTTCCGGACCCCCTCCTCCTGCCCCTGCTGCCTGG 91898
                                                                      i      
  rnd-4_family-        501 ACTCCGCTCCGGATACTTCCGGACCCCCTCCTCCTGCCCCTGCCGCCTGG 550

  contig10_pilo      91899 CAGGCTACCTTCAGCTTCAATCTGCCTGATACTTCGGCTGTGGGTGCCCC 91948
                                                            i                
  rnd-4_family-        551 CAGGCTACCTTCAGCTTCAATCTGCCTGATACTCCGGCTGTGGGTGCCCC 600

  contig10_pilo      91949 TAGGGATGCTCTGCCCTCTCAGCCAGTTGCTCCTGACCAGGACCAGGATA 91998
                                                                             
  rnd-4_family-        601 TAGGGATGCTCTGCCCTCTCAGCCAGTTGCTCCTGACCAGGACCAGGATA 650

  contig10_pilo      91999 TGGAGCCACAGAATGACTCTGAGGCTCTGGACCCTGATACACTTGCTGCT 92048
                           i                     v                           
  rnd-4_family-        651 CGGAGCCACAGAATGACTCTGATGCTCTGGACCCTGATACACTTGCTGCT 700

  contig10_pilo      92049 GAACTGGATGCCAAGCCTGTGACCTCCAGGAAGGCTAGGGACATTATTTC 92098
                                                                             
  rnd-4_family-        701 GAACTGGATGCCAAGCCTGTGACCTCCAGGAAGGCTAGGGACATTATTTC 750

  contig10_pilo      92099 CTTCCTGACCTACTGCCTGGACATGGTCAAGGTGATCTATGATCGCAAGG 92148
                                                                             
  rnd-4_family-        751 CTTCCTGACCTACTGCCTGGACATGGTCAAGGTGATCTATGATCGCAAGG 800

  contig10_pilo      92149 ACCGCTTTCCTAGTGCTGCTGGAGCTGCAGTTGAGGA 92185
                                                          i     
  rnd-4_family-        801 ACCGCTTTCCTAGTGCTGCTGGAGCTGCAGTCGAGGA 837

Matrix = 20p53g.matrix
Kimura (with divCpGMod) = 1.38
Transitions / transversions = 6.50 (13/2)
Gap_init rate = 0.00 (0 / 836), avg. gap size = 0.0 (0 / 0)

 
 
 
 
 
  +   10226    0.8  0.1  1.1  contig10_pilon    92188   93453 (1721426) C  rnd-4_family-2054  LINE/Tad1          (0)   1253       1    44     
 
 ANNOTATION EVIDENCE: 
 10226   0.80 0.08 1.12  contig10_pilon    92188   93453   1721426 C  rnd-4_family-2054  LINE/Tad1            1   1253       0      
10226 0.80 0.08 1.12 contig10_pilon 92188 93453 (1721426) C rnd-4_family-2054#LINE/Tad1 (0) 1253 1 m_b496s001i30

  contig10_pilo      92188 GAGAGAGAGAGAGAAATTCTTGAATTTTAGACAGCGTAAGCAGGCTACAA 92237
                                                                             
C rnd-4_family-       1253 GAGAGAGAGAGAGAAATTCTTGAATTTTAGACAGCGTAAGCAGGCTACAA 1204

  contig10_pilo      92238 ATACAGAGCCAAGACGCTGTGGTCTGTACAAAGTACATTCCAATGGGTCA 92287
                                                                             
C rnd-4_family-       1203 ATACAGAGCCAAGACGCTGTGGTCTGTACAAAGTACATTCCAATGGGTCA 1154

  contig10_pilo      92288 ACATGGGGCAGGCAAGGGGGG-TCGTAACAGGGGTGTAGAGGGATCTAGT 92336
                                                -                            
C rnd-4_family-       1153 ACATGGGGCAGGCAAGGGGGGGTCGTAACAGGGGTGTAGAGGGATCTAGT 1104

  contig10_pilo      92337 GCAAACTGGTGTGAATTCTGGGTGTAACAAAGTTGTGAGCGCAGTTCCAT 92386
                                        i                                    
C rnd-4_family-       1103 GCAAACTGGTGTGGATTCTGGGTGTAACAAAGTTGTGAGCGCAGTTCCAT 1054

  contig10_pilo      92387 AGGGGTATAGGGGCATAGGGGCATAGAATAAATAAAAATATACAAACATA 92436
                                                                             
C rnd-4_family-       1053 AGGGGTATAGGGGCATAGGGGCATAGAATAAATAAAAATATACAAACATA 1004

  contig10_pilo      92437 CCATAACTTCATGCGCCTCATCAGCACATGAAGTGACCAGCTCGCCAATA 92486
                                                                             
C rnd-4_family-       1003 CCATAACTTCATGCGCCTCATCAGCACATGAAGTGACCAGCTCGCCAATA 954

  contig10_pilo      92487 AGCAAGCAGATGTTAGCAAGTAAGCAGCTGCATGTGGCCCTTTAGGGCCA 92536
                                                                             
C rnd-4_family-        953 AGCAAGCAGATGTTAGCAAGTAAGCAGCTGCATGTGGCCCTTTAGGGCCA 904

  contig10_pilo      92537 TTGTGGGTGTATCTCTTGAGACACTCAAGAAAAGTATCTTAGAGACTGAT 92586
                           i                                                 
C rnd-4_family-        903 CTGTGGGTGTATCTCTTGAGACACTCAAGAAAAGTATCTTAGAGACTGAT 854

  contig10_pilo      92587 AGAGGGTAGAGGGCAGAGTCTGGATGGGTGGGGGTGTAGCCTACAGACAA 92636
                                                            v                
C rnd-4_family-        853 AGAGGGTAGAGGGCAGAGTCTGGATGGGTGGGGCTGTAGCCTACAGACAA 804

  contig10_pilo      92637 AGGCGCACCGCAACATGGTCAAAGCCATGTGGAGCCACGGCGCAGCTCAT 92686
                                                                             
C rnd-4_family-        803 AGGCGCACCGCAACATGGTCAAAGCCATGTGGAGCCACGGCGCAGCTCAT 754

  contig10_pilo      92687 GGTCCTCCCAACCCATTGTATGAATGGTTAAGGGTCCTGCGGCGGTGGCT 92736
                                         --------------                      
C rnd-4_family-        753 GGTCCTCCCAACCC--------------TAAGGGTCCTGCGGCGGTGGCT 718

  contig10_pilo      92737 CACCAGTTTTGGTGAACGCACCCGACGAGTGGATGAATTTGGCCAAGGCA 92786
                                                                             
C rnd-4_family-        717 CACCAGTTTTGGTGAACGCACCCGACGAGTGGATGAATTTGGCCAAGGCA 668

  contig10_pilo      92787 GAGATGCCCTTGCGCGTACTGAGTAGCGCATGCGTCCCAAGCAGGTCAGG 92836
                                                                      i      
C rnd-4_family-        667 GAGATGCCCTTGCGCGTACTGAGTAGCGCATGCGTCCCAAGCAAGTCAGG 618

  contig10_pilo      92837 CTTGAAGGAGGCAAAGTGGTGATAGTGCTCGTCATGGCGCTCGCAATCTC 92886
                                                                             
C rnd-4_family-        617 CTTGAAGGAGGCAAAGTGGTGATAGTGCTCGTCATGGCGCTCGCAATCTC 568

  contig10_pilo      92887 TAAGGATGTGATCGCGTGTCTGCAAGTGGTATCCACAAGGGCAAGTCACA 92936
                                                         i                   
C rnd-4_family-        567 TAAGGATGTGATCGCGTGTCTGCAAGTGGTGTCCACAAGGGCAAGTCACA 518

  contig10_pilo      92937 TCTTCAGTGGGAACATGGCGCTGATAGTACTCGCCCATGAACGCATGTCC 92986
                                                                             
C rnd-4_family-        517 TCTTCAGTGGGAACATGGCGCTGATAGTACTCGCCCATGAACGCATGTCC 468

  contig10_pilo      92987 AAGGCGGCACTGCATGACCAGTCCATAAAGACGGCGAGGCAGCTCACCAA 93036
                                                                             
C rnd-4_family-        467 AAGGCGGCACTGCATGACCAGTCCATAAAGACGGCGAGGCAGCTCACCAA 418

  contig10_pilo      93037 AGTGAGGTGGAGGGCGCCTCTTGGGTGGCCCATAGGCTGCCCTGGCATAC 93086
                                                                             
C rnd-4_family-        417 AGTGAGGTGGAGGGCGCCTCTTGGGTGGCCCATAGGCTGCCCTGGCATAC 368

  contig10_pilo      93087 AAACCTCGAGGAGGTGACTCTGCCCACTCCTCTATCCAAGTCTCCATGAC 93136
                                i                                            
C rnd-4_family-        367 AAACCCCGAGGAGGTGACTCTGCCCACTCCTCTATCCAAGTCTCCATGAC 318

  contig10_pilo      93137 ACGCTCCTTGGCGCGTCTTTTGAGGTGGGACACAGTGGCCCATGAGGGAC 93186
                                       i                                     
C rnd-4_family-        317 ACGCTCCTTGGCACGTCTTTTGAGGTGGGACACAGTGGCCCATGAGGGAC 268

  contig10_pilo      93187 CTTGCATGCGAGCTGCGTCCTTGGCTAACTCATCTGCACGCTCATTGCCC 93236
                                           v                                 
C rnd-4_family-        267 CTTGCATGCGAGCTGCCTCCTTGGCTAACTCATCTGCACGCTCATTGCCC 218

  contig10_pilo      93237 CTTATGCCAATGTGGCTGGGGACCCAAGAGATGGTCACCTCATGAGAAGC 93286
                                      i                                      
C rnd-4_family-        217 CTTATGCCAATATGGCTGGGGACCCAAGAGATGGTCACCTCATGAGAAGC 168

  contig10_pilo      93287 ATCAGCAGCAAGGAAGTCACGAGCATGCTCGCAGAAAGAAATGGAAAACT 93336
                                                                             
C rnd-4_family-        167 ATCAGCAGCAAGGAAGTCACGAGCATGCTCGCAGAAAGAAATGGAAAACT 118

  contig10_pilo      93337 GCTGGGCAGAAGCAGGACGCTCCTTGACAATGGCCTCAATAGCTGAAGAG 93386
                                                                  i          
C rnd-4_family-        117 GCTGGGCAGAAGCAGGACGCTCCTTGACAATGGCCTCAACAGCTGAAGAG 68

  contig10_pilo      93387 TTGTCAGCAAAGAAGTGAAGGTGGGAGGCCGAGCCCATGGGCAAGTCCAT 93436
                                                                             
C rnd-4_family-         67 TTGTCAGCAAAGAAGTGAAGGTGGGAGGCCGAGCCCATGGGCAAGTCCAT 18

  contig10_pilo      93437 GTAATGGGCAACATGGT 93453
                                            
C rnd-4_family-         17 GTAATGGGCAACATGGT 1

Matrix = 20p53g.matrix
Kimura (with divCpGMod) = 0.80
Transitions / transversions = 4.00 (8/2)
Gap_init rate = 0.01 (15 / 1265), avg. gap size = 1.00 (15 / 15)

 
 

 
 
   +   36588    2.1  0.2  0.0  contig10_pilon    93454   98007 (1716872) C  rnd-3_family-161   LINE/Tad1          (0)   4561       1    45     
 
 ANNOTATION EVIDENCE: 
 36588   2.06 0.20 0.04  contig10_pilon    93454   98007   1716872 C  rnd-3_family-161   LINE/Tad1            1   4561       0      
36588 2.06 0.20 0.04 contig10_pilon 93454 98007 (1716872) C rnd-3_family-161#LINE/Tad1 (0) 4561 1 m_b496s001i31

  contig10_pilo      93454 CCATGGCAGCAGAGAGGGCATGCATCTCAGCATCATAGACCTCAGCCTGG 93503
                                                                             
C rnd-3_family-       4561 CCATGGCAGCAGAGAGGGCATGCATCTCAGCATCATAGACCTCAGCCTGG 4512

  contig10_pilo      93504 GACCCCATAGGGATGCGACCGCAAGACTGCTCCTGACCCTCCAGGTAGGT 93553
                                                                             
C rnd-3_family-       4511 GACCCCATAGGGATGCGACCGCAAGACTGCTCCTGACCCTCCAGGTAGGT 4462

  contig10_pilo      93554 CACAAAAGCTGCACCAGTCCTGCGACCAGTTGGAGTGTCTAAAAGGGAAC 93603
                                                                             
C rnd-3_family-       4461 CACAAAAGCTGCACCAGTCCTGCGACCAGTTGGAGTGTCTAAAAGGGAAC 4412

  contig10_pilo      93604 CATCAGTGTAGATCAGCAGGTTTGCAGAGCTGAGTTCCAGCATCTGCACA 93653
                                                                             
C rnd-3_family-       4411 CATCAGTGTAGATCAGCAGGTTTGCAGAGCTGAGTTCCAGCATCTGCACA 4362

  contig10_pilo      93654 AGTCTGACATGGGACTCTGCAGCATCAGCTTTGCTCACACCCTTTGGCAC 93703
                                                                             
C rnd-3_family-       4361 AGTCTGACATGGGACTCTGCAGCATCAGCTTTGCTCACACCCTTTGGCAC 4312

  contig10_pilo      93704 AGGCCTGAGGTGGATGCGACCTGGAAAAGATGCAAGCAGAGAAACGGCCC 93753
                                                        v              i     
C rnd-3_family-       4311 AGGCCTGAGGTGGATGCGACCTGGAAAAGCTGCAAGCAGAGAAATGGCCC 4262

  contig10_pilo      93754 ATGGGGCAATGTGGAAGGGCTCAATGCGCTCATTGCCAGGTGAGGTGAGC 93803
                                                                             
C rnd-3_family-       4261 ATGGGGCAATGTGGAAGGGCTCAATGCGCTCATTGCCAGGTGAGGTGAGC 4212

  contig10_pilo      93804 TTGGCAAGGTAGCGAAGAGAGGAGCGAGTTTGACCACGCTTGTGAACCAC 93853
                                                                       v     
C rnd-3_family-       4211 TTGGCAAGGTAGCGAAGAGAGGAGCGAGTTTGACCACGCTTGTGCACCAC 4162

  contig10_pilo      93854 TGGTGGCTTCTCCATTGGAGACTGGTGCCAGGCAGGGTGCAGGCGCTGCA 93903
                                                                             
C rnd-3_family-       4161 TGGTGGCTTCTCCATTGGAGACTGGTGCCAGGCAGGGTGCAGGCGCTGCA 4112

  contig10_pilo      93904 AGACAGGACTGCAAGGCCCCAACTTGTGTAGGCGAGCAGCAGCAAGGTCA 93953
                                       i                                     
C rnd-3_family-       4111 AGACAGGACTGCGAGGCCCCAACTTGTGTAGGCGAGCAGCAGCAAGGTCA 4062

  contig10_pilo      93954 TCGGCCTTGTCTAATACCAAATGTATAGGTGGAATGGAGGCCTCCAACTC 94003
                                                              i vv           
C rnd-3_family-       4061 TCGGCCTTGTCTAATACCAAATGTATAGGTGGAATAGCTGCCTCCAACTC 4012

  contig10_pilo      94004 CAGAGCATAAATGGGCGAAGTTTTGAAGACAGCACAGATCATGCGCAGAC 94053
                                                i                            
C rnd-3_family-       4011 CAGAGCATAAATGGGCGAAGTCTTGAAGACAGCACAGATCATGCGCAGAC 3962

  contig10_pilo      94054 ACAAGTTCTGCACACGCATGAGCTTCTCAGCTTGCCATGCCCTTTGCATC 94103
                                                                             
C rnd-3_family-       3961 ACAAGTTCTGCACACGCATGAGCTTCTCAGCTTGCCATGCCCTTTGCATC 3912

  contig10_pilo      94104 CACCATACTGGTGAAGCATAGAGGAGGATGGGGAGAATGCAGGAGATGTA 94153
                                i                    i                       
C rnd-3_family-       3911 CACCACACTGGTGAAGCATAGAGGAGAATGGGGAGAATGCAGGAGATGTA 3862

  contig10_pilo      94154 CAGCCTGCGCATGTGTCCCTGGTGGAGACCACGCACAGTGTTGGCCAACA 94203
                                                                          i  
C rnd-3_family-       3861 CAGCCTGCGCATGTGTCCCTGGTGGAGACCACGCACAGTGTTGGCCAGCA 3812

  contig10_pilo      94204 TGCGCAATCCCATGGCTGCCTTGCGCGCCTTGGCACAGGCCTTATCCACA 94253
                                                                      i      
C rnd-3_family-       3811 TGCGCAATCCCATGGCTGCCTTGCGCGCCTTGGCACAGGCCTTGTCCACA 3762

  contig10_pilo      94254 TGTGCGCGATAGGTCAACTTGGGGTCAAAGAAGATACCCAGCCAGCGTAC 94303
                             v                                v              
C rnd-3_family-       3761 TGGGCGCGATAGGTCAACTTGGGGTCAAAGAAGATCCCCAGCCAGCGTAC 3712

  contig10_pilo      94304 AACTGGGAGAGGGCGCAGGCGAGCAACCTGGCCCTGAGCATCAGTGTAGG 94353
                                                                             
C rnd-3_family-       3711 AACTGGGAGAGGGCGCAGGCGAGCAACCTGGCCCTGAGCATCAGTGTAGG 3662

  contig10_pilo      94354 CCATGGCCAAGTCATCATTGGCAGCAATGTCAGCAAAGGAGCCATCCTTG 94403
                                                                             
C rnd-3_family-       3661 CCATGGCCAAGTCATCATTGGCAGCAATGTCAGCAAAGGAGCCATCCTTG 3612

  contig10_pilo      94404 CGACGCCAGGTGTGGTGCATAAGCTCACACTTGTCCAGGTCAGGCTGAAG 94453
                                                                             
C rnd-3_family-       3611 CGACGCCAGGTGTGGTGCATAAGCTCACACTTGTCCAGGTCAGGCTGAAG 3562

  contig10_pilo      94454 ACCTGCCAGGTTGAACCACTCCCGAATAATGCAGAAGCAGAAGCGCAGCT 94503
                                                                             
C rnd-3_family-       3561 ACCTGCCAGGTTGAACCACTCCCGAATAATGCAGAAGCAGAAGCGCAGCT 3512

  contig10_pilo      94504 CAGCAGTGTTCAGCTCGAGGCTGTCAGAGTGCACAGTGCATCTCCCATCA 94553
                                           v                                 
C rnd-3_family-       3511 CAGCAGTGTTCAGCTCCAGGCTGTCAGAGTGCACAGTGCATCTCCCATCA 3462

  contig10_pilo      94554 TCCACATAGAGGAATAGGGTGGGTGCCACAAATGTGGGATTGAGCTGGCG 94603
                                         v  v                                
C rnd-3_family-       3461 TCCACATAGAGGAAGAGCGTGGGTGCCACAAATGTGGGATTGAGCTGGCG 3412

  contig10_pilo      94604 CATCTCTGCCTCGCGAGCAGCTCGCCTCTGCTGCATGAACTCAATGAGCC 94653
                                          i                                  
C rnd-3_family-       3411 CATCTCTGCCTCGCGGGCAGCTCGCCTCTGCTGCATGAACTCAATGAGCC 3362

  contig10_pilo      94654 CAGTAGAGTAGAAGGCAGAGAGGATGCCAGAGACTGGAGAGCCCTGAGGC 94703
                                                                             
C rnd-3_family-       3361 CAGTAGAGTAGAAGGCAGAGAGGATGCCAGAGACTGGAGAGCCCTGAGGC 3312

  contig10_pilo      94704 ACTCCATTCTCAACTGGCTTACAGCTGCTGACTCTACCATCCAGGCATAT 94753
                                                                          v  
C rnd-3_family-       3311 ACTCCATTCTCAACTGGCTTACAGCTGCTGACTCTACCATCCAGGCAGAT 3262

  contig10_pilo      94754 GGATGCCTCTCTGTCCTGCAAGAAGGATGCAACCCACTGCACAATGGGCA 94803
                                                                             
C rnd-3_family-       3261 GGATGCCTCTCTGTCCTGCAAGAAGGATGCAACCCACTGCACAATGGGCA 3212

  contig10_pilo      94804 GAGGCACACGCTTCTCTCGCAAGACCAGCAGGAGACGCTGGTGGTTGACA 94853
                                                i         v  i               
C rnd-3_family-       3211 GAGGCACACGCTTCTCTCGCAGGACCAGCAGCAGGCGCTGGTGGTTGACA 3162

  contig10_pilo      94854 AAGTCAAAGTAACCCTTGATGTCAAAGGTCAGGGAACTAGTCACCAAACC 94903
                                i     i                    v  i              
C rnd-3_family-       3161 AAGTCGAAGTAGCCCTTGATGTCAAAGGTCAGTGAGCTAGTCACCAAACC 3112

  contig10_pilo      94904 CTTGTCACGAGCAGTCTGCACATCATGAGTGTAGCACAGAGCAGCATCCA 94953
                                                                             
C rnd-3_family-       3111 CTTGTCACGAGCAGTCTGCACATCATGAGTGTAGCACAGAGCAGCATCCA 3062

  contig10_pilo      94954 CAGTGGAGGACGCAGGCATGGCCC--------TTGGGGTGCACAAGACCC 94995
                                                   --------                  
C rnd-3_family-       3061 CAGTGGAGGACGCAGGCATGGCCCCAAACTGGTTGGGGTGCACAAGACCC 3012

  contig10_pilo      94996 ATGGCAGATGCTATGTAGGTCAAGCGCTTTGCCTGAATGCGCTCCAAGAC 95045
                                      i                                      
C rnd-3_family-       3011 ATGGCAGATGCCATGTAGGTCAAGCGCTTTGCCTGAATGCGCTCCAAGAC 2962

  contig10_pilo      95046 TTTTCCCAAGCAGACAAGCAAGGTAATCAGTCTGTAAGACCTTGGATTAG 95095
                                                         i                   
C rnd-3_family-       2961 TTTTCCCAAGCAGACAAGCAAGGTAATCAGCCTGTAAGACCTTGGATTAG 2912

  contig10_pilo      95096 ACCAATCAGTCTTGCCTGCTTTCTTGAGCACATATGCAATGGCTCGTCTC 95145
                                           v                                 
C rnd-3_family-       2911 ACCAATCAGTCTTGCCAGCTTTCTTGAGCACATATGCAATGGCTCGTCTC 2862

  contig10_pilo      95146 CAGCGACGAGGGTGGTAGCCCCAAAGAGCACAGCGCGCAATGAGAAGATA 95195
                                i                                            
C rnd-3_family-       2861 CAGCGGCGAGGGTGGTAGCCCCAAAGAGCACAGCGCGCAATGAGAAGATA 2812

  contig10_pilo      95196 AATCTCCTCCTGTGCCACATCCCAAGCCCAGCGCAACCCAGTGTAGGGAA 95245
                           i                                                 
C rnd-3_family-       2811 GATCTCCTCCTGTGCCACATCCCAAGCCCAGCGCAACCCAGTGTAGGGAA 2762

  contig10_pilo      95246 CTCCCTCATCACCAGGCGCTTTCTGGGGGTTAGGTCCAAAGATGCCTTCT 95295
                                                             i               
C rnd-3_family-       2761 CTCCCTCATCACCAGGCGCTTTCTGGGGGTTAGGCCCAAAGATGCCTTCT 2712

  contig10_pilo      95296 CTCACCTCCTCCAGAGTGACCTCCCTGTAGGGAATGTCTTCTGGGTGTGG 95345
                                                                             
C rnd-3_family-       2711 CTCACCTCCTCCAGAGTGACCTCCCTGTAGGGAATGTCTTCTGGGTGTGG 2662

  contig10_pilo      95346 TGTGGTCAGGTCTGGCTCGTCCAGGCCCTCTATGTGAGGAGGGCGCTGGT 95395
                                            v                                
C rnd-3_family-       2661 TGTGGTCAGGTCTGGCTGGTCCAGGCCCTCTATGTGAGGAGGGCGCTGGT 2612

  contig10_pilo      95396 ACAAGGTCTCGCGCAGTGCATCACACTTCTCAGCATGAGTGCGCGCAAAG 95445
                                                       i                     
C rnd-3_family-       2611 ACAAGGTCTCGCGCAGTGCATCACACTTTTCAGCATGAGTGCGCGCAAAG 2562

  contig10_pilo      95446 GTACCATCTGGGCGACGAATGTCAGGAGAACGGTACCCCCGACGTCCCTG 95495
                             i v               i                         --  
C rnd-3_family-       2561 GTGCAATCTGGGCGACGAATATCAGGAGAACGGTACCCCCGACGTC--TG 2514

  contig10_pilo      95496 GGACCACTTGCGCATGTCCCAGATGTCATCCACAGTGGCATCCTCCACTA 95545
                                                                            i
C rnd-3_family-       2513 GGACCACTTGCGCATGTCCCAGATGTCATCCACAGTGGCATCCTCCACTG 2464

  contig10_pilo      95546 CCTTGTTCCAGTAGTCACGCTTGGTGCGCTTTACCTGGCGACGATGGTAG 95595
                                                                             
C rnd-3_family-       2463 CCTTGTTCCAGTAGTCACGCTTGGTGCGCTTTACCTGGCGACGATGGTAG 2414

  contig10_pilo      95596 TTGCGCTCCCTGGCTGCAGTTGCAGTCAGGATTGCATTGGAGCGGCGCAT 95645
                             i                   v        iv  v              
C rnd-3_family-       2413 TTACGCTCCCTGGCTGCAGTTGAAGTCAGGACAGCCTTGGAGCGGCGCAT 2364

  contig10_pilo      95646 GCGCCTTGCAAAGTCCTGTGCAGCTCTCTCTGCATCTCGCATCTTCTGCA 95695
                                                                             
C rnd-3_family-       2363 GCGCCTTGCAAAGTCCTGTGCAGCTCTCTCTGCATCTCGCATCTTCTGCA 2314

  contig10_pilo      95696 AGCTCTGGGTGAGTGCAGGAGTCCACCAGGGCTGAGAGTGCTTAGAGGGT 95745
                                                             v               
C rnd-3_family-       2313 AGCTCTGGGTGAGTGCAGGAGTCCACCAGGGCTGTGAGTGCTTAGAGGGT 2264

  contig10_pilo      95746 CTCCTCTTGGGCACCTTGGCCTGAGTGGCACCATTGAGAGCATCATGGAG 95795
                                      v                             i        
C rnd-3_family-       2263 CTCCTCTTGGGGACCTTGGCCTGAGTGGCACCATTGAGAGCGTCATGGAG 2214

  contig10_pilo      95796 TGCTGCTGCAGTGCGCTCTAGGAGCTCAGTGTTGCGAGTGTGCTTGTGAG 95845
                                                   i         v           v   
C rnd-3_family-       2213 TGCTGCTGCAGTGCGCTCTAGGAGTTCAGTGTTGAGAGTGTGCTTGGGAG 2164

  contig10_pilo      95846 CACTTTGCAGCTCTGCAAACACTGCTGCATGGCCATCCAGGCGAGAGCGA 95895
                                                                             
C rnd-3_family-       2163 CACTTTGCAGCTCTGCAAACACTGCTGCATGGCCATCCAGGCGAGAGCGA 2114

  contig10_pilo      95896 AACTCTGCCTTGAACTCGGCCTCATCTGCATGCTTGAAGTTGTAGCTGGC 95945
                                                  i                          
C rnd-3_family-       2113 AACTCTGCCTTGAACTCGGCCTCGTCTGCATGCTTGAAGTTGTAGCTGGC 2064

  contig10_pilo      95946 TCCAGAGCCAAGGTCCACATCCTCAGCACCCAGGTCAAAGACCCAGGAGA 95995
                                 i                       ?                   
C rnd-3_family-       2063 TCCAGAACCAAGGTCCACATCCTCAGCACCNAGGTCAAAGACCCAGGAGA 2014

  contig10_pilo      95996 CAGTGTGATGGTCAGAGAAGTCATGGACGTCGTCTGACACCGCAAAGTCT 96045
                                                          v                  
C rnd-3_family-       2013 CAGTGTGATGGTCAGAGAAGTCATGGACGTCTTCTGACACCGCAAAGTCT 1964

  contig10_pilo      96046 TGCAGCAGACCAAGTGCAGCCATTTGGTCATTCTGCCACACCAAGTCCAG 96095
                                                                             
C rnd-3_family-       1963 TGCAGCAGACCAAGTGCAGCCATTTGGTCATTCTGCCACACCAAGTCCAG 1914

  contig10_pilo      96096 AACAGAGGACTGAGAGTGGTTGTCCTTGCGCCAAGTGGCCACATCAGGAC 96145
                                                                             
C rnd-3_family-       1913 AACAGAGGACTGAGAGTGGTTGTCCTTGCGCCAAGTGGCCACATCAGGAC 1864

  contig10_pilo      96146 TGTTGAGCACAGAGAACCCATGCTCCTCCATCCACTCCAGCATCTGCTCA 96195
                                                                             
C rnd-3_family-       1863 TGTTGAGCACAGAGAACCCATGCTCCTCCATCCACTCCAGCATCTGCTCA 1814

  contig10_pilo      96196 CCCCCTCGAGAGGAGTTGGCCGCAAACAAATCCCACACATGGTGGTGTTC 96245
                                                        i                    
C rnd-3_family-       1813 CCCCCTCGAGAGGAGTTGGCCGCAAACAAGTCCCACACATGGTGGTGTTC 1764

  contig10_pilo      96246 ATTCGCATCCATGGCAATGGCGATTGGCATGTCTGGAAGGTCAATGAGCT 96295
                              i                 i                            
C rnd-3_family-       1763 ATTTGCATCCATGGCAATGGCAATTGGCATGTCTGGAAGGTCAATGAGCT 1714

  contig10_pilo      96296 TGAAGCGCGCAAAAGTGCGGCCAAGTCGCTCAGCCCCCTCAGGAGCGTCC 96345
                                                    v                        
C rnd-3_family-       1713 TGAAGCGCGCAAAAGTGCGGCCAAGGCGCTCAGCCCCCTCAGGAGCGTCC 1664

  contig10_pilo      96346 TCATTGTACATGAGGACAAGTAAGAACGCTGGACGCCTCCCTTGACGAAT 96395
                                                                             
C rnd-3_family-       1663 TCATTGTACATGAGGACAAGTAAGAACGCTGGACGCCTCCCTTGACGAAT 1614

  contig10_pilo      96396 CTCAAGGATGAGAAAGTCAAGATCTTGGGCAAGGTCATAGCGGTTAAATA 96445
                                 i                                           
C rnd-3_family-       1613 CTCAAGAATGAGAAAGTCAAGATCTTGGGCAAGGTCATAGCGGTTAAATA 1564

  contig10_pilo      96446 CTTCAAAGTCACGCCGCGTCGCAGACGCATAGGCAAAGACACGTGGCCTC 96495
                                                                             
C rnd-3_family-       1563 CTTCAAAGTCACGCCGCGTCGCAGACGCATAGGCAAAGACACGTGGCCTC 1514

  contig10_pilo      96496 TCAAGAGCAGGCACTACTTGAACTGGCAGGATGGGATACATGCCTGCTGG 96545
                                                                             
C rnd-3_family-       1513 TCAAGAGCAGGCACTACTTGAACTGGCAGGATGGGATACATGCCTGCTGG 1464

  contig10_pilo      96546 TTCCTTGACAGAGTAGTGTGGCTCGTCTGGTTCATCACCAACGCGCCACC 96595
                              i                                      v       
C rnd-3_family-       1463 TTCTTTGACAGAGTAGTGTGGCTCGTCTGGTTCATCACCAACTCGCCACC 1414

  contig10_pilo      96596 ACGGTTCAGTGACCAGATAAATATCAGATGCAAGAGGGGTCCCATAATGG 96645
                                                                             
C rnd-3_family-       1413 ACGGTTCAGTGACCAGATAAATATCAGATGCAAGAGGGGTCCCATAATGG 1364

  contig10_pilo      96646 GAGCCAGGAGTGAAGAGTGCAGACTGCGAGTCATTGAGACGTCGCACATT 96695
                                             i                               
C rnd-3_family-       1363 GAGCCAGGAGTGAAGAGTACAGACTGCGAGTCATTGAGACGTCGCACATT 1314

  contig10_pilo      96696 GGTCATGGAGACACGAATGGGGCGAGTGGTGTCTCTCATGCAGACTCAGT 96745
                                                                             
C rnd-3_family-       1313 GGTCATGGAGACACGAATGGGGCGAGTGGTGTCTCTCATGCAGACTCAGT 1264

  contig10_pilo      96746 GTCAGGTGCAGGTGTGCTGGGATTGTCACGAGTGCTGCTTGCAGTCGCTG 96795
                                 i              v                       i    
C rnd-3_family-       1263 GTCAGGCGCAGGTGTGCTGGGCTTGTCACGAGTGCTGCTTGCAGTTGCTG 1214

  contig10_pilo      96796 CTGCCTTGGCCGCGGCGTCTGCCTCTGAGCGAGAGTTAGTCCCCAGAGGG 96845
                                                                i            
C rnd-3_family-       1213 CTGCCTTGGCCGCGGCGTCTGCCTCTGAGCGAGAGTTGGTCCCCAGAGGG 1164

  contig10_pilo      96846 ACCGCATTGCTGCCAGAAACATTGGCCTTGGGGCCAAGTGCTGCTCTGCT 96895
                             v              v  i                    vi       
C rnd-3_family-       1163 ACGGCATTGCTGCCAGACACGTTGGCCTTGGGGCCAAGTGCAACTCTGCT 1114

  contig10_pilo      96896 CTTGGTGCGGCGCAGCTTGGGTGCAGTTGCAGCCTTGTTTGCATCATATC 96945
                                                i               vv        i  
C rnd-3_family-       1113 CTTGGTGCGGCGCAGCTTGGGCGCAGTTGCAGCCTTGGGTGCATCATGTC 1064

  contig10_pilo      96946 TGGGCTTGCGCAGCGCCATGCGACGCCGCTGCCAGATCTTGCACTGGGAG 96995
                                                                             
C rnd-3_family-       1063 TGGGCTTGCGCAGCGCCATGCGACGCCGCTGCCAGATCTTGCACTGGGAG 1014

  contig10_pilo      96996 ACGCGCACAACATGTGCCCCACCACAGTGAGGGCACTTGAGCTGAGTGTG 97045
                           v           i                                     
C rnd-3_family-       1013 TCGCGCACAACACGTGCCCCACCACAGTGAGGGCACTTGAGCTGAGTGTG 964

  contig10_pilo      97046 CTCGCAGTGAGTTCCAGGTGCATGTACAGAGCTGTAGAACTCGCACTCCA 97095
                                                    i                        
C rnd-3_family-        963 CTCGCAGTGAGTTCCAGGTGCATGTGCAGAGCTGTAGAACTCGCACTCCA 914

  contig10_pilo      97096 TCTGCTCGCAAGTGTGGTTGGCAGTGGTGTGGGTGCTGGCACTGCAGACT 97145
                                                                             
C rnd-3_family-        913 TCTGCTCGCAAGTGTGGTTGGCAGTGGTGTGGGTGCTGGCACTGCAGACT 864

  contig10_pilo      97146 CCACAGCGCATGGGCTTGTCGCAAATGCGAGCAGTGTGGTCAAGGCTGCA 97195
                                                                             
C rnd-3_family-        863 CCACAGCGCATGGGCTTGTCGCAAATGCGAGCAGTGTGGTCAAGGCTGCA 814

  contig10_pilo      97196 ACACCTGTCACAGGTGCGCAGTCGAGGCATGTCCTCAAAGTAGCGAACTG 97245
                                    i           v                            
C rnd-3_family-        813 ACACCTGTCGCAGGTGCGCAGACGAGGCATGTCCTCAAAGTAGCGAACTG 764

  contig10_pilo      97246 AGCAGAGGCGTCCAAAGACACTAACTGCATGCTCAACTGCATAGCGAGCG 97295
                                                       i                     
C rnd-3_family-        763 AGCAGAGGCGTCCAAAGACACTAACTGCGTGCTCAACTGCATAGCGAGCG 714

  contig10_pilo      97296 TCTGCCTCACTCAGGAATGCCAGAATGACAGAGCCTGAGCTCTTGCCAGC 97345
                                                                             
C rnd-3_family-        713 TCTGCCTCACTCAGGAATGCCAGAATGACAGAGCCTGAGCTCTTGCCAGC 664

  contig10_pilo      97346 AAGGTCTGCTGCACTTCCCAGCCAGCGTGGCATGGTGGCCCAGTCAAACT 97395
                                                                             
C rnd-3_family-        663 AAGGTCTGCTGCACTTCCCAGCCAGCGTGGCATGGTGGCCCAGTCAAACT 614

  contig10_pilo      97396 TGTGAGTGTTGAACTCAACAATGTCTGAGAGGAGGTCTTGCCCAGTGGGC 97445
                                                                             
C rnd-3_family-        613 TGTGAGTGTTGAACTCAACAATGTCTGAGAGGAGGTCTTGCCCAGTGGGC 564

  contig10_pilo      97446 AGTCCCTCAATGTCATGAACTCGAGTCGAGACACCATTGACTAGCACTTG 97495
                                i           i            i                   
C rnd-3_family-        563 AGTCCTTCAATGTCATGGACTCGAGTCGAGGCACCATTGACTAGCACTTG 514

  contig10_pilo      97496 GTGCCAAGGTTCGTCGCGAGACGCCTTGATGAATGAGCCAGAGTCTGGCA 97545
                                    i                                        
C rnd-3_family-        513 GTGCCAAGGCTCGTCGCGAGACGCCTTGATGAATGAGCCAGAGTCTGGCA 464

  contig10_pilo      97546 CGATGGCAGACGCAATTGCATCTCCATGTTCAACCAGATCTGCTGAAGTC 97595
                                                                i            
C rnd-3_family-        463 CGATGGCAGACGCAATTGCATCTCCATGTTCAACCAGGTCTGCTGAAGTC 414

  contig10_pilo      97596 AGTCCATCTGCCGCAACCACCACAATGTTGCCAGAGGCGGTGGTGTCCAC 97645
                                                                 i           
C rnd-3_family-        413 AGTCCATCTGCCGCAACCACCACAATGTTGCCAGAGGCAGTGGTGTCCAC 364

  contig10_pilo      97646 ACTTGTGACACGAAGTGGGAGCTTGGCAGGCGACTTGCTCTTGTGAGGAG 97695
                                                                             
C rnd-3_family-        363 ACTTGTGACACGAAGTGGGAGCTTGGCAGGCGACTTGCTCTTGTGAGGAG 314

  contig10_pilo      97696 CAAGCGCAGTGTTCACTGCGGCGCGCAAGCGGCTTGCATTGTTGCGCACA 97745
                                                             i               
C rnd-3_family-        313 CAAGCGCAGTGTTCACTGCGGCGCGCAAGCGGCTCGCATTGTTGCGCACA 264

  contig10_pilo      97746 TGAGCAGTGAGCTCAGGGGTGGCACGGACGCTCAGGATGAGACGACCTGG 97795
                                                              i              
C rnd-3_family-        263 TGAGCAGTGAGCTCAGGGGTGGCACGGACGCTCAGAATGAGACGACCTGG 214

  contig10_pilo      97796 GTGGTGGCGCGCACGACCATTGGTAGGAGGTGGCTTTGGCACCGGACTGG 97845
                                                                       v     
C rnd-3_family-        213 GTGGTGGCGCGCACGACCATTGGTAGGAGGTGGCTTTGGCACCGCACTGG 164

  contig10_pilo      97846 GACGGGCAGGCACCACAGTGCCTTTGGCAGCTGGCTGAGCAGCACGCGAC 97895
                                                                             
C rnd-3_family-        163 GACGGGCAGGCACCACAGTGCCTTTGGCAGCTGGCTGAGCAGCACGCGAC 114

  contig10_pilo      97896 GCATAGCAGCGGCCAGGTGCGCTTGCCTTTGCAGGAGCAG-CTGGAGGAG 97944
                                 v          i                      -   vi    
C rnd-3_family-        113 GCATAGGAGCGGCCAGGCGCGCTTGCCTTTGCAGGAGCAGCCTGTGGGAG 64

  contig10_pilo      97945 CTGCTACTGCTGCAGGCTGCTGCTGTTGGGCACTTGAAGGCGCAGAGGCC 97994
                            v   i                                            
C rnd-3_family-         63 CAGCTGCTGCTGCAGGCTGCTGCTGTTGGGCACTTGAAGGCGCAGAGGCC 14

  contig10_pilo      97995 TGTTGGGCAGTGA 98007
                                        
C rnd-3_family-         13 TGTTGGGCAGTGA 1

Matrix = 20p53g.matrix
Kimura (with divCpGMod) = 1.85
Transitions / transversions = 1.54 (57/37)
Gap_init rate = 0.00 (4 / 4553), avg. gap size = 2.75 (11 / 4)

 
 
 
 
 
  +   14114    0.9  0.0  0.0  contig10_pilon    98009   99745 (1715134) C  rnd-4_family-2631  Unknown            (0)   1737       1    46     
 
 ANNOTATION EVIDENCE: 
 14114   0.92 0.00 0.00  contig10_pilon    98009   99745   1715134 C  rnd-4_family-2631  Unknown              1   1737       0      
14114 0.92 0.00 0.00 contig10_pilon 98009 99745 (1715134) C rnd-4_family-2631#Unknown (0) 1737 1 m_b496s001i32

  contig10_pilo      98009 GAAGGCAATGTTGCGCTTGACCTCGGCCATATCCGAGACAAGGGTGTCAA 98058
                                                                             
C rnd-4_family-       1737 GAAGGCAATGTTGCGCTTGACCTCGGCCATATCCGAGACAAGGGTGTCAA 1688

  contig10_pilo      98059 GGAGTGTGGGCTGTGGGCAGGACAAGTACCCAAAGTGTTGCTCTGCTTCT 98108
                                                                             
C rnd-4_family-       1687 GGAGTGTGGGCTGTGGGCAGGACAAGTACCCAAAGTGTTGCTCTGCTTCT 1638

  contig10_pilo      98109 CCTGCAGCTGCCGAAGAGCACATGACTCTGGCAAGCTCAGTGACACGCTC 98158
                                                                             
C rnd-4_family-       1637 CCTGCAGCTGCCGAAGAGCACATGACTCTGGCAAGCTCAGTGACACGCTC 1588

  contig10_pilo      98159 TGCTATCCACAGGTCTGCCCAGCGCCAGTACTGGTCTTCCTGGTTCCAGA 98208
                               i                                             
C rnd-4_family-       1587 TGCTGTCCACAGGTCTGCCCAGCGCCAGTACTGGTCTTCCTGGTTCCAGA 1538

  contig10_pilo      98209 CAGCCGCATTGACTGCAGTGGCACGTGCAATGAGGTGCTGCAGGTCAGAC 98258
                                                                             
C rnd-4_family-       1537 CAGCCGCATTGACTGCAGTGGCACGTGCAATGAGGTGCTGCAGGTCAGAC 1488

  contig10_pilo      98259 CTGATGTCATTGACTGCCTCCTCAAGTGGTGTGCCGTCTGGCAGACAGCC 98308
                                                                             
C rnd-4_family-       1487 CTGATGTCATTGACTGCCTCCTCAAGTGGTGTGCCGTCTGGCAGACAGCC 1438

  contig10_pilo      98309 CAAGTTGGCTGGCACATTCTGTGCCCTCCAGTCCTGAAGTGAGTGCACAG 98358
                                                                 v           
C rnd-4_family-       1437 CAAGTTGGCTGGCACATTCTGTGCCCTCCAGTCCTGAACTGAGTGCACAG 1388

  contig10_pilo      98359 ACTGCATGTCCTCGTCCCCACAGACTGACATAGTCTCCCCATGGGGGATT 98408
                                           v                       v         
C rnd-4_family-       1387 ACTGCATGTCCTCGTCACCACAGACTGACATAGTCTCCCCTTGGGGGATT 1338

  contig10_pilo      98409 GAGGTGCTAGGGCGAGCATAGGGCAAGTTTGTTGCAGCAGAAGACAACTT 98458
                                                     v                       
C rnd-4_family-       1337 GAGGTGCTAGGGCGAGCATAGGGCAACTTTGTTGCAGCAGAAGACAACTT 1288

  contig10_pilo      98459 GGTTGCCTGTGGTTCACCTGGGGCAGAACTGCCCCAGTTATGCGCCGATT 98508
                                                                             
C rnd-4_family-       1287 GGTTGCCTGTGGTTCACCTGGGGCAGAACTGCCCCAGTTATGCGCCGATT 1238

  contig10_pilo      98509 GAGGCTCTCCAGGAGCTGAGTCGACACCCCAGTCATTTCTGACCGAGGGG 98558
                                                                             
C rnd-4_family-       1237 GAGGCTCTCCAGGAGCTGAGTCGACACCCCAGTCATTTCTGACCGAGGGG 1188

  contig10_pilo      98559 GCGCGATCTGAGCCGCTGCCATGTGCAGCAACTCGAGGAGCGCGTCCAGA 98608
                                                                             
C rnd-4_family-       1187 GCGCGATCTGAGCCGCTGCCATGTGCAGCAACTCGAGGAGCGCGTCCAGA 1138

  contig10_pilo      98609 AGCGGTGGGAACGAGGCCCTGCATTCTGGATGCTCCCTCAGCCTGTTCTG 98658
                                                                             
C rnd-4_family-       1137 AGCGGTGGGAACGAGGCCCTGCATTCTGGATGCTCCCTCAGCCTGTTCTG 1088

  contig10_pilo      98659 GTGAGGCCGAACTCGTTTCAACGGTTTTGGGCGCAGCAGGTGAGTTAGTG 98708
                                              i                        i     
C rnd-4_family-       1087 GTGAGGCCGAACTCGTTTCGACGGTTTTGGGCGCAGCAGGTGAGCTAGTG 1038

  contig10_pilo      98709 GCTCGATATGCCGCGCCCTGCTCGAGGACGAGCCCATCCACCTTTCCTGA 98758
                                      v                                      
C rnd-4_family-       1037 GCTCGATATGCGGCGCCCTGCTCGAGGACGAGCCCATCCACCTTTCCTGA 988

  contig10_pilo      98759 CGCCCCCAGCCGGTCATCGATACCAATGCCCGTTGAACGAGCCGGAATTG 98808
                                                                             
C rnd-4_family-        987 CGCCCCCAGCCGGTCATCGATACCAATGCCCGTTGAACGAGCCGGAATTG 938

  contig10_pilo      98809 CGCCCAGGGCATCCTTGGACTCCCCAGAGCGCACAGCAGATGTTGTGAGT 98858
                                               v                             
C rnd-4_family-        937 CGCCCAGGGCATCCTTGGACACCCCAGAGCGCACAGCAGATGTTGTGAGT 888

  contig10_pilo      98859 GCAGCAGGCTCGTCCCCAGTGTGCAGTGCACTAAGGGGGCGAGGAGCAGA 98908
                                                                             
C rnd-4_family-        887 GCAGCAGGCTCGTCCCCAGTGTGCAGTGCACTAAGGGGGCGAGGAGCAGA 838

  contig10_pilo      98909 CACTGTGGGCCGCATTGAAGCGGTAGGAGCGTGTTCTGAGCGGTTTGGAG 98958
                                                                             
C rnd-4_family-        837 CACTGTGGGCCGCATTGAAGCGGTAGGAGCGTGTTCTGAGCGGTTTGGAG 788

  contig10_pilo      98959 TCAGTACAGCCAGCTGCTGCACACCAGAGTGTGCAGCTCGAGTTGGGGAG 99008
                                                                             
C rnd-4_family-        787 TCAGTACAGCCAGCTGCTGCACACCAGAGTGTGCAGCTCGAGTTGGGGAG 738

  contig10_pilo      99009 GCTGGAGGGGACTGCTGGGCTCCTCCTGCGCGTTCCGGCGCTTCCTGGAG 99058
                                                                             
C rnd-4_family-        737 GCTGGAGGGGACTGCTGGGCTCCTCCTGCGCGTTCCGGCGCTTCCTGGAG 688

  contig10_pilo      99059 GCGGTACCGGTCCGCTCCGGCCGGAGCCGATGGGGCCACGTCACCCTCGT 99108
                                                                            i
C rnd-4_family-        687 GCGGTACCGGTCCGCTCCGGCCGGAGCCGATGGGGCCACGTCACCCTCGC 638

  contig10_pilo      99109 GCAAAAACGAGCCTGGGACGCGCACAGAGTGCGCTCTGGTGCCAGGAATG 99158
                                                                             
C rnd-4_family-        637 GCAAAAACGAGCCTGGGACGCGCACAGAGTGCGCTCTGGTGCCAGGAATG 588

  contig10_pilo      99159 GTGTGGAATGCAGGAGGGGATGTAGGAGATGTGTGTGCGCGAAAATCGAG 99208
                                                  i                          
C rnd-4_family-        587 GTGTGGAATGCAGGAGGGGATGTGGGAGATGTGTGTGCGCGAAAATCGAG 538

  contig10_pilo      99209 TCCAGAAACGAAGGGGTCGTCCCCAGGGGCTGCAAAGCGAGCCTCCTGGT 99258
                                                                             
C rnd-4_family-        537 TCCAGAAACGAAGGGGTCGTCCCCAGGGGCTGCAAAGCGAGCCTCCTGGT 488

  contig10_pilo      99259 TCGTCTCGACCCCAGGATGCGAAGAACGAAAGGAGGAGCGTCTATAACGC 99308
                                                                             
C rnd-4_family-        487 TCGTCTCGACCCCAGGATGCGAAGAACGAAAGGAGGAGCGTCTATAACGC 438

  contig10_pilo      99309 TTGGAAACTGGCTGGGGCGCACCTGAGTGTCTAGGAGGGCTGTCAGACCG 99358
                               i                                             
C rnd-4_family-        437 TTGGGAACTGGCTGGGGCGCACCTGAGTGTCTAGGAGGGCTGTCAGACCG 388

  contig10_pilo      99359 CTTCCTAGTGAGCTGCCTGCTCTGCAGCTCGTTCCTGGTGCTCCTGGTGC 99408
                                                                         vi  
C rnd-4_family-        387 CTTCCTAGTGAGCTGCCTGCTCTGCAGCTCGTTCCTGGTGCTCCTGCCGC 338

  contig10_pilo      99409 TCCTGGGGGTCAACCGGCGTACACCCGACCGCACAAAGCTGCCAAGTCGC 99458
                                                                             
C rnd-4_family-        337 TCCTGGGGGTCAACCGGCGTACACCCGACCGCACAAAGCTGCCAAGTCGC 288

  contig10_pilo      99459 TGCAGAGTCGTGGGGGAGTGCGCAGGGTCGTTATCTAGCTCCATGGAAGC 99508
                                                                             
C rnd-4_family-        287 TGCAGAGTCGTGGGGGAGTGCGCAGGGTCGTTATCTAGCTCCATGGAAGC 238

  contig10_pilo      99509 GAGGGAAGCGGAGGGGGAGTGGTGGTGCGCTCTAGGAGTCCCAGTTTTGT 99558
                                                                             
C rnd-4_family-        237 GAGGGAAGCGGAGGGGGAGTGGTGGTGCGCTCTAGGAGTCCCAGTTTTGT 188

  contig10_pilo      99559 CTGGAACTCGCACCTGCGATTTCTCGCGATCCACCGGCCCAGGAGGGTCG 99608
                                                                             
C rnd-4_family-        187 CTGGAACTCGCACCTGCGATTTCTCGCGATCCACCGGCCCAGGAGGGTCG 138

  contig10_pilo      99609 CGCCCGGGCTCGATAGACTCCTGGTGGGTCCCGGAAGATGCCATGATCGT 99658
                                                          v                  
C rnd-4_family-        137 CGCCCGGGCTCGATAGACTCCTGGTGGGTCCAGGAAGATGCCATGATCGT 88

  contig10_pilo      99659 TAGATCGAAGAGGCGGATGAAGGGGTGCGCTGGAAAAGCGAGAATTGCTC 99708
                                                    i                        
C rnd-4_family-         87 TAGATCGAAGAGGCGGATGAAGGGGCGCGCTGGAAAAGCGAGAATTGCTC 38

  contig10_pilo      99709 CACTGACTCGGACTTGGTGGAAGTCGGAGTTCAAGAA 99745
                                                                
C rnd-4_family-         37 CACTGACTCGGACTTGGTGGAAGTCGGAGTTCAAGAA 1

Matrix = 20p53g.matrix
Kimura (with divCpGMod) = 0.72
Transitions / transversions = 1.00 (8/8)
Gap_init rate = 0.00 (0 / 1736), avg. gap size = 0.0 (0 / 0)

 
 

 
 
   +   12020    4.2  1.8  1.6  contig10_pilon    99753  101433 (1713446) +  rnd-4_family-108   Unknown             15   1698     (0)    47     
 
 ANNOTATION EVIDENCE: 
 12020   4.23 1.78 1.60  contig10_pilon    99753  101433   1713446 +  rnd-4_family-108   Unknown             15   1698       0      
12020 4.23 1.78 1.60 contig10_pilon 99753 101433 (1713446) rnd-4_family-108#Unknown 15 1698 (0) m_b496s001i33

  contig10_pilo      99753 CCGTGCGCACGTGCACGGGGTCCGTGCGCAGCAGTAATCCTGCCCTAGAA 99802
                                         i  i            i         ?   ?     
  rnd-4_family-         15 CCGTGCGCACGTGCGCGAGGTCCGTGCGCAACAGTAATCCNGCCNTAGAA 64

  contig10_pilo      99803 GACTGTTCAGACACCCTAGA----ATGATTGTTGAGGGTCATTCCAGTAG 99848
                            ? i         i      ----i    i     i   ?     i    
  rnd-4_family-         65 GNCCGTTCAGACATCCTAGATATTGTGATCGTTGAAGGTNATTCCGGTAG 114

  contig10_pilo      99849 CT-TACACCTACCCGCACATGTCTGTGTGCACAAAC--CTATCAAACCTT 99895
                             - i         ? i      ??       ?   -- i          
  rnd-4_family-        115 CTATGCACCTACCCNCGCATGTCNNTGTGCACNAACGGCCATCAAACCTT 164

  contig10_pilo      99896 AAAAACTGGCCAAGATATTGTCAAATTACTGGTTTCAAGTTCCGCGCGCA 99945
                                 iv   i          i ?v   ?v                   
  rnd-4_family-        165 AAAAACCCGCCGAGATATTGTCGANATACNCGTTTCAAGTTCCGCGCGCA 214

  contig10_pilo      99946 TGTTGCGCATGTTGCGCA---CGGATGCGCATGGACTGCGCACAGATTTT 99992
                           i  -     i  -     --- vi i i  ii---        i      
  rnd-4_family-        215 CGT-GCGCACGT-GCGCAAAACTAACGTGCGC---CTGCGCACGGATTTT 259

  contig10_pilo      99993 TCACACTGCGCACGAAAGTTGAAATCATGCGCACGGATTGTAAATCTGTG 100042
                                         ?         ?           --------------
  rnd-4_family-        260 TCACACTGCGCACGNAAGTTGAAANCATGCGCACGG-------------- 295

  contig10_pilo     100043 CGCACGGATTGTAAATCTGTGCGCACAGATGAAGGAGGACTGCGCACGGA 100092
                           -------                   i                       
  rnd-4_family-        296 -------ATTGTAAATCTGTGCGCACGGATGAAGGAGGACTGCGCACGGA 338

  contig10_pilo     100093 TTGTGATTTTGTGTGCACAACATTTTGGACCTTGCGCACGGA-TGTTAGA 100141
                                   i ?  i    i   ?        i          -    i  
  rnd-4_family-        339 TTGTGATTCTNTGCGCACGACANTTTGGACCCTGCGCACGGACTGTTGGA 388

  contig10_pilo     100142 ATCATGCGCACGGACA-TGGTAGACAT-GCGCACGGACATGCTGACACT- 100188
                                           - v    ? i -       ??      vi    -
  rnd-4_family-        389 ATCATGCGCACGGACAGTCGTAGNCGTTGCGCACGNNCATGCTTGCACTT 438

  contig10_pilo     100189 -GCGCACGGATTTTGAGTGTTGCGCACGGATTCTGTATAAAGCACAACTG 100237
                           -              v                                  
  rnd-4_family-        439 TGCGCACGGATTTTGTGTGTTGCGCACGGATTCTGTATAAAGCACAACTG 488

  contig10_pilo     100238 CATTATTTTTTTCACATTGGCACATCTAGGCGACTTGATTGAACTCATTG 100287
                                          v                                  
  rnd-4_family-        489 CATTATTTTTTTCACTTTGGCACATCTAGGCGACTTGATTGAACTCATTG 538

  contig10_pilo     100288 AAGTTCATTAACAAACACCAACAGTTTCCATAAGTCTTGTTGCAATTGAC 100337
                                                  i                          
  rnd-4_family-        539 AAGTTCATTAACAAACACCAACAATTTCCATAAGTCTTGTTGCAATTGAC 588

  contig10_pilo     100338 TGCTCAAGTACAGTTTGTTAATACACTACTGAATGTAGTAAACACAGGTA 100387
                                                        v                    
  rnd-4_family-        589 TGCTCAAGTACAGTTTGTTAATACACTACAGAATGTAGTAAACACAGGTA 638

  contig10_pilo     100388 GAAGTGCCACAAAATGGATGTCTATGACTGGATTTGAACTGAGGGTGTTT 100437
                             -                                               
  rnd-4_family-        639 GA-GTGCCACAAAATGGATGTCTATGACTGGATTTGAACTGAGGGTGTTT 687

  contig10_pilo     100438 CGAATCCATTAAGAACATGTACATATTAACCACTAGTCCAAACTACAGAA 100487
                                                   i                         
  rnd-4_family-        688 CGAATCCATTAAGAACATGTACATGTTAACCACTAGTCCAAACTACAGAA 737

  contig10_pilo     100488 AATACATCCTGGTAACTTGAGTATGATATGGTGTGGGCG----------- 100526
                                                   i              -----------
  rnd-4_family-        738 AATACATCCTGGTAACTTGAGTATAATATGGTGTGGGCGTGCAAGTCCCT 787

  contig10_pilo     100527 --GTAAGATGGCATGGTGAGGATGAGGTTGGAACCCATCTAATCAACAAG 100574
                           --  i                                             
  rnd-4_family-        788 CTGTGAGATGGCATGGTGAGGATGAGGTTGGAACCCATCTAATCAACAAG 837

  contig10_pilo     100575 AAGATCTGAAGGGACGGGATGGTGGCGTGATGCAGGAGAGTGAGCACCTC 100624
                                                    i i                      
  rnd-4_family-        838 AAGATCTGAAGGGACGGGATGGTGGTGCGATGCAGGAGAGTGAGCACCTC 887

  contig10_pilo     100625 TGTGGTACAGAGGTGGTGGAGCAGACACAGCAAACTTTGGCTTGTTCCAG 100674
                                                                             
  rnd-4_family-        888 TGTGGTACAGAGGTGGTGGAGCAGACACAGCAAACTTTGGCTTGTTCCAG 937

  contig10_pilo     100675 GTCCACTCCATGGCCCAAAACACGTGTTTCGTCTCTCCTTGACCCAGACA 100724
                                     i                i                      
  rnd-4_family-        938 GTCCACTCCACGGCCCAAAACACGTGTCTCGTCTCTCCTTGACCCAGACA 987

  contig10_pilo     100725 ATTCATTTGAGATTTTTTGACCAAACTGGGATCAGGGCTTAGAATCTATT 100774
                                                                             
  rnd-4_family-        988 ATTCATTTGAGATTTTTTGACCAAACTGGGATCAGGGCTTAGAATCTATT 1037

  contig10_pilo     100775 AGTAGATGAGTGAATCATGGTCACTCAGGCTGCAAAGGATATTACTCCTA 100824
                                                i                           i
  rnd-4_family-       1038 AGTAGATGAGTGAATCATGGTTACTCAGGCTGCAAAGGATATTACTCCTG 1087

  contig10_pilo     100825 AATTCATGAACCAAAATAACATAAGTTCTAACCTTAGCAAGCTTAATCTA 100874
                                                                             
  rnd-4_family-       1088 AATTCATGAACCAAAATAACATAAGTTCTAACCTTAGCAAGCTTAATCTA 1137

  contig10_pilo     100875 ACCTAACATAACATAACTGTATGAGGGTAACATCTTTTGAGTTATGAGTA 100924
                                                                             
  rnd-4_family-       1138 ACCTAACATAACATAACTGTATGAGGGTAACATCTTTTGAGTTATGAGTA 1187

  contig10_pilo     100925 CCTACTTAGAGCCTCTTCAGGAAATCAGCTTGGAACACAAACCGACATGC 100974
                                                          i           i      
  rnd-4_family-       1188 CCTACTTAGAGCCTCTTCAGGAAATCAGCTTAGAACACAAACCAACATGC 1237

  contig10_pilo     100975 TGGCTGAATATTTCTGATCCTGTATTAACGTGGAAGTGAAATTTCGAACA 101024
                                                   i                         
  rnd-4_family-       1238 TGGCTGAATATTTCTGATCCTGTACTAACGTGGAAGTGAAATTTCGAACA 1287

  contig10_pilo     101025 AATCATGTAGCACAGTAGTTTATCTAAAATAGTTGACACTAGAGATGTGC 101074
                                                                             
  rnd-4_family-       1288 AATCATGTAGCACAGTAGTTTATCTAAAATAGTTGACACTAGAGATGTGC 1337

  contig10_pilo     101075 ACCTCTATCAATGCACACAGTGCCATAAATACATCTATGATATACATCTT 101124
                                                                             
  rnd-4_family-       1338 ACCTCTATCAATGCACACAGTGCCATAAATACATCTATGATATACATCTT 1387

  contig10_pilo     101125 CAAAAGATTTTTGATTTTTTTCGCACTGCGCACG-GATTTTGTACTTTGC 101173
                                          v      i           -?v?i   v       
  rnd-4_family-       1388 CAAAAGATTTTTGATGTTTTTCACACTGCGCACGCNCNCTTGGACTTTGC 1437

  contig10_pilo     101174 GCACGGATTGTGAAAACTGCGCACGG-ATATCAGGAGCCTGCGCACGGAT 101222
                                v  i       ?         - iv  ?              v i
  rnd-4_family-       1438 GCACGCATCGTGAAAANTGCGCACGGCACTTCNGGAGCCTGCGCACGCAC 1487

  contig10_pilo     101223 TGTGAAAATTGCGCACGAAAGCACATTTCATGCGCACGGATGACCAGGAC 101272
                                   ?                                         
  rnd-4_family-       1488 TGTGAAAANTGCGCACGAAAGCACATTTCATGCGCACGGATGACCAGGAC 1537

  contig10_pilo     101273 CCTGCGCACGGATTTTGAGAAGTGCGCACAGACATTTGAACCCTGCGCAC 101322
                                             i          i        i           
  rnd-4_family-       1538 CCTGCGCACGGATTTTGAAAAGTGCGCACGGACATTTGGACCCTGCGCAC 1587

  contig10_pilo     101323 GAAAGTTAAACTCATGCGCACAAAAGTTCTAACCCTGCGCACGGAAGTTC 101372
                                                i                            
  rnd-4_family-       1588 GAAAGTTAAACTCATGCGCACGAAAGTTCTAACCCTGCGCACGGAAGTTC 1637

  contig10_pilo     101373 TGCACACGGATATTCTTGCGCACGGATGCGCACGGACCCTGCGCACGGTT 101422
                              i                                              
  rnd-4_family-       1638 TGCGCACGGATATTCTTGCGCACGGATGCGCACGGACCCTGCGCACGGTT 1687

  contig10_pilo     101423 TTCTAGGAGTC 101433
                                  i   
  rnd-4_family-       1688 TTCTAGGGGTC 1698

Matrix = 20p53g.matrix
Kimura (with divCpGMod) = 3.15
Transitions / transversions = 3.67 (55/15)
Gap_init rate = 0.02 (40 / 1680), avg. gap size = 1.43 (57 / 40)

 
 
 
 
 
  +    1211    7.9  0.0  0.0  contig10_pilon   101432  101620 (1713259) +  rnd-3_family-398   LTR/Gypsy           97    285  (8345)    48 *   
 
 ANNOTATION EVIDENCE: 
  1211   7.94 0.00 0.00  contig10_pilon   101432  101620   1713259 +  rnd-3_family-398   LTR/Gypsy           97    285    8345      
1211 7.94 0.00 0.00 contig10_pilon 101432 101620 (1713259) rnd-3_family-398#LTR/Gypsy 97 285 (8345) m_b496s001i34

  contig10_pilo     101432 TCCCGAATACCCGGCTTATCTCCTCTCATGTTCATACGCTCAGGATATGT 101481
                                                  i   i        i    i        
  rnd-3_family-         97 TCCCGAATACCCGGCTTATCTCCCCTCGTGTTCATATGCTCGGGATATGT 146

  contig10_pilo     101482 TCTCCTCATCGTCCTCTACCTCTAGACTTCTCCCTCTCATCTCTAGTCTC 101531
                                                      i        ? ?       ii  
  rnd-3_family-        147 TCTCCTCATCGTCCTCTACCTCTAGACCTCTCCCTCNCNTCTCTAGCTTC 196

  contig10_pilo     101532 TCTCCTTACTCGCATACATCCTCCTAACTCGCCTAGTCTCTACTGTATCG 101581
                                                v            v   i           
  rnd-3_family-        197 TCTCCTTACTCGCATACATCCACCTAACTCGCCTTGTCCCTACTGTATCG 246

  contig10_pilo     101582 ATAGTGTTCATATCCGGCTCGCTCCCCTAGTCTTTGTTA 101620
                                            i       v  v     v  i 
  rnd-3_family-        247 ATAGTGTTCATATCCGGTTCGCTCCACTCGTCTTAGTCA 285

Matrix = 20p53g.matrix
Kimura (with divCpGMod) = 7.45
Transitions / transversions = 2.00 (10/5)
Gap_init rate = 0.00 (0 / 188), avg. gap size = 0.0 (0 / 0)

 
 

 
   +      12   28.9  0.0  0.0  contig10_pilon   102040  102081 (1712798) +  A-rich             Low_complexity       1     42     (0)    49     
 
 ANNOTATION EVIDENCE: 
    12  28.89 0.00 0.00  contig10_pilon   102040  102081   1712798 +  (GAAAGA)n          Simple_repeat        1     42       0      
12 28.89 0.00 0.00 contig10_pilon 102040 102081 (1712798) (GAAAGA)n#Simple_repeat 1 42 (0) m_b496s252i2

  contig10_pilo     102040 GAGAGAGAACGAGATAGAGGGAGAGAGTGAGATAGAACAAGA 102081
                             i      v    v    ii     iv    v   iv    
  (GAAAGA)n#Sim          1 GAAAGAGAAAGAGAAAGAGAAAGAGAAAGAGAAAGAGAAAGA 42

Matrix = Unknown
Transitions / transversions = 1.00 (5/5)
Gap_init rate = 0.00 (0 / 41), avg. gap size = 0.0 (0 / 0)

    16  18.85 0.00 0.00  contig10_pilon   102040  102075   1712804 +  (GAGA)n            Simple_repeat        1     36       0      
16 18.85 0.00 0.00 contig10_pilon 102040 102075 (1712804) (GAGA)n#Simple_repeat 1 36 (0) m_b496s252i3

  contig10_pilo     102040 GAGAGAGAACGAGATAGAGGGAGAGAGTGAGATAGA 102075
                                   iv    v    i       v    v   
  (GAGA)n#Simpl          1 GAGAGAGAGAGAGAGAGAGAGAGAGAGAGAGAGAGA 36

Matrix = Unknown
Transitions / transversions = 0.50 (2/4)
Gap_init rate = 0.00 (0 / 35), avg. gap size = 0.0 (0 / 0)

 
 
 
 
  +    3244    6.7  0.2  0.0  contig10_pilon   102221  102670 (1712209) +  rnd-3_family-398   LTR/Gypsy          276    726  (7904)    48     
 
 ANNOTATION EVIDENCE: 
  3244   6.67 0.22 0.00  contig10_pilon   102221  102670   1712209 +  rnd-3_family-398   LTR/Gypsy          276    726    7904      
3244 6.67 0.22 0.00 contig10_pilon 102221 102670 (1712209) rnd-3_family-398#LTR/Gypsy 276 726 (7904) m_b496s001i35

  contig10_pilo     102221 GTCTTACTCATTTGCCTCTCGTCATCCGCCTGATCCCC-AATACCTTACT 102269
                                 v     i            i    i       -i         i
  rnd-3_family-        276 GTCTTAGTCATTCGCCTCTCGTCATTCGCCCGATCCCCCGATACCTTACC 325

  contig10_pilo     102270 CATTCGCCCTTACTCCTCCTATCGGCCGATGATGGAGATCTCTATTGATC 102319
                             i    i       v                i i?              
  rnd-3_family-        326 CACTCGCTCTTACTCATCCTATCGGCCGATGACGANGATCTCTATTGATC 375

  contig10_pilo     102320 TGAGGCTCCCTCTCCCTCGTCTCTAGGATACACTCCGATTACATCGGATC 102369
                                 i        i                      ?           
  rnd-3_family-        376 TGAGGCCCCCTCTCCTTCGTCTCTAGGATACACTCCGANTACATCGGATC 425

  contig10_pilo     102370 ACCTCCCCAGGTATAAAAGACCCTCAACTTTGTATCTAGCCCCCCAGTTA 102419
                                                    i              i         
  rnd-3_family-        426 ACCTCCCCAGGTATAAAAGACCCTCGACTTTGTATCTAGCTCCCCAGTTA 475

  contig10_pilo     102420 CGAAATCGACATAGTCGTGCCCCTCGCACACTCTATCTCTCTCTCTCGAC 102469
                                                        i                    
  rnd-3_family-        476 CGAAATCGACATAGTCGTGCCCCTCGCACGCTCTATCTCTCTCTCTCGAC 525

  contig10_pilo     102470 TTCACCTCTGGGTTCTTCCCTCGCCCGATCTACTCGTCTGAGTTCTCTGT 102519
                              i                        ii             i     i
  rnd-3_family-        526 TTCGCCTCTGGGTTCTTCCCTCGCCCGACTTACTCGTCTGAGTCCTCTGC 575

  contig10_pilo     102520 TGCTCACGGTCCCCTCGGTCGTGCTACTCAATGTAGCCGGTGTGCTCAAT 102569
                                i       i               i i                  
  rnd-3_family-        576 TGCTCGCGGTCCCTTCGGTCGTGCTACTCGACGTAGCCGGTGTGCTCAAT 625

  contig10_pilo     102570 AACGCACCAAAGGCTCTAGTTGTTCGTTCGAGTGTTCTCTGCACCTGGAA 102619
                                   i                                 i  i   v
  rnd-3_family-        626 AACGCACCGAAGGCTCTAGTTGTTCGTTCGAGTGTTCTCTGCGCCCGGAC 675

  contig10_pilo     102620 GTTCGACTATCCTTGCAACCAAAACCCCAAGGCACGGCAGGCTTAACAGT 102669
                                                              i              
  rnd-3_family-        676 GTTCGACTATCCTTGCAACCAAAACCCCAAGGCACAGCAGGCTTAACAGT 725

  contig10_pilo     102670 A 102670
                            
  rnd-3_family-        726 A 726

Matrix = 20p53g.matrix
Kimura (with divCpGMod) = 4.24
Transitions / transversions = 9.00 (27/3)
Gap_init rate = 0.00 (1 / 449), avg. gap size = 1.00 (1 / 1)

 
 

 
 
   +     554    2.6  1.3  1.3  contig10_pilon   102761  102839 (1712040) C  rnd-4_family-3316  LTR/Gypsy         (15)    508     430    50     
 
 ANNOTATION EVIDENCE: 
   554   2.56 1.27 1.27  contig10_pilon   102761  102839   1712040 C  rnd-4_family-3316  LTR/Gypsy          430    508      15      
554 2.56 1.27 1.27 contig10_pilon 102761 102839 (1712040) C rnd-4_family-3316#LTR/Gypsy (15) 508 430 m_b496s001i36

  contig10_pilo     102761 CCCCCCACATTCCAGGTGGATTCCAGGTGGAAAAATGCAACTATGC-AAA 102809
                                  -                                      -   
C rnd-4_family-        508 CCCCCCA-ATTCCAGGTGGATTCCAGGTGGAAAAATGCAACTATGCAAAA 460

  contig10_pilo     102810 AAAGTGTCAAAATGTTAAATCCATCATATT 102839
                                    i     i              
C rnd-4_family-        459 AAAGTGTCAGAATGTCAAATCCATCATATT 430

Matrix = 20p53g.matrix
Kimura (with divCpGMod) = 2.63
Transitions / transversions = 1.00 (2/0)
Gap_init rate = 0.03 (2 / 78), avg. gap size = 1.00 (2 / 2)

 
 
 
 
 
  +     320   27.0  4.1  0.8  contig10_pilon   102894  103016 (1711863) C  rnd-4_family-105   Unknown           (12)   1396    1270    51     
 
 ANNOTATION EVIDENCE: 
   320  27.04 4.07 0.79  contig10_pilon   102894  103016   1711863 C  rnd-4_family-105   Unknown           1270   1396      12      
320 27.04 4.07 0.79 contig10_pilon 102894 103016 (1711863) C rnd-4_family-105#Unknown (12) 1396 1270 m_b496s001i37

  contig10_pilo     102894 TATTCCTGCTACCTTGGTAGTAGGTCTTAGCGTT-CGATGCCG--GAATC 102940
                                 i              i         vi - i ii  i--     
C rnd-4_family-       1396 TATTCCCGCTACCTTGGTAGTGGGTCTTAGCCCTGCAACACCAGCGAATC 1347

  contig10_pilo     102941 CGCAATAGAAAACTATTTGATACGAAAC-GTTCCTCCTGCTGCACCT-TC 102988
                               v  vvv  i  -      vv    - iiv  ii  v  v  i -i 
C rnd-4_family-       1346 CGCACTATTTAATTA-TTGATAGTAAACTGCCACTTTTGATGAACTTCCC 1298

  contig10_pilo     102989 CTTCTATGGGCATTTTCCGCGGAAAGTT 103016
                              i     vv i  i vv    iv   
C rnd-4_family-       1297 CTTTTATGGTAACTTCCGCCGGAGCGTT 1270

Matrix = 20p53g.matrix
Kimura (with divCpGMod) = 32.87
Transitions / transversions = 1.20 (18/15)
Gap_init rate = 0.04 (5 / 122), avg. gap size = 1.20 (6 / 5)

 
 

 
 
   +    1597    0.0  0.0  0.0  contig10_pilon   106678  106873 (1708006) +  rnd-4_family-2054  LINE/Tad1         1058   1253     (0)    52     
 
 ANNOTATION EVIDENCE: 
  1597   0.00 0.00 0.00  contig10_pilon   106678  106873   1708006 +  rnd-4_family-2054  LINE/Tad1         1058   1253       0      
1597 0.00 0.00 0.00 contig10_pilon 106678 106873 (1708006) rnd-4_family-2054#LINE/Tad1 1058 1253 (0) m_b496s001i38

  contig10_pilo     106678 AACTGCGCTCACAACTTTGTTACACCCAGAATCCACACCAGTTTGCACTA 106727
                                                                             
  rnd-4_family-       1058 AACTGCGCTCACAACTTTGTTACACCCAGAATCCACACCAGTTTGCACTA 1107

  contig10_pilo     106728 GATCCCTCTACACCCCTGTTACGACCCCCCCTTGCCTGCCCCATGTTGAC 106777
                                                                             
  rnd-4_family-       1108 GATCCCTCTACACCCCTGTTACGACCCCCCCTTGCCTGCCCCATGTTGAC 1157

  contig10_pilo     106778 CCATTGGAATGTACTTTGTACAGACCACAGCGTCTTGGCTCTGTATTTGT 106827
                                                                             
  rnd-4_family-       1158 CCATTGGAATGTACTTTGTACAGACCACAGCGTCTTGGCTCTGTATTTGT 1207

  contig10_pilo     106828 AGCCTGCTTACGCTGTCTAAAATTCAAGAATTTCTCTCTCTCTCTC 106873
                                                                         
  rnd-4_family-       1208 AGCCTGCTTACGCTGTCTAAAATTCAAGAATTTCTCTCTCTCTCTC 1253

Matrix = 20p53g.matrix
Kimura (with divCpGMod) = 0.00
Transitions / transversions = 1.00 (0/0)
Gap_init rate = 0.00 (0 / 195), avg. gap size = 0.0 (0 / 0)

 
 
 
 
 
  +    2120   31.0  2.3  2.3  contig10_pilon   108112  108980 (1705899) +  rnd-4_family-2348  RC/Helitron        843   1711  (7418)    53     
 
 ANNOTATION EVIDENCE: 
  2120  30.97 2.30 2.30  contig10_pilon   108112  108980   1705899 +  rnd-4_family-2348  RC/Helitron        843   1711    7418      
2120 30.97 2.30 2.30 contig10_pilon 108112 108980 (1705899) rnd-4_family-2348#RC/Helitron 843 1711 (7418) m_b496s001i39

  contig10_pilo     108112 GCTGTTCCACATGGCGAGACTC---CTGAAACTCTATGGCACAACCCAAA 108158
                             v  i  v     --- i   ---          v     i  ivvvi 
  rnd-4_family-        843 GCAGTCCCCCATGG---GGCTCATGCTGAAACTCTCTGGCATAATAGTGA 889

  contig10_pilo     108159 TTTGTTTCCGAACATGTTTCCCTGTCTATTTCCTTATGGTGTTGGGGGCG 108208
                           i        i  v           v  i     v     vi v     vi
  rnd-4_family-        890 CTTGTTTCCAAAGATGTTTCCCTGGCTGTTTCCGTATGGAATGGGGGGAA 939

  contig10_pilo     108209 TCTGCAA----CTCGTTGATTCCAAATGCAATATCCGAACATGCACATAA 108254
                            v  i  ----  ii     ----iii v            i  v     
  rnd-4_family-        940 TATGTAATAGTCTTATTGAT----GGCGAAATATCCGAACACGCTCATAA 985

  contig10_pilo     108255 GAGGCATCTCTTGATGTATCATGATAAAAGATTTCAGATGGACAGAGATT 108304
                             ii  i  i         i i  i  iv v  i        ivv   v 
  rnd-4_family-        986 GAAACACCTTTTGATGTATTACGACAAGCGCTTCCAGATGGATCCAGAAT 1035

  contig10_pilo     108305 TTGCTATCATGGCATTCAATCAGGAACAGATTACAGAGGGAAGTAAGGC- 108353
                                v i  i     i     i        iiiiiiv  v    i   -
  rnd-4_family-       1036 TTGCTCTTATAGCATTTAATCAAGAACAGATCGTGAGTGGTAGTAGGGCT 1085

  contig10_pilo     108354 --AAGTTTGTTTCTAAGTACGAAATCAAAGTTCCAGCATATTGTAGATAT 108401
                           --   i  v  ---i  i  i   v v  i  iv  vivv i i   v i
  rnd-4_family-       1086 GGAAGCTTCTT---GAGCACAAAAACTAAATTTAAGAGGCTCGCAGAAAC 1132

  contig10_pilo     108402 GATTATGGGCATTGAACCATCAGTATTTAGCACTCTCGCTGAGAGATTGC 108451
                           v  i vvi i  i  vvvi         i viv i v     v   i vv
  rnd-4_family-       1133 TATCAGCAGTATCGATGAGTCAGTATTTGGAGATTTAGCTGATAGACTCA 1182

  contig10_pilo     108452 TAGAAAATCCTGGTTCCAAGCCT----CAAA-ATTCGGAAGAGGCACTCT 108496
                           vi v i    iv i vv      ----  i -i  vi   i i ----- 
  rnd-4_family-       1183 GGGTAGATCCCTGCTGGAAGCCTGTCACAGACGTTGAGAAAAAG-----T 1227

  contig10_pilo     108497 GTTTTAAGGTGTTGGGCGAGCTTGATATGGTCAACAGTCATGTGCCAGGA 108546
                                   i vi v  i  i              ii v     i     v
  rnd-4_family-       1228 GTTTTAAGATCCTCGGTGAACTTGATATGGTCAATGGACATGTACCAGGC 1277

  contig10_pilo     108547 TCGGTTGCAACAAAGCGGTATGAGAGAAATGAGTTGTGGTCGCTACTATC 108596
                             v  ii vi v   vii   v  v v     ii       ii ii    
  rnd-4_family-       1278 TCCGTCACTGCCAAGAAATATCAGCGCAATGAACTGTGGTCATTGTTATC 1327

  contig10_pilo     108597 ATATATAGGGGCCCCCTCATGGTTTATTACCTTTGCACCATCAGACACGC 108646
                           v  i  i     v  vv i        i  v        vv v  iiiv 
  rnd-4_family-       1328 CTACATGGGGGCACCGACGTGGTTTATCACATTTGCACCTGCTGATGTTC 1377

  contig10_pilo     108647 ACCATCCGCTCTCTGTATATTATGCAGGTACTGATGTTAGATTTGAGGAG 108696
                            i  i  vi v v v v    vi     v      ivvii      v  i
  rnd-4_family-       1378 ATCACCCTTTATGTCTTTATTTCGCAGGGACTGATAAGGAATTTGATGAA 1427

  contig10_pilo     108697 AACAGGATTGGCTTGCTACATAAAGACACTAAGCTAAAT-----TTGATC 108741
                             v i   ii ii ii   ---     iv i --     -----i  i  
  rnd-4_family-       1428 AAAAAGATCAGTCTATTAC---AAGACGATGA--TAAATGGCGACTGGTC 1472

  contig10_pilo     108742 AAAAATAATCCTGTTGCTAGTGCACGGTTTTTCCATTTCGTTGTTAAATT 108791
                           ivv  i           v        v           ii i   ivvv 
  rnd-4_family-       1473 GCCAACAATCCTGTTGCGAGTGCACGCTTTTTCCATTTTATCGTTGCTGT 1522

  contig10_pilo     108792 ATTCATACAGGAGGTCTTCAAGTGGGGAAGTGAGGAGTCTGGATTGTTTG 108841
                              i   v     i ii iviv    i  iv  v  ii    v  i    
  rnd-4_family-       1523 ATTTATAGAGGAGATTCTTCGTTGGGAAAAGGACGAACCTGGTTTATTTG 1572

  contig10_pilo     108842 GTCATACTGATGCATTCTATGGCACAGTCGAGCAACAAGGACGTCTCAGC 108891
                             vvv   i i v   i     v           i  i     i  v vv
  rnd-4_family-       1573 GTATAACTAACGGATTTTATGGAACAGTCGAGCAGCAGGGACGCCTGACA 1622

  contig10_pilo     108892 TTACATCTACATGTTCTTATCTGGATTATATTTGCTCTGTCTCCAGAAGA 108941
                             i         v v  vv i   v v v  v   v  v  v  v     
  rnd-4_family-       1623 TTGCATCTACATTTACTACTTTGGTTAAAATATGCACTCTCACCCGAAGA 1672

  contig10_pilo     108942 AATCCGACATCGCCTTTTGGATCCAGATTCTGACTTTCA 108980
                           i  vviiv v  i   i i        v   ivv     
  rnd-4_family-       1673 GATAAAGAAACGTCTTCTAGATCCAGAATCTACATTTCA 1711

Matrix = 20p53g.matrix
Kimura (with divCpGMod) = 38.02
Transitions / transversions = 1.17 (142/121)
Gap_init rate = 0.03 (27 / 868), avg. gap size = 1.48 (40 / 27)

 
 

 
 
   +     261   33.9  0.0  0.0  contig10_pilon   108984  109110 (1705769) +  rnd-4_family-2348  RC/Helitron       1624   1750  (7379)    54     
 
 ANNOTATION EVIDENCE: 
   261  33.86 0.00 0.00  contig10_pilon   108984  109110   1705769 +  rnd-4_family-2348  RC/Helitron       1624   1750    7379      
261 33.86 0.00 0.00 contig10_pilon 108984 109110 (1705769) rnd-4_family-2348#RC/Helitron 1624 1750 (7379) m_b496s001i40

  contig10_pilo     108984 TACATCTACATGTTCTTATCTGGATTATATTTGCTCTGTCTCCAGAAGAA 109033
                            i         v v  vv i   v v v  v   v  v  v  v     i
  rnd-4_family-       1624 TGCATCTACATTTACTACTTTGGTTAAAATATGCACTCTCACCCGAAGAG 1673

  contig10_pilo     109034 ATCCGACGTCGCCTTTTGGATCCAGATTCTGACTTTCAGCGGAGGATGTG 109083
                             vviiviv  i   i i        v   ivv     ivi vii   vv
  rnd-4_family-       1674 ATAAAGAAACGTCTTCTAGATCCAGAATCTACATTTCAAAAGCAAATGAT 1723

  contig10_pilo     109084 TGAATATATAGACAGCTGTCACCAAGG 109110
                           v vv   v    i i            
  rnd-4_family-       1724 AGCCTATCTAGATAACTGTCACCAAGG 1750

Matrix = 20p53g.matrix
Kimura (with divCpGMod) = 45.08
Transitions / transversions = 0.59 (16/27)
Gap_init rate = 0.00 (0 / 126), avg. gap size = 0.0 (0 / 0)

 
 
 
  +    1130   31.9  0.8  0.8  contig10_pilon   109286  109785 (1705094) +  rnd-4_family-2348  RC/Helitron       1923   2422  (6707)    54     
 
 ANNOTATION EVIDENCE: 
  1130  31.85 0.80 0.80  contig10_pilon   109286  109785   1705094 +  rnd-4_family-2348  RC/Helitron       1923   2422    6707      
1130 31.85 0.80 0.80 contig10_pilon 109286 109785 (1705094) rnd-4_family-2348#RC/Helitron 1923 2422 (6707) m_b496s001i41

  contig10_pilo     109286 CATTCATGTGGTGATCACTGTCTGGATAAAAATCGAATATGTAAAGCACG 109335
                              vv     i i   iv     v iv vvv vv i vi  i   iv   
  rnd-4_family-       1923 CATAAATGTGATAATCGATGTCTTGGGACCCAGGGGAAGTGCAAAAGACG 1972

  contig10_pilo     109336 CTTCCCACGTAGTTTGATACCTCGCACTTCTGTCGATGATACTGGATATA 109385
                           v  i  v  i iv   i vvvvviv    v   v     v     v  i 
  rnd-4_family-       1973 GTTTCCTCGCAAATTGGTTAAGGAGACTTATGTGGATGAGACTGGTTACA 2022

  contig10_pilo     109386 TTCATGTCCGACACGATGAACGCAGAATGAATACGGTTAATCATACACTT 109435
                            v  i  iv           vivv          vi      vv ivi v
  rnd-4_family-       2023 TACACGTTAGACACGATGAAAAGCGAATGAATACTATTAATCCAATTTTA 2072

  contig10_pilo     109436 ACTTATCTTTTGAGGTGCAATACTGATGTCACCTGCTGACTGAATGGAAC 109485
                             v   i v  iv v  i  i           v    vi  v ii     
  rnd-4_family-       2073 ACATATTTATTACGCTGTAACACTGATGTCACGTGCTTGCTTAGCGGAAC 2122

  contig10_pilo     109486 TGCGATCAAGGCCGCCATTGCATATGCGACCGATTACATCACAAAGGTTG 109535
                           v  v  i  i  v ii     i     i vi        i  i       
  rnd-4_family-       2123 AGCTATTAAAGCAGTTATTGCGTATGCAAGTGATTACATTACGAAGGTTG 2172

  contig10_pilo     109536 GGCTAAAGACACCTAGTATGTTCCAGCTAATTCGCACACAGCTTGAT-CG 109584
                                  i  v   i v      i v  v  i  vv v   -   v -  
  rnd-4_family-       2173 GGCTAAAAACTCCTGGAATGTTCTATCTTATCCGATCCCAG-TTGCTGCG 2221

  contig10_pilo     109585 CAGTGCTTCAATTTCA---ACTGGTGAAGATGCACGATTAGAGAAGGGGA 109631
                           ivi    v vv viv ---   i v vvii   -i  --   v  vii v
  rnd-4_family-       2222 TCATGCTGCCCTACAAGGCACTAGAGCCAGTGC-TGA--AGATAACAAGC 2268

  contig10_pilo     109632 GGAAGTTAATTACAGGCATTATTAATAGTTTTACATCTAAAACTGAGATT 109681
                            v  iv vi    v  v              i  v  i  i  v     v
  rnd-4_family-       2269 GCAAAATTGTTACTGGAATTATTAATAGTTTCACCTCCAAGACAGAGATA 2318

  contig10_pilo     109682 GGATCACCTATGGCTGCAAGCTATATCCTCGATCTTCCTGACCATTATAA 109731
                                v  v     v   ivv   v    i  i  v  v       v   
  rnd-4_family-       2319 GGATCTCCAATGGCAGCAGCGTATCTCCTTGACCTGCCAGACCATTTTAA 2368

  contig10_pilo     109732 AATCCATGAGTTTAAAGTATTATACTGGAAGAGCTTTGTCGCAGAGGTGC 109781
                           i vi   v i      vi v        vivi i     vv    i   i
  rnd-4_family-       2369 GAGTCATCAATTTAAACCAATATACTGGCGTGGTTTTGTATCAGAAGTGT 2418

  contig10_pilo     109782 TGTC 109785
                            i  
  rnd-4_family-       2419 TATC 2422

Matrix = 20p53g.matrix
Kimura (with divCpGMod) = 39.70
Transitions / transversions = 0.74 (67/91)
Gap_init rate = 0.01 (6 / 499), avg. gap size = 1.33 (8 / 6)

 
 

   +     548   32.7  3.4  3.1  contig10_pilon   110104  110396 (1704483) +  rnd-4_family-2348  RC/Helitron       2726   3019  (6110)    54     
 
 ANNOTATION EVIDENCE: 
   548  32.74 3.41 3.06  contig10_pilon   110104  110396   1704483 +  rnd-4_family-2348  RC/Helitron       2726   3019    6110      
548 32.74 3.41 3.06 contig10_pilon 110104 110396 (1704483) rnd-4_family-2348#RC/Helitron 2726 3019 (6110) m_b496s001i42

  contig10_pilo     110104 AGGTCATTCACAGGAAGGCACGCACTCTGTATTCCTCGATG------GTG 110147
                              i     v     i iiv i  iiiv   ivi  v   i------   
  rnd-4_family-       2726 AGGCCATTCCCAGGAGGATTCACATCTGGTACATCTGGATAACCGTCGTG 2775

  contig10_pilo     110148 CAAGAGCCGACGTGGTACCAAATTTTGTAGGAGGAACTCTACCGCGCTGT 110197
                              ------ i  v  i     i  ii v  v  vv vv v  iv vi i
  rnd-4_family-       2776 CAA------ATGTTGTGCCAAACTTCATTGGTGGTTCGATTCCAAGGCGC 2819

  contig10_pilo     110198 GATATGGGTGATAGAGAATATTACTGCTGCACTATGTTAAC-CTGTTCAA 110246
                              iiv  v  i  i  v v   i  i              -  i     
  rnd-4_family-       2820 GATGCCGGGGACAGGGATTTTTATTGTTGCACTATGTTAACACTATTCAA 2869

  contig10_pilo     110247 ACCATGGAGGACGGGTTTGGACCTGAAATCCCTAGATGTTGCTTGGAATC 110296
                                    i  i   i    iv i   v  vvvi   vvv i   i vv
  rnd-4_family-       2870 ACCATGGAGAACAGGTCTGGATATAAAAACCAACAATGAGTCCTGGGAAG 2919

  contig10_pilo     110297 AGGCCTTCGAAAACAAT---GGCTTCAGTGAAAGGCAGAGACAGATCATG 110343
                           i  iv  i - vi  v --- vi   --  vvv v  ivvv  i  i   
  rnd-4_family-       2920 GGGTATTTG-ATGCATTCCAGTTTTC--TGCCCGTCAATCTCAAATTATG 2966

  contig10_pilo     110344 AATAATTTTAACCTGAGATATGAGTGTCTAGATGAGCGAGACGATTATAG 110393
                           i    i      i    i     i  ii      v v          i  
  rnd-4_family-       2967 GATAACTTTAACTTGAGGTATGAATGCTTAGATGCGAGAGACGATTACAG 3016

  contig10_pilo     110394 CCA 110396
                           i  
  rnd-4_family-       3017 TCA 3019

Matrix = 20p53g.matrix
Kimura (with divCpGMod) = 42.86
Transitions / transversions = 1.21 (51/42)
Gap_init rate = 0.04 (12 / 292), avg. gap size = 1.58 (19 / 12)

 
 
 
  +     610   35.4  3.1  5.1  contig10_pilon   110876  111491 (1703388) +  rnd-4_family-2348  RC/Helitron       3502   4105  (5024)    54     
 
 ANNOTATION EVIDENCE: 
   610  35.42 3.08 5.13  contig10_pilon   110876  111491   1703388 +  rnd-4_family-2348  RC/Helitron       3502   4105    5024      
610 35.42 3.08 5.13 contig10_pilon 110876 111491 (1703388) rnd-4_family-2348#RC/Helitron 3502 4105 (5024) m_b496s001i43

  contig10_pilo     110876 TAATCCATGAATTCTCTCTCAATACTGAACAATCACGTGCCTTCCAGATG 110925
                                vvv     iivv  v     v  i   iv   v  v  i     v
  rnd-4_family-       3502 TAATCACAGAATTTCAACTAAATACAGAGCAACAACGGGCATTTCAGATT 3551

  contig10_pilo     110926 GTTACACGGCACCATGCCAATGGCTCTCAGGGCCAGCTGAAACTGTATAT 110975
                             ii v ii  ii  viivii  i i   i ii vv   viiv    iv 
  rnd-4_family-       3552 GTCGCCCAACATTATCTTTGCGGTTTTCAAGATCCCCTGCGGATGTACTT 3601

  contig10_pilo     110976 GGCTGGTATGGCAGGGACGGGAAAATCCAGAGTCATAAATGCGATTTCGA 111025
                           i              i  i        v        vv     v v   v
  rnd-4_family-       3602 AGCTGGTATGGCAGGAACAGGAAAATCGAGAGTCATTCATGCGTTATCGT 3651

  contig10_pilo     111026 TGTATTTTAGAAGGCTGGGAATATCCAATA--TG-CTGCTGTTACTTGCT 111072
                           ii -    v  iv   -v    i- vii  --  -i    vi    v   
  rnd-4_family-       3652 CAT-TTTTTGAGCGCT-TGAATG-CAGGTAGCTGTTTGCTCCTACTAGCT 3698

  contig10_pilo     111073 CCAACTGGAACTGCTGCGGCAAATATAGCGGGTTATACTTATCATGCTGC 111122
                             v  v  v  v     v        v vv  vi    v  i   v i  
  rnd-4_family-       3699 CCCACAGGCACAGCTGCTGCAAATATTGGTGGACATACATACCATTCCGC 3748

  contig10_pilo     111123 ATTTGGTTTGAGGCC---AGGCGCCTATGCAGCTCGAAAACTCAGTTCCT 111169
                            i v  v  i  vi --- i i   --- v vvv  iiviv v iv  iv
  rnd-4_family-       3749 ACTAGGATTAAGCTCAAAAAGTGCC---GGACAACGGGCGATGAAATCTG 3795

  contig10_pilo     111170 CTGTACGGTCACTATTAGAGCCTGTTCG--------ATATATTATTATTG 111211
                            ii i  --------   v   ivi   --------      vv    v 
  rnd-4_family-       3796 CCATGCG--------TAGTGCCCTCTCGCGGGTAAAATATATATTTATAG 3837

  contig10_pilo     111212 ATGAGGTTTCCATGTTATCCTGTAGAGATTTG-----TATACAATTTCAT 111256
                               i  i  v   v v  v  ivv    v  -----    iv vvv i 
  rnd-4_family-       3838 ATGAAGTCTCAATGATTTCATGCTCAGATATGAAACTTATATCAGAACGT 3887

  contig10_pilo     111257 CTGTATTATCATCCATTAGCGACGACCACGTGAATCCCTATGGTGGACTT 111306
                             -i v   v  v  ---- i i vv ii iii vv v  i     vi i
  rnd-4_family-       3888 CT-CAGTATGATACA----CAATGTACGTGCAGAGGCATACGGTGGCTTC 3932

  contig10_pilo     111307 AATATTATGTTTGCCGGGGATTTTGCACAATTGCCCCCTGTAGGAGGAAG 111356
                             i     v     v  v        i  i     v      vv   viv
  rnd-4_family-       3933 AACATTATTTTTGCAGGCGATTTTGCGCAGTTGCCACCTGTATTAGGTGC 3982

  contig10_pilo     111357 AAGGTTATATGACTCTATGGTTGGTAAATCAGTTGGTAAGTCTAGGCAGG 111406
                           v vi  i  ii i  v v   i  - i   i--i   ---   -- vv  
  rnd-4_family-       3983 TACATTGTACAATTCAAAGGTCGG-AGATCG--CGGT---TCT--GATGG 4024

  contig10_pilo     111407 TGTCAAACAGCCAGAAAGATGCCACTGGCAAGGCACAGTGGTTACAGGTT 111456
                            vvii v  i ----i vi   v iv  v  i  vivi   i v     i
  rnd-4_family-       4025 TTATGATCAAC----GATGTGCAATAGGGAAAGCTTTATGGCTCCAGGTC 4070

  contig10_pilo     111457 GTCGATGTTGTGATTCTTCGTGAGAACATGAGGCA 111491
                           i iivi  v  v  ii v  v  i           
  rnd-4_family-       4071 ATTACCGTAGTTATCTTGCGAGAAAACATGAGGCA 4105

Matrix = 20p53g.matrix
Kimura (with divCpGMod) = 44.18
Transitions / transversions = 0.92 (99/108)
Gap_init rate = 0.06 (36 / 615), avg. gap size = 1.39 (50 / 36)

 
 

   +     973   37.4  3.0  1.1  contig10_pilon   111621  112564 (1702315) +  rnd-4_family-2348  RC/Helitron       4235   5195  (3934)    54     
 
 ANNOTATION EVIDENCE: 
   973  37.40 2.97 1.14  contig10_pilon   111621  112564   1702315 +  rnd-4_family-2348  RC/Helitron       4235   5195    3934      
973 37.40 2.97 1.14 contig10_pilon 111621 112564 (1702315) rnd-4_family-2348#RC/Helitron 4235 5195 (3934) m_b496s001i44

  contig10_pilo     111621 GTTGGAAGAAGAGCAATATAGGTATCTTTCTGTTATTACGGGGTTTAACT 111670
                                    v  i iv v   ii iv i     v  v     vv v  iv
  rnd-4_family-       4235 GTTGGAAGACGAACGTTTTAGACACGTCTCTGTAATAACGGGTGTAAATG 4284

  contig10_pilo     111671 CTCATCGGGACACCATCAACAGTATGGGATGTGAAAAATTTGCTCGAGAC 111720
                            vi    v   i v  v    vvv i              i  v iv  i
  rnd-4_family-       4285 CATATCGTGACGCAATAAACACGTTAGGATGTGAAAAATTCGCACATGAT 4334

  contig10_pilo     111721 ACAGGTCAGGAGCTTATATCATTCTACTCTATTGACCATCCAGCTAGTGC 111770
                            ii i v iviv  iivv           v      v --- ii i   -
  rnd-4_family-       4335 ATGGATGAACGTCTCGATTCATTCTACTCAATTGACAA---AATTGGTG- 4380

  contig10_pilo     111771 AAAGAGTGGCGAGACTCGG---------------CGCCATGGTAAGCAGT 111805
                           -   -   ii  v    i ---------------   vivv       i 
  rnd-4_family-       4381 -AAG-GTGATGACACTCAGTCTGGTCCCAAAAAACGCGGGCGTAAGCAAT 4428

  contig10_pilo     111806 CTATTGCAATTACTCCTGAA-ACC--CGAGACATGTACTGGAATCTGACT 111852
                           vvvv   ii v ivi v  v-i  --  ii    v vv   i  i  v  
  rnd-4_family-       4429 GGCGTGCGGTAATATCAGATCGCCTCCGGAACATTTTATGGGATTTGCCT 4478

  contig10_pilo     111853 CACTTTGAGTCTTCAAATGTTGCTGGTCTTTTACGATTGTGTAGGGGTAT 111902
                               v   v  vvvv     vv     v i  i  vv v  i ii     
  rnd-4_family-       4479 CACTATGACTCAGACAATGTGCCTGGTGTCTTGCGCATTTGCAAAGGTAT 4528

  contig10_pilo     111903 GCCGATAATAGTCAAAAAGAACCAT-GCGACAGAGTGCGGTGTAACTAAC 111951
                               i i  vi  -       vi  -        i        i     i
  rnd-4_family-       4529 GCCGGTGATTATC-AAAAGAAATATAGCGACAGAATGCGGTGTGACTAAT 4577

  contig10_pilo     111952 GGGAGCGAGGGAGTAGTGGTAGATTGGATATCACGCCCACTATTTGATGG 112001
                             vi v  i  v  v  v  i i    vv   vv iv vv  vvv     
  rnd-4_family-       4578 GGTGGAGAAGGCGTTGTTGTGGGTTGGCAATCCAGTGCCATAGGAGATGG 4627

  contig10_pilo     112002 TAAACTGGTCCTGGACGTTCTTTTTGTCAAACTGACTGCTGATAATGTAG 112051
                           i    viiii  v  i  v  v     ivii  v  iiv  i  vvv ii
  rnd-4_family-       4628 CAAACGAACTCTTGATGTACTATTTGTTCGGCTCACCAATGGTACATTGA 4677

  contig10_pilo     112052 ATGTCCAGATTGAGGGCTTACCACCTAATGTTGTCCCCATATGCTCCGA- 112100
                             i i   v v        i  i     i  v  i  vv v   v v  -
  rnd-4_family-       4678 ATATTCAGCTGGAGGGCTTGCCGCCTAACGTAGTTCCACTCTGCGCAGAG 4727

  contig10_pilo     112101 -TGTTTCCGACATTGATTGTATGTTGGATAGTGACCACCGTGTACGGATA 112149
                           -v v iv v   --i    i  v  ivviii   ivvvii v  vvi  v
  rnd-4_family-       4728 GAGATCACTACA--AATTGCATTTTACCCGATGATACGTATTTAACAATC 4775

  contig10_pilo     112150 AGAAGACAGCAGGTTCATGTGCTGCCAAATTTCTCAATGACTGACTTTGC 112199
                           v vv  v    i  v  i  i  v     i  iv v     v  i vi v
  rnd-4_family-       4776 CGTCGAAAGCAAGTACACGTACTCCCAAACTTTGCCATGACAGATTACGG 4825

  contig10_pilo     112200 GAGCCAAGGAAAGACGAGGAAGTACAATCTAGTTGATCTTCATAACCTCT 112249
                           v        v  i  vv v vvv     v v  v   i vivv  i  vi
  rnd-4_family-       4826 CAGCCAAGGTAAAACTCGTATTAACAATGTTGTGGATTTGTTGAATCTGC 4875

  contig10_pilo     112250 ATTCTCACCATGGATACTATACCGCATTATCTCGAAGTTCATATGCGCAA 112299
                           ivvv      v vi  i     i   i v  v  v   v v v   v   
  rnd-4_family-       4876 GAGGTCACCAGGCGTATTATACTGCACTTTCGCGTAGTGCCTTTGCTCAA 4925

  contig10_pilo     112300 AATACCCTAATATCACAAGGCGGTTTCTCG---CTTATGT----GGAGGG 112342
                           v       v  -  v v   - i  i v  ---  i ii ----  v  i
  rnd-4_family-       4926 TATACCCTCAT-TCTCCAGG-GATTCCACGAATCTCACATTCAGGGTGGA 4973

  contig10_pilo     112343 CTTGACTATGACCTTAAAGCCGAATTCCGCGATCTTGAAATTCTGGATGA 112392
                             vii v v  i  vvv v i     i  i  iv v     vi    i  
  rnd-4_family-       4974 CTGAGCAAGGATCTATTATCTGAATTTCGTGACATAGAAATATTGGACGA 5023

  contig10_pilo     112393 CATTACACGCATGCGTTATGAAGGTAAATTACCAATTACCGTAATATCAG 112442
                                 v   v   iv viiivii v i  i  v viv i  vvi    v
  rnd-4_family-       5024 CATTACTCGCTTGCAATTCAGCAATCAGTTGCCCAACTCTGTTTCATCAC 5073

  contig10_pilo     112443 AATTGAGATATCCCTGCATAGCCCAATTTCTATTAACTCAGGGTGTTCAG 112492
                           v    v     v vv v     v  i viv  vv  v  iv  ii v  v
  rnd-4_family-       5074 CATTGCGATATACAAGGATAGCACAGTACATAGAAAGTCGTGGCATACAT 5123

  contig10_pilo     112493 TACCGACCACCCCATCTGCACCCTACTTTACAATGGACTGATGATTATAG 112542
                             iv    v  vv ii    ivvv v i iv v   iv  v v    iiv
  rnd-4_family-       5124 TATAGACCTCCAAACTTGCATAAAAGTCTGGATTGGGATGTTCATTACGC 5173

  contig10_pilo     112543 TCGCCTTCTTGTTTTCAGGGAT 112564
                               ii v vv v  i  ii  
  rnd-4_family-       5174 TCGCTCTATACTATTTAGAAAT 5195

Matrix = 20p53g.matrix
Kimura (with divCpGMod) = 48.26
Transitions / transversions = 0.73 (147/202)
Gap_init rate = 0.02 (19 / 943), avg. gap size = 2.05 (39 / 19)

 
 
 
 
 
  +      14   31.1  0.0  0.0  contig10_pilon   116880  116927 (1697952) +  (CTC)n             Simple_repeat        1     48     (0)    55     
 
 ANNOTATION EVIDENCE: 
    14  31.12 0.00 0.00  contig10_pilon   116880  116927   1697952 +  (CTC)n             Simple_repeat        1     48       0      
14 31.12 0.00 0.00 contig10_pilon 116880 116927 (1697952) (CTC)n#Simple_repeat 1 48 (0) m_b497s252i0

  contig10_pilo     116880 CTACTCCTCCTCCAGAGGCGCCTCCTCCTCGTACTCTACGTCCTCCTC 116927
                             v          vvvvv v          v v   iv v        
  (CTC)n#Simple          1 CTCCTCCTCCTCCTCCTCCTCCTCCTCCTCCTCCTCCTCCTCCTCCTC 48

Matrix = Unknown
Transitions / transversions = 0.09 (1/11)
Gap_init rate = 0.00 (0 / 47), avg. gap size = 0.0 (0 / 0)

 
 

 
 
   +      13   21.8  6.2  0.0  contig10_pilon   123427  123474 (1691405) +  (CACGCCG)n         Simple_repeat        1     51     (0)    56     
 
 ANNOTATION EVIDENCE: 
    13  21.80 6.25 0.00  contig10_pilon   123427  123474   1691405 +  (CACGCCG)n         Simple_repeat        1     51       0      
13 21.80 6.25 0.00 contig10_pilon 123427 123474 (1691405) (CACGCCG)n#Simple_repeat 1 51 (0) m_b497s252i1

  contig10_pilo     123427 CACGCC-C-CGCC-CACGCAGCATATCGCGCGCTGAACGCCGCCCGCAGC 123473
                                 - -    -     v   iii   i   i v       v   v  
  (CACGCCG)n#Si          1 CACGCCGCACGCCGCACGCCGCACGCCGCACGCCGCACGCCGCACGCCGC 50

  contig10_pilo     123474 A 123474
                            
  (CACGCCG)n#Si         51 A 51

Matrix = Unknown
Transitions / transversions = 1.25 (5/4)
Gap_init rate = 0.06 (3 / 47), avg. gap size = 1.00 (3 / 3)

 
 
 
 
 
  +      13   22.4  0.0  4.7  contig10_pilon   124834  124878 (1690001) +  (GCCTTG)n          Simple_repeat        1     43     (0)    57     
 
 ANNOTATION EVIDENCE: 
    13  22.43 0.00 4.65  contig10_pilon   124834  124878   1690001 +  (GCCTTG)n          Simple_repeat        1     43       0      
13 22.43 0.00 4.65 contig10_pilon 124834 124878 (1690001) (GCCTTG)n#Simple_repeat 1 43 (0) m_b497s252i2

  contig10_pilo     124834 GCATTTGCCTCGTCCTTGGACTTGGACTTGACCTTTCGGCCCTGG 124878
                             v  v    i v      v     v    i  -  -    i   
  (GCCTTG)n#Sim          1 GCCTTGGCCTTGGCCTTGGCCTTGGCCTTGGCC-TT-GGCCTTGG 43

Matrix = Unknown
Transitions / transversions = 0.60 (3/5)
Gap_init rate = 0.05 (2 / 44), avg. gap size = 1.00 (2 / 2)

 
 

 
 
   +      13   33.1  0.0  4.5  contig10_pilon   128161  128230 (1686649) +  (GCG)n             Simple_repeat        1     67     (0)    58     
 
 ANNOTATION EVIDENCE: 
    13  33.13 0.00 4.48  contig10_pilon   128161  128230   1686649 +  (GCG)n             Simple_repeat        1     67       0      
13 33.13 0.00 4.48 contig10_pilon 128161 128230 (1686649) (GCG)n#Simple_repeat 1 67 (0) m_b497s252i3

  contig10_pilo     128161 GCGACGACGGAGGAGGTGAAGGCGGACGCGAGCCGGCGGGGGATAGCAGC 128210
                              i  i   v  v  i iv     -  - v v      v  -ii  i  
  (GCG)n#Simple          1 GCGGCGGCGGCGGCGGCGGCGGCGG-CG-GCGGCGGCGGCGG-CGGCGGC 47

  contig10_pilo     128211 GGTGACGGCGTCGGCGGTGG 128230
                             i i     v      i  
  (GCG)n#Simple         48 GGCGGCGGCGGCGGCGGCGG 67

Matrix = Unknown
Transitions / transversions = 1.43 (10/7)
Gap_init rate = 0.04 (3 / 69), avg. gap size = 1.00 (3 / 3)

 
 
 
 
 
  +      13    0.0  0.0  0.0  contig10_pilon   131531  131547 (1683332) +  (GCG)n             Simple_repeat        1     17     (0)    59     
 
 ANNOTATION EVIDENCE: 
    13   0.00 0.00 0.00  contig10_pilon   131531  131547   1683332 +  (GCG)n             Simple_repeat        1     17       0      
13 0.00 0.00 0.00 contig10_pilon 131531 131547 (1683332) (GCG)n#Simple_repeat 1 17 (0) m_b497s252i4

  contig10_pilo     131531 GCGGCGGCGGCGGCGGC 131547
                                            
  (GCG)n#Simple          1 GCGGCGGCGGCGGCGGC 17

Matrix = Unknown
Transitions / transversions = 1.00 (0/0)
Gap_init rate = 0.00 (0 / 16), avg. gap size = 0.0 (0 / 0)

 
 

 
 
   +      11   21.2  2.5  2.5  contig10_pilon   136633  136672 (1678207) +  (GTCCGA)n          Simple_repeat        1     40     (0)    60     
 
 ANNOTATION EVIDENCE: 
    11  21.20 2.50 2.50  contig10_pilon   136633  136672   1678207 +  (GTCCGA)n          Simple_repeat        1     40       0      
11 21.20 2.50 2.50 contig10_pilon 136633 136672 (1678207) (GTCCGA)n#Simple_repeat 1 40 (0) m_b497s252i5

  contig10_pilo     136633 GTCCGAGTCCGCGT-AGAGCACGAGTTGCGTCTCCGAGTCC 136672
                                      v  -v   iv    - v  vv         
  (GTCCGA)n#Sim          1 GTCCGAGTCCGAGTCCGAGTCCGAG-TCCGAGTCCGAGTCC 40

Matrix = Unknown
Transitions / transversions = 0.17 (1/6)
Gap_init rate = 0.05 (2 / 39), avg. gap size = 1.00 (2 / 2)

 
 
 
 
 
  +      12    7.5  6.7  3.2  contig10_pilon   153885  153914 (1660965) +  (CTCG)n            Simple_repeat        1     31     (0)    61     
 
 ANNOTATION EVIDENCE: 
    12   7.49 6.67 3.23  contig10_pilon   153885  153914   1660965 +  (CTCG)n            Simple_repeat        1     31       0      
12 7.49 6.67 3.23 contig10_pilon 153885 153914 (1660965) (CTCG)n#Simple_repeat 1 31 (0) m_b497s252i6

  contig10_pilo     153885 CTCGCCCGCTC-CTCGCTCGC-CGCCTGGCTC 153914
                                i     -         -  -  v    
  (CTCG)n#Simpl          1 CTCGCTCGCTCGCTCGCTCGCTCG-CTCGCTC 31

Matrix = Unknown
Transitions / transversions = 1.00 (1/1)
Gap_init rate = 0.10 (3 / 29), avg. gap size = 1.00 (3 / 3)

 
 

 
 
   +      13   21.7  0.0  0.0  contig10_pilon   161416  161447 (1653432) +  GA-rich            Low_complexity       1     32     (0)    62     
 
 ANNOTATION EVIDENCE: 
    13  21.71 0.00 0.00  contig10_pilon   161416  161447   1653432 +  (GGA)n             Simple_repeat        1     32       0      
13 21.71 0.00 0.00 contig10_pilon 161416 161447 (1653432) (GGA)n#Simple_repeat 1 32 (0) m_b497s252i7

  contig10_pilo     161416 GGCGGTGGAGGAGGCGGTGGAGGAGGGGGTGG 161447
                             v  v        v  v        i  v  
  (GGA)n#Simple          1 GGAGGAGGAGGAGGAGGAGGAGGAGGAGGAGG 32

Matrix = Unknown
Transitions / transversions = 0.20 (1/5)
Gap_init rate = 0.00 (0 / 31), avg. gap size = 0.0 (0 / 0)

 
 
 
 
 
  +      21   21.1  1.6  3.2  contig10_pilon   167132  167195 (1647684) +  (ACCTCG)n          Simple_repeat        1     63     (0)    63     
 
 ANNOTATION EVIDENCE: 
    21  21.09 1.56 3.17  contig10_pilon   167132  167195   1647684 +  (ACCTCG)n          Simple_repeat        1     63       0      
21 21.09 1.56 3.17 contig10_pilon 167132 167195 (1647684) (ACCTCG)n#Simple_repeat 1 63 (0) m_b497s252i8

  contig10_pilo     167132 ACCT-GACCTTGGTCCTCGTCCTCGACCTCATCCTCGCTCCCCGACCCCG 167180
                               -    - v v     v          iv     -v  i     i  
  (ACCTCG)n#Sim          1 ACCTCGACC-TCGACCTCGACCTCGACCTCGACCTCG-ACCTCGACCTCG 48

  contig10_pilo     167181 ACCCCGACCCCGGCC 167195
                              i     i  i  
  (ACCTCG)n#Sim         49 ACCTCGACCTCGACC 63

Matrix = Unknown
Transitions / transversions = 1.20 (6/5)
Gap_init rate = 0.05 (3 / 63), avg. gap size = 1.00 (3 / 3)

 
 

 
 
   +      19   12.8  0.0  0.0  contig10_pilon   169843  169876 (1645003) +  (GGA)n             Simple_repeat        1     34     (0)    64     
 
 ANNOTATION EVIDENCE: 
    19  12.84 0.00 0.00  contig10_pilon   169843  169876   1645003 +  (GGA)n             Simple_repeat        1     34       0      
19 12.84 0.00 0.00 contig10_pilon 169843 169876 (1645003) (GGA)n#Simple_repeat 1 34 (0) m_b497s252i9

  contig10_pilo     169843 GGAAGATGACGAGGAGGAGGAGGAAGAGGAGGAG 169876
                              i  v  v              i         
  (GGA)n#Simple          1 GGAGGAGGAGGAGGAGGAGGAGGAGGAGGAGGAG 34

Matrix = Unknown
Transitions / transversions = 1.00 (2/2)
Gap_init rate = 0.00 (0 / 33), avg. gap size = 0.0 (0 / 0)

 
 
 
 
 
  +      17   21.8  0.0  5.9  contig10_pilon   180488  180541 (1634338) +  (CTG)n             Simple_repeat        1     51     (0)    65     
 
 ANNOTATION EVIDENCE: 
    17  21.82 0.00 5.88  contig10_pilon   180488  180541   1634338 +  (CTG)n             Simple_repeat        1     51       0      
17 21.82 0.00 5.88 contig10_pilon 180488 180541 (1634338) (CTG)n#Simple_repeat 1 51 (0) m_b498s252i0

  contig10_pilo     180488 CTGCTGTTGCTGCTGTTGCTGCTCGCTTCTCTTACTTCCCGCCTGTTGCT 180537
                                 i        i       -   v  vi i - v i -   i    
  (CTG)n#Simple          1 CTGCTGCTGCTGCTGCTGCTGCT-GCTGCTGCTGC-TGCTG-CTGCTGCT 47

  contig10_pilo     180538 GCTG 180541
                               
  (CTG)n#Simple         48 GCTG 51

Matrix = Unknown
Transitions / transversions = 2.00 (6/3)
Gap_init rate = 0.06 (3 / 53), avg. gap size = 1.00 (3 / 3)

 
 

 
 
   +      12   22.0  4.8  0.0  contig10_pilon   182359  182400 (1632479) +  (GCGTGG)n          Simple_repeat        1     44     (0)    66     
 
 ANNOTATION EVIDENCE: 
    12  21.98 4.76 0.00  contig10_pilon   182359  182400   1632479 +  (GCGTGG)n          Simple_repeat        1     44       0      
12 21.98 4.76 0.00 contig10_pilon 182359 182400 (1632479) (GCGTGG)n#Simple_repeat 1 44 (0) m_b498s252i1

  contig10_pilo     182359 GCGCGTGCGGGGGC-TGGGCGCGGGCATGCGCGACGGC-TGGGC 182400
                              i v   v    -      i    i  v   vv   -     
  (GCGTGG)n#Sim          1 GCGTGGGCGTGGGCGTGGGCGTGGGCGTGGGCGTGGGCGTGGGC 44

Matrix = Unknown
Transitions / transversions = 0.60 (3/5)
Gap_init rate = 0.05 (2 / 41), avg. gap size = 1.00 (2 / 2)

 
 
 
 
 
  +      12   22.4  0.0  2.7  contig10_pilon   182445  182482 (1632397) +  (GCTGGT)n          Simple_repeat        1     37     (0)    67     
 
 ANNOTATION EVIDENCE: 
    12  22.42 0.00 2.70  contig10_pilon   182445  182482   1632397 +  (GCTGGT)n          Simple_repeat        1     37       0      
12 22.42 0.00 2.70 contig10_pilon 182445 182482 (1632397) (GCTGGT)n#Simple_repeat 1 37 (0) m_b498s252i2

  contig10_pilo     182445 GCTGTGGCTGCTGGTGCTGCTGGTACTGGCGCTGAGTG 182482
                               vv    v  v  v       i    i    -   
  (GCTGGT)n#Sim          1 GCTGGTGCTGGTGCTGGTGCTGGTGCTGGTGCTG-GTG 37

Matrix = Unknown
Transitions / transversions = 0.40 (2/5)
Gap_init rate = 0.03 (1 / 37), avg. gap size = 1.00 (1 / 1)

 
 

 
 
   +     252    5.2  0.0  7.7  contig10_pilon   192900  192941 (1621938) +  rnd-4_family-509   rRNA              9542   9580 (17176)    68     
 
 ANNOTATION EVIDENCE: 
   252   5.16 0.00 7.69  contig10_pilon   192900  192941   1621938 +  rnd-4_family-509   rRNA              9542   9580   17176      
252 5.16 0.00 7.69 contig10_pilon 192900 192941 (1621938) rnd-4_family-509#rRNA 9542 9580 (17176) m_b498s001i0

  contig10_pilo     192900 GGCCAAGTCAATGCATCGGTATTTCGTTTTACACCCTGGAAG 192941
                              i       v  ---                         
  rnd-4_family-       9542 GGCTAAGTCAAAGC---GGTATTTCGTTTTACACCCTGGAAG 9580

Matrix = 20p53g.matrix
Kimura (with divCpGMod) = 5.32
Transitions / transversions = 1.00 (1/1)
Gap_init rate = 0.07 (3 / 41), avg. gap size = 1.00 (3 / 3)

 
 
 
 
 
  +      12    5.5  0.0  0.0  contig10_pilon   193734  193752 (1621127) +  (GAC)n             Simple_repeat        1     19     (0)    69     
 
 ANNOTATION EVIDENCE: 
    12   5.48 0.00 0.00  contig10_pilon   193734  193752   1621127 +  (GAC)n             Simple_repeat        1     19       0      
12 5.48 0.00 0.00 contig10_pilon 193734 193752 (1621127) (GAC)n#Simple_repeat 1 19 (0) m_b498s252i3

  contig10_pilo     193734 GACGACGAGGACGACGACG 193752
                                   v          
  (GAC)n#Simple          1 GACGACGACGACGACGACG 19

Matrix = Unknown
Transitions / transversions = 0.00 (0/1)
Gap_init rate = 0.00 (0 / 18), avg. gap size = 0.0 (0 / 0)

 
 

 
 
   +      13   31.3  0.0  0.0  contig10_pilon   196600  196646 (1618233) +  (CGA)n             Simple_repeat        1     47     (0)    70     
 
 ANNOTATION EVIDENCE: 
    13  31.29 0.00 0.00  contig10_pilon   196600  196646   1618233 +  (CGA)n             Simple_repeat        1     47       0      
13 31.29 0.00 0.00 contig10_pilon 196600 196646 (1618233) (CGA)n#Simple_repeat 1 47 (0) m_b498s252i4

  contig10_pilo     196600 CGACGGCGGCGAGCACGACGAGGACGAGGAGGTCTATGCCGTCGACG 196646
                                i  i   vv       v     v  v v v i v  v     
  (CGA)n#Simple          1 CGACGACGACGACGACGACGACGACGACGACGACGACGACGACGACG 47

Matrix = Unknown
Transitions / transversions = 0.33 (3/9)
Gap_init rate = 0.00 (0 / 46), avg. gap size = 0.0 (0 / 0)

 
 
 
 
 
  +      15    5.5  0.0  0.0  contig10_pilon   199661  199679 (1615200) +  (CT)n              Simple_repeat        1     19     (0)    71     
 
 ANNOTATION EVIDENCE: 
    15   5.48 0.00 0.00  contig10_pilon   199661  199679   1615200 +  (CT)n              Simple_repeat        1     19       0      
15 5.48 0.00 0.00 contig10_pilon 199661 199679 (1615200) (CT)n#Simple_repeat 1 19 (0) m_b498s252i5

  contig10_pilo     199661 CTCTCTCTCTCACTCTCTC 199679
                                      v       
  (CT)n#Simple_          1 CTCTCTCTCTCTCTCTCTC 19

Matrix = Unknown
Transitions / transversions = 0.00 (0/1)
Gap_init rate = 0.00 (0 / 18), avg. gap size = 0.0 (0 / 0)

 
 

 
 
   +      39    0.0  0.0  0.0  contig10_pilon   211472  211507 (1603372) +  (T)n               Simple_repeat        1     36     (0)    72     
 
 ANNOTATION EVIDENCE: 
    39   0.00 0.00 0.00  contig10_pilon   211472  211507   1603372 +  (T)n               Simple_repeat        1     36       0      
39 0.00 0.00 0.00 contig10_pilon 211472 211507 (1603372) (T)n#Simple_repeat 1 36 (0) c_b498s251i0

  contig10_pilo     211472 TTTTTTTTTTTTTTTTTTTTTTTTTTTTTTTTTTTT 211507
                                                               
  (T)n#Simple_r          1 TTTTTTTTTTTTTTTTTTTTTTTTTTTTTTTTTTTT 36

Matrix = Unknown
Transitions / transversions = 1.00 (0/0)
Gap_init rate = 0.00 (0 / 35), avg. gap size = 0.0 (0 / 0)

 
 
 
 
 
  +     243   33.2  0.0  6.8  contig10_pilon   211611  211736 (1603143) C  rnd-4_family-874   LINE/L1-Tx1      (118)   6107    5990    73     
 
 ANNOTATION EVIDENCE: 
   243  33.20 0.00 6.78  contig10_pilon   211611  211736   1603143 C  rnd-4_family-874   LINE/L1-Tx1       5990   6107     118      
243 33.20 0.00 6.78 contig10_pilon 211611 211736 (1603143) C rnd-4_family-874#LINE/L1-Tx1 (118) 6107 5990 m_b498s001i1

  contig10_pilo     211611 TGTAATGACGCTTCCCCCCGGGGAAATCGCACTTGGCACGCCTTTTCAGG 211660
                             v  i    viv  vv    ii    v  vi  i    i   v  vi  
C rnd-4_family-       6107 TGAAACGACGACACCGACCGGAAAAATAGCCTTTAGCACACCTGTTGGGG 6058

  contig10_pilo     211661 TAGTGTGGGGCTCGGAGTTAGGCGTCTCAGCTGACGCCGTCTGCTACTTG 211710
                                       i i-------- vv   i i   i    iivivi v v
C rnd-4_family-       6057 TAGTGTGGGGCTTGA--------GAATCAACCGACACCGTTCCTGGCGTC 6016

  contig10_pilo     211711 CTCTCCTATACCCACTAAAACCCCGG 211736
                           v ii  ii            vv    
C rnd-4_family-       6015 ATTCCCCGTACCCACTAAAAAACCGG 5990

Matrix = 20p53g.matrix
Kimura (with divCpGMod) = 40.00
Transitions / transversions = 1.17 (21/18)
Gap_init rate = 0.06 (8 / 125), avg. gap size = 1.00 (8 / 8)

 
 

   +     466   37.1  2.1  2.1  contig10_pilon   211870  212301 (1602578) C  rnd-4_family-874   LINE/L1-Tx1      (372)   5853    5422    73     
 
 ANNOTATION EVIDENCE: 
   466  37.11 2.08 2.08  contig10_pilon   211870  212301   1602578 C  rnd-4_family-874   LINE/L1-Tx1       5422   5853     372      
466 37.11 2.08 2.08 contig10_pilon 211870 212301 (1602578) C rnd-4_family-874#LINE/L1-Tx1 (372) 5853 5422 m_b498s001i2

  contig10_pilo     211870 ACGTGACTTCAGCATGCTTTTCCATTTCTTCTGGACCTGCTCCGGTGACC 211919
                                 i ivvi   vi i      i    vvvv    v   ivv  i -
C rnd-4_family-       5853 ACGTGATTCGCACATTTTCTTCCATCTCTTGGCCACCTCCTCTTCTGGC- 5805

  contig10_pilo     211920 AATGCCTT--TCGGGGTCATCTCCGTGCTCGATGCGACGAAGGCACCTCA 211967
                           -   v ii--  v  i  v  vv       i  i  ivvvvvi     i 
C rnd-4_family-       5804 -ATGACCCAGTCTGGATCCTCAGCGTGCTCAATACGGGTTTCACACCTTA 5756

  contig10_pilo     211968 GTTTCCATATTAGGAATGCGGATTCAGCTACTACTATGCGGAAGGCGCGG 212017
                           vi     v  i     v        v vi  v iv    i    ii   v
C rnd-4_family-       5755 TCTTCCAGATCAGGAAGGCGGATTCTGACACAATAATGCAGAAGATGCGC 5706

  contig10_pilo     212018 GCCAGGCCTTCAGTGC--GCTGTCGCCATGTACCTTCTGGGCGCGTACTC 212065
                           v    vv viv v v --  v v v      ii i v v--   i iv  
C rnd-4_family-       5705 TCCAGCGCACGATTTCTTGCAGACCCCATGTGTCCTATT--CGCATGGTC 5658

  contig10_pilo     212066 GCGCAGCCCCATATCGTTCCTATGGAGGGACGGATGGAGATCTGTTTCTT 212115
                             i  i   vvv  viii  v   vvviviivv   vv     vvivvi 
C rnd-4_family-       5657 GCACAACCCATGATAACCCCGATGTCTATGTCTATGTTGATCTTGCGACT 5608

  contig10_pilo     212116 CTTCAAGAATAATGTTA--CCAGTTTCCAGATCGTCTCCTGGCCAG---T 212160
                                i   --i vi i--   ivvi   v ii  i  vvii  v --- 
C rnd-4_family-       5607 CTTCAGGAA--GTCCTGGGCCAAGGCCCATACTGTTTCAAAACCTGAGCT 5560

  contig10_pilo     212161 CTCCGGGCACTCAAGGAGGATGTGCTCCATCGACTCCGTCACGTGGCACG 212210
                              ---   i iv           i     v v   v  iv v     i 
C rnd-4_family-       5559 CTC---GCATTTTAGGAGGATGTGTTCCATGGTCTCAGTTTCCTGGCATG 5513

  contig10_pilo     212211 TAGGGCAGTTCTCGCGGGACTTCCAGGGTTCCGGCATTTTGCTAAAGTAG 212260
                           vv  i  vv vv iiiv   vi   v     i           i  i   
C rnd-4_family-       5512 ATGGACATGTGGCATATGACGCCCATGGTTCTGGCATTTTGCTGAAATAG 5463

  contig10_pilo     212261 GTGCCGACTTTGTGGGCGTCGTGTAGTCCTTTCCAGAGGAA 212301
                           vi   i        v  ii i  vivivi      i     
C rnd-4_family-       5462 TCGCCAACTTTGTGCGCACCATGGGCCATTTTCCAAAGGAA 5422

Matrix = 20p53g.matrix
Kimura (with divCpGMod) = 50.00
Transitions / transversions = 0.85 (72/85)
Gap_init rate = 0.03 (13 / 431), avg. gap size = 1.38 (18 / 13)

 
 
 
  +     238   30.4  1.7  1.7  contig10_pilon   212624  212740 (1602139) C  rnd-4_family-874   LINE/L1-Tx1     (1126)   5099    4983    73     
 
 ANNOTATION EVIDENCE: 
   238  30.43 1.71 1.71  contig10_pilon   212624  212740   1602139 C  rnd-4_family-874   LINE/L1-Tx1       4983   5099    1126      
238 30.43 1.71 1.71 contig10_pilon 212624 212740 (1602139) C rnd-4_family-874#LINE/L1-Tx1 (1126) 5099 4983 m_b498s001i3

  contig10_pilo     212624 TTCTTTGCTACGTGTGTGAACGTCGTCGCAGCACTCCGGCTACGCAGGGC 212673
                                v    i ii        vv    vv    ii v vi      i  
C rnd-4_family-       5099 TTCTTGGCTATGCATGTGAACGAGGTCGGTGCACCTCTGGCACGCAGAGC 5050

  contig10_pilo     212674 AGCGATCGCTGCTCTCATGGCCGGGCCGTCTCCGT--GTAGGTTTTGCCA 212721
                           v  iii        v      -- v i i   vv -- i i  iivv   
C rnd-4_family-       5049 TGCAGCCGCTGCTCGCATGGC--GTCTGCCTCATTTGGCAAGTCCATCCA 5002

  contig10_pilo     212722 TCCCGTGTCCTCGTTCTTG 212740
                              v   vv   i v    
C rnd-4_family-       5001 TCCAGTGGACTCATGCTTG 4983

Matrix = 20p53g.matrix
Kimura (with divCpGMod) = 39.48
Transitions / transversions = 0.94 (17/18)
Gap_init rate = 0.03 (3 / 116), avg. gap size = 1.33 (4 / 3)

 
 

   +    2538   35.9  1.6  1.8  contig10_pilon   213221  214992 (1599887) C  rnd-4_family-874   LINE/L1-Tx1     (1723)   4502    2734    73     
 
 ANNOTATION EVIDENCE: 
  2538  35.86 1.64 1.81  contig10_pilon   213221  214992   1599887 C  rnd-4_family-874   LINE/L1-Tx1       2734   4502    1723      
2538 35.86 1.64 1.81 contig10_pilon 213221 214992 (1599887) C rnd-4_family-874#LINE/L1-Tx1 (1723) 4502 2734 m_b498s001i4

  contig10_pilo     213221 GTAGCGGTGTTCCATTTCTCGGCTATCGAGCTGAGAACATCACTCGCCAG 213270
                             v  i        i  vvvv  v v vv   v  v ii    vv  v i
C rnd-4_family-       4502 GTCGCAGTGTTCCACTTGGGCGCAAGCTCGCTCAGTATGTCACGAGCAAA 4453

  contig10_pilo     213271 GTCCTGGCATCTCGCTGGGTTTCTGCATCCTTCGGCGCGGTCTGTCGCGC 213320
                           v         i vivv     ii    v  i  v iv vi  v   ii  
C rnd-4_family-       4452 CTCCTGGCATTTGAAGGGGTTCTTGCAGCCCTCTGTTCTATCAGTCATGC 4403

  contig10_pilo     213321 AGTTCTCACAGGGGCAGGTCC--GCCTTCCGGTGTGGTCCGGGCAGTCCA 213368
                            i  v  i  viv      v -- v  i i--ii     vii ivi  vi
C rnd-4_family-       4402 AATTGTCGCATATGCAGGTACTAGACTCCT--CATGGTCGAAGTCATCAG 4355

  contig10_pilo     213369 GTTCCGCTAGTTCTTCGAGGTCTTCGACTGTCGCGACAGCGTGGTTGGTT 213418
                               v    vi        i     i  i  vvv   v  i  ivv   i
C rnd-4_family-       4354 GTTCAGCTATCTCTTCGAGATCTTCAACCGTACAGACCGCATGAGAGGTC 4305

  contig10_pilo     213419 CGGAGGCACTTCAGTTGGCCACGTTTATTGT-GTA--ATATGTTGCCATC 213465
                           ivv     iiv  v  vv iv - v    v - i -- i  v v iii  
C rnd-4_family-       4304 TTTAGGCATCGCACTTCTCTCC-TATATTCTCGCAGGACATCTGGTTGTC 4256

  contig10_pilo     213466 CAGAAAGGCTGGGTGCGACCAGACCTGCACCCTCTCTCTGATTTCCAGCG 213515
                            v-- v         i v   v ivv   iviii  i  v  v v vvi 
C rnd-4_family-       4255 CC--ATGGCTGGGTGTGCCCATATGGGCATATCTTCCCTTATGTGCTCTG 4208

  contig10_pilo     213516 GTATCACCGGTGCATCCACCGTGACACCGTATTTTCGTGCCGTTCTTACC 213565
                            v   vvv  i     v vv i    iii     ivv   viii  i   
C rnd-4_family-       4207 GGATCTGAGGCGCATCGAGGGCGACATTATATTTCGCTGCAACCCTCACC 4158

  contig10_pilo     213566 ATTTGCCTTAGCACAAACGGCAGCTTGTTTAGATTTGGACGCCAGTCCTG 213615
                                 ivi iv ii  v  ?  i  v vivvi  v  v     vv    
C rnd-4_family-       4157 ATTTGCTGCAAGATGAAAGGNAGTTTCTGCTTGTTGGGTCGCCACACCTG 4108

  contig10_pilo     213616 GTAGAAGGGGTTCGCCCTTTGATCTGGAGGGATTCGGGCCTTCTGGTC-T 213664
                           v i         viiv v vvi    vv  v ii i---i    v   - 
C rnd-4_family-       4107 TTGGAAGGGGTTGATGCGTGCGTCTGTTGGCACCCA---TTTCTTGTCCT 4061

  contig10_pilo     213665 AG--GATCATCGCCGTTTCGAAGAT----TCGCTCAGCTACTACTGCCCA 213708
                           v --   v  v  v   ii      ----  vvi   v  vvv ii vi?
C rnd-4_family-       4060 TGTTGATGATGGCAGTTCTGAAGATGAAGTCTGCCAGGTAGGTCCACATN 4011

  contig10_pilo     213709 GGCTG-GCCTCAATGGTTCTGGCGCCATGAATGCGCTGATCCAGGATAGG 213757
                             vi - i    viiv  i  -----   v      iv i   vvvi   
C rnd-4_family-       4010 GGACGCGTCTCATCATTTTTG-----ATGTATGCGCCCACCCATTTCAGG 3966

  contig10_pilo     213758 TACTGTGCTTCATTCCTCTCTTCTGCATGAAAAAGGTTCATCCCGCCTTC 213807
                                i  v        viii  v ii  i  v   ii  vv  v  v -
C rnd-4_family-       3965 TACTGCGCATCATTCCTGCTCTCAGTGTGGAACAGGCCCAGACCTCCAT- 3917

  contig10_pilo     213808 CGATCTCGGG--AGCCGTAGAGTCGTCGCGTTAATGGGGTGTCGTTTCAT 213855
                           - i    v v--  v  i     vvi iii     v     v  vivviv
C rnd-4_family-       3916 -GGTCTCTGCCCAGGCGCAGAGTATCCATATTAATTGGGTGGCGGCGAGA 3868

  contig10_pilo     213856 GTTCCCCCAGAAGAAATTCAACGTCGTCTTATCTAGCCGTCTCTCTATGC 213905
                            iiiv      i   i iv i    i i     i  v   i i  i -  
C rnd-4_family-       3867 GCCTGCCCAGAGGAAGTCAAGCGTCATTTTATCCAGGCGTTTTTCCA-GC 3819

  contig10_pilo     213906 TCTGC-GGCATGCCCTGTGCTGCCGTCAGGAATTGCGTGTGGCCGCCGAT 213954
                            iv i-        i  v  v  v  v   v i  v  iviv     v i
C rnd-4_family-       3818 TTGGTTGGCATGCCTTGGGCAGCAGTAAGGTACTGGGTAAATCCGCCCAC 3769

  contig10_pilo     213955 GATCACTCTGGAGACCATACTTCTGCCGTTTAGCGACGGTC--TTGCGCG 214002
                           v iv  iivv  v iv  i  i  i  i  i     v  v --  i viv
C rnd-4_family-       3768 CACAACCTGTGATATAATGCTCCTACCATTCAGCGAGGGGCGCTTACTTT 3719

  contig10_pilo     214003 CGACCAGCGCGCCAGTTTGTCGTCGAGCTTGTCTAGGATCGTGGACCAGG 214052
                            --   v vivvi  vv v  iv i vi     iv    v v        
C rnd-4_family-       3718 C--CCATCTTTGTAGAGTTTCAGCAATTTTGTCCTGGATGGAGGACCAGG 3671

  contig10_pilo     214053 CGTTTACAACGCCGACTGCGTTTCCGGGCCATGCGCCTAGAATACGTGTG 214102
                            i  i   vivivv   v i  v  v     v ii  v  v  i  vi  
C rnd-4_family-       3670 CATTCACATTCTGCACTCCATTGCCTGGCCAGGTACCAAGTATGCGAATG 3621

  contig10_pilo     214103 GGTTCGCCATCCCTGGCTATACGTGCTTGCGCTGGGAGTTCTATATCCGT 214152
                                      i     v  i  ii  vivv      vvv vivi   vi
C rnd-4_family-       3620 GGTTCGCCATCTCTGGCAATGCGCACTGAGTCTGGGATGGCAGGGTCCCC 3571

  contig10_pilo     214153 TTCGTTCAGGCGGCGCGACTGGATCAGG---CGTGCTCTGTACTCCGGAG 214199
                           v  v  v    iv v   v ---i    ---i   ii        ivvv 
C rnd-4_family-       3570 GTCTTTGAGGCATCTCGAAT---CCAGGACTTGTGTCCTGTACTCTTTTG 3524

  contig10_pilo     214200 AGCCCAACGGGATGATCTCAGTCTTCTTTATGTTAAACTTCGCACCTGAG 214249
                           vi    vi  i i   i     i    vi ii  i  vivv       vv
C rnd-4_family-       3523 TACCCATTGGAACGATTTCAGTTTTCTGCACATTGAAGCGAGCACCTGCT 3474

  contig10_pilo     214250 GCTAGACACCAC--GTCCTTAGCATGCTCCGGA-GAGTC--TCGTATGAG 214294
                             v  i      --    ----- v v  i   -   v -- v   v   
C rnd-4_family-       3473 GCAAGGCACCACTGGTCC-----AGGATCTGGAAGAGGCGCTGGTAAGAG 3429

  contig10_pilo     214295 TCAGCTGCCGAGAGGAATACCGTCGTGTCGTCCGCATACAGGGAGGCGAC 214344
                              viiv vvvv v v i  i  i  i  i  i       iv v     i
C rnd-4_family-       3428 TCATTCTCACTCATGTACACTGTTGTATCATCTGCATACAATGTGGCGAT 3379

  contig10_pilo     214345 CAGCTTCTCCGTGAGGCCGTTTACACTTAT-GCCGTGCAGCGTCGAGTTC 214393
                           i  i  vi  vii v   i iv i-   i -   v      viv   ivi
C rnd-4_family-       3378 TAGTTTGCCCTCAATGCCATCAAT-CTTGTAGCCTTGCAGCTCAGAGCGT 3330

  contig10_pilo     214394 CTCAGCATGGCTGAGAGTGGTTCGATCGCGAGGTTGAACAGTAGGCACGA 214443
                             v     i        i  i  v  i  v i   i  v  i i   v  
C rnd-4_family-       3329 CTGAGCATAGCTGAGAGCGGCTCTATTGCCAAGTTAAAAAGCAAGCAGGA 3280

  contig10_pilo     214444 TAGCGGGTCGCCTTGCCGTACCCCTCTCGTGACCGTGTATTTCGTGCTAA 214493
                           v  ?  i  v  i    vv  v     v  i  iv iv i  iv    vi
C rnd-4_family-       3279 GAGNGGATCTCCCTGCCTGACGCCTCTGGTAACTCTAAACTTTTTGCTTG 3230

  contig10_pilo     214494 TGCAGCCGTTTACTATGACCGCTGTTCTCGCATTTTTGTACAGAGACTCT 214543
                           i v    i  v ii      i v  vvvv       ii     v v iiv
C rnd-4_family-       3229 CGAAGCCATTGATCATGACCACAGTGAAAGCATTTTCATACAGCGTCCTG 3180

  contig10_pilo     214544 ATCGTTCGTACCAAGCGTTCTGGGAAATTGTATCCACGGAGAATGGTCAG 214593
                            i  ivivi  v   i i        iiiiv vv viv      vv vv 
C rnd-4_family-       3179 ACCGCATTCACAAAGTGCTCTGGGAAGCCAAAGGCTTTGAGAATCTTGTG 3130

  contig10_pilo     214594 GAGGTAGTCATGTCTTATTCGGTCATATGCTTTTTCCTGGTCAAGGGCAA 214643
                           v  i              v  i     v              v  v  v 
C rnd-4_family-       3129 CAGATAGTCATGTCTTATGCGATCATAAGCTTTTTCCTGGTCCAGCGCCA 3080

  contig10_pilo     214644 CTATTGCGCCGTCTGTTTCTGTCGCTTCCGCATAGTCGATGACCAGTGTA 214693
                            v  v     i i vi   iv v     v  i  i  i v  iv  vv v
C rnd-4_family-       3079 CGATGGCGCCATTTTCTTCCTTGGCTTCAGCGTAATCAAGGATGAGACTC 3030

  contig10_pilo     214694 GTGAGTTTAGTCTGGTCTGTGATGCTCCGACCTCGCACGAAACCTGCTTG 214743
                               v   i vv           i  v     ii v  i  i  v  i  
C rnd-4_family-       3029 GTGATTTTGGAATGGTCTGTGATACTGCGACCCTGGACAAAGCCAGCCTG 2980

  contig10_pilo     214744 TGATTTGTGAATGATTTCGGGGGC--TGCTCGGGCCAGCCTTAGGGCCAG 214791
                           v    ii       vi v   v  --    --i        v  v    i
C rnd-4_family-       2979 AGATTCATGAATGAGCTGGGGCGCGATGCT--AGCCAGCCTGAGCGCCAA 2932

  contig10_pilo     214792 CGCCTTCGTGTATGTCTTATAGTCCGAGTTCAGAAGGGTTATCGGTCTGT 214841
                           v     v     vi i  i     i v   v  i  v  v     i  i 
C rnd-4_family-       2931 GGCCTTGGTGTAAATTTTGTAGTCTGTGTTGAGGAGTGTGATCGGCCTAT 2882

  contig10_pilo     214842 AGTTTGATATATCGTCCCTGTCCCCTTTTTTGTACAGGGGGCATAACCAA 214891
                               i vv  v   iii  i  i  i  i       i      i i   i
C rnd-4_family-       2881 AGTTCGCGATCTCGCTTCTATCTCCCTTCTTGTACAAGGGGCACAGCCAG 2832

  contig10_pilo     214892 CCGTCCGTGAAGTCGGAGGAGTGATCCACTCCGAAGGTTTCCAGGTCCTC 214941
                             v  i i   v i    vvvvvi  i vv  ivi iv   v vi  i  
C rnd-4_family-       2831 CCTTCTGCGAATTTGGAGTCTGCGTCTAAGCCATGGAGTTCAATATCTTC 2782

  contig10_pilo     214942 GAATACTAGGAGCATTGCGCCGACGACGTCGAAGGCAGGTTGGTTTCTTT 214991
                           i  v  i vviv   iiii  v        i    i---   i i ii  
C rnd-4_family-       2781 AAAGACCATTGTCATCATACCCACGACGTCAAAGGT---TTGATCTTCTT 2735

  contig10_pilo     214992 T 214992
                            
C rnd-4_family-       2734 T 2734

Matrix = 20p53g.matrix
Kimura (with divCpGMod) = 47.49
Transitions / transversions = 0.94 (302/322)
Gap_init rate = 0.03 (48 / 1771), avg. gap size = 1.27 (61 / 48)

 
 
 
  +     437   36.7  2.9  2.9  contig10_pilon   215538  216576 (1598303) C  rnd-4_family-874   LINE/L1-Tx1     (4040)   2185    1147    73     
 
 ANNOTATION EVIDENCE: 
   437  36.67 2.89 2.89  contig10_pilon   215538  216576   1598303 C  rnd-4_family-874   LINE/L1-Tx1       1147   2185    4040      
437 36.67 2.89 2.89 contig10_pilon 215538 216576 (1598303) C rnd-4_family-874#LINE/L1-Tx1 (4040) 2185 1147 m_b498s001i5

  contig10_pilo     215538 TTCTCGTCTTCTCTGCGGTTTT--CGTTTTGGATTGTCGTGCGCAGTTTA 215585
                                - v    -    v    -- iv   v vvi i vv        v 
C rnd-4_family-       2185 TTCTC-TGTTCT-TGCGCTTTTGTCAATTTTGTGCGCCTGGCGCAGTTAA 2138

  contig10_pilo     215586 ATTCTCTGGCTCTCGCTTTGGCGCACTTAGCTATGCGTGACTTTAGGTCT 215635
                           iv   i  vi   v  i     vvv   vvi   vi ivvi  i  iiv 
C rnd-4_family-       2137 GATCTTTGCTTCTGGCCTTGGCTGCCTTTCTTATCTGCTCTTTCAGACGT 2088

  contig10_pilo     215636 TTCCAGATTAGTTGCGGGTTGTTCTTGTCGGTCCTGTTCTGACTCGAGGC 215685
                            i    i vv i  v        vvvvv v      ii viivvv v ii
C rnd-4_family-       2087 TCCCAGGTACGCTGGGGGTTGTTGAATGCTGTCCTGCCCAAGAAGGCGAT 2038

  contig10_pilo     215686 GGCTTTCATTT--GCTCGAGCGCAGTCCTGCCCATCTCGTCTATCATGGA 215733
                              v i   i -- vii i    -- ii    v vi i  vi i viv  
C rnd-4_family-       2037 GGCGTCCATCTCTGACTGGGCGC--TTTTGCCAAGTTTGTACACCTCTGA 1990

  contig10_pilo     215734 CATAAGTCTCGTGTCCCGTATCGCAGATGCTTG--CATGGTCCATCTGCC 215781
                              i ivviiv      v i i--  i v  ii--         v vv  
C rnd-4_family-       1989 CATGAAAACTTTGTCCCTTGTT--AGGTTCTCAGACATGGTCCAGCGTCC 1942

  contig10_pilo     215782 AGGGCCGATATCTGGTGCGTCTAGGTGTGCGATTTGGACCGAGACACGGC 215831
                              i  i  i     v  i   vv iiii v v  vv  v    iv v  
C rnd-4_family-       1941 AGGACCAATGTCTGGAGCATCTCTGCACACTAGTTCCACAGAGATTCTGC 1892

  contig10_pilo     215832 TGTGGTCGT-TGGATGTCAGGCCTGAGATTTGCGTCTGCCATTCTCTCGT 215880
                                  i -i v  - i v   ?  v iv vvi i v   i v   vv 
C rnd-4_family-       1891 TGTGGTCATACGTAT-TTATGCCNGACACGTTAATTTCCCACTGTCTGCT 1843

  contig10_pilo     215881 GGATGA--GGATAACGCTCGTGAAAGGTATATTCTGTCCAATCGTGACAT 215928
                            ivi v-- v  vvi i v  v --    v  i  i  v vv  v     
C rnd-4_family-       1842 GACCGCACGCATTTTGTTGGTCA--GGTAGATCCTATCAATGCGAGACAT 1795

  contig10_pilo     215929 CGAGGACCTCGTTGGGTTCCTCCAGGTCCACTCCACTGTGTCCGGGAAGG 215978
                           v      vvv viiv   i v   v     i  v ivv    v     i 
C rnd-4_family-       1794 GGAGGACAGAGACATGTTTCGCCATGTCCATTCGATGCTGTCAGGGAAAG 1745

  contig10_pilo     215979 TTTCTCGCCATACGTCCGTTACTCGCA-ACGCCTTCTTTAGTGTTCTCAG 216027
                            v    v   iv i  v  v   iv? - iii   i vvi  v   vv  
C rnd-4_family-       1744 TGTCTCTCCACCCATCGGTGACTTTNAGATATCTTTTGAGGTCTTCGAAG 1695

  contig10_pilo     216028 CGACTCTGGATTGTCTATGGGCGTTAGCTTGGCTGGGAATCTGTCTACTG 216077
                           -    v   vvi      vvvi vi ii  v  v     v vi  v iv 
C rnd-4_family-       1694 -GACTGTGGTGCGTCTATCTTTGACAATTTTGCAGGGAAGCGATCGATGG 1646

  contig10_pilo     216078 GGTCCTCGACGCAGTTCATGTCACCCAGCAGTATTTCTGGGCGTGCCAGG 216127
                                  v  vii   v vi  v    i  vv iv     - v ivviiv
C rnd-4_family-       1645 GGTCCTCCACCTGGTTGAAATCTCCCAACATGACATCTGG-CTTAGGGAT 1597

  contig10_pilo     216128 TTGAAGCGAGGCTCGCGT---AGCCTGCGTTTTATAAGCTTCCAGAAACT 216174
                           i v vv vvv  v   v ---  i  v--     v iv  i      vii
C rnd-4_family-       1596 CTTACCCTTTGCACGCTTGAGAGTCTT--TTTTAGAGCCTCCCAGAATTC 1549

  contig10_pilo     216175 TTTGTTCTCGTGCTTGT---TTGTTGGAGCGTATACCGCCAGGATACAAA 216221
                            i i  iv ---    v--- v  v  v       iv  v    iivvv 
C rnd-4_family-       1548 TCTATTTGC---CTTGGCAGTGGTGGGCGCGTATATGGCGAGGACGGCCA 1502

  contig10_pilo     216222 GACGCTTG--CCTCTATGCCATTCGAATTCTAC-TGCTATCGCTCTTCCT 216268
                            --     --i i  i          -  i i -ii       i      
C rnd-4_family-       1501 G--GCTTGAGTCCCTGTGCCATTCGA-TTTTGCACACTATCGCCCTTCCT 1455

  contig10_pilo     216269 TCCACAAGGGTGACCTGTTTGGCC---GTTTGGGTGTTCGTCAGGAGTTT 216315
                           vvv  v   ---  v  vv  v v---  vi ivv   i  v  v     
C rnd-4_family-       1454 GGAACTAGG---ACATGAGTGTCAATAGTGCGATAGTTTGTGAGCAGTTT 1408

  contig10_pilo     216316 ATTCAGAACGATCGCGACCCCTCCGGCGCCCGTTGGGTTCGCTGGCAGGG 216365
                           i  v ii  v v   v  v  i  i        v     i        v 
C rnd-4_family-       1407 GTTGAAGACCAGCGCCACGCCCCCAGCGCCCGTGGGGTTTGCTGGCAGCG 1358

  contig10_pilo     216366 CGGAATTAAATATTTTCAGGT-TTCG--ATACATGGT---TTCCGCC--T 216407
                            ivv   vvv  iv vv    -i   --i   -    --- ii ii -- 
C rnd-4_family-       1357 CACTATTCTCTACGTGGAGGTGCTCGTAGTAC-TGGTGAATCTCATCAAT 1309

  contig10_pilo     216408 GGGTTACATGTTCATTCGACATATGTGTCTCCTGCAGGGCTATCGCTCCT 216457
                            v i i i----   -   vi -- v        v v   i  vii   v
C rnd-4_family-       1308 GTGCTGCG----CAT-CGAGGT--GGGTCTCCTGGATGGCCATGATTCCG 1266

  contig10_pilo     216458 ATCTTCTGCGCGCGGATGTCGTCTGCAAGTTTGCGCCACTTCAAGCTTCC 216507
                                v  vvi   i     v       vi  viv   i  v v   v  
C rnd-4_family-       1265 ATCTTATGACTGCGAATGTCTTCTGCAATCTTTTTCCATTTGATGCTGCC 1216

  contig10_pilo     216508 TCGGATCGTGAGGGCGCCTCGTCCTCGGATGTTTTCGGTCGCGATGGTGA 216557
                           vvvv i v vv v     i  v   ivv  i  i  i vv  v vvv   
C rnd-4_family-       1215 AGCTACCCTCTGCGCGCCCCGGCCTTTCATATTCTCAGAGGCCAGTTTGA 1166

  contig10_pilo     216558 GGTATGCCTTGGTCTTCTT 216576
                           vi  v        vi    
C rnd-4_family-       1165 TATAGGCCTTGGTGCTCTT 1147

Matrix = 20p53g.matrix
Kimura (with divCpGMod) = 48.87
Transitions / transversions = 0.70 (152/218)
Gap_init rate = 0.04 (45 / 1038), avg. gap size = 1.33 (60 / 45)

 
 

   +     225   25.2  8.6  3.7  contig10_pilon   217373  217500 (1597379) C  rnd-4_family-874   LINE/L1-Tx1     (5554)    671     538    73     
 
 ANNOTATION EVIDENCE: 
   225  25.16 8.59 3.73  contig10_pilon   217373  217500   1597379 C  rnd-4_family-874   LINE/L1-Tx1        538    671    5554      
225 25.16 8.59 3.73 contig10_pilon 217373 217500 (1597379) C rnd-4_family-874#LINE/L1-Tx1 (5554) 671 538 m_b498s001i6

  contig10_pilo     217373 TTCTCAT-GAACTTAAGTGTCTCCACACGGCGAGCCCT-GACCTAAATAG 217420
                             ii   - ii   v  i      i i    vv  i  - ii        
C rnd-4_family-        671 TTTCCATCGGGCTTCAGCGTCTCCGCGCGGCTCGCTCTTGGTCTAAATAG 622

  contig10_pilo     217421 GTCTCCGTTCCCGGATCTC-CT--TTCCTG---CCG--GGGCAGTATGTG 217462
                           ii     i  v ---  i -  --      ---   --  v     vi v
C rnd-4_family-        621 ACCTCCGCTCAC---TCCCGCTGCTTCCTGTCCCCGAAGGTCAGTAAATT 575

  contig10_pilo     217463 GGG-AGAACCGGGGTACTGGCTTGGAGGTTGTACGTAGC 217500
                           i  -  v vvi   i   i   v   --    i      
C rnd-4_family-        574 AGGCAGCAGGAGGGCACTAGCTAGGA--TTGTGCGTAGC 538

Matrix = 20p53g.matrix
Kimura (with divCpGMod) = 28.09
Transitions / transversions = 1.82 (20/11)
Gap_init rate = 0.09 (12 / 127), avg. gap size = 1.33 (16 / 12)

 
 
 
 
 
  +      14   11.2  3.5  0.0  contig10_pilon   225283  225311 (1589568) +  (TGGA)n            Simple_repeat        1     30     (0)    74     
 
 ANNOTATION EVIDENCE: 
    14  11.25 3.45 0.00  contig10_pilon   225283  225311   1589568 +  (TGGA)n            Simple_repeat        1     30       0      
14 11.25 3.45 0.00 contig10_pilon 225283 225311 (1589568) (TGGA)n#Simple_repeat 1 30 (0) m_b498s252i6

  contig10_pilo     225283 TGG-TGCATGGATAGATGGATGGATGGGTG 225311
                              -  v      i             i  
  (TGGA)n#Simpl          1 TGGATGGATGGATGGATGGATGGATGGATG 30

Matrix = Unknown
Transitions / transversions = 2.00 (2/1)
Gap_init rate = 0.04 (1 / 28), avg. gap size = 1.00 (1 / 1)

 
 

 
 
   +      14   24.7  3.3  3.3  contig10_pilon   228607  228618 (1586261) +  (GCAGCA)n          Simple_repeat        1     34    (27)    75     
 
 ANNOTATION EVIDENCE: 
    14  24.70 3.28 3.28  contig10_pilon   228607  228667   1586212 +  (GCAGCA)n          Simple_repeat        1     61       0      
14 24.70 3.28 3.28 contig10_pilon 228607 228667 (1586212) (GCAGCA)n#Simple_repeat 1 61 (0) m_b499s252i0

  contig10_pilo     228607 GCACCACCA-CAAGAGGAGAAGCAGCAGCAGCAGGAGGAGCTGGCTG-AG 228654
                              v  v  -  iv  v  v              v  v   -i  v -  
  (GCAGCA)n#Sim          1 GCAGCAGCAGCAGCAGCAGCAGCAGCAGCAGCAGCAGCAGC-AGCAGCAG 49

  contig10_pilo     228655 CAGGAGAACGCAG 228667
                              v  v -    
  (GCAGCA)n#Sim         50 CAGCAGCA-GCAG 61

Matrix = Unknown
Transitions / transversions = 0.20 (2/10)
Gap_init rate = 0.07 (4 / 60), avg. gap size = 1.00 (4 / 4)

 
 
 
 
  +      13   29.2  0.0  0.0  contig10_pilon   228619  228660 (1586219) +  GA-rich            Low_complexity       1     42     (0)    76     
 
 ANNOTATION EVIDENCE: 
    13  29.17 0.00 0.00  contig10_pilon   228619  228660   1586219 +  (GAG)n             Simple_repeat        1     42       0      
13 29.17 0.00 0.00 contig10_pilon 228619 228660 (1586219) (GAG)n#Simple_repeat 1 42 (0) m_b499s252i1

  contig10_pilo     228619 GAGGAGAAGCAGCAGCAGCAGGAGGAGCTGGCTGAGCAGGAG 228660
                                 i  v  v  v  v        vv  vv   v     
  (GAG)n#Simple          1 GAGGAGGAGGAGGAGGAGGAGGAGGAGGAGGAGGAGGAGGAG 42

Matrix = Unknown
Transitions / transversions = 0.11 (1/9)
Gap_init rate = 0.00 (0 / 41), avg. gap size = 0.0 (0 / 0)

 
 

 
   +      14   24.7  3.3  3.3  contig10_pilon   228661  228667 (1586212) +  (GCAGCA)n          Simple_repeat       35     61     (0)    75     
 
 ANNOTATION EVIDENCE: 
    14  24.70 3.28 3.28  contig10_pilon   228607  228667   1586212 +  (GCAGCA)n          Simple_repeat        1     61       0      
14 24.70 3.28 3.28 contig10_pilon 228607 228667 (1586212) (GCAGCA)n#Simple_repeat 1 61 (0) m_b499s252i0

  contig10_pilo     228607 GCACCACCA-CAAGAGGAGAAGCAGCAGCAGCAGGAGGAGCTGGCTG-AG 228654
                              v  v  -  iv  v  v              v  v   -i  v -  
  (GCAGCA)n#Sim          1 GCAGCAGCAGCAGCAGCAGCAGCAGCAGCAGCAGCAGCAGC-AGCAGCAG 49

  contig10_pilo     228655 CAGGAGAACGCAG 228667
                              v  v -    
  (GCAGCA)n#Sim         50 CAGCAGCA-GCAG 61

Matrix = Unknown
Transitions / transversions = 0.20 (2/10)
Gap_init rate = 0.07 (4 / 60), avg. gap size = 1.00 (4 / 4)

 
 
 
 
 
  +      13   22.1  0.0  0.0  contig10_pilon   237515  237546 (1577333) +  (GCC)n             Simple_repeat        1     32     (0)    77     
 
 ANNOTATION EVIDENCE: 
    13  22.07 0.00 0.00  contig10_pilon   237515  237546   1577333 +  (GCC)n             Simple_repeat        1     32       0      
13 22.07 0.00 0.00 contig10_pilon 237515 237546 (1577333) (GCC)n#Simple_repeat 1 32 (0) m_b499s252i2

  contig10_pilo     237515 GCCGTCGCCGTCGTCGTCGCCGCCTGCGCCGC 237546
                               i     i  i  i       vv      
  (GCC)n#Simple          1 GCCGCCGCCGCCGCCGCCGCCGCCGCCGCCGC 32

Matrix = Unknown
Transitions / transversions = 2.00 (4/2)
Gap_init rate = 0.00 (0 / 31), avg. gap size = 0.0 (0 / 0)

 
 

 
 
   +      16    7.8  0.0  3.6  contig10_pilon   238187  238215 (1576664) +  (TGCG)n            Simple_repeat        1     28     (0)    78     
 
 ANNOTATION EVIDENCE: 
    16   7.84 0.00 3.57  contig10_pilon   238187  238215   1576664 +  (TGCG)n            Simple_repeat        1     28       0      
16 7.84 0.00 3.57 contig10_pilon 238187 238215 (1576664) (TGCG)n#Simple_repeat 1 28 (0) m_b499s252i3

  contig10_pilo     238187 TGCGTGCCGTGCGTGCGTGCGGGCTTGCG 238215
                                 -              v  v    
  (TGCG)n#Simpl          1 TGCGTG-CGTGCGTGCGTGCGTGCGTGCG 28

Matrix = Unknown
Transitions / transversions = 0.00 (0/2)
Gap_init rate = 0.04 (1 / 28), avg. gap size = 1.00 (1 / 1)

 
 
 
 
 
  +      15   15.3  0.0  0.0  contig10_pilon   250340  250368 (1564511) +  (ACG)n             Simple_repeat        1     29     (0)    79     
 
 ANNOTATION EVIDENCE: 
    15  15.26 0.00 0.00  contig10_pilon   250340  250368   1564511 +  (ACG)n             Simple_repeat        1     29       0      
15 15.26 0.00 0.00 contig10_pilon 250340 250368 (1564511) (ACG)n#Simple_repeat 1 29 (0) m_b499s252i4

  contig10_pilo     250340 ACGACGTCGGCGACGACGACGACCAGGAC 250368
                                 v  i             v v   
  (ACG)n#Simple          1 ACGACGACGACGACGACGACGACGACGAC 29

Matrix = Unknown
Transitions / transversions = 0.33 (1/3)
Gap_init rate = 0.00 (0 / 28), avg. gap size = 0.0 (0 / 0)

 
 

 
 
   +      14   13.6  4.5  4.5  contig10_pilon   251248  251291 (1563588) +  (TCCTGC)n          Simple_repeat        1     44     (0)    80     
 
 ANNOTATION EVIDENCE: 
    14  13.61 4.55 4.55  contig10_pilon   251248  251291   1563588 +  (TCCTGC)n          Simple_repeat        1     44       0      
14 13.61 4.55 4.55 contig10_pilon 251248 251291 (1563588) (TCCTGC)n#Simple_repeat 1 44 (0) m_b499s252i5

  contig10_pilo     251248 TCCTGCTCCTGCCTCGTGCTGC-CCT-CTGCCTCGCGCTCCTGCTC 251291
                                      -   v    v -v  -   -   vi          
  (TCCTGC)n#Sim          1 TCCTGCTCCTG-CTCCTGCTCCTGCTCCTG-CTCCTGCTCCTGCTC 44

Matrix = Unknown
Transitions / transversions = 0.25 (1/4)
Gap_init rate = 0.09 (4 / 43), avg. gap size = 1.00 (4 / 4)

 
 
 
 
 
  +      19   17.4  0.0  7.3  contig10_pilon   253805  253863 (1561016) +  (TCTCC)n           Simple_repeat        1     55     (0)    81     
 
 ANNOTATION EVIDENCE: 
    19  17.44 0.00 7.27  contig10_pilon   253805  253863   1561016 +  (TCTCC)n           Simple_repeat        1     55       0      
19 17.44 0.00 7.27 contig10_pilon 253805 253863 (1561016) (TCTCC)n#Simple_repeat 1 55 (0) m_b499s252i6

  contig10_pilo     253805 TCTCCGTCTCCGCTCCTCACCTCTCCTCTCCCCATGCCATGACTCTCCGT 253854
                                -     v      v            i - -  vivv      - 
  (TCTCC)n#Simp          1 TCTCC-TCTCCTCTCCTCTCCTCTCCTCTCCTC-T-CCTCTCCTCTCC-T 46

  contig10_pilo     253855 CTCCTCCCC 253863
                                 i  
  (TCTCC)n#Simp         47 CTCCTCTCC 55

Matrix = Unknown
Transitions / transversions = 0.60 (3/5)
Gap_init rate = 0.07 (4 / 58), avg. gap size = 1.00 (4 / 4)

 
 

 
 
   +      12   17.6  5.0  2.4  contig10_pilon   260640  260679 (1554200) +  (CGC)n             Simple_repeat        1     41     (0)    82     
 
 ANNOTATION EVIDENCE: 
    12  17.65 5.00 2.44  contig10_pilon   260640  260679   1554200 +  (CGC)n             Simple_repeat        1     41       0      
12 17.65 5.00 2.44 contig10_pilon 260640 260679 (1554200) (CGC)n#Simple_repeat 1 41 (0) m_b499s252i7

  contig10_pilo     260640 CGCTGCCG-AGCCCCCG-AGCGCGCCGCCGCCGCCACCGACG 260679
                              i    -v   v   -v  -             i   v  
  (CGC)n#Simple          1 CGCCGCCGCCGCCGCCGCCGC-CGCCGCCGCCGCCGCCGCCG 41

Matrix = Unknown
Transitions / transversions = 0.50 (2/4)
Gap_init rate = 0.08 (3 / 39), avg. gap size = 1.00 (3 / 3)

 
 
 
 
 
  +      22   23.4  4.1  4.1  contig10_pilon   268498  268503 (1546376) +  (CCGACG)n          Simple_repeat        1     18    (79)    83     
 
 ANNOTATION EVIDENCE: 
    22  23.36 4.12 4.12  contig10_pilon   268498  268594   1546285 +  (CCGACG)n          Simple_repeat        1     97       0      
22 23.36 4.12 4.12 contig10_pilon 268498 268594 (1546285) (CCGACG)n#Simple_repeat 1 97 (0) m_b499s252i8

  contig10_pilo     268498 CCGCCG-CGACACCGCCGCCGACGCCGACGACGACGACGACGAGCTCGAC 268546
                              v  -    i   v              v     v    --  -    
  (CCGACG)n#Sim          1 CCGACGCCGACGCCGACGCCGACGCCGACGCCGACGCCGAC--GC-CGAC 47

  contig10_pilo     268547 -CAGACATTCGCCGACGACGCC-ACGACGCTGCCCAAGCTG-CGCGGGCG 268593
                           - v   -vi  v  v       -   v  vi   v v  i -   v i  
  (CCGACG)n#Sim         48 GCCGAC-GCCGACGCCGACGCCGACGCCGACGCCGACGCCGACGCCGACG 96

  contig10_pilo     268594 C 268594
                            
  (CCGACG)n#Sim         97 C 97

Matrix = Unknown
Transitions / transversions = 0.38 (5/13)
Gap_init rate = 0.08 (8 / 96), avg. gap size = 1.00 (8 / 8)

 
 

 
   +      24   22.3  2.9  1.4  contig10_pilon   268504  268573 (1546306) +  (CGA)n             Simple_repeat        1     71     (0)    84     
 
 ANNOTATION EVIDENCE: 
    24  22.33 2.86 1.41  contig10_pilon   268504  268573   1546306 +  (CGA)n             Simple_repeat        1     71       0      
24 22.33 2.86 1.41 contig10_pilon 268504 268573 (1546306) (CGA)n#Simple_repeat 1 71 (0) m_b499s252i9

  contig10_pilo     268504 CGACACCGCCGCCGACGCCGACGACGACGACGACGAGCTCGACCA-GACA 268552
                               iv  v  v     v                  vvv    v -   -
  (CGA)n#Simple          1 CGACGACGACGACGACGACGACGACGACGACGACGACGACGACGACGAC- 49

  contig10_pilo     268553 TTCGCCGACGACG-CCACGACG 268573
                           vv  v        - v      
  (CGA)n#Simple         50 GACGACGACGACGACGACGACG 71

Matrix = Unknown
Transitions / transversions = 0.08 (1/12)
Gap_init rate = 0.04 (3 / 69), avg. gap size = 1.00 (3 / 3)

 
 
 
 
  +      22   23.4  4.1  4.1  contig10_pilon   268574  268594 (1546285) +  (CCGACG)n          Simple_repeat       19     97     (0)    83     
 
 ANNOTATION EVIDENCE: 
    22  23.36 4.12 4.12  contig10_pilon   268498  268594   1546285 +  (CCGACG)n          Simple_repeat        1     97       0      
22 23.36 4.12 4.12 contig10_pilon 268498 268594 (1546285) (CCGACG)n#Simple_repeat 1 97 (0) m_b499s252i8

  contig10_pilo     268498 CCGCCG-CGACACCGCCGCCGACGCCGACGACGACGACGACGAGCTCGAC 268546
                              v  -    i   v              v     v    --  -    
  (CCGACG)n#Sim          1 CCGACGCCGACGCCGACGCCGACGCCGACGCCGACGCCGAC--GC-CGAC 47

  contig10_pilo     268547 -CAGACATTCGCCGACGACGCC-ACGACGCTGCCCAAGCTG-CGCGGGCG 268593
                           - v   -vi  v  v       -   v  vi   v v  i -   v i  
  (CCGACG)n#Sim         48 GCCGAC-GCCGACGCCGACGCCGACGCCGACGCCGACGCCGACGCCGACG 96

  contig10_pilo     268594 C 268594
                            
  (CCGACG)n#Sim         97 C 97

Matrix = Unknown
Transitions / transversions = 0.38 (5/13)
Gap_init rate = 0.08 (8 / 96), avg. gap size = 1.00 (8 / 8)

 
 

 
 
   +      13   14.3  3.0  3.0  contig10_pilon   272931  272963 (1541916) +  (ATC)n             Simple_repeat        1     33     (0)    85     
 
 ANNOTATION EVIDENCE: 
    13  14.30 3.03 3.03  contig10_pilon   272931  272963   1541916 +  (ATC)n             Simple_repeat        1     33       0      
13 14.30 3.03 3.03 contig10_pilon 272931 272963 (1541916) (ATC)n#Simple_repeat 1 33 (0) m_b499s252i10

  contig10_pilo     272931 ATCATCCTCA-CACTCTTCATCAGCATAATCATC 272963
                                 v   -  -  v      v   v      
  (ATC)n#Simple          1 ATCATCATCATCA-TCATCATCATCATCATCATC 33

Matrix = Unknown
Transitions / transversions = 0.00 (0/4)
Gap_init rate = 0.06 (2 / 32), avg. gap size = 1.00 (2 / 2)

 
 
 
 
 
  +      12   27.3  2.0  2.0  contig10_pilon   287730  287779 (1527100) +  (CGA)n             Simple_repeat        1     50     (0)    86     
 
 ANNOTATION EVIDENCE: 
    12  27.34 2.00 2.00  contig10_pilon   287730  287779   1527100 +  (CGA)n             Simple_repeat        1     50       0      
12 27.34 2.00 2.00 contig10_pilon 287730 287779 (1527100) (CGA)n#Simple_repeat 1 50 (0) m_b500s252i0

  contig10_pilo     287730 CGACGGCGAGGACGACGACGA-GCCTGAGGATGATGCCAACGCCAAGGAC 287778
                                i   v           - v -  v  i  i v i   v i v   
  (CGA)n#Simple          1 CGACGACGACGACGACGACGACGAC-GACGACGACGACGACGACGACGAC 49

  contig10_pilo     287779 G 287779
                            
  (CGA)n#Simple         50 G 50

Matrix = Unknown
Transitions / transversions = 0.83 (5/6)
Gap_init rate = 0.04 (2 / 49), avg. gap size = 1.00 (2 / 2)

 
 

 
 
   +      14    0.0  0.0  0.0  contig10_pilon   293893  293910 (1520969) +  (TCG)n             Simple_repeat        1     18     (0)    87     
 
 ANNOTATION EVIDENCE: 
    14   0.00 0.00 0.00  contig10_pilon   293893  293910   1520969 +  (TCG)n             Simple_repeat        1     18       0      
14 0.00 0.00 0.00 contig10_pilon 293893 293910 (1520969) (TCG)n#Simple_repeat 1 18 (0) m_b500s252i1

  contig10_pilo     293893 TCGTCGTCGTCGTCGTCG 293910
                                             
  (TCG)n#Simple          1 TCGTCGTCGTCGTCGTCG 18

Matrix = Unknown
Transitions / transversions = 1.00 (0/0)
Gap_init rate = 0.00 (0 / 17), avg. gap size = 0.0 (0 / 0)

 
 
 
 
 
  +      13   17.5  6.2  6.2  contig10_pilon   294774  294838 (1520041) +  (GGTGCGC)n         Simple_repeat        1     65     (0)    88     
 
 ANNOTATION EVIDENCE: 
    13  17.54 6.15 6.15  contig10_pilon   294774  294838   1520041 +  (GGTGCGC)n         Simple_repeat        1     65       0      
13 17.54 6.15 6.15 contig10_pilon 294774 294838 (1520041) (GGTGCGC)n#Simple_repeat 1 65 (0) m_b500s252i2

  contig10_pilo     294774 GGTG-GAGGAGCGCGG-GCGCGGTGCGC-GTGGACAGGTTGGCGCGCTGC 294820
                               - v  v      -           -   vi -  - -     v
[truncated: 32,722,803 more chars]
