# Supplementary material for: Comparative Genomic Analysis of Two Monokaryons of Auricularia heimuer Hei29
Source: J Fungi (Basel). 2025 Feb 6;11(2):122. doi: 10.3390/jof11020122 (PMC11856363; doi:10.3390/jof11020122)
Supplement: Supplementary file 1 [file jof-11-00122-s001.zip › Hei29-D2Repeat.html]

 


 


 

 [ close all hsps ] 
 [ open all hsps ] 
 
 
  
                                          ----position in query----                                    --position in repeat--
            %      %      %      query                                       C   matching            repeat           (left)     end      begin    linkage 
  +    score     div.   del.   ins.    sequence           begin      end      (left)     +   repeat              class/family      begin     end     (left)    id/graphic  
 
 
 
  +      12   18.3  0.0  3.1  contig10_pilon     3499    3531 (1849148) +  (TTTC)n            Simple_repeat        1     32     (0)     1     
 
 ANNOTATION EVIDENCE: 
    12  18.29 0.00 3.12  contig10_pilon     3499    3531   1849148 +  (TTTC)n            Simple_repeat        1     32       0      
12 18.29 0.00 3.12 contig10_pilon 3499 3531 (1849148) (TTTC)n#Simple_repeat 1 32 (0) m_b684s252i0

  contig10_pilo       3499 TTTTTTTCTGATTTATTTTTCTTTCTTTCTTTC 3531
                              i     -v i v i                
  (TTTC)n#Simpl          1 TTTCTTTCT-TTCTTTCTTTCTTTCTTTCTTTC 32

Matrix = Unknown
Transitions / transversions = 1.50 (3/2)
Gap_init rate = 0.03 (1 / 32), avg. gap size = 1.00 (1 / 1)

 
 

 
 
   +      13   24.1  1.7  5.3  contig10_pilon     3657    3715 (1848964) +  (CCGCGC)n          Simple_repeat        1     57     (0)     2     
 
 ANNOTATION EVIDENCE: 
    13  24.12 1.69 5.26  contig10_pilon     3657    3715   1848964 +  (CCGCGC)n          Simple_repeat        1     57       0      
13 24.12 1.69 5.26 contig10_pilon 3657 3715 (1848964) (CCGCGC)n#Simple_repeat 1 57 (0) m_b684s252i1

  contig10_pilo       3657 CCTCGCACTCGCCGAGCACCCGCGCGCCAACGTCCGCGCCCACGCGCTCG 3706
                             v   v v    -v  i       -  -i  i        i   v v  
  (CCGCGC)n#Sim          1 CCGCGCCCGCGCC-CGCGCCCGCGC-CC-GCGCCCGCGCCCGCGCCCGCG 47

  contig10_pilo       3707 -CCGCGCTCG 3715
                           -      i  
  (CCGCGC)n#Sim         48 CCCGCGCCCG 57

Matrix = Unknown
Transitions / transversions = 0.83 (5/6)
Gap_init rate = 0.07 (4 / 58), avg. gap size = 1.00 (4 / 4)

 
 
 
 
 
  +      12    4.9  0.0  4.5  contig10_pilon     4942    4964 (1847715) +  (TGCA)n            Simple_repeat        1     22     (0)     3     
 
 ANNOTATION EVIDENCE: 
    12   4.93 0.00 4.55  contig10_pilon     4942    4964   1847715 +  (TGCA)n            Simple_repeat        1     22       0      
12 4.93 0.00 4.55 contig10_pilon 4942 4964 (1847715) (TGCA)n#Simple_repeat 1 22 (0) m_b684s252i2

  contig10_pilo       4942 TGCATGCTTGCATGCATGGCATG 4964
                                  v         -     
  (TGCA)n#Simpl          1 TGCATGCATGCATGCAT-GCATG 22

Matrix = Unknown
Transitions / transversions = 0.00 (0/1)
Gap_init rate = 0.05 (1 / 22), avg. gap size = 1.00 (1 / 1)

 
 

 
 
   +      12   18.3  0.0  3.1  contig10_pilon    12144   12176 (1840503) +  (TTTC)n            Simple_repeat        1     32     (0)     4     
 
 ANNOTATION EVIDENCE: 
    12  18.29 0.00 3.12  contig10_pilon    12144   12176   1840503 +  (TTTC)n            Simple_repeat        1     32       0      
12 18.29 0.00 3.12 contig10_pilon 12144 12176 (1840503) (TTTC)n#Simple_repeat 1 32 (0) m_b684s252i3

  contig10_pilo      12144 TTTTTTTCTGATTTATTTTTCTTTCTTTCTTTC 12176
                              i     -v i v i                
  (TTTC)n#Simpl          1 TTTCTTTCT-TTCTTTCTTTCTTTCTTTCTTTC 32

Matrix = Unknown
Transitions / transversions = 1.50 (3/2)
Gap_init rate = 0.03 (1 / 32), avg. gap size = 1.00 (1 / 1)

 
 
 
 
 
  +      13   24.1  1.7  5.3  contig10_pilon    12302   12360 (1840319) +  (CCGCGC)n          Simple_repeat        1     57     (0)     5     
 
 ANNOTATION EVIDENCE: 
    13  24.12 1.69 5.26  contig10_pilon    12302   12360   1840319 +  (CCGCGC)n          Simple_repeat        1     57       0      
13 24.12 1.69 5.26 contig10_pilon 12302 12360 (1840319) (CCGCGC)n#Simple_repeat 1 57 (0) m_b684s252i4

  contig10_pilo      12302 CCTCGCACTCGCCGAGCACCCGCGCGCCAACGTCCGCGCCCACGCGCTCG 12351
                             v   v v    -v  i       -  -i  i        i   v v  
  (CCGCGC)n#Sim          1 CCGCGCCCGCGCC-CGCGCCCGCGC-CC-GCGCCCGCGCCCGCGCCCGCG 47

  contig10_pilo      12352 -CCGCGCTCG 12360
                           -      i  
  (CCGCGC)n#Sim         48 CCCGCGCCCG 57

Matrix = Unknown
Transitions / transversions = 0.83 (5/6)
Gap_init rate = 0.07 (4 / 58), avg. gap size = 1.00 (4 / 4)

 
 

 
 
   +      12    4.9  0.0  4.5  contig10_pilon    13587   13609 (1839070) +  (TGCA)n            Simple_repeat        1     22     (0)     6     
 
 ANNOTATION EVIDENCE: 
    12   4.93 0.00 4.55  contig10_pilon    13587   13609   1839070 +  (TGCA)n            Simple_repeat        1     22       0      
12 4.93 0.00 4.55 contig10_pilon 13587 13609 (1839070) (TGCA)n#Simple_repeat 1 22 (0) m_b684s252i5

  contig10_pilo      13587 TGCATGCTTGCATGCATGGCATG 13609
                                  v         -     
  (TGCA)n#Simpl          1 TGCATGCATGCATGCAT-GCATG 22

Matrix = Unknown
Transitions / transversions = 0.00 (0/1)
Gap_init rate = 0.05 (1 / 22), avg. gap size = 1.00 (1 / 1)

 
 
 
 
 
  +      14    4.1  3.7  3.7  contig10_pilon    20945   20971 (1831708) +  (CGC)n             Simple_repeat        1     27     (0)     7     
 
 ANNOTATION EVIDENCE: 
    14   4.11 3.70 3.70  contig10_pilon    20945   20971   1831708 +  (CGC)n             Simple_repeat        1     27       0      
14 4.11 3.70 3.70 contig10_pilon 20945 20971 (1831708) (CGC)n#Simple_repeat 1 27 (0) m_b684s252i6

  contig10_pilo      20945 CGCCGCCGCCGCCGACGCC-CCGCACGC 20971
                                         v    -    -   
  (CGC)n#Simple          1 CGCCGCCGCCGCCGCCGCCGCCGC-CGC 27

Matrix = Unknown
Transitions / transversions = 0.00 (0/1)
Gap_init rate = 0.08 (2 / 26), avg. gap size = 1.00 (2 / 2)

 
 

 
 
   +      12   18.3  0.0  3.1  contig10_pilon    21521   21553 (1831126) +  (TTTC)n            Simple_repeat        1     32     (0)     8     
 
 ANNOTATION EVIDENCE: 
    12  18.29 0.00 3.12  contig10_pilon    21521   21553   1831126 +  (TTTC)n            Simple_repeat        1     32       0      
12 18.29 0.00 3.12 contig10_pilon 21521 21553 (1831126) (TTTC)n#Simple_repeat 1 32 (0) m_b684s252i7

  contig10_pilo      21521 TTTTTTTCTGATTTATTTTTCTTTCTTTCTTTC 21553
                              i     -v i v i                
  (TTTC)n#Simpl          1 TTTCTTTCT-TTCTTTCTTTCTTTCTTTCTTTC 32

Matrix = Unknown
Transitions / transversions = 1.50 (3/2)
Gap_init rate = 0.03 (1 / 32), avg. gap size = 1.00 (1 / 1)

 
 
 
 
 
  +      13   24.1  1.7  5.3  contig10_pilon    21679   21737 (1830942) +  (CCGCGC)n          Simple_repeat        1     57     (0)     9     
 
 ANNOTATION EVIDENCE: 
    13  24.12 1.69 5.26  contig10_pilon    21679   21737   1830942 +  (CCGCGC)n          Simple_repeat        1     57       0      
13 24.12 1.69 5.26 contig10_pilon 21679 21737 (1830942) (CCGCGC)n#Simple_repeat 1 57 (0) m_b684s252i8

  contig10_pilo      21679 CCTCGCACTCGCCGAGCACCCGCGCGCCAACGTCCGCGCCCACGCGCTCG 21728
                             v   v v    -v  i       -  -i  i        i   v v  
  (CCGCGC)n#Sim          1 CCGCGCCCGCGCC-CGCGCCCGCGC-CC-GCGCCCGCGCCCGCGCCCGCG 47

  contig10_pilo      21729 -CCGCGCTCG 21737
                           -      i  
  (CCGCGC)n#Sim         48 CCCGCGCCCG 57

Matrix = Unknown
Transitions / transversions = 0.83 (5/6)
Gap_init rate = 0.07 (4 / 58), avg. gap size = 1.00 (4 / 4)

 
 

 
 
   +      25   17.8  0.0  0.0  contig10_pilon    22226   22276 (1830403) +  (GACGAT)n          Simple_repeat        1     51     (0)    10     
 
 ANNOTATION EVIDENCE: 
    25  17.84 0.00 0.00  contig10_pilon    22226   22276   1830403 +  (GACGAT)n          Simple_repeat        1     51       0      
25 17.84 0.00 0.00 contig10_pilon 22226 22276 (1830403) (GACGAT)n#Simple_repeat 1 51 (0) m_b684s252i9

  contig10_pilo      22226 GACGATGATGATGATGATTACGATGACGATGAAGACTATGACGACGATGA 22275
                                   i     i   v             v  iv i  i        
  (GACGAT)n#Sim          1 GACGATGACGATGACGATGACGATGACGATGACGATGACGATGACGATGA 50

  contig10_pilo      22276 C 22276
                            
  (GACGAT)n#Sim         51 C 51

Matrix = Unknown
Transitions / transversions = 1.67 (5/3)
Gap_init rate = 0.00 (0 / 50), avg. gap size = 0.0 (0 / 0)

 
 
 
 
 
  +      13   10.3  6.1  8.3  contig10_pilon    22473   22521 (1830158) +  (CCTTT)n           Simple_repeat        1     48     (0)    11     
 
 ANNOTATION EVIDENCE: 
    13  10.33 6.12 8.33  contig10_pilon    22473   22521   1830158 +  (CCTTT)n           Simple_repeat        1     48       0      
13 10.33 6.12 8.33 contig10_pilon 22473 22521 (1830158) (CCTTT)n#Simple_repeat 1 48 (0) m_b684s252i10

  contig10_pilo      22473 CCTTGCGCTTT-CTTTCCTATCCTGATGCCCGTTCC-TT-CTTTCCTTTC 22519
                               v -    -       v    -- -i  v    -  -          
  (CCTTT)n#Simp          1 CCTTTC-CTTTCCTTTCCTTTCCT--T-TCCTTTCCTTTCCTTTCCTTTC 46

  contig10_pilo      22520 CT 22521
                             
  (CCTTT)n#Simp         47 CT 48

Matrix = Unknown
Transitions / transversions = 0.33 (1/3)
Gap_init rate = 0.15 (7 / 48), avg. gap size = 1.00 (7 / 7)

 
 

 
 
   +      17    5.0  0.0  0.0  contig10_pilon    24193   24213 (1828466) +  (TC)n              Simple_repeat        1     21     (0)    12     
 
 ANNOTATION EVIDENCE: 
    17   5.00 0.00 0.00  contig10_pilon    24193   24213   1828466 +  (TC)n              Simple_repeat        1     21       0      
17 5.00 0.00 0.00 contig10_pilon 24193 24213 (1828466) (TC)n#Simple_repeat 1 21 (0) m_b684s252i11

  contig10_pilo      24193 TCTCTCTCTCTCCCTCTCTCT 24213
                                       i        
  (TC)n#Simple_          1 TCTCTCTCTCTCTCTCTCTCT 21

Matrix = Unknown
Transitions / transversions = 1.00 (1/0)
Gap_init rate = 0.00 (0 / 20), avg. gap size = 0.0 (0 / 0)

 
 
 
 
 
  +      29   20.5  1.5  0.0  contig10_pilon    29620   29686 (1822993) +  GA-rich            Low_complexity       1     68     (0)    13     
 
 ANNOTATION EVIDENCE: 
    29  20.47 1.49 0.00  contig10_pilon    29620   29686   1822993 +  (GGA)n             Simple_repeat        1     68       0      
29 20.47 1.49 0.00 contig10_pilon 29620 29686 (1822993) (GGA)n#Simple_repeat 1 68 (0) m_b684s252i12

  contig10_pilo      29620 GGACGACGAGGAGGGCGAGGAGGGCGAGGAGGAGGCGGAGAGGGAGGAGG 29669
                              v  v       iv       iv          v    ii        
  (GGA)n#Simple          1 GGAGGAGGAGGAGGAGGAGGAGGAGGAGGAGGAGGAGGAGGAGGAGGAGG 50

  contig10_pilo      29670 AGGAGG-CGAGGCGGTGG 29686
                                 -v    v  v  
  (GGA)n#Simple         51 AGGAGGAGGAGGAGGAGG 68

Matrix = Unknown
Transitions / transversions = 0.50 (4/8)
Gap_init rate = 0.02 (1 / 66), avg. gap size = 1.00 (1 / 1)

 
 

 
 
   +      20   17.2  3.7  1.8  contig10_pilon    46109   46162 (1806517) +  (CGAGC)n           Simple_repeat        1     55     (0)    14     
 
 ANNOTATION EVIDENCE: 
    20  17.18 3.70 1.82  contig10_pilon    46109   46162   1806517 +  (CGAGC)n           Simple_repeat        1     55       0      
20 17.18 3.70 1.82 contig10_pilon 46109 46162 (1806517) (CGAGC)n#Simple_repeat 1 55 (0) m_b684s252i13

  contig10_pilo      46109 CGAGCCG-GCCGAGCCGAGCCGTTTGTAGGCCGAGCCTAGCCGGGCCG-T 46156
                                  -              vvivv -        v     i    -v
  (CGAGC)n#Simp          1 CGAGCCGAGCCGAGCCGAGCCGAGCCGA-GCCGAGCCGAGCCGAGCCGAG 49

  contig10_pilo      46157 CCGAGC 46162
                                 
  (CGAGC)n#Simp         50 CCGAGC 55

Matrix = Unknown
Transitions / transversions = 0.33 (2/6)
Gap_init rate = 0.06 (3 / 53), avg. gap size = 1.00 (3 / 3)

 
 
 
 
 
  +      13   23.1  2.3  2.3  contig10_pilon    48233   48275 (1804404) +  (AGC)n             Simple_repeat        1     43     (0)    15     
 
 ANNOTATION EVIDENCE: 
    13  23.09 2.33 2.33  contig10_pilon    48233   48275   1804404 +  (AGC)n             Simple_repeat        1     43       0      
13 23.09 2.33 2.33 contig10_pilon 48233 48275 (1804404) (AGC)n#Simple_repeat 1 43 (0) m_b684s252i14

  contig10_pilo      48233 AGCAGCAGCAGCACGC-GCAGGTGGATCATCATCATCAGAAGCA 48275
                                        -  -    vv v v  v  v  v   v    
  (AGC)n#Simple          1 AGCAGCAGCAGCA-GCAGCAGCAGCAGCAGCAGCAGCAGCAGCA 43

Matrix = Unknown
Transitions / transversions = 0.00 (0/8)
Gap_init rate = 0.05 (2 / 42), avg. gap size = 1.00 (2 / 2)

 
 

 
 
   +      16   14.2  0.0  0.0  contig10_pilon    54112   54142 (1798537) +  (GAGCGG)n          Simple_repeat        1     31     (0)    16     
 
 ANNOTATION EVIDENCE: 
    16  14.21 0.00 0.00  contig10_pilon    54112   54142   1798537 +  (GAGCGG)n          Simple_repeat        1     31       0      
16 14.21 0.00 0.00 contig10_pilon 54112 54142 (1798537) (GAGCGG)n#Simple_repeat 1 31 (0) m_b684s252i15

  contig10_pilo      54112 GAGCGGGAGCGGGAGCAGCACTGGGAGCGGG 54142
                                           i v vi         
  (GAGCGG)n#Sim          1 GAGCGGGAGCGGGAGCGGGAGCGGGAGCGGG 31

Matrix = Unknown
Transitions / transversions = 1.00 (2/2)
Gap_init rate = 0.00 (0 / 30), avg. gap size = 0.0 (0 / 0)

 
 
 
 
 
  +      26   11.2  6.8  6.8  contig10_pilon    54569   54656 (1798023) +  (CGACAG)n          Simple_repeat        1     88     (0)    17     
 
 ANNOTATION EVIDENCE: 
    26  11.21 6.82 6.82  contig10_pilon    54569   54656   1798023 +  (CGACAG)n          Simple_repeat        1     88       0      
26 11.21 6.82 6.82 contig10_pilon 54569 54656 (1798023) (CGACAG)n#Simple_repeat 1 88 (0) m_b684s252i16

  contig10_pilo      54569 CGACAGCGACAGCGACA-C-CCAGCGGCAGCG-CAGGCGCGAGCGGC-GC 54614
                                            - -v     i     -  -   vv    i -  
  (CGACAG)n#Sim          1 CGACAGCGACAGCGACAGCGACAGCGACAGCGACA-GCGACAGCGACAGC 49

  contig10_pilo      54615 GAGCAGCAGGAGCAG-GAGCAGC-AGAGCGACCGCAACAGCGAC 54656
                             -    --  -   -  -    - v      v  i        
  (CGACAG)n#Sim         50 GA-CAGC--GA-CAGCGA-CAGCGACAGCGACAGCGACAGCGAC 88

Matrix = Unknown
Transitions / transversions = 0.60 (3/5)
Gap_init rate = 0.14 (12 / 87), avg. gap size = 1.00 (12 / 12)

 
 

 
 
   +      15   22.5  2.3  2.3  contig10_pilon    54705   54747 (1797932) +  (CG)n              Simple_repeat        1     43     (0)    18     
 
 ANNOTATION EVIDENCE: 
    15  22.54 2.33 2.33  contig10_pilon    54705   54747   1797932 +  (CG)n              Simple_repeat        1     43       0      
15 22.54 2.33 2.33 contig10_pilon 54705 54747 (1797932) (CG)n#Simple_repeat 1 43 (0) m_b684s252i17

  contig10_pilo      54705 CGCGGCCGCGCGAGC-CCCGTCGCGCACGAGGACGCGCGCGCGC 54747
                               vv      v  - v  -     i  v vi           
  (CG)n#Simple_          1 CGCGCGCGCGCGCGCGCGCG-CGCGCGCGCGCGCGCGCGCGCGC 43

Matrix = Unknown
Transitions / transversions = 0.33 (2/6)
Gap_init rate = 0.05 (2 / 42), avg. gap size = 1.00 (2 / 2)

 
 
 
 
 
  +      21   18.1  3.9  1.9  contig10_pilon    55201   55252 (1797427) +  (CG)n              Simple_repeat        1     53     (0)    19     
 
 ANNOTATION EVIDENCE: 
    21  18.07 3.85 1.89  contig10_pilon    55201   55252   1797427 +  (CG)n              Simple_repeat        1     53       0      
21 18.07 3.85 1.89 contig10_pilon 55201 55252 (1797427) (CG)n#Simple_repeat 1 53 (0) m_b684s252i18

  contig10_pilo      55201 CGCCCCCGC-CGCCCGCGC-CGCGCGTCCTCGTCGCGCTCGCCCGCGCGC 55248
                              v v   -   v     -      iv v  -     v   v       
  (CG)n#Simple_          1 CGCGCGCGCGCGCGCGCGCGCGCGCGCGCGCG-CGCGCGCGCGCGCGCGC 49

  contig10_pilo      55249 GCGC 55252
                               
  (CG)n#Simple_         50 GCGC 53

Matrix = Unknown
Transitions / transversions = 0.14 (1/7)
Gap_init rate = 0.06 (3 / 51), avg. gap size = 1.00 (3 / 3)

 
 

 
 
   +      16   14.7  0.0  0.0  contig10_pilon    56291   56320 (1796359) +  (CGC)n             Simple_repeat        1     30     (0)    20     
 
 ANNOTATION EVIDENCE: 
    16  14.73 0.00 0.00  contig10_pilon    56291   56320   1796359 +  (CGC)n             Simple_repeat        1     30       0      
16 14.73 0.00 0.00 contig10_pilon 56291 56320 (1796359) (CGC)n#Simple_repeat 1 30 (0) m_b684s252i19

  contig10_pilo      56291 CGCCGCGAGCGCCGCTGCCGCCGCCGCCGC 56320
                                 viv      i              
  (CGC)n#Simple          1 CGCCGCCGCCGCCGCCGCCGCCGCCGCCGC 30

Matrix = Unknown
Transitions / transversions = 1.00 (2/2)
Gap_init rate = 0.00 (0 / 29), avg. gap size = 0.0 (0 / 0)

 
 
 
 
 
  +     464   27.4  3.4  0.7  contig10_pilon    65799   65945 (1786734) C  rnd-4_family-1001  LINE           (12529)   4203    4053    21 *   
 
 ANNOTATION EVIDENCE: 
   464  27.39 3.40 0.66  contig10_pilon    65799   65945   1786734 C  rnd-4_family-1001  LINE              4053   4203   12529      
464 27.39 3.40 0.66 contig10_pilon 65799 65945 (1786734) C rnd-4_family-1001#LINE (12529) 4203 4053 m_b685s001i0

  contig10_pilo      65799 TTGCCCAGGCATACTGTAAAGCTAATAAT-TATTACCGCGTCACGCGTCA 65847
                              i  v i         v-  vv i i -             vi     
C rnd-4_family-       4203 TTGTCCTGACATACTGTAC-GCACACAGTCTATTACCGCGTCAAACGTCA 4155

  contig10_pilo      65848 CGGAGGCCTCATGGCCCGGTAATAAACACGTTTTGTAGCATGTCACGGGC 65897
                            i    i v ii   v    i      vi        i  v     ii  
C rnd-4_family-       4154 CAGAGGTCGCGCGGCGCGGTGATAAACCTGTTTTGTAACAGGTCACAAGC 4105

  contig10_pilo      65898 TCCTCATGGTAATAGGGTGTCACG---GTGTTCG-AATACTGAATTTCTC 65943
                            v i ii    vii  i     i ---  v  v -i v     ii i i 
C rnd-4_family-       4104 TGCCCGCGGTATCGGGATGTCATGGGAGTTTTAGCGAGACTGAGCTCCCC 4055

  contig10_pilo      65944 AC 65945
                             
C rnd-4_family-       4054 AC 4053

Matrix = 20p53g.matrix
Kimura (with divCpGMod) = 28.96
Transitions / transversions = 1.86 (26/14)
Gap_init rate = 0.03 (4 / 146), avg. gap size = 1.50 (6 / 4)

 
 

 
 
   +    2476   13.4  2.9  2.9  contig10_pilon    65817   66299 (1786380) C  rnd-4_family-3933  Unknown           (84)    483       1    22     
 
 ANNOTATION EVIDENCE: 
  2476  13.43 2.90 2.90  contig10_pilon    65817   66299   1786380 C  rnd-4_family-3933  Unknown              1    483      84      
2476 13.43 2.90 2.90 contig10_pilon 65817 66299 (1786380) C rnd-4_family-3933#Unknown (84) 483 1 m_b685s001i1

  contig10_pilo      65817 AAGCTAATAATTATTACCGCGTCACGCGTCACGGAGGCCTCATGGCCCGG 65866
                                                                  v  i       
C rnd-4_family-        483 AAGCTAATAATTATTACCGCGTCACGCGTCACGGAGGCCGCACGGCCCGG 434

  contig10_pilo      65867 TAATAAACACGTTTTGTAGCATGTCACGGGCTCCTCATGGTAATAGGGTG 65916
                                   i           i          iv    i         i  
C rnd-4_family-        433 TAATAAACGCGTTTTGTAGCGTGTCACGGGCCGCTCACGGTAATAGGATG 384

  contig10_pilo      65917 TCACGGTGTTCGAATACTGAATTTCTCACCGGTAACCGTCCGGAGCAGCG 65966
                                         vi iv          iv  i      v     i   
C rnd-4_family-        383 TCACGGTGTTCGAAGGCCCAATTTCTCACTTGTGACCGTCGGGAGCGGCG 334

  contig10_pilo      65967 CGCCTTCGGCGCGCAGATTGTACAAGTTACATAACAGCAAAATTTCACA- 66015
                                          ?ivi    i -           v?    i     -
C rnd-4_family-        333 CGCCTTCGGCGCGCANGACGTACGA-TTACATAACAGGNAAATCTCACAC 285

  contig10_pilo      66016 -GAATCTCTGCAGAAACACAGTGTTTGGCATCCCTATGGTCGATGTATTG 66064
                           - v            v       v  i   i ?iv  v?      ?  i 
C rnd-4_family-        284 GGTATCTCTGCAGAATCACAGTGGTTAGCACCNTGATTNTCGATGNATCG 235

  contig10_pilo      66065 CACAGATAAAGTGTTTGAGGGCCTGAGGAGGCCCCAGAAACAGCCTAGAA 66114
                            v i       i   i i  i             ------------i   
C rnd-4_family-        234 CCCGGATAAAGCGTTCGGGGACCTGAGGAGGCCC------------GGAA 197

  contig10_pilo      66115 ATGAGATAA-CTGTATCAAATACACTTTCTCACTGTCCAGTTGTGCC-TT 66162
                             v      - i vv    ii i-                       -i 
C rnd-4_family-        196 ATCAGATAATCCGGCTCAAGCAT-CTTTCTCACTGTCCAGTTGTGCCACT 148

  contig10_pilo      66163 GGT------CTGCTGAGAAGCGCTGATTGAGCCAAAATTTATCTTGTATG 66206
                            v ------   v        i i    v           v i  v vv 
C rnd-4_family-        147 GCTGTCCTCCTGGTGAGAAGCACCGATTCAGCCAAAATTTTTTTTCTCAG 98

  contig10_pilo      66207 TC---TGGAAATG-TCACGTGTCACGGGTGCCCCAAGGTAATGTCTCCGA 66252
                             ---   v    -              ?                     
C rnd-4_family-         97 TCGTATGGCAATGGTCACGTGTCACGGGNGCCCCAAGGTAATGTCTCCGA 48

  contig10_pilo      66253 CATGTCACGGTCCAACGTCCATGGTAATAATTATTAGCTCTACAGTA 66299
                            i            i? ?  vi                 i       
C rnd-4_family-         47 CGTGTCACGGTCCAGNGNCCCCGGTAATAATTATTAGCTTTACAGTA 1

Matrix = 20p53g.matrix
Kimura (with divCpGMod) = 11.15
Transitions / transversions = 1.42 (37/26)
Gap_init rate = 0.04 (21 / 482), avg. gap size = 1.33 (28 / 21)

 
 
 
 
 
  +      15   15.8  0.0  2.8  contig10_pilon    73432   73468 (1779211) +  (TCATAAT)n         Simple_repeat        1     36     (0)    23     
 
 ANNOTATION EVIDENCE: 
    15  15.81 0.00 2.78  contig10_pilon    73432   73468   1779211 +  (TCATAAT)n         Simple_repeat        1     36       0      
15 15.81 0.00 2.78 contig10_pilon 73432 73468 (1779211) (TCATAAT)n#Simple_repeat 1 36 (0) m_b685s252i0

  contig10_pilo      73432 TCATAATTCATGACCTCAAAACTCATAATTCATATTT 73468
                                      - vi   v  i            v  
  (TCATAAT)n#Si          1 TCATAATTCAT-AATTCATAATTCATAATTCATAATT 36

Matrix = Unknown
Transitions / transversions = 0.67 (2/3)
Gap_init rate = 0.03 (1 / 36), avg. gap size = 1.00 (1 / 1)

 
 

 
 
   +      17    8.8  0.0  0.0  contig10_pilon    88937   88960 (1763719) +  (C)n               Simple_repeat        1     24     (0)    24     
 
 ANNOTATION EVIDENCE: 
    17   8.85 0.00 0.00  contig10_pilon    88937   88960   1763719 +  (C)n               Simple_repeat        1     24       0      
17 8.85 0.00 0.00 contig10_pilon 88937 88960 (1763719) (C)n#Simple_repeat 1 24 (0) m_b685s252i1

  contig10_pilo      88937 CCCCCCCGCCCCCCCTCCCCCCCC 88960
                                  v       i        
  (C)n#Simple_r          1 CCCCCCCCCCCCCCCCCCCCCCCC 24

Matrix = Unknown
Transitions / transversions = 1.00 (1/1)
Gap_init rate = 0.00 (0 / 23), avg. gap size = 0.0 (0 / 0)

 
 
 
 
 
  +      12   15.0  8.1  0.0  contig10_pilon    89446   89482 (1763197) +  (CACGGTC)n         Simple_repeat        1     40     (0)    25     
 
 ANNOTATION EVIDENCE: 
    12  15.04 8.11 0.00  contig10_pilon    89446   89482   1763197 +  (CACGGTC)n         Simple_repeat        1     40       0      
12 15.04 8.11 0.00 contig10_pilon 89446 89482 (1763197) (CACGGTC)n#Simple_repeat 1 40 (0) m_b685s252i2

  contig10_pilo      89446 CACGGT-CACGGCTCAAGATCGAC-GTCCACGGTCC-CGG 89482
                                 -     ii  v i  v  -           -   
  (CACGGTC)n#Si          1 CACGGTCCACGGTCCACGGTCCACGGTCCACGGTCCACGG 40

Matrix = Unknown
Transitions / transversions = 1.50 (3/2)
Gap_init rate = 0.08 (3 / 36), avg. gap size = 1.00 (3 / 3)

 
 

 
 
   +      16    9.1  0.0 10.3  contig10_pilon   100872  100914 (1751765) +  (CACACC)n          Simple_repeat        1     39     (0)    26     
 
 ANNOTATION EVIDENCE: 
    16   9.05 0.00 10.26  contig10_pilon   100872  100914   1751765 +  (CACACC)n          Simple_repeat        1     39       0      
16 9.05 0.00 10.26 contig10_pilon 100872 100914 (1751765) (CACACC)n#Simple_repeat 1 39 (0) m_b685s252i3

  contig10_pilo     100872 CACACCCACACCCACAGCCGCACACCGACCCCCCAGCACTCAC 100914
                                           -  -      v - v    -   i   
  (CACACC)n#Sim          1 CACACCCACACCCACA-CC-CACACCCA-CACCCA-CACCCAC 39

Matrix = Unknown
Transitions / transversions = 0.50 (1/2)
Gap_init rate = 0.10 (4 / 42), avg. gap size = 1.00 (4 / 4)

 
 
 
 
 
  +      13   19.5  0.0  2.8  contig10_pilon   104681  104717 (1747962) +  (CGGCGC)n          Simple_repeat        1     36     (0)    27     
 
 ANNOTATION EVIDENCE: 
    13  19.48 0.00 2.78  contig10_pilon   104681  104717   1747962 +  (CGGCGC)n          Simple_repeat        1     36       0      
13 19.48 0.00 2.78 contig10_pilon 104681 104717 (1747962) (CGGCGC)n#Simple_repeat 1 36 (0) m_b685s252i4

  contig10_pilo     104681 CGGCGGCGGCGCCGACGCAGGCAGCCGCCGCTAGCGC 104717
                                v        i   v   -    v   ii    
  (CGGCGC)n#Sim          1 CGGCGCCGGCGCCGGCGCCGGC-GCCGGCGCCGGCGC 36

Matrix = Unknown
Transitions / transversions = 1.00 (3/3)
Gap_init rate = 0.03 (1 / 36), avg. gap size = 1.00 (1 / 1)

 
 

 
 
   +      13   17.1  2.3  4.8  contig10_pilon   106754  106796 (1745883) +  (TCAACGG)n         Simple_repeat        1     42     (0)    28     
 
 ANNOTATION EVIDENCE: 
    13  17.09 2.33 4.76  contig10_pilon   106754  106796   1745883 +  (TCAACGG)n         Simple_repeat        1     42       0      
13 17.09 2.33 4.76 contig10_pilon 106754 106796 (1745883) (TCAACGG)n#Simple_repeat 1 42 (0) m_b685s252i5

  contig10_pilo     106754 TCAACGGTCAAAGCGCCAACGGTCAAAGCGCGCACGGTC-ACGG 106796
                                      v - i          v - ivv      -    
  (TCAACGG)n#Si          1 TCAACGGTCAACG-GTCAACGGTCAACG-GTCAACGGTCAACGG 42

Matrix = Unknown
Transitions / transversions = 0.50 (2/4)
Gap_init rate = 0.07 (3 / 42), avg. gap size = 1.00 (3 / 3)

 
 
 
 
 
  +     252   17.6  2.9  0.0  contig10_pilon   111958  112025 (1740654) +  rnd-4_family-1220  Simple_repeat      598    667     (0)    29     
 
 ANNOTATION EVIDENCE: 
   252  17.65 2.94 0.00  contig10_pilon   111958  112025   1740654 +  rnd-4_family-1220  Simple_repeat      598    667    2969      
252 17.65 2.94 0.00 contig10_pilon 111958 112025 (1740654) rnd-4_family-1220#Simple_repeat 598 667 (2969) m_b685s001i2

  contig10_pilo     111958 CTCTCCACGGCGAAGGTGACGAG--GATGAGGACACCGACGACGAAGAAG 112005
                              v v   i  ?v     v   --        viv?           i 
  rnd-4_family-        598 CTCGCGACGACGNCGGTGAAGAGACGATGAGGAAGANGACGACGAAGAGG 647

  contig10_pilo     112006 ACGATGAGGACGAAGATGAG 112025
                               i        v  i   
  rnd-4_family-        648 ACGACGAGGACGACGACGAG 667

Matrix = 20p53g.matrix
Kimura (with divCpGMod) = 17.31
Transitions / transversions = 0.71 (5/7)
Gap_init rate = 0.01 (1 / 67), avg. gap size = 2.00 (2 / 1)

 
 

 
 
   +      17   19.2  0.0  0.0  contig10_pilon   112142  112177 (1740502) +  (CGA)n             Simple_repeat        1     36     (0)    30     
 
 ANNOTATION EVIDENCE: 
    17  19.22 0.00 0.00  contig10_pilon   112142  112177   1740502 +  (CGA)n             Simple_repeat        1     36       0      
17 19.22 0.00 0.00 contig10_pilon 112142 112177 (1740502) (CGA)n#Simple_repeat 1 36 (0) m_b685s252i6

  contig10_pilo     112142 CGACGAAGGCGAGGACGATAATGACGACGACGACGA 112177
                                 v i   v     ii i              
  (CGA)n#Simple          1 CGACGACGACGACGACGACGACGACGACGACGACGA 36

Matrix = Unknown
Transitions / transversions = 2.00 (4/2)
Gap_init rate = 0.00 (0 / 35), avg. gap size = 0.0 (0 / 0)

 
 
 
 
 
  +      13   18.2  0.0  0.0  contig10_pilon   112217  112247 (1740432) +  (CGATGA)n          Simple_repeat        1     31     (0)    31     
 
 ANNOTATION EVIDENCE: 
    13  18.22 0.00 0.00  contig10_pilon   112217  112247   1740432 +  (CGATGA)n          Simple_repeat        1     31       0      
13 18.22 0.00 0.00 contig10_pilon 112217 112247 (1740432) (CGATGA)n#Simple_repeat 1 31 (0) m_b685s252i7

  contig10_pilo     112217 CGACGAGGATGACGATGAAGATGAGGAAGAC 112247
                              i  v           v     v  v   
  (CGATGA)n#Sim          1 CGATGACGATGACGATGACGATGACGATGAC 31

Matrix = Unknown
Transitions / transversions = 0.25 (1/4)
Gap_init rate = 0.00 (0 / 30), avg. gap size = 0.0 (0 / 0)

 
 

 
 
   +      15   12.2  0.0  0.0  contig10_pilon   125784  125810 (1726869) +  (ACC)n             Simple_repeat        1     27     (0)    32     
 
 ANNOTATION EVIDENCE: 
    15  12.16 0.00 0.00  contig10_pilon   125784  125810   1726869 +  (ACC)n             Simple_repeat        1     27       0      
15 12.16 0.00 0.00 contig10_pilon 125784 125810 (1726869) (ACC)n#Simple_repeat 1 27 (0) m_b686s252i0

  contig10_pilo     125784 ACCATCACCACCACTCCCACCACCACC 125810
                               i         iv           
  (ACC)n#Simple          1 ACCACCACCACCACCACCACCACCACC 27

Matrix = Unknown
Transitions / transversions = 2.00 (2/1)
Gap_init rate = 0.00 (0 / 26), avg. gap size = 0.0 (0 / 0)

 
 
 
 
 
  +      12   15.3  3.5  0.0  contig10_pilon   126262  126290 (1726389) +  (GCGCC)n           Simple_repeat        1     30     (0)    33     
 
 ANNOTATION EVIDENCE: 
    12  15.26 3.45 0.00  contig10_pilon   126262  126290   1726389 +  (GCGCC)n           Simple_repeat        1     30       0      
12 15.26 3.45 0.00 contig10_pilon 126262 126290 (1726389) (GCGCC)n#Simple_repeat 1 30 (0) m_b686s252i1

  contig10_pilo     126262 GCGCCGCGCGGCGCTG-GCGTCGCCGCGCC 126290
                                    v    i -  vv         
  (GCGCC)n#Simp          1 GCGCCGCGCCGCGCCGCGCCGCGCCGCGCC 30

Matrix = Unknown
Transitions / transversions = 0.33 (1/3)
Gap_init rate = 0.04 (1 / 28), avg. gap size = 1.00 (1 / 1)

 
 

 
 
   +      12   20.0  0.0  2.9  contig10_pilon   136854  136889 (1715790) +  (CGCCGA)n          Simple_repeat        1     35     (0)    34     
 
 ANNOTATION EVIDENCE: 
    12  20.03 0.00 2.86  contig10_pilon   136854  136889   1715790 +  (CGCCGA)n          Simple_repeat        1     35       0      
12 20.03 0.00 2.86 contig10_pilon 136854 136889 (1715790) (CGCCGA)n#Simple_repeat 1 35 (0) m_b686s252i2

  contig10_pilo     136854 CGCCGTCACACGACGCCGACGCCGGCGCGCACGACG 136889
                                v i -              i   vv   v  
  (CGCCGA)n#Sim          1 CGCCGACGC-CGACGCCGACGCCGACGCCGACGCCG 35

Matrix = Unknown
Transitions / transversions = 0.50 (2/4)
Gap_init rate = 0.03 (1 / 35), avg. gap size = 1.00 (1 / 1)

 
 
 
 
 
  +      12    7.2 10.0  0.0  contig10_pilon   138587  138616 (1714063) +  (CGACGG)n          Simple_repeat        1     33     (0)    35     
 
 ANNOTATION EVIDENCE: 
    12   7.16 10.00 0.00  contig10_pilon   138587  138616   1714063 +  (CGACGG)n          Simple_repeat        1     33       0      
12 7.16 10.00 0.00 contig10_pilon 138587 138616 (1714063) (CGACGG)n#Simple_repeat 1 33 (0) m_b686s252i3

  contig10_pilo     138587 CGACGACGATGGCGA--G-GACGGCGACGGCGA 138616
                                i   i     -- -              
  (CGACGG)n#Sim          1 CGACGGCGACGGCGACGGCGACGGCGACGGCGA 33

Matrix = Unknown
Transitions / transversions = 1.00 (2/0)
Gap_init rate = 0.07 (2 / 29), avg. gap size = 1.50 (3 / 2)

 
 

 
 
   +     339    2.4  0.0  0.0  contig10_pilon   147418  147459 (1705220) +  rnd-3_family-237   Unknown              1     42   (293)    36     
 
 ANNOTATION EVIDENCE: 
   339   2.38 0.00 0.00  contig10_pilon   147418  147459   1705220 +  rnd-3_family-237   Unknown              1     42     293      
339 2.38 0.00 0.00 contig10_pilon 147418 147459 (1705220) rnd-3_family-237#Unknown 1 42 (293) m_b686s001i0

  contig10_pilo     147418 TCTCCTTCGCAGATTTTGACGTTCGAAGATAATCGTTCGAAG 147459
                                        i                            
  rnd-3_family-          1 TCTCCTTCGCAGACTTTGACGTTCGAAGATAATCGTTCGAAG 42

Matrix = 20p53g.matrix
Kimura (with divCpGMod) = 2.44
Transitions / transversions = 1.00 (1/0)
Gap_init rate = 0.00 (0 / 41), avg. gap size = 0.0 (0 / 0)

 
 
 
 
 
  +      13   15.7  0.0  7.9  contig10_pilon   154579  154619 (1698060) +  (GCAGG)n           Simple_repeat        1     38     (0)    37     
 
 ANNOTATION EVIDENCE: 
    13  15.74 0.00 7.89  contig10_pilon   154579  154619   1698060 +  (GCAGG)n           Simple_repeat        1     38       0      
13 15.74 0.00 7.89 contig10_pilon 154579 154619 (1698060) (GCAGG)n#Simple_repeat 1 38 (0) m_b686s252i4

  contig10_pilo     154579 GCAGGTCGATGGGCACGGCGAGGGCAGGGCTGCGCGGGGCA 154619
                                v - -     v   -          v v  i     
  (GCAGG)n#Simp          1 GCAGGGC-A-GGGCAGGGC-AGGGCAGGGCAGGGCAGGGCA 38

Matrix = Unknown
Transitions / transversions = 0.25 (1/4)
Gap_init rate = 0.07 (3 / 40), avg. gap size = 1.00 (3 / 3)

 
 

 
 
   +      13    9.6  2.6  8.1  contig10_pilon   156941  156979 (1695700) +  (CCGACG)n          Simple_repeat        1     37     (0)    38     
 
 ANNOTATION EVIDENCE: 
    13   9.61 2.56 8.11  contig10_pilon   156941  156979   1695700 +  (CCGACG)n          Simple_repeat        1     37       0      
13 9.61 2.56 8.11 contig10_pilon 156941 156979 (1695700) (CCGACG)n#Simple_repeat 1 37 (0) m_b686s252i5

  contig10_pilo     156941 CCGAGGACCGA-GCCGACGACCCACGCCAACGCCGAGCGC 156979
                               v -    -       -  v     i       -   
  (CCGACG)n#Sim          1 CCGACG-CCGACGCCGACG-CCGACGCCGACGCCGA-CGC 37

Matrix = Unknown
Transitions / transversions = 0.50 (1/2)
Gap_init rate = 0.11 (4 / 38), avg. gap size = 1.00 (4 / 4)

 
 
 
 
 
  +    2426    3.8  0.0  0.0  contig10_pilon   157439  157757 (1694922) C  rnd-4_family-4503  Unknown            (0)    319       1    39     
 
 ANNOTATION EVIDENCE: 
  2426   3.76 0.00 0.00  contig10_pilon   157439  157757   1694922 C  rnd-4_family-4503  Unknown              1    319       0      
2426 3.76 0.00 0.00 contig10_pilon 157439 157757 (1694922) C rnd-4_family-4503#Unknown (0) 319 1 m_b686s001i1

  contig10_pilo     157439 ACAGGGAGCAGGGTAGAGAATCCATAGAGTATCTATTGAGTATCCCGACT 157488
                             v        i             ?                        
C rnd-4_family-        319 ACTGGGAGCAGAGTAGAGAATCCATNGAGTATCTATTGAGTATCCCGACT 270

  contig10_pilo     157489 TGACATTCAGATGAATGGCACGAGTTTCCCAAGAGTATCATATCCTGAAA 157538
                                           v                                v
C rnd-4_family-        269 TGACATTCAGATGAATTGCACGAGTTTCCCAAGAGTATCATATCCTGAAC 220

  contig10_pilo     157539 TACTCTGGAGATACTCTATGCACAAGGTAGAATATTGAATGAGTATCTCA 157588
                                                        iv                   
C rnd-4_family-        219 TACTCTGGAGATACTCTATGCACAAGGTAATATATTGAATGAGTATCTCA 170

  contig10_pilo     157589 TGAGAACATTTGTGCCTCAAGGGATACTCATCCAATATATTACCTTGTGT 157638
                                       ?                                     
C rnd-4_family-        169 TGAGAACATTTGNGCCTCAAGGGATACTCATCCAATATATTACCTTGTGT 120

  contig10_pilo     157639 ATAGAGTATCTCCAGAGTAGTTCAGGATATGATACTCTTAGGAAACTCGT 157688
                                             i           ?        i          
C rnd-4_family-        119 ATAGAGTATCTCCAGAGTGGTTCAGGATATNATACTCTTGGGAAACTCGT 70

  contig10_pilo     157689 GCCATTCATCTGAATGTCAAGTCGGGATACTCAATAGATACTCTAAGGAT 157738
                                                v           i?        v i    
C rnd-4_family-         69 GCCATTCATCTGAATGTCAAGGCGGGATACTCAGNAGATACTCAAGGGAT 20

  contig10_pilo     157739 CCTCGGCCTTGCTCCCAGT 157757
                                              
C rnd-4_family-         19 CCTCGGCCTTGCTCCCAGT 1

Matrix = 20p53g.matrix
Kimura (with divCpGMod) = 3.91
Transitions / transversions = 1.00 (6/6)
Gap_init rate = 0.00 (0 / 318), avg. gap size = 0.0 (0 / 0)

 
 

 
 
   +     356   24.1  2.7  2.7  contig10_pilon   173908  174056 (1678623) +  rnd-4_family-134   Unknown            472    620  (9252)    40     
 
 ANNOTATION EVIDENCE: 
   356  24.14 2.68 2.68  contig10_pilon   173908  174056   1678623 +  rnd-4_family-134   Unknown            472    620    9252      
356 24.14 2.68 2.68 contig10_pilon 173908 174056 (1678623) rnd-4_family-134#Unknown 472 620 (9252) m_b686s001i2

  contig10_pilo     173908 CAGGACAGATGGTACATCTTCGATCCCGCAGCGCTGCACGACCTCGCGAG 173957
                              v  v v v ? v        i    vi v        v   v   v 
  rnd-4_family-        472 CAGCACCGCTCGNAAATCTTCGACCCCGAGGAGCTGCACGCCCTGGCGCG 521

  contig10_pilo     173958 ---GACGTCCGTGGACCTGCACGCGAACGACACGCGCGCGATGGT-GAAC 174003
                           --- i   iv i  ---i    v                vi v  -  vv
  rnd-4_family-        522 CTCGGCGTTGGCGG---CGCACCCGAACGACACGCGCGCCGTCGTCGATA 568

  contig10_pilo     174004 CACATCGTCTCCACGCTCGCGCACACGCACGGCGCGCAGCACATCAATGT 174053
                             -      vv           iv ii       v    vvvi?   i  
  rnd-4_family-        569 CA-ATCGTCAGCACGCTCGCGCGGATACACGGCGAGCAGACGGNCAACGT 617

  contig10_pilo     174054 GCG 174056
                              
  rnd-4_family-        618 GCG 620

Matrix = 20p53g.matrix
Kimura (with divCpGMod) = 25.15
Transitions / transversions = 0.52 (12/23)
Gap_init rate = 0.04 (6 / 148), avg. gap size = 1.33 (8 / 6)

 
 
 
  +     651   20.4  1.5  1.5  contig10_pilon   174180  174378 (1678301) +  rnd-4_family-134   Unknown            671    869  (9003)    40     
 
 ANNOTATION EVIDENCE: 
   651  20.41 1.51 1.51  contig10_pilon   174180  174378   1678301 +  rnd-4_family-134   Unknown            671    869    9003      
651 20.41 1.51 1.51 contig10_pilon 174180 174378 (1678301) rnd-4_family-134#Unknown 671 869 (9003) m_b686s001i3

  contig10_pilo     174180 TCAGCAACGCGGGCGGCGCGATGGGCGCGGTGTACGTGATCCACGCGTCG 174229
                              ii                       vi     vvv    v   -iv 
  rnd-4_family-        671 TCAATAACGCGGGCGGCGCGATGGGCGCCATGTACCACATCCCCGC-CAG 719

  contig10_pilo     174230 C-TGACTGAGTACCTCATGATCTTCGGCACGCCGCTGGGCACCGAGGGCC 174278
                            - v  i   v       v        i  v     v?    i     i 
  rnd-4_family-        720 CATCACCGAGGACCTCATCATCTTCGGTACCCCGCTCNGCACTGAGGGTC 769

  contig10_pilo     174279 ACACCGGGCGCTACCTCGCCGAGGACTACTT-CCACATCCTCCAGGGCGA 174327
                            iv  - v   i ivi     vv        - v        v       
  rnd-4_family-        770 ATTCC-GTCGCCATACCGCCGTCGACTACTTACAACATCCTCGAGGGCGA 818

  contig10_pilo     174328 GCAGTGGGCGTTCTCCGCC-GGCAGCCTCGAAAAGGAGGTGCGTTCTCCG 174376
                            v  i    v  - v    -   iv      v ?      v   i     
  rnd-4_family-        819 GGAGCGGGCCTT-TGCGCCAGGCGCCCTCGACANGGAGGTCCGTCCTCCG 867

  contig10_pilo     174377 TT 174378
                           i 
  rnd-4_family-        868 CT 869

Matrix = 20p53g.matrix
Kimura (with divCpGMod) = 22.22
Transitions / transversions = 0.67 (16/24)
Gap_init rate = 0.03 (6 / 198), avg. gap size = 1.00 (6 / 6)

 
 

   +     633   17.4  0.0  0.0  contig10_pilon   174526  174657 (1678022) +  rnd-4_family-134   Unknown            912   1043  (8829)    40     
 
 ANNOTATION EVIDENCE: 
   633  17.42 0.00 0.00  contig10_pilon   174526  174657   1678022 +  rnd-4_family-134   Unknown            912   1043    8829      
633 17.42 0.00 0.00 contig10_pilon 174526 174657 (1678022) rnd-4_family-134#Unknown 912 1043 (8829) m_b687s001i2

  contig10_pilo     174526 AGGTCTACAAACCGGGAAGTGTGCACGTCCTCCCGCGCGGGGCGGTGCAG 174575
                                   vv v vv v  iv  i  vv            v iv  vv  
  rnd-4_family-        912 AGGTCTACCCAGCCCGCAGCCTGTACCACCTCCCGCGCGGCGTCGTCAAG 961

  contig10_pilo     174576 CAGTACAAGATGCACGAGGGCTGCTGGGCCTTCGAGTATGCGCGCGGCTG 174625
                                                        vi       i           
  rnd-4_family-        962 CAGTACAAGATGCACGAGGGCTGCTGGGCGCTCGAGTACGCGCGCGGCTG 1011

  contig10_pilo     174626 GATCCCACTCATGCTGCCCTTCGGACTCGCCG 174657
                                 i i               vi      
  rnd-4_family-       1012 GATCCCGCCCATGCTGCCCTTCGGCTTCGCCG 1043

Matrix = 20p53g.matrix
Kimura (with divCpGMod) = 18.08
Transitions / transversions = 0.53 (8/15)
Gap_init rate = 0.00 (0 / 131), avg. gap size = 0.0 (0 / 0)

 
 
 
  +     323   24.1  0.0  0.0  contig10_pilon   174745  174823 (1677856) +  rnd-4_family-134   Unknown           1057   1135  (8737)    40     
 
 ANNOTATION EVIDENCE: 
   323  24.05 0.00 0.00  contig10_pilon   174745  174823   1677856 +  rnd-4_family-134   Unknown           1057   1135    8737      
323 24.05 0.00 0.00 contig10_pilon 174745 174823 (1677856) rnd-4_family-134#Unknown 1057 1135 (8737) m_b687s001i3

  contig10_pilo     174745 CACGCTGGACTTCATCACGATATACAACACCGCCCGCGTCTCCGCCCGCG 174794
                                 v         v viiv   v      i    i  v   v     
  rnd-4_family-       1057 CACGCTCGACTTCATCTCCGCCTACCACACCGTCCGCATCACCGGCCGCG 1106

  contig10_pilo     174795 AGATGCTTCGTAATCTGCTGGTTGGCAAG 174823
                                v i  i  i     vivi      
  rnd-4_family-       1107 AGATGATCCGCAACCTGCTCAACGGCAAG 1135

Matrix = 20p53g.matrix
Kimura (with divCpGMod) = 25.77
Transitions / transversions = 0.90 (9/10)
Gap_init rate = 0.00 (0 / 78), avg. gap size = 0.0 (0 / 0)

 
 

 
 
   +      26   16.6  2.7  4.2  contig10_pilon   186112  186184 (1666495) +  (CGTGTG)n          Simple_repeat        1     72     (0)    41     
 
 ANNOTATION EVIDENCE: 
    26  16.55 2.74 4.17  contig10_pilon   186112  186184   1666495 +  (CGTGTG)n          Simple_repeat        1     72       0      
26 16.55 2.74 4.17 contig10_pilon 186112 186184 (1666495) (CGTGTG)n#Simple_repeat 1 72 (0) m_b687s252i0

  contig10_pilo     186112 CGTCGTGCGTGTGCGACTGCGAGTGCGTCTCCGCGTGCATGCTGTCGTCT 186161
                              -           vv    v      v v  i    i  -  -   v 
  (CGTGTG)n#Sim          1 CGT-GTGCGTGTGCGTGTGCGTGTGCGTGTGCGTGTGCGTG-TG-CGTGT 47

  contig10_pilo     186162 --GTGTGCGCGTGCGTGTGCGCGTG 186184
                           --       i           i   
  (CGTGTG)n#Sim         48 GCGTGTGCGTGTGCGTGTGCGTGTG 72

Matrix = Unknown
Transitions / transversions = 0.67 (4/6)
Gap_init rate = 0.06 (4 / 72), avg. gap size = 1.25 (5 / 4)

 
 
 
 
 
  +      27   29.2  1.0  1.0  contig10_pilon   187453  187549 (1665130) +  (GTCGTC)n          Simple_repeat        1     97     (0)    42     
 
 ANNOTATION EVIDENCE: 
    27  29.17 1.03 1.03  contig10_pilon   187453  187549   1665130 +  (GTCGTC)n          Simple_repeat        1     97       0      
27 29.17 1.03 1.03 contig10_pilon 187453 187549 (1665130) (GTCGTC)n#Simple_repeat 1 97 (0) m_b687s252i1

  contig10_pilo     187453 GTCGTCCTGGTGGTGTTCGTGTTCGTCGGTGTCCATGTAGTCAGT-GTCG 187501
                                 v v  v  vv    vv      vi   vvi  v   -  -    
  (GTCGTC)n#Sim          1 GTCGTCGTCGTCGTCGTCGTCGTCGTCGTCGTCGTCGTCGTC-GTCGTCG 49

  contig10_pilo     187502 TCTTCGTTATCGTCTCCGTAGTCGTCGTAATCGTCGTTGTCGTCCTCG 187549
                             v    ii     vi   v        vi       i      v   
  (GTCGTC)n#Sim         50 TCGTCGTCGTCGTCGTCGTCGTCGTCGTCGTCGTCGTCGTCGTCGTCG 97

Matrix = Unknown
Transitions / transversions = 0.44 (7/16)
Gap_init rate = 0.02 (2 / 96), avg. gap size = 1.00 (2 / 2)

 
 

 
 
   +      12   22.2  6.4  0.0  contig10_pilon   188029  188075 (1664604) +  (CTCGGCG)n         Simple_repeat        1     50     (0)    43     
 
 ANNOTATION EVIDENCE: 
    12  22.17 6.38 0.00  contig10_pilon   188029  188075   1664604 +  (CTCGGCG)n         Simple_repeat        1     50       0      
12 22.17 6.38 0.00 contig10_pilon 188029 188075 (1664604) (CTCGGCG)n#Simple_repeat 1 50 (0) m_b687s252i2

  contig10_pilo     188029 CTCGTAGCTCTTTGCGCGGCGCTCGGCGCT-GAC-C-CGGCGCTGGTCGC 188075
                               vv    vvi  v              - i - -       v v   
  (CTCGGCG)n#Si          1 CTCGGCGCTCGGCGCTCGGCGCTCGGCGCTCGGCGCTCGGCGCTCGGCGC 50

Matrix = Unknown
Transitions / transversions = 0.29 (2/7)
Gap_init rate = 0.07 (3 / 46), avg. gap size = 1.00 (3 / 3)

 
 
 
 
 
  +      19   23.4  0.0  0.0  contig10_pilon   188284  188333 (1664346) +  (CGCCGG)n          Simple_repeat        1     50     (0)    44     
 
 ANNOTATION EVIDENCE: 
    19  23.41 0.00 0.00  contig10_pilon   188284  188333   1664346 +  (CGCCGG)n          Simple_repeat        1     50       0      
19 23.41 0.00 0.00 contig10_pilon 188284 188333 (1664346) (CGCCGG)n#Simple_repeat 1 50 (0) m_b687s252i3

  contig10_pilo     188284 CGTCGGCGGCGGCGCTGGACCCGGCGCTGACGCGGGCGTCGGCGACGGCG 188333
                             i     v      i  vv       i i   v    i     v     
  (CGCCGG)n#Sim          1 CGCCGGCGCCGGCGCCGGCGCCGGCGCCGGCGCCGGCGCCGGCGCCGGCG 50

Matrix = Unknown
Transitions / transversions = 1.00 (5/5)
Gap_init rate = 0.00 (0 / 49), avg. gap size = 0.0 (0 / 0)

 
 

 
 
   +      13   15.0  3.3  0.0  contig10_pilon   199777  199806 (1652873) +  (TCTTT)n           Simple_repeat        1     31     (0)    45     
 
 ANNOTATION EVIDENCE: 
    13  15.01 3.33 0.00  contig10_pilon   199777  199806   1652873 +  (TCTTT)n           Simple_repeat        1     31       0      
13 15.01 3.33 0.00 contig10_pilon 199777 199806 (1652873) (TCTTT)n#Simple_repeat 1 31 (0) m_b687s252i5

  contig10_pilo     199777 TCCTTTCTTTTCTTTTC-CTTCTTTGCCTTT 199806
                             i              -i      v i   
  (TCTTT)n#Simp          1 TCTTTTCTTTTCTTTTCTTTTCTTTTCTTTT 31

Matrix = Unknown
Transitions / transversions = 3.00 (3/1)
Gap_init rate = 0.03 (1 / 29), avg. gap size = 1.00 (1 / 1)

 
 
 
 
 
  +      12    5.5  0.0  0.0  contig10_pilon   200594  200612 (1652067) +  (GTC)n             Simple_repeat        1     19     (0)    46     
 
 ANNOTATION EVIDENCE: 
    12   5.48 0.00 0.00  contig10_pilon   200594  200612   1652067 +  (GTC)n             Simple_repeat        1     19       0      
12 5.48 0.00 0.00 contig10_pilon 200594 200612 (1652067) (GTC)n#Simple_repeat 1 19 (0) m_b687s252i6

  contig10_pilo     200594 GTCGTCGTCCTCGTCGTCG 200612
                                    v         
  (GTC)n#Simple          1 GTCGTCGTCGTCGTCGTCG 19

Matrix = Unknown
Transitions / transversions = 0.00 (0/1)
Gap_init rate = 0.00 (0 / 18), avg. gap size = 0.0 (0 / 0)

 
 

 
 
   +      11   24.1  0.0  0.0  contig10_pilon   204257  204290 (1648389) +  (CCCCTG)n          Simple_repeat        1     34     (0)    47     
 
 ANNOTATION EVIDENCE: 
    11  24.12 0.00 0.00  contig10_pilon   204257  204290   1648389 +  (CCCCTG)n          Simple_repeat        1     34       0      
11 24.12 0.00 0.00 contig10_pilon 204257 204290 (1648389) (CCCCTG)n#Simple_repeat 1 34 (0) m_b687s252i7

  contig10_pilo     204257 CCCCGGCCCCTTGCTCTGCCCTTGCCGCTGCTCC 204290
                               v      vv i      i    v    i  
  (CCCCTG)n#Sim          1 CCCCTGCCCCTGCCCCTGCCCCTGCCCCTGCCCC 34

Matrix = Unknown
Transitions / transversions = 0.75 (3/4)
Gap_init rate = 0.00 (0 / 33), avg. gap size = 0.0 (0 / 0)

 
 
 
 
 
  +     282   25.4  6.2  9.3  contig10_pilon   205953  206195 (1646484) C  rnd-4_family-1155  LTR/Gypsy          (0)  11186   10951    48     
 
 ANNOTATION EVIDENCE: 
   282  25.42 6.17 9.32  contig10_pilon   205953  206195   1646484 C  rnd-4_family-1155  LTR/Gypsy        10951  11186       0      
282 25.42 6.17 9.32 contig10_pilon 205953 206195 (1646484) C rnd-4_family-1155#LTR/Gypsy (0) 11186 10951 m_b687s001i4

  contig10_pilo     205953 GTAAGGCCAAGCCGCCATGGGCGGCGTGTGAATCTATCTAGTCCAAAATA 206002
                                       v      i i ivvv     -------    v v  vi
C rnd-4_family-      11186 GTAAGGCCAAGCGGCCATGAGTGAATAGTGAA-------AGTCAATAAAG 11144

  contig10_pilo     206003 TAATTCAGACGCACCTATGGC-TTGGGCCTTTAGACA--CTTGAGGAGCT 206049
                            v   ---  v    v     -               --     -     
C rnd-4_family-      11143 TCATT---ACTCACCGATGGCTTTGGGCCTTTAGACAATCTTGA-GAGCT 11098

  contig10_pilo     206050 TCCAAGG-GTGAGATCACACAGTAAACTGAATGGGATATCACTCCGTGTT 206098
                           v ivi  -v vvvv    v   vi --         vv v?   --    
C rnd-4_family-      11097 GCTTGGGCCTTTTCTCACTCAGGGA--TGAATGGGAATTGNCTC--TGTT 11052

  contig10_pilo     206099 AGGAAGACTGAAGA----------TTATAGTACATGATTCTATCCGTATT 206138
                            i v       i  ----------i  v i    -  ---   iii  i 
C rnd-4_family-      11051 AAGCAGACTGAGGAGCCTAGAGACCTAAAATACA-GA---TATTTATACT 11006

  contig10_pilo     206139 TAAACTACCAATTGAGTCATAATACTCAGGTCTGTGAGTCG-GTTCCTAA 206187
                           v   i   vii v       ii-?i ?  --  v   v   - i    v 
C rnd-4_family-      11005 AAAATTACAGGTAGAGTCATGG-NTTNAG--CTCTGACTCGAGCTCCTCA 10959

  contig10_pilo     206188 GTCATTTC 206195
                            ii  ii 
C rnd-4_family-      10958 GCTATCCC 10951

Matrix = 20p53g.matrix
Kimura (with divCpGMod) = 31.64
Transitions / transversions = 0.87 (26/30)
Gap_init rate = 0.11 (27 / 242), avg. gap size = 1.37 (37 / 27)

 
 

 
 
   +      12   26.4  2.1  2.1  contig10_pilon   208469  208516 (1644163) +  (GCTGCA)n          Simple_repeat        1     48     (0)    49     
 
 ANNOTATION EVIDENCE: 
    12  26.37 2.08 2.08  contig10_pilon   208469  208516   1644163 +  (GCTGCA)n          Simple_repeat        1     48       0      
12 26.37 2.08 2.08 contig10_pilon 208469 208516 (1644163) (GCTGCA)n#Simple_repeat 1 48 (0) m_b687s252i8

  contig10_pilo     208469 GCTGGAGCTTGAGGAGCACCTGCCGACGGCA-CTGCAGGAGCAGCTGCA 208516
                               v    vv  vv   v    v - v   -      vv         
  (GCTGCA)n#Sim          1 GCTGCAGCTGCAGCTGCAGCTGCAG-CTGCAGCTGCAGCTGCAGCTGCA 48

Matrix = Unknown
Transitions / transversions = 0.00 (0/10)
Gap_init rate = 0.04 (2 / 47), avg. gap size = 1.00 (2 / 2)

 
 
 
 
 
  +      14   14.8  0.0  3.2  contig10_pilon   211874  211905 (1640774) +  (GCC)n             Simple_repeat        1     31     (0)    50     
 
 ANNOTATION EVIDENCE: 
    14  14.85 0.00 3.23  contig10_pilon   211874  211905   1640774 +  (GCC)n             Simple_repeat        1     31       0      
14 14.85 0.00 3.23 contig10_pilon 211874 211905 (1640774) (GCC)n#Simple_repeat 1 31 (0) m_b687s252i9

  contig10_pilo     211874 GCCGCCGCCGCCGCCGGAGACGCACGACGCCG 211905
                                           vv v   -  v     
  (GCC)n#Simple          1 GCCGCCGCCGCCGCCGCCGCCGC-CGCCGCCG 31

Matrix = Unknown
Transitions / transversions = 0.00 (0/4)
Gap_init rate = 0.03 (1 / 31), avg. gap size = 1.00 (1 / 1)

 
 

 
 
   +      18   13.3  5.7  3.7  contig10_pilon   212030  212082 (1640597) +  (CAGCGCG)n         Simple_repeat        1     54     (0)    51     
 
 ANNOTATION EVIDENCE: 
    18  13.29 5.66 3.70  contig10_pilon   212030  212082   1640597 +  (CAGCGCG)n         Simple_repeat        1     54       0      
18 13.29 5.66 3.70 contig10_pilon 212030 212082 (1640597) (CAGCGCG)n#Simple_repeat 1 54 (0) m_b687s252i10

  contig10_pilo     212030 CAGCGTGCAGGCGCGCAGCGTGC-GCTCGCTGCGC-CATGCGCGGAGCGA 212077
                                i   -          i  -  v   v    -  -     v    v
  (CAGCGCG)n#Si          1 CAGCGCGCA-GCGCGCAGCGCGCAGCGCGCAGCGCGCA-GCGCGCAGCGC 48

  contig10_pilo     212078 G-AGCG 212082
                            -    
  (CAGCGCG)n#Si         49 GCAGCG 54

Matrix = Unknown
Transitions / transversions = 0.50 (2/4)
Gap_init rate = 0.10 (5 / 52), avg. gap size = 1.00 (5 / 5)

 
 
 
 
 
  +      19   18.8  4.7  3.1  contig10_pilon   218642  218705 (1633974) +  (AGGAGTG)n         Simple_repeat        1     65     (0)    52     
 
 ANNOTATION EVIDENCE: 
    19  18.80 4.69 3.08  contig10_pilon   218642  218705   1633974 +  (AGGAGTG)n         Simple_repeat        1     65       0      
19 18.80 4.69 3.08 contig10_pilon 218642 218705 (1633974) (AGGAGTG)n#Simple_repeat 1 65 (0) m_b687s252i11

  contig10_pilo     218642 AGCAGTG-TGAGTTGAGTAGTGAGGAGTGAGCA-TGAGGAGTGA-AAGGG 218688
                             v    -v   -    v             v -          -i  v 
  (AGGAGTG)n#Si          1 AGGAGTGAGGAG-TGAGGAGTGAGGAGTGAGGAGTGAGGAGTGAGGAGTG 49

  contig10_pilo     218689 GGGAGAAGACGTGTGAG 218705
                           i    -v  v v     
  (AGGAGTG)n#Si         50 AGGAG-TGAGGAGTGAG 65

Matrix = Unknown
Transitions / transversions = 0.25 (2/8)
Gap_init rate = 0.08 (5 / 63), avg. gap size = 1.00 (5 / 5)

 
 

 
 
   +    3519    5.9  2.6  0.4  contig10_pilon   220746  221240 (1631439) C  rnd-4_family-3166  Unknown            (1)    506       1    53     
 
 ANNOTATION EVIDENCE: 
  3519   5.88 2.63 0.40  contig10_pilon   220746  221240   1631439 C  rnd-4_family-3166  Unknown              1    506       1      
3519 5.88 2.63 0.40 contig10_pilon 220746 221240 (1631439) C rnd-4_family-3166#Unknown (1) 506 1 m_b687s001i5

  contig10_pilo     220746 CAAGGGGCAGGCAAGGGGAAAATAGTGGACATGCACCCAAAAAAAAGTCA 220795
                                                          i                  
C rnd-4_family-        506 CAAGGGGCAGGCAAGGGGAAAATAGTGGACACGCACCCAAAAAAAAGTCA 457

  contig10_pilo     220796 TAAGCGGTTTGCCGAGCCCCCCCTCCCCCCC-TCGGAAGCAAATTTGACC 220844
                                                          -                  
C rnd-4_family-        456 TAAGCGGTTTGCCGAGCCCCCCCTCCCCCCCCTCGGAAGCAAATTTGACC 407

  contig10_pilo     220845 CCGCGGATGCCGTATTTGGAACTGAAACTTGGTCAACTCCCGTTAAATGC 220894
                                          ?   i  i                    ?      
C rnd-4_family-        406 CCGCGGATGCCGTATNTGGGACCGAAACTTGGTCAACTCCCGTNAAATGC 357

  contig10_pilo     220895 TCAAATGTCGATTTTCAAAGTGTCCAGCAGCTCAGTAGACGCAGCGCAAG 220944
                             i    ?   i             i     v  ii              
C rnd-4_family-        356 TCGAATGNCGACTTTCAAAGTGTCCGGCAGCGCAACAGACGCAGCGCAAG 307

  contig10_pilo     220945 TGTAGCCGCGCTCCTGGCGGAGGTAAATTCTGGAAAAGGAAACCGCTGAC 220994
                                        iv                                   
C rnd-4_family-        306 TGTAGCCGCGCTCTGGGCGGAGGTAAATTCTGGAAAAGGAAACCGCTGAC 257

  contig10_pilo     220995 ATAAGCCCTCGGACATCGCCGCTCCCAGCGTGTTCCTGCTCCTCAAATGG 221044
                                                     i     i            i    
C rnd-4_family-        256 ATAAGCCCTCGGACATCGCCGCTCCCGGCGTGCTCCTGCTCCTCAGATGG 207

  contig10_pilo     221045 CCAAATCACTGCATAGAGTC------------TTGCTAAAGTTCTTGGAG 221082
                                 ii            ------------i     i  i        
C rnd-4_family-        206 CCAAATTGCTGCATAGAGTCATGATTCCATTGCTGCTAGAGCTCTTGGAG 157

  contig10_pilo     221083 CAAGTAAAGAAAGGGATATGCTGTGGAAGACGCAGACAATGTTCAAATGG 221132
                              i    i?vi iv  i?       i          ?  ?         
C rnd-4_family-        156 CAAATAAAANCGGATATGNGCTGTGGGAGACGCAGACNATNTTCAAATGG 107

  contig10_pilo     221133 GCCTCCCGGACCCCCTTCGGAAGCAAATATAAAAAAAAGTTATAAGCGTT 221182
                                          i          --                      
C rnd-4_family-        106 GCCTCCCGGACCCCCCTCGGAAGCAA--ATAAAAAAAAGTTATAAGCGTT 59

  contig10_pilo     221183 TGCCTGGACCCACCCCCCACCCGGTCCGTGTACCGGAGTTTGCCGATGCC 221232
                                                                         i   
C rnd-4_family-         58 TGCCTGGACCCACCCCCCACCCGGTCCGTGTACCGGAGTTTGCCGACGCC 9

  contig10_pilo     221233 TGCCCCTT 221240
                                   
C rnd-4_family-          8 TGCCCCTT 1

Matrix = 20p53g.matrix
Kimura (with divCpGMod) = 4.87
Transitions / transversions = 6.25 (25/4)
Gap_init rate = 0.01 (4 / 494), avg. gap size = 3.75 (15 / 4)

 
 
 
 
 
  +      32    0.0  0.0  0.0  contig10_pilon   229318  229347 (1623332) +  (CCTT)n            Simple_repeat        1     30     (0)    54     
 
 ANNOTATION EVIDENCE: 
    32   0.00 0.00 0.00  contig10_pilon   229318  229347   1623332 +  (CCTT)n            Simple_repeat        1     30       0      
32 0.00 0.00 0.00 contig10_pilon 229318 229347 (1623332) (CCTT)n#Simple_repeat 1 30 (0) c_b687s251i0

  contig10_pilo     229318 CCTTCCTTCCTTCCTTCCTTCCTTCCTTCC 229347
                                                         
  (CCTT)n#Simpl          1 CCTTCCTTCCTTCCTTCCTTCCTTCCTTCC 30

Matrix = Unknown
Transitions / transversions = 1.00 (0/0)
Gap_init rate = 0.00 (0 / 29), avg. gap size = 0.0 (0 / 0)

 
 

 
 
   +      13   25.9  0.0  2.4  contig10_pilon   233555  233597 (1619082) +  (GTC)n             Simple_repeat        1     42     (0)    55     
 
 ANNOTATION EVIDENCE: 
    13  25.94 0.00 2.38  contig10_pilon   233555  233597   1619082 +  (GTC)n             Simple_repeat        1     42       0      
13 25.94 0.00 2.38 contig10_pilon 233555 233597 (1619082) (GTC)n#Simple_repeat 1 42 (0) m_b688s252i0

  contig10_pilo     233555 GTCCTCGTCGTCCACGCCGCCGGCGTTCGCCGTCGTCAGCGTC 233597
                              v        vv  i  i  v  -   i       iv    
  (GTC)n#Simple          1 GTCGTCGTCGTCGTCGTCGTCGTCG-TCGTCGTCGTCGTCGTC 42

Matrix = Unknown
Transitions / transversions = 0.80 (4/5)
Gap_init rate = 0.02 (1 / 42), avg. gap size = 1.00 (1 / 1)

 
 
 
 
 
  +      12   15.3  3.5  0.0  contig10_pilon   238216  238244 (1614435) +  (TCTC)n            Simple_repeat        1     30     (0)    56     
 
 ANNOTATION EVIDENCE: 
    12  15.30 3.45 0.00  contig10_pilon   238216  238244   1614435 +  (TCTC)n            Simple_repeat        1     30       0      
12 15.30 3.45 0.00 contig10_pilon 238216 238244 (1614435) (TCTC)n#Simple_repeat 1 30 (0) m_b688s252i1

  contig10_pilo     238216 TCTCCCTCTCACT-TCTCTCTGTCTCCCTC 238244
                               i     v  -       v    i   
  (TCTC)n#Simpl          1 TCTCTCTCTCTCTCTCTCTCTCTCTCTCTC 30

Matrix = Unknown
Transitions / transversions = 1.00 (2/2)
Gap_init rate = 0.04 (1 / 28), avg. gap size = 1.00 (1 / 1)

 
 

 
 
   +     849   21.9  1.4  0.5  contig10_pilon   250334  250544 (1602135) C  rnd-4_family-877   Unknown            (0)  10303   10091    57     
 
 ANNOTATION EVIDENCE: 
   849  21.90 1.42 0.47  contig10_pilon   250334  250544   1602135 C  rnd-4_family-877   Unknown          10091  10303       0      
849 21.90 1.42 0.47 contig10_pilon 250334 250544 (1602135) C rnd-4_family-877#Unknown (0) 10303 10091 m_b688s001i0

  contig10_pilo     250334 CAATGGTACCGGGGACTTTCGCTTAACCGATACCCGCACTTTGTGCAGCG 250383
                                                v        ???        i        
C rnd-4_family-      10303 CAATGGTACCGGGGACTTTCGGTTAACCGANNNCCGCACTTCGTGCAGCG 10254

  contig10_pilo     250384 AATCGGTTAAGCGAAACCCGCACTTTGGCACGTTTTTCAA---AGTAATT 250430
                           v         v    v               i    ?v i---     v 
C rnd-4_family-      10253 CATCGGTTAACCGAATCCCGCACTTTGGCACATTTTNGAGCCTAGTAAGT 10204

  contig10_pilo     250431 AAAATGTTATCGGGGTGAAAGTAGATGTACGATCCTGCTGTACAAATCAA 250480
                           -      v   i vii vv     vv iv    vi i i   i   i iv
C rnd-4_family-      10203 -AAATGTAATCAGCACGTCAGTAGCGGCTCGATATTACCGTATAAACCGC 10155

  contig10_pilo     250481 AGAGAACCGCTTGGAGGTCTCGTAAGCTCCATAATAATTCGTTGAGTTTC 250530
                           v              v iv    i  v?  iivi   v   i   viv  
C rnd-4_family-      10154 TGAGAACCGCTTGGACGCATCGTGAGANCCGCTGTAAATCGCTGACCATC 10105

  contig10_pilo     250531 GCTGGATTTCGCTG 250544
                            v  i i       
C rnd-4_family-      10104 GGTGAACTTCGCTG 10091

Matrix = 20p53g.matrix
Kimura (with divCpGMod) = 23.64
Transitions / transversions = 0.92 (22/24)
Gap_init rate = 0.01 (2 / 210), avg. gap size = 2.00 (4 / 2)

 
 
 
 
 
  +     538   22.6 13.5  8.2  contig10_pilon   250598  250886 (1601793) C  rnd-4_family-877   Unknown         (9806)    497     195    58     
 
 ANNOTATION EVIDENCE: 
   538  22.63 13.49 8.25  contig10_pilon   250598  250886   1601793 C  rnd-4_family-877   Unknown            195    497    9806      
538 22.63 13.49 8.25 contig10_pilon 250598 250886 (1601793) C rnd-4_family-877#Unknown (9806) 497 195 m_b688s001i1

  contig10_pilo     250598 GCACGGATGGACTCTGAACTCAGCT----ACACA--------------TA 250629
                                     i     i ii     ----     -------------- i
C rnd-4_family-        497 GCACGGATGGGCTCTGGATCCAGCTCGCCACACAGGTGAACGCGCCCCTG 448

  contig10_pilo     250630 CCCGATATC-------TTAATTAGATTTAATACGAAGAACCGGCATGTGA 250672
                             i  vi  -------  i        vi    v   vii          
C rnd-4_family-        447 CCTGAGGTCATAGACCTTGATTAGATTAGATACCAAGTGTCGGCATGTGA 398

  contig10_pilo     250673 TTGAACCCTCTTG-AGCTAGTGCACCCCTATGCT-TCAGGTCTATATAAC 250720
                              vivvv vvv - v  vi v         v  - v i          i
C rnd-4_family-        397 TTGCGGAATAAAGTACCTTATCCACCCCTATCCTGTGAAGTCTATATAAT 348

  contig10_pilo     250721 GTAGCACATGTTTGCCACAGTAGCCGCATTG-CCCTTGATCCCTTGTTCT 250769
                            i      v i     i  v  ---      -   i  --    i     
C rnd-4_family-        347 GCAGCACAAGCTTGCCGCACTA---GCATTGTCCCCTG--CCCTCGTTCT 303

  contig10_pilo     250770 CTCTGCTGCATTCTGAGCCTGCTCCTGAGCTCGTGCA-------TGCACT 250812
                           -----------------  i     i       i  i-------i    ?
C rnd-4_family-        302 -----------------CCCGCTCCCGAGCTCGCGCGCCGCCACCGCACN 270

  contig10_pilo     250813 TGAGCTCGCTCCCGCGCTCGTGCACTCACTGCGCCTGCACTTG----AGC 250858
                           i        v i            ---  v  i  ii   vi ----   
C rnd-4_family-        269 CGAGCTCGCGCTCGCGCTCGTGCA---ACGGCACCCACACGCGCTGTAGC 223

  contig10_pilo     250859 TCGCTCCCGCGGTCGTGCACAGGCATTG 250886
                           i        v v   i  i v v  ?i 
C rnd-4_family-        222 CCGCTCCCGAGCTCGCGCGCCGCCANCG 195

Matrix = 20p53g.matrix
Kimura (with divCpGMod) = 21.25
Transitions / transversions = 1.40 (35/25)
Gap_init rate = 0.11 (33 / 288), avg. gap size = 1.94 (64 / 33)

 
 

 
 
   +     277   16.4  6.6  0.0  contig10_pilon   252190  252250 (1600429) +  rnd-4_family-877   Unknown           5885   5949  (4354)    59     
 
 ANNOTATION EVIDENCE: 
   277  16.39 6.56 0.00  contig10_pilon   252190  252250   1600429 +  rnd-4_family-877   Unknown           5885   5949    4354      
277 16.39 6.56 0.00 contig10_pilon 252190 252250 (1600429) rnd-4_family-877#Unknown 5885 5949 (4354) m_b688s001i2

  contig10_pilo     252190 GCGCGTGCATTCGTGCGCGTTGAC----TCTGCGCATCCCCATTCGGGGG 252235
                                          v    i   ----       ii i     ?  i  
  rnd-4_family-       5885 GCGCGTGCATTCGTGGGCGTCGACACCATCTGCGCGCCTCCATTNGGAGG 5934

  contig10_pilo     252236 CGTGCAATTGTCCCA 252250
                           i     v i  v   
  rnd-4_family-       5935 TGTGCATTCGTACCA 5949

Matrix = 20p53g.matrix
Kimura (with divCpGMod) = 13.38
Transitions / transversions = 2.33 (7/3)
Gap_init rate = 0.02 (1 / 60), avg. gap size = 4.00 (4 / 1)

 
 
 
 
 
  +      11   12.1  4.8  7.3  contig10_pilon   252845  252886 (1599793) +  (TGCCGC)n          Simple_repeat        1     41     (0)    60     
 
 ANNOTATION EVIDENCE: 
    11  12.10 4.76 7.32  contig10_pilon   252845  252886   1599793 +  (TGCCGC)n          Simple_repeat        1     41       0      
11 12.10 4.76 7.32 contig10_pilon 252845 252886 (1599793) (TGCCGC)n#Simple_repeat 1 41 (0) m_b688s252i2

  contig10_pilo     252845 TGCGCGCTGCCGCTGCGTGTTGCTG-TGCCGGCTG-CGCTCCCG 252886
                              -            -i i   i -    -    -    v   
  (TGCCGC)n#Sim          1 TGC-CGCTGCCGCTGC-CGCTGCCGCTGCC-GCTGCCGCTGCCG 41

Matrix = Unknown
Transitions / transversions = 3.00 (3/1)
Gap_init rate = 0.12 (5 / 41), avg. gap size = 1.00 (5 / 5)

 
 

 
 
   +     475   14.3  0.0  0.0  contig10_pilon   254322  254405 (1598274) +  rnd-4_family-877   Unknown          10215  10298     (5)    61     
 
 ANNOTATION EVIDENCE: 
   475  14.29 0.00 0.00  contig10_pilon   254322  254405   1598274 +  rnd-4_family-877   Unknown          10215  10298       5      
475 14.29 0.00 0.00 contig10_pilon 254322 254405 (1598274) rnd-4_family-877#Unknown 10215 10298 (5) m_b688s001i3

  contig10_pilo     254322 TTCAAAATGTGCCAAAGTGCGGGAATCGCTTAACCGATGTGGTGCACAAA 254371
                            i?                     v   v          i v     i  
  rnd-4_family-      10215 TCNAAAATGTGCCAAAGTGCGGGATTCGGTTAACCGATGCGCTGCACGAA 10264

  contig10_pilo     254372 GTGCGGGTTTCGGTTAAGCGAAAAGTCCCGACAC 254405
                                 ???        v     ivi    ii  
  rnd-4_family-      10265 GTGCGGNNNTCGGTTAACCGAAAGTCCCCGGTAC 10298

Matrix = 20p53g.matrix
Kimura (with divCpGMod) = 14.03
Transitions / transversions = 1.40 (7/5)
Gap_init rate = 0.00 (0 / 83), avg. gap size = 0.0 (0 / 0)

 
 
 
 
 
  +      16   20.1  5.0  0.0  contig10_pilon   261159  261198 (1591481) +  GA-rich            Low_complexity       1     42     (0)    62     
 
 ANNOTATION EVIDENCE: 
    16  20.07 5.00 0.00  contig10_pilon   261159  261198   1591481 +  (GA)n              Simple_repeat        1     42       0      
16 20.07 5.00 0.00 contig10_pilon 261159 261198 (1591481) (GA)n#Simple_repeat 1 42 (0) m_b688s252i3

  contig10_pilo     261159 GAGTGCGAGAGAGAGAGAGAGCGTGAACGAGATA-AGA-AGA 261198
                              v v               v v  iv    v -   -   
  (GA)n#Simple_          1 GAGAGAGAGAGAGAGAGAGAGAGAGAGAGAGAGAGAGAGAGA 42

Matrix = Unknown
Transitions / transversions = 0.17 (1/6)
Gap_init rate = 0.05 (2 / 39), avg. gap size = 1.00 (2 / 2)

 
 

 
 
   +      19   15.4  0.0  4.4  contig10_pilon   261370  261416 (1591263) +  G-rich             Low_complexity       1     45     (0)    63     
 
 ANNOTATION EVIDENCE: 
    19  15.41 0.00 4.44  contig10_pilon   261370  261416   1591263 +  (GGGAG)n           Simple_repeat        1     45       0      
19 15.41 0.00 4.44 contig10_pilon 261370 261416 (1591263) (GGGAG)n#Simple_repeat 1 45 (0) m_b688s252i4

  contig10_pilo     261370 GGGAGGACAGGGGATGGGGTGGGAAGGGGAGTGGAGGGGGAGAGGAG 261416
                                 iv      -    v   i       v   -      i    
  (GGGAG)n#Simp          1 GGGAGGGGAGGGGA-GGGGAGGGGAGGGGAGGGGA-GGGGAGGGGAG 45

Matrix = Unknown
Transitions / transversions = 1.00 (3/3)
Gap_init rate = 0.04 (2 / 46), avg. gap size = 1.00 (2 / 2)

 
 
 
 
 
  +      14   24.7  0.0  0.0  contig10_pilon   267238  267275 (1585404) +  (GCG)n             Simple_repeat        1     38     (0)    64     
 
 ANNOTATION EVIDENCE: 
    14  24.72 0.00 0.00  contig10_pilon   267238  267275   1585404 +  (GCG)n             Simple_repeat        1     38       0      
14 24.72 0.00 0.00 contig10_pilon 267238 267275 (1585404) (GCG)n#Simple_repeat 1 38 (0) m_b688s252i5

  contig10_pilo     267238 GCGGCGGCGAGAGCGGCGGAGCCGGAGGCAGCTGCGGC 267275
                                    ivi       v v   v   i  v     
  (GCG)n#Simple          1 GCGGCGGCGGCGGCGGCGGCGGCGGCGGCGGCGGCGGC 38

Matrix = Unknown
Transitions / transversions = 0.60 (3/5)
Gap_init rate = 0.00 (0 / 37), avg. gap size = 0.0 (0 / 0)

 
 

 
 
   +      13   23.8  0.0  4.3  contig10_pilon   268628  268675 (1584004) +  (CATCCA)n          Simple_repeat        1     46     (0)    65     
 
 ANNOTATION EVIDENCE: 
    13  23.76 0.00 4.35  contig10_pilon   268628  268675   1584004 +  (CATCCA)n          Simple_repeat        1     46       0      
13 23.76 0.00 4.35 contig10_pilon 268628 268675 (1584004) (CATCCA)n#Simple_repeat 1 46 (0) m_b688s252i6

  contig10_pilo     268628 CATCCTGATCCTCCGTCCACGCCGCCCATCCTCGTCCACATCCACATC 268675
                                vv    -v i     ii - v     v i              
  (CATCCA)n#Sim          1 CATCCACATCC-ACATCCACATC-CACATCCACATCCACATCCACATC 46

Matrix = Unknown
Transitions / transversions = 0.80 (4/5)
Gap_init rate = 0.04 (2 / 47), avg. gap size = 1.00 (2 / 2)

 
 
 
 
 
  +      19   16.3  4.2  7.2  contig10_pilon   268861  268931 (1583748) +  (CCG)n             Simple_repeat        1     69     (0)    66     
 
 ANNOTATION EVIDENCE: 
    19  16.26 4.23 7.25  contig10_pilon   268861  268931   1583748 +  (CCG)n             Simple_repeat        1     69       0      
19 16.26 4.23 7.25 contig10_pilon 268861 268931 (1583748) (CCG)n#Simple_repeat 1 69 (0) m_b688s252i7

  contig10_pilo     268861 CCGCCGCCG-AGCCGCCG-CGCC-CCGACATGCGGTCGTACGCGCGGCGC 268907
                                    -v       -    -   - -i  v i  -v   -  v   
  (CCG)n#Simple          1 CCGCCGCCGCCGCCGCCGCCGCCGCCG-C-CGCCGCCG-CCGC-CGCCGC 46

  contig10_pilo     268908 CACCACGAGCCGCCGCCGCCGCCG 268931
                            i  i -v                
  (CCG)n#Simple         47 CGCCGC-CGCCGCCGCCGCCGCCG 69

Matrix = Unknown
Transitions / transversions = 0.80 (4/5)
Gap_init rate = 0.11 (8 / 70), avg. gap size = 1.00 (8 / 8)

 
 

 
 
   +      13   19.5  0.0  2.8  contig10_pilon   269028  269064 (1583615) +  (AGCCCA)n          Simple_repeat        1     36     (0)    67     
 
 ANNOTATION EVIDENCE: 
    13  19.49 0.00 2.78  contig10_pilon   269028  269064   1583615 +  (AGCCCA)n          Simple_repeat        1     36       0      
13 19.49 0.00 2.78 contig10_pilon 269028 269064 (1583615) (AGCCCA)n#Simple_repeat 1 36 (0) m_b688s252i8

  contig10_pilo     269028 AGCTCCAAGCCGACCTCCTCGCCCAAGCCCAAGCCCA 269064
                              -       v vvi  vv                 
  (AGCCCA)n#Sim          1 AGC-CCAAGCCCAAGCCCAAGCCCAAGCCCAAGCCCA 36

Matrix = Unknown
Transitions / transversions = 0.20 (1/5)
Gap_init rate = 0.03 (1 / 36), avg. gap size = 1.00 (1 / 1)

 
 
 
 
 
  +      16    8.5  0.0  0.0  contig10_pilon   280976  281000 (1571679) +  (GGC)n             Simple_repeat        1     25     (0)    68     
 
 ANNOTATION EVIDENCE: 
    16   8.53 0.00 0.00  contig10_pilon   280976  281000   1571679 +  (GGC)n             Simple_repeat        1     25       0      
16 8.53 0.00 0.00 contig10_pilon 280976 281000 (1571679) (GGC)n#Simple_repeat 1 25 (0) m_b688s252i9

  contig10_pilo     280976 GGCGGCGGCGGCGGCGGCGCAGGCG 281000
                                              vv    
  (GGC)n#Simple          1 GGCGGCGGCGGCGGCGGCGGCGGCG 25

Matrix = Unknown
Transitions / transversions = 0.00 (0/2)
Gap_init rate = 0.00 (0 / 24), avg. gap size = 0.0 (0 / 0)

 
 

 
 
   +      15    4.7  0.0  0.0  contig10_pilon   283810  283831 (1568848) +  (CTG)n             Simple_repeat        1     22     (0)    69     
 
 ANNOTATION EVIDENCE: 
    15   4.71 0.00 0.00  contig10_pilon   283810  283831   1568848 +  (CTG)n             Simple_repeat        1     22       0      
15 4.71 0.00 0.00 contig10_pilon 283810 283831 (1568848) (CTG)n#Simple_repeat 1 22 (0) m_b688s252i10

  contig10_pilo     283810 CTGCTCCTGCTGCTGCTGCTGC 283831
                                v                
  (CTG)n#Simple          1 CTGCTGCTGCTGCTGCTGCTGC 22

Matrix = Unknown
Transitions / transversions = 0.00 (0/1)
Gap_init rate = 0.00 (0 / 21), avg. gap size = 0.0 (0 / 0)

 
 
 
 
 
  +      30    0.0  0.0  0.0  contig10_pilon   291040  291067 (1561612) +  (CAGCCT)n          Simple_repeat        1     28     (0)    70     
 
 ANNOTATION EVIDENCE: 
    30   0.00 0.00 0.00  contig10_pilon   291040  291067   1561612 +  (CAGCCT)n          Simple_repeat        1     28       0      
30 0.00 0.00 0.00 contig10_pilon 291040 291067 (1561612) (CAGCCT)n#Simple_repeat 1 28 (0) c_b689s251i0

  contig10_pilo     291040 CAGCCTCAGCCTCAGCCTCAGCCTCAGC 291067
                                                       
  (CAGCCT)n#Sim          1 CAGCCTCAGCCTCAGCCTCAGCCTCAGC 28

Matrix = Unknown
Transitions / transversions = 1.00 (0/0)
Gap_init rate = 0.00 (0 / 27), avg. gap size = 0.0 (0 / 0)

 
 

 
 
   +      18    4.1  0.0  0.0  contig10_pilon   291108  291132 (1561547) +  (TCC)n             Simple_repeat        1     25     (0)    71     
 
 ANNOTATION EVIDENCE: 
    18   4.13 0.00 0.00  contig10_pilon   291108  291132   1561547 +  (TCC)n             Simple_repeat        1     25       0      
18 4.13 0.00 0.00 contig10_pilon 291108 291132 (1561547) (TCC)n#Simple_repeat 1 25 (0) m_b689s252i0

  contig10_pilo     291108 TCCTCCTCCTCCTCCTCCTCCACCT 291132
                                                v   
  (TCC)n#Simple          1 TCCTCCTCCTCCTCCTCCTCCTCCT 25

Matrix = Unknown
Transitions / transversions = 0.00 (0/1)
Gap_init rate = 0.00 (0 / 24), avg. gap size = 0.0 (0 / 0)

 
 
 
 
 
  +      29    0.0  0.0  0.0  contig10_pilon   291328  291354 (1561325) +  (CAG)n             Simple_repeat        1     27     (0)    72     
 
 ANNOTATION EVIDENCE: 
    29   0.00 0.00 0.00  contig10_pilon   291328  291354   1561325 +  (CAG)n             Simple_repeat        1     27       0      
29 0.00 0.00 0.00 contig10_pilon 291328 291354 (1561325) (CAG)n#Simple_repeat 1 27 (0) c_b689s251i1

  contig10_pilo     291328 CAGCAGCAGCAGCAGCAGCAGCAGCAG 291354
                                                      
  (CAG)n#Simple          1 CAGCAGCAGCAGCAGCAGCAGCAGCAG 27

Matrix = Unknown
Transitions / transversions = 1.00 (0/0)
Gap_init rate = 0.00 (0 / 26), avg. gap size = 0.0 (0 / 0)

 
 

 
 
   +      15   10.7  3.0  3.0  contig10_pilon   293313  293345 (1559334) +  (CGC)n             Simple_repeat        1     33     (0)    73     
 
 ANNOTATION EVIDENCE: 
    15  10.70 3.03 3.03  contig10_pilon   293313  293345   1559334 +  (CGC)n             Simple_repeat        1     33       0      
15 10.70 3.03 3.03 contig10_pilon 293313 293345 (1559334) (CGC)n#Simple_repeat 1 33 (0) m_b689s252i1

  contig10_pilo     293313 CGCC-CCGCCGTCGCTGCTCGCCGCCGTCGCCGC 293345
                               -      i   i  -        i      
  (CGC)n#Simple          1 CGCCGCCGCCGCCGCCGC-CGCCGCCGCCGCCGC 33

Matrix = Unknown
Transitions / transversions = 1.00 (3/0)
Gap_init rate = 0.06 (2 / 32), avg. gap size = 1.00 (2 / 2)

 
 
 
 
 
  +      15   16.3  0.0  2.9  contig10_pilon   295009  295044 (1557635) +  (GCGCAG)n          Simple_repeat        1     35     (0)    74     
 
 ANNOTATION EVIDENCE: 
    15  16.33 0.00 2.86  contig10_pilon   295009  295044   1557635 +  (GCGCAG)n          Simple_repeat        1     35       0      
15 16.33 0.00 2.86 contig10_pilon 295009 295044 (1557635) (GCGCAG)n#Simple_repeat 1 35 (0) m_b689s252i2

  contig10_pilo     295009 GCGCAGGCGCAATCGCGCGCCCAGGCGCAGGTCGCA 295044
                                      iv   iv  v          -    
  (GCGCAG)n#Sim          1 GCGCAGGCGCAGGCGCAGGCGCAGGCGCAGG-CGCA 35

Matrix = Unknown
Transitions / transversions = 0.67 (2/3)
Gap_init rate = 0.03 (1 / 35), avg. gap size = 1.00 (1 / 1)

 
 

 
 
   +    3484    6.9  0.6  0.2  contig10_pilon   325166  325670 (1527009) +  rnd-4_family-3166  Unknown              1    507     (0)    75     
 
 ANNOTATION EVIDENCE: 
  3484   6.94 0.59 0.20  contig10_pilon   325166  325670   1527009 +  rnd-4_family-3166  Unknown              1    507       0      
3484 6.94 0.59 0.20 contig10_pilon 325166 325670 (1527009) rnd-4_family-3166#Unknown 1 507 (0) m_b689s001i0

  contig10_pilo     325166 AAGGGGCAGGCTTCGGCAAATTCCGATACACGGACCGGGTGGGGGGTGGG 325215
                                      v        i    i                        
  rnd-4_family-          1 AAGGGGCAGGCGTCGGCAAACTCCGGTACACGGACCGGGTGGGGGGTGGG 50

  contig10_pilo     325216 TCCAGGCAAACACTTATAACTTTTTTTTATTTGCTTCCGAGGGGGGTCTG 325265
                                      i                                    i 
  rnd-4_family-         51 TCCAGGCAAACGCTTATAACTTTTTTTTATTTGCTTCCGAGGGGGGTCCG 100

  contig10_pilo     325266 GGAGGCCGATTTAAAAACAATCTGAATCTCCCACAGCCCATATCCAATTT 325315
                                  v    i  ? i?i    vi           ?       i?   
  rnd-4_family-        101 GGAGGCCCATTTGAANATNGTCTGCGTCTCCCACAGCNCATATCCGNTTT 150

  contig10_pilo     325316 TATTTGCTCGAAGAGCTCTAGCAGCAATGGAATCATGACTC-ATGCAGCA 325364
                                    v                               -        
  rnd-4_family-        151 TATTTGCTCCAAGAGCTCTAGCAGCAATGGAATCATGACTCTATGCAGCA 200

  contig10_pilo     325365 GTTTGGCCATCTGAGGAGCAGGAGCACGCCCGAGGCGGCGATATCCG-GT 325413
                           i                             v ii        i    - v
  rnd-4_family-        201 ATTTGGCCATCTGAGGAGCAGGAGCACGCCGGGAGCGGCGATGTCCGAGG 250

  contig10_pilo     325414 GCTTATGTAAG-GGTTTCCTTTTCCAGAGTTTACCTCCGCCCAGAGCGCG 325462
                                   v  -                i                     
  rnd-4_family-        251 GCTTATGTCAGCGGTTTCCTTTTCCAGAATTTACCTCCGCCCAGAGCGCG 300

  contig10_pilo     325463 GCTGCACTTGCCCTGCGTTTGTTGCGCTGCCGGACACTTTGGGGGTCGTC 325512
                              i       v      i                      iii    ? 
  rnd-4_family-        301 GCTACACTTGCGCTGCGTCTGTTGCGCTGCCGGACACTTTGAAAGTCGNC 350

  contig10_pilo     325513 TTTCAAGCATTTCACGGTAGTTGACCAAGTTTCGGTCCCATATACGTCAT 325562
                           v   i       ?    v                      ?     v   
  rnd-4_family-        351 ATTCGAGCATTTNACGGGAGTTGACCAAGTTTCGGTCCCANATACGGCAT 400

  contig10_pilo     325563 CCGCGGAGTCAAATTTGCTTCCGAGGGGGGAGGGGGGGGGGCTCAGCAAA 325612
                                 i                       -  i          i     
  rnd-4_family-        401 CCGCGGGGTCAAATTTGCTTCCGAGGGGGG-GGAGGGGGGGCTCGGCAAA 449

  contig10_pilo     325613 CCGCTTATGACTTTTTTTTGGGTGGGTGTCCACTATTTTCCCCTTGCCTG 325662
                                                   v                         
  rnd-4_family-        450 CCGCTTATGACTTTTTTTTGGGTGCGTGTCCACTATTTTCCCCTTGCCTG 499

  contig10_pilo     325663 CCCCTTGC 325670
                                   
  rnd-4_family-        500 CCCCTTGC 507

Matrix = 20p53g.matrix
Kimura (with divCpGMod) = 6.23
Transitions / transversions = 1.92 (23/12)
Gap_init rate = 0.01 (4 / 504), avg. gap size = 1.00 (4 / 4)

 
 
 
 
 
  +      19   24.5  0.0  7.8  contig10_pilon   327450  327532 (1525147) +  (GTCCCAA)n         Simple_repeat        1     77     (0)    76     
 
 ANNOTATION EVIDENCE: 
    19  24.49 0.00 7.79  contig10_pilon   327450  327532   1525147 +  (GTCCCAA)n         Simple_repeat        1     77       0      
19 24.49 0.00 7.79 contig10_pilon 327450 327532 (1525147) (GTCCCAA)n#Simple_repeat 1 77 (0) m_b689s252i3

  contig10_pilo     327450 GTCCCAAGAACCCAGTCCCAGGTCCCAACTTTTACAGGTCCTAAGAAATC 327499
                                   vv  v       i       --v iv  i    i   -vvi 
  (GTCCCAA)n#Si          1 GTCCCAAGTCCCAAGTCCCAAGTCCCAA--GTCCCAAGTCCCAAG-TCCC 47

  contig10_pilo     327500 GAGTCCCAAGTCCCAGCTTTCCTCAAGTCCCAA 327532
                           i              --vv   -          
  (GTCCCAA)n#Si         48 AAGTCCCAAGTCCCA--AGTCC-CAAGTCCCAA 77

Matrix = Unknown
Transitions / transversions = 0.67 (6/9)
Gap_init rate = 0.07 (6 / 82), avg. gap size = 1.00 (6 / 6)

 
 

 
 
   +      14   32.4  1.6  1.6  contig10_pilon   328058  328121 (1524558) +  (CCG)n             Simple_repeat        1     64     (0)    77     
 
 ANNOTATION EVIDENCE: 
    14  32.40 1.56 1.56  contig10_pilon   328058  328121   1524558 +  (CCG)n             Simple_repeat        1     64       0      
14 32.40 1.56 1.56 contig10_pilon 328058 328121 (1524558) (CCG)n#Simple_repeat 1 64 (0) m_b689s252i4

  contig10_pilo     328058 CCGCCGCTGCTGACGATCG-CGCGGCCGCTGCTGAGGCCAACGCCGCCGC 328106
                                  i  i v  -i  -   v     i  i vv   iv         
  (CCG)n#Simple          1 CCGCCGCCGCCGCCG-CCGCCGCCGCCGCCGCCGCCGCCGCCGCCGCCGC 49

  contig10_pilo     328107 CTTCGTCGCTGCTGC 328121
                            vi  i   i  i  
  (CCG)n#Simple         50 CGCCGCCGCCGCCGC 64

Matrix = Unknown
Transitions / transversions = 1.67 (10/6)
Gap_init rate = 0.03 (2 / 63), avg. gap size = 1.00 (2 / 2)

 
 
 
 
 
  +      14   20.5  1.7  7.3  contig10_pilon   330892  330949 (1521730) +  (CGTCTG)n          Simple_repeat        1     55     (0)    78     
 
 ANNOTATION EVIDENCE: 
    14  20.51 1.72 7.27  contig10_pilon   330892  330949   1521730 +  (CGTCTG)n          Simple_repeat        1     55       0      
14 20.51 1.72 7.27 contig10_pilon 330892 330949 (1521730) (CGTCTG)n#Simple_repeat 1 55 (0) m_b689s252i5

  contig10_pilo     330892 CGTCTGCCTCTGCGTCTCTGCGGCT-CGGCTGACGACGCCTGTACGCGTC 330940
                                  v      --      v  -  v   -  v vv -  vi     
  (CGTCTG)n#Sim          1 CGTCTGCGTCTGCG--TCTGCGTCTGCGTCTG-CGTCTGC-GTCTGCGTC 46

  contig10_pilo     330941 TGCGTGTGC 330949
                                v   
  (CGTCTG)n#Sim         47 TGCGTCTGC 55

Matrix = Unknown
Transitions / transversions = 0.12 (1/8)
Gap_init rate = 0.09 (5 / 57), avg. gap size = 1.00 (5 / 5)

 
 

 
 
   +    9080   13.7  4.9  2.0  contig10_pilon   340212  342182 (1510497) +  rnd-4_family-158   Unknown              1   1939  (9828)    79     
 
 ANNOTATION EVIDENCE: 
  9080  13.47 4.88 2.01  contig10_pilon   340212  342097   1510582 +  rnd-4_family-158   Unknown              1   1939    9828      
9080 13.47 4.88 2.01 contig10_pilon 340212 342097 (1510582) rnd-4_family-158#Unknown 1 1939 (9828) m_b689s001i1

  contig10_pilo     340212 TCGGGTCGTCTATGGACGGCCAGTAGTACTGGTATGGCGTCCATGTCGAT 340261
                               i      v                  v            i     -
  rnd-4_family-          1 TCGGATCGTCTCTGGACGGCCAGTAGTACTTGTATGGCGTCCACGTCGA- 49

  contig10_pilo     340262 TTCGCGTCCTCCGTCTCCCCCCAGGCTAACGCCAGTTCTGAGGGCGGCGA 340311
                           --     i     vv         vii       v      v        
  rnd-4_family-         50 --CGCGTTCTCCGGGTCCCCCCAGCTCAACGCCACTTCTGACGGCGGCGA 97

  contig10_pilo     340312 AGGTGCGCGCGGTATCGCTGGCGGTTTCCTCGCCTCCCAGAGCTCCCACG 340361
                              i  v v    ?                   i     v          
  rnd-4_family-         98 AGGCGCCCTCGGTNTCGCTGGCGGTTTCCTCGCTTCCCACAGCTCCCACG 147

  contig10_pilo     340362 CCACGCCGATGAGATGTTGCAATGTGGGATCTCTCTGCACAACGTCGATA 340411
                               i    i            v           v  v  ii        
  rnd-4_family-        148 CCACACCGACGAGATGTTGCAAGGTGGGATCTCTGTGGACGGCGTCGATA 197

  contig10_pilo     340412 GTCGGGATCATGCCCTGGTATTTCTTCGGGACAATCAGCTGAAACGAGGG 340461
                                  ?                     v  i               v 
  rnd-4_family-        198 GTCGGGANCATGCCCTGGTATTTCTTCGGCACGATCAGCTGAAACGAGCG 247

  contig10_pilo     340462 ATCGCCAAAGACCCGGCCGCCCTCCATGTATTGCCGCCAAGTCATCGTCG 340511
                                          vv  v             v                
  rnd-4_family-        248 ATCGCCAAAGACCCGTACGGCCTCCATGTATTGACGCCAAGTCATCGTCG 297

  contig10_pilo     340512 TGCCCACGACGAAGCCCTTCGTGCCGTGGTACTTGTTGATGCTGTGAAAT 340561
                               ---------------------    ii     i          i  
  rnd-4_family-        298 TGCC---------------------GTGGCGCTTGTCGATGCTGTGAGAT 326

  contig10_pilo     340562 ACACC----GTCAGATCTGTTTGACAGGTACCGTTGAGCGGCTCACTCAT 340607
                            vi  ----         i            v                  
  rnd-4_family-        327 AGGCCCCAGGTCAGATCTATTTGACAGGTACGGTTGAGCGGCTCACTCAT 376

  contig10_pilo     340608 AGCCCGCCAGGTCTTCAATGGTCAAGTCTTCTGGCCGTTTGACGGGCTCC 340657
                                             i   i  i     i  v        v      
  rnd-4_family-        377 AGCCCGCCAGGTCTTCAACGGTTAAATCTTCCGGGCGTTTGACCGGCTCC 426

  contig10_pilo     340658 CTACGTACATTCAGGACATTAGAATATACATTTTCAGCAGTGCTTACTCT 340707
                                      i        v        v   i      ii        
  rnd-4_family-        427 CTACGTACATTTAGGACATTCGAATATACCTTTCCAGCAGCACTTACTCT 476

  contig10_pilo     340708 TCCGTTCGCGACTCGTCCTCGCTGGCTCGTCCAAGCCGCTCCCTGCGCTG 340757
                                  vv  i        i     i      i       v      i 
  rnd-4_family-        477 TCCGTTCCGGATTCGTCCTCACTGGCCCGTCCAGGCCGCTCGCTGCGCCG 526

  contig10_pilo     340758 ATGTCAGCTGTGTTAATTGGCGAGTTGACAGACACGCACCGCTCATCGTT 340807
                               i   i     i v v      i    v  i                
  rnd-4_family-        527 ATGTTAGCCGTGTTGAATCGCGAGTCGACACACGCGCACCGCTCATCGTT 576

  contig10_pilo     340808 CCCGTGCCTCAGCTCGGGCGCCGTGCGAGCTCCGTCGCCGCTGCCGCCCT 340857
                                           vi          v                     
  rnd-4_family-        577 CCCGTGCCTCAGCTCGTACGCCGTGCGATCTCCGTCGCCGCTGCCGCCCT 626

  contig10_pilo     340858 CGCTGCCACAGCATGAGTCTCGGTCATCGTGTCAGCGTCATTGATGTTCC 340907
                                   i     i     v      v   i   v       i      
  rnd-4_family-        627 CGCTGCCATAGCATAAGTCTGGGTCATGGTGCCAGAGTCATTGGTGTTCC 676

  contig10_pilo     340908 TTACCCTCGAGACCGAGC-GGCGCCTTCCCGTCGC-TCCATCTCCACGCG 340955
                                   v         -     v          -   i          
  rnd-4_family-        677 TTACCCTCTAGACCGAGCGGGCGCGTTCCCGTCGCTTCCGTCTCCACGCG 726

  contig10_pilo     340956 CTCCTGC----------TCCT----CCCTCTGTCGCTCGAGCTTCTCTCT 340991
                               i  ----------    ----     i ?          i   i  
  rnd-4_family-        727 CTCCCGCCTCCGNTCGATCCTCTCCCCCTCCGNCGCTCGAGCTCCTCCCT 776

  contig10_pilo     340992 CTCGCTCTGCTCCCTCCTCGTCCTCCACGCCTCCATTTCCGCTTGGTTCA 341041
                                                                 v           
  rnd-4_family-        777 CTCGCTCTGCTCCCTCCTCGTCCTCCACGCCTCCATTTGCGCTTGGTTCA 826

  contig10_pilo     341042 TCCGCCGTTTCTTCCACTTTGGCATCGCGCTTCGCGGAATTCAGTCTGTA 341091
                                   ?vi                      v    v           
  rnd-4_family-        827 TCCGCCGTNGTTTCCACTTTGGCATCGCGCTTCCCGGATTTCAGTCTGTA 876

  contig10_pilo     341092 TCGGGCGGTG-AGCATCAATGAGGATGACTGGTCTCATTCGAGAGCCA-- 341138
                                     -            v  -                    i--
  rnd-4_family-        877 TCGGGCGGTGTAGCATCAATGAGCAT-ACTGGTCTCATTCGAGAGCCGAC 925

  contig10_pilo     341139 ---GCTGTACTCACATCCGTAGTATGAGCTGATCTGGGC--TGCGACGCC 341183
                           ---  i  ?     iv   i?  ?        ?      --    i  --
  rnd-4_family-        926 GCCGCCGTNCTCACGGCCGCNGTNTGAGCTGANCTGGGCGGTGCGGCG-- 973

  contig10_pilo     341184 GCCGATGCGACTTGAGCTTTGTCTA-ACAAATGTCTGCGCTTGGTACTCA 341232
                                 ?          vi  i?  - i              v       
  rnd-4_family-        974 GCCGATNCGACTTGAGCGCTGCNTAGATAAATGTCTGCGCTTCGTACTCA 1023

  contig10_pilo     341233 CGGTGACACACCTCGTCTGCGGCAGGCGGCGGGTGTTTTGGTTCGCTGTG 341282
                             ?    i i             i       i      v   v       
  rnd-4_family-       1024 CGNTGACGCGCCTCGTCTGCGGCGGGCGGCGAGTGTTTGGGTGCGCTGTG 1073

  contig10_pilo     341283 AAATATAATCAGCTTCGAAGAGGATGGCCTTGAGCGCAGACA---AGTAT 341329
                                    i           v    -----  v   v   i---i ii 
  rnd-4_family-       1074 AAATATAATTAGCTTCGAAGACGATG-----GATCGCCGACGTCCGGCGT 1118

  contig10_pilo     341330 GGCCTCTG---CATGGACG------------CGCGACTTGCGGTCGCCCA 341364
                             v i i --- ?? v   ------------      v vii i  -  i
  rnd-4_family-       1119 GGGCCCCGCGCCNNGCACGGCAGCGAGCCGCCGCGACATCTAGCCG-CCG 1167

  contig10_pilo     341365 CCGC---------GAG-TGA---AGCACTCGCCGCAGAGCAGGC-CGTAC 341400
                               ---------   -  i---   i         i   v   -     
  rnd-4_family-       1168 CCGCCTGACGACGGAGATGGTCCAGCGCTCGCCGCAAAGCTGGCGCGTAC 1217

  contig10_pilo     341401 ACCCGATCGCCAACGTCTGGCCTGGGCCCCGAGCTTCGCTCTGCGGCGAG 341450
                                      i     i   v         v                  
  rnd-4_family-       1218 ACCCGATCGCCGACGTCCGGCGTGGGCCCCGCGCTTCGCTCTGCGGCGAG 1267

  contig10_pilo     341451 -GTACATCTC---GT--CTACGACCGAGGCATGCATGATGACAGTGAACA 341494
                           - vi v    ---  -- iv  i  vvv  ii   -i v    i  -i i
  rnd-4_family-       1268 CGGGCCTCTCACGGTTCCCCCGGCCCTCGCGCGCA-AAAGACAATG-GCG 1315

  contig10_pilo     341495 AAA--ATCTACTCACGATTCTCGTGGGGGT---GCTTGATGTCGACAGGG 341539
                            ? --  v          iii   vv i i---   i i  v  iv   v
  rnd-4_family-       1316 ANATTATATACTCACGATCTCCGTCTGAGCCGAGCTCGGTGGCGGGAGGC 1365

  contig10_pilo     341540 AC--GGCAGT-----GCTCTGTCGAATTTCATTAGCATCGAAGACCATGA 341582
                           i --  v  v-----  v   -    vv vi i   v      ? v    
  rnd-4_family-       1366 GCCGGGAAGATTTTGGCACTG-CGAAGGTGGTCAGCTTCGAAGNCAATGA 1414

  contig10_pilo     341583 --CATTGA-GCCCAGGCGAGTTTTGCGTCTGCATCGACGCGCGACT--TG 341627
                           -- i v  -   vi      vviv v i   v v          i --  
  rnd-4_family-       1415 ATCGTAGAGGCCGGGGCGAGGACGGGGCCTGAAGCGACGCGCGATTGCTG 1464

  contig10_pilo     341628 CGATCTCCCACCGCGAGTAGGGCACTCGCCGCAGAGCAGGCCGTACACCC 341677
                              v  -v  v       - ii               -            
  rnd-4_family-       1465 CGAGCT-GCAACGCGAGT-GAACACTCGCCGCAGAGC-GGCCGTACACCC 1511

  contig10_pilo     341678 GATCGCCAACGTCTGGCCTGGGCCCCGAGCTTCGCTCTGCGGCGAGGTAC 341727
                                            v                   ?        vv  
  rnd-4_family-       1512 GATCGCCAACGTCTGGCGTGGGCCCCGAGCTTCGCTCNGCGGCGAGCGAC 1561

  contig10_pilo     341728 ATCT--CGTCTACGACCGAGGCATGCATGATGACAGTGAACAAAAATCTA 341775
                               --     i     v         vv i         v   v  v  
  rnd-4_family-       1562 ATCTGCCGTCTGCGACCCAGGCATGCAATACGACAGTGAAGAAACATGTA 1611

  contig10_pilo     341776 CTCACGATTCTCGTGGGGGTGCTCGATGTCGACAGGGACGGCAGTGCTCT 341825
                                 v       v v     v            v  v  v        
  rnd-4_family-       1612 CTCACGTTTCTCGTCGCGGTGCGCGATGTCGACAGTGAGGGGAGTGCTCT 1661

  contig10_pilo     341826 GTCGAATTTCATTAGCATCGAAGACCATGACATTGAGCCCA-GGCGAGTT 341874
                            iv      v      v        v     i         -        
  rnd-4_family-       1662 GCGGAATTTGATTAGCTTCGAAGACAATGACGTTGAGCCCAGGGCGAGTT 1711

  contig10_pilo     341875 TTGCGTCTGCATCGACGCGCGACTTGC----GATCTCCCACCGCGAGTAG 341920
                            v                    v    ----  v    v  v      ii
  rnd-4_family-       1712 TGGCGTCTGCATCGACGCGCGAGTTGCTGCAGAGCTCCAACAGCGAGTGA 1761

  contig10_pilo     341921 GGCACTCGCCGCAGAGCAGGCCGTACACCCGATCGCCAACGTCTGGCCTG 341970
                           i                                          i      
  rnd-4_family-       1762 AGCACTCGCCGCAGAGCAGGCCGTACACCCGATCGCCAACGTCCGGCCTG 1811

  contig10_pilo     341971 GGCCCCGAGCTTCGCTCTGCGGCGAGGTACATCTTGTCTACGACCGAGGC 342020
                                             i       vv      i    i     v    
  rnd-4_family-       1812 GGCCCCGAGCTTCGCTCTACGGCGAGCGACATCTCGTCTGCGACCCAGGC 1861

  contig10_pilo     342021 ATGCAGGACGACAGTGAACAAAAATCT-ACTCACGTTTCTCGTGGGCGTG 342069
                                   i         v  i  iv -           v   v vv   
  rnd-4_family-       1862 ATGCAGGATGACAGTGAAGAAGAACGTCACTCACGTTTCGCGTCGCGGTG 1911

  contig10_pilo     342070 CTCGATGTCGACAGGGACGGCAGTGCTC 342097
                            v i    v   v  v v  v  i  i 
  rnd-4_family-       1912 CGCAATGTGGACTGGCAGGGGAGCGCCC 1939

Matrix = 20p53g.matrix
Kimura (with divCpGMod) = 12.40
Transitions / transversions = 0.92 (119/130)
Gap_init rate = 0.04 (70 / 1885), avg. gap size = 1.87 (131 / 70)

  1967  18.55 5.06 1.08  contig10_pilon   341649  342182   1510497 +  rnd-4_family-158   Unknown           1192   1746   10021      
1967 18.55 5.06 1.08 contig10_pilon 341649 342182 (1510497) rnd-4_family-158#Unknown 1192 1746 (10021) m_b689s001i2

  contig10_pilo     341649 GCACTCGCCGCAGAGCAGGC-CGTACACCCGATCGCCAACGTCTGGCCTG 341697
                             i         i   v   -                i     i   v  
  rnd-4_family-       1192 GCGCTCGCCGCAAAGCTGGCGCGTACACCCGATCGCCGACGTCCGGCGTG 1241

  contig10_pilo     341698 GGCCCCGAGCTTCGCTCTGCGGCGAG-GTACATCTC---GT--CTACGAC 341741
                                  v                  - vi v    ---  -- iv  i 
  rnd-4_family-       1242 GGCCCCGCGCTTCGCTCTGCGGCGAGCGGGCCTCTCACGGTTCCCCCGGC 1291

  contig10_pilo     341742 CGAGGCATGCATGATGACAGTGAACAAAA--ATCTACTCACGATTCTCGT 341789
                            vvv  ii   vi -    i  i- i ? --  v          iii   
  rnd-4_family-       1292 CCTCGCGCGCAAAA-GACAATGG-CGANATTATATACTCACGATCTCCGT 1339

  contig10_pilo     341790 GGGGGT---GCTCGATGTCGACAGGGAC--GGCAGT-----GCTCTGTCG 341829
                           vv i i---     i  v  iv   vi --  v  v-----  v   -  
  rnd-4_family-       1340 CTGAGCCGAGCTCGGTGGCGGGAGGCGCCGGGAAGATTTTGGCACTG-CG 1388

  contig10_pilo     341830 AATTTCATTAGCATCGAAGACCATGA--CATTGAGCCCAGG-CGAGTTTT 341876
                             vv vi i   v      ? v    -- i v   v  i  -    vviv
  rnd-4_family-       1389 AAGGTGGTCAGCTTCGAAGNCAATGAATCGTAGAGGCCGGGGCGAGGACG 1438

  contig10_pilo     341877 GCGTCTGCATCGACGCGCGACT--TGCGATCTCCCACCGCGAGTAGGGCA 341924
                            v i   v v          i --     v  v v  -      - ii  
  rnd-4_family-       1439 GGGCCTGAAGCGACGCGCGATTGCTGCGAGCTGCAAC-GCGAGT-GAACA 1486

  contig10_pilo     341925 CTCGCCGCAGAGCAGGCCGTACACCCGATCGCCAACGTCTGGCCTGGGCC 341974
                                        -                             v      
  rnd-4_family-       1487 CTCGCCGCAGAGC-GGCCGTACACCCGATCGCCAACGTCTGGCGTGGGCC 1535

  contig10_pilo     341975 CCGAGCTTCGCTCTGCGGCGAGGTACATCT--TGTCTACGACCGAGGCAT 342022
                                        ?        vv      --i    i     v      
  rnd-4_family-       1536 CCGAGCTTCGCTCNGCGGCGAGCGACATCTGCCGTCTGCGACCCAGGCAT 1585

  contig10_pilo     342023 GCAGGACGACAGTGAACAAAAATCTACTCACGTTTCTCGTGGGCGTGCTC 342072
                              iv           v   v  v                v vv    v 
  rnd-4_family-       1586 GCAATACGACAGTGAAGAAACATGTACTCACGTTTCTCGTCGCGGTGCGC 1635

  contig10_pilo     342073 GATGTCGACAGGGACGGCAGTGCTCTGCAAAATTTTATTAGCTTCGATGA 342122
                                      v  v  v          ii     v           v  
  rnd-4_family-       1636 GATGTCGACAGTGAGGGGAGTGCTCTGCGGAATTTGATTAGCTTCGAAGA 1685

  contig10_pilo     342123 CAATGGTGTCGAGCCCA-AGCGAGAATAGTGACTGCGTCGACGCGCGACT 342171
                                ii  i       -i     vv i i v    i           v 
  rnd-4_family-       1686 CAATGACGTTGAGCCCAGGGCGAGTTTGGCGTCTGCATCGACGCGCGAGT 1735

  contig10_pilo     342172 TGCTGCAGAGC 342182
                                      
  rnd-4_family-       1736 TGCTGCAGAGC 1746

Matrix = 20p53g.matrix
Kimura (with divCpGMod) = 18.34
Transitions / transversions = 0.75 (42/56)
Gap_init rate = 0.04 (19 / 533), avg. gap size = 1.74 (33 / 19)

 
 
 
 
 
  +   22533   11.7  0.8  0.8  contig10_pilon   342197  345962 (1506717) +  rnd-4_family-158   Unknown           1100   5429  (6338)    80     
 
 ANNOTATION EVIDENCE: 
   467  25.63 0.54 8.82  contig10_pilon   342197  342380   1510299 +  rnd-4_family-158   Unknown           1100   1269   10498      
467 25.63 0.54 8.82 contig10_pilon 342197 342380 (1510299) rnd-4_family-158#Unknown 1100 1269 (10498) m_b689s001i3

  contig10_pilo     342197 GATCGCCTAGGTAGGGCGTGGGTCTCGCAACCTGCACTGCAGCAAGTCTC 342246
                                  v v  vv        i i   iv ??    v     i  i v 
  rnd-4_family-       1100 GATCGCCGACGTCCGGCGTGGGCCCCGCGCCNNGCACGGCAGCGAGCCGC 1149

  contig10_pilo     342247 GAGTGTCATGCAGCCACAGAAACTGAGTGGCGGATAATATCTCCAGCAGC 342296
                           v- i v   vi    i v vvi iviii i    v vvi  i--------
  rnd-4_family-       1150 C-GCGACATCTAGCCGCCGCCGCCTGACGACGGAGATGGTCC-------- 1190

  contig10_pilo     342297 GAGTGAAGCGCTCGCCGCAGAGCAGGC-CGTACACCCGATCGCCAACGTC 342345
                           ------             i   v   -                i     
  rnd-4_family-       1191 ------AGCGCTCGCCGCAAAGCTGGCGCGTACACCCGATCGCCGACGTC 1234

  contig10_pilo     342346 TGGCCTGGGCCCCAAGCCTTGCTCTACGGCGAGCG 342380
                           i   v        iv  i i     i         
  rnd-4_family-       1235 CGGCGTGGGCCCCGCGCTTCGCTCTGCGGCGAGCG 1269

Matrix = 20p53g.matrix
Kimura (with divCpGMod) = 23.79
Transitions / transversions = 1.15 (23/20)
Gap_init rate = 0.09 (16 / 183), avg. gap size = 1.00 (16 / 16)

 22533  11.33 0.84 0.62  contig10_pilon   342287  345962   1506717 +  rnd-4_family-158   Unknown           1746   5429    6338      
22533 11.33 0.84 0.62 contig10_pilon 342287 345962 (1506717) rnd-4_family-158#Unknown 1746 5429 (6338) m_b689s001i4

  contig10_pilo     342287 CTCCAGCAGCGAGTGAAGCGCTCGCCGCAGAGCAGGCCGTACACCCGATC 342336
                                i             i                              
  rnd-4_family-       1746 CTCCAACAGCGAGTGAAGCACTCGCCGCAGAGCAGGCCGTACACCCGATC 1795

  contig10_pilo     342337 GCCAACGTCTGGCCTGGGCCCCAAGCCTTGCTCTACGGCGAGCGACATCT 342386
                                    i            i   i i                     
  rnd-4_family-       1796 GCCAACGTCCGGCCTGGGCCCCGAGCTTCGCTCTACGGCGAGCGACATCT 1845

  contig10_pilo     342387 CGTGTGCCACCCAGGCAAGCAGAATGACAGCGAAGAAAATCGT-ACTCAC 342435
                              v   v         v    i       i      i v   -      
  rnd-4_family-       1846 CGTCTGCGACCCAGGCATGCAGGATGACAGTGAAGAAGAACGTCACTCAC 1895

  contig10_pilo     342436 AGTTCGCGTCGCGGTGCGCAATGTGGACTGGCAGGGGAGCACCCACGTGT 342485
                           iv                                      i       v 
  rnd-4_family-       1896 GTTTCGCGTCGCGGTGCGCAATGTGGACTGGCAGGGGAGCGCCCACGTCT 1945

  contig10_pilo     342486 GTGCGCGCGAAGTTGGGTAGATGTATTACCTGCGCTGCGACTACGGCCAC 342535
                                       i-i v i   i            i              
  rnd-4_family-       1946 GTGCGCGCGAAGC-AGTTGGATATATTACCTGCGCCGCGACTACGGCCAC 1994

  contig10_pilo     342536 ACCTAACGTCTAGGGTGACCGGCGCAGAGCAAGGCGGGGAAGGGGTGGGC 342585
                                            i                      i        v
  rnd-4_family-       1995 ACCTAACGTCTAGGGTGGCCGGCGCAGAGCAAGGCGGGGAGGGGGTGGGG 2044

  contig10_pilo     342586 TTGCGGGGGTGCTGATGGCGTTGGACGCCGTGAGCGATGGTGGTCGGGAA 342635
                                   -                         i i             
  rnd-4_family-       2045 TTGCGGGG-TGCTGATGGCGTTGGACGCCGTGAGTGGTGGTGGTCGGGAA 2093

  contig10_pilo     342636 GATATCGGCCAAAGCCGAGACTGCCCAGTCACAGAGAGTCGGACTTGGGC 342685
                              i       v                   v--------          
  rnd-4_family-       2094 GATGTCGGCCACAGCCGAGACTGCCCAGTCAG--------GGACTTGGGC 2135

  contig10_pilo     342686 GGAT-TTTGCCCAAGTGCCCCGCCGAAATGCATCCGGACTCGTCTCCCCG 342734
                            i v- ii        v vv           v       i        i 
  rnd-4_family-       2136 GAAAATCCGCCCAAGTCCAGCGCCGAAATGCTTCCGGACCCGTCTCCCTG 2185

  contig10_pilo     342735 CATCGGCAGCCCTATGATACTAATGTCATGCGTGTTGGTGAGCATGCCCA 342784
                              v         ii   v i        v                vv  
  rnd-4_family-       2186 CATGGGCAGCCCTGCGATCCCAATGTCATTCGTGTTGGTGAGCATGGGCA 2235

  contig10_pilo     342785 GGTCATACAGAACAAAACGAGCACTTCACTAATTTACTCCGTTGTGTCCT 342834
                            v    v       iv i           i      i     i i     
  rnd-4_family-       2236 GCTCATTCAGAACAGTATGAGCACTTCACCAATTTATTCCGTCGCGTCCT 2285

  contig10_pilo     342835 CTTTCCGTTCTCGGTCGATTT------TC-TCGTTCACCGCAGTGCATAT 342877
                                i    v i        ------  -i     v i     i     
  rnd-4_family-       2286 CTTTCTGTTCGCAGTCGATTTCTCGTTTCGCCGTTCCCTGCAGTACATAT 2335

  contig10_pilo     342878 CTTAACTTGTTTATTACCGTCTGTTGCTCGTCCTCATGAGATAGCCCTGG 342927
                           vv   v  v v    i i  vi              v             
  rnd-4_family-       2336 GGTAAGTTTTGTATTGCTGTGCGTTGCTCGTCCTCAAGAGATAGCCCTGG 2385

  contig10_pilo     342928 TTGAGATTCAAAGAGTCAGTACCATCTCTGTTGAAGCCAGTCAAAATGTT 342977
                                      v  v        i        v     i   i   i   
  rnd-4_family-       2386 TTGAGATTCAATGACTCAGTACCGTCTCTGTTCAAGCCGGTCGAAACGTT 2435

  contig10_pilo     342978 CCTTACCCACAAATCCTCCGTTTGACAGCTGAGACTGAGGTGAGAAGTCC 343027
                                                 iv    v     i iv            
  rnd-4_family-       2436 CCTTACCCACAAATCCTCCGTTCCACAGATGAGATTACGGTGAGAAGTCC 2485

  contig10_pilo     343028 ATTCTGCTTTGCAAACCGACAATGAACTCCATTATCCAGTGGTCCCACTG 343077
                            i v  vv v v    -   v  v    i               ii    
  rnd-4_family-       2486 ACTGTGAATGGAAAAC-GACCATCAACTTCATTATCCAGTGGTCTTACTG 2534

  contig10_pilo     343078 TTCCTACCGTGCCTTCGAGGTGAGAATGAGGTCACGTGACCTGTTGACCG 343127
                                    v            v v i      i         i   i  
  rnd-4_family-       2535 TTCCTACCGAGCCTTCGAGGTGTGCACGAGGTCGCGTGACCTGCTGATCG 2584

  contig10_pilo     343128 CTGGACACATTCGCCCTGGTGTTCGCGCGGATGATGCTGGGCTGGTGGTG 343177
                           vi    v i   v         v                       v  v
  rnd-4_family-       2585 GCGGACCCGTTCCCCCTGGTGTGCGCGCGGATGATGCTGGGCTGGTCGTT 2634

  contig10_pilo     343178 TGGCTGCACATGGGCCTCCAATGCGCGTCGCCGTGCATCCATAG-CGTCA 343226
                                      v                                -     
  rnd-4_family-       2635 TGGCTGCACATCGGCCTCCAATGCGCGTCGCCGTGCATCCATAGGCGTCA 2684

  contig10_pilo     343227 TAACGCATTTTCCGCCCCACGAGGACGCGCTACACGCAACATATTTCATG 343276
                                 v         i                                 
  rnd-4_family-       2685 TAACGCCTTTTCCGCCTCACGAGGACGCGCTACACGCAACATATTTCATG 2734

  contig10_pilo     343277 TAGTCGTCCCGACGCGCAATGGAAGCCGATGTGCCTCCACACCCCAGCCA 343326
                                   v                     i                   
  rnd-4_family-       2735 TAGTCGTCGCGACGCGCAATGGAAGCCGATATGCCTCCACACCCCAGCCA 2784

  contig10_pilo     343327 CTTGAGGCCTACTTCGACTGTCATGCGCGGTTTTGCGCGCAATACGACGC 343376
                           i v v v         vviv                         i i  
  rnd-4_family-       2785 TTGGTGTCCTACTTCGCGCTTCATGCGCGGTTTTGCGCGCAATACAATGC 2834

  contig10_pilo     343377 AGGAGCGGCGCTGGGACGGCTAGGTGCCCGTCGGTCGCCTGTCTGGTCGT 343426
                           ii    i        v v       i       v  i v     i  i i
  rnd-4_family-       2835 GAGAGCAGCGCTGGGCCTGCTAGGTACCCGTCGTTCACGTGTCTAGTTGC 2884

  contig10_pilo     343427 CTGTGGTTGGTTTGAGGTTGAGCGTGCTAACTCCGCACGCGCGCGCTCGC 343476
                             v         i   i         v                 --    
  rnd-4_family-       2885 CTCTGGTTGGTTCGAGATTGAGCGTGATAACTCCGCACGCGCGC--TCGC 2932

  contig10_pilo     343477 CGGCCGCCCAAACGGGAAGTGATCTTATCGGTATCTCCAGATTGGTCCCG 343526
                             i        v             i     i      i           
  rnd-4_family-       2933 CGACCGCCCAATCGGGAAGTGATCTCATCGGCATCTCCGGATTGGTCCCG 2982

  contig10_pilo     343527 CGCCACACGATCCTCACCTCA---CCGCCGCCGCCCCGCATGCGCGTGTA 343573
                                    i   i i  v v---  i?                   ?  
  rnd-4_family-       2983 CGCCACACGGTCCCCGCCACTGCCCCANCGCCGCCCCGCATGCGCGTNTA 3032

  contig10_pilo     343574 CGGCGCACCTGC-ATGCCGGATATCTAGACTGCAGGTGAGATGACTCAGA 343622
                            i    ii    -        i   i  -   v                 
  rnd-4_family-       3033 CAGCGCGTCTGCCATGCCGGACATCCAG-CTGGAGGTGAGATGACTCAGA 3081

  contig10_pilo     343623 TATCTCACCCCGATAGCTC---------TCTCAGTCTACTTGCTATGAGT 343663
                           v        v  i v    ---------               v    v 
  rnd-4_family-       3082 GATCTCACCACGGTCGCTCCTTGCGCTCTCTCAGTCTACTTGCAATGACT 3131

  contig10_pilo     343664 TCCCCGGACATCGCTTCAGGTGTTGCTTGCTCCAGCAGTCAGGTCTATGC 343713
                              v                  v    i   v i           ?    
  rnd-4_family-       3132 TCCACGGACATCGCTTCAGGTGATGCTCGCTGCGGCAGTCAGGTCNATGC 3181

  contig10_pilo     343714 AGATGCTGACACTGTCAAGGTCCCTACGCGGCCGCGGTCCAACTCGACAT 343763
                            i                       v                        
  rnd-4_family-       3182 AAATGCTGACACTGTCAAGGTCCCTTCGCGGCCGCGGTCCAACTCGACAT 3231

  contig10_pilo     343764 GGAGCAG--ATTTTCCTGTCCGGACAACTCGATCGCCCCCTCACCGCTGC 343811
                                  --v                      i             v  i
  rnd-4_family-       3232 GGAGCAGCTTTTTTCCTGTCCGGACAACTCGACCGCCCCCTCACCGGTGT 3281

  contig10_pilo     343812 CGACTCCATTCTCGACCATGCCTTGTTGGCCGCTGCTGCCGATCGCGAGC 343861
                                  i         ii        v         v   ?        
  rnd-4_family-       3282 CGACTCCGTTCTCGACCGCGCCTTGTTCGCCGCTGCTCCCGNTCGCGAGC 3331

  contig10_pilo     343862 ACGCATCCATGGTCCAGCATTTAGAGGGCAGCCAAAGTCAGAATGTCCCG 343911
                                   ii                   i  v           i  v  
  rnd-4_family-       3332 ACGCATCCGCGGTCCAGCATTTAGAGGGCGGCGAAAGTCAGAATATCGCG 3381

  contig10_pilo     343912 TCCGACTCCTCCGAAGATGTGCCGCTAGCTGCCTTGCGCTCAAAATCACG 343961
                             ?     v        i        v  v                 i ?
  rnd-4_family-       3382 TCNGACTCGTCCGAAGACGTGCCGCTCGCGGCCTTGCGCTCAAAATCGCN 3431

  contig10_pilo     343962 TCGTACTGGTTCAAGCTCGTCCGAGGACATTCCTCTAGCACACCAGGCCC 344011
                                 ? vv           v              v   v v       
  rnd-4_family-       3432 TCGTACNGCGTCAAGCTCGTCAGAGGACATTCCTCTCGCAGAGCAGGCCC 3481

  contig10_pilo     344012 GCGCGCGCCACGCAGCGGA-GAAAGGCACGGAACCCGCACTCTCAGTGGC 344060
                                             v-i   i                   i   v 
  rnd-4_family-       3482 GCGCGCGCCACGCAGCGGCCAAAAAGCACGGAACCCGCACTCTCGGTGTC 3531

  contig10_pilo     344061 AAGACACGAAGCTTCGCCCGCTGCAGCGCGTCCCGCACCCCGCGATGGCA 344110
                             viv ?  i ?   ?                             i    
  rnd-4_family-       3532 AACGAANGAGGNTTCNCCCGCTGCAGCGCGTCCCGCACCCCGCGACGGCA 3581

  contig10_pilo     344111 TTGCGCAACTCATTCCCGATCTCCGAATTCGCGAGTGCGTTCACACGCCA 344160
                                                 vv  v            i          
  rnd-4_family-       3582 TTGCGCAACTCATTCCCGATCTGGGACTTCGCGAGTGCGCTCACACGCCA 3631

  contig10_pilo     344161 CCCCGAGGCGCCGCGCAATATCTCACGCCAAAGTAGCTCTTCACAGGCCG 344210
                            v    v         i  i      ?   v  i      v i       
  rnd-4_family-       3632 CACCGATGCGCCGCGCGATGTCTCACNCCACAGCAGCTCTGCGCAGGCCG 3681

  contig10_pilo     344211 TTTCCGCGGCGAGGAGCGGCATGTTGCCTCAACCACTGCTTGCTGGGCAC 344260
                             v     v         i i    ?                       i
  rnd-4_family-       3682 TTGCCGCGTCGAGGAGCGACGTGTTNCCTCAACCACTGCTTGCTGGGCAT 3731

  contig10_pilo     344261 CTCAGGTTCATTTTCTCAGTGTGACCCTGACATCTGCACGACTTTGACAT 344310
                           v                   v          i         v i      
  rnd-4_family-       3732 GTCAGGTTCATTTTCTCAGTCTGACCCTGACGTCTGCACGAATCTGACAT 3781

  contig10_pilo     344311 GTCCTACGACGCAGCACGGCTCCATCTCCCACGGATATCAAGAAGGCCAA 344360
                           v        i          ?  i                          
  rnd-4_family-       3782 CTCCTACGATGCAGCACGGCNCCGTCTCCCACGGATATCAAGAAGGCCAA 3831

  contig10_pilo     344361 GAAATTATGGAGGAACTGGCAAAGCATGAACCGCAGAAAGACTCGCCAGG 344410
                               viv     i  i    i                  i          
  rnd-4_family-       3832 GAAAACCTGGAGAAATTGGCGAAGCATGAACCGCAGAAAAACTCGCCAGG 3881

  contig10_pilo     344411 CCATTGCGAACTCTAGTAAGCGTCTCATTTCTGCTCGAACATGCGATTGA 344460
                                       v    vv       iv v    i      v     v  
  rnd-4_family-       3882 CCATTGCGAACTGTAGTTCGCGTCTCGGTGCTGCCCGAACAGGCGATGGA 3931

  contig10_pilo     344461 GCTGATATTGCGCTGCTGCAGAACATGTTCCCGGGATACGCCTCGCAGCG 344510
                                vi i   v  i          i i                  ?  
  rnd-4_family-       3932 GCTGAGGTCGCGATGTTGCAGAACATATCCCCGGGATACGCCTCGCANCG 3981

  contig10_pilo     344511 TTCACAGATGCCACGCAGGAGAACTGGACCGGGAAGAA-ATTCCCACGCA 344559
                                v        i              v  vv v  -           
  rnd-4_family-       3982 TTCACCGATGCCACACAGGAGAACTGGACAGGTCATAAGATTCCCACGCA 4031

  contig10_pilo     344560 GCCTGACAGCGTGAGATGCGAGGGGACAGAAGCTTTTGTCTTGCGCGACG 344609
                             v   i     iv v     i  v        i    i           
  rnd-4_family-       4032 GCGTGATAGCGTACGCTGCGAAGGTACAGAAGCCTTTGCCTTGCGCGACG 4081

  contig10_pilo     344610 ATGACGGTGTCGCGCTATGCTGGGTCTTTCCAAACTTCCTCGGTGACGAC 344659
                                         i                            i      
  rnd-4_family-       4082 ATGACGGTGTCGCGTTATGCTGGGTCTTTCCAAACTTCCTCGGCGACGAC 4131

  contig10_pilo     344660 GCTCACGCAAGTCTCCGAGTTCTGCTGTACATCTGAGGCGCTGAGCTCAC 344709
                                i i          i i      i ii v   v vi i v   v  
  rnd-4_family-       4132 GCTCATGTAAGTCTCCGAATCCTGCTGCATGTGTGACGGACCGCGCTGAC 4181

  contig10_pilo     344710 TTCATTCACAGAAACTACTGTTCCGATATCTCCAGGACTTCGGCGAGGAC 344759
                                        i  i       ii  i                    i
  rnd-4_family-       4182 TTCATTCACAGAAGCTGCTGTTCCAGTACCTCCAGGACTTCGGCGAGGAT 4231

  contig10_pilo     344760 GTGGAAATCTCTCTCGACACTCGGCCGGGTGCCGATGCCAACACTCGCAA 344809
                                i  i          vv     i           i  i        
  rnd-4_family-       4232 GTGGAGATTTCTCTCGACAAGCGGCCAGGTGCCGATGCTAATACTCGCAA 4281

  contig10_pilo     344810 CAAGCGCAGTGCATACAACCGCAGTAACATTAAGGGAGAGATCTCGGGCT 344859
                                       v          ii    vvi        v i  v    
  rnd-4_family-       4282 CAAGCGCAGTGCCTACAACCGCAACAACAAGGAGGGAGAGCTTTCTGGCT 4331

  contig10_pilo     344860 GCTTCCAACTCGTCAACATATGGAATGCCATCGGACACGACGTGAGTATC 344909
                               i     i     i                      v      i   
  rnd-4_family-       4332 GCTTTCAACTTGTCAATATATGGAATGCCATCGGACACGCCGTGAGCATC 4381

  contig10_pilo     344910 GTCCAAACTTGCACGTATGACACGGCTGATGACCGTCGCCAGTTGCAAGG 344959
                                 v       v    v    i         ii   vvv i      
  rnd-4_family-       4382 GTCCAATCTTGCACCTATGTCACGACTGATGACCACCGCGTTTCGCAAGG 4431

  contig10_pilo     344960 GCGGGCAAGCTGGTCCCGCCACTGACGTTGTTGGCGGTCGCGTGAAGGAT 345009
                                     i                                       
  rnd-4_family-       4432 GCGGGCAAGCCGGTCCCGCCACTGACGTTGTTGGCGGTCGCGTGAAGGAT 4481

  contig10_pilo     345010 TTCAACGCGGCGATGCTTCTCATGGACAAGCTGAGAGTCGTCTCTCAGCG 345059
                                   i           v        i        ?           
  rnd-4_family-       4482 TTCAACGCAGCGATGCTTCTAATGGACAAACTGAGAGTNGTCTCTCAGCG 4531

  contig10_pilo     345060 TATCGACCACCTTCTGCAGTGCATCGACCCGGACCAACACGCGCTCCTCA 345109
                                             i                    i          
  rnd-4_family-       4532 TATCGACCACCTTCTGCAATGCATCGACCCGGACCAACATGCGCTCCTCA 4581

  contig10_pilo     345110 CCGCAGCAAGAGATCAGCTGAGAAACGCTATCCCGTCCTACAAGATCCTG 345159
                            v                                               v
  rnd-4_family-       4582 CGGCAGCAAGAGATCAGCTGAGAAACGCTATCCCGTCCTACAAGATCCTC 4631

  contig10_pilo     345160 TCTCAATATGATCCCACATATTTCCACGGCCGGTCACTCATCT-ACAATC 345208
                                   i        i              v          -      
  rnd-4_family-       4632 TCTCAATACGATCCCACGTATTTCCACGGCCGTTCACTCATCTAACAATC 4681

  contig10_pilo     345209 GGCAAACGGCCCTGCACACAGACCGGCGTGATAAAAAATTCGCATGGACG 345258
                                                    v  i  i     i            
  rnd-4_family-       4682 GGCAAACGGCCCTGCACACAGACCGCCGCGACAAAAAGTTCGCATGGACG 4731

  contig10_pilo     345259 CCAGTTCTCACGATTGGCAACTACACTGAGGGTTGCTTCAAGGTCCTCAA 345308
                                                                             
  rnd-4_family-       4732 CCAGTTCTCACGATTGGCAACTACACTGAGGGTTGCTTCAAGGTCCTCAA 4781

  contig10_pilo     345309 CCACGACATCGACTACATGCCCGGCACCCTCATTCTCCTTCGCGGCGCCG 345358
                              i                       i                      
  rnd-4_family-       4782 CCATGACATCGACTACATGCCCGGCACTCTCATTCTCCTTCGCGGCGCCG 4831

  contig10_pilo     345359 TTTTTCCTCACAGAGTCACATACAGCGGCGGGCAACGCATCTGCATCGCG 345408
                            i  i                                             
  rnd-4_family-       4832 TCTTCCCTCACAGAGTCACATACAGCGGCGGGCAACGCATCTGCATCGCG 4881

  contig10_pilo     345409 CATTTCACGCATGAGAGCATCGTCAATCGCACGACCGTCCGCGCGCTGCC 345458
                                      i                                      
  rnd-4_family-       4882 CATTTCACGCACGAGAGCATCGTCAATCGCACGACCGTCCGCGCGCTGCC 4931

  contig10_pilo     345459 TCTCATGACCGTCGAACAGGTTGCGGCTCGCCTCGCCAAGCATGCGGAGA 345508
                                    v  i ii        i            v            
  rnd-4_family-       4932 TCTCATGACAGTTGGGCAGGTTGCAGCTCGCCTCGCCCAGCATGCGGAGA 4981

  contig10_pilo     345509 AACCTGAGCACACCGCCGGCTGACGAGCTCAAGATATACAAATGGTCCCC 345558
                                     ------            v      ivi         v i
  rnd-4_family-       4982 AACCTGAGCA------CGGCTGACGAGCACAAGATGAGCAAATGGTCGCT 5025

  contig10_pilo     345559 CTGTAGTTACGGATGCGTGTCCGACAATTGACGACGGCGGTTTTGCGCCA 345608
                                      v i        v         v            i    
  rnd-4_family-       5026 CTGTAGTTACGCACGCGTGTCCCACAATTGACCACGGCGGTTTTGTGCCA 5075

  contig10_pilo     345609 TATCCGCAGATTAGATGAACGATTTTTTTTTTCTCTGTAAAGAGTTTTCT 345658
                              v v    vv    v     -viv        v   v           
  rnd-4_family-       5076 TATGCTCAGAAAAGATCAACGA-ACGTTTTTTCTGTGTCAAGAGTTTTCT 5124

  contig10_pilo     345659 ACACATTCTGGTTTCAAAGAAGCAGGCTCGGTCTTTGAGATTATTGCAAA 345708
                               i  iiv i  --   ii                             
  rnd-4_family-       5125 ACACGTTTCTGCTT--AAGGGGCAGGCTCGGTCTTTGAGATTATTGCAAA 5172

  contig10_pilo     345709 A-AGATACCTATCCATCAAGACGAAAACGGATTGATCTTCCTCTTCTTCT 345757
                            - i i                 v  v   i  i       i        
  rnd-4_family-       5173 AGAAACACCTATCCATCAAGACGCAATCGGGTTAATCTTCCCCTTCTTCT 5222

  contig10_pilo     345758 TGGGCGATTCATCGAGCCCGCGCGACGCGGGCC--GTTTGGAGGACGACT 345805
                                           v                --v           ?  
  rnd-4_family-       5223 TGGGCGATTCATCGAGGCCGCGCGACGCGGGCCTTTTTTGGAGGACGNCT 5272

  contig10_pilo     345806 TAGAAGTCGACGGCGATGCGCTCTGAGCCACGGCCCCGCTCCTCTTGACA 345855
                            v  v           i                vi     v         
  rnd-4_family-       5273 TCGACGTCGACGGCGACGCGCTCTGAGCCACGGATCCGCTGCTCTTGACA 5322

  contig10_pilo     345856 CTCGCGGTACCCATGGCTTTCCTGACGCTAGCGCCAGCAGCGGTGACCTT 345905
                                       ii       i v     i                  v 
  rnd-4_family-       5323 CTCGCGGTACCCGCGGCTTTCTTCACGCTGGCGCCAGCAGCGGTGACCGT 5372

  contig10_pilo     345906 CGGCGCACCTTTGCCAGAGCCCTCCGCGCGCTGGGAGGTAGCCTTCCCAT 345955
                                       v  i     v  vv          v  vv     v  v
  rnd-4_family-       5373 CGGCGCACCTTTTCCGGAGCCGTCGCCGCGCTGGGATGTCTCCTTCGCAA 5422

  contig10_pilo     345956 CTCGCGG 345962
                            v  i  
  rnd-4_family-       5423 CGCGTGG 5429

Matrix = 20p53g.matrix
Kimura (with divCpGMod) = 10.63
Transitions / transversions = 1.11 (218/196)
Gap_init rate = 0.01 (37 / 3675), avg. gap size = 1.46 (54 / 37)

 
 

 
   +      13   30.0  1.8  1.8  contig10_pilon   349411  349466 (1503213) +  (CCG)n             Simple_repeat        1     56     (0)    81     
 
 ANNOTATION EVIDENCE: 
    13  30.01 1.79 1.79  contig10_pilon   349411  349466   1503213 +  (CCG)n             Simple_repeat        1     56       0      
13 30.01 1.79 1.79 contig10_pilon 349411 349466 (1503213) (CCG)n#Simple_repeat 1 56 (0) m_b690s252i0

  contig10_pilo     349411 CCGCTGCCG-CGAAGACTGCTGCCACTGCTGACGCTGACACCGCCGCTGC 349459
                               i    -  vv - i  i   i i  i v   i v i       i  
  (CCG)n#Simple          1 CCGCCGCCGCCGCCG-CCGCCGCCGCCGCCGCCGCCGCCGCCGCCGCCGC 49

  contig10_pilo     349460 CGCCGCC 349466
                                  
  (CCG)n#Simple         50 CGCCGCC 56

Matrix = Unknown
Transitions / transversions = 2.25 (9/4)
Gap_init rate = 0.04 (2 / 55), avg. gap size = 1.00 (2 / 2)

 
 
 
 
  +     987   10.0  0.0  0.6  contig10_pilon   349885  350055 (1502624) +  rnd-4_family-158   Unknown           5418   5587  (6180)    80     
 
 ANNOTATION EVIDENCE: 
   987  10.00 0.00 0.59  contig10_pilon   349885  350055   1502624 +  rnd-4_family-158   Unknown           5418   5587    6180      
987 10.00 0.00 0.59 contig10_pilon 349885 350055 (1502624) rnd-4_family-158#Unknown 5418 5587 (6180) m_b690s001i0

  contig10_pilo     349885 CGCATACACGTGGCACAGCGACCGCGTCGAGGTCGACGCGCCCACCCTGC 349934
                               -  i        i   i i              v            
  rnd-4_family-       5418 CGCA-ACGCGTGGCACGGCGGCTGCGTCGAGGTCGACTCGCCCACCCTGC 5466

  contig10_pilo     349935 CCCAAGCAATCCGGGACGTGTGGTTCCGTGAGATGACCGGGGCGCGGCAG 349984
                                           i           v       v   v    vv   
  rnd-4_family-       5467 CCCAAGCAATCCGGGATGTGTGGTTCCGGGAGATGAGCGGCGCGCCCCAG 5516

  contig10_pilo     349985 CAGCAGGCGGCCGAGACGGCAGCCGCAGTCGAGGAGCTTCGTAAGGCCAA 350034
                                           i         i  i                    
  rnd-4_family-       5517 CAGCAGGCGGCCGAGATGGCAGCCGCGGTTGAGGAGCTTCGTAAGGCCAA 5566

  contig10_pilo     350035 GAAGGACGCAGAGGACGCCCT 350055
                                    v      v  i 
  rnd-4_family-       5567 GAAGGACGCCGAGGACCCCTT 5587

Matrix = 20p53g.matrix
Kimura (with divCpGMod) = 8.93
Transitions / transversions = 1.12 (9/8)
Gap_init rate = 0.01 (1 / 170), avg. gap size = 1.00 (1 / 1)

 
 

 
 
   +     634   35.8  4.4  4.7  contig10_pilon   352413  352762 (1499917) C  rnd-4_family-94    Unknown        (13114)   3127    2479    82     
 
 ANNOTATION EVIDENCE: 
   634  35.78 4.41 4.70  contig10_pilon   352413  353500   1499179 C  rnd-4_family-94    Unknown           2043   3127   13114      
634 35.78 4.41 4.70 contig10_pilon 352413 353500 (1499179) C rnd-4_family-94#Unknown (13114) 3127 2043 m_b690s001i1

  contig10_pilo     352413 CGCAGCGTGTCCTACCTGCCCGTCGCACCCCCGGAATGGGCGCGT-CTGC 352461
                             v     i   ii   vvv     vv  v    vi        i-i   
C rnd-4_family-       3127 CGGAGCGTATCCCGCCTCAACGTCGGCCCACCGGCGTGGGCGCGCATTGC 3078

  contig10_pilo     352462 CTC-GA----GATGACATTGCGTC---ATTTGCACTGGTCGATCCTCTCC 352503
                              -  ----  i  viv   ii ---i  i  v  v  i      v   
C rnd-4_family-       3077 CTCAGATTCTGACGAGGATGCACCCACGTTCGCTCTCGTTGATCCTATCC 3028

  contig10_pilo     352504 CAGCCAGGTTTAGGATCGACACAGACGCTCGGTGTGCATGTGGCGGTGTG 352553
                            i v viv  iv vv   i iv   iv    v  ivvv     vi   vv
C rnd-4_family-       3027 CGGACTACTTCTGCCTCGGCGAAGATCCTCGTTGCCGCTGTGGGAGTGGT 2978

  contig10_pilo     352554 GCTTTGGCGCTGGCGCCCGTCCAGGCTAGGAAGTGCATCGTGTAC--ACC 352601
                           v vvv  viii      v   v   ii vvi v        i v --  i
C rnd-4_family-       2977 CCGAAGGAATCGGCGCCAGTCAAGGTCATCGACTGCATCGTATTCGGACT 2928

  contig10_pilo     352602 TCAGTTCGCGCCTACGAGGCCACGATCGAAGTTCAGCGCTGC----TCGT 352647
                           iiv  i    -- i  vv   vvi        i  ivv    ----i vv
C rnd-4_family-       2927 CTCGTCCGCG--TGCGCTGCCCAAATCGAAGTCCAAGTCTGCAGCACCTG 2880

  contig10_pilo     352648 CGTGCCGCAAA-----CGG--TGC-GGACCGGACATGCGGGAGCTCGGGA 352689
                             v   v    ----- i --   -  i  i   v    v   i    v 
C rnd-4_family-       2879 CGAGCCCCAAAGCCATCAGAATGCCGGGCCAGACCTGCGCGAGTTCGGTA 2830

  contig10_pilo     352690 TATTCAACTACAACAACGAGCACCTCTACAGTCACGAGCTGCTCAACTCG 352739
                            v       v   i  ivvv i   v    vv        v   i  vv 
C rnd-4_family-       2829 TCTTCAACTTCAATAATTCCCGCCTGTACACGCACGAGCTTCTCGACAAG 2780

  contig10_pilo     352740 TTCACGAGCAGCATGACGAGCTACGAGGCGCCTTTCCACGCATTTTGCAA 352789
                            v   v   vv       vv vv     iv  i        i  iiii v
C rnd-4_family-       2779 TACACTAGCCTCATGACGCTCGCCGAGGTTCCCTTCCACGCGTTCCATAT 2730

  contig10_pilo     352790 GCACA-CTGTCAATAGCTATATCGAACACCGCTCGCCGGAGGCCTTCGTA 352838
                           iiv i-i    i v -i    i i i------   i iivvv       v
C rnd-4_family-       2729 ATCCGTTTGTCGAGA-TTATACCAAG------TCGTCAATTCCCTTCGTC 2687

  contig10_pilo     352839 TCCGATACCACCCTACGCAAAGTCTGGTTCTCGTTCCTGAGAATCCAGCA 352888
                            v   vvvv  vi v  v v  iv iv         iiv   v     v 
C rnd-4_family-       2686 TGCGAGCGGACGTTCCGAACAGCATATTTCTCGTTCTCCAGACTCCAGAA 2637

  contig10_pilo     352889 ACTCATCAACAGTTTCGCATGCTCCCAGTGCGGAGACAGCCCGGAAGTTG 352938
                           i  viv ivi  i    iv   vv vvv     v  viiv  v  i  i 
C rnd-4_family-       2636 GCTGGACGTTAGCTTCGTCTGCGACATCTGCGGTGAAGAACCCGAGGTCG 2587

  contig10_pilo     352939 TCATTTTCGACGGCGTCACAGCCGGTTTCGACGCTAAACTGAAGTCGGGG 352988
                             i v vi  i     v  v  i  i   iiv  ii vi  iv v   i 
C rnd-4_family-       2586 TCGTGTGTGATGGCGTGACTGCTGGCTTCAGAGCCGACTTGGTGACGGAG 2537

  contig10_pilo     352989 ACTCTCCGGCCGCCGACCACGACTGGAG-ACAC-CGTCAGGGA----CAA 353032
                           v    v  i  v  v  v  v ii iv -i   -     vv  ----  i
C rnd-4_family-       2536 TCTCTGCGACCTCCTACGACCATCGATGCGCACGCGTCATCGATACTCAG 2487

  contig10_pilo     353033 CGTCTTACCGCCGTCGGAAAAGTTAACCCTCGT-TACGGGTTTATTGCGA 353081
                              vvv    vi i   iii  viiv vi v  -v    vvv i ii-  
C rnd-4_family-       2486 CGTGAAACCGATGCCGGGGGAGACGTCGTTGGTGAACGGCGATGTCA-GA 2438

  contig10_pilo     353082 ACCCGAGCGCAGAACGCGGTTCGATGGAGGAAAAAGGTTGGGGCGGGAAA 353131
                            vv  v  v  iv v  i         v vvvi  -    v   i  i -
C rnd-4_family-       2437 AAGCGCGCCCAACAGGCAGTTCGATGGCGCCTGAA-GTTGCGGCAGGGA- 2390

  contig10_pilo     353132 CAAGAGCCCGCTGCCGGCGCAAGGCCGCGACGCCAAGGAGTCCATATTCG 353181
                           -    ---   iv iv  v vi   vi   iv vi        ---i i 
C rnd-4_family-       2389 -AAGA---CGCCTCTCGCTCCGGGCGACGATTCGGAGGAGTCC---CTTG 2347

  contig10_pilo     353182 ATACCATGCTCGTCGACGCGGACGTGGACGAGACCGCAGCTCAGCGGCGC 353231
                           --  v v v -     i  v  ------- i  v  v   ------   i
C rnd-4_family-       2346 --ACGAAGAT-GTCGATGCTGA-------GGGAGCGGAGC------GCGT 2313

  contig10_pilo     353232 -GCGAAGAAGCGTCGC--CAAGATATGGATGAGAAGGACGACGCCATGCA 353278
                           -     v  i  v   --     v --    v i i  ii v      vi
C rnd-4_family-       2312 GGCGAATAAACGACGCAACAAGAGA--GATGCGGAAGATAAGGCCATGAG 2265

  contig10_pilo     353279 GAAGAGCCTCCCCGGGATTGCGAAAGAGTTGAAGAAAGTCAACGTCG-GG 353327
                           i    ivi v  i       i i v  ii viv   i--  iv i  - i
C rnd-4_family-       2264 AAAGAAGTTGCCTGGGATTGTGGACGAACTCGCGAAG--CAGAGCCGTGA 2217

  contig10_pilo     353328 CTGGGCA-AACTGTTCGAGACGCATGTA-CGGTCGACGTGGAGAGAAGTT 353375
                           v vi   - iv      iv iii     -  ii   iv  i    vvviv
C rnd-4_family-       2216 ATTAGCACAGATGTTCGGCATATATGTAGCGACCGATTTGAAGAGCCTCG 2167

  contig10_pilo     353376 ACAGAGCCG--CAAACGAAGCTTTATCTCGAGCTGTTGGAACAGGTGA-- 353421
                               vi   --iv  v   ---   ii vv v  i     i      i--
C rnd-4_family-       2166 ACAGCACCGTCTTAAAGAA---TTACTTGCATCTATTGGAGCAGGTGGGT 2120

  contig10_pilo     353422 -------CTGACAGTGATGATGTTCCGCTCAACATAAGCTGATATCCCAG 353464
                           -------  vv viv i i     viviii    ----- vi   i----
C rnd-4_family-       2119 TCGTTTCCTTTCCAGGGTAATGTTGTCTCTAACA-----TCGTATT---- 2079

  contig10_pilo     353465 CAGCTGCTCGCATATGAGCCAGTCATCCAGTTCGCA 353500
                           i       v         i i  vv v         
C rnd-4_family-       2078 TAGCTGCTGGCATATGAGTCGGTGCTACAGTTCGCA 2043

Matrix = 20p53g.matrix
Kimura (with divCpGMod) = 44.62
Transitions / transversions = 0.87 (173/198)
Gap_init rate = 0.07 (73 / 1087), avg. gap size = 1.36 (99 / 73)

 
 
 
 
  +    2371   20.2  0.0  0.0  contig10_pilon   352763  353267 (1499412) +  rnd-4_family-158   Unknown           5576   6080  (5687)    83     
 
 ANNOTATION EVIDENCE: 
  2371  20.20 0.00 0.00  contig10_pilon   352763  353267   1499412 +  rnd-4_family-158   Unknown           5576   6080    5687      
2371 20.20 0.00 0.00 contig10_pilon 352763 353267 (1499412) rnd-4_family-158#Unknown 5576 6080 (5687) m_b690s001i2

  contig10_pilo     352763 CGAGGCGCCTTTCCACGCATTTTGCAAGCACACTGTCAATAGCTATATCG 352812
                                vv  i        i   i          v v     v i      
  rnd-4_family-       5576 CGAGGACCCCTTCCACGCGTTTCGCAAGCACACGGACAATACCCATATCG 5625

  contig10_pilo     352813 AACACCGCTCGCCGGAGGCCTTCGTATCCGATACCACCCTACGCAAAGTC 352862
                            i i    v v  v   v v     i     i  v  v  i   i     
  rnd-4_family-       5626 AGCGCCGCACCCCCGAGCCGTTCGTGTCCGACACGACGCTGCGCGAAGTC 5675

  contig10_pilo     352863 TGGTTCTCGTTCCTGAGAATCCAGCAACTCATCAACAGTTTCGCATGCTC 352912
                                   i                    i  v           i     
  rnd-4_family-       5676 TGGTTCTCATTCCTGAGAATCCAGCAACTTATAAACAGTTTCGCGTGCTC 5725

  contig10_pilo     352913 CCAGTGCGGAGACAGCCCGGAAGTTGTCATTTTCGACGGCGTCACAGCCG 352962
                           vv i     vv i  i  vv       i           vi    i  v 
  rnd-4_family-       5726 GAAATGCGGCCATAGTCCCCAAGTTGTTATTTTCGACGGAATCACGGCAG 5775

  contig10_pilo     352963 GTTTCGACGCTAAACTGAAGTCGGGGACTCTCCGGCCGCCGACCACGACT 353012
                            v        i        iv v        vv       v  i    i 
  rnd-4_family-       5776 GATTCGACGCCAAACTGAAAACTGGGACTCTGAGGCCGCCCACTACGATT 5825

  contig10_pilo     353013 GGAGACACCGTCAGGGACAACGTCTTACCGCCGTCGGAAAAGTTAACCCT 353062
                                   v       v       viv     vv    i     i  v  
  rnd-4_family-       5826 GGAGACACAGTCAGGGCCAACGTCGCCCCGCCCGCGGAGAAGTTGACGCT 5875

  contig10_pilo     353063 CGTTACGGGTTTATTGCGAACCCGAGCGCAGAACGCGGTTCGATGGAGGA 353112
                              v  i   i      i   v iv           i  v          
  rnd-4_family-       5876 CGTGACAGGTCTATTGCAAACGCACGCGCAGAACGCAGTGCGATGGAGGA 5925

  contig10_pilo     353113 AAAAGGTTGGGGCGGGAAACAAGAGCCCGCTGCCGGCGCAAGGCCGCGAC 353162
                            i     v  v     i        v  vi             v    i 
  rnd-4_family-       5926 AGAAGGTAGGCGCGGGGAACAAGAGGCCCTTGCCGGCGCAAGGGCGCGGC 5975

  contig10_pilo     353163 GCCAAGGAGTCCATATTCGATACCATGCTCGTCGACGCGGACGTGGACGA 353212
                            i v  i  v v  v        ivv v v       v   i      vi
  rnd-4_family-       5976 GTCCAGAAGGCGATCTTCGATACTTGGATGGTCGACGAGGATGTGGACCG 6025

  contig10_pilo     353213 GACCGCAGCTCAGCGGCGCGCGAAGAAGCGTCGCCAAGATATGGATGAGA 353262
                                 i v v          i    i       v     v v  i    
  rnd-4_family-       6026 GACCGCGGGTGAGCGGCGCGCAAAGAGGCGTCGCGAAGATCTCGACGAGA 6075

  contig10_pilo     353263 AGGAC 353267
                                
  rnd-4_family-       6076 AGGAC 6080

Matrix = 20p53g.matrix
Kimura (with divCpGMod) = 21.38
Transitions / transversions = 0.76 (44/58)
Gap_init rate = 0.00 (0 / 504), avg. gap size = 0.0 (0 / 0)

 
 

 
   +     634   35.8  4.4  4.7  contig10_pilon   353268  353500 (1499179) C  rnd-4_family-94    Unknown        (13114)   2478    2043    82     
 
 ANNOTATION EVIDENCE: 
   634  35.78 4.41 4.70  contig10_pilon   352413  353500   1499179 C  rnd-4_family-94    Unknown           2043   3127   13114      
634 35.78 4.41 4.70 contig10_pilon 352413 353500 (1499179) C rnd-4_family-94#Unknown (13114) 3127 2043 m_b690s001i1

  contig10_pilo     352413 CGCAGCGTGTCCTACCTGCCCGTCGCACCCCCGGAATGGGCGCGT-CTGC 352461
                             v     i   ii   vvv     vv  v    vi        i-i   
C rnd-4_family-       3127 CGGAGCGTATCCCGCCTCAACGTCGGCCCACCGGCGTGGGCGCGCATTGC 3078

  contig10_pilo     352462 CTC-GA----GATGACATTGCGTC---ATTTGCACTGGTCGATCCTCTCC 352503
                              -  ----  i  viv   ii ---i  i  v  v  i      v   
C rnd-4_family-       3077 CTCAGATTCTGACGAGGATGCACCCACGTTCGCTCTCGTTGATCCTATCC 3028

  contig10_pilo     352504 CAGCCAGGTTTAGGATCGACACAGACGCTCGGTGTGCATGTGGCGGTGTG 352553
                            i v viv  iv vv   i iv   iv    v  ivvv     vi   vv
C rnd-4_family-       3027 CGGACTACTTCTGCCTCGGCGAAGATCCTCGTTGCCGCTGTGGGAGTGGT 2978

  contig10_pilo     352554 GCTTTGGCGCTGGCGCCCGTCCAGGCTAGGAAGTGCATCGTGTAC--ACC 352601
                           v vvv  viii      v   v   ii vvi v        i v --  i
C rnd-4_family-       2977 CCGAAGGAATCGGCGCCAGTCAAGGTCATCGACTGCATCGTATTCGGACT 2928

  contig10_pilo     352602 TCAGTTCGCGCCTACGAGGCCACGATCGAAGTTCAGCGCTGC----TCGT 352647
                           iiv  i    -- i  vv   vvi        i  ivv    ----i vv
C rnd-4_family-       2927 CTCGTCCGCG--TGCGCTGCCCAAATCGAAGTCCAAGTCTGCAGCACCTG 2880

  contig10_pilo     352648 CGTGCCGCAAA-----CGG--TGC-GGACCGGACATGCGGGAGCTCGGGA 352689
                             v   v    ----- i --   -  i  i   v    v   i    v 
C rnd-4_family-       2879 CGAGCCCCAAAGCCATCAGAATGCCGGGCCAGACCTGCGCGAGTTCGGTA 2830

  contig10_pilo     352690 TATTCAACTACAACAACGAGCACCTCTACAGTCACGAGCTGCTCAACTCG 352739
                            v       v   i  ivvv i   v    vv        v   i  vv 
C rnd-4_family-       2829 TCTTCAACTTCAATAATTCCCGCCTGTACACGCACGAGCTTCTCGACAAG 2780

  contig10_pilo     352740 TTCACGAGCAGCATGACGAGCTACGAGGCGCCTTTCCACGCATTTTGCAA 352789
                            v   v   vv       vv vv     iv  i        i  iiii v
C rnd-4_family-       2779 TACACTAGCCTCATGACGCTCGCCGAGGTTCCCTTCCACGCGTTCCATAT 2730

  contig10_pilo     352790 GCACA-CTGTCAATAGCTATATCGAACACCGCTCGCCGGAGGCCTTCGTA 352838
                           iiv i-i    i v -i    i i i------   i iivvv       v
C rnd-4_family-       2729 ATCCGTTTGTCGAGA-TTATACCAAG------TCGTCAATTCCCTTCGTC 2687

  contig10_pilo     352839 TCCGATACCACCCTACGCAAAGTCTGGTTCTCGTTCCTGAGAATCCAGCA 352888
                            v   vvvv  vi v  v v  iv iv         iiv   v     v 
C rnd-4_family-       2686 TGCGAGCGGACGTTCCGAACAGCATATTTCTCGTTCTCCAGACTCCAGAA 2637

  contig10_pilo     352889 ACTCATCAACAGTTTCGCATGCTCCCAGTGCGGAGACAGCCCGGAAGTTG 352938
                           i  viv ivi  i    iv   vv vvv     v  viiv  v  i  i 
C rnd-4_family-       2636 GCTGGACGTTAGCTTCGTCTGCGACATCTGCGGTGAAGAACCCGAGGTCG 2587

  contig10_pilo     352939 TCATTTTCGACGGCGTCACAGCCGGTTTCGACGCTAAACTGAAGTCGGGG 352988
                             i v vi  i     v  v  i  i   iiv  ii vi  iv v   i 
C rnd-4_family-       2586 TCGTGTGTGATGGCGTGACTGCTGGCTTCAGAGCCGACTTGGTGACGGAG 2537

  contig10_pilo     352989 ACTCTCCGGCCGCCGACCACGACTGGAG-ACAC-CGTCAGGGA----CAA 353032
                           v    v  i  v  v  v  v ii iv -i   -     vv  ----  i
C rnd-4_family-       2536 TCTCTGCGACCTCCTACGACCATCGATGCGCACGCGTCATCGATACTCAG 2487

  contig10_pilo     353033 CGTCTTACCGCCGTCGGAAAAGTTAACCCTCGT-TACGGGTTTATTGCGA 353081
                              vvv    vi i   iii  viiv vi v  -v    vvv i ii-  
C rnd-4_family-       2486 CGTGAAACCGATGCCGGGGGAGACGTCGTTGGTGAACGGCGATGTCA-GA 2438

  contig10_pilo     353082 ACCCGAGCGCAGAACGCGGTTCGATGGAGGAAAAAGGTTGGGGCGGGAAA 353131
                            vv  v  v  iv v  i         v vvvi  -    v   i  i -
C rnd-4_family-       2437 AAGCGCGCCCAACAGGCAGTTCGATGGCGCCTGAA-GTTGCGGCAGGGA- 2390

  contig10_pilo     353132 CAAGAGCCCGCTGCCGGCGCAAGGCCGCGACGCCAAGGAGTCCATATTCG 353181
                           -    ---   iv iv  v vi   vi   iv vi        ---i i 
C rnd-4_family-       2389 -AAGA---CGCCTCTCGCTCCGGGCGACGATTCGGAGGAGTCC---CTTG 2347

  contig10_pilo     353182 ATACCATGCTCGTCGACGCGGACGTGGACGAGACCGCAGCTCAGCGGCGC 353231
                           --  v v v -     i  v  ------- i  v  v   ------   i
C rnd-4_family-       2346 --ACGAAGAT-GTCGATGCTGA-------GGGAGCGGAGC------GCGT 2313

  contig10_pilo     353232 -GCGAAGAAGCGTCGC--CAAGATATGGATGAGAAGGACGACGCCATGCA 353278
                           -     v  i  v   --     v --    v i i  ii v      vi
C rnd-4_family-       2312 GGCGAATAAACGACGCAACAAGAGA--GATGCGGAAGATAAGGCCATGAG 2265

  contig10_pilo     353279 GAAGAGCCTCCCCGGGATTGCGAAAGAGTTGAAGAAAGTCAACGTCG-GG 353327
                           i    ivi v  i       i i v  ii viv   i--  iv i  - i
C rnd-4_family-       2264 AAAGAAGTTGCCTGGGATTGTGGACGAACTCGCGAAG--CAGAGCCGTGA 2217

  contig10_pilo     353328 CTGGGCA-AACTGTTCGAGACGCATGTA-CGGTCGACGTGGAGAGAAGTT 353375
                           v vi   - iv      iv iii     -  ii   iv  i    vvviv
C rnd-4_family-       2216 ATTAGCACAGATGTTCGGCATATATGTAGCGACCGATTTGAAGAGCCTCG 2167

  contig10_pilo     353376 ACAGAGCCG--CAAACGAAGCTTTATCTCGAGCTGTTGGAACAGGTGA-- 353421
                               vi   --iv  v   ---   ii vv v  i     i      i--
C rnd-4_family-       2166 ACAGCACCGTCTTAAAGAA---TTACTTGCATCTATTGGAGCAGGTGGGT 2120

  contig10_pilo     353422 -------CTGACAGTGATGATGTTCCGCTCAACATAAGCTGATATCCCAG 353464
                           -------  vv viv i i     viviii    ----- vi   i----
C rnd-4_family-       2119 TCGTTTCCTTTCCAGGGTAATGTTGTCTCTAACA-----TCGTATT---- 2079

  contig10_pilo     353465 CAGCTGCTCGCATATGAGCCAGTCATCCAGTTCGCA 353500
                           i       v         i i  vv v         
C rnd-4_family-       2078 TAGCTGCTGGCATATGAGTCGGTGCTACAGTTCGCA 2043

Matrix = 20p53g.matrix
Kimura (with divCpGMod) = 44.62
Transitions / transversions = 0.87 (173/198)
Gap_init rate = 0.07 (73 / 1087), avg. gap size = 1.36 (99 / 73)

 
 
 
  +    1247   32.0  1.2  1.2  contig10_pilon   353805  354382 (1498297) C  rnd-4_family-94    Unknown        (14496)   1745    1168    82     
 
 ANNOTATION EVIDENCE: 
  1247  32.05 1.21 1.21  contig10_pilon   353805  354382   1498297 C  rnd-4_family-94    Unknown           1168   1745   14496      
1247 32.05 1.21 1.21 contig10_pilon 353805 354382 (1498297) C rnd-4_family-94#Unknown (14496) 1745 1168 m_b690s001i3

  contig10_pilo     353805 CAGACAGGAACGTTCTATGGGCGACGGCGCGTTCGCGACCGACCGACATA 353854
                                v  v  v     i  i  i vv   i    vi  v  i  iii  
C rnd-4_family-       1745 CAGACTGGCACCTTCTACGGACGGCCCCGCATTCGAAACAGATCGGTGTA 1696

  contig10_pilo     353855 TCCGCGTCTACCGACAGACAAGAAGACGGAGCGCGCGACTGTCAGCGATA 353904
                           i  vviiv v  viv    iivivi  v  v        v    -  iii
C rnd-4_family-       1695 CCCCAACATTCCCGGAGACGGCGCAACCGATCGCGCGACGGTCA-CGGCG 1647

  contig10_pilo     353905 -CGGGCGGGGCGGTCGGCTGCTCGAAGTACTATGAGGCCTACGTGAAGAA 353953
                           -        iv i v vv     v    vi   v  i    i vv     
C rnd-4_family-       1646 TCGGGCGGGAAGATGGCGTGCTCCAAGTTTTATCAGACCTATGGCAAGAA 1597

  contig10_pilo     353954 AGGCATCACCGGCGGACTAATGGCAGTTTGGTGCACGCACAGCGTATGCC 354003
                               v v  v  i  vi       vi i      v       i  v   i
C rnd-4_family-       1596 AGGCTTAACGGGTGGCTTAATGGCCATCTGGTGCCCGCACAGTGTCTGCT 1547

  contig10_pilo     354004 TCGGGTTCCACGTTATACCGCACGGCGAGGGGCGCAACGACGTTTTTTCT 354053
                           v      i   vvi  v   v v           v        i     i
C rnd-4_family-       1546 ACGGGTTTCACTGCATCCCGAAAGGCGAGGGGCGGAACGACGTCTTTTCC 1497

  contig10_pilo     354054 GCTCTGTTCACGCATTGGAAGAAAGCGCCAAAATACGTGATATACGACTT 354103
                             v    v   v  i   ivvviiv i  v   vv   vi          
C rnd-4_family-       1496 GCGCTGTACACTCACTGGGTCCGGCCACCCAAAGTCGTCGTATACGACTT 1447

  contig10_pilo     354104 CGCGTGCGCACTCGCACCTTACTGCATGCTGCGCGAGGCCGAGTTCTTCA 354153
                              v     i  vv v  v         v v  v  iv  ivv v    i
C rnd-4_family-       1446 CGCCTGCGCGCTGTCCCCGTACTGCATGATTCGGGAACCCATCTACTTCG 1397

  contig10_pilo     354154 AGGACACGATTTTCCTCATCGACGAGTTTCACAAGGAGGGCCACTCACGC 354203
                           iv     vv    iv         iv  i  ii  v v i    v i  v
C rnd-4_family-       1396 GCGACACTCTTTTTGTCATCGACGGCTTCCATGAGCACGACCACACGCGG 1347

  contig10_pilo     354204 TGTTCGCCGGCGTGCTTTATCAACAACTACAAGCACT----TCAACTCGC 354249
                             i     v     i  iv   i ivv    v v v ---- v  v-v i
C rnd-4_family-       1346 TGCTCGCCTGCGTGTTTCCTCAGCGCGTACACGGAATGGTGTGAAA-AGT 1298

  contig10_pilo     354250 AGCTCAGACATATCAACTCGAGCGCCGCGGAATGCGGAAACTCGGGGCTT 354299
                            v v  --- i        v     v vv     i  i     vi v  v
C rnd-4_family-       1297 ATCGCA---ACATCAACTCCAGCGCGGGCGAATGTGGGAACTCCAGTCTG 1251

  contig10_pilo     354300 CGAAAGATACGCAAGACACT--GTCATATTTGTCGCAAGCCCACGCAATT 354347
                            vv     v      v i  --  i i  vv i--   vvv    vv  i
C rnd-4_family-       1250 CTCAAGATCCGCAAGTCGCTCAGTTACATGGGC--CAACGGCACGGCATC 1203

  contig10_pilo     354348 CTGTTTGGCTACGCATTTCTCGCCATCTGGAATCG 354382
                           v vi i vv   i i  i  i     i     i  
C rnd-4_family-       1202 GTTCTCGTGTACACGTTCCTTGCCATTTGGAACCG 1168

Matrix = 20p53g.matrix
Kimura (with divCpGMod) = 37.52
Transitions / transversions = 0.71 (76/107)
Gap_init rate = 0.02 (10 / 577), avg. gap size = 1.40 (14 / 10)

 
 

 
 
   +      18   12.2  0.0  0.0  contig10_pilon   354868  354894 (1497785) +  (C)n               Simple_repeat        1     27     (0)    84     
 
 ANNOTATION EVIDENCE: 
    18  12.17 0.00 0.00  contig10_pilon   354868  354894   1497785 +  (C)n               Simple_repeat        1     27       0      
18 12.17 0.00 0.00 contig10_pilon 354868 354894 (1497785) (C)n#Simple_repeat 1 27 (0) m_b690s252i1

  contig10_pilo     354868 CCCCCCCCCCCCCCCCCCAGACCCCCC 354894
                                             vvv      
  (C)n#Simple_r          1 CCCCCCCCCCCCCCCCCCCCCCCCCCC 27

Matrix = Unknown
Transitions / transversions = 0.00 (0/3)
Gap_init rate = 0.00 (0 / 26), avg. gap size = 0.0 (0 / 0)

 
 
 
 
 
  +      14   20.2  0.0  0.0  contig10_pilon   359782  359815 (1492864) +  (CGAGGA)n          Simple_repeat        1     34     (0)    85     
 
 ANNOTATION EVIDENCE: 
    14  20.23 0.00 0.00  contig10_pilon   359782  359815   1492864 +  (CGAGGA)n          Simple_repeat        1     34       0      
14 20.23 0.00 0.00 contig10_pilon 359782 359815 (1492864) (CGAGGA)n#Simple_repeat 1 34 (0) m_b690s252i2

  contig10_pilo     359782 CGAGGACGAGGATGACGAGGAGGGAGAGGATGAG 359815
                                       i  v  v    iv     i   
  (CGAGGA)n#Sim          1 CGAGGACGAGGACGAGGACGAGGACGAGGACGAG 34

Matrix = Unknown
Transitions / transversions = 1.00 (3/3)
Gap_init rate = 0.00 (0 / 33), avg. gap size = 0.0 (0 / 0)

 
 

 
 
   +      13   22.2  4.9  3.2  contig10_pilon   363291  363351 (1489328) +  (CGAGCGC)n         Simple_repeat        1     62     (0)    86     
 
 ANNOTATION EVIDENCE: 
    13  22.17 4.92 3.23  contig10_pilon   363291  363351   1489328 +  (CGAGCGC)n         Simple_repeat        1     62       0      
13 22.17 4.92 3.23 contig10_pilon 363291 363351 (1489328) (CGAGCGC)n#Simple_repeat 1 62 (0) m_b690s252i3

  contig10_pilo     363291 CGACCGCCGATGCGCTGCTTGCGGAGGCGCAACGCGCCG-CCGCCGAGCT 363339
                              v      -    i vvi  v  -    viv      -v        v
  (CGAGCGC)n#Si          1 CGAGCGCCGA-GCGCCGAGCGCCGA-GCGCCGAGCGCCGAGCGCCGAGCG 48

  contig10_pilo     363340 CCG-GCGCC-AGCG 363351
                              -     -    
  (CGAGCGC)n#Si         49 CCGAGCGCCGAGCG 62

Matrix = Unknown
Transitions / transversions = 0.38 (3/8)
Gap_init rate = 0.08 (5 / 60), avg. gap size = 1.00 (5 / 5)

 
 
 
 
 
  +      17   16.0  0.0  0.0  contig10_pilon   366372  366406 (1486273) +  (CAACAC)n          Simple_repeat        1     35     (0)    87     
 
 ANNOTATION EVIDENCE: 
    17  16.01 0.00 0.00  contig10_pilon   366372  366406   1486273 +  (CAACAC)n          Simple_repeat        1     35       0      
17 16.01 0.00 0.00 contig10_pilon 366372 366406 (1486273) (CAACAC)n#Simple_repeat 1 35 (0) m_b690s252i4

  contig10_pilo     366372 CAGCACGAACACAAACACCAACGCCAGCACCAACA 366406
                             i   v     v         i   i        
  (CAACAC)n#Sim          1 CAACACCAACACCAACACCAACACCAACACCAACA 35

Matrix = Unknown
Transitions / transversions = 1.50 (3/2)
Gap_init rate = 0.00 (0 / 34), avg. gap size = 0.0 (0 / 0)

 
 

 
 
   +      16   17.3  2.4  2.4  contig10_pilon   366500  366541 (1486138) +  (GCTCCA)n          Simple_repeat        1     42     (0)    88     
 
 ANNOTATION EVIDENCE: 
    16  17.28 2.38 2.38  contig10_pilon   366500  366541   1486138 +  (GCTCCA)n          Simple_repeat        1     42       0      
16 17.28 2.38 2.38 contig10_pilon 366500 366541 (1486138) (GCTCCA)n#Simple_repeat 1 42 (0) m_b690s252i5

  contig10_pilo     366500 GCTCCGGCTCCAGCTCGGGCTCCAGTTCACA-CTTCAGTTCCA 366541
                                i          vi       i  -  -  i   i    
  (GCTCCA)n#Sim          1 GCTCCAGCTCCAGCTCCAGCTCCAGCTC-CAGCTCCAGCTCCA 42

Matrix = Unknown
Transitions / transversions = 5.00 (5/1)
Gap_init rate = 0.05 (2 / 41), avg. gap size = 1.00 (2 / 2)

 
 
 
 
 
  +      18   28.2  1.4  2.9  contig10_pilon   366699  366767 (1485912) +  (GAC)n             Simple_repeat        1     68     (0)    89     
 
 ANNOTATION EVIDENCE: 
    18  28.22 1.45 2.94  contig10_pilon   366699  366767   1485912 +  (GAC)n             Simple_repeat        1     68       0      
18 28.22 1.45 2.94 contig10_pilon 366699 366767 (1485912) (GAC)n#Simple_repeat 1 68 (0) m_b690s252i6

  contig10_pilo     366699 GATGACGACGCCTGGC-ACTGGCGATGAAAACGACAACGACGCCGATGCC 366747
                             i       v - i -  - i   i  vi     i      v   i v 
  (GAC)n#Simple          1 GACGACGACGAC-GACGAC-GACGACGACGACGACGACGACGACGACGAC 48

  contig10_pilo     366748 GACGGCGAGAACGACGATGA 366767
                               i   vi       i  
  (GAC)n#Simple         49 GACGACGACGACGACGACGA 68

Matrix = Unknown
Transitions / transversions = 2.00 (10/5)
Gap_init rate = 0.04 (3 / 68), avg. gap size = 1.00 (3 / 3)

 
 

 
 
   +      12   19.0  7.7  4.5  contig10_pilon   373821  373885 (1478794) +  (GCGGACGA)n        Simple_repeat        1     67     (0)    90     
 
 ANNOTATION EVIDENCE: 
    12  19.01 7.69 4.48  contig10_pilon   373821  373885   1478794 +  (GCGGACGA)n        Simple_repeat        1     67       0      
12 19.01 7.69 4.48 contig10_pilon 373821 373885 (1478794) (GCGGACGA)n#Simple_repeat 1 67 (0) m_b690s252i7

  contig10_pilo     373821 GCGGGCGAGCGG-CCAGTGGTAC-TG-GGAGGTGAAGAGCAGCTGGACGA 373867
                               i       - v  i  -  -v -   v v vi  vv   -      
  (GCGGACGA)n#S          1 GCGGACGAGCGGACGAGCGG-ACGAGCGGACGAGCGGACGAGC-GGACGA 48

  contig10_pilo     373868 GCAGGACG-GC-GACGAGCG 373885
                             -     -  -        
  (GCGGACGA)n#S         49 GC-GGACGAGCGGACGAGCG 67

Matrix = Unknown
Transitions / transversions = 0.43 (3/7)
Gap_init rate = 0.12 (8 / 64), avg. gap size = 1.00 (8 / 8)

 
 
 
 
 
  +      13   24.8  0.0  0.0  contig10_pilon   383468  383505 (1469174) +  (GCGAACT)n         Simple_repeat        1     38     (0)    91     
 
 ANNOTATION EVIDENCE: 
    13  24.76 0.00 0.00  contig10_pilon   383468  383505   1469174 +  (GCGAACT)n         Simple_repeat        1     38       0      
13 24.76 0.00 0.00 contig10_pilon 383468 383505 (1469174) (GCGAACT)n#Simple_repeat 1 38 (0) m_b690s252i8

  contig10_pilo     383468 GCGGAATGCGAACTGCGTACTGGGACCGGCGAGCAGCG 383505
                              i v           v    v  v v    i v   
  (GCGAACT)n#Si          1 GCGAACTGCGAACTGCGAACTGCGAACTGCGAACTGCG 38

Matrix = Unknown
Transitions / transversions = 0.33 (2/6)
Gap_init rate = 0.00 (0 / 37), avg. gap size = 0.0 (0 / 0)

 
 

 
 
   +      20   18.5  0.0  0.0  contig10_pilon   385616  385658 (1467021) +  (CGCCGT)n          Simple_repeat        1     43     (0)    92     
 
 ANNOTATION EVIDENCE: 
    20  18.47 0.00 0.00  contig10_pilon   385616  385658   1467021 +  (CGCCGT)n          Simple_repeat        1     43       0      
20 18.47 0.00 0.00 contig10_pilon 385616 385658 (1467021) (CGCCGT)n#Simple_repeat 1 43 (0) m_b690s252i9

  contig10_pilo     385616 CGCCGTCGCCGTCGGGCTCGCTGTCGCGCACGCCGTCGCCGTC 385658
                                         vvv    i     vvv             
  (CGCCGT)n#Sim          1 CGCCGTCGCCGTCGCCGTCGCCGTCGCCGTCGCCGTCGCCGTC 43

Matrix = Unknown
Transitions / transversions = 0.17 (1/6)
Gap_init rate = 0.00 (0 / 42), avg. gap size = 0.0 (0 / 0)

 
 
 
 
 
  +      15   24.4  2.0  2.0  contig10_pilon   395755  395804 (1456875) +  (CGA)n             Simple_repeat        1     50     (0)    93     
 
 ANNOTATION EVIDENCE: 
    15  24.42 2.00 2.00  contig10_pilon   395755  395804   1456875 +  (CGA)n             Simple_repeat        1     50       0      
15 24.42 2.00 2.00 contig10_pilon 395755 395804 (1456875) (CGA)n#Simple_repeat 1 50 (0) m_b690s252i10

  contig10_pilo     395755 CGCCGACGACGACACCGACTCCGCCGACGA-GAGCGAGCCCGACGACGGC 395803
                             v          iv    vv  v      -  -   vvv        i 
  (CGA)n#Simple          1 CGACGACGACGACGACGACGACGACGACGACGA-CGACGACGACGACGAC 49

  contig10_pilo     395804 G 395804
                            
  (CGA)n#Simple         50 G 50

Matrix = Unknown
Transitions / transversions = 0.25 (2/8)
Gap_init rate = 0.04 (2 / 49), avg. gap size = 1.00 (2 / 2)

 
 

 
 
   +      11   25.2  2.0  4.0  contig10_pilon   395920  395970 (1456709) +  (CGACGG)n          Simple_repeat        1     50     (0)    94     
 
 ANNOTATION EVIDENCE: 
    11  25.17 1.96 4.00  contig10_pilon   395920  395970   1456709 +  (CGACGG)n          Simple_repeat        1     50       0      
11 25.17 1.96 4.00 contig10_pilon 395920 395970 (1456709) (CGACGG)n#Simple_repeat 1 50 (0) m_b690s252i11

  contig10_pilo     395920 CGACGAGGACGGCGACGGCGAGCCGCTCCCGCCGCCGTCGTC-GCGAGGG 395968
                                iv              - v  -vv  v  v  v  v -    v  
  (CGACGG)n#Sim          1 CGACGGCGACGGCGACGGCGA-CGGC-GACGGCGACGGCGACGGCGACGG 48

  contig10_pilo     395969 CG 395970
                             
  (CGACGG)n#Sim         49 CG 50

Matrix = Unknown
Transitions / transversions = 0.11 (1/9)
Gap_init rate = 0.06 (3 / 50), avg. gap size = 1.00 (3 / 3)

 
 
 
 
 
  +      12   20.4  0.0  7.1  contig10_pilon   399145  399189 (1453490) +  (TCGCGT)n          Simple_repeat        1     42     (0)    95     
 
 ANNOTATION EVIDENCE: 
    12  20.44 0.00 7.14  contig10_pilon   399145  399189   1453490 +  (TCGCGT)n          Simple_repeat        1     42       0      
12 20.44 0.00 7.14 contig10_pilon 399145 399189 (1453490) (TCGCGT)n#Simple_repeat 1 42 (0) m_b690s252i12

  contig10_pilo     399145 TCGCGTTCGCATTCCAGTACGTCGTGCGCAGGACGCGGTTCGCGT 399189
                                     i   vv  v  -   v   - vv   -        
  (TCGCGT)n#Sim          1 TCGCGTTCGCGTTCGCGTTCG-CGTTCGC-GTTCGC-GTTCGCGT 42

Matrix = Unknown
Transitions / transversions = 0.17 (1/6)
Gap_init rate = 0.07 (3 / 44), avg. gap size = 1.00 (3 / 3)

 
 

 
 
   +      14    3.7  6.7  3.2  contig10_pilon   399763  399792 (1452887) +  (CCCCGT)n          Simple_repeat        1     31     (0)    96     
 
 ANNOTATION EVIDENCE: 
    14   3.66 6.67 3.23  contig10_pilon   399763  399792   1452887 +  (CCCCGT)n          Simple_repeat        1     31       0      
14 3.66 6.67 3.23 contig10_pilon 399763 399792 (1452887) (CCCCGT)n#Simple_repeat 1 31 (0) m_b690s252i13

  contig10_pilo     399763 CCCCGTCCCTCGGCCCCGT-CCC-TCCCCGTC 399792
                                    -  v      -   -        
  (CCCCGT)n#Sim          1 CCCCGTCCC-CGTCCCCGTCCCCGTCCCCGTC 31

Matrix = Unknown
Transitions / transversions = 0.00 (0/1)
Gap_init rate = 0.10 (3 / 29), avg. gap size = 1.00 (3 / 3)

 
 
 
 
 
  +      15    0.0  0.0  0.0  contig10_pilon   401664  401680 (1450999) +  (CA)n              Simple_repeat        1     17     (0)    97     
 
 ANNOTATION EVIDENCE: 
    15   0.00 0.00 0.00  contig10_pilon   401664  401680   1450999 +  (CA)n              Simple_repeat        1     17       0      
15 0.00 0.00 0.00 contig10_pilon 401664 401680 (1450999) (CA)n#Simple_repeat 1 17 (0) m_b690s252i14

  contig10_pilo     401664 CACACACACACACACAC 401680
                                            
  (CA)n#Simple_          1 CACACACACACACACAC 17

Matrix = Unknown
Transitions / transversions = 1.00 (0/0)
Gap_init rate = 0.00 (0 / 16), avg. gap size = 0.0 (0 / 0)

 
 

 
 
   +      14   20.7  5.1  3.3  contig10_pilon   406569  406627 (1446052) +  (CCGCGGCG)n        Simple_repeat        1     60     (0)    98     
 
 ANNOTATION EVIDENCE: 
    14  20.69 5.08 3.33  contig10_pilon   406569  406627   1446052 +  (CCGCGGCG)n        Simple_repeat        1     60       0      
14 20.69 5.08 3.33 contig10_pilon 406569 406627 (1446052) (CCGCGGCG)n#Simple_repeat 1 60 (0) m_b691s252i0

  contig10_pilo     406569 CCGCGGTGCC-CGGCGCCGGCGGCGTC-AAGCGCTCGCTG-ACCGCCGCG 406615
                                 i   -       -      i -vi    -   v -i    v   
  (CCGCGGCG)n#S          1 CCGCGGCGCCGCGGCGCC-GCGGCGCCGCGGCGC-CGCGGCGCCGCGGCG 48

  contig10_pilo     406616 CCGCTCGGCCGC 406627
                               vvv     
  (CCGCGGCG)n#S         49 CCGCGGCGCCGC 60

Matrix = Unknown
Transitions / transversions = 0.67 (4/6)
Gap_init rate = 0.09 (5 / 58), avg. gap size = 1.00 (5 / 5)

 
 
 
 
 
  +      12   21.6  3.6  5.6  contig10_pilon   408092  408146 (1444533) +  (TTGCTCG)n         Simple_repeat        1     54     (0)    99     
 
 ANNOTATION EVIDENCE: 
    12  21.59 3.64 5.56  contig10_pilon   408092  408146   1444533 +  (TTGCTCG)n         Simple_repeat        1     54       0      
12 21.59 3.64 5.56 contig10_pilon 408092 408146 (1444533) (TTGCTCG)n#Simple_repeat 1 54 (0) m_b691s252i1

  contig10_pilo     408092 TTGCTCCGTTGTTCGTTGCTTGTTGTCCGATGTCGCGTCTGC-C-TTGCC 408139
                                -     i        i    ii  v  - v   -   - -    i
  (TTGCTCG)n#Si          1 TTGCT-CGTTGCTCGTTGCTCGTTGCTCGTTG-CTCGT-TGCTCGTTGCT 47

  contig10_pilo     408140 TGCTGCT 408146
                           i i    
  (TTGCTCG)n#Si         48 CGTTGCT 54

Matrix = Unknown
Transitions / transversions = 3.50 (7/2)
Gap_init rate = 0.09 (5 / 54), avg. gap size = 1.00 (5 / 5)

 
 

 
 
   +      13   14.9  3.3  0.0  contig10_pilon   409697  409726 (1442953) +  (GCGC)n            Simple_repeat        1     31     (0)   100     
 
 ANNOTATION EVIDENCE: 
    13  14.91 3.33 0.00  contig10_pilon   409697  409726   1442953 +  (GCGC)n            Simple_repeat        1     31       0      
13 14.91 3.33 0.00 contig10_pilon 409697 409726 (1442953) (GCGC)n#Simple_repeat 1 31 (0) m_b691s252i2

  contig10_pilo     409697 GCGGGCTCGCGCGC-CGCGGGCGCTCGCGCG 409726
                              v  v       -    v    v      
  (GCGC)n#Simpl          1 GCGCGCGCGCGCGCGCGCGCGCGCGCGCGCG 31

Matrix = Unknown
Transitions / transversions = 0.00 (0/4)
Gap_init rate = 0.03 (1 / 29), avg. gap size = 1.00 (1 / 1)

 
 
 
 
 
  +      17   15.9  0.0  0.0  contig10_pilon   413639  413673 (1439006) +  (CCTCGG)n          Simple_repeat        1     35     (0)   101     
 
 ANNOTATION EVIDENCE: 
    17  15.89 0.00 0.00  contig10_pilon   413639  413673   1439006 +  (CCTCGG)n          Simple_repeat        1     35       0      
17 15.89 0.00 0.00 contig10_pilon 413639 413673 (1439006) (CCTCGG)n#Simple_repeat 1 35 (0) m_b691s252i3

  contig10_pilo     413639 CCGCGGCCTCGGCGTCAGCCTCGCCCTGGGCCTCG 413673
                             v          v  i      v   v       
  (CCTCGG)n#Sim          1 CCTCGGCCTCGGCCTCGGCCTCGGCCTCGGCCTCG 35

Matrix = Unknown
Transitions / transversions = 0.25 (1/4)
Gap_init rate = 0.00 (0 / 34), avg. gap size = 0.0 (0 / 0)

 
 

 
 
   +     309    8.7  0.0  0.0  contig10_pilon   415482  415527 (1437152) C  rnd-4_family-452   Unknown            (0)    639     594   102     
 
 ANNOTATION EVIDENCE: 
   309   8.70 0.00 0.00  contig10_pilon   415482  415527   1437152 C  rnd-4_family-452   Unknown            594    639       0      
309 8.70 0.00 0.00 contig10_pilon 415482 415527 (1437152) C rnd-4_family-452#Unknown (0) 639 594 m_b691s001i0

  contig10_pilo     415482 TAAGGATGTCAACCGTCCCGTCGACGGGTGACGGCCTTCTCGAGGC 415527
                             i      v                          v       i 
C rnd-4_family-        639 TAGGGATGTAAACCGTCCCGTCGACGGGTGACGGCCGTCTCGAGAC 594

Matrix = 20p53g.matrix
Kimura (with divCpGMod) = 9.26
Transitions / transversions = 1.00 (2/2)
Gap_init rate = 0.00 (0 / 45), avg. gap size = 0.0 (0 / 0)

 
 
 
 
 
  +    3526    1.2  0.0  0.0  contig10_pilon   419821  420248 (1432431) C  rnd-4_family-153   Unknown            (0)    428       1   103     
 
 ANNOTATION EVIDENCE: 
  3526   1.17 0.00 0.00  contig10_pilon   419821  420248   1432431 C  rnd-4_family-153   Unknown              1    428       0      
3526 1.17 0.00 0.00 contig10_pilon 419821 420248 (1432431) C rnd-4_family-153#Unknown (0) 428 1 m_b691s001i1

  contig10_pilo     419821 CACTGCTGGGAACCATGATTTTTCCGAGCTCATGGAATAGGTCAAATTTT 419870
                                                                             
C rnd-4_family-        428 CACTGCTGGGAACCATGATTTTTCCGAGCTCATGGAATAGGTCAAATTTT 379

  contig10_pilo     419871 GCAAGGCTTGGAAAACTCTGTTCAAGATTCCACATTTTTCGAAGCCACAA 419920
                                                                 v           
C rnd-4_family-        378 GCAAGGCTTGGAAAACTCTGTTCAAGATTCCACATTTTGCGAAGCCACAA 329

  contig10_pilo     419921 CCATTTGGGCCCTTACCGATGGCAAAAGTTCAAAATTTCCGAAACTCGGA 419970
                                                             ?               
C rnd-4_family-        328 CCATTTGGGCCCTTACCGATGGCAAAAGTTCAAANTTTCCGAAACTCGGA 279

  contig10_pilo     419971 GAATTGACCCGGATTGTCCGAATCAGGCACAACATTGAATTTTGCGAACC 420020
                                                                             
C rnd-4_family-        278 GAATTGACCCGGATTGTCCGAATCAGGCACAACATTGAATTTTGCGAACC 229

  contig10_pilo     420021 GTAAAAGCCGCCTGAGCCGGAATTTTGCGAACACAAACATCCTGTTATGT 420070
                                                                             
C rnd-4_family-        228 GTAAAAGCCGCCTGAGCCGGAATTTTGCGAACACAAACATCCTGTTATGT 179

  contig10_pilo     420071 CCGTCGCCGGGAAAATCCCGCGGGCTTCTCCGAGTTTCGGAAAATTTGAA 420120
                                                                             
C rnd-4_family-        178 CCGTCGCCGGGAAAATCCCGCGGGCTTCTCCGAGTTTCGGAAAATTTGAA 129

  contig10_pilo     420121 CTTCTGCAATCAGTAAGGGCCCAAATGGTTGTGGCTTCGCAAAATGTGGA 420170
                                  v   i                                      
C rnd-4_family-        128 CTTCTGCCATCGGTAAGGGCCCAAATGGTTGTGGCTTCGCAAAATGTGGA 79

  contig10_pilo     420171 ATTTTGAACAGAGTTTTCCAAGCCTTGCAAAATTTAACCTATTCCATGAG 420220
                             i                                i              
C rnd-4_family-         78 ATCTTGAACAGAGTTTTCCAAGCCTTGCAAAATTTGACCTATTCCATGAG 29

  contig10_pilo     420221 CTCGGAAAAATCATGGTTCCCAGCAGTG 420248
                                                       
C rnd-4_family-         28 CTCGGAAAAATCATGGTTCCCAGCAGTG 1

Matrix = 20p53g.matrix
Kimura (with divCpGMod) = 0.97
Transitions / transversions = 1.50 (3/2)
Gap_init rate = 0.00 (0 / 427), avg. gap size = 0.0 (0 / 0)

 
 

 
 
   +      15    0.0  0.0  0.0  contig10_pilon   428485  428503 (1424176) +  (CGG)n             Simple_repeat        1     19     (0)   104     
 
 ANNOTATION EVIDENCE: 
    15   0.00 0.00 0.00  contig10_pilon   428485  428503   1424176 +  (CGG)n             Simple_repeat        1     19       0      
15 0.00 0.00 0.00 contig10_pilon 428485 428503 (1424176) (CGG)n#Simple_repeat 1 19 (0) m_b691s252i4

  contig10_pilo     428485 CGGCGGCGGCGGCGGCGGC 428503
                                              
  (CGG)n#Simple          1 CGGCGGCGGCGGCGGCGGC 19

Matrix = Unknown
Transitions / transversions = 1.00 (0/0)
Gap_init rate = 0.00 (0 / 18), avg. gap size = 0.0 (0 / 0)

 
 
 
 
 
  +      18    0.0  0.0  0.0  contig10_pilon   429568  429587 (1423092) +  (G)n               Simple_repeat        1     20     (0)   105     
 
 ANNOTATION EVIDENCE: 
    18   0.00 0.00 0.00  contig10_pilon   429568  429587   1423092 +  (G)n               Simple_repeat        1     20       0      
18 0.00 0.00 0.00 contig10_pilon 429568 429587 (1423092) (G)n#Simple_repeat 1 20 (0) m_b691s252i5

  contig10_pilo     429568 GGGGGGGGGGGGGGGGGGGG 429587
                                               
  (G)n#Simple_r          1 GGGGGGGGGGGGGGGGGGGG 20

Matrix = Unknown
Transitions / transversions = 1.00 (0/0)
Gap_init rate = 0.00 (0 / 19), avg. gap size = 0.0 (0 / 0)

 
 

 
 
   +      22   23.1  1.4  2.9  contig10_pilon   430462  430531 (1422148) +  G-rich             Low_complexity       1     69     (0)   106     
 
 ANNOTATION EVIDENCE: 
    22  23.11 1.43 2.90  contig10_pilon   430462  430531   1422148 +  (TCGGGAC)n         Simple_repeat        1     69       0      
22 23.11 1.43 2.90 contig10_pilon 430462 430531 (1422148) (TCGGGAC)n#Simple_repeat 1 69 (0) m_b691s252i6

  contig10_pilo     430462 TCGGGACTTGGGACTGGGTTCTTGGGACCCGGGAAACTTGGGACTTGGGA 430511
                                   i      v  vv  i     i    --   i      i    
  (TCGGGAC)n#Si          1 TCGGGACTCGGGACTCGGGACTCGGGACTCGGG--ACTCGGGACTCGGGA 48

  contig10_pilo     430512 CTGGATATTTGGGAC-CGGGA 430531
                             v iv i i     -     
  (TCGGGAC)n#Si         49 CTCGGGACTCGGGACTCGGGA 69

Matrix = Unknown
Transitions / transversions = 1.60 (8/5)
Gap_init rate = 0.04 (3 / 69), avg. gap size = 1.00 (3 / 3)

 
 
 
 
 
  +    1200   10.4  1.0  0.0  contig10_pilon   430911  431102 (1421577) C  rnd-4_family-2551  Unknown            (0)    726     533   107 *   
 
 ANNOTATION EVIDENCE: 
  1200  10.42 1.04 0.00  contig10_pilon   430911  431102   1421577 C  rnd-4_family-2551  Unknown            533    726       0      
1200 10.42 1.04 0.00 contig10_pilon 430911 431102 (1421577) C rnd-4_family-2551#Unknown (0) 726 533 m_b691s001i2

  contig10_pilo     430911 ATCCTCCTTATACTGTACAAAAA--AATTTATCCAAAATCAAGCCCACAT 430958
                                                ? -- v             i     v   
C rnd-4_family-        726 ATCCTCCTTATACTGTACAAANATTATTTTATCCAAAATCGAGCCCTCAT 677

  contig10_pilo     430959 CCAAATTTCCAAGGTGTTTTCCAGAATCTCACCCCTCCATCCAAGACTTT 431008
                               v   i i            ii           v         i   
C rnd-4_family-        676 CCAATTTTTCGAGGTGTTTTCCAAGATCTCACCCCTGCATCCAAGATTTT 627

  contig10_pilo     431009 GTGAGGGATCCAAGCTGGAGCACTTGTCATCCAAACTGATGACACCGGAT 431058
                                            v     i         i ii    v        
C rnd-4_family-        626 GTGAGGGATCCAAGCTGCAGCACCTGTCATCCAGATCGATGTCACCGGAT 577

  contig10_pilo     431059 CCAAATCCGGTGCCGGCAATCCAAACATGGCACACCCCAGATTC 431102
                                  i                  vv              i 
C rnd-4_family-        576 CCAAATCTGGTGCCGGCAATCCAAACTGGGCACACCCCAGATCC 533

Matrix = 20p53g.matrix
Kimura (with divCpGMod) = 9.69
Transitions / transversions = 1.50 (12/8)
Gap_init rate = 0.01 (1 / 191), avg. gap size = 2.00 (2 / 1)

 
 

 
 
   +    5739    3.7  0.5  0.5  contig10_pilon   431100  431863 (1420816) C  rnd-4_family-2312  Unknown            (0)    764       1   108     
 
 ANNOTATION EVIDENCE: 
  5739   3.68 0.52 0.52  contig10_pilon   431100  431863   1420816 C  rnd-4_family-2312  Unknown              1    764       0      
5739 3.68 0.52 0.52 contig10_pilon 431100 431863 (1420816) C rnd-4_family-2312#Unknown (0) 764 1 m_b691s001i3

  contig10_pilo     431100 TTCCCGCTACCTTGTAGTGGGTCTTTGGCGTGTGATGACCAAATCTGCAA 431149
                                                                             
C rnd-4_family-        764 TTCCCGCTACCTTGTAGTGGGTCTTTGGCGTGTGATGACCAAATCTGCAA 715

  contig10_pilo     431150 TACAACGCGTTTTGGCTCAAGATCTCTCGATTGATGCCCATGTCGATAAA 431199
                                                                   ?         
C rnd-4_family-        714 TACAACGCGTTTTGGCTCAAGATCTCTCGATTGATGCCCANGTCGATAAA 665

  contig10_pilo     431200 TTAGGGTAATCTTCCGCGGATGGTGCCAAACGAGGAGCTCGTCCGCGGGC 431249
                            -                  i      ?                      
C rnd-4_family-        664 T-AGGGTAATCTTCCGCGGACGGTGCCNAACGAGGAGCTCGTCCGCGGGC 616

  contig10_pilo     431250 TGTTTGTTCCGAGGTTGATGCAGCTCCGAAACA-AGCTTGACGCCAGCCC 431298
                            ?                         - iv  -    i           
C rnd-4_family-        615 TNTTTGTTCCGAGGTTGATGCAGCTCC-AGCCACAGCTCGACGCCAGCCC 567

  contig10_pilo     431299 TTGAGCTCAAGCTCATG---AGAGTAATGGCATTATAACTCATTAACCTT 431345
                                    i    ?  ---                    i  ?     ?
C rnd-4_family-        566 TTGAGCTCAGGCTCNTGTGTAGAGTAATGGCATTATAACTTATNAACCTN 517

  contig10_pilo     431346 GTAAAGGTGTTCGTGGCGCAAATGGCGAGGCGCAAAGCGCCTCGCATGAG 431395
                               i              i             i                
C rnd-4_family-        516 GTAAGGGTGTTCGTGGCGCGAATGGCGAGGCGCGAAGCGCCTCGCATGAG 467

  contig10_pilo     431396 CGCCTCAATCATATTTAGGTGCACGAACGGCGCGGCACTGCGTGCCGCGC 431445
                                  i           i                i vvv i       
C rnd-4_family-        466 CGCCTCAGTCATATTTAGGCGCACGAACGGCGCGGCGCGCAGCGCCGCGC 417

  contig10_pilo     431446 GTGAGTGCGCCCATTCGATTTCTTTCACCCGGTACCACAATGTTGAAATG 431495
                                           i       i                         
C rnd-4_family-        416 GTGAGTGCGCCCATTCAATTTCTTCCACCCGGTACCACAATGTTGAAATG 367

  contig10_pilo     431496 TGGGCGACAGACGTGCTGTTTTGGATGTGCAGAGAATCTCATGCTTTAAG 431545
                                                                 - i     -   
C rnd-4_family-        366 TGGGCGACAGACGTGCTGTTTTGGATGTGCAGAGAATC-CGTGCTT-AAG 319

  contig10_pilo     431546 CTTTGGACTGACCAAATCGTTCAGGAGCACAGATTCAGTTCTTAAACAAG 431595
                                                 i                           
C rnd-4_family-        318 CTTTGGACTGACCAAATCGTTCGGGAGCACAGATTCAGTTCTTAAACAAG 269

  contig10_pilo     431596 AAGTTGTCACATGCTGCAGCCGCTCACAGGGCTCATAGCTGTCATTAACT 431645
                                 i                  i                      v 
C rnd-4_family-        268 AAGTTGCCACATGCTGCAGCCGCTCGCAGGGCTCATAGCTGTCATTAAGT 219

  contig10_pilo     431646 CTCATGAAAAGGTAATGAGAACGGTGTGCTCGCTAATATTACAGATACTA 431695
                                 v                                           
C rnd-4_family-        218 CTCATGCAAAGGTAATGAGAACGGTGTGCTCGCTAATATTACAGATACTA 169

  contig10_pilo     431696 GGTACAGCTGCTGGCATTGTCTGGTGAGCGGGTGAGGTGCCGAATTGGCT 431745
                                                           i                 
C rnd-4_family-        168 GGTACAGCTGCTGGCATTGTCTGGTGAGCGGGCGAGGTGCCGAATTGGCT 119

  contig10_pilo     431746 GTCACGAGTCTAAGAAGCAGCATGAGCAGGGGAGAGCCCGTGTCCACACC 431795
                                         v       i               ?           
C rnd-4_family-        118 GTCACGAGTCTAAGCAGCAGCACGAGCAGGGGAGAGCCNGTGTCCACACC 69

  contig10_pilo     431796 TTCTCATAGACTGAATGCATTGGGTCCATGAAGGTAGCGGGTGGTGCCGG 431845
                                      ?   ?          i                       
C rnd-4_family-         68 TTCTCATAGACNGAANGCATTGGGTCTATGAAGGTAGCGGGTGGTGCCGG 19

  contig10_pilo     431846 AACGGCCTATGGGACTTA 431863
                                             
C rnd-4_family-         18 AACGGCCTATGGGACTTA 1

Matrix = 20p53g.matrix
Kimura (with divCpGMod) = 2.33
Transitions / transversions = 3.00 (21/7)
Gap_init rate = 0.01 (6 / 763), avg. gap size = 1.33 (8 / 6)

 
 
 
 
 
  +    3690    8.6  0.4  0.2  contig10_pilon   431863  432396 (1420283) +  rnd-4_family-2551  Unknown            192    726     (0)   109 *   
 
 ANNOTATION EVIDENCE: 
  3690   8.63 0.37 0.19  contig10_pilon   431863  432396   1420283 +  rnd-4_family-2551  Unknown            192    726       0      
3690 8.63 0.37 0.19 contig10_pilon 431863 432396 (1420283) rnd-4_family-2551#Unknown 192 726 (0) m_b691s001i4

  contig10_pilo     431863 ATCCAAGAAATACTTCCGGAGATCCAACTGCAATCCAACCCAATTCCAAG 431912
                                      i               v                      
  rnd-4_family-        192 ATCCAAGAAATGCTTCCGGAGATCCAAGTGCAATCCAACCCAATTCCAAG 241

  contig10_pilo     431913 CATCTCTGAGCTCTGTTATGAGAAAAATCAAAGTCATTATCTATTTATTC 431962
                           i               i            i                    
  rnd-4_family-        242 TATCTCTGAGCTCTGTCATGAGAAAAATCGAAGTCATTATCTATTTATTC 291

  contig10_pilo     431963 AGGGACTGGCAGGGTCGGCCGATTCTCGGGCCCGAAGGTCGGCAGGGTCC 432012
                                     i i                                v    
  rnd-4_family-        292 AGGGACTGGCGGAGTCGGCCGATTCTCGGGCCCGAAGGTCGGCAGTGTCC 341

  contig10_pilo     432013 GGAGGGTCCGGATCCTCAGCCATAGAACTGCTCCGAGGGTCCGACCGACA 432062
                                                     i        i     v v v    
  rnd-4_family-        342 GGAGGGTCCGGATCCTCAGCCATAGAGCTGCTCCGGGGGTCGGTCGGACA 391

  contig10_pilo     432063 CTGCCGACCTTCGGGCCTGAGAATCGGCCGACTCCTCCAGCCCCTGAATA 432112
                                            i                 v    i         
  rnd-4_family-        392 CTGCCGACCTTCGGGCCCGAGAATCGGCCGACTCCGCCAGTCCCTGAATA 441

  contig10_pilo     432113 AATAAATAATGACTTCGATTTTTCTCATGAGACAGCTTA-AAATACTTAG 432161
                                                         v v    i - i      i 
  rnd-4_family-        442 AATAAATAATGACTTCGATTTTTCTCATGACAGAGCTCAGAGATACTTGG 491

  contig10_pilo     432162 AATTGAGTTGGATTGGGCTTGGAACCTCCGGAAGCAATTCTTGGATCTGG 432211
                                i          i     - i       i   v             
  rnd-4_family-        492 AATTGGGTTGGATTGGACTTGG-ATCTCCGGAGGCATTTCTTGGATCTGG 540

  contig10_pilo     432212 GGTGTGCCCAGTTTGGATC-CCGGCATCAGATTTGGATCCGGTGACCTCA 432260
                                             i-      i                   v  i
  rnd-4_family-        541 GGTGTGCCCAGTTTGGATTGCCGGCACCAGATTTGGATCCGGTGACATCG 590

  contig10_pilo     432261 GTCTGGATGACAGATGCTGCCGCTTGGATCCCTCACAAAATATTGGATGG 432310
                           i            i      v                    v       v
  rnd-4_family-        591 ATCTGGATGACAGGTGCTGCAGCTTGGATCCCTCACAAAATCTTGGATGC 640

  contig10_pilo     432311 AGGGGTGAGATCTTGGAAGACACCTCAAAAAACTGAATGGGGGCTTAATT 432360
                                             i       i     i  i   i     ii   
  rnd-4_family-        641 AGGGGTGAGATCTTGGAAAACACCTCGAAAAATTGGATGAGGGCTCGATT 690

  contig10_pilo     432361 TTGGATAAAATAATTATTGTACACCTTAAGGAGGAT 432396
                                         ?v       viv          
  rnd-4_family-        691 TTGGATAAAATAATNTTTGTACAGTATAAGGAGGAT 726

Matrix = 20p53g.matrix
Kimura (with divCpGMod) = 8.07
Transitions / transversions = 1.88 (30/16)
Gap_init rate = 0.01 (3 / 533), avg. gap size = 1.00 (3 / 3)

 
 

 
 
   +     649   26.7  0.5  0.0  contig10_pilon   432439  432625 (1420054) +  rnd-4_family-5053  Unknown              4    191   (227)   110     
 
 ANNOTATION EVIDENCE: 
   649  26.74 0.53 0.00  contig10_pilon   432439  432625   1420054 +  rnd-4_family-5053  Unknown              4    191     227      
649 26.74 0.53 0.00 contig10_pilon 432439 432625 (1420054) rnd-4_family-5053#Unknown 4 191 (227) m_b691s001i5

  contig10_pilo     432439 GGGGGTGTCACAGCTGTAGAAACGATGCCAGAAAATGCAAGTCGAAACTC 432488
                                vi          v      v   i     vv v v          
  rnd-4_family-          4 GGGGGAATCACAGCTGTCGAAACGTTGCTAGAAACGGGACGTCGAAACTC 53

  contig10_pilo     432489 ATCGTGCCGGTCGAAACGTTTCGAGAATCAACAGTGACTGCCCC-GAACC 432537
                           v vivivviiiv     i ivi          v      i    - i v 
  rnd-4_family-         54 TTGAAAGAAACAGAAACATCGTGAGAATCAACTGTGACTACCCCAGGAAC 103

  contig10_pilo     432538 TGCGAGCCAAAACGCACAAATCCGAACTTCAGAAACATACTTTCTTCAAG 432587
                               i   i    v i    v    v  ? i i  iv   v     iv  
  rnd-4_family-        104 TGCGGGCCGAAACTCGCAAAACCGACCTNCGGGAATTTACATTCTTTTAG 153

  contig10_pilo     432588 CAGGGACAGTTTATGGAGGTCCTTGACAGCATGTAATC 432625
                               v  iv  ii v    i     i       v    
  rnd-4_family-        154 CAGGCACGCTTCGTTGAGGCCCTTGGCAGCATGGAATC 191

Matrix = 20p53g.matrix
Kimura (with divCpGMod) = 28.88
Transitions / transversions = 0.92 (24/26)
Gap_init rate = 0.01 (1 / 186), avg. gap size = 1.00 (1 / 1)

 
 
 
 
 
  +    4262   13.1  0.8  1.5  contig10_pilon   432627  433357 (1419322) C  rnd-4_family-2551  Unknown            (0)    726       1   111     
 
 ANNOTATION EVIDENCE: 
  4262  13.06 0.82 1.52  contig10_pilon   432627  433357   1419322 C  rnd-4_family-2551  Unknown              1    726       0      
4262 13.06 0.82 1.52 contig10_pilon 432627 433357 (1419322) C rnd-4_family-2551#Unknown (0) 726 1 m_b691s001i6

  contig10_pilo     432627 ATCCTCCTTATACTGTACAAAAATTATTTTATCCAAAATCAAGCCCTCAT 432676
                                                ?                  i         
C rnd-4_family-        726 ATCCTCCTTATACTGTACAAANATTATTTTATCCAAAATCGAGCCCTCAT 677

  contig10_pilo     432677 CCAAATTTCCAAGGTGTTTTCCAGAATCTCACCCCTCCATCCAAGACTTT 432726
                               v   i i            ii           v         i   
C rnd-4_family-        676 CCAATTTTTCGAGGTGTTTTCCAAGATCTCACCCCTGCATCCAAGATTTT 627

  contig10_pilo     432727 GTGGGGGATCCAAGCTGGAGCACTTGTCATCCAAACTGATGACACCGGAT 432776
                              i             v     i         i ii    v        
C rnd-4_family-        626 GTGAGGGATCCAAGCTGCAGCACCTGTCATCCAGATCGATGTCACCGGAT 577

  contig10_pilo     432777 CCAAATCCGGTGCCGGCAATCCAAACATGGCACACCCCAGATCCAAGAAA 432826
                                  i                  vv                      
C rnd-4_family-        576 CCAAATCTGGTGCCGGCAATCCAAACTGGGCACACCCCAGATCCAAGAAA 527

  contig10_pilo     432827 TACTTCCGGTCATCCAAGTGCAATCCAAACTCATTCCAAATATCTTTGAG 432876
                            i i     vv        v        v iv       i     i    
C rnd-4_family-        526 TGCCTCCGGAGATCCAAGTCCAATCCAACCCAATTCCAAGTATCTCTGAG 477

  contig10_pilo     432877 CCCTG--ATGAGAAAAATCGAAGCCATTATCTATCTACACAGTGAGTGTC 432924
                            i   --                i      i   i  iv   v  v  v 
C rnd-4_family-        476 CTCTGTCATGAGAAAAATCGAAGTCATTATTTATTTATTCAGGGACTGGC 427

  contig10_pilo     432925 GGAGTCTGT-GCTTCTCCGGCCCGAAGGTCGGTCGGACACGGCCGACCTT 432973
                                 v i- v     v              iv viiv  i      ii
C rnd-4_family-        426 GGAGTCGGCCGATTCTCGGGCCCGAAGGTCGGCAGTGTCCGACCGACCCC 377

  contig10_pilo     432974 CGGAGCAGCTCAATAGCTGAGGAAACGGACCCTTCGGACATGCTCCGGAG 433023
                                      v  i        vv        i      ----------
C rnd-4_family-        376 CGGAGCAGCTCTATGGCTGAGGATCCGGACCCTCCGGACA---------- 337

  contig10_pilo     433024 ACGGCCGACCTTCGGGCCCGAGAAGCA-CAGACTCCGACATTCACTGTGT 433072
                           - v                     v i- v       v  v  v   vi 
C rnd-4_family-        336 -CTGCCGACCTTCGGGCCCGAGAATCGGCCGACTCCGCCAGTCCCTGAAT 288

  contig10_pilo     433073 AGATAGATAATGGCTTCGATTTTTCTCAT--CAGGGCTCAAAGATATTTG 433120
                            i          i                --   i     i     i   
C rnd-4_family-        287 AAATAGATAATGACTTCGATTTTTCTCATGACAGAGCTCAGAGATACTTG 238

  contig10_pilo     433121 GAATGAGTTTGGATTGCACTTGGATGACCGGAAGTATTTCTTGGATCTGG 433170
                               vi v                 vv       i               
C rnd-4_family-        237 GAATTGGGTTGGATTGCACTTGGATCTCCGGAAGCATTTCTTGGATCTGG 188

  contig10_pilo     433171 GGTGTGCCATGTTTGGATTGCCGGCACCGGATTTGGATCCGGTGTCATCA 433220
                                   vv                  i               v    i
C rnd-4_family-        187 GGTGTGCCCAGTTTGGATTGCCGGCACCAGATTTGGATCCGGTGACATCG 138

  contig10_pilo     433221 GTTTGGATGACCAGTGCTCCAGCTTGGATCCCTCACAAAGTCTTGGATGG 433270
                           i i        vi     v                    i i       ?
C rnd-4_family-        137 ATCTGGATGACAGGTGCTGCAGCTTGGATCCCTCACAAAATTTTGGATGN 88

  contig10_pilo     433271 AGGGGTGAGATTCTGGAAAACACCTTGGAAATTTGGATGAGGGCTTGATT 433320
                           ?     i     i            i     v             i    
C rnd-4_family-         87 NGGGGTAAGATTTTGGAAAACACCTCGGAAAATTGGATGAGGGCTCGATT 38

  contig10_pilo     433321 TTGAATAAAATAATTTTTGTACAGTATAAGGAGGATA 433357
                              i          v                      
C rnd-4_family-         37 TTGGATAAAATAATATTTGTACAGTATAAGGAGGATA 1

Matrix = 20p53g.matrix
Kimura (with divCpGMod) = 13.41
Transitions / transversions = 1.24 (52/42)
Gap_init rate = 0.02 (15 / 730), avg. gap size = 1.13 (17 / 15)

 
 

 
 
   +     675   15.5 16.0  0.5  contig10_pilon   433360  433474 (1419205) +  rnd-4_family-5053  Unknown            188    315   (103)   112     
 
 ANNOTATION EVIDENCE: 
   675  15.46 15.98 0.51  contig10_pilon   433360  433474   1419205 +  rnd-4_family-5053  Unknown            188    315     103      
675 15.46 15.98 0.51 contig10_pilon 433360 433474 (1419205) rnd-4_family-5053#Unknown 188 315 (103) m_b691s001i7

  contig10_pilo     433360 AATCGTACTTTAAAGTGAGAGAAGCAAAGGGACTGGT-TTTCGGTGAAGG 433408
                                i  i ii   v vi    v         v i -            
  rnd-4_family-        188 AATCGCACCTCGAAGAGTAAGAATCAAAGGGACGGATATTTCGGTGAAGG 237

  contig10_pilo     433409 GAAAGGCGTTGCAGCGGAGCAGTTTCGACGGATCGTTTCGAGCGTTCCGA 433458
                            i       v  v ?v  v             ?                 
  rnd-4_family-        238 GGAAGGCGTAGCTGNCGACCAGTTTCGACGGANCGTTTCGAGCGTTCCGA 287

  contig10_pilo     433459 CTCCCGGAGTCGA------------AAC 433474
                                     v  ------------   
  rnd-4_family-        288 CTCCCGGAGTAGAAACGTCGGGACGAAC 315

Matrix = 20p53g.matrix
Kimura (with divCpGMod) = 15.70
Transitions / transversions = 0.78 (7/9)
Gap_init rate = 0.02 (2 / 114), avg. gap size = 6.50 (13 / 2)

 
 
 
 
  +      69    0.0  0.0  0.0  contig10_pilon   433475  433536 (1419143) +  (TCAAAACGG)n       Simple_repeat        1     62     (0)   113     
 
 ANNOTATION EVIDENCE: 
    69   0.00 0.00 0.00  contig10_pilon   433475  433536   1419143 +  (TCAAAACGG)n       Simple_repeat        1     62       0      
69 0.00 0.00 0.00 contig10_pilon 433475 433536 (1419143) (TCAAAACGG)n#Simple_repeat 1 62 (0) c_b691s251i0

  contig10_pilo     433475 TCAAAACGGTCAAAACGGTCAAAACGGTCAAAACGGTCAAAACGGTCAAA 433524
                                                                             
  (TCAAAACGG)n#          1 TCAAAACGGTCAAAACGGTCAAAACGGTCAAAACGGTCAAAACGGTCAAA 50

  contig10_pilo     433525 ACGGTCAAAACG 433536
                                       
  (TCAAAACGG)n#         51 ACGGTCAAAACG 62

Matrix = Unknown
Transitions / transversions = 1.00 (0/0)
Gap_init rate = 0.00 (0 / 61), avg. gap size = 0.0 (0 / 0)

 
 

 
   +     675   15.5 16.0  0.5  contig10_pilon   433537  433577 (1419102) +  rnd-4_family-5053  Unknown            316    370    (48)   112     
 
 ANNOTATION EVIDENCE: 
   675  15.46 15.98 0.51  contig10_pilon   433537  433577   1419102 +  rnd-4_family-5053  Unknown            316    370      48      
675 15.46 15.98 0.51 contig10_pilon 433537 433577 (1419102) rnd-4_family-5053#Unknown 316 370 (48) m_b691s001i7

  contig10_pilo     433537 TTTCGA--------------CCGTTTCGACCGTTTCGATTGCCCGTTCCG 433572
                            ?    --------------v                 i  v     i v
  rnd-4_family-        316 TNTCGAAAAGGNCGAGGNANACGTTTCGACCGTTTCGACTGACCGTTTCT 365

  contig10_pilo     433573 ACTCG 433577
                             i  
  rnd-4_family-        366 ACCCG 370

Matrix = 20p53g.matrix
Kimura (with divCpGMod) = 15.70
Transitions / transversions = 1.00 (3/3)
Gap_init rate = 0.03 (1 / 40), avg. gap size = 14.00 (14 / 1)

 
 
 
 
  +      48    2.1  0.0  0.0  contig10_pilon   433578  433626 (1419053) +  (CGTTTCGACT)n      Simple_repeat        1     49     (0)   114     
 
 ANNOTATION EVIDENCE: 
    48   2.07 0.00 0.00  contig10_pilon   433578  433626   1419053 +  (CGTTTCGACT)n      Simple_repeat        1     49       0      
48 2.07 0.00 0.00 contig10_pilon 433578 433626 (1419053) (CGTTTCGACT)n#Simple_repeat 1 49 (0) c_b691s251i1

  contig10_pilo     433578 CGTTTCGACGCGTTTCGACTCGTTTCGACTCGTTTCGACTCGTTTCGAC 433626
                                    v                                       
  (CGTTTCGACT)n          1 CGTTTCGACTCGTTTCGACTCGTTTCGACTCGTTTCGACTCGTTTCGAC 49

Matrix = Unknown
Transitions / transversions = 0.00 (0/1)
Gap_init rate = 0.00 (0 / 48), avg. gap size = 0.0 (0 / 0)

 
 

 
   +     675   15.5 16.0  0.5  contig10_pilon   433627  433637 (1419042) +  rnd-4_family-5053  Unknown            371    381    (37)   112     
 
 ANNOTATION EVIDENCE: 
   675  15.46 15.98 0.51  contig10_pilon   433627  433637   1419042 +  rnd-4_family-5053  Unknown            371    381      37      
675 15.46 15.98 0.51 contig10_pilon 433627 433637 (1419042) rnd-4_family-5053#Unknown 371 381 (37) m_b691s001i7

  contig10_pilo     433627 CGCTGTGACAC 433637
                             i vi   i 
  rnd-4_family-        371 CGTTTCGACGC 381

Matrix = 20p53g.matrix
Kimura (with divCpGMod) = 15.70
Transitions / transversions = 3.00 (3/1)
Gap_init rate = 0.00 (0 / 10), avg. gap size = 0.0 (0 / 0)

 
 
 
 
 
  +    1068    3.6  0.0  0.0  contig10_pilon   433650  433789 (1418890) +  rnd-4_family-5053  Unknown              1    140   (278)   115 *   
 
 ANNOTATION EVIDENCE: 
  1068   3.57 0.00 0.00  contig10_pilon   433650  433789   1418890 +  rnd-4_family-5053  Unknown              1    140     278      
1068 3.57 0.00 0.00 contig10_pilon 433650 433789 (1418890) rnd-4_family-5053#Unknown 1 140 (278) m_b691s001i8

  contig10_pilo     433650 ATAGGGGGAATCACAGCTGTCGAAACGTTGCTAGAAACGAGACGTCGAAA 433699
                                                                  i          
  rnd-4_family-          1 ATAGGGGGAATCACAGCTGTCGAAACGTTGCTAGAAACGGGACGTCGAAA 50

  contig10_pilo     433700 CTCTTGAAAGAAACAGAAACATCGTCAGAATCAACTGTGACTACCCCAGG 433749
                                                    v                        
  rnd-4_family-         51 CTCTTGAAAGAAACAGAAACATCGTGAGAATCAACTGTGACTACCCCAGG 100

  contig10_pilo     433750 AACTGCAGGCCGAAACTTGCAAAACCGACCTGCAGGAATT 433789
                                 i          i             ? i      
  rnd-4_family-        101 AACTGCGGGCCGAAACTCGCAAAACCGACCTNCGGGAATT 140

Matrix = 20p53g.matrix
Kimura (with divCpGMod) = 1.67
Transitions / transversions = 4.00 (4/1)
Gap_init rate = 0.00 (0 / 139), avg. gap size = 0.0 (0 / 0)

 
 

 
 
   +    1249   17.0 12.9  0.0  contig10_pilon   433788  433866 (1418813) C  rnd-4_family-5053  Unknown            (0)    418     317   116     
 
 ANNOTATION EVIDENCE: 
  1249  17.01 12.93 0.00  contig10_pilon   433788  433866   1418813 C  rnd-4_family-5053  Unknown            317    418       0      
1249 17.01 12.93 0.00 contig10_pilon 433788 433866 (1418813) C rnd-4_family-5053#Unknown (0) 418 317 m_b691s001i9

  contig10_pilo     433788 TTAGGGGGTGTCACAGCAATCGAAACGAGTCGAAACGCGTCGAAACGCGA 433837
                                   ?i       ii       vv                     i
C rnd-4_family-        418 TTAGGGGGNATCACAGCGGTCGAAACTCGTCGAAACGCGTCGAAACGCGG 369

  contig10_pilo     433838 GTCGGAACGGGCAATCGAAA----CGA-------------------TCGA 433864
                             v i     v  i      ----   -------------------    
C rnd-4_family-        368 GTAGAAACGGTCAGTCGAAACGGTCGAAACGTNTNCCTCGNCCTTTTCGA 319

  contig10_pilo     433865 AA 433866
                           ? 
C rnd-4_family-        318 NA 317

Matrix = 20p53g.matrix
Kimura (with divCpGMod) = 16.96
Transitions / transversions = 1.50 (6/4)
Gap_init rate = 0.03 (2 / 78), avg. gap size = 11.50 (23 / 2)

 
 
 
 
  +      48    0.0  0.0  0.0  contig10_pilon   433867  433910 (1418769) +  (CGTTTTGAC)n       Simple_repeat        1     44     (0)   117     
 
 ANNOTATION EVIDENCE: 
    48   0.00 0.00 0.00  contig10_pilon   433867  433910   1418769 +  (CGTTTTGAC)n       Simple_repeat        1     44       0      
48 0.00 0.00 0.00 contig10_pilon 433867 433910 (1418769) (CGTTTTGAC)n#Simple_repeat 1 44 (0) c_b691s251i2

  contig10_pilo     433867 CGTTTTGACCGTTTTGACCGTTTTGACCGTTTTGACCGTTTTGA 433910
                                                                       
  (CGTTTTGAC)n#          1 CGTTTTGACCGTTTTGACCGTTTTGACCGTTTTGACCGTTTTGA 44

Matrix = Unknown
Transitions / transversions = 1.00 (0/0)
Gap_init rate = 0.00 (0 / 43), avg. gap size = 0.0 (0 / 0)

 
 

 
   +    1249   17.0 12.9  0.0  contig10_pilon   433911  434124 (1418555) C  rnd-4_family-5053  Unknown          (102)    316      88   116     
 
 ANNOTATION EVIDENCE: 
  1249  17.01 12.93 0.00  contig10_pilon   433911  434124   1418555 C  rnd-4_family-5053  Unknown             88    316     102      
1249 17.01 12.93 0.00 contig10_pilon 433911 434124 (1418555) C rnd-4_family-5053#Unknown (102) 316 88 m_b691s001i9

  contig10_pilo     433911 GTT------------TCGACTCCGGGAGTCGGAACACTCGAAACGATCCG 433948
                              ------------  v                 i         ?    
C rnd-4_family-        316 GTTCGTCCCGACGTTTCTACTCCGGGAGTCGGAACGCTCGAAACGNTCCG 267

  contig10_pilo     433949 TCGAAACTGCTCTGCTGCAACGCCTTTCCCTTCACCGAAA-ACCAGTCCC 433997
                                    v  v? v  v       i             - i v     
C rnd-4_family-        266 TCGAAACTGGTCGNCAGCTACGCCTTCCCCTTCACCGAAATATCCGTCCC 217

  contig10_pilo     433998 TTTGCTTCTCTCACTTTAAAGTACGGTTACATGCTGTCAAGGACCTCCAT 434047
                               v    iv v   ii i  i  i  v       i     i    v i
C rnd-4_family-        216 TTTGATTCTTACTCTTCGAGGTGCGATTCCATGCTGCCAAGGGCCTCAAC 167

  contig10_pilo     434048 GAACTGTCCCTGCTTGAAGAAAGTATGTTTCTGAAGTTCGGATTTGTGTG 434097
                              vi  v      vi     v   vi  i i ?  v    v    i v 
C rnd-4_family-        166 GAAGCGTGCCTGCTAAAAGAATGTAAATTCCCGNAGGTCGGTTTTGCGAG 117

  contig10_pilo     434098 TTTCGGCTCGCAGTTTC--GGGCAGTCAC 434124
                                  i       i --   i      
C rnd-4_family-        116 TTTCGGCCCGCAGTTCCTGGGGTAGTCAC 88

Matrix = 20p53g.matrix
Kimura (with divCpGMod) = 16.96
Transitions / transversions = 1.11 (21/19)
Gap_init rate = 0.01 (3 / 213), avg. gap size = 5.00 (15 / 3)

 
 
 
 
 
  +    2580    4.8 10.3  0.0  contig10_pilon   434125  434425 (1418254) +  rnd-4_family-5053  Unknown              2    309   (109)   118     
 
 ANNOTATION EVIDENCE: 
  2580   4.77 10.34 0.00  contig10_pilon   434125  434425   1418254 +  rnd-4_family-5053  Unknown              2    309     109      
2580 4.77 10.34 0.00 contig10_pilon 434125 434425 (1418254) rnd-4_family-5053#Unknown 2 309 (109) m_b691s001i10

  contig10_pilo     434125 TAGGGGGAATCACAGCTGTCGAAACGTTGCTAGAAACGGGACGTCGAAAC 434174
                                                                             
  rnd-4_family-          2 TAGGGGGAATCACAGCTGTCGAAACGTTGCTAGAAACGGGACGTCGAAAC 51

  contig10_pilo     434175 TCTTGAAAGAAACAGAAACATCGTGAGAATCAACTGTGACTACCCCAGGA 434224
                                                                             
  rnd-4_family-         52 TCTTGAAAGAAACAGAAACATCGTGAGAATCAACTGTGACTACCCCAGGA 101

  contig10_pilo     434225 ACTGCAGGCCGAAACTTGCAAAACCGACCTCCGGAAATTTACATTCTTTT 434274
                                i          i             ?   i               
  rnd-4_family-        102 ACTGCGGGCCGAAACTCGCAAAACCGACCTNCGGGAATTTACATTCTTTT 151

  contig10_pilo     434275 AGCTGGCACGCTTTGTTGAGGCCCTTAGTAGCATGGAATCGCACCTCGAA 434324
                              v         i            i i                     
  rnd-4_family-        152 AGCAGGCACGCTTCGTTGAGGCCCTTGGCAGCATGGAATCGCACCTCGAA 201

  contig10_pilo     434325 GAGTAAGAATCAAAGGGATGAATATTTC-GTAAAGGGGAAGGCATAGATG 434373
                                             i i       -  i           i   v  
  rnd-4_family-        202 GAGTAAGAATCAAAGGGACGGATATTTCGGTGAAGGGGAAGGCGTAGCTG 251

  contig10_pilo     434374 TCAACCAGTTTCGACGGAACGTTTCGAGCGTTCCGACTCCCGGAGTAGAA 434423
                           ? i               ?                               
  rnd-4_family-        252 NCGACCAGTTTCGACGGANCGTTTCGAGCGTTCCGACTCCCGGAGTAGAA 301

  contig10_pilo     434424 AC------ 434425
                             ------
  rnd-4_family-        302 ACGTCGGG 309

Matrix = 20p53g.matrix
Kimura (with divCpGMod) = 2.47
Transitions / transversions = 5.50 (11/2)
Gap_init rate = 0.01 (2 / 300), avg. gap size = 3.50 (7 / 2)

 
 

 
   +      31    2.8  0.0  2.6  contig10_pilon   434426  434464 (1418215) +  (TCGAAACGG)n       Simple_repeat        1     38     (0)   119     
 
 ANNOTATION EVIDENCE: 
    31   2.76 0.00 2.63  contig10_pilon   434426  434464   1418215 +  (TCGAAACGG)n       Simple_repeat        1     38       0      
31 2.76 0.00 2.63 contig10_pilon 434426 434464 (1418215) (TCGAAACGG)n#Simple_repeat 1 38 (0) c_b691s251i3

  contig10_pilo     434426 TCGAAACGGTCGAAACGGTCGAAACTTGTCGAAACGGTC 434464
                                                    -v            
  (TCGAAACGG)n#          1 TCGAAACGGTCGAAACGGTCGAAAC-GGTCGAAACGGTC 38

Matrix = Unknown
Transitions / transversions = 0.00 (0/1)
Gap_init rate = 0.03 (1 / 38), avg. gap size = 1.00 (1 / 1)

 
 
 
 
  +    2580    4.8 10.3  0.0  contig10_pilon   434465  434539 (1418140) +  rnd-4_family-5053  Unknown            310    416     (2)   118     
 
 ANNOTATION EVIDENCE: 
  2580   4.77 10.34 0.00  contig10_pilon   434465  434539   1418140 +  rnd-4_family-5053  Unknown            310    416       2      
2580 4.77 10.34 0.00 contig10_pilon 434465 434539 (1418140) rnd-4_family-5053#Unknown 310 416 (2) m_b691s001i10

  contig10_pilo     434465 CGACCGTTTC--------------------------------GACTGACC 434482
                              v   ?  --------------------------------        
  rnd-4_family-        310 CGAACGTNTCGAAAAGGNCGAGGNANACGTTTCGACCGTTTCGACTGACC 359

  contig10_pilo     434483 GTTTCTACCCACGTTTCGATGCGTTTCGACAAGTTTCGACCACTGTGATT 434532
                                     i        i          i          i       ?
  rnd-4_family-        360 GTTTCTACCCGCGTTTCGACGCGTTTCGACGAGTTTCGACCGCTGTGATN 409

  contig10_pilo     434533 CCCCCTA 434539
                                  
  rnd-4_family-        410 CCCCCTA 416

Matrix = 20p53g.matrix
Kimura (with divCpGMod) = 2.47
Transitions / transversions = 4.00 (4/1)
Gap_init rate = 0.01 (1 / 74), avg. gap size = 32.00 (32 / 1)

 
 

 
 
   +      84    0.0  0.0  0.0  contig10_pilon   434634  434724 (1417955) +  (A)n               Simple_repeat        1     91     (0)   120     
 
 ANNOTATION EVIDENCE: 
    84   0.00 0.00 0.00  contig10_pilon   434634  434724   1417955 +  (A)n               Simple_repeat        1     91       0      
84 0.00 0.00 0.00 contig10_pilon 434634 434724 (1417955) (A)n#Simple_repeat 1 91 (0) m_b691s252i7

  contig10_pilo     434634 AAAAAAAAAAAAAAAAAAAAAAAAAAAAAAAAAAAAAAAAAAAAAAAAAA 434683
                                                                             
  (A)n#Simple_r          1 AAAAAAAAAAAAAAAAAAAAAAAAAAAAAAAAAAAAAAAAAAAAAAAAAA 50

  contig10_pilo     434684 AAAAAAAAAAAAAAAAAAAAAAAAAAAAAAAAAAAAAAAAA 434724
                                                                    
  (A)n#Simple_r         51 AAAAAAAAAAAAAAAAAAAAAAAAAAAAAAAAAAAAAAAAA 91

Matrix = Unknown
Transitions / transversions = 1.00 (0/0)
Gap_init rate = 0.00 (0 / 90), avg. gap size = 0.0 (0 / 0)

 
 
 
 
 
  +      13   15.3  2.5  5.1  contig10_pilon   438256  438295 (1414384) +  (TGCTCG)n          Simple_repeat        1     39     (0)   121     
 
 ANNOTATION EVIDENCE: 
    13  15.28 2.50 5.13  contig10_pilon   438256  438295   1414384 +  (TGCTCG)n          Simple_repeat        1     39       0      
13 15.28 2.50 5.13 contig10_pilon 438256 438295 (1414384) (TGCTCG)n#Simple_repeat 1 39 (0) m_b691s252i8

  contig10_pilo     438256 TGCTGGTAGCTTGTGCTCGTGCTCGTATCTGCTGCT-GTGC 438295
                               v  -   i              -v  vv    -    
  (TGCTCG)n#Sim          1 TGCTCGT-GCTCGTGCTCGTGCTCGT-GCTCGTGCTCGTGC 39

Matrix = Unknown
Transitions / transversions = 0.25 (1/4)
Gap_init rate = 0.08 (3 / 39), avg. gap size = 1.00 (3 / 3)

 
 

 
 
   +    2132    1.5  0.0  0.0  contig10_pilon   446044  446304 (1406375) C  rnd-4_family-85    Unknown            (0)    542     282   122     
 
 ANNOTATION EVIDENCE: 
  2132   1.53 0.00 0.00  contig10_pilon   446044  446304   1406375 C  rnd-4_family-85    Unknown            282    542       0      
2132 1.53 0.00 0.00 contig10_pilon 446044 446304 (1406375) C rnd-4_family-85#Unknown (0) 542 282 m_b691s001i11

  contig10_pilo     446044 ATTACCCTTATATTAACCATATGACGAACATTAGGAATTCTGGTTTGTGT 446093
                                                                             
C rnd-4_family-        542 ATTACCCTTATATTAACCATATGACGAACATTAGGAATTCTGGTTTGTGT 493

  contig10_pilo     446094 TCTTAGTCTGCTCTAGGACCGACGCTGGGGCCGCTCTCACTGCCGCTGTC 446143
                                                   v                         
C rnd-4_family-        492 TCTTAGTCTGCTCTAGGACCGACGGTGGGGCCGCTCTCACTGCCGCTGTC 443

  contig10_pilo     446144 TTATATACACAACTGACCCAGCCGGTCCAAGCTCTGACCCATGACCGTCC 446193
                                                                             
C rnd-4_family-        442 TTATATACACAACTGACCCAGCCGGTCCAAGCTCTGACCCATGACCGTCC 393

  contig10_pilo     446194 GCGGCCCAAGCGGTATCGTAGGTCCGTCCGTCTCCGGTGTCTCACAGCTG 446243
                                                                             
C rnd-4_family-        392 GCGGCCCAAGCGGTATCGTAGGTCCGTCCGTCTCCGGTGTCTCACAGCTG 343

  contig10_pilo     446244 CAGTAACTGAGATAAATACTGCTGCCGCAGTGGTGTCCTCGACGTATTTG 446293
                                      i       i                           i  
C rnd-4_family-        342 CAGTAACTGAGGTAAATACCGCTGCCGCAGTGGTGTCCTCGACGTATCTG 293

  contig10_pilo     446294 ACAGCTGTCAG 446304
                            ?         
C rnd-4_family-        292 ANAGCTGTCAG 282

Matrix = 20p53g.matrix
Kimura (with divCpGMod) = 1.20
Transitions / transversions = 3.00 (3/1)
Gap_init rate = 0.00 (0 / 260), avg. gap size = 0.0 (0 / 0)

 
 
 
  +     884    2.6  0.0  0.0  contig10_pilon   446305  446420 (1406259) C  rnd-4_family-85    Unknown          (371)    171      56   122     
 
 ANNOTATION EVIDENCE: 
   884   2.59 0.00 0.00  contig10_pilon   446305  446420   1406259 C  rnd-4_family-85    Unknown             56    171     371      
884 2.59 0.00 0.00 contig10_pilon 446305 446420 (1406259) C rnd-4_family-85#Unknown (371) 171 56 m_b691s001i12

  contig10_pilo     446305 TTAACCAAGTCTCTCCCTCTGCTCTACGCGCCCGAGACGATAGTATTTCC 446354
                             i           v                                   
C rnd-4_family-        171 TTGACCAAGTCTCTACCTCTGCTCTACGCGCCCGAGACGATAGTATTTCC 122

  contig10_pilo     446355 CACCGCCGCCACGCTTGAATAAGCTGTGCTCTGCCGTTCCCTCATACTGC 446404
                                                                           v 
C rnd-4_family-        121 CACCGCCGCCACGCTTGAATAAGCTGTGCTCTGCCGTTCCCTCATACTCC 72

  contig10_pilo     446405 AACGCTGTCTAATCAT 446420
                                ?          
C rnd-4_family-         71 AACGCNGTCTAATCAT 56

Matrix = 20p53g.matrix
Kimura (with divCpGMod) = 2.66
Transitions / transversions = 0.50 (1/2)
Gap_init rate = 0.00 (0 / 115), avg. gap size = 0.0 (0 / 0)

 
 

 
 
   +     243    0.0  0.0  0.0  contig10_pilon   446419  446451 (1406228) C  rnd-4_family-85    Unknown            (0)    542     510   123 *   
 
 ANNOTATION EVIDENCE: 
   243   0.00 0.00 0.00  contig10_pilon   446419  446451   1406228 C  rnd-4_family-85    Unknown            510    542       0      
243 0.00 0.00 0.00 contig10_pilon 446419 446451 (1406228) C rnd-4_family-85#Unknown (0) 542 510 m_b691s001i13

  contig10_pilo     446419 ATTACCCTTATATTAACCATATGACGAACATTA 446451
                                                            
C rnd-4_family-        542 ATTACCCTTATATTAACCATATGACGAACATTA 510

Matrix = 20p53g.matrix
Kimura (with divCpGMod) = 0.00
Transitions / transversions = 1.00 (0/0)
Gap_init rate = 0.00 (0 / 32), avg. gap size = 0.0 (0 / 0)

 
 
 
 
 
  +      12   21.8  2.7  0.0  contig10_pilon   453915  453951 (1398728) +  (GTTCTTG)n         Simple_repeat        1     38     (0)   124     
 
 ANNOTATION EVIDENCE: 
    12  21.81 2.70 0.00  contig10_pilon   453915  453951   1398728 +  (GTTCTTG)n         Simple_repeat        1     38       0      
12 21.81 2.70 0.00 contig10_pilon 453915 453951 (1398728) (GTTCTTG)n#Simple_repeat 1 38 (0) m_b691s252i9

  contig10_pilo     453915 GTTCACGGTT-GTGCTCCTTGGTTCTTTGTTCTTCGTT 453951
                               vi    -v  v i          v      v   
  (GTTCTTG)n#Si          1 GTTCTTGGTTCTTGGTTCTTGGTTCTTGGTTCTTGGTT 38

Matrix = Unknown
Transitions / transversions = 0.40 (2/5)
Gap_init rate = 0.03 (1 / 36), avg. gap size = 1.00 (1 / 1)

 
 

 
 
   +      12   26.0  2.1  2.1  contig10_pilon   455935  455982 (1396697) +  (TCGTCA)n          Simple_repeat        1     48     (0)   125     
 
 ANNOTATION EVIDENCE: 
    12  26.01 2.08 2.08  contig10_pilon   455935  455982   1396697 +  (TCGTCA)n          Simple_repeat        1     48       0      
12 26.01 2.08 2.08 contig10_pilon 455935 455982 (1396697) (TCGTCA)n#Simple_repeat 1 48 (0) m_b691s252i10

  contig10_pilo     455935 TCGTCCTCGTCTCCATCATCATCACTGTCGTCTTC-TAGTTCATCGTCA 455982
                                v     vi i     i   ii   i  v  - v -         
  (TCGTCA)n#Sim          1 TCGTCATCGTCATCGTCATCGTCATCGTCATCGTCATCG-TCATCGTCA 48

Matrix = Unknown
Transitions / transversions = 1.50 (6/4)
Gap_init rate = 0.04 (2 / 47), avg. gap size = 1.00 (2 / 2)

 
 
 
 
 
  +      13   13.6  2.9  2.9  contig10_pilon   458769  458802 (1393877) +  (TCCGCA)n          Simple_repeat        1     34     (0)   126     
 
 ANNOTATION EVIDENCE: 
    13  13.63 2.94 2.94  contig10_pilon   458769  458802   1393877 +  (TCCGCA)n          Simple_repeat        1     34       0      
13 13.63 2.94 2.94 contig10_pilon 458769 458802 (1393877) (TCCGCA)n#Simple_repeat 1 34 (0) m_b691s252i11

  contig10_pilo     458769 TCGGCATTCGCATCCGCATCGCGC-GCCGAATCCG 458802
                             v    i            -   -v   v     
  (TCCGCA)n#Sim          1 TCCGCATCCGCATCCGCATC-CGCATCCGCATCCG 34

Matrix = Unknown
Transitions / transversions = 0.33 (1/3)
Gap_init rate = 0.06 (2 / 33), avg. gap size = 1.00 (2 / 2)

 
 

 
 
   +      17   17.8  2.1  4.3  contig10_pilon   461059  461106 (1391573) +  (TGC)n             Simple_repeat        1     47     (0)   127     
 
 ANNOTATION EVIDENCE: 
    17  17.79 2.08 4.26  contig10_pilon   461059  461106   1391573 +  (TGC)n             Simple_repeat        1     47       0      
17 17.79 2.08 4.26 contig10_pilon 461059 461106 (1391573) (TGC)n#Simple_repeat 1 47 (0) m_b691s252i12

  contig10_pilo     461059 TGCTGTATGACTGCTGCTGTTGTTGCTGTTGCTGCTG-TGGTGGTCCTG 461106
                                -v  -         i  i     i        -  v  v v   
  (TGC)n#Simple          1 TGCTG-CTG-CTGCTGCTGCTGCTGCTGCTGCTGCTGCTGCTGCTGCTG 47

Matrix = Unknown
Transitions / transversions = 0.75 (3/4)
Gap_init rate = 0.06 (3 / 47), avg. gap size = 1.00 (3 / 3)

 
 
 
 
 
  +      15   19.5  4.1  2.0  contig10_pilon   461321  461369 (1391310) +  (CGGCGCG)n         Simple_repeat        1     50     (0)   128     
 
 ANNOTATION EVIDENCE: 
    15  19.52 4.08 2.00  contig10_pilon   461321  461369   1391310 +  (CGGCGCG)n         Simple_repeat        1     50       0      
15 19.52 4.08 2.00 contig10_pilon 461321 461369 (1391310) (CGGCGCG)n#Simple_repeat 1 50 (0) m_b691s252i13

  contig10_pilo     461321 CGGCGCGTGGCGCGCGCCG-GTGGTCGCGCGACGCAGGACGC-CGCCGCG 461368
                                  i        v  - i  -      i   iv i   -  v    
  (CGGCGCG)n#Si          1 CGGCGCGCGGCGCGCGGCGCGCGG-CGCGCGGCGCGCGGCGCGCGGCGCG 49

  contig10_pilo     461369 C 461369
                            
  (CGGCGCG)n#Si         50 C 50

Matrix = Unknown
Transitions / transversions = 1.67 (5/3)
Gap_init rate = 0.06 (3 / 48), avg. gap size = 1.00 (3 / 3)

 
 

 
 
   +      12   16.3  2.8  2.8  contig10_pilon   470909  470944 (1381735) +  (AGCGTC)n          Simple_repeat        1     36     (0)   129     
 
 ANNOTATION EVIDENCE: 
    12  16.31 2.78 2.78  contig10_pilon   470909  470944   1381735 +  (AGCGTC)n          Simple_repeat        1     36       0      
12 16.31 2.78 2.78 contig10_pilon 470909 470944 (1381735) (AGCGTC)n#Simple_repeat 1 36 (0) m_b692s252i0

  contig10_pilo     470909 AGCGTCGAG-GTCGGCATCCTCGTCAGCGTCACCGTC 470944
                                 -  -   i  i  vv           v    
  (AGCGTC)n#Sim          1 AGCGTC-AGCGTCAGCGTCAGCGTCAGCGTCAGCGTC 36

Matrix = Unknown
Transitions / transversions = 0.67 (2/3)
Gap_init rate = 0.06 (2 / 35), avg. gap size = 1.00 (2 / 2)

 
 
 
 
 
  +      14    5.0  0.0  0.0  contig10_pilon   477453  477473 (1375206) +  (TGAA)n            Simple_repeat        1     21     (0)   130     
 
 ANNOTATION EVIDENCE: 
    14   5.00 0.00 0.00  contig10_pilon   477453  477473   1375206 +  (TGAA)n            Simple_repeat        1     21       0      
14 5.00 0.00 0.00 contig10_pilon 477453 477473 (1375206) (TGAA)n#Simple_repeat 1 21 (0) m_b692s252i1

  contig10_pilo     477453 TGAATGAATGAATGAACGAAT 477473
                                           i    
  (TGAA)n#Simpl          1 TGAATGAATGAATGAATGAAT 21

Matrix = Unknown
Transitions / transversions = 1.00 (1/0)
Gap_init rate = 0.00 (0 / 20), avg. gap size = 0.0 (0 / 0)

 
 

 
 
   +      15   17.5  3.8  7.8  contig10_pilon   490085  490137 (1362542) +  (CG)n              Simple_repeat        1     51     (0)   131     
 
 ANNOTATION EVIDENCE: 
    15  17.49 3.77 7.84  contig10_pilon   490085  490137   1362542 +  (CG)n              Simple_repeat        1     51       0      
15 17.49 3.77 7.84 contig10_pilon 490085 490137 (1362542) (CG)n#Simple_repeat 1 51 (0) m_b692s252i2

  contig10_pilo     490085 CGCGCGCGCTGATC-CTCTCGCGCGCG-GAGGGCGTCGCGTCGCGCGGGC 490132
                                    - vv - v v        - v v   -    -      v  
  (CG)n#Simple_          1 CGCGCGCGC-GCGCGCGCGCGCGCGCGCGCGCGCG-CGCG-CGCGCGCGC 47

  contig10_pilo     490133 TGCGC 490137
                           -    
  (CG)n#Simple_         48 -GCGC 51

Matrix = Unknown
Transitions / transversions = 0.00 (0/7)
Gap_init rate = 0.12 (6 / 52), avg. gap size = 1.00 (6 / 6)

 
 
 
 
 
  +      17   27.0  2.9  1.4  contig10_pilon   490206  490273 (1362406) +  (CGCGG)n           Simple_repeat        1     69     (0)   132     
 
 ANNOTATION EVIDENCE: 
    17  26.98 2.94 1.45  contig10_pilon   490206  490273   1362406 +  (CGCGG)n           Simple_repeat        1     69       0      
17 26.98 2.94 1.45 contig10_pilon 490206 490273 (1362406) (CGCGG)n#Simple_repeat 1 69 (0) m_b692s252i3

  contig10_pilo     490206 CGCCGCGATGCGCGACCCGCCGCGGCGCGGCGTCGCG-GACGCCATCGCG 490254
                              v   vv     i v  v            iv   - i  - iv    
  (CGCGG)n#Simp          1 CGCGGCGCGGCGCGGCGCGGCGCGGCGCGGCGCGGCGCGGCG-CGGCGCG 49

  contig10_pilo     490255 GACCTGCACGGCGCGG-GCG 490273
                            vv v  i        -   
  (CGCGG)n#Simp         50 GCGCGGCGCGGCGCGGCGCG 69

Matrix = Unknown
Transitions / transversions = 0.50 (5/10)
Gap_init rate = 0.04 (3 / 67), avg. gap size = 1.00 (3 / 3)

 
 

 
 
   +      16    0.0  0.0  0.0  contig10_pilon   491930  491949 (1360730) +  (CAG)n             Simple_repeat        1     20     (0)   133     
 
 ANNOTATION EVIDENCE: 
    16   0.00 0.00 0.00  contig10_pilon   491930  491949   1360730 +  (CAG)n             Simple_repeat        1     20       0      
16 0.00 0.00 0.00 contig10_pilon 491930 491949 (1360730) (CAG)n#Simple_repeat 1 20 (0) m_b692s252i4

  contig10_pilo     491930 CAGCAGCAGCAGCAGCAGCA 491949
                                               
  (CAG)n#Simple          1 CAGCAGCAGCAGCAGCAGCA 20

Matrix = Unknown
Transitions / transversions = 1.00 (0/0)
Gap_init rate = 0.00 (0 / 19), avg. gap size = 0.0 (0 / 0)

 
 
 
 
 
  +      14   29.6  1.3  4.1  contig10_pilon   498927  499001 (1353678) +  G-rich             Low_complexity       1     73     (0)   134     
 
 ANNOTATION EVIDENCE: 
    14  29.63 1.33 4.11  contig10_pilon   498927  499001   1353678 +  (GGGCGG)n          Simple_repeat        1     73       0      
14 29.63 1.33 4.11 contig10_pilon 498927 499001 (1353678) (GGGCGG)n#Simple_repeat 1 73 (0) m_b692s252i5

  contig10_pilo     498927 GGGCGGGGTTCAGCGGGGCCGGCGGGGGCAGAGGTAAGGGGC-GGGGAGG 498975
                                   -v i v  v vv         - i  vvi     -    v  
  (GGGCGG)n#Sim          1 GGGCGGGG-GCGGGGGCGGGGGCGGGGGC-GGGGGCGGGGGCGGGGGCGG 48

  contig10_pilo     498976 GCGTGGGGGACGGGAGCGACAGCGGG 499001
                            v i     -    i   ivi     
  (GGGCGG)n#Sim         49 GGGCGGGGG-CGGGGGCGGGGGCGGG 73

Matrix = Unknown
Transitions / transversions = 0.70 (7/10)
Gap_init rate = 0.05 (4 / 74), avg. gap size = 1.00 (4 / 4)

 
 

 
 
   +   17155    0.3  0.0  0.0  contig10_pilon   503306  505420 (1347259) +  rnd-4_family-1653  LTR/Gypsy            1   2115     (0)   135     
 
 ANNOTATION EVIDENCE: 
 17155   0.33 0.00 0.00  contig10_pilon   503306  505420   1347259 +  rnd-4_family-1653  LTR/Gypsy            1   2115       0      
17155 0.33 0.00 0.00 contig10_pilon 503306 505420 (1347259) rnd-4_family-1653#LTR/Gypsy 1 2115 (0) m_b692s001i0

  contig10_pilo     503306 TGTTACAGCTCATTCGCTTTTCGCTCCTATCGCCGTTGGGCTTATCGGAC 503355
                                 i                                           
  rnd-4_family-          1 TGTTACGGCTCATTCGCTTTTCGCTCCTATCGCCGTTGGGCTTATCGGAC 50

  contig10_pilo     503356 CTCACTCGCGCCGACGTCATACGGACCGAGTCATCAGACCGAGACCCGCC 503405
                                                                             
  rnd-4_family-         51 CTCACTCGCGCCGACGTCATACGGACCGAGTCATCAGACCGAGACCCGCC 100

  contig10_pilo     503406 GGCGCATGCGTGGCCCCGGCCTGCGGTCAGCGGTCCGGCCGGATCCGGAC 503455
                                                                             
  rnd-4_family-        101 GGCGCATGCGTGGCCCCGGCCTGCGGTCAGCGGTCCGGCCGGATCCGGAC 150

  contig10_pilo     503456 CCCCTTTATATACCGTGTGTTGTCCGCTCTTTGTATGCTAGATTCGAGCT 503505
                                              i               i              
  rnd-4_family-        151 CCCCTTTATATACCGTGTGCTGTCCGCTCTTTGTACGCTAGATTCGAGCT 200

  contig10_pilo     503506 GGTTTGCCCACCGCCGATACATCTCGTCCTTTTTCCCCCACCCGCAGAGC 503555
                                                                             
  rnd-4_family-        201 GGTTTGCCCACCGCCGATACATCTCGTCCTTTTTCCCCCACCCGCAGAGC 250

  contig10_pilo     503556 GTCCGTTCGGCGAGTATCCTCGGTCACCGTAACATAAGCTCGAATCTACC 503605
                                                                             
  rnd-4_family-        251 GTCCGTTCGGCGAGTATCCTCGGTCACCGTAACATAAGCTCGAATCTACC 300

  contig10_pilo     503606 CCTCCCTTACCGACGCTCCGCGTATCCCCCGTTCCTTCCCCCGCCTTCAC 503655
                                                                             
  rnd-4_family-        301 CCTCCCTTACCGACGCTCCGCGTATCCCCCGTTCCTTCCCCCGCCTTCAC 350

  contig10_pilo     503656 CCCGCGCCCTCGGTCAAGCTGTCGCACACCACCCCCGACGTCCAGGCCGA 503705
                                                                             
  rnd-4_family-        351 CCCGCGCCCTCGGTCAAGCTGTCGCACACCACCCCCGACGTCCAGGCCGA 400

  contig10_pilo     503706 CGGCGAAGAACTCGCCTCCCATCTCCAGCGCCTTTCGCTTTCCCCCGACA 503755
                                                                             
  rnd-4_family-        401 CGGCGAAGAACTCGCCTCCCATCTCCAGCGCCTTTCGCTTTCCCCCGACA 450

  contig10_pilo     503756 CCACCGTCAAGATGCCGATGACGACCGAGGAGTTCGCTACCTCCATCGAA 503805
                                                                             
  rnd-4_family-        451 CCACCGTCAAGATGCCGATGACGACCGAGGAGTTCGCTACCTCCATCGAA 500

  contig10_pilo     503806 ACGTCGTTGGTCAACGCCATCAAATCGCTCGGACTCAAACCGGGCAACCC 503855
                                                                             
  rnd-4_family-        501 ACGTCGTTGGTCAACGCCATCAAATCGCTCGGACTCAAACCGGGCAACCC 550

  contig10_pilo     503856 ACCCACCAAAAACTACGTCCAGGCCCCGGAGGATTACGATGGGACCCGCG 503905
                                                                             
  rnd-4_family-        551 ACCCACCAAAAACTACGTCCAGGCCCCGGAGGATTACGATGGGACCCGCG 600

  contig10_pilo     503906 CCAACTACGAGACCTTCCGTCGGACCTTGGAGCTCCACGTCAAAGGAATC 503955
                                                                             
  rnd-4_family-        601 CCAACTACGAGACCTTCCGTCGGACCTTGGAGCTCCACGTCAAAGGAATC 650

  contig10_pilo     503956 CCCGGCGACCGCAACAAGATACTCGCCGCGCTCGGCTTCCTGACCAAAGG 504005
                                                                             
  rnd-4_family-        651 CCCGGCGACCGCAACAAGATACTCGCCGCGCTCGGCTTCCTGACCAAAGG 700

  contig10_pilo     504006 TGACGCCGACGCTTGGGCACAGAACTGGGGTCAGCTCCACGACCTGGACT 504055
                                       i                                     
  rnd-4_family-        701 TGACGCCGACGCCTGGGCACAGAACTGGGGTCAGCTCCACGACCTGGACT 750

  contig10_pilo     504056 CGCCGGACCTCAAGTGGAGCGACTTCCTTCGCGACCTCGACGAGAAGTTC 504105
                                                                             
  rnd-4_family-        751 CGCCGGACCTCAAGTGGAGCGACTTCCTTCGCGACCTCGACGAGAAGTTC 800

  contig10_pilo     504106 CTCGACCCGCGCATCGCCGAGCACGCCCGCGAAGCGATCTCCAAGCTCAC 504155
                                                                             
  rnd-4_family-        801 CTCGACCCGCGCATCGCCGAGCACGCCCGCGAAGCGATCTCCAAGCTCAC 850

  contig10_pilo     504156 TCAAGGCCGAGGCGACGCCGACACCTTCTTCCTCAAGTTCGACGAGCTCC 504205
                                                                             
  rnd-4_family-        851 TCAAGGCCGAGGCGACGCCGACACCTTCTTCCTCAAGTTCGACGAGCTCC 900

  contig10_pilo     504206 GCATCAAGGCCGGATTTACCAACCCGGAGTATCACGACATCGTTCTCGTC 504255
                                                                             
  rnd-4_family-        901 GCATCAAGGCCGGATTTACCAACCCGGAGTATCACGACATCGTTCTCGTC 950

  contig10_pilo     504256 GACTACCTTCGGCGCAACCTCAAGCCGGCGCTCGTCCTCGCCGTGATGCA 504305
                                                                             
  rnd-4_family-        951 GACTACCTTCGGCGCAACCTCAAGCCGGCGCTCGTCCTCGCCGTGATGCA 1000

  contig10_pilo     504306 GTCGCACGAAATCTTCCGGACCACCAGTCTCGCCACGGTCGAAGGACTTC 504355
                                                                             
  rnd-4_family-       1001 GTCGCACGAAATCTTCCGGACCACCAGTCTCGCCACGGTCGAAGGACTTC 1050

  contig10_pilo     504356 GCAAGGCCGAGGTCGCCACCGAGGACAAGCTCGTCAAGATGGAGGCCGAC 504405
                                                                             
  rnd-4_family-       1051 GCAAGGCCGAGGTCGCCACCGAGGACAAGCTCGTCAAGATGGAGGCCGAC 1100

  contig10_pilo     504406 GCCGAGACACCGCTCGGATACTTTCAATTCCGCAAGTACGCCCTTGAGCA 504455
                                  v                                          
  rnd-4_family-       1101 GCCGAGAAACCGCTCGGATACTTTCAATTCCGCAAGTACGCCCTTGAGCA 1150

  contig10_pilo     504456 GGACCCGATTATCCGCCGCCACGGTGACCACGCCCCGGCCACCAACTCTA 504505
                                                                             
  rnd-4_family-       1151 GGACCCGATTATCCGCCGCCACGGTGACCACGCCCCGGCCACCAACTCTA 1200

  contig10_pilo     504506 CGCCGCGCCCGGCGGCCAAGCCCTTCGTTCACCATTACACCGAGAGGGTG 504555
                                                                             
  rnd-4_family-       1201 CGCCGCGCCCGGCGGCCAAGCCCTTCGTTCACCATTACACCGAGAGGGTG 1250

  contig10_pilo     504556 ACTCAACCGGTGATTGTACCTGCCGCGGCGCCCGCTCCGGCGATCACCGC 504605
                                                                             
  rnd-4_family-       1251 ACTCAACCGGTGATTGTACCTGCCGCGGCGCCCGCTCCGGCGATCACCGC 1300

  contig10_pilo     504606 CCCAGCCACCACCGGTCCTGCGCACGACCCGGACCCCATGGACGTGGACC 504655
                              v                                              
  rnd-4_family-       1301 CCCCGCCACCACCGGTCCTGCGCACGACCCGGACCCCATGGACGTGGACC 1350

  contig10_pilo     504656 GTCACCGCGCTCGGACCCTAGGACTCTGCTACCGCTGCAAGAAACCCGGC 504705
                                                                             
  rnd-4_family-       1351 GTCACCGCGCTCGGACCCTAGGACTCTGCTACCGCTGCAAGAAACCCGGC 1400

  contig10_pilo     504706 CATCTCGCTCGGGACTGCCAGGAGAGGAACTTCAAGGACGTCATCCGAGG 504755
                                                                             
  rnd-4_family-       1401 CATCTCGCTCGGGACTGCCAGGAGAGGAACTTCAAGGACGTCATCCGAGG 1450

  contig10_pilo     504756 CCTTTCCACCGAGGACATGGAGGAGATCGCTCGGATGGTCGAAGGAAAAT 504805
                                                                             
  rnd-4_family-       1451 CCTTTCCACCGAGGACATGGAGGAGATCGCTCGGATGGTCGAAGGAAAAT 1500

  contig10_pilo     504806 CCACGCTCCTGGAGGAACACGCCGAGGAGACCGAGGACAAGGATTTTTCC 504855
                                                                             
  rnd-4_family-       1501 CCACGCTCCTGGAGGAACACGCCGAGGAGACCGAGGACAAGGATTTTTCC 1550

  contig10_pilo     504856 GCCCCGCAGTGATTTCCGCGCGATCCCTGTGGGAAGGCGACGGTGTGCCC 504905
                                                                             
  rnd-4_family-       1551 GCCCCGCAGTGATTTCCGCGCGATCCCTGTGGGAAGGCGACGGTGTGCCC 1600

  contig10_pilo     504906 TCCGTACCGAATACAGATGAACGTCTTGTCGATACGCACACCGAGCGTGA 504955
                                                                             
  rnd-4_family-       1601 TCCGTACCGAATACAGATGAACGTCTTGTCGATACGCACACCGAGCGTGA 1650

  contig10_pilo     504956 ACACCTAAGACTCGCCGAGGTCCGACCCTCGGCCCTAAGGCAAATGAACA 505005
                                                                             
  rnd-4_family-       1651 ACACCTAAGACTCGCCGAGGTCCGACCCTCGGCCCTAAGGCAAATGAACA 1700

  contig10_pilo     505006 TACCTCTCACCCTCACCTCCCTCGACACCGGAGAACGGATCCCGGCCAAA 505055
                                                                            i
  rnd-4_family-       1701 TACCTCTCACCCTCACCTCCCTCGACACCGGAGAACGGATCCCGGCCAAG 1750

  contig10_pilo     505056 GCTCTTTTGGATAGTGGATGCACGACCTCAAGTGTGGACCAGGCGTTTGT 505105
                                                                             
  rnd-4_family-       1751 GCTCTTTTGGATAGTGGATGCACGACCTCAAGTGTGGACCAGGCGTTTGT 1800

  contig10_pilo     505106 CAAACGCCGGCAGCTTACAACCCAAGCCGCAGCCGTCGTTCGGACCGTCT 505155
                                                                             
  rnd-4_family-       1801 CAAACGCCGGCAGCTTACAACCCAAGCCGCAGCCGTCGTTCGGACCGTCT 1850

  contig10_pilo     505156 ACAACGCCGACGGCTCGGTCAACGGATTTATCAAGGAGTACGTCGAACTC 505205
                                                                             
  rnd-4_family-       1851 ACAACGCCGACGGCTCGGTCAACGGATTTATCAAGGAGTACGTCGAACTC 1900

  contig10_pilo     505206 CTTGTGGTCGTGCGCGACGCACGCGGCCGCGAACACCGCGAACGGCGCGA 505255
                                                                             
  rnd-4_family-       1901 CTTGTGGTCGTGCGCGACGCACGCGGCCGCGAACACCGCGAACGGCGCGA 1950

  contig10_pilo     505256 CTTTCCGGTAGCCAACCTTGGTGGAAAACACGACCTCTTCCTAGGATACG 505305
                                    ?                                        
  rnd-4_family-       1951 CTTTCCGGTNGCCAACCTTGGTGGAAAACACGACCTCTTCCTAGGATACG 2000

  contig10_pilo     505306 ACTGGTTGGTGGAGCACAACCCGGAAATTGACTGGCCGAAGGGGGCGCTC 505355
                                                                             
  rnd-4_family-       2001 ACTGGTTGGTGGAGCACAACCCGGAAATTGACTGGCCGAAGGGGGCGCTC 2050

  contig10_pilo     505356 ACGTTCACCCGCTGCCCGCCTCGCTGTGGTATGCCGAACCCGGCGCCGAG 505405
                                                                             
  rnd-4_family-       2051 ACGTTCACCCGCTGCCCGCCTCGCTGTGGTATGCCGAACCCGGCGCCGAG 2100

  contig10_pilo     505406 CTCGGCCCGCTACCT 505420
                                          
  rnd-4_family-       2101 CTCGGCCCGCTACCT 2115

Matrix = 20p53g.matrix
Kimura (with divCpGMod) = 0.25
Transitions / transversions = 2.50 (5/2)
Gap_init rate = 0.00 (0 / 2114), avg. gap size = 0.0 (0 / 0)

 
 
 
 
 
  +     388   28.5  1.2  5.0  contig10_pilon   505597  505762 (1346917) +  rnd-4_family-119   LTR/Gypsy         2985   3144     (1)   136     
 
 ANNOTATION EVIDENCE: 
   388  28.54 1.20 5.00  contig10_pilon   505597  505762   1346917 +  rnd-4_family-119   LTR/Gypsy         2985   3144       1      
388 28.54 1.20 5.00 contig10_pilon 505597 505762 (1346917) rnd-4_family-119#LTR/Gypsy 2985 3144 (1) m_b692s001i1

  contig10_pilo     505597 AATTCGACAAGCTACCCCCCCATCGTCGGTGGGATCACGAAATCAACCTC 505646
                            i      i       i  v ii  i v      i    vi   i v   
  rnd-4_family-       2985 AGTTCGACGAGCTACCTCCGCGCCGCCCGTGGGACCACGCGATCGAGCTC 3034

  contig10_pilo     505647 CGGCCCGGATGGGAGAACGACCGCAAGCTCCGCGGA--AAGGTTTACGCC 505694
                             v    - i v -  i vv   v------      -- i  i   iv v
  rnd-4_family-       3035 CGCCCCG-ACGCG-GAGCTCCCGG------CGCGGACGAGGGCTTATCCG 3076

  contig10_pilo     505695 TTGTCGCCGAAGGAGACACAGGCGATGAACGAGTTCTTGGACGAGAACCT 505744
                           i        ivi   vviv   viv  i   v    i    v     i  
  rnd-4_family-       3077 CTGTCGCCGGCAGAGCAGAAGGAACTGGACGCGTTCCTGGAGGAGAATCT 3126

  contig10_pilo     505745 CGCGACGGGTCGTATTCG 505762
                            vv   v        i  
  rnd-4_family-       3127 CCGGACTGGTCGTATCCG 3144

Matrix = 20p53g.matrix
Kimura (with divCpGMod) = 31.53
Transitions / transversions = 1.05 (23/22)
Gap_init rate = 0.05 (9 / 165), avg. gap size = 1.11 (10 / 9)

 
 

 
 
   +     426   36.3  2.7  3.2  contig10_pilon   505788  506408 (1346271) +  rnd-3_family-208   LTR/Gypsy         3548   4165  (4466)   137     
 
 ANNOTATION EVIDENCE: 
   426  36.28 2.74 3.24  contig10_pilon   505788  506408   1346271 +  rnd-3_family-208   LTR/Gypsy         3548   4165    4466      
426 36.28 2.74 3.24 contig10_pilon 505788 506408 (1346271) rnd-3_family-208#LTR/Gypsy 3548 4165 (4466) m_b692s001i2

  contig10_pilo     505788 CCGCTTTTCTTCGTCGGCAAGAAGGATGGAGGACTCCGACCGACTATG-- 505835
                              v  i        ---       vi - v ii  ivvi  v     --
  rnd-3_family-       3548 CCGGTTCTCTTCGTC---AAGAAGGCCG-ACGGTTCTCTTCGCCTATGCG 3593

  contig10_pilo     505836 --GATTACAGGAGGCTCAACGACCACACCGTGCGGGACTCCTACCCCCTC 505883
                           --     iv vi        i vvv   ivv vi    vvv     v   
  rnd-3_family-       3594 TCGATTATCGTGGGCTCAACAAGATCACTCGGAAGGACAAGTACCCGCTC 3643

  contig10_pilo     505884 CCGCTGGTC--GCCGACGTCATGACCAAGATCAAGGAC-GCCCGTTATTT 505930
                                vi  -- i v   - vi    ivvv  -- v  -   vivv v  
  rnd-3_family-       3644 CCGCTCATCTCGTCCACG-CTCGACCGCCTTC--GCACCGCCAAGAAGTT 3690

  contig10_pilo     505931 CTCTAAGTTCGACGTCCGATGGGGGTACAACAACGTCCGAATC-CGACCG 505979
                            v i   i    iv i  vvvv  i    i      i  v   -  i   
  rnd-3_family-       3691 CACCAAGCTCGATCTTCGTGTCGGATACAGCAACGTTCGCATCGCGGCCG 3740

  contig10_pilo     505980 GGAGACGAATTCAAGGCCGCGTTCATCACGAGCCGAGGTCTGTTCGAACC 506029
                            v-     i vv   i i  v  ivvv   v  iiv  iviv v   iiv
  rnd-3_family-       3741 GC-GACGAGTGGAAGACTGCCTTTCGAACGCGCTACGGCACCTACGAGTA 3789

  contig10_pilo     506030 CACCGTCATGTTCTTCGGCATGACCAACAGCCCGGCCACCTTCCAACGCA 506079
                           ivi       iii     iv v  v   ivi  i   i i     i   v
  rnd-3_family-       3790 TCTCGTCATGCCTTTCGGTCTCACGAACGCTCCAGCCGCTTTCCAGCGCT 3839

  contig10_pilo     506080 TGGTGGACGACATATTCGGCGACCTTATTCGGCGGATCGTCCTTATCGTC 506129
                            vi  i       v   vv      i-i   i  --  iivv i i    
  rnd-3_family-       3840 TCATGAACGACATCTTCTCCGACCTC-CTCGACG--TCACGGTCACCGTC 3886

  contig10_pilo     506130 TATATGGACGACCTTTTGGTTTTTACCCGCAC-----CTTGGAGGACCAT 506174
                             iv v      v ii --iv i   i  v  -----  i i  v iv -
  rnd-3_family-       3887 TACCTCGACGACATCCT--CATCTACTCGGACGATCCCTCGAAGCATGA- 3933

  contig10_pilo     506175 CGGACCGCGGTTCGGGCCGTACTGGAACTGTGTCGGCAGACAGGCCTATA 506224
                           -   v i  -    i vv  v  vvi  v ivv   v   vv i   v v
  rnd-3_family-       3934 -GGAACACG-TTCGAGAGGTCCTTCGACGGCTGCGGGAGAATGACCTCTT 3981

  contig10_pilo     506225 CCTCAAACTGGAGAAATGCGAGTTCGA-GCAAGCCCAAGTCAAGTTCCTC 506273
                            ivi  i iv     i         v -  vii v v -  i   v    
  rnd-3_family-       3982 CTGTAAGCCCGAGAAGTGCGAGTTCCATGCTGACACCA-TCGAGTACCTC 4030

  contig10_pilo     506274 GGCCTGGTTGTAGGAAACGGGCAGGTATC--CATGGACCCAGTCAAGGTG 506321
                             vivvi ii vi vvv  i --  i   --         iivv   i v
  rnd-3_family-       4031 GGGTACATCATCAGCCCCGAG--GGCATCAGCATGGACCCGAAGAAGATT 4078

  contig10_pilo     506322 GAGGCCATACGCCGTTGGAACCCCCCGCGCAACCTGCGGGAGGTCCGGTC 506371
                            vvi  v v v viv   vv vii  v      v v  v  vi   i   
  rnd-3_family-       4079 GCCACCCTCCTCGAGTGGCCCGTTCCTCGCAACGTTCGCGACATCCAGTC 4128

  contig10_pilo     506372 GTTCATGCAGTTCTGT-AACTTCTACCGGAACTTCATC 506408
                               v - i i ii i-        i  iviv      
  rnd-3_family-       4129 GTTCCT-CGGCTTCGCGAACTTCTATCGACGATTCATC 4165

Matrix = 20p53g.matrix
Kimura (with divCpGMod) = 45.89
Transitions / transversions = 0.86 (101/117)
Gap_init rate = 0.05 (29 / 620), avg. gap size = 1.28 (37 / 29)

 
 
 
  +     450   28.9  4.8  2.5  contig10_pilon   506571  506840 (1345839) +  rnd-3_family-208   LTR/Gypsy         4328   4603  (4028)   137     
 
 ANNOTATION EVIDENCE: 
   450  28.88 4.81 2.54  contig10_pilon   506571  506840   1345839 +  rnd-3_family-208   LTR/Gypsy         4328   4603    4028      
450 28.88 4.81 2.54 contig10_pilon 506571 506840 (1345839) rnd-3_family-208#LTR/Gypsy 4328 4603 (4028) m_b692s001i3

  contig10_pilo     506571 GAGACCGACGCATCAGACTACGCGGCAGGAGGAGTCCTCCACCAGGTCAT 506620
                                i     v  v         iiv vi vii  i  ivv  v   v 
  rnd-3_family-       4328 GAGACTGACGCTTCCGACTACGCGATCGCGGCGATCTTCTCGCACGTCCT 4377

  contig10_pilo     506621 CGACGGGAAGCTACGCCCAC----TCGGGTTCT-TTAGCAAGAGCTTCAG 506665
                            v-   ivi   vi ii   ----   vv   i-  v  iviv   -  v
  rnd-3_family-       4378 CC-CGGACGGCTCTGTTCACCCCGTCGCTTTCCATTCGCGCACGCT-CAC 4425

  contig10_pilo     506666 TCCCGCCGAGCGTAATTACGAGATCTACGACAAGGAGATGCTCGCGATGA 506715
                               i      vi  i     v ivi           v i        - 
  rnd-3_family-       4426 TCCCACCGAGCTCAACTACGACACGCACGACAAGGAGCTACTCGCGAT-A 4474

  contig10_pilo     506716 TGCTTTGC-CTCGAACATTGGCGCCACTTCCTCAAAGGGGCC----CCGC 506760
                            v vvv  -i      vv     v    v    i        ----  v 
  rnd-3_family-       4475 TTCGAGGCGTTCGAACTATGGCGACACTACCTCGAAGGGGCCGAGACCCC 4524

  contig10_pilo     506761 AGTTCCAGATATGG--ACCGACCATCGGAATCTGCAGTACTTCCGCGAGC 506808
                            i ---   ii v --  v     ivi   i  vv    i   i- v v 
  rnd-3_family-       4525 AAT---AGACGTCGTCACGGACCACAAGAACCTCGAGTATTTCT-CTACC 4570

  contig10_pilo     506809 -CGCAAAAGCTCAACCGTCGGCAAGCTCGTTGG 506840
                           -  v i v     vi              i   
  rnd-3_family-       4571 ACGAAGATGCTCACTCGTCGGCAAGCTCGCTGG 4603

Matrix = 20p53g.matrix
Kimura (with divCpGMod) = 32.71
Transitions / transversions = 0.85 (35/41)
Gap_init rate = 0.05 (13 / 269), avg. gap size = 1.54 (20 / 13)

 
 

 
 
   +     237   31.9  3.9  3.9  contig10_pilon   507580  507886 (1344793) +  rnd-4_family-1875  LTR/Gypsy         5859   6165  (2904)   138     
 
 ANNOTATION EVIDENCE: 
   237  31.87 3.91 3.91  contig10_pilon   507580  507886   1344793 +  rnd-4_family-1875  LTR/Gypsy         5859   6165    2904      
237 31.87 3.91 3.91 contig10_pilon 507580 507886 (1344793) rnd-4_family-1875#LTR/Gypsy 5859 6165 (2904) m_b692s001i4

  contig10_pilo     507580 TCTTCAAACGGTTCGGCCTGTTCAAGAAGCTCATCAGCGACCGCGGCCCG 507629
                              v   i v?iv   vv  iiii  iv v ?i v     i  v iiv  
  rnd-4_family-       5859 TCTACAAGCTNCACGGGATGCCTGAGGCGATNGTGAGCGATCGGGATACG 5908

  contig10_pilo     507630 CAGTTCGTGTCGGGATTCGCGAACGAGCTCTACAAGCTCCTCGGTATCGA 507679
                            v   iii vvv v    vv v vi i  vi  i     vi   ii vi 
  rnd-4_family-       5909 CTGTTTACGAGCGCATTCTGGCAGAAACTGCACGAGCTCACCGGCGTGAA 5958

  contig10_pilo     507680 GGCAAACCCGTCCACCGCGTACCACCCGCAGACCGACGG----ACAGACC 507725
                            viivi?vi   ?v v        i        v     ----  i  v 
  rnd-4_family-       5959 GTTGCGNATGTCNTCGGCGTACCATCCGCAGACGGACGGCGCGACGGAAC 6008

  contig10_pilo     507726 GAGCGGGA-GAACGCGGAGATCGAGAAGTACCTTC-GCGCATGGACCAAC 507773
                                iiv- i    v i -- v   v vv  v  - i  i ?v- ii v
  rnd-4_family-       6009 GAGCGAATCGGACGCTGGG--CCAGATGATCCGTCAGTGCGTNT-CTGAG 6055

  contig10_pilo     507774 GCCCGGCAGGATGACTGGGCC----GATTGGCTCGCTATGGCCGAGTTCG 507819
                           ? v i  i   ---     i?----i    v  -  ?  ---        
  rnd-4_family-       6056 NCGCAGCGGGA---CTGGGTNCAGAAATTGCCT-GCNAT---CGAGTTCG 6098

  contig10_pilo     507820 CCATCAACAACCGCGTG-TCCGAGGCGACGGG-TACGTCTCCGTTCTTCC 507867
                            i  v    i -   i -  v v i ?     -   i- v          
  rnd-4_family-       6099 CTATGAACAGC-GCGCGATCGGCGACNACGGGNTACA-CGCCGTTCTTCC 6146

  contig10_pilo     507868 TCAATCACGGTCGCCACCC 507886
                            v   i    ? ivv    
  rnd-4_family-       6147 TGAATTACGGNCAGAACCC 6165

Matrix = 20p53g.matrix
Kimura (with divCpGMod) = 39.31
Transitions / transversions = 1.00 (47/47)
Gap_init rate = 0.06 (18 / 306), avg. gap size = 1.33 (24 / 18)

 
 
 
 
 
  +      14   11.5  0.0  3.5  contig10_pilon   508253  508282 (1344397) +  (CGCCC)n           Simple_repeat        1     29     (0)   139     
 
 ANNOTATION EVIDENCE: 
    14  11.53 0.00 3.45  contig10_pilon   508253  508282   1344397 +  (CGCCC)n           Simple_repeat        1     29       0      
14 11.53 0.00 3.45 contig10_pilon 508253 508282 (1344397) (CGCCC)n#Simple_repeat 1 29 (0) m_b692s252i6

  contig10_pilo     508253 CGCCCCGCACCAACGCCGCCCCGCCCCGCC 508282
                                   -  vi v               
  (CGCCC)n#Simp          1 CGCCCCGC-CCCGCCCCGCCCCGCCCCGCC 29

Matrix = Unknown
Transitions / transversions = 0.50 (1/2)
Gap_init rate = 0.03 (1 / 29), avg. gap size = 1.00 (1 / 1)

 
 

 
 
   +      11   21.8  2.2  4.4  contig10_pilon   508571  508616 (1344063) +  (CCGCGGC)n         Simple_repeat        1     45     (0)   140     
 
 ANNOTATION EVIDENCE: 
    11  21.82 2.17 4.44  contig10_pilon   508571  508616   1344063 +  (CCGCGGC)n         Simple_repeat        1     45       0      
11 21.82 2.17 4.44 contig10_pilon 508571 508616 (1344063) (CCGCGGC)n#Simple_repeat 1 45 (0) m_b692s252i7

  contig10_pilo     508571 CCCCGACCCG-GTCACCGCGGCCCGCAGGGCGCGGGCCCTCGGCACG 508616
                             v  i    - v -           i vv   -     v    v  
  (CCGCGGC)n#Si          1 CCGCGGCCCGCGGC-CCGCGGCCCGCGGCCCGC-GGCCCGCGGCCCG 45

Matrix = Unknown
Transitions / transversions = 0.33 (2/6)
Gap_init rate = 0.07 (3 / 45), avg. gap size = 1.00 (3 / 3)

 
 
 
 
 
  +    2281    0.7  0.0  0.0  contig10_pilon   508646  508929 (1343750) +  rnd-4_family-1653  LTR/Gypsy            1    284  (1831)   141     
 
 ANNOTATION EVIDENCE: 
  2281   0.70 0.00 0.00  contig10_pilon   508646  508929   1343750 +  rnd-4_family-1653  LTR/Gypsy            1    284    1831      
2281 0.70 0.00 0.00 contig10_pilon 508646 508929 (1343750) rnd-4_family-1653#LTR/Gypsy 1 284 (1831) m_b692s001i5

  contig10_pilo     508646 TGTTACGGCTCATTCGCTTTTCGCTCCTATCGCCGTTGGGCTTATCGGAC 508695
                                                                             
  rnd-4_family-          1 TGTTACGGCTCATTCGCTTTTCGCTCCTATCGCCGTTGGGCTTATCGGAC 50

  contig10_pilo     508696 CTCACTCGCGCCGACGTCATACGGACCGAGTCATCAGACCGAGACCCGCC 508745
                                                                             
  rnd-4_family-         51 CTCACTCGCGCCGACGTCATACGGACCGAGTCATCAGACCGAGACCCGCC 100

  contig10_pilo     508746 GGCGCATGCGTGGCCCCGGCCTGCGGTCAGCGGTCCGGCCGGATCCGGAC 508795
                                                                             
  rnd-4_family-        101 GGCGCATGCGTGGCCCCGGCCTGCGGTCAGCGGTCCGGCCGGATCCGGAC 150

  contig10_pilo     508796 CCCCTTTATATACCGTGTGTTGTCCGCTCTTTGTATGCTAGATTCGAGCT 508845
                                              i               i              
  rnd-4_family-        151 CCCCTTTATATACCGTGTGCTGTCCGCTCTTTGTACGCTAGATTCGAGCT 200

  contig10_pilo     508846 GGTTTGCCCACCGCCGATACATCTCGTCCTTTTTCCCCCACCCGCAGAGC 508895
                                                                             
  rnd-4_family-        201 GGTTTGCCCACCGCCGATACATCTCGTCCTTTTTCCCCCACCCGCAGAGC 250

  contig10_pilo     508896 GTCCGTTCGGCGAGTATCCTCGGTCACCGTAACA 508929
                                                             
  rnd-4_family-        251 GTCCGTTCGGCGAGTATCCTCGGTCACCGTAACA 284

Matrix = 20p53g.matrix
Kimura (with divCpGMod) = 0.39
Transitions / transversions = 1.00 (2/0)
Gap_init rate = 0.00 (0 / 283), avg. gap size = 0.0 (0 / 0)

 
 

 
 
   +      58    1.5  0.0  0.0  contig10_pilon   510919  510984 (1341695) +  (A)n               Simple_repeat        1     66     (0)   142     
 
 ANNOTATION EVIDENCE: 
    58   1.54 0.00 0.00  contig10_pilon   510919  510984   1341695 +  (A)n               Simple_repeat        1     66       0      
58 1.54 0.00 0.00 contig10_pilon 510919 510984 (1341695) (A)n#Simple_repeat 1 66 (0) m_b692s252i8

  contig10_pilo     510919 AAAAAAAAAAAAAAAAAAAAAAAAAAAAAAAAAAAAAAAAAAAAAAAAAA 510968
                                                                             
  (A)n#Simple_r          1 AAAAAAAAAAAAAAAAAAAAAAAAAAAAAAAAAAAAAAAAAAAAAAAAAA 50

  contig10_pilo     510969 AAAAAAAAAAAAAGAA 510984
                                        i  
  (A)n#Simple_r         51 AAAAAAAAAAAAAAAA 66

Matrix = Unknown
Transitions / transversions = 1.00 (1/0)
Gap_init rate = 0.00 (0 / 65), avg. gap size = 0.0 (0 / 0)

 
 
 
 
 
  +      16   27.4  0.0  4.7  contig10_pilon   517514  517580 (1335099) +  (GCGCCG)n          Simple_repeat        1     64     (0)   143     
 
 ANNOTATION EVIDENCE: 
    16  27.42 0.00 4.69  contig10_pilon   517514  517580   1335099 +  (GCGCCG)n          Simple_repeat        1     64       0      
16 27.42 0.00 4.69 contig10_pilon 517514 517580 (1335099) (GCGCCG)n#Simple_repeat 1 64 (0) m_b692s252i9

  contig10_pilo     517514 GCGCGCGCGGCGTCGCGGTCGACGGCGTACGTGCGCGCGGCGCCGCCCTT 517563
                               vv   v  v   v v  v     -v  -    -        v vii
  (GCGCCG)n#Sim          1 GCGCCGGCGCCGGCGCCGGCGCCGGCG-CCG-GCGC-CGGCGCCGGCGCC 47

  contig10_pilo     517564 GCCGCCGGCGCCTGCGC 517580
                            v          v    
  (GCGCCG)n#Sim         48 GGCGCCGGCGCCGGCGC 64

Matrix = Unknown
Transitions / transversions = 0.17 (2/12)
Gap_init rate = 0.05 (3 / 66), avg. gap size = 1.00 (3 / 3)

 
 

 
 
   +      15   16.2  4.3  6.5  contig10_pilon   521159  521205 (1331474) +  (GC)n              Simple_repeat        1     46     (0)   144     
 
 ANNOTATION EVIDENCE: 
    15  16.16 4.26 6.52  contig10_pilon   521159  521205   1331474 +  (GC)n              Simple_repeat        1     46       0      
15 16.16 4.26 6.52 contig10_pilon 521159 521205 (1331474) (GC)n#Simple_repeat 1 46 (0) m_b692s252i10

  contig10_pilo     521159 GCGAGCTGCTCGAGAGCGTCGCGCGCAGCTCGCG-GCGCGTG-GCGCGC 521205
                              v  -  v  v v   -       -  v    -     i -      
  (GC)n#Simple_          1 GCGCGC-GCGCGCGCGCG-CGCGCGC-GCGCGCGCGCGCGCGCGCGCGC 46

Matrix = Unknown
Transitions / transversions = 0.20 (1/5)
Gap_init rate = 0.11 (5 / 46), avg. gap size = 1.00 (5 / 5)

 
 
 
 
 
  +     231   18.5  1.8  1.8  contig10_pilon   552426  552480 (1300199) +  rnd-4_family-2312  Unknown            710    764     (0)   145     
 
 ANNOTATION EVIDENCE: 
   231  18.52 1.82 1.82  contig10_pilon   552426  552480   1300199 +  rnd-4_family-2312  Unknown            710    764       0      
231 18.52 1.82 1.82 contig10_pilon 552426 552480 (1300199) rnd-4_family-2312#Unknown 710 764 (0) m_b693s001i0

  contig10_pilo     552426 TTCTATTGCGGATTCCGGCATCGAACGCTAA-GACCTACTACCAAGGTAG 552474
                             v      i    iv v    iv    i  -    i     -       
  rnd-4_family-        710 TTGTATTGCAGATTTGGTCATCACACGCCAAAGACCCACTAC-AAGGTAG 758

  contig10_pilo     552475 CAGGAA 552480
                            i    
  rnd-4_family-        759 CGGGAA 764

Matrix = 20p53g.matrix
Kimura (with divCpGMod) = 19.26
Transitions / transversions = 1.50 (6/4)
Gap_init rate = 0.04 (2 / 54), avg. gap size = 1.00 (2 / 2)

 
 

 
 
   +     299   30.0  7.1  3.0  contig10_pilon   561495  561621 (1291058) +  rnd-4_family-124   Unknown              1    132  (4931)   146     
 
 ANNOTATION EVIDENCE: 
   299  30.04 7.09 3.03  contig10_pilon   561495  561621   1291058 +  rnd-4_family-124   Unknown              1    132    4931      
299 30.04 7.09 3.03 contig10_pilon 561495 561621 (1291058) rnd-4_family-124#Unknown 1 132 (4931) m_b693s001i1

  contig10_pilo     561495 TGCTACAGTTCTTGGGGATTGTTGCTCATACACCACTCCTGTACAATCTC 561544
                             i   i  v vi  v   i-    i     i  i  i i  vvi     
  rnd-4_family-          1 TGTTACGGTGCGCGGCGATC-TTGCCCATACGCCGCTTCCGTTGGATCTC 49

  contig10_pilo     561545 TTGTGCTGCTTCTCCTAC---------CGGATACACCTGTTCTATTACAT 561585
                             vi  i     v  ii --------- v    i   i    ii---   
  rnd-4_family-         50 TTTCGCCGCTTCACCCGCTCCGTCGGACCGATATACCCGTTCCG---CAT 96

  contig10_pilo     561586 GCCTCTCCAGGTCATCAGCACCTTGGATACTGTCTC 561621
                                   i ii iiii      i  v   iv    
  rnd-4_family-         97 GCCTCTCCGGACCGCTGGCACCTCGGCTACCTTCTC 132

Matrix = 20p53g.matrix
Kimura (with divCpGMod) = 24.22
Transitions / transversions = 2.70 (27/10)
Gap_init rate = 0.04 (5 / 126), avg. gap size = 2.60 (13 / 5)

 
 
 
 
  +      37    0.0  0.0  0.0  contig10_pilon   561622  561655 (1291024) +  (T)n               Simple_repeat        1     34     (0)   147     
 
 ANNOTATION EVIDENCE: 
    37   0.00 0.00 0.00  contig10_pilon   561622  561655   1291024 +  (T)n               Simple_repeat        1     34       0      
37 0.00 0.00 0.00 contig10_pilon 561622 561655 (1291024) (T)n#Simple_repeat 1 34 (0) c_b693s251i0

  contig10_pilo     561622 TTTTTTTTTTTTTTTTTTTTTTTTTTTTTTTTTT 561655
                                                             
  (T)n#Simple_r          1 TTTTTTTTTTTTTTTTTTTTTTTTTTTTTTTTTT 34

Matrix = Unknown
Transitions / transversions = 1.00 (0/0)
Gap_init rate = 0.00 (0 / 33), avg. gap size = 0.0 (0 / 0)

 
 

 
   +    1077   29.9 11.9  1.5  contig10_pilon   569410  569953 (1282726) +  rnd-4_family-124   Unknown            115    714  (4349)   146     
 
 ANNOTATION EVIDENCE: 
  1077  29.86 11.95 1.50  contig10_pilon   569410  569953   1282726 +  rnd-4_family-124   Unknown            115    714    4349      
1077 29.86 11.95 1.50 contig10_pilon 569410 569953 (1282726) rnd-4_family-124#Unknown 115 714 (4349) m_b693s001i2

  contig10_pilo     569410 CACCTTGGATACTGTCTCCCGGCTTTACTGGACCCCTGCTGACCTCAATC 569459
                                i  v   iv    i    v i  i         iii     ii  
  rnd-4_family-        115 CACCTCGGCTACCTTCTCTCGGCGTCACCGGACCCCTGTCAACCTCGGTC 164

  contig10_pilo     569460 CCAGGATGCATTGTGCTGTCACTGTCCTCCTCAACCACATCGATATCTGC 569509
                            iv      ivi i   i ii ii iv iv  i i   --  ----    
  rnd-4_family-        165 CTTGGATGCGACGCGCTATTGCCATTGTTGTCGATCAC--CG----CTGC 208

  contig10_pilo     569510 ATTGATTTCTGACCAGCTCTTAGCC-----------------CTCAATCT 569542
                           i   i    ii      viv     -----------------   ii v 
  rnd-4_family-        209 GTTGGTTTCCAACCAGCGTGTAGCCGTTCGTCGGTCCGTCTCCTCGGTGT 258

  contig10_pilo     569543 CAC--TAC---CTACACCTCTGGAGAAGCAACACACCATACCCACTGGTA 569587
                              --   ---        ii     v  vi       i   i- v    
  rnd-4_family-        259 CACATTACGGCCTACACCTTCGGAGATGCCGCACACCACACCT-CGGGTA 307

  contig10_pilo     569588 TTTCCCCATATTGTCAGTGTGTG--GGGTGTATTGGCCAAGCTACAATGA 569635
                             v   iii  v i i   i i --   v   iv  iv     i   i  
  rnd-4_family-        308 TTGCCCTGCATGGCCGGTGCGCGCTGGGAGTACAGGTGAAGCTGCAACGA 357

  contig10_pilo     569636 TCTTGCTGATCTCCTACCTTGTGCCTCTATCCCGTGGGTGCTCATCCACT 569685
                             v   i  iv v vv    vvv     i    v   i     iv  i  
  rnd-4_family-        358 TCGTGCCGACGTACATCCTTCACCCTCTGTCCCCTGGATGCTCGACCGCT 407

  contig10_pilo     569686 CCAGCCTGGCCAACCTACACCAACATGCATGCCTTGGTTGTTGCCCTGCA 569735
                             i  vivv vi    vvi  iv  ii viv   i   ivvi  v v   
  rnd-4_family-        408 CCGGCACCTCGGACCTCGGCCGCCACACTCTCCTCGGTCCGCGCGCGGCA 457

  contig10_pilo     569736 TGGAATAGGATCCGGAGCCTGTTGTGATGTGCAATTGGAATCTTAATTGC 569785
                              vvi v i       v    ivv  --  v i v   i v  i    i
  rnd-4_family-        458 TGGTCCATGGTCCGGAGGCTGTCCGGA--TGGAGTGGGAGTGTTGATTGT 505

  contig10_pilo     569786 TGGTCAGTGTGCTC----------TGCCGA-----TGTGCT--------- 569811
                              vii   i    ----------i     -----  i   ---------
  rnd-4_family-        506 TGGGTGGTGCGCTCGTACCTCGCTCGCCGACGCGTTGCGCTCGCTGCTGC 555

  contig10_pilo     569812 GCTCATCATTGCA-CAACCATATACACCTACGGTGTC--TGC-----TCC 569853
                               ii      i- ii vviv v i  iv       --   ----- v 
  rnd-4_family-        556 GCTCGCCATTGCGTCGGCGCCTTTCGCCCCCGGTGTCGGTGCGTGCATAC 605

  contig10_pilo     569854 ATGCAGGGACAACTTGGTCTGTCTGGTATATCTGCTCAATACCCTCCATA 569903
                                i     v  i         v v   i i  i i            
  rnd-4_family-        606 ATGCAAGGACACCTCGGTCTGTCTCGGATACCCGCCCGATACCCTCCATA 655

  contig10_pilo     569904 GGCACGAACAACCTTTATTCT---------GAGTTATTCTGTGCCTTTGG 569944
                                 i   v       i i---------    ii     i     i  
  rnd-4_family-        656 GGCACGGACATCCTTTATCCCCTTCGTCCGGAGTCGTTCTGCGCCTTCGG 705

  contig10_pilo     569945 CAAGACACT 569953
                            iv   i  
  rnd-4_family-        706 CGCGACGCT 714

Matrix = 20p53g.matrix
Kimura (with divCpGMod) = 28.44
Transitions / transversions = 1.58 (98/62)
Gap_init rate = 0.04 (20 / 543), avg. gap size = 3.70 (74 / 20)

 
 
 
 
 
  +      11   20.2  2.9  0.0  contig10_pilon   570880  570913 (1281766) +  (CACCAG)n          Simple_repeat        1     35     (0)   148     
 
 ANNOTATION EVIDENCE: 
    11  20.23 2.94 0.00  contig10_pilon   570880  570913   1281766 +  (CACCAG)n          Simple_repeat        1     35       0      
11 20.23 2.94 0.00 contig10_pilon 570880 570913 (1281766) (CACCAG)n#Simple_repeat 1 35 (0) m_b693s252i1

  contig10_pilo     570880 CAGCAGGCCCAGCAGCAGCCCCAGCGCCAGC-CCA 570913
                             v   vv      v    v     i     -   
  (CACCAG)n#Sim          1 CACCAGCACCAGCACCAGCACCAGCACCAGCACCA 35

Matrix = Unknown
Transitions / transversions = 0.20 (1/5)
Gap_init rate = 0.03 (1 / 33), avg. gap size = 1.00 (1 / 1)

 
 

 
 
   +      22    6.5  0.0  0.0  contig10_pilon   571503  571534 (1281145) +  (GCA)n             Simple_repeat        1     32     (0)   149     
 
 ANNOTATION EVIDENCE: 
    22   6.54 0.00 0.00  contig10_pilon   571503  571534   1281145 +  (GCA)n             Simple_repeat        1     32       0      
22 6.54 0.00 0.00 contig10_pilon 571503 571534 (1281145) (GCA)n#Simple_repeat 1 32 (0) m_b693s252i2

  contig10_pilo     571503 GCAGCAGCAGCAGCAGCAGCAGCCGCGGCAGC 571534
                                                  v  i     
  (GCA)n#Simple          1 GCAGCAGCAGCAGCAGCAGCAGCAGCAGCAGC 32

Matrix = Unknown
Transitions / transversions = 1.00 (1/1)
Gap_init rate = 0.00 (0 / 31), avg. gap size = 0.0 (0 / 0)

 
 
 
 
 
  +     227    8.8  0.0  0.0  contig10_pilon   574893  574926 (1277753) +  rnd-4_family-106   LTR/Gypsy         4545   4578 (19129)   150     
 
 ANNOTATION EVIDENCE: 
   227   8.82 0.00 0.00  contig10_pilon   574893  574926   1277753 +  rnd-4_family-106   LTR/Gypsy         4545   4578   19129      
227 8.82 0.00 0.00 contig10_pilon 574893 574926 (1277753) rnd-4_family-106#LTR/Gypsy 4545 4578 (19129) m_b693s001i3

  contig10_pilo     574893 ATTTGACCTACCATCATCGTCATCTATAGAACGT 574926
                                                       vi i  
  rnd-4_family-       4545 ATTTGACCTACCATCATCGTCATCTATACGATGT 4578

Matrix = 20p53g.matrix
Kimura (with divCpGMod) = 6.46
Transitions / transversions = 2.00 (2/1)
Gap_init rate = 0.00 (0 / 33), avg. gap size = 0.0 (0 / 0)

 
 

 
 
   +     767   32.4  6.7  3.4  contig10_pilon   575506  576211 (1276468) +  rnd-4_family-106   LTR/Gypsy        15225  15952  (7755)   151     
 
 ANNOTATION EVIDENCE: 
   767  32.41 6.66 3.43  contig10_pilon   575506  576211   1276468 +  rnd-4_family-106   LTR/Gypsy        15225  15952    7755      
767 32.41 6.66 3.43 contig10_pilon 575506 576211 (1276468) rnd-4_family-106#LTR/Gypsy 15225 15952 (7755) m_b693s001i4

  contig10_pilo     575506 TGCATAGCAGACGGAATCCGCGATTTCTATTCTAACCGCTGACGACTCCC 575555
                               v i v    iv  v    i    i ii v      i       v i
  rnd-4_family-      15225 TGCAGAACCGACGACATACGCGGTTTCCACCCAAACCGCCGACGACTGCT 15274

  contig10_pilo     575556 GACTGCCCATGTTACAGCCATCATCACTGACTCATTGATTGCTGATCAGG 575605
                            ivvvii  i    vvi v    v vv            v      vvv 
  rnd-4_family-      15275 GGGGTTTCACGTTAATACAATCAGCTATGACTCATTGATGGCTGATGCTG 15324

  contig10_pilo     575606 ATGC-----GCGTGGAGCGCGGGAAGTATACGCTGCCGGGAAAC-GCGAG 575649
                              i-----    ivi   v   v-  - i i i    i     -  ivi
  rnd-4_family-      15325 ATGTATCTAGCGTATGGCGAGGGC-GT-TGCACCGCCGAGAAACTGCATA 15372

  contig10_pilo     575650 CGCCGCTGGAA-GGTGTTTGGTAGCATACGGAC----GTTATACCCCTCT 575694
                           i    i     -  vi   v iiv v i i  v---- v   v  v  ii
  rnd-4_family-      15373 TGCCGTTGGAATGGGATTTTGCGCCCTGCAGAGAGGGGGTATCCCGCTTC 15422

  contig10_pilo     575695 GTCCCGTTCTCCTCCGGCCTCAGTGAGAA-TCACGTGACTAATATTA-GA 575742
                           ivi vvi    iivv  --    i v  i-         vvv  i i-  
  rnd-4_family-      15423 AATCGTCTCTCTCAAGG--TCAGCGTGAGGTCACGTGACATTTACTGCGA 15470

  contig10_pilo     575743 GCACGCATGCACGAGAGAGAGATTGGGAGAGTCAGGTCAGGTCGACAATG 575792
                           vvvv   i  vi  vivv i --i  i  v v i  vv      iv    
  rnd-4_family-      15471 TGTGGCACGCCTGATGTTGGG--CGGAAGTGGCGGGAGAGGTCGGGAATG 15518

  contig10_pilo     575793 CCAAA-CCAGTCTGGAAATTTGA--TTCGCACCGCGGCTTTCACGGGCGC 575839
                             i  -  iivvv  i ii i  -- iv ivi    i ---v        
  rnd-4_family-      15519 CCGAATCCGAGGGGGGAGCTCGACCTCAGTCTCGCGAC---AACGGGCGC 15565

  contig10_pilo     575840 TTATTTAGCACGAGGACCTCCAAATTTATTAAGCGCTCATGATTTGGCTG 575889
                                 i v  v   i vii   vvvi    i    v i  v    ivvv
  rnd-4_family-      15566 TTATTTGGGACCAGGGCACTCAATAACATTAGGCGCACGTGTTTTGAGGT 15615

  contig10_pilo     575890 GAGGAAG-GTTCGGGTTGGCGCGAGAAATCGGTTGGATCACGAGCACGGG 575938
                              i   -  i v  -vii iv  i        i v   i  i  i  v 
  rnd-4_family-      15616 GAGAAAGTGTCCTGG-AAACAGGAAAAATCGGTCGTATCGCGGGCGCGCG 15664

  contig10_pilo     575939 ACCAGGAAGGCGTGTTCCAAACTCCGCGCAGCAGGGACAACGC------- 575981
                             v        ii i iv         v    vvv    v   -------
  rnd-4_family-      15665 ACGAGGAAGGCACGCTTGAAACTCCGCTCAGCCTCGACACCGCGGGTGTC 15714

  contig10_pilo     575982 ----CGGCCGCCGCGGTCGC-GGAGCGGCCTCCTCGGCATACAACTGCAG 576026
                           ----    v ii   ii i -      v     i  i vivv    v i 
  rnd-4_family-      15715 GAGTCGGCGGTTGCGACCACAGGAGCGCCCTCCCCGACTCCAAACTTCGG 15764

  contig10_pilo     576027 AAATCAAT--------GCATAAGTGAGTTGCTTAGCTGCCCCGCATAAGA 576068
                           v  -    --------  ii v v v    vv ------i i  i i i 
  rnd-4_family-      15765 TAA-CAATATTATTTCGCGCACGGGCGTTGGAT------TCTGCGTGAAA 15807

  contig10_pilo     576069 TTTTGGCAGGACGAGGCCCAGAAACGCG-CCGGAGGCTGGG-AGTCGATC 576116
                           -  i iivi              vi  i-       ii   - iv ii i
  rnd-4_family-      15808 -TTCGATTAGACGAGGCCCAGAACTGCACCCGGAGGTCGGGCAAACAGTT 15856

  contig10_pilo     576117 CAGCCATTCAGCGAAAA-CGGCGGTCGTTGCGTTGGCACAT--CGTTTCA 576163
                           vi          i    - i      i iv i  --  -  --   v   
  rnd-4_family-      15857 GGGCCATTCAGCAAAAACCAGCGGTCATCTCATT--CA-ATCGCGTATCA 15903

  contig10_pilo     576164 CGGCCATCAGGTTCGCA-TTAG--AAGCGT--GATGGCGTGCAGATCCTG 576208
                                    -- v i  -i  i--      --        --      i 
  rnd-4_family-      15904 CGGCCATCA--TACACAGCTAAACAAGCGTGAGATGGCGT--AGATCCCG 15949

  contig10_pilo     576209 CCG 576211
                            i 
  rnd-4_family-      15950 CTG 15952

Matrix = 20p53g.matrix
Kimura (with divCpGMod) = 37.99
Transitions / transversions = 1.19 (120/101)
Gap_init rate = 0.06 (45 / 705), avg. gap size = 1.60 (72 / 45)

 
 
 
 
 
  +      11   20.2  2.9  0.0  contig10_pilon   580709  580742 (1271937) +  (CACCAG)n          Simple_repeat        1     35     (0)   152     
 
 ANNOTATION EVIDENCE: 
    11  20.23 2.94 0.00  contig10_pilon   580709  580742   1271937 +  (CACCAG)n          Simple_repeat        1     35       0      
11 20.23 2.94 0.00 contig10_pilon 580709 580742 (1271937) (CACCAG)n#Simple_repeat 1 35 (0) m_b694s252i0

  contig10_pilo     580709 CAGCAGGCCCAGCAGCAGCCCCAGCGCCAGC-CCA 580742
                             v   vv      v    v     i     -   
  (CACCAG)n#Sim          1 CACCAGCACCAGCACCAGCACCAGCACCAGCACCA 35

Matrix = Unknown
Transitions / transversions = 0.20 (1/5)
Gap_init rate = 0.03 (1 / 33), avg. gap size = 1.00 (1 / 1)

 
 

 
 
   +      22    6.5  0.0  0.0  contig10_pilon   581329  581360 (1271319) +  (GCA)n             Simple_repeat        1     32     (0)   153     
 
 ANNOTATION EVIDENCE: 
    22   6.54 0.00 0.00  contig10_pilon   581329  581360   1271319 +  (GCA)n             Simple_repeat        1     32       0      
22 6.54 0.00 0.00 contig10_pilon 581329 581360 (1271319) (GCA)n#Simple_repeat 1 32 (0) m_b694s252i1

  contig10_pilo     581329 GCAGCAGCAGCAGCAGCAGCAGCCGCGGCAGC 581360
                                                  v  i     
  (GCA)n#Simple          1 GCAGCAGCAGCAGCAGCAGCAGCAGCAGCAGC 32

Matrix = Unknown
Transitions / transversions = 1.00 (1/1)
Gap_init rate = 0.00 (0 / 31), avg. gap size = 0.0 (0 / 0)

 
 
 
 
 
  +     227    8.8  0.0  0.0  contig10_pilon   584719  584752 (1267927) +  rnd-4_family-106   LTR/Gypsy         4545   4578 (19129)   154     
 
 ANNOTATION EVIDENCE: 
   227   8.82 0.00 0.00  contig10_pilon   584719  584752   1267927 +  rnd-4_family-106   LTR/Gypsy         4545   4578   19129      
227 8.82 0.00 0.00 contig10_pilon 584719 584752 (1267927) rnd-4_family-106#LTR/Gypsy 4545 4578 (19129) m_b694s001i0

  contig10_pilo     584719 ATTTGACCTACCATCATCGTCATCTATAGAACGT 584752
                                                       vi i  
  rnd-4_family-       4545 ATTTGACCTACCATCATCGTCATCTATACGATGT 4578

Matrix = 20p53g.matrix
Kimura (with divCpGMod) = 6.46
Transitions / transversions = 2.00 (2/1)
Gap_init rate = 0.00 (0 / 33), avg. gap size = 0.0 (0 / 0)

 
 

 
 
   +     767   32.4  6.7  3.4  contig10_pilon   585332  586037 (1266642) +  rnd-4_family-106   LTR/Gypsy        15225  15952  (7755)   155     
 
 ANNOTATION EVIDENCE: 
   767  32.41 6.66 3.43  contig10_pilon   585332  586037   1266642 +  rnd-4_family-106   LTR/Gypsy        15225  15952    7755      
767 32.41 6.66 3.43 contig10_pilon 585332 586037 (1266642) rnd-4_family-106#LTR/Gypsy 15225 15952 (7755) m_b694s001i1

  contig10_pilo     585332 TGCATAGCAGACGGAATCCGCGATTTCTATTCTAACCGCTGACGACTCCC 585381
                               v i v    iv  v    i    i ii v      i       v i
  rnd-4_family-      15225 TGCAGAACCGACGACATACGCGGTTTCCACCCAAACCGCCGACGACTGCT 15274

  contig10_pilo     585382 GACTGCCCATGTTACAGCCATCATCACTGACTCATTGATTGCTGATCAGG 585431
                            ivvvii  i    vvi v    v vv            v      vvv 
  rnd-4_family-      15275 GGGGTTTCACGTTAATACAATCAGCTATGACTCATTGATGGCTGATGCTG 15324

  contig10_pilo     585432 ATGC-----GCGTGGAGCGCGGGAAGTATACGCTGCCGGGAAAC-GCGAG 585475
                              i-----    ivi   v   v-  - i i i    i     -  ivi
  rnd-4_family-      15325 ATGTATCTAGCGTATGGCGAGGGC-GT-TGCACCGCCGAGAAACTGCATA 15372

  contig10_pilo     585476 CGCCGCTGGAA-GGTGTTTGGTAGCATACGGAC----GTTATACCCCTCT 585520
                           i    i     -  vi   v iiv v i i  v---- v   v  v  ii
  rnd-4_family-      15373 TGCCGTTGGAATGGGATTTTGCGCCCTGCAGAGAGGGGGTATCCCGCTTC 15422

  contig10_pilo     585521 GTCCCGTTCTCCTCCGGCCTCAGTGAGAA-TCACGTGACTAATATTA-GA 585568
                           ivi vvi    iivv  --    i v  i-         vvv  i i-  
  rnd-4_family-      15423 AATCGTCTCTCTCAAGG--TCAGCGTGAGGTCACGTGACATTTACTGCGA 15470

  contig10_pilo     585569 GCACGCATGCACGAGAGAGAGATTGGGAGAGTCAGGTCAGGTCGACAATG 585618
                           vvvv   i  vi  vivv i --i  i  v v i  vv      iv    
  rnd-4_family-      15471 TGTGGCACGCCTGATGTTGGG--CGGAAGTGGCGGGAGAGGTCGGGAATG 15518

  contig10_pilo     585619 CCAAA-CCAGTCTGGAAATTTGA--TTCGCACCGCGGCTTTCACGGGCGC 585665
                             i  -  iivvv  i ii i  -- iv ivi    i ---v        
  rnd-4_family-      15519 CCGAATCCGAGGGGGGAGCTCGACCTCAGTCTCGCGAC---AACGGGCGC 15565

  contig10_pilo     585666 TTATTTAGCACGAGGACCTCCAAATTTATTAAGCGCTCATGATTTGGCTG 585715
                                 i v  v   i vii   vvvi    i    v i  v    ivvv
  rnd-4_family-      15566 TTATTTGGGACCAGGGCACTCAATAACATTAGGCGCACGTGTTTTGAGGT 15615

  contig10_pilo     585716 GAGGAAG-GTTCGGGTTGGCGCGAGAAATCGGTTGGATCACGAGCACGGG 585764
                              i   -  i v  -vii iv  i        i v   i  i  i  v 
  rnd-4_family-      15616 GAGAAAGTGTCCTGG-AAACAGGAAAAATCGGTCGTATCGCGGGCGCGCG 15664

  contig10_pilo     585765 ACCAGGAAGGCGTGTTCCAAACTCCGCGCAGCAGGGACAACGC------- 585807
                             v        ii i iv         v    vvv    v   -------
  rnd-4_family-      15665 ACGAGGAAGGCACGCTTGAAACTCCGCTCAGCCTCGACACCGCGGGTGTC 15714

  contig10_pilo     585808 ----CGGCCGCCGCGGTCGC-GGAGCGGCCTCCTCGGCATACAACTGCAG 585852
                           ----    v ii   ii i -      v     i  i vivv    v i 
  rnd-4_family-      15715 GAGTCGGCGGTTGCGACCACAGGAGCGCCCTCCCCGACTCCAAACTTCGG 15764

  contig10_pilo     585853 AAATCAAT--------GCATAAGTGAGTTGCTTAGCTGCCCCGCATAAGA 585894
                           v  -    --------  ii v v v    vv ------i i  i i i 
  rnd-4_family-      15765 TAA-CAATATTATTTCGCGCACGGGCGTTGGAT------TCTGCGTGAAA 15807

  contig10_pilo     585895 TTTTGGCAGGACGAGGCCCAGAAACGCG-CCGGAGGCTGGG-AGTCGATC 585942
                           -  i iivi              vi  i-       ii   - iv ii i
  rnd-4_family-      15808 -TTCGATTAGACGAGGCCCAGAACTGCACCCGGAGGTCGGGCAAACAGTT 15856

  contig10_pilo     585943 CAGCCATTCAGCGAAAA-CGGCGGTCGTTGCGTTGGCACAT--CGTTTCA 585989
                           vi          i    - i      i iv i  --  -  --   v   
  rnd-4_family-      15857 GGGCCATTCAGCAAAAACCAGCGGTCATCTCATT--CA-ATCGCGTATCA 15903

  contig10_pilo     585990 CGGCCATCAGGTTCGCA-TTAG--AAGCGT--GATGGCGTGCAGATCCTG 586034
                                    -- v i  -i  i--      --        --      i 
  rnd-4_family-      15904 CGGCCATCA--TACACAGCTAAACAAGCGTGAGATGGCGT--AGATCCCG 15949

  contig10_pilo     586035 CCG 586037
                            i 
  rnd-4_family-      15950 CTG 15952

Matrix = 20p53g.matrix
Kimura (with divCpGMod) = 37.99
Transitions / transversions = 1.19 (120/101)
Gap_init rate = 0.06 (45 / 705), avg. gap size = 1.60 (72 / 45)

 
 
 
 
 
  +      11   20.2  2.9  0.0  contig10_pilon   590238  590271 (1262408) +  (CACCAG)n          Simple_repeat        1     35     (0)   156     
 
 ANNOTATION EVIDENCE: 
    11  20.23 2.94 0.00  contig10_pilon   590238  590271   1262408 +  (CACCAG)n          Simple_repeat        1     35       0      
11 20.23 2.94 0.00 contig10_pilon 590238 590271 (1262408) (CACCAG)n#Simple_repeat 1 35 (0) m_b694s252i2

  contig10_pilo     590238 CAGCAGGCCCAGCAGCAGCCCCAGCGCCAGC-CCA 590271
                             v   vv      v    v     i     -   
  (CACCAG)n#Sim          1 CACCAGCACCAGCACCAGCACCAGCACCAGCACCA 35

Matrix = Unknown
Transitions / transversions = 0.20 (1/5)
Gap_init rate = 0.03 (1 / 33), avg. gap size = 1.00 (1 / 1)

 
 

 
 
   +      22    6.5  0.0  0.0  contig10_pilon   590858  590889 (1261790) +  (GCA)n             Simple_repeat        1     32     (0)   157     
 
 ANNOTATION EVIDENCE: 
    22   6.54 0.00 0.00  contig10_pilon   590858  590889   1261790 +  (GCA)n             Simple_repeat        1     32       0      
22 6.54 0.00 0.00 contig10_pilon 590858 590889 (1261790) (GCA)n#Simple_repeat 1 32 (0) m_b694s252i3

  contig10_pilo     590858 GCAGCAGCAGCAGCAGCAGCAGCCGCGGCAGC 590889
                                                  v  i     
  (GCA)n#Simple          1 GCAGCAGCAGCAGCAGCAGCAGCAGCAGCAGC 32

Matrix = Unknown
Transitions / transversions = 1.00 (1/1)
Gap_init rate = 0.00 (0 / 31), avg. gap size = 0.0 (0 / 0)

 
 
 
 
 
  +     227    8.8  0.0  0.0  contig10_pilon   594248  594281 (1258398) +  rnd-4_family-106   LTR/Gypsy         4545   4578 (19129)   158     
 
 ANNOTATION EVIDENCE: 
   227   8.82 0.00 0.00  contig10_pilon   594248  594281   1258398 +  rnd-4_family-106   LTR/Gypsy         4545   4578   19129      
227 8.82 0.00 0.00 contig10_pilon 594248 594281 (1258398) rnd-4_family-106#LTR/Gypsy 4545 4578 (19129) m_b694s001i2

  contig10_pilo     594248 ATTTGACCTACCATCATCGTCATCTATAGAACGT 594281
                                                       vi i  
  rnd-4_family-       4545 ATTTGACCTACCATCATCGTCATCTATACGATGT 4578

Matrix = 20p53g.matrix
Kimura (with divCpGMod) = 6.46
Transitions / transversions = 2.00 (2/1)
Gap_init rate = 0.00 (0 / 33), avg. gap size = 0.0 (0 / 0)

 
 

 
 
   +     767   32.4  6.7  3.4  contig10_pilon   594861  595566 (1257113) +  rnd-4_family-106   LTR/Gypsy        15225  15952  (7755)   159     
 
 ANNOTATION EVIDENCE: 
   767  32.41 6.66 3.43  contig10_pilon   594861  595566   1257113 +  rnd-4_family-106   LTR/Gypsy        15225  15952    7755      
767 32.41 6.66 3.43 contig10_pilon 594861 595566 (1257113) rnd-4_family-106#LTR/Gypsy 15225 15952 (7755) m_b694s001i3

  contig10_pilo     594861 TGCATAGCAGACGGAATCCGCGATTTCTATTCTAACCGCTGACGACTCCC 594910
                               v i v    iv  v    i    i ii v      i       v i
  rnd-4_family-      15225 TGCAGAACCGACGACATACGCGGTTTCCACCCAAACCGCCGACGACTGCT 15274

  contig10_pilo     594911 GACTGCCCATGTTACAGCCATCATCACTGACTCATTGATTGCTGATCAGG 594960
                            ivvvii  i    vvi v    v vv            v      vvv 
  rnd-4_family-      15275 GGGGTTTCACGTTAATACAATCAGCTATGACTCATTGATGGCTGATGCTG 15324

  contig10_pilo     594961 ATGC-----GCGTGGAGCGCGGGAAGTATACGCTGCCGGGAAAC-GCGAG 595004
                              i-----    ivi   v   v-  - i i i    i     -  ivi
  rnd-4_family-      15325 ATGTATCTAGCGTATGGCGAGGGC-GT-TGCACCGCCGAGAAACTGCATA 15372

  contig10_pilo     595005 CGCCGCTGGAA-GGTGTTTGGTAGCATACGGAC----GTTATACCCCTCT 595049
                           i    i     -  vi   v iiv v i i  v---- v   v  v  ii
  rnd-4_family-      15373 TGCCGTTGGAATGGGATTTTGCGCCCTGCAGAGAGGGGGTATCCCGCTTC 15422

  contig10_pilo     595050 GTCCCGTTCTCCTCCGGCCTCAGTGAGAA-TCACGTGACTAATATTA-GA 595097
                           ivi vvi    iivv  --    i v  i-         vvv  i i-  
  rnd-4_family-      15423 AATCGTCTCTCTCAAGG--TCAGCGTGAGGTCACGTGACATTTACTGCGA 15470

  contig10_pilo     595098 GCACGCATGCACGAGAGAGAGATTGGGAGAGTCAGGTCAGGTCGACAATG 595147
                           vvvv   i  vi  vivv i --i  i  v v i  vv      iv    
  rnd-4_family-      15471 TGTGGCACGCCTGATGTTGGG--CGGAAGTGGCGGGAGAGGTCGGGAATG 15518

  contig10_pilo     595148 CCAAA-CCAGTCTGGAAATTTGA--TTCGCACCGCGGCTTTCACGGGCGC 595194
                             i  -  iivvv  i ii i  -- iv ivi    i ---v        
  rnd-4_family-      15519 CCGAATCCGAGGGGGGAGCTCGACCTCAGTCTCGCGAC---AACGGGCGC 15565

  contig10_pilo     595195 TTATTTAGCACGAGGACCTCCAAATTTATTAAGCGCTCATGATTTGGCTG 595244
                                 i v  v   i vii   vvvi    i    v i  v    ivvv
  rnd-4_family-      15566 TTATTTGGGACCAGGGCACTCAATAACATTAGGCGCACGTGTTTTGAGGT 15615

  contig10_pilo     595245 GAGGAAG-GTTCGGGTTGGCGCGAGAAATCGGTTGGATCACGAGCACGGG 595293
                              i   -  i v  -vii iv  i        i v   i  i  i  v 
  rnd-4_family-      15616 GAGAAAGTGTCCTGG-AAACAGGAAAAATCGGTCGTATCGCGGGCGCGCG 15664

  contig10_pilo     595294 ACCAGGAAGGCGTGTTCCAAACTCCGCGCAGCAGGGACAACGC------- 595336
                             v        ii i iv         v    vvv    v   -------
  rnd-4_family-      15665 ACGAGGAAGGCACGCTTGAAACTCCGCTCAGCCTCGACACCGCGGGTGTC 15714

  contig10_pilo     595337 ----CGGCCGCCGCGGTCGC-GGAGCGGCCTCCTCGGCATACAACTGCAG 595381
                           ----    v ii   ii i -      v     i  i vivv    v i 
  rnd-4_family-      15715 GAGTCGGCGGTTGCGACCACAGGAGCGCCCTCCCCGACTCCAAACTTCGG 15764

  contig10_pilo     595382 AAATCAAT--------GCATAAGTGAGTTGCTTAGCTGCCCCGCATAAGA 595423
                           v  -    --------  ii v v v    vv ------i i  i i i 
  rnd-4_family-      15765 TAA-CAATATTATTTCGCGCACGGGCGTTGGAT------TCTGCGTGAAA 15807

  contig10_pilo     595424 TTTTGGCAGGACGAGGCCCAGAAACGCG-CCGGAGGCTGGG-AGTCGATC 595471
                           -  i iivi              vi  i-       ii   - iv ii i
  rnd-4_family-      15808 -TTCGATTAGACGAGGCCCAGAACTGCACCCGGAGGTCGGGCAAACAGTT 15856

  contig10_pilo     595472 CAGCCATTCAGCGAAAA-CGGCGGTCGTTGCGTTGGCACAT--CGTTTCA 595518
                           vi          i    - i      i iv i  --  -  --   v   
  rnd-4_family-      15857 GGGCCATTCAGCAAAAACCAGCGGTCATCTCATT--CA-ATCGCGTATCA 15903

  contig10_pilo     595519 CGGCCATCAGGTTCGCA-TTAG--AAGCGT--GATGGCGTGCAGATCCTG 595563
                                    -- v i  -i  i--      --        --      i 
  rnd-4_family-      15904 CGGCCATCA--TACACAGCTAAACAAGCGTGAGATGGCGT--AGATCCCG 15949

  contig10_pilo     595564 CCG 595566
                            i 
  rnd-4_family-      15950 CTG 15952

Matrix = 20p53g.matrix
Kimura (with divCpGMod) = 37.99
Transitions / transversions = 1.19 (120/101)
Gap_init rate = 0.06 (45 / 705), avg. gap size = 1.60 (72 / 45)

 
 
 
 
 
  +      11   20.2  2.9  0.0  contig10_pilon   599767  599800 (1252879) +  (CACCAG)n          Simple_repeat        1     35     (0)   160     
 
 ANNOTATION EVIDENCE: 
    11  20.23 2.94 0.00  contig10_pilon   599767  599800   1252879 +  (CACCAG)n          Simple_repeat        1     35       0      
11 20.23 2.94 0.00 contig10_pilon 599767 599800 (1252879) (CACCAG)n#Simple_repeat 1 35 (0) m_b694s252i4

  contig10_pilo     599767 CAGCAGGCCCAGCAGCAGCCCCAGCGCCAGC-CCA 599800
                             v   vv      v    v     i     -   
  (CACCAG)n#Sim          1 CACCAGCACCAGCACCAGCACCAGCACCAGCACCA 35

Matrix = Unknown
Transitions / transversions = 0.20 (1/5)
Gap_init rate = 0.03 (1 / 33), avg. gap size = 1.00 (1 / 1)

 
 

 
 
   +      22    6.5  0.0  0.0  contig10_pilon   600387  600418 (1252261) +  (GCA)n             Simple_repeat        1     32     (0)   161     
 
 ANNOTATION EVIDENCE: 
    22   6.54 0.00 0.00  contig10_pilon   600387  600418   1252261 +  (GCA)n             Simple_repeat        1     32       0      
22 6.54 0.00 0.00 contig10_pilon 600387 600418 (1252261) (GCA)n#Simple_repeat 1 32 (0) m_b694s252i5

  contig10_pilo     600387 GCAGCAGCAGCAGCAGCAGCAGCCGCGGCAGC 600418
                                                  v  i     
  (GCA)n#Simple          1 GCAGCAGCAGCAGCAGCAGCAGCAGCAGCAGC 32

Matrix = Unknown
Transitions / transversions = 1.00 (1/1)
Gap_init rate = 0.00 (0 / 31), avg. gap size = 0.0 (0 / 0)

 
 
 
 
 
  +      15   20.1  0.0  0.0  contig10_pilon   601093  601126 (1251553) +  (GGC)n             Simple_repeat        1     34     (0)   162     
 
 ANNOTATION EVIDENCE: 
    15  20.12 0.00 0.00  contig10_pilon   601093  601126   1251553 +  (GGC)n             Simple_repeat        1     34       0      
15 20.12 0.00 0.00 contig10_pilon 601093 601126 (1251553) (GGC)n#Simple_repeat 1 34 (0) m_b694s252i6

  contig10_pilo     601093 GGCGGCCGCGGCCGCGGCACAGGTGGCGGCGGCG 601126
                                 v     v     ivv  i          
  (GGC)n#Simple          1 GGCGGCGGCGGCGGCGGCGGCGGCGGCGGCGGCG 34

Matrix = Unknown
Transitions / transversions = 0.50 (2/4)
Gap_init rate = 0.00 (0 / 33), avg. gap size = 0.0 (0 / 0)

 
 

 
 
   +      23   18.2  5.7  4.5  contig10_pilon   602004  602093 (1250586) +  (CGGCGCG)n         Simple_repeat        1     91     (0)   163     
 
 ANNOTATION EVIDENCE: 
    23  18.16 5.68 4.49  contig10_pilon   602004  602091   1250588 +  (CGGCGCG)n         Simple_repeat        1     89       0      
23 18.16 5.68 4.49 contig10_pilon 602004 602091 (1250588) (CGGCGCG)n#Simple_repeat 1 89 (0) m_b694s252i7

  contig10_pilo     602004 CGGTGCGCGAACCCGCCGCGCGCGAGC-AACGGCGGGCAGAGCGCCG-GC 602051
                              i     -i v   v       -  -vi     v  i v    v -  
  (CGGCGCG)n#Si          1 CGGCGCGCG-GCGCGCGGCGCGCG-GCGCGCGGCGCGCGGCGCGCGGCGC 48

  contig10_pilo     602052 G-GGC-CGCTGCGCAGCGCGCGCTCGGCGGGCGGCGCG-GGCG 602091
                            -   -   v    -   -    v     v        -    
  (CGGCGCG)n#Si         49 GCGGCGCGCGGCGC-GCG-GCGCGCGGCGCGCGGCGCGCGGCG 89

Matrix = Unknown
Transitions / transversions = 0.44 (4/9)
Gap_init rate = 0.10 (9 / 87), avg. gap size = 1.00 (9 / 9)

    15  23.65 1.67 5.17  contig10_pilon   602034  602093   1250586 +  (GGCG)n            Simple_repeat        1     58       0      
15 23.65 1.67 5.17 contig10_pilon 602034 602093 (1250586) (GGCG)n#Simple_repeat 1 58 (0) m_b694s252i8

  contig10_pilo     602034 GGCGGGC-AGAGCGCCGGCGGGCCGCTGCGCAGCGCGCGCTCGGCGGGCG 602082
                                  -i v v  v       v vi v  - v   v  -v        
  (GGCG)n#Simpl          1 GGCGGGCGGGCGGGCGGGCGGGCGGGCGGGC-GGGCGGGC-GGGCGGGCG 48

  contig10_pilo     602083 GCGCGGGCGGG 602093
                            -         
  (GGCG)n#Simpl         49 G-GCGGGCGGG 58

Matrix = Unknown
Transitions / transversions = 0.22 (2/9)
Gap_init rate = 0.07 (4 / 59), avg. gap size = 1.00 (4 / 4)

 
 
 
 
 
  +      26   13.7  0.0  3.9  contig10_pilon   602501  602554 (1250125) +  (TTCT)n            Simple_repeat        1     52     (0)   164     
 
 ANNOTATION EVIDENCE: 
    26  13.65 0.00 3.85  contig10_pilon   602501  602554   1250125 +  (TTCT)n            Simple_repeat        1     52       0      
26 13.65 0.00 3.85 contig10_pilon 602501 602554 (1250125) (TTCT)n#Simple_repeat 1 52 (0) m_b694s252i10

  contig10_pilo     602501 TTCTTTCCTTTTTTTTTCCTTTTTTTCTTTCCTTCTTTTCTTTTCTTTCT 602550
                                  i  i   i  i    i        i   -    -         
  (TTCT)n#Simpl          1 TTCTTTCTTTCTTTCTTTCTTTCTTTCTTTCTTTC-TTTC-TTTCTTTCT 48

  contig10_pilo     602551 TTCT 602554
                               
  (TTCT)n#Simpl         49 TTCT 52

Matrix = Unknown
Transitions / transversions = 1.00 (6/0)
Gap_init rate = 0.04 (2 / 53), avg. gap size = 1.00 (2 / 2)

 
 

 
 
   +      15   13.9  0.0  0.0  contig10_pilon   602922  602945 (1249734) +  (CT)n              Simple_repeat        1     24     (0)   165     
 
 ANNOTATION EVIDENCE: 
    15  13.86 0.00 0.00  contig10_pilon   602922  602945   1249734 +  (CT)n              Simple_repeat        1     24       0      
15 13.86 0.00 0.00 contig10_pilon 602922 602945 (1249734) (CT)n#Simple_repeat 1 24 (0) m_b694s252i11

  contig10_pilo     602922 CTCTCTCCCTCTCGCTCTCTCCCT 602945
                                  i     v       i  
  (CT)n#Simple_          1 CTCTCTCTCTCTCTCTCTCTCTCT 24

Matrix = Unknown
Transitions / transversions = 2.00 (2/1)
Gap_init rate = 0.00 (0 / 23), avg. gap size = 0.0 (0 / 0)

 
 
 
 
 
  +      13   25.0  0.0  0.0  contig10_pilon   610457  610494 (1242185) +  (GAAGAC)n          Simple_repeat        1     38     (0)   166     
 
 ANNOTATION EVIDENCE: 
    13  25.00 0.00 0.00  contig10_pilon   610457  610494   1242185 +  (GAAGAC)n          Simple_repeat        1     38       0      
13 25.00 0.00 0.00 contig10_pilon 610457 610494 (1242185) (GAAGAC)n#Simple_repeat 1 38 (0) m_b694s252i12

  contig10_pilo     610457 GACGACGAAGAGGAAGGAGATGACGATGCCGAAGAAGA 610494
                             v        v    iv  v     v v      v  
  (GAAGAC)n#Sim          1 GAAGACGAAGACGAAGACGAAGACGAAGACGAAGACGA 38

Matrix = Unknown
Transitions / transversions = 0.14 (1/7)
Gap_init rate = 0.00 (0 / 37), avg. gap size = 0.0 (0 / 0)

 
 

 
 
   +      17    7.2  3.5  0.0  contig10_pilon   614791  614819 (1237860) +  (GAAC)n            Simple_repeat        1     30     (0)   167     
 
 ANNOTATION EVIDENCE: 
    17   7.25 3.45 0.00  contig10_pilon   614791  614819   1237860 +  (GAAC)n            Simple_repeat        1     30       0      
17 7.25 3.45 0.00 contig10_pilon 614791 614819 (1237860) (GAAC)n#Simple_repeat 1 30 (0) m_b694s252i13

  contig10_pilo     614791 GAGCGAACGAACGAACG-ACGAACGAAGGA 614819
                             i              -         v  
  (GAAC)n#Simpl          1 GAACGAACGAACGAACGAACGAACGAACGA 30

Matrix = Unknown
Transitions / transversions = 1.00 (1/1)
Gap_init rate = 0.04 (1 / 28), avg. gap size = 1.00 (1 / 1)

 
 
 
 
 
  +      16   20.0  5.0  0.0  contig10_pilon   615154  615193 (1237486) +  (CG)n              Simple_repeat        1     42     (0)   168     
 
 ANNOTATION EVIDENCE: 
    16  19.96 5.00 0.00  contig10_pilon   615154  615193   1237486 +  (CG)n              Simple_repeat        1     42       0      
16 19.96 5.00 0.00 contig10_pilon 615154 615193 (1237486) (CG)n#Simple_repeat 1 42 (0) m_b694s252i14

  contig10_pilo     615154 CGCGAGCGCGCG-GCCCG-GTACGGGCGCGCGCACGCTCGCG 615193
                               v       -  v  - ii  v        i   v    
  (CG)n#Simple_          1 CGCGCGCGCGCGCGCGCGCGCGCGCGCGCGCGCGCGCGCGCG 42

Matrix = Unknown
Transitions / transversions = 0.75 (3/4)
Gap_init rate = 0.05 (2 / 39), avg. gap size = 1.00 (2 / 2)

 
 

 
 
   +      13    5.3  0.0  0.0  contig10_pilon   616163  616182 (1236497) +  (CCG)n             Simple_repeat        1     20     (0)   169     
 
 ANNOTATION EVIDENCE: 
    13   5.27 0.00 0.00  contig10_pilon   616163  616182   1236497 +  (CCG)n             Simple_repeat        1     20       0      
13 5.27 0.00 0.00 contig10_pilon 616163 616182 (1236497) (CCG)n#Simple_repeat 1 20 (0) m_b694s252i15

  contig10_pilo     616163 CCGCCGCCGCCGCCACCGCC 616182
                                         i     
  (CCG)n#Simple          1 CCGCCGCCGCCGCCGCCGCC 20

Matrix = Unknown
Transitions / transversions = 1.00 (1/0)
Gap_init rate = 0.00 (0 / 19), avg. gap size = 0.0 (0 / 0)

 
 
 
 
 
  +      15    4.7  0.0  0.0  contig10_pilon   623065  623086 (1229593) +  (CTTA)n            Simple_repeat        1     22     (0)   170     
 
 ANNOTATION EVIDENCE: 
    15   4.71 0.00 0.00  contig10_pilon   623065  623086   1229593 +  (CTTA)n            Simple_repeat        1     22       0      
15 4.71 0.00 0.00 contig10_pilon 623065 623086 (1229593) (CTTA)n#Simple_repeat 1 22 (0) m_b694s252i16

  contig10_pilo     623065 CTTACTTACTTACTTTCTTACT 623086
                                          v      
  (CTTA)n#Simpl          1 CTTACTTACTTACTTACTTACT 22

Matrix = Unknown
Transitions / transversions = 0.00 (0/1)
Gap_init rate = 0.00 (0 / 21), avg. gap size = 0.0 (0 / 0)

 
 

 
 
   +      28    4.3  3.9  3.9  contig10_pilon   636830  636881 (1215798) +  (GCGTCGAGA)n       Simple_repeat        1     52     (0)   171     
 
 ANNOTATION EVIDENCE: 
    28   4.34 3.85 3.85  contig10_pilon   636830  636881   1215798 +  (GCGTCGAGA)n       Simple_repeat        1     52       0      
28 4.34 3.85 3.85 contig10_pilon 636830 636881 (1215798) (GCGTCGAGA)n#Simple_repeat 1 52 (0) c_b694s251i0

  contig10_pilo     636830 GCGTCGAGAGCGCCGACG-GCGTCGAGAGCGCCGACG-GCGTCGAGAGCG 636877
                                       i   - -            i   - -            
  (GCGTCGAGA)n#          1 GCGTCGAGAGCGTCGA-GAGCGTCGAGAGCGTCGA-GAGCGTCGAGAGCG 48

  contig10_pilo     636878 TCGA 636881
                               
  (GCGTCGAGA)n#         49 TCGA 52

Matrix = Unknown
Transitions / transversions = 1.00 (2/0)
Gap_init rate = 0.08 (4 / 51), avg. gap size = 1.00 (4 / 4)

 
 
 
 
 
  +      30    0.0  0.0  0.0  contig10_pilon   645450  645482 (1207197) +  (A)n               Simple_repeat        1     33     (0)   172     
 
 ANNOTATION EVIDENCE: 
    30   0.00 0.00 0.00  contig10_pilon   645450  645482   1207197 +  (A)n               Simple_repeat        1     33       0      
30 0.00 0.00 0.00 contig10_pilon 645450 645482 (1207197) (A)n#Simple_repeat 1 33 (0) m_b695s252i0

  contig10_pilo     645450 AAAAAAAAAAAAAAAAAAAAAAAAAAAAAAAAA 645482
                                                            
  (A)n#Simple_r          1 AAAAAAAAAAAAAAAAAAAAAAAAAAAAAAAAA 33

Matrix = Unknown
Transitions / transversions = 1.00 (0/0)
Gap_init rate = 0.00 (0 / 32), avg. gap size = 0.0 (0 / 0)

 
 

 
 
   +      16   15.4  6.4  4.2  contig10_pilon   653863  653909 (1198770) +  (CG)n              Simple_repeat        1     48     (0)   173     
 
 ANNOTATION EVIDENCE: 
    16  15.37 6.38 4.17  contig10_pilon   653863  653909   1198770 +  (CG)n              Simple_repeat        1     48       0      
16 15.37 6.38 4.17 contig10_pilon 653863 653909 (1198770) (CG)n#Simple_repeat 1 48 (0) m_b695s252i1

  contig10_pilo     653863 CGCGCACGCAGCGCGTCCTCGCGCGCAACGC-CGC-CGAGC-CGCGCGCG 653909
                                i   -     iv v       -i   -   -  v  -        
  (CG)n#Simple_          1 CGCGCGCGC-GCGCGCGCGCGCGCGC-GCGCGCGCGCGCGCGCGCGCGCG 48

Matrix = Unknown
Transitions / transversions = 1.00 (3/3)
Gap_init rate = 0.11 (5 / 46), avg. gap size = 1.00 (5 / 5)

 
 
 
 
 
  +      17   22.0  5.2  0.0  contig10_pilon   656953  657010 (1195669) +  (GGCCGC)n          Simple_repeat        1     61     (0)   174     
 
 ANNOTATION EVIDENCE: 
    17  21.98 5.17 0.00  contig10_pilon   656953  657010   1195669 +  (GGCCGC)n          Simple_repeat        1     61       0      
17 21.98 5.17 0.00 contig10_pilon 656953 657010 (1195669) (GGCCGC)n#Simple_repeat 1 61 (0) m_b695s252i2

  contig10_pilo     656953 GGCCGCGGCCGCGGCGGCCGCC-CGTCTG-GG-AGAGGTCGCGGACGAGG 656999
                                          v  v   -  v i -  -v v  i     v  v  
  (GGCCGC)n#Sim          1 GGCCGCGGCCGCGGCCGCGGCCGCGGCCGCGGCCGCGGCCGCGGCCGCGG 50

  contig10_pilo     657000 ACGAGGCCGCG 657010
                           v  v       
  (GGCCGC)n#Sim         51 CCGCGGCCGCG 61

Matrix = Unknown
Transitions / transversions = 0.22 (2/9)
Gap_init rate = 0.05 (3 / 57), avg. gap size = 1.00 (3 / 3)

 
 

 
 
   +      15   28.1  0.0  0.0  contig10_pilon   659053  659099 (1193580) +  (GGC)n             Simple_repeat        1     47     (0)   175     
 
 ANNOTATION EVIDENCE: 
    15  28.09 0.00 0.00  contig10_pilon   659053  659099   1193580 +  (GGC)n             Simple_repeat        1     47       0      
15 28.09 0.00 0.00 contig10_pilon 659053 659099 (1193580) (GGC)n#Simple_repeat 1 47 (0) m_b695s252i3

  contig10_pilo     659053 GGTGGCGGATGCGGCGGCGGGTGTGGGGGTGGAGGGTGCGGCGGCGG 659099
                             i     vv          vv i  v  i  v  vv          
  (GGC)n#Simple          1 GGCGGCGGCGGCGGCGGCGGCGGCGGCGGCGGCGGCGGCGGCGGCGG 47

Matrix = Unknown
Transitions / transversions = 0.38 (3/8)
Gap_init rate = 0.00 (0 / 46), avg. gap size = 0.0 (0 / 0)

 
 
 
 
 
  +      12   23.5  0.0  0.0  contig10_pilon   665463  665497 (1187182) +  (TCGACC)n          Simple_repeat        1     35     (0)   176     
 
 ANNOTATION EVIDENCE: 
    12  23.47 0.00 0.00  contig10_pilon   665463  665497   1187182 +  (TCGACC)n          Simple_repeat        1     35       0      
12 23.47 0.00 0.00 contig10_pilon 665463 665497 (1187182) (TCGACC)n#Simple_repeat 1 35 (0) m_b695s252i4

  contig10_pilo     665463 TCGAGGTCGTCGGCGAGCTCGACCTTGACCTCGAC 665497
                               vv   v vv   v        i         
  (TCGACC)n#Sim          1 TCGACCTCGACCTCGACCTCGACCTCGACCTCGAC 35

Matrix = Unknown
Transitions / transversions = 0.17 (1/6)
Gap_init rate = 0.00 (0 / 34), avg. gap size = 0.0 (0 / 0)

 
 

 
 
   +      15    8.8  0.0  0.0  contig10_pilon   669669  669692 (1182987) +  (TCG)n             Simple_repeat        1     24     (0)   177     
 
 ANNOTATION EVIDENCE: 
    15   8.85 0.00 0.00  contig10_pilon   669669  669692   1182987 +  (TCG)n             Simple_repeat        1     24       0      
15 8.85 0.00 0.00 contig10_pilon 669669 669692 (1182987) (TCG)n#Simple_repeat 1 24 (0) m_b695s252i5

  contig10_pilo     669669 TCGCCGGCGTCGTCGTCGTCGTCG 669692
                              i  v                 
  (TCG)n#Simple          1 TCGTCGTCGTCGTCGTCGTCGTCG 24

Matrix = Unknown
Transitions / transversions = 1.00 (1/1)
Gap_init rate = 0.00 (0 / 23), avg. gap size = 0.0 (0 / 0)

 
 
 
 
 
  +      14   16.3  0.0  5.6  contig10_pilon   670469  670506 (1182173) +  (GTAA)n            Simple_repeat        1     36     (0)   178     
 
 ANNOTATION EVIDENCE: 
    14  16.27 0.00 5.56  contig10_pilon   670469  670506   1182173 +  (GTAA)n            Simple_repeat        1     36       0      
14 16.27 0.00 5.56 contig10_pilon 670469 670506 (1182173) (GTAA)n#Simple_repeat 1 36 (0) m_b695s252i6

  contig10_pilo     670469 GTGAGTAAGTAAGTACATATGTAAGTAACTGTAACTAA 670506
                             i            vi  v        --    v   
  (GTAA)n#Simpl          1 GTAAGTAAGTAAGTAAGTAAGTAAGTAA--GTAAGTAA 36

Matrix = Unknown
Transitions / transversions = 0.67 (2/3)
Gap_init rate = 0.05 (2 / 37), avg. gap size = 1.00 (2 / 2)

 
 

 
 
   +      25    0.0  0.0  0.0  contig10_pilon   694394  694420 (1158259) +  (A)n               Simple_repeat        1     27     (0)   179     
 
 ANNOTATION EVIDENCE: 
    25   0.00 0.00 0.00  contig10_pilon   694394  694420   1158259 +  (A)n               Simple_repeat        1     27       0      
25 0.00 0.00 0.00 contig10_pilon 694394 694420 (1158259) (A)n#Simple_repeat 1 27 (0) m_b695s252i7

  contig10_pilo     694394 AAAAAAAAAAAAAAAAAAAAAAAAAAA 694420
                                                      
  (A)n#Simple_r          1 AAAAAAAAAAAAAAAAAAAAAAAAAAA 27

Matrix = Unknown
Transitions / transversions = 1.00 (0/0)
Gap_init rate = 0.00 (0 / 26), avg. gap size = 0.0 (0 / 0)

 
 
 
 
 
  +      12   23.5  0.0  0.0  contig10_pilon   694881  694915 (1157764) +  (TCGACC)n          Simple_repeat        1     35     (0)   180     
 
 ANNOTATION EVIDENCE: 
    12  23.47 0.00 0.00  contig10_pilon   694881  694915   1157764 +  (TCGACC)n          Simple_repeat        1     35       0      
12 23.47 0.00 0.00 contig10_pilon 694881 694915 (1157764) (TCGACC)n#Simple_repeat 1 35 (0) m_b695s252i8

  contig10_pilo     694881 TCGAGGTCGTCGGCGAGCTCGACCTTGACCTCGAC 694915
                               vv   v vv   v        i         
  (TCGACC)n#Sim          1 TCGACCTCGACCTCGACCTCGACCTCGACCTCGAC 35

Matrix = Unknown
Transitions / transversions = 0.17 (1/6)
Gap_init rate = 0.00 (0 / 34), avg. gap size = 0.0 (0 / 0)

 
 

 
 
   +     412   21.6 10.3  1.4  contig10_pilon   706219  706354 (1146325) C  rnd-4_family-1113  LTR/Gypsy          (0)  27733   27586   181     
 
 ANNOTATION EVIDENCE: 
   412  21.61 10.29 1.35  contig10_pilon   706219  706354   1146325 C  rnd-4_family-1113  LTR/Gypsy        27586  27733       0      
412 21.61 10.29 1.35 contig10_pilon 706219 706354 (1146325) C rnd-4_family-1113#LTR/Gypsy (0) 27733 27586 m_b696s001i0

  contig10_pilo     706219 TTACGGTCACGCTTAGTGAACGTTGTTTAACGGGGAGGTCCCCCATACGG 706268
                                                  vvv   vv      ii vv    v   
C rnd-4_family-      27733 TTACGGTCACGCTTAGTGAACGTGTATTATGGGGGAGACCGGCCATTCGG 27684

  contig10_pilo     706269 AGTATGCC--GCCGCTGTGTTTGAATTTAAGCGCCCGTTT-GTCAGCGAA 706315
                           ii v i  -- i  -- v i  v   v  i         i-      iv 
C rnd-4_family-      27683 GATTTACCCAGTCG--GAGCTTTAATATAGGCGCCCGTTCGGTCAGCACA 27636

  contig10_pilo     706316 ATC-----------GAGTCATTCTGAGTCACTTGCCATTTAAGCGCCCGT 706354
                           v  -----------         i      i    vvv            
C rnd-4_family-      27635 TTCACACACACACAGAGTCATTCCGAGTCATTTGCATATTAAGCGCCCGT 27586

Matrix = 20p53g.matrix
Kimura (with divCpGMod) = 23.68
Transitions / transversions = 0.71 (12/17)
Gap_init rate = 0.04 (5 / 135), avg. gap size = 3.20 (16 / 5)

 
 
 
 
 
  +   23421    9.2  1.6  0.5  contig10_pilon   706746  710230 (1142449) C  rnd-4_family-1113  LTR/Gypsy        (595)  27138   23613   182     
 
 ANNOTATION EVIDENCE: 
 23421   9.16 1.64 0.45  contig10_pilon   706746  710230   1142449 C  rnd-4_family-1113  LTR/Gypsy        23613  27138     595      
23421 9.16 1.64 0.45 contig10_pilon 706746 710230 (1142449) C rnd-4_family-1113#LTR/Gypsy (595) 27138 23613 m_b696s001i1

  contig10_pilo     706746 TGGTGGGAGGCTTTGAGTTATCGCTTCCGTGGAACACTCTATGAACTCGA 706795
                               i               v v    i        vv  v      i v
C rnd-4_family-      27138 TGGTAGGAGGCTTTGAGTTAACTCTTCTGTGGAACAAACTTTGAACTTGT 27089

  contig10_pilo     706796 ACTTCACATTCGCACACAATCAACACGATATTCAGTTGTTTTCTTCGATT 706845
                           -        iv     i   v  i  iii         v           
C rnd-4_family-      27088 -CTTCACATCGGCACATAATAAATACAGCATTCAGTTGGTTTCTTCGATT 27040

  contig10_pilo     706846 TAGAACGTGAAGCTTTACTGAAAAGCTTGCGTATCTTTCGATTGTCCCAC 706895
                            i              i  v       i v   v                
C rnd-4_family-      27039 TGGAACGTGAAGCTTTGCTTAAAAGCTCGGGTAACTTTCGATTGTCCCAC 26990

  contig10_pilo     706896 CGTCGAGTCTTGCCCTGCGTTCATGGGTCGCAAGCCAGTCCCCATCCCCA 706945
                                                          v                  
C rnd-4_family-      26989 CGTCGAGTCTTGCCCTGCGTTCATGGGTCGCCAGCCAGTCCCCATCCCCA 26940

  contig10_pilo     706946 AGACCGCCGCCCAGGCCGCGGCGCAGTACAAGTTCCCCGCCATCACGCAC 706995
                                                                             
C rnd-4_family-      26939 AGACCGCCGCCCAGGCCGCGGCGCAGTACAAGTTCCCCGCCATCACGCAC 26890

  contig10_pilo     706996 TTTGTTCGGCAGGATGTACCTGATGATTTTGTTTGCTGTGTTCATGATCT 707045
                                               i                             
C rnd-4_family-      26889 TTTGTTCGGCAGGATGTACCCGATGATTTTGTTTGCTGTGTTCATGATCT 26840

  contig10_pilo     707046 CGTCGCCGTCCACGATATTCTGGATGCTAACCGAGACACACTTCGCTGCG 707095
                                                                             
C rnd-4_family-      26839 CGTCGCCGTCCACGATATTCTGGATGCTAACCGAGACACACTTCGCTGCG 26790

  contig10_pilo     707096 TTGCGCGTCGGCCTGATGTCTGTCATATCGACAAGTATCCCAAAAGTCTA 707145
                            i        v   i                                   
C rnd-4_family-      26789 TCGCGCGTCGTCCTAATGTCTGTCATATCGACAAGTATCCCAAAAGTCTA 26740

  contig10_pilo     707146 TTTGTAAGCACCTTTTCTCCCACTGCTTGTTCGTCGCTGATAAGATCGTT 707195
                                             vi             iiv              
C rnd-4_family-      26739 TTTGTAAGCACCTTTTCTGTCACTGCTTGTTCGCTCCTGATAAGATCGTT 26690

  contig10_pilo     707196 AGCCTACGCTTGATACCAACGCTAACGGTGTCATATGTGCGTTCGTTCTC 707245
                                                                             
C rnd-4_family-      26689 AGCCTACGCTTGATACCAACGCTAACGGTGTCATATGTGCGTTCGTTCTC 26640

  contig10_pilo     707246 GGCGCGTACGTCCCGCTCGACGCCAATCTCCTTGATGCGGGCGACGCTCC 707295
                                     i                     i                 
C rnd-4_family-      26639 GGCGCGTACGCCCCGCTCGACGCCAATCTCCTCGATGCGGGCGACGCTCC 26590

  contig10_pilo     707296 CGACGCGGAAGCTCGCGCGTTCAGGTTTCTCGTTCGCGCATATGTTGTCT 707345
                           i                                                 
C rnd-4_family-      26589 TGACGCGGAAGCTCGCGCGTTCAGGTTTCTCGTTCGCGCATATGTTGTCT 26540

  contig10_pilo     707346 ATCGACTTGTCTCGGACGCGATGGCAGGTCGGTCAGCGGCAGTCAAGGGG 707395
                                               i                             
C rnd-4_family-      26539 ATCGACTTGTCTCGGACGCGGTGGCAGGTCGGTCAGCGGCAGTCAAGGGG 26490

  contig10_pilo     707396 GACTCGAAGCATCACTGGGCCGTTCGAACTTGCAGCATGCTAGAGCCCCT 707445
                                                         v                   
C rnd-4_family-      26489 GACTCGAAGCATCACTGGGCCGTTCGAACTAGCAGCATGCTAGAGCCCCT 26440

  contig10_pilo     707446 TCTCGCAGGTATTCACATTGACAAGAAATCGCACTTGTGGAATGCATCCT 707495
                                                                     i       
C rnd-4_family-      26439 TCTCGCAGGTATTCACATTGACAAGAAATCGCACTTGTGGAACGCATCCT 26390

  contig10_pilo     707496 TAGCAGTCGGCGGTGCCCTGGTCAGCATTCTCGGTGCTCGTCGTCTCCTA 707545
                                                                             
C rnd-4_family-      26389 TAGCAGTCGGCGGTGCCCTGGTCAGCATTCTCGGTGCTCGTCGTCTCCTA 26340

  contig10_pilo     707546 CCCACCGCTTTGGTCGACATTTTCGACGATATCTCGGGCTCTCTGTCGTA 707595
                              i                                              
C rnd-4_family-      26339 CCCGCCGCTTTGGTCGACATTTTCGACGATATCTCGGGCTCTCTGTCGTA 26290

  contig10_pilo     707596 TTACAAGTCCTTTGGCGAGGGGCCAGGCGTTCCTTCCCTCTTCGCTTGCG 707645
                                                                             
C rnd-4_family-      26289 TTACAAGTCCTTTGGCGAGGGGCCAGGCGTTCCTTCCCTCTTCGCTTGCG 26240

  contig10_pilo     707646 ACTTGTGTGCGTATGATCCGGACCATTTCAATGCGCGCATGAAGAAAAGA 707695
                                                                             
C rnd-4_family-      26239 ACTTGTGTGCGTATGATCCGGACCATTTCAATGCGCGCATGAAGAAAAGA 26190

  contig10_pilo     707696 GCTTGCCTCCGCTACACACGCGAGCGCTGTTGCCACGGTTGCTGGTGCCA 707745
                                                v                            
C rnd-4_family-      26189 GCTTGCCTCCGCTACACACGCCAGCGCTGTTGCCACGGTTGCTGGTGCCA 26140

  contig10_pilo     707746 GAACAAGCGGACAAGGATGTGTTTTGTGATCACCAATGGAGATGAGGGGG 707795
                                            i      i                         
C rnd-4_family-      26139 GAACAAGCGGACAAGGACGTGTTTCGTGATCACCAATGGAGATGAGGGGG 26090

  contig10_pilo     707796 CCGCGGAGGGGGGAGACGACGCGGGGGGCGAGGAGGAAGAAGAGGATGAG 707845
                                v           i                            i   
C rnd-4_family-      26089 CCGCGCAGGGGGGAGACAACGCGGGGGGCGAGGAGGAAGAAGAGGACGAG 26040

  contig10_pilo     707846 TTGGAATCTGAGGATGCTAACGATGACAACGAGGTGAGTACGCAATGGTC 707895
                                         v                        v          
C rnd-4_family-      26039 TTGGAATCTGAGGAAGCTAACGATGACAACGAGGTGAGTTCGCAATGGTC 25990

  contig10_pilo     707896 ATCGTATTATTTTATTTGACGTGTTCTCAGGTCTCAATTCCACCGCAGTC 707945
                                               i                    i        
C rnd-4_family-      25989 ATCGTATTATTTTATTTGACATGTTCTCAGGTCTCAATTCCGCCGCAGTC 25940

  contig10_pilo     707946 CAAGCGTAGGAAGGTGGCAGCTAATGCTGCTGTCGCTGGGCCTCGTCGCA 707995
                                                                v            
C rnd-4_family-      25939 CAAGCGTAGGAAGGTGGCAGCTAATGCTGCTGTCGCTCGGCCTCGTCGCA 25890

  contig10_pilo     707996 AGAAAGTCACTCGCGATGTCCCCCGTCGTTCCACGCGCCGATGCACAGTC 708045
                                                                   i   i     
C rnd-4_family-      25889 AGAAAGTCACTCGCGATGTCCCCCGTCGTTCCACGCGCCGGTGCGCAGTC 25840

  contig10_pilo     708046 AAAGGAGGGCCGGCGGCGCCTGTCGCAGTGACGTCGCAACCATCGCCCGG 708095
                                                       i                     
C rnd-4_family-      25839 AAAGGAGGGCCGGCGGCGCCTGTCGCAGCGACGTCGCAACCATCGCCCGG 25790

  contig10_pilo     708096 GTTAAACGCAGGGACGTCACAACTGCCTGCCGCCCCCGTCGCAGGCATGT 708145
                            i                   i               i            
C rnd-4_family-      25789 GCTAAACGCAGGGACGTCACAGCTGCCTGCCGCCCCCATCGCAGGCATGT 25740

  contig10_pilo     708146 CACAACCACCGCCCGCACCCAACGCAGGGACGCCGCATCGTCCAGCTGCA 708195
                                                    i       i     v          
C rnd-4_family-      25739 CACAACCACCGCCCGCACCCAACGCGGGGACGCTGCATCCTCCAGCTGCA 25690

  contig10_pilo     708196 GGCCAGTCGCAGGGTGGCAATGCTGGTCACGTACAGGCTCAGCCCCCTGT 708245
                                                                             
C rnd-4_family-      25689 GGCCAGTCGCAGGGTGGCAATGCTGGTCACGTACAGGCTCAGCCCCCTGT 25640

  contig10_pilo     708246 CGCAGGACGTCCTGCCCGGCGCCATGTCCACACCTATGTTCTCCTCGACG 708295
                           i              i                                  
C rnd-4_family-      25639 TGCAGGACGTCCTGCTCGGCGCCATGTCCACACCTATGTTCTCCTCGACG 25590

  contig10_pilo     708296 AGTTGCGTGGACTTCACGAGAAGGTCGACAAGATGGGGCGCAACCAGGAT 708345
                                  i                          v i             
C rnd-4_family-      25589 AGTTGCGCGGACTTCACGAGAAGGTCGACAAGATTGAGCGCAACCAGGAT 25540

  contig10_pilo     708346 GAGATTCTGTCCTTCTTCCGCGCTGGTTCTGGCGATGTTATGCGTCGCAC 708395
                                                                             
C rnd-4_family-      25539 GAGATTCTGTCCTTCTTCCGCGCTGGTTCTGGCGATGTTATGCGTCGCAC 25490

  contig10_pilo     708396 TCAGCGTTCGCGCGTCTCGCCCACCCCAGATGAGTAAAGTTCGTGTTTCT 708445
                                                                 i          i
C rnd-4_family-      25489 TCAGCGTTCGCGCGTCTCGCCCACCCCAGATGAGTAAAATTCGTGTTTCC 25440

  contig10_pilo     708446 TGATTTTCTTGGGATCATGTAGCTCTTTCGGCAATGGTCATGTATGCAGA 708495
                              i                                              
C rnd-4_family-      25439 TGACTTTCTTGGGATCATGTAGCTCTTTCGGCAATGGTCATGTATGCAGA 25390

  contig10_pilo     708496 GATTAGCTACCTTTCTGTGCTGCAATGAAGTTCCATTTCTTGGTCAACAT 708545
                                      v                                      
C rnd-4_family-      25389 GATTAGCTACCATTCTGTGCTGCAATGAAGTTCCATTTCTTGGTCAACAT 25340

  contig10_pilo     708546 ATCAACTGTACTCGTCAGTTTATAAGATCAGAGTGAAATCGAGCAGGTAG 708595
                                             i                               
C rnd-4_family-      25339 ATCAACTGTACTCGTCAGCTTATAAGATCAGAGTGAAATCGAGCAGGTAG 25290

  contig10_pilo     708596 CAAGTAGTTACACATCTCGCTCACAGCGTTTCCGAACAGCAGTGTTGAGA 708645
                                                                             
C rnd-4_family-      25289 CAAGTAGTTACACATCTCGCTCACAGCGTTTCCGAACAGCAGTGTTGAGA 25240

  contig10_pilo     708646 GCGAAACGGCGAGAGGTGCCGGTTTGTTATCTCGCACGCTGCTGACCATG 708695
                                           i                         i       
C rnd-4_family-      25239 GCGAAACGGCGAGAGGCGCCGGTTTGTTATCTCGCACGCTGCCGACCATG 25190

  contig10_pilo     708696 CATATATTACATCATATTCCGCTTCTTCTCTTCGCGCAACAACTTGCGCT 708745
                                                                i            
C rnd-4_family-      25189 CATATATTACATCATATTCCGCTTCTTCTCTTCGCGCGACAACTTGCGCT 25140

  contig10_pilo     708746 TTTTCCATGCTGCATATTCCGATTCCAGCTCTGTGCCTTTGACAGCCTCG 708795
                                                                             
C rnd-4_family-      25139 TTTTCCATGCTGCATATTCCGATTCCAGCTCTGTGCCTTTGACAGCCTCG 25090

  contig10_pilo     708796 GAATGGCGCTGTTCGCATTCCGGATGATTGTCCCAGTGTGTTTCCCTTGC 708845
                            v      i                                         
C rnd-4_family-      25089 GCATGGCGTTGTTCGCATTCCGGATGATTGTCCCAGTGTGTTTCCCTTGC 25040

  contig10_pilo     708846 CCTATTGCGCGTGTAAGATTTGCCGCAACCACCCAAACATTTTTTCCTCA 708895
                                                                            i
C rnd-4_family-      25039 CCTATTGCGCGTGTAAGATTTGCCGCAACCACCCAAACATTTTTTCCTCG 24990

  contig10_pilo     708896 TGGTCGGGCGCCTTTGGCGGATCTCTGTCTTCTGGCCCAGGCGTCCCTAA 708945
                            v  iv ii    i    v v v i    i    i   v   ii i  ii
C rnd-4_family-      24989 TCGTTCGATGCCTCTGGCCGCTGTTTGTCCTCTGACCCTGGCACCTCTGG 24940

  contig10_pilo     708946 AGATGAGAATCATGGGAGGAGGATGGGAGCGATACAGCGCTCACCCTTTG 708995
                                        i           ii   iv                  
C rnd-4_family-      24939 AGATGAGAATCATAGGAGGAGGATGAAAGCACTACAGCGCTCACCCTTTG 24890

  contig10_pilo     708996 CTTCGCAGCTTCTGACAGTGTGGCGGGCGGAATCGAGACTGTCCTGGGGT 709045
                                          vv i     i ii    iiii              
C rnd-4_family-      24889 CTTCGCAGCTTCTGAGCGCGTGGCAGATGGAACTAGGACTGTCCTGGGGT 24840

  contig10_pilo     709046 CCGAAAGGACAGTACTATTCGGGACTGCAGTGGTGTGATCCTTGCAGTGC 709095
                              v vi          i   iv  v           i    i   i  i
C rnd-4_family-      24839 CCGCATAGACAGTACTACTCGACACGGCAGTGGTGTGGTCCTCGCAATGT 24790

  contig10_pilo     709096 TTCTCCTCGACGTCTAGGGCTATGAGAGCGACAGGGACAGGGAGCCGGCG 709145
                                v v  v        v v    i   i    i v   i  i     
C rnd-4_family-      24789 TTCTCGTGGAGGTCTAGGGATCTGAGGGCGGCAGGAAGAGGAAGTCGGCG 24740

  contig10_pilo     709146 AGATAGGTGTGCGCGGGCCTGCAGCCTGATCGAGTTGCAGTGCGCTATGA 709195
                                  i    iv v i           v i    i     i   v   
C rnd-4_family-      24739 AGATAGGCGTGCAAGCGTCTGCAGCCTGAGCAAGTTACAGTGTGCTCTGA 24690

  contig10_pilo     709196 CGGGGTCCTCGTCCATGGGTTGGTAGGGGGGCCAGGACGTTGGGGTCGTG 709245
                            i                 i  i    v  i  iv           i i 
C rnd-4_family-      24689 CAGGGTCCTCGTCCATGGGCTGATAGGCGGACCGCGACGTTGGGGTTGCG 24640

  contig10_pilo     709246 TGAGACACGGCACGTACAAGCAAGGATTTAAATCACCTCATGAACTGGTG 709295
                           i   ivvv         i   i   ii        i        i     
C rnd-4_family-      24639 CGAGGGCGGGCACGTACGAGCGAGGGCTTAAATCATCTCATGAATTGGTG 24590

  contig10_pilo     709296 TGCAATGATCGGCATACAGATGAGGGAAAGTATTGGTTCGGATAATTGCT 709345
                                    i     v   i    v                 i    i i
C rnd-4_family-      24589 TGCAATGATTGGCATTCAGGTGAGCGAAAGTATTGGTTCGGACAATTACC 24540

  contig10_pilo     709346 GATGAACGTGGTACAGCAATTTGGTTGAGTGAGAGCGCATTTGTTTGCGC 709395
                             v     i i       i     ivvv       i     v   i    
C rnd-4_family-      24539 GAAGAACGCGATACAGCAGTTTGGCGCTGTGAGAGTGCATTGGTTCGCGC 24490

  contig10_pilo     709396 TTCGAATTTCGAAAGTTGAATCTGATCGTTTGGGAGCAACCCTCTCGCTT 709445
                             i      i    v    vv        v   ii  i  i   i    v
C rnd-4_family-      24489 TTTGAATTTTGAAATTTGACGCTGATCGTATGGAGGCGACTCTCCCGCTG 24440

  contig10_pilo     709446 TACCCTGAATTCAAACAAACTGCTGCGAGACATCGCTTCCATGAACACAC 709495
                                  i        i v ivvi  i  ?v   iv i   i iv i i 
C rnd-4_family-      24439 TACCCTGGATTCAAACGATCCTGCGCAAGNGATCAATCCCACGGCCGCGC 24390

  contig10_pilo     709496 TAATC-------------------------CACGCCTGGCTGCGACTT-T 709519
                            i   -------------------------     v vi  ii     - 
C rnd-4_family-      24389 TGATCCCTGCATGCGGAAGGCACGGTCTCGCACGCGTTACTATGACTTCT 24340

  contig10_pilo     709520 AACTTTGCATATCTGCGGGAATGATTCGAGGGTCAGCTACATTACATGTT 709569
                           v     vi  v ii i     v i     v         i   v      
C rnd-4_family-      24339 CACTTTTTATTTTCGTGGGAAAGGTTCGATGGTCAGCTATATTTCATGTT 24290

  contig10_pilo     709570 CTCATCTGCGTCACTCTCATTGTCCGTTTTGCGCAGAACCGTGCAGGTTG 709619
                                       v               i i   i            v  
C rnd-4_family-      24289 CTCATCTGCGTCCCTCTCATTGTCCGTTCTACGCGGAACCGTGCAGGATG 24240

  contig10_pilo     709620 TAAATAACAATTACAATTGACAGCCTGATTCCTTGCTCCGGTGCGCGTCT 709669
                            ---i     i     v      v      - v   iv v    i v   
C rnd-4_family-      24239 T---CAACAACTACAAATGACAGACTGATT-CGTGCCGCTGTGCACCTCT 24194

  contig10_pilo     709670 CTAGTAGAGAATAGACGAGTTGAAGCTCGCGTGGAAGACGCACGAGGGGC 709719
                                  i   v  v v              iv                 
C rnd-4_family-      24193 CTAGTAGGGAAGAGCCCAGTTGAAGCTCGCGCTGAAGACGCACGAGGGGC 24144

  contig10_pilo     709720 AGCGCGCCGGGAAGTCAGCCCACACTTGCCAGGCCGCACCAGAAGACATC 709769
                               i                          v  i               
C rnd-4_family-      24143 AGCGTGCCGGGAAGTCAGCCCACACTTGCCATGCTGCACCAGAAGACATC 24094

  contig10_pilo     709770 TTGTCGTTTGTCAGTGGTGGTTGCCAAAGATACGCAATAAATCGTACCTG 709819
                            i  i   i        i  i       v                     
C rnd-4_family-      24093 TCGTTGTTCGTCAGTGGCGGCTGCCAAATATACGCAATAAATCGTACCTG 24044

  contig10_pilo     709820 GGAACAGAATGCGCATCTACATGCGAGGGTGGGAGCCTGCCCACAGATTG 709869
                                                i           v i      i      i
C rnd-4_family-      24043 GGAACAGAATGCGCATCTACACGCGAGGGTGGGTGTCTGCCCGCAGATTA 23994

  contig10_pilo     709870 TCGATACACATTTGATGTAGCAGGTTGGCGACGATGAGAAGGGGGCTGAG 709919
                            i     i                          i        v      
C rnd-4_family-      23993 TTGATACGCATTTGATGTAGCAGGTTGGCGACGACGAGAAGGGCGCTGAG 23944

  contig10_pilo     709920 TGCTGTGGGGCATTAGCAGAGGCTGGATTGGAGCAGG-CAAAGCATACAC 709968
                                i                  vv    i i    -i           
C rnd-4_family-      23943 TGCTGCGGGGCATTAGCAGAGGCTTCATTGAAACAGGTTAAAGCATACAC 23894

  contig10_pilo     709969 CGCAATGGGCGAATCGAACGTAGCAACTACGGTGGGTGAGCGAGTACAGA 710018
                               ii v        i  i  i      v           i    vi  
C rnd-4_family-      23893 CGCAGCGCGCGAATCGGACATAACAACTAGGGTGGGTGAGCAAGTAGGGA 23844

  contig10_pilo     710019 GTGGTAAGGCACGTGAGACGCACAACAGAAATCTGCACATCGGCG---AC 710065
                            i v i   i              i   v  i           i ---  
C rnd-4_family-      23843 GCGCTGAGGTACGTGAGACGCACAGCAGCAACCTGCACATCGGTGTGCAC 23794

  contig10_pilo     710066 TAGGCGATTTGGATGCGACCGTGGAAGGGAAACTCCAGCCCATATAAATA 710115
                           v   v        v    i   ------        i  i     -  i 
C rnd-4_family-      23793 GAGGGGATTTGGAGGCGATCGT------GAAACTCCGGCTCATAT-AACA 23751

  contig10_pilo     710116 ATGTGG-CGAGACGTCCCCCAATCTGGGAGATTGCCAGTGAGC------- 710157
                            v  i -   i----i    i                   v  -------
C rnd-4_family-      23750 AGGTAGTCGAA----TCCCCGATCTGGGAGATTGCCAGTGCGCTTACTGC 23705

  contig10_pilo     710158 --------------GCG-----GTTCGCGATGCATCAGAGCCGCACTATC 710188
                           --------------   -----   i v ii                   
C rnd-4_family-      23704 AAATGACATTGTTTGCGCATATGTTTGGGGCGCATCAGAGCCGCACTATC 23655

  contig10_pilo     710189 GCGAGGGAGCCGCTGTCAGTGACACAATAATATTAACAGACG 710230
                             i    i i i  v    i     i v      vi i    
C rnd-4_family-      23654 GCAAGGGGGTCACTTTCAGCGACACGAAAATATTTGCGGACG 23613

Matrix = 20p53g.matrix
Kimura (with divCpGMod) = 8.18
Transitions / transversions = 1.84 (206/112)
Gap_init rate = 0.01 (24 / 3484), avg. gap size = 3.04 (73 / 24)

 
 

   +   39790   17.6  1.4  2.6  contig10_pilon   710316  718189 (1134490) C  rnd-4_family-1113  LTR/Gypsy       (4141)  23592   15807   182     
 
 ANNOTATION EVIDENCE: 
 39790  17.56 1.45 2.59  contig10_pilon   710316  718189   1134490 C  rnd-4_family-1113  LTR/Gypsy        15807  23592    4141      
39790 17.56 1.45 2.59 contig10_pilon 710316 718189 (1134490) C rnd-4_family-1113#LTR/Gypsy (4141) 23592 15807 m_b696s001i2

  contig10_pilo     710316 GAGCTGTGGTGACACGGGAACAAAGCGGATCTTACCGTGGATACGGGTTC 710365
                                  i                            i    v  ii    
C rnd-4_family-      23592 GAGCTGTAGTGACACGGGAACAAAGCGGATCTTACCATGGAGACAAGTTC 23543

  contig10_pilo     710366 CGAC--CGTGACTCGGACGTCTCGAGCTCGCTGAGCCGTATGTTGCTATC 710413
                               --i          i     i     ii         i     i   
C rnd-4_family-      23542 CGACGGTGTGACTCGGATGTCTCAAGCTCATTGAGCCGTACGTTGCCATC 23493

  contig10_pilo     710414 CGTAGTCCCACTTTAAGCTGCGGAATTTGCAGACAGTCATGGTCCATAGA 710463
                            i         v  ii           i    v      v    i   v 
C rnd-4_family-      23492 CATAGTCCCACATTGGGCTGCGGAATTCGCAGCCAGTCAGGGTCTATACA 23443

  contig10_pilo     710464 TAATCCGGGCATTGAGCTATCGGCATTGGGAACTCTGG---CATGTGGGT 710510
                                   vviiv   vi  i ?      v        ---  i i    
C rnd-4_family-      23442 TAATCCGGTGGCGGAGGCATTGNCATTGGCAACTCTGGAGCCACGCGGGT 23393

  contig10_pilo     710511 GGGACGAAGCAGCAGTGTCATGAATCCCGATTTGCCTGGCCCAGCAGGTT 710560
                               v v      i     i    iv  i                     
C rnd-4_family-      23392 GGGAGGTAGCAGCGGTGTCGTGAACACCAATTTGCCTGGCCCAGCAGGTT 23343

  contig10_pilo     710561 CGAAGCAGGCACGTTGATCAACACAGTGGAGGCCGCAGCACGCGAATGCG 710610
                           i        v      i     i    i  v v   ii  i       v 
C rnd-4_family-      23342 TGAAGCAGGAACGTTGGTCAACGCAGTAGACGGCGCGACATGCGAATGGG 23293

  contig10_pilo     710611 AGCGGATGTCACACATTTCTACTGTGTGGAGCAA-CACATCCCTGCTTCG 710659
                                                   i         -               
C rnd-4_family-      23292 AGCGGATGTCACACATTTCTACTGCGTGGAGCAAACACATCCCTGCTTCG 23243

  contig10_pilo     710660 AACTTGATAGGCACGCCGGACA-AGAAGTGTTTCAGACAGT-TTGCTTAA 710707
                              ?            -     -  v vv        i   -iiv ii  
C rnd-4_family-      23242 AACNTGATAGGCACGC-GGACATAGCACAGTTTCAGATAGTGCCTCCCAA 23194

  contig10_pilo     710708 TCTGGGCGAGGCGCCCATTGATATGAACCCATTGTCATGTTGAACACATG 710757
                           v      v  i             --         i v--    i ?   
C rnd-4_family-      23193 ACTGGGCCAGACGCCCATTGATAT--ACCCATTGTTAA--TGAATANATG 23148

  contig10_pilo     710758 AGCAGATAGGCGCTGGAAGGCACGGGGACCTTGCTGGAAATGTGGATGGA 710807
                                              i i                 i          
C rnd-4_family-      23147 AGCAGATAGGCGCTGGAAGACGCGGGGACCTTGCTGGAAGTGTGGATGGA 23098

  contig10_pilo     710808 CCGTGTGAGCGCAGCAGGAGATCATCGGCTATCACCTGTGT--GTGCAAA 710855
                                      v         v v            i    --    v i
C rnd-4_family-      23097 CCGTGTGAGCGAAGCAGGAGAGCTTCGGCTATCACCCGTGTCTGTGCCAG 23048

  contig10_pilo     710856 AACCACTCGTGCTTTGGTGCCGTGCAGCGTCGTCCTCTTGCTCCTGCCAC 710905
                           ivi    i i         ii     ------     ---     --   
C rnd-4_family-      23047 GCTCACTTGCGCTTTGGTGTTGTGCA------TCCTC---CTCCT--CAC 23009

  contig10_pilo     710906 GATGACATCCCAGACATTTCCGGGCATGGCCTTCGCCTTCGATCTCGCTC 710955
                                          i  i  v           i              v 
C rnd-4_family-      23008 GATGACATCCCAGACGTTCCCCGGCATGGCCTTTGCCTTCGATCTCGCAC 22959

  contig10_pilo     710956 CATTTGAAGTTATCCCAGCTTCTATTGGACTGCTGCTCAAGATGTCTCCG 711005
                            i  i        v      i i    v vi          v        
C rnd-4_family-      22958 CGTTCGAAGTTATACCAGCTCCCATTGCAACGCTGCTCAAGTTGTCTCCG 22909

  contig10_pilo     711006 GATGGCATTCCCCTGGCTTACACGGACGCTGGGCTCCTCGCCGCGATATG 711055
                             i  i  i  iv v                          v  i   i 
C rnd-4_family-      22908 GACGGTATCCCTGTCGCTTACACGGACGCTGGGCTCCTCGCAGCAATACG 22859

  contig10_pilo     711056 CGCGCATGACGCTTCTAAGCAACCCTCGGATATTAGCGATCTCAGTCAAA 711105
                                    v           i v            i    i i i  i 
C rnd-4_family-      22858 CGCGCATGAGGCTTCTAAGCAGCACTCGGATATTAGTGATCCCGGCCAGA 22809

  contig10_pilo     711106 GTCGCGCTACCGATCCTGTACAGCCGAGCCAGCAACCGGTACCCAGTAGT 711155
                             i                   v           ii     i        
C rnd-4_family-      22808 GTTGCGCTACCGATCCTGTACACCCGAGCCAGCAGTCGGTATCCAGTAGT 22759

  contig10_pilo     711156 GCGGACGTGGCTACATCAACTCCGTGCCTTTCCGTCGCACCCTCCCACGC 711205
                                  i  i       vi   ii i i   ivi v     v v   v 
C rnd-4_family-      22758 GCGGACGCGGTTACATCACTTCCACGTCCTTCTTCCTCACCCGCACACCC 22709

  contig10_pilo     711206 TGCGAACGACGTTTGCGCTCATCTTCTGCCATCAGAGGAACATCTCAGCC 711255
                                          v vi  iv iviv     i    vivi        
C rnd-4_family-      22708 TGCGAACGACGTTTGGGACCACATCGCCCCATCGGAGGCGAGTCTCAGCC 22659

  contig10_pilo     711256 AGCAGGGCCCTTGGCCGTTTGCGTGTGAGTTTCGTGGTGCATTCAGTTTC 711305
                                  i i  vvi ii             ii         v i   - 
C rnd-4_family-      22658 AGCAGGGTCTTTCCTCACTTGCGTGTGAGTTCTGTGGTGCATGCGGTT-C 22610

  contig10_pilo     711306 TCATCCCAGGAAGTGATGGATCATTCTAGAGTGTAGCCGCTCTTCAGGGC 711355
                              i      vi    i    i  v      i iiv  i          i
C rnd-4_family-      22609 TCACCCCAGGCGGTGACGGATTATACTAGAGCGCGTCCACTCTTCAGGGT 22560

  contig10_pilo     711356 TCTTTAAGGCGTCTCGGAGGTTGCGAATGATTGCGCTGTCAGTTTCATGT 711405
                            i i  i   i            i i   v   iii    i i    i v
C rnd-4_family-      22559 TTTCTAGGGCATCTCGGAGGTTGTGGATGCTTGTATTGTCGGCTTCACGA 22510

  contig10_pilo     711406 TCGCTGTTCACACGCGGGCAATGCT-GCAAAGAACGTTACGCAGAGCAAG 711454
                            v --    i i  ------     -  --------i i  v i i i -
C rnd-4_family-      22509 TGG--GTTCGCGCG------ATGCTTGC--------CTGCGGAAAACGA- 22477

  contig10_pilo     711455 CCTGTACATGCATGGTCGCTGAGGATCTGCAGCTGGGGTTCTTGTCTGAT 711504
                           --   ----   i     v  v  i vi  i  i  i     i     ii
C rnd-4_family-      22476 --TGT----GCACGGTCGGTGCGGGTACGCGGCCGGAGTTCTCGTCTGGC 22433

  contig10_pilo     711505 GAGCGTGCCGAAATATGTGTAATCGACGTGTGTAACCGACGAGCTGCCGT 711554
                               i  v      i             -------------  i   i  
C rnd-4_family-      22432 GAGCATGGCGAAATGTGTGTAATCGACG-------------AGTTGCTGT 22396

  contig10_pilo     711555 GAGCCAAGTTGACCAGAAAATGATTCTGGACTCGCGTTAGATTGCATTGA 711604
                            vvi        vi      i          i    ii i  v  i    
C rnd-4_family-      22395 GCCTCAAGTTGAATAGAAAACGATTCTGGACCCGCGCCAAATGGCGTTGA 22346

  contig10_pilo     711605 TTTCCACTAGGCTACGACCATGATGGTGGGATAATGAAACACTCGAAGTT 711654
                              v          i      i   iv       i   v iii      v
C rnd-4_family-      22345 TTTGCACTAGGCTATGACCATAATGAAGGGATAACGAATCGTCCGAAGTG 22296

  contig10_pilo     711655 GTTTATATGCCACGAACCGATCACCGGACCAGTCATAGTTCAATCTACTC 711704
                            vv   ivv  i      i        vv      v    i    i  i 
C rnd-4_family-      22295 GGGTATGGCCCGCGAACCAATCACCGGCACAGTCAAAGTTTAATCCACCC 22246

  contig10_pilo     711705 AAATCCTCATAAAGATCGGCTGTCGACGTGAGGTTGTTGTAAGTTCCCAT 711754
                           v  i           i           -      i       i i     
C rnd-4_family-      22245 CAACCCTCATAAAGACCGGCTGTCGAC-TGAGGTCGTTGTAAATCCCCAT 22197

  contig10_pilo     711755 CTCTGTTGACACAACAATCACTCTCACCGTTATCGTCGTGAATGTGTCAA 711804
                                i i  i      i ii     v i     vv v ii     i   
C rnd-4_family-      22196 CTCTGCTAACGCAACAACCGTTCTCAGCATTATCCACTTAGATGTGCCAA 22147

  contig10_pilo     711805 CGTGTGCGTCACGTTGAGAAGGCAGGCTGTCTTCTCCGCTGGAGAACCGG 711854
                                      i           i                       ?  
C rnd-4_family-      22146 CGTGTGCGTCATGTTGAGAAGGCGGGCTGTCTTCTCCGCTGGAGAACNGG 22097

  contig10_pilo     711855 GAAGGACGTGTTGGTGAGTCGCAGTACTGGAGATGGTTTAAGTATTGAAG 711904
                                           ?  i   i  vi      v   ii vii     i
C rnd-4_family-      22096 GAAGGACGTGTTGGTGNGTTGCAATAGCGGAGATTGTTCGACCGTTGAAA 22047

  contig10_pilo     711905 AAATAAGTAGCTGGACTGTGGGCGGTCAAATTGTGCATGGAGTCAGATGC 711954
                            v v   i                v  i     i          v  i  
C rnd-4_family-      22046 ATAAAAGCAGCTGGACTGTGGGCGCTCGAATTGCGCATGGAGTCTGACGC 21997

  contig10_pilo     711955 ACGACAAACGCGCGCACCGCTGCTTCTCCACGTGCGAGCAGAGCCTCATG 712004
                                v iv       i  i           i               vi 
C rnd-4_family-      21996 ACGACCAGAGCGCGCATCGTTGCTTCTCCACATGCGAGCAGAGCCTCTCG 21947

  contig10_pilo     712005 CAGCCCATCGAGCGAATCGTTAAGGTGTATCGTGAGGCCTGCTCCACCTG 712054
                              v       i  ii          v      i i      iv      
C rnd-4_family-      21946 CAGACCATCGAACGGGTCGTTAAGGTTTATCGTAAAGCCTGCCGCACCTG 21897

  contig10_pilo     712055 TCAGCGCAATGGACGGACGACCCATCTGCCACCATTTTTACGTGAGGACA 712104
                           i ii     i        i i   i i         vi           i
C rnd-4_family-      21896 CCGACGCAACGGACGGACAATCCACCCGCCACCATTGCTACGTGAGGACG 21847

  contig10_pilo     712105 AATGGAGCGAATCCGAGGATGGTCATGTCTGGAATTGCTAACTAGTGTT- 712153
                                  i   v vv     i        i    ii -------     -
C rnd-4_family-      21846 AATGGAGTGAAACACAGGATAGTCATGTCCGGAACCG-------GTGTTT 21804

  contig10_pilo     712154 GCTGCTGTACGGGGAGCGGGAATCCATATCAATCAGTTGCAAGGTGGCTT 712203
                            ii      v       i     i  i  v iv  vi   i     ii  
C rnd-4_family-      21803 GTCGCTGTAAGGGGAGCAGGAATTCACATAAGGCATCTGCGAGGTGATTT 21754

  contig10_pilo     712204 -GCTG----CACGTACACAGAATACGAAGACGAGCTGATACAT------- 712241
                           -i   ---- i     i        i  i      i v i   -------
C rnd-4_family-      21753 CACTGTGGGCGCGTACGCAGAATACAAAAACGAGCCGTTGCATCCTCACT 21704

  contig10_pilo     712242 --------------------GTGAATATGACGTCGCAGCAAGAGTGCGCT 712271
                           --------------------i      v    i   v   i   i v  i
C rnd-4_family-      21703 GCGAGANAATCGGACGCCATATGAATAAGACGCCGCCGCAGGAGCGGGCC 21654

  contig10_pilo     712272 GAGTCTACAAAAAAGCATGGCTTGGAAGTCAGTCCCCTGCATTCTGTGCA 712321
                             iii   i        i           iv   v -        v   i
C rnd-4_family-      21653 GAACTTACGAAAAAGCACGGCTTGGAAGTTCGTCAC-TGCATTCTCTGCG 21605

  contig10_pilo     712322 ATCGTAAACCAGCCATTGCACTTACCATTTTTTGAGCTTTGGACTCTGCG 712371
                           i v i  i  i i ii   v   v  v v v    vi   v  i      
C rnd-4_family-      21604 GTAGCAAGCCGGTCGCTGCCCTTCCCCTGTGTTGACTTTTCGATTCTGCG 21555

  contig10_pilo     712372 TACATACTCGCTCGAGCACAGGCGGACGCGGTAGAATTCGGTTATTGCGT 712421
                             i  i v i    vv i ii    v  i  v  i        i  ii  
C rnd-4_family-      21554 TATATGCACACTCGCCCGCGAGCGGTCGTGGGAGGATTCGGTTGTTATGT 21505

  contig10_pilo     712422 GTTATCTCCGA-------------GGGGCCGCAGC--------CTGACCT 712450
                            i         ------------- i      i i--------      v
C rnd-4_family-      21504 GCTATCTCCGAAAAACCACGCGTTGAGGCCGCGGTTCCGCCGTCTGACCG 21455

  contig10_pilo     712451 CTGCCGATTACCCACTCATGTCTGACGAGTAACGCGTCCTCAGATCACGG 712500
                                i v    v     i   i  i  i   vv   v    i i i   
C rnd-4_family-      21454 CTGCCAAGTACCAACTCACGTCCGATGAATAAACCGTACTCAAACCGCGG 21405

  contig10_pilo     712501 TCCTTTTTATTTTACAGCTTTACAAGTCTGCTGATCAAGAGGTCTCGTAC 712550
                            ----     --------------i  v v  i   i  -    vv  i 
C rnd-4_family-      21404 T----TTTAT--------------GGTATTCTAATCGAG-GGTCAGGTGC 21374

  contig10_pilo     712551 ACATTCGTGGTTTCGTCTCCATT--CTTTTCGGCTCCGG--------AGG 712590
                             v  i    i  i      i  -- vv     i     --------i  
C rnd-4_family-      21373 ACCTTTGTGGCTTTGTCTCCGTTTACGGTTCGGTTCCGGCGGCGGAGGGG 21324

  contig10_pilo     712591 ATTTCAAGGTCCGCTACACTCCTTGTGTC--CGTTTTACTATTGTCTGCC 712638
                           v              v  i  v    v i--  ivi    --      i 
C rnd-4_family-      21323 TTTTCAAGGTCCGCTCCATTCGTTGTCTTTACGCGCTACT--TGTCTGTC 21276

  contig10_pilo     712639 TGGCACCCACAT-ACTTATCGTCTCATTTGGAATCCCCGAGGCGCGCTTG 712687
                           v   ii v  v -v          vv   i   i  v   vi v v   v
C rnd-4_family-      21275 AGGCGTCAACCTCTCTTATCGTCTGCTTTAGAACCCGCGACACCCTCTTC 21226

  contig10_pilo     712688 GTCACAGACAGTGGCTTGAGCAGTTTACGAATTGTAGACCCGCTTGGTTG 712737
                             i i      i ii                               i i 
C rnd-4_family-      21225 GTTATAGACAGCGATTTGAGCAGTTTACGAATTGTAGACCCGCTTGATCG 21176

  contig10_pilo     712738 CTGCTCTGTGCCTTCGAGCGTCTATTTGACTTCGTGATAACGCGTCCAGC 712787
                                      v              i ii    vi   v         i
C rnd-4_family-      21175 CTGCTCTGTGCGTTCGAGCGTCTATTCGGTTTCGGAATACCGCGTCCAGT 21126

  contig10_pilo     712788 ATCCGACATGGTACGCGCTCGTTCATCGTCAAGAACCT-GAGCGCTGATG 712836
                            v          i   i     i i  v  i   i   -           
C rnd-4_family-      21125 AACCGACATGGTGCGCACTCGTCCGTCCTCGAGAGCCTCGAGCGCTGATG 21076

  contig10_pilo     712837 CTATCAGGCGAAGAATCACGCCGATGGCGGTCCAGATGATAGACGCGTGC 712886
                             i               ii  i i  i        i       i     
C rnd-4_family-      21075 CTGTCAGGCGAAGAATCATACCAACGGTGGTCCAGACGATAGACACGTGC 21026

  contig10_pilo     712887 ATAACCTGCGCGTCGCATATACAAAGTCGGTGTCTGTCACAAGGGCGGAG 712936
                                        i     i     i                        
C rnd-4_family-      21025 ATAACCTGCGCGTTGCATACACAAAATCGGTGTCTGTCACAAGGGCGGAG 20976

  contig10_pilo     712937 ATAGAACGGTGAATTGAATATTACACCTCCACTCTCTTAATGCTGATCCA 712986
                             vv   i      i           i    i  v    ii         
C rnd-4_family-      20975 ATTCAACAGTGAATCGAATATTACACTTCCATTCGCTTAGCGCTGATCCA 20926

  contig10_pilo     712987 GTTTTATTGCATAGTGCTCGGGAAAACATGTCTCGATTCGGCTTCGGGTC 713036
                           v         i         i    i                 v      
C rnd-4_family-      20925 TTTTTATTGCGTAGTGCTCGAGAAAGCATGTCTCGATTCGGCTACGGGTC 20876

  contig10_pilo     713037 ACATCGCTGGAGGCCTGGATGCACTCTTACGTTCGATATGCTTGTTGAGA 713086
                                          v  v        i  v     i     v  i    
C rnd-4_family-      20875 ACATCGCTGGAGGCCGGGCTGCACTCTCACCTTCGACATGCTGGTCGAGA 20826

  contig10_pilo     713087 TCAACCGCGTCCGTCTCATGGCTGGCTTGTCACCTCTTTCCGCGCGTGCG 713136
                                                          i         i    i   
C rnd-4_family-      20825 TCAACCGCGTCCGTCTCATGGCTGGCTTGTCGCCTCTTTCCACGCGCGCG 20776

  contig10_pilo     713137 GATGGGCTCATTGTCAGCGACAGTGAGCAGGACGATCTTACATTGCTCGC 713186
                                v    i     vi                  i i           
C rnd-4_family-      20775 GATGGTCTCACTGTCACTGACAGTGAGCAGGACGATTTCACATTGCTCGC 20726

  contig10_pilo     713187 ATTTCTCCACATGGAGAAAATGGTCCGTTTCTCGCGATCTACGACCGTTG 713236
                                                     i       v       v       
C rnd-4_family-      20725 ATTTCTCCACATGGAGAAAATGGTCCATTTCTCGAGATCTACTACCGTTG 20676

  contig10_pilo     713237 CTGATTCCAGGCTGTTGGACCTCCCTCTGACATTACGCCTTTCTCGGAGG 713286
                                  i      v    i           i     i            
C rnd-4_family-      20675 CTGATTCTAGGCTGGTGGATCTCCCTCTGACGTTACGTCTTTCTCGGAGG 20626

  contig10_pilo     713287 CAGGCGCTCCTGGCTCCTCGAGACAGATATATTAGTGACTTCGACGCCAA 713336
                              v            v        i    i i     i           
C rnd-4_family-      20625 CAGTCGCTCCTGGCTCATCGAGACAAATATGTCAGTGATTTCGACGCCAA 20576

  contig10_pilo     713337 TGCTGCTGAATGGCAGGACCCGACACGGCCGCAGTTGTTCGAATGTGTCC 713386
                                    i        i     i        i  i       v     
C rnd-4_family-      20575 TGCTGCTGAGTGGCAGGATCCGACGCGGCCGCAATTATTCGAATCTGTCC 20526

  contig10_pilo     713387 GGGAATCCCGTCATCTGCAGCGGTTCTTTCGCGTCTTCGAAGATCGTTTT 713436
                                                 ?     i  i                  
C rnd-4_family-      20525 GGGAATCCCGTCATCTGCAGCGNTTCTTCCGTGTCTTCGAAGATCGTTTT 20476

  contig10_pilo     713437 CTCGTCATCTCCAACACGCCCTTTGTGCCTAGGCTTCCGCCGAGCAGAAA 713486
                                      v                 i                    
C rnd-4_family-      20475 CTCGTCATCTCGAACACGCCCTTTGTGCCCAGGCTTCCGCCGAGCAGAAA 20426

  contig10_pilo     713487 GGCTCAAGCCCCTATTTATCTTGACGGGCGTCGCGGCTACTTCGAACATT 713536
                                i   i  i        i  i              i          
C rnd-4_family-      20425 GGCTCGAGCTCCCATTTATCTCGATGGGCGTCGCGGCTATTTCGAACATT 20376

  contig10_pilo     713537 CGCTCTATCCTCAGCTGCATCAGCCTGGTCTCTGGCATCACGCATTTATC 713586
                                                    i                        
C rnd-4_family-      20375 CGCTCTATCCTCAGCTGCATCAGCCCGGTCTCTGGCATCACGCATTTATC 20326

  contig10_pilo     713587 CCTGCGCTAAACGCCAGGCATTCTAATCTCGAGTCCGACTTTGCTTGGTA 713636
                                   i     v        ii          i              
C rnd-4_family-      20325 CCTGCGCTGAACGCGAGGCATTCCGATCTCGAGTCTGACTTTGCTTGGTA 20276

  contig10_pilo     713637 CGACCCAGATTTCGGTCGTCACTTTGTTGCTGTGGCTTCAACGGATTGCG 713686
                           i             i   i        iv             v   i i 
C rnd-4_family-      20275 TGACCCAGATTTCGATCGCCACTTTGTCTCTGTGGCTTCAACCGATCGTG 20226

  contig10_pilo     713687 AATTATACCGTGTTGGTGAGAAACTGCGCATGGTCATACTCGATGCACGG 713736
                               i           i  v            iv    v           
C rnd-4_family-      20225 AATTGTACCGTGTTGGCGACAAACTGCGCATGAACATAGTCGATGCACGG 20176

  contig10_pilo     713737 GTCTGGGCTCTGCGAGACTTCGGCTACCCCTCCCCAGAGCAATGGGACAC 713786
                                      v                 i     v           i  
C rnd-4_family-      20175 GTCTGGGCTCTTCGAGACTTCGGCTACCCTTCCCCCGAGCAATGGGATAC 20126

  contig10_pilo     713787 CCCGTACCTAGCCCGCGAGTCCGTTCACCATGCGACAACGCACCTGTGCA 713836
                                    i     v                    i  i          
C rnd-4_family-      20125 CCCGTACCTGGCCCGAGAGTCCGTTCACCATGCGACGACACACCTGTGCA 20076

  contig10_pilo     713837 ATCACATCCTGACTCGGGGTGAGATTGTGCGAACTTTGGTGGAACTACAA 713886
                                                                   v  i  i   
C rnd-4_family-      20075 ATCACATCCTGACTCGGGGTGAGATTGTGCGAACTTTGGTTGAGCTGCAA 20026

  contig10_pilo     713887 AGATCTATTCTGGAACTCCGAGGTTGGTCTGCGTACAACGCCGCGCAACG 713936
                                         i     v  v       v                  
C rnd-4_family-      20025 AGATCTATTCTGGAGCTCCGCGGGTGGTCTGAGTACAACGCCGCGCAACG 19976

  contig10_pilo     713937 CCGTCAACAGAACCTCACAACGAGACTGGGCTGCGATTGGCTGCATGTCC 713986
                                       v   i    i i                          
C rnd-4_family-      19975 CCGTCAACAGAAGCTCGCAACAAAACTGGGCTGCGATTGGCTGCATGTCC 19926

  contig10_pilo     713987 TAGTCCCAGCCGCGCAGGGTTTCGTCAACCCGGCATTCCGCGGCGTCTTT 714036
                               i              i                    i  i      
C rnd-4_family-      19925 TAGTTCCAGCCGCGCAGGGCTTCGTCAACCCGGCATTCCGTGGTGTCTTT 19876

  contig10_pilo     714037 GTTTCGGATTCGGAGACATGCAAAATATATGGCCATTTCGGTGTCCCGAC 714086
                              v                         i  i                 
C rnd-4_family-      19875 GTTGCGGATTCGGAGACATGCAAAATATACGGTCATTTCGGTGTCCCGAC 19826

  contig10_pilo     714087 CTGGTGGATTCGTCTTTTCTCAAAAGATCTCTTGCGCGAGGTTAGGAGCT 714136
                                                 i                          i
C rnd-4_family-      19825 CTGGTGGATTCGTCTTTTCTCAGAAGATCTCTTGCGCGAGGTTAGGAGCC 19776

  contig10_pilo     714137 GTGGCTATTCAGAAGTGGGCAACCACGATGCCGAGATCCCCCCTGATCTC 714186
                                           v        i              i  i      
C rnd-4_family-      19775 GTGGCTATTCAGAAGTCGGCAACCATGATGCCGAGATCCCTCCCGATCTC 19726

  contig10_pilo     714187 AGGCGCGCGGATGAGGTCGCCGCGCATGCCGATGGTTACCATAACTCTAT 714236
                             i        v       vi     v  i  i       i      i  
C rnd-4_family-      19725 AGACGCGCGGAGGAGGTCGATGCGCAAGCTGACGGTTACCGTAACTCCAT 19676

  contig10_pilo     714237 CTTACCGCCGCCAGAATTCTCATTCGGCGAGGATGATGAGACGGCTGCAT 714286
                            i    v              i                         i  
C rnd-4_family-      19675 CCTACCTCCGCCAGAATTCTCGTTCGGCGAGGATGATGAGACGGCTGTAT 19626

  contig10_pilo     714287 CTCATCTGGCATTGCATGTGGGCGGAGGCAAGTTGTTCAAGTTCTCAGCC 714336
                               i i   i    vv       i        i              i 
C rnd-4_family-      19625 CTCACCCGGCGTTGCCGGTGGGCGAAGGCAAGTCGTTCAAGTTCTCAGTC 19576

  contig10_pilo     714337 CAGTCTCAGCCATTGATGTAAAGCATTCAACAGGCGAAATAACCAGAGTT 714386
                                  ii  i         i  i  vv              iiv  ii
C rnd-4_family-      19575 CAGTCTCGACCGTTGATGTAAGGCGTTATACAGGCGAAATAACTGCAGCC 19526

  contig10_pilo     714387 GGCGAGGGTGATAGACTGCATTGTGATACCCCGGCGTCGACAGGCACTTC 714436
                                   i  v   v v   ---------------------        
C rnd-4_family-      19525 GGCGAGGGCGAAAGAGTTCAT---------------------GGCACTTC 19497

  contig10_pilo     714437 GGGCAAACGAGATGGTATGGTATAGCTGTCAGTTTGTCACGTGATCACTG 714486
                             v              vi   v                          v
C rnd-4_family-      19496 GGTCAAACGAGATGGTAAAGTAGAGCTGTCAGTTTGTCACGTGATCACTC 19447

  contig10_pilo     714487 ACGCAA-----ACAGCGCGCGCCGCCAATCTAAACGCCGACCGTGCAGCG 714531
                            i    -----i         ii            v     v i iv i 
C rnd-4_family-      19446 ATGCAACGCACGCAGCGCGCGTTGCCAATCTAAACTCCGACGGCGTCGTG 19397

  contig10_pilo     714532 TCAGGAAAACGGCCACGGTTATACAACAAGAATCACGCGTATCGCAGCAA 714581
                             v  i                  ii      i                 
C rnd-4_family-      19396 TCCGGGAAACGGCCACGGTTATACGGCAAGAACCACGCGTATCGCAGCAA 19347

  contig10_pilo     714582 CCGGCCTATTAAGCCCGTCTTGACTTCGGTTGCTTGGTTTCCTCGGCGTG 714631
                            v     i       i      i iv    ii          i     i 
C rnd-4_family-      19346 CAGGCCTGTTAAGCCTGTCTTGGCCGCGGTCACTTGGTTTCCCCGGCGCG 19297

  contig10_pilo     714632 CTTGCTGGGATTACGAGGACGGCAGTCAAATTTTGCCAGACGTAACCGAT 714681
                             ii                       i                      
C rnd-4_family-      19296 CTCACTGGGATTACGAGGACGGCAGTCGAATTTTGCCAGACGTAACCGAT 19247

  contig10_pilo     714682 GAACGTGCTGACGCTTTGTTCCACACTGCGGACTGTCCCATTCCTCGCAA 714731
                                   i     i         i   i   i     v           
C rnd-4_family-      19246 GAACGTGCCGACGCCTTGTTCCACGCTGTGGATTGTCCAATTCCTCGCAA 19197

  contig10_pilo     714732 CCTTCTTCAGCAGAAGCGTCCGCATAGGGTTCCGGTCGCCAGCTTCTTCG 714781
                           i  i                              i    v          
C rnd-4_family-      19196 TCTCCTTCAGCAGAAGCGTCCGCATAGGGTTCCGATCGCGAGCTTCTTCG 19147

  contig10_pilo     714782 CGAACGCAGACACAACGAATCTACCGTACATCGTCCATGTGTGGCTTACC 714831
                            i           ii       i     i   v i               
C rnd-4_family-      19146 CAAACGCAGACACGGCGAATCTGCCGTATATCCTTCATGTGTGGCTTACC 19097

  contig10_pilo     714832 ATTCGACCATATTATCTTCGGCTACTAGCCACTTCGTCGCTCTGGAGCAC 714881
                                i     i              i  i  i     v      v    
C rnd-4_family-      19096 ATTCGGCCATACTATCTTCGGCTACTGGCTACCTCGTCTCTCTGGTGCAC 19047

  contig10_pilo     714882 ACGTGGGTTTGACAGCACAACGTGGCGGGCCATCCTGAAAAGCCGCATCT 714931
                                 v           v  v     i     vi               
C rnd-4_family-      19046 ACGTGGCTTTGACAGCACCACCTGGCGAGCCATATTGAAAAGCCGCATCT 18997

  contig10_pilo     714932 CAAATGAAGCTGCTGAGGGCATGTTGCGCGATCTTGGAATGCTTAGCAAG 714981
                               i  i  i                       i              v
C rnd-4_family-      18996 CAAACGAGGCCGCTGAGGGCATGTTGCGCGATCTCGGAATGCTTAGCAAT 18947

  contig10_pilo     714982 CCTGGCCCTCAGGCGGCTGCTTCACCGTCTTCCGACGCTGCCGAGCAATC 715031
                           i i iv         v i i    i vi  i    i     i        
C rnd-4_family-      18946 TCCGAACCTCAGGCGCCCGTTTCATCCCCTCCCGATGCTGCTGAGCAATC 18897

  contig10_pilo     715032 CATTCCAGATCATGAAGTTTGCGGTGGCAGCGGCTTGGAGGTTGGCGATC 715081
                                     v            v     v  v        i        
C rnd-4_family-      18896 CATTCCAGATAATGAAGTTTGCGTTGGCACCGCCTTGGAGGCTGGCGATC 18847

  contig10_pilo     715082 CTTTCGTCGACGTTTTTGGACAGTATCCCACGCTGGTGGCAGATGTTGAG 715131
                                                i   i   i   i vi        i    
C rnd-4_family-      18846 CTTTCGTCGACGTTTTTGGACGGTACCCCGCGCCGTCGGCAGATGCTGAG 18797

  contig10_pilo     715132 ACAGCTAGGATCACATGCTTTGCATGTGATTATCTGGAATCCTGCGTCGA 715181
                                i      i    i                    i  v  i  i  
C rnd-4_family-      18796 ACAGCCAGGATCGCATGTTTTGCATGTGATTATCTGGAGTCATGTGTTGA 18747

  contig10_pilo     715182 TGGCGAACGCCCCTGTCGCTCAGAATCTGGACGTCTGGCTGTGGAGCCGG 715231
                           i     i                    i  v    iv     i       
C rnd-4_family-      18746 CGGCGAGCGCCCCTGTCGCTCAGAATCCGGCCGTCCTGCTGTAGAGCCGG 18697

  contig10_pilo     715232 TGGATTGGCATACCGTTGCCAAGCGGTACTTTCGCGAGGTACACAACGGA 715281
                           i   i           ivv                   v v i i v   
C rnd-4_family-      18696 CGGACTGGCATACCGTCCGCAAGCGGTACTTTCGCGAGCTCCGCGAGGGA 18647

  contig10_pilo     715282 GCCATGGAGGAATGTGCTCCGTTCGAGCACGGCAACCCTGGCAGTGACTG 715331
                            i                       v   i  v   ii            
C rnd-4_family-      18646 GTCATGGAGGAATGTGCTCCGTTCGCGCATGGGAACTTTGGCAGTGACTG 18597

  contig10_pilo     715332 CGCTATCTGGTTCGACTACGACAAGTCGCGCATTTTGATGTTTCGTGTCT 715381
                               i v    v   i           v  v                   
C rnd-4_family-      18596 CGCTGTGTGGTACGATTACGACAAGTCTCGGATTTTGATGTTTCGTGTCT 18547

  contig10_pilo     715382 GGAGTCGCTGCGACGTGCATGAAGATGTAACGCATGGAGAGTACCTTCTC 715431
                                 i                     v     i           i   
C rnd-4_family-      18546 GGAGTCACTGCGACGTGCATGAAGATGTCACGCACGGAGAGTACCTCCTC 18497

  contig10_pilo     715432 AGACCACTCGAGTTTCGCTCTACCACAGGCGAGTGGTATCCACCATCACG 715481
                                      i          iii      vi    i  v      i  
C rnd-4_family-      18496 AGACCACTCGAATTTCGCTCTATTGCAGGCGCATGGTGTCAACCATCGCG 18447

  contig10_pilo     715482 TCGGATATGGCCACGGGAACCGTCAGTGATACTTTGCATTGCATGCACCG 715531
                                                  v       vviv     ivi ---   
C rnd-4_family-      18446 TCGGATATGGCCACGGGAACCGTGAGTGATAAGCAGCATTAAGT---CCG 18400

  contig10_pilo     715532 ATGACGACTGACAGTTCACGTGATAGAGAGCGTACCGTGACGGCACCAGA 715581
                           vi   i   v         i      i  i      v    i i      
C rnd-4_family-      18399 CCGACAACTCACAGTTCACATGATAGGGAACGTACCCTGACAGTACCAGA 18350

  contig10_pilo     715582 GCAGGCATGCTCATTTGCGTACCCCAGCGATAAGCCGCAGATGGAGCCCA 715631
                              i  i     i  i     i  v v       v i   i v      i
C rnd-4_family-      18349 GCAAGCGTGCTCGTTCGCGTATCCAACCGATAAGGCACAGGTTGAGCCCG 18300

  contig10_pilo     715632 CGGACCTACCCTCACGCGATGGCGGCGCGCAGCCGCCAGGGAATAGCGCA 715681
                           i   i  iv          ---v            i v         ivi
C rnd-4_family-      18299 TGGATCTGACCTCACGCGA---AGGCGCGCAGCCGTCTGGGAATAGCAGG 18253

  contig10_pilo     715682 TGTTCTGTTTTCCGGGAATTCATATTGCGAACGGGACAACATCTGGATTT 715731
                                i                 v  i     v        i        
C rnd-4_family-      18252 TGTTCCGTTTTCCGGGAATTCATTTTACGAACCGGACAACACCTGGATTT 18203

  contig10_pilo     715732 CAACGGCCAGCGATTGCCGCGGTTTATTCCCGGTCCAGGCGCTGGTTCCT 715781
                                 v                 i            i    i   v   
C rnd-4_family-      18202 CAACGGGCAGCGATTGCCGCGGTTCATTCCCGGTCCAAGCGCCGGTGCCT 18153

  contig10_pilo     715782 GGCTGAGCGATCAGTGGCTATCGTACATCGTGTGGGAGTTGACGGAACTC 715831
                               v       v         v   i   i            v      
C rnd-4_family-      18152 GGCTCAGCGATCTGTGGCTATCTTACGTCGCGTGGGAGTTGACTGAACTC 18103

  contig10_pilo     715832 GAGTTCAGAAATGAACTGTTTGCGCTGGACGAACTACTCAGGGCACTGTA 715881
                                            v  i  ii    i     i        i     
C rnd-4_family-      18102 GAGTTCAGAAATGAACTCTTCGCATTGGATGAACTGCTCAGGGCGCTGTA 18053

  contig10_pilo     715882 TCCGTCACTGCGCGAACTGCATGCCATCCCGCCCCTGGAACGCCGATCTC 715931
                              i     v           i                            
C rnd-4_family-      18052 TCCATCACTCCGCGAACTGCACGCCATCCCGCCCCTGGAACGCCGATCTC 18003

  contig10_pilo     715932 AGCTGCTTGCTTGCTGGGGTGGCGGTGATTTGAAACCGTGCGAAGACGGT 715981
                                                       i                     
C rnd-4_family-      18002 AGCTGCTTGCTTGCTGGGGTGGCGGTGACTTGAAACCGTGCGAAGACGGT 17953

  contig10_pilo     715982 CCAACGCCACTTGCCGGCGACGGCGCGCGGGAGGACGTGTTTGCTTGCCT 716031
                             v     i               i i i              vv     
C rnd-4_family-      17952 CCCACGCCGCTTGCCGGCGACGGCACACAGGAGGACGTGTTTGGATGCCT 17903

  contig10_pilo     716032 GAAGGCTTTGTTCGCATTCATGTCTGTCTGGCCTCGCTCTCATCTGTTTC 716081
                           i v    i                i                  i      
C rnd-4_family-      17902 AATGGCTCTGTTCGCATTCATGTCCGTCTGGCCTCGCTCTCATTTGTTTC 17853

  contig10_pilo     716082 TCTCGGTCCCGGAGGGTGGATGGAGGTTGGAATCTATGGACGAGTTAGGA 716131
                                                             i               
C rnd-4_family-      17852 TCTCGGTCCCGGAGGGTGGATGGAGGTTGGAATCCATGGACGAGTTAGGA 17803

  contig10_pilo     716132 ATTGTTGTTTGGAAATTTTACGCTCAGACATATTTTGATTACCGACGCCA 716181
                           i             i           i                       
C rnd-4_family-      17802 GTTGTTGTTTGGAAGTTTTACGCTCAAACATATTTTGATTACCGACGCCA 17753

  contig10_pilo     716182 GTATGCCCCGCTGCCGTTCCTGCGACCTTCGGTGCCTTGGCATAGAATTG 716231
                                       v               v iii             iiii
C rnd-4_family-      17752 GTATGCCCCGCTCCCGTTCCTGCGACCTGCAACGCCTTGGCATAGAGCCA 17703

  contig10_pilo     716232 CTTTACCTTAGCCACCTGTAATCCGGACCGGACAAATTGTATGAACTGGT 716281
                           v i i vi           ---------------- i      -   v -
C rnd-4_family-      17702 GTCTGCGCTAGCCACCTGT----------------ACTGTATG-ACTCG- 17671

  contig10_pilo     716282 TTGCGCAGCAGTGTTTCAGGGCATGATGAATACATGCTATTACCAATTC- 716330
                           ----i  iv i   -------v i    v v    ivi v i v   ii-
C rnd-4_family-      17670 ----ACAAAAATGT-------AACGATGCAAACATAACAATGCGAATCTG 17632

  contig10_pilo     716331 --CAGCAGTGTAGTTA----AATGTCACGTGCCCGGACGTTGAGTCAAAC 716374
                           --i     v - i i ----  ivi i  i-  v i    i      iv 
C rnd-4_family-      17631 CGTAGCAGAG-AATCAGGGCAACTCCGCGC-CCGGAACGTCGAGTCAGCC 17584

  contig10_pilo     716375 TGAGTCAAGTGTATGCAGTGCGAGG--CACAGGTCCTAGAATCCACAGTG 716422
                                   vv  ii  v   v    --i    v   -    i iiiii  
C rnd-4_family-      17583 TGAGTCAACGGTGCGCCGTGGGAGGGCTACAGCTCC-AGAACCTGTGATG 17535

  contig10_pilo     716423 CCTCGAATTTCACGTGCTGTGCATAC----TCGT--GGTACAAAAACTGT 716466
                             ii i--- vv  i    i    i ----  v --   v     i i  
C rnd-4_family-      17534 CCCTGG---TGCCGCGCTGCGCATGCGATTTCTTCAGGTTCAAAAGCCGT 17488

  contig10_pilo     716467 TGGGGGATGGCACAACGCGGTTTTAGTCCAGTCCTTTGCCACGATGCCCG 716516
                           i    i-----i        v  i  i iiv  v             v v
C rnd-4_family-      17487 CGGGGA-----GCAACGCGGGTTCAGCCTGTTCGTTTGCCACGATGCGCC 17443

  contig10_pilo     716517 CCCGCCGGCCTCCAGTCATGCCACACGACGTTCAATGCCTGCTTCTGTCC 716566
                           v   i   v    v         i     i       i  v  i  vv i
C rnd-4_family-      17442 GCCGTCGGACTCCCGTCATGCCATACGACATTCAATGTCTCCTCCTCGCT 17393

  contig10_pilo     716567 TATCTCGGGACACCAAGCCTTCAGCGGTTTGCAAGGACAAATCGCACCCT 716616
                                 vv   i  i     i                 iv i   ivv  
C rnd-4_family-      17392 TATCTCCCGACGCCGAGCCTCCAGCGGTTTGCAAGGACGCACCGCGAACT 17343

  contig10_pilo     716617 GTATCGGCATTTCAAGGCTACCGAGCACCACCTTTACAACGTTCCCGCCA 716666
                                                i  i        i    i           
C rnd-4_family-      17342 GTATCGGCATTTCAAGGCTACTGAACACCACCTCTACAGCGTTCCCGCCA 17293

  contig10_pilo     716667 TAATCTCACAGTTTTTCGAGGACGTCAGCGGCTTCCGAGAGATGCAGAGC 716716
                                  i vv  i           vi                      v
C rnd-4_family-      17292 TAATCTCGCCCTTCTTCGAGGACGTGGGCGGCTTCCGAGAGATGCAGAGG 17243

  contig10_pilo     716717 AACACAGGGTTGCTCGTGTCTGGGTCGCAGGCTCTCCAGCTCTTCGCCCG 716766
                           v    i        i     i  i  v     v  i   i i     v  
C rnd-4_family-      17242 CACACGGGGTTGCTTGTGTCCGGATCCCAGGCGCTTCAGTTTTTCGCGCG 17193

  contig10_pilo     716767 TGTCCGTTACGCAGGGTCTGACATGGACCTCTGTGTTTCGCAGCGCTTTG 716816
                           i  i  v  i        i   ii                   ii   i 
C rnd-4_family-      17192 CGTTCGGTATGCAGGGTCCGACGCGGACCTCTGTGTTTCGCAGTACTTCG 17143

  contig10_pilo     716817 CTGCCATGGCTCTGCAGTGGATTTTGGATCACGGGTATCAGCTGCTCGAT 716866
                            i iv vi              iv       i     i            
C rnd-4_family-      17142 CCGTGAGAGCTCTGCAGTGGATCATGGATCATGGGTACCAGCTGCTCGAT 17093

  contig10_pilo     716867 CTCTCGCGGCCGAACGACGGTCTAGACGACGTATTCACGGTGCCGAGCCT 716916
                             ---       v vi  i    v   i i iv       ii   i    
C rnd-4_family-      17092 CT---GCGGCCGCAAAACAGTCTCGACAATGCTTTCACGGCACCGGGCCT 17046

  contig10_pilo     716917 TCGACATATGGCCATGCGTCATGCGTACAACTTGCGCTACGGTCAGCTGG 716966
                               v i           v  i         v      v       i   
C rnd-4_family-      17045 TCGAGACATGGCCATGCGACACGCGTACAACATGCGCTTCGGTCAGTTGG 16996

  contig10_pilo     716967 TGTTCCGTTTCGTAGGTGTCGATTCGCCT---CGACGCGTCGATCTCATC 717013
                            v  i   i i  v  i      i i   ---  i            v  
C rnd-4_family-      16995 TCTTTCGTCTTGTTGGCGTCGATCCACCTAGGCGGCGCGTCGATCTCCTC 16946

  contig10_pilo     717014 GTGTCGAGTGGGTGCGCTCTCACATCGGTTCTCGATTTTCACTCCAGTGC 717063
                                      v     i   v i     v     i     i  v     
C rnd-4_family-      16945 GTGTCGAGTGGCTGCGCCCTCTCGTCGGTGCTCGACTTTCATTCAAGTGC 16896

  contig10_pilo     717064 GTCATGTGTTCCCTTTCGTATTTCCCGTGGCTAACGATGTCTCCCAGCGG 717113
                               i i   vii v vvv i                i   i vi     
C rnd-4_family-      16895 GTCACGCGTTGTTTGTACGACTTCCCGTGGCTAACGACGTCCCATAGCGG 16846

  contig10_pilo     717114 CCGTTATGAACTTTATTGCGGCAGACTACGCCATTTCGCTCTTCCCGCAC 717163
                            i           i  i          i   v  i     i         
C rnd-4_family-      16845 CTGTTATGAACTTCATCGCGGCAGACTGCGCGATCTCGCTTTTCCCGCAC 16796

  contig10_pilo     717164 GCAACATTTGTCCAGGGTCTCTCACTGGTCCTGCGCAGCGATGATGAATA 717213
                                i   i    iv i        v  i     i            i 
C rnd-4_family-      16795 GCAACGTTTATCCAACGCCTCTCACTCGTTCTGCGTAGCGATGATGAACA 16746

  contig10_pilo     717214 TCGCACGAAGAAATGCGTCGAGAAGTACGACAAGAGGGGATTCACCCCGG 717263
                                                v       viv    i        i    
C rnd-4_family-      16745 TCGCACGAAGAAATGCGTCGACAAGTACGTTCAGAGAGGATTCACTCCGG 16696

  contig10_pilo     717264 TTTACTCTTCGCGGGCGTCGTTCGCATTTGCACCTGGTTTCAGGTGGATC 717313
                            v  i  v    i            i            ivii        
C rnd-4_family-      16695 TGTATTCGTCGCAGGCGTCGTTCGCGTTTGCACCTGGTCATGGGTGGATC 16646

  contig10_pilo     717314 CGCGACTCGATAAGCGACGCTGGGCTACAGCTTGCACGTGGTACACGGCG 717363
                             i     v ii        v  v i      i     i  i  i     
C rnd-4_family-      16645 CGTGACTCCACGAGCGACGCAGGTCCACAGCTCGCACGCGGCACGCGGCG 16596

  contig10_pilo     717364 CAATACTGCCATTCGGGCCCGCGCCTGCTGGATCCTCAGATTACCCAAAC 717413
                                 i              ii v         v    ii    ii v 
C rnd-4_family-      16595 CAATACCGCCATTCGGGCCCGTACGTGCTGGATCGTCAGGCTACCTGACC 16546

  contig10_pilo     717414 TGTCCGGGAAACCGCAGCCAAGGACCGTATCACGATCTTCGTGGTTCTTT 717463
                           i i v vi    i v     v    v     ivvi     vvv i   vi
C rnd-4_family-      16545 CGCCGGCAAAACTGGAGCCACGGACGGTATCGATGTCTTCCGTGCTCTAC 16496

  contig10_pilo     717464 GGAGGGTTCGAGGATAGGTCGCCCGGCGGGGGTTTAGAGCATTGGCATCG 717513
                             vvi iiivi vii v   i  i  i  v   i     i i    i   
C rnd-4_family-      16495 GGTCAGCCTTGGCGCATGTCACCTGGTGGCGGTCTAGAGTACTGGCGTCG 16446

  contig10_pilo     717514 GAATGGGTGGAGGCTCGTGGAGAGCGCCGAAGGATGCGAGATTTCTATCG 717563
                           i     i    i   i       v ivi         i       i  i 
C rnd-4_family-      16445 AAATGGATGGAAGCTTGTGGAGACCAGTGAAGGATGCAAGATTTCCATTG 16396

  contig10_pilo     717564 CGGGGCTGATCTGTAACCGAGGAGCTGAGTACAGCGTCCCCCCCGAGTTT 717613
                           v  viv       i i v       i                        
C rnd-4_family-      16395 AGGCAATGATCTGCAGCGGAGGAGCCGAGTACAGCGTCCCCCCCGAGTTT 16346

  contig10_pilo     717614 CACAGGTTCGTGATGAATCGGTTGTCCGAACTCGGCATTCGGGAAAGCTT 717663
                               vi  i i      i    v vvv   i  ii v iiv   i vi i
C rnd-4_family-      16345 CACACATTTGCGATGAACCGGTGGGGGGAATTCAACCTCTTGGAGATTTC 16296

  contig10_pilo     717664 CGGTCAAGATAACCGGTGAGTTTCTTTGCTGCGCATGTCGTCAAGGCGCT 717713
                           v   ivv iv  i i      ii  i  ii -i ii     i     i  
C rnd-4_family-      16295 GGGTTTCGGAAATCAGTGAGTCCCTCTGTCG-ACGCGTCGTTAAGGCACT 16247

  contig10_pilo     717714 GATCTAGTTGTTGCAGCTATGATGTATGGCAGCGACAGTTTATGCTCTTG 717763
                              v v  i i           i vi       i      i     ii  
C rnd-4_family-      16246 GATATCGTCGCTGCAGCTATGACGAGTGGCAGCAACAGTTCATGCTTCTG 16197

  contig10_pilo     717764 AATATTTGATTGCAGAGTGCATTTCGCGGCGCGA---------TACATTG 717804
                              i i     viv   i iv  i vi     v ---------    i  
C rnd-4_family-      16196 AATGTCTGATTTTTGAGCGTCTTCCCTGGCGCCACGGCCGATGTACACTG 16147

  contig10_pilo     717805 TAATATTTAATCAGTAGCAATTATTTGTAGGGGGTGCATCCCCAGGAAGA 717854
                              v       i   v  i  vi  --i         vi  v i     v
C rnd-4_family-      16146 TAAGATTTAATTAGTTGCGATAGTT--CAGGGGGTGCCCCCGCGGGAAGC 16099

  contig10_pilo     717855 TTGCTCTATTGTAATCCATCAGTTTAATCATGAAGACAGATGCTCGCGGA 717904
                            i              v i  v       ii  vviii ------  i  
C rnd-4_family-      16098 TCGCTCTATTGTAATCAACCATTTTAATCGCGACTGTGG------GCAGA 16055

  contig10_pilo     717905 AACGAGAGGCGAGCCGTCGATTGCAAGCTTCGGAATTCAAGCTTCGGACT 717954
                             iiv    viv  vi i             v vi   v i i i  i  
C rnd-4_family-      16054 AATATGAGGGACGCAATTGATTGCAAGCTTCCGCGTTCCAACCTTGGGCT 16005

  contig10_pilo     717955 TATGCTACCTCGCACGGTGGCATAATACCATAATGACATCGCTGACGAGA 718004
                            ivvvv      ii v i  i  -------iii    v i -   -----
C rnd-4_family-      16004 TGACAGACCTCGTGCTGCGGTAT-------CGGTGACTTTG-TGA----- 15968

  contig10_pilo     718005 GGCATCTTAGACAAGGGTGGTGCCAAGCCATGCTCGTGATG---ACGGAT 718051
                           -   -----------------      --    ii v v  ---  ivi 
C rnd-4_family-      15967 -GCA-----------------GCCAAG--ATGCCTGGGTTGCGAACATGT 15938

  contig10_pilo     718052 GCAATCCTGGATATCCCATATGTGAATGCTTCGATCTTCATTGCCCGATT 718101
                             i  iivv  iv v  i   v  i i     v    v  i v       
C rnd-4_family-      15937 GCGATTTGCGACTTGCCGTATCTGGACGCTTCCATCTACACTTCCCGATT 15888

  contig10_pilo     718102 TGGTGCAGATTTGCACTACTGCGGAGTACCTATGTTCACGGCATTTTATA 718151
                               iiii  ii   vii -------i  v  iii  i   v v  v   
C rnd-4_family-      15887 TGGTATGAATCCGCAGCGC-------CACATACACTCGCGGAAATTGATA 15845

  contig10_pilo     718152 TGCATCCTCCAAACAGTCGTTCATATACCTGCCTACGG 718189
                                vivi   i   ii  vii  i  i v  i    
C rnd-4_family-      15844 TGCATGTGTCAAGCAGCTGTATGTACACTTTCCCACGG 15807

Matrix = 20p53g.matrix
Kimura (with divCpGMod) = 16.78
Transitions / transversions = 1.93 (888/459)
Gap_init rate = 0.03 (229 / 7873), avg. gap size = 1.38 (316 / 229)

 
 
 
 
 
  +    5984   33.1  3.6  3.4  contig10_pilon   718665  722087 (1130592) C  rnd-4_family-1113  LTR/Gypsy      (13597)  14136   10709   183     
 
 ANNOTATION EVIDENCE: 
  5984  33.13 3.59 3.44  contig10_pilon   718665  722087   1130592 C  rnd-4_family-1113  LTR/Gypsy        10709  14136   13597      
5984 33.13 3.59 3.44 contig10_pilon 718665 722087 (1130592) C rnd-4_family-1113#LTR/Gypsy (13597) 14136 10709 m_b696s001i3

  contig10_pilo     718665 GTCAAGGTCCAT------GGTTCATCGGCAAGTTCCTTT--TTCGCCCTT 718706
                            i      v   ------   vv     i       v i--   v    i
C rnd-4_family-      14136 GCCAAGGTACATAATGCAGGTGGATCGGTAAGTTCCATCAGTTCCCCCTC 14087

  contig10_pilo     718707 CATTCTGCTCTCTGGCGTGTCGTGCTCTCCTATGCTACTTGCGCCCAACT 718756
                           iviii i     --  vvv   i i iiv v    vv  i     v    
C rnd-4_family-      14086 TTCCTTACTCTC--GCTACTCGCGTTTCGCAATGCGCCTCGCGCCGAACT 14039

  contig10_pilo     718757 TTTGTGTCTGCAATTTTGTCGATGGAACTGGCCGGCGGTCTCACCCGCAC 718806
                           v            i      ii   v i   v  i  i v v v  i  i
C rnd-4_family-      14038 ATTGTGTCTGCAACTTTGTCAGTGGCATTGGGCGACGATGTGAACCACAT 13989

  contig10_pilo     718807 AGAAGGTGCCAGCTGATGTGTGCGAAGGGAGATTCCTCGGTGAGTTCGCT 718856
                           v iv v        v   vvi  ii i  i    iv     ii   vv  
C rnd-4_family-      13988 CGGCGCTGCCAGCTCATGGTCGCAGAAGGGGATTTGTCGGTAGGTTGCCT 13939

  contig10_pilo     718857 GTAGGTGTATCGCCCTCC-TAACG---GTGCTGACGATTTGTGTAGCATC 718902
                           viii ii i    vi  i-iv   ---v v  v i  vvvvv    v   
C rnd-4_family-      13938 CCGAGCATGTCGCGTTCTGCCACGCATTTTCTCATGAGAGTGGTAGAATC 13889

  contig10_pilo     718903 GCGCGAGCATACAGCTCGTGTCCGCTCTGGAGTTTGCCAGCGATCTCTAC 718952
                               i     v   i  i i  i  vi      v         i      
C rnd-4_family-      13888 GCGCAAGCATTCAGTTCATATCTGCATTGGAGTATGCCAGCGACCTCTAC 13839

  contig10_pilo     718953 ACCGTCCCGGTCTGGACTGCTATCAACGACGCCCCGACATCGGGCTCCAT 719002
                           vvi        i        ii                vvi   v  v  
C rnd-4_family-      13838 CATGTCCCGGTTTGGACTGCCGTCAACGACGCCCCGACCGTGGGATCAAT 13789

  contig10_pilo     719003 GCTGATGCGATGTCTCGCTGTGGCTTTTTCATTGTGTGGAGCCCGTGCGC 719052
                            i  v       i  v     v  iivi  vi i  i  vi i      i
C rnd-4_family-      13788 GTTGCTGCGATGCCTGGCTGTCGCCCACTCTCTATGCGGCACTCGTGCGT 13739

  contig10_pilo     719053 ACGTCAAGACGTGGAAGTACGAGGTTCTCGCGATTCTGGTCAGTCGGGTG 719102
                           v   iiv        i  i               i     v   i  vi 
C rnd-4_family-      13738 TCGTTGCGACGTGGAGGTGCGAGGTTCTCGCGATCCTGGTGAGTTGGTCG 13689

  contig10_pilo     719103 GCATTCTCTCCAGCATTTCACATTTGACCGTAGATTGTCCAGAATATAGC 719152
                               vv-     - i  v i   i    vvivv   vv i       v  
C rnd-4_family-      13688 GCATGA-CTCCA-CGTTGCGCATCTGACACCCCATTCGCTAGAATATCGC 13641

  contig10_pilo     719153 CAAAATCATGTTGGAGGACGCCACAATGCCCTTCTTCCATCGTGTAATCC 719202
                           v      ii i v     iv v  ii v  v     i iv          
C rnd-4_family-      13640 AAAAATCGCGCTTGAGGATTCAACGGTTCCGTTCTTTCGGCGTGTAATCC 13591

  contig10_pilo     719203 AGCGCCTTGTGGTCATGTTCGTGCAACGGTTTTTCCCTGGTCTCTGGCAG 719252
                           i       vv          i v iv  v        vviiv  iii   
C rnd-4_family-      13590 GGCGCCTTCAGGTCATGTTCATTCGTCGTTTTTTCCCGTACATCCAACAG 13541

  contig10_pilo     719253 GCCGACGGGGAGCCCTACCTCTCGGGCGTGGAATGGATGTAAACGCGCAT 719302
                            v     i     i   i  v  v     v  i       i  iv  i i
C rnd-4_family-      13540 GACGACGAGGAGCTCTATCTGTCCGGCGTCGAGTGGATGTGAATTCGTAC 13491

  contig10_pilo     719303 AC-----CAAGTGCCCAA---GGATGCTT--TTTGGATGTTCCCGCATAT 719342
                             ----- vi ii  ii ---  i  iv --  iii    --- i vvi 
C rnd-4_family-      13490 ACTGTTGCTGGCACCTGATGCGGGTGTATCCTTCAAATGT---CACTAGT 13444

  contig10_pilo     719343 CAGTTACATCG--AGGTCGGGGTCCAGGCACGGGGAAGGCACGGTGCACC 719390
                            v v  v   i-- v v vv iii     i  i -   ivi iv  ii?i
C rnd-4_family-      13443 CCGGTAAATCATCATGGCTTGACTCAGGCGCGAG-AAGAAGCATTGTGNT 13395

  contig10_pilo     719391 ATATGGAGTGATGTCGGCGGTATTGCTCCGTCCGTGTTAGCAAGAATACA 719440
                                     vii     v i vii  i   iivv   i  i   v    
C rnd-4_family-      13394 ATATGGAGTGTCATCGGCTGCAACACTTCGTTTTGGTTGGCGAGATTACA 13345

  contig10_pilo     719441 CCTTTGTTCGAATTCGGCGTACACAGTTAATTTACAGCTTGTGAGAGTTT 719490
                               i    ?v  v  i-- v  iv    v  i ivii i i  v-    
C rnd-4_family-      13344 CCTTCGTTCNCATACGA--TCCATTGTTATTTCATCATTCGCGAC-GTTT 13298

  contig10_pilo     719491 ATCCGGATTTCGGTTGTCGTACTATAAAGTCGAGCTAGTGGACAACACGT 719540
                           i i v vi  i     i iii       vii i     i ii v ii v 
C rnd-4_family-      13297 GTTCCGCCTTTGGTTGCCACGCTATAAATCTGGGCTAGCGAGCTATGCTT 13248

  contig10_pilo     719541 G-GTTCATC-----TTCTATA--TTTCGGAGTGAGTTCTTCACTGCCCGA 719582
                            -iiv i  ----- i   v -- i  ivvi  vivv ii i i   ii 
C rnd-4_family-      13247 GTACACGTCGAGGTTCCTAGAGCTCTCATTATGCAGACCCCGCCGCCTAA 13198

  contig10_pilo     719583 CCTCCCAGGGCAGCCGGTGTGC-GTGATGTGTAGCTG-------CAGGTT 719624
                             v  i v    v  v i    -  v  -  v  i v------- i  i 
C rnd-4_family-      13197 CCACCTATGGCATCCTGCGTGCAGTCAT-TGGAGTTCAGGGACCCGGGCT 13149

  contig10_pilo     719625 ATTGATATGCAGGCGGGGTCACATCTTCGAAAGATGCTCACATGATGAAT 719674
                             iiii    ii  v ii -i  iv   v  viv         v ii  i
C rnd-4_family-      13148 ATCAGCATGCGAGCCGAAT-GCACGTTCTAACACTGCTCACATCACAAAC 13100

  contig10_pilo     719675 GAATCAAAGCCTCATGGCCTCGCGTTGGCAAGCGCTTACTGGCAGCCGGC 719724
                           iv  ii    - vii v     i v vi  i          v        
C rnd-4_family-      13099 ATATTGAAGC-TAGCGTCCTCGTGGTCACAGGCGCTTACTGCCAGCCGGC 13051

  contig10_pilo     719725 GCAGCA-GGTGGCGCTATTTGTTGATAAGCGGAGAGTTCACAGGCGAGGC 719773
                                 -            ii      v            i  v     v
C rnd-4_family-      13050 GCAGCAAGGTGGCGCTATTCATTGATATGCGGAGAGTTCATAGTCGAGGA 13001

  contig10_pilo     719774 TGCATCAGACACGGCG-ACCAGCCAGATCTGGATTGCATTGCTT-GCGCG 719821
                           i        v    v - v   i                i    - v v 
C rnd-4_family-      13000 CGCATCAGAAACGGAGCAACAGTCAGATCTGGATTGCATCGCTTAGGGAG 12951

  contig10_pilo     719822 GT-GTTTGTGTGAGCTCTCAGCGCATTTATGAATTGCTCACTGCGCTGTG 719870
                             -   i i          i      iv         ii        i  
C rnd-4_family-      12950 GTTGTTCGCGTGAGCTCTCGGCGCATCAATGAATTGCCTACTGCGCTATG 12901

  contig10_pilo     719871 GGGGCTGCCAGTAGAACGGCTGTTACCGCGAGGCAAACAGCGCCGGTGGC 719920
                           v v    i                  i  -   v v  i i ivi i  i
C rnd-4_family-      12900 CGTGCTGTCAGTAGAACGGCTGTTACTGC-AGGGACACGGTGTGAGCGGT 12852

  contig10_pilo     719921 GATAGTCATGGTCGCACAAAGAAAGGTGGGCCGGTGCGGACATTGGAATG 719970
                            i iv  vv  v  iv i i        v  -  i i i v  i    i 
C rnd-4_family-      12851 GGTGTTCCAGGGCGTTCGAGGAAAGGTGCGC-GGCGTGAAGATCGGAACG 12803

  contig10_pilo     719971 GCATCA-GTCGCG---AGTTTTTAATACTTTTCCCACGGTCGCATGTGGT 720016
                            i-   - i i  ---  v  v   i v   i    vv i ii   iv i
C rnd-4_family-      12802 GT-TCACGCCACGCGCAGGTTATAACAATTTCCCCAACGCCATATGCTGC 12754

  contig10_pilo     720017 C-TTCTCGACGATCGGTGGTAGG----TCAGATCAGGACAGGTGCTAATC 720061
                            -   ii        ii  iiv ----    i       iv         
C rnd-4_family-      12753 CATTCCTGACGATCGACGGCGTGCTGCTCAGGTCAGGACGTGTGCTAATC 12704

  contig10_pilo     720062 -CGCATGCATTTAGTATTACCGTTTTGCCCCATTTGTGCCACAGGGTGTT 720110
                           - i  i    vvi i ii  i v  i  v ii    i   v     vv i
C rnd-4_family-      12703 ACACACGCATGGGGCACCACTGATTCGCGCTGTTTGCGCCCCAGGGACTC 12654

  contig10_pilo     720111 TGGTCTCTGCGTTCAGCTGATGATGTCAAATAGTCGTCGCCGGCGGAGTT 720160
                               i  vv viv  viv  i vvvi    i v i   iii -  ---- 
C rnd-4_family-      12653 TGGTTTCATCCCACATTGGACGCACCCAAACATTTGTCATTG-CG----T 12609

  contig10_pilo     720161 GTTGTGTGACTCCAATGTT-CATACTCAGGTGGCCCGCGGGCCGGCATTG 720209
                            iv  i --   iiv i i- iv v  i  ivi i iiv   v  vv ii
C rnd-4_family-      12608 GCGGTAT--CTCTGCTATCACGAAATCGGGCCACTCATTGGCGGGGCTCA 12561

  contig10_pilo     720210 GCTTCCATAGCCTTTTATTCAGTGCAGCAGGTCGGATCCCGCGGTCCTGT 720259
                            vii iii   i  v      i    i   i  i     ? -------- 
C rnd-4_family-      12560 GGCCCTGCAGCTTTATATTCAATGCAACAGATCAGATCCNG--------T 12519

  contig10_pilo     720260 C-CGTTCGACAAGTGCATCTTTG--GGCTGCCTTGGGCTGTCAGGTCTAG 720306
                            -    - i vi   i   ii  -- vi    v  v i vvv v  ? v 
C rnd-4_family-      12518 CACGTT-GGCTGGTGTATCCCTGCAGCTTGCCATGTGTTCAAATGTNTTG 12470

  contig10_pilo     720307 TGAGACGTTCCCGTTCTTGCTCGCCG---ATCTCCGACCTGCTGACCTCA 720353
                                   i  ---   iiii   i --- vi  vv v i     i   -
C rnd-4_family-      12469 TGAGACGTCCC---TCTCATCCGCTGCAAAGTTCACAACCGCTGATCTC- 12424

  contig10_pilo     720354 CCGCATTT----AGCGATAATACAATCTTAGACACCTGCGGCGTATGGTT 720399
                           ---     ----      viv  i  i i  viiv    viv   ii   
C rnd-4_family-      12423 ---CATTTCCTCAGCGATCGGACGATTTCAGCTGACTGCCAGGTACAGTT 12377

  contig10_pilo     720400 TGTGACGCGTGTCGT-GCAATGGATCGCTCATACATAACATTCCAGCACT 720448
                           v  vii ivi i   -   ---    ii   i   ii  v ii     i 
C rnd-4_family-      12376 GGTTGTGTTCGCCGTCGCA---GATCATTCACACACGACCTCTCAGCATT 12330

  contig10_pilo     720449 ACGAAAATCGCAAATCGCAGAAATTTGCACTGTG-CGAAATCAACGTGTT 720497
                            i   i iv  i v  v iv    vi - i    - i   v  -    i 
C rnd-4_family-      12329 ATGAAGACAGCGATTCTCGTAAATACG-ATTGTGGCAAAAGCA-CGTGCT 12282

  contig10_pilo     720498 GGGCAATTTTATTCTACCTATGGCCTCGCAT-TGATGTCATTGGAACGTA 720546
                               v v i v  v   vv v v   vv i -iiii ivii     v  i
C rnd-4_family-      12281 GGGCCAGTCTCTTGTACGAAGGTCCTACCGTACAGCGCAGCTGGAAGGTG 12232

  contig10_pilo     720547 TGTCGTGCGGGAACCCA----CGACTTTACGACTATAACTGACATGGCGT 720592
                           i    i i v    v v----  i    v    v  vv    iv v  v 
C rnd-4_family-      12231 CGTCGCGTGTGAACGCTTTATCGGCTTTCCGACGATCTCTGATTTTGCCT 12182

  contig10_pilo     720593 CACGTCTCTCACAGGAGCTCACGAGGAAGTCCATCAACGAAGGAGCGCGA 720642
                            vi       v     i  vv  vv  ii v         i    v vv 
C rnd-4_family-      12181 CCTGTCTCTCCCAGGAACTACCGCCGAGATACATCAACGAGGGAGAGATA 12132

  contig10_pilo     720643 ACAAGCGCCCT----ATGTTTTGAGTGTTCGAACGCATCGCATCGGTGAC 720688
                            vi ii v   ----        vvi     vi   vi ----   i   
C rnd-4_family-      12131 AGGAATGGCCTGTGCATGTTTTGTTCGTTCGTGCGCTCC----CGGCGAC 12086

  contig10_pilo     720689 AGTGCAG-GGTTGTGTCTACATTCCTGGACCTGCGGGC-ATCCAATCCAG 720736
                             v  v -   v i   v viv ii  i   - ii v -i ii   iv  
C rnd-4_family-      12085 AGGGCCGTGGTGGCGTCAAAGGTTTTGAACC-GTAGCCTGTTTAATTGAG 12037

  contig10_pilo     720737 ATTGTGACTCAATCGAATTCTTATGGGTGGTGAACTGGGGTGGCAGTTTG 720786
                               ii i      ---i v iii vvii                  i  
C rnd-4_family-      12036 ATTGCAATTCAATC---CTGTCGCGCTCAGTGAACTGGGGTGGCAGTCTG 11990

  contig10_pilo     720787 TATAATGCGACCGTCGGAATGACCCTGTCGTAAATGTTCCGCTTCGATTG 720836
                           v vi i  vi    i   v         ivi v i  ---------  v 
C rnd-4_family-      11989 GAAGACGCTGCCGTTGGACTGACCCTGTTCCATACGT---------ATGG 11949

  contig10_pilo     720837 TCA-GTCGATGATTGGTCAATTTAAGCAGAACCTGAAACGAAGTCACCGC 720885
                           i  -   --i      iiv    iv vi   iv    iv     ii   i
C rnd-4_family-      11948 CCATGTC--CGATTGGCTCATTTGCGAGGAATGTGAAGAGAAGTTGCCGT 11901

  contig10_pilo     720886 ACGTTGCAATGAGCGCCCCGTAGAGCCCCCTGTCCAACGCAGTTTGACTG 720935
                           i i i  i   v     iivi v      iv iviiviv iv    v i 
C rnd-4_family-      11900 GCATCGCGATGCGCGCCTTTCATAGCCCCTAGCATGCTCCGCTTTGCCCG 11851

  contig10_pilo     720936 TCGACTTTGTCGGACGGAACTTTATTCAGAAGGATAGAAAACCCCTGCGC 720985
                            i  viviiv     v    i    v i  ii    v    i i v i  
C rnd-4_family-      11850 TTGAACACAGCGGACTGAACCTTATGCGGAGAGATATAAAATCTCAGTGC 11801

  contig10_pilo     720986 ACGTCGAGGCACCTGCA------TCAGAGTACCCCCCATTTTACAGTCTG 721029
                             i  v v i i i   ------ v   v i i i   ------ v    
C rnd-4_family-      11800 ACATCCACGTATCCGCACTGGATTGAGATTGCTCTCCA------ATTCTG 11757

  contig10_pilo     721030 CCACGTGTGTTCGATATGTGCCGTGATCTCACCGTTTGGTGTGAACGTGC 721079
                           i-  ii i i iv i        v      i i      vvi    iv  
C rnd-4_family-      11756 T-ACACGCGCTTTACATGTGCCGGGATCTCGCTGTTTGGACCGAACAAGC 11708

  contig10_pilo     721080 TTGCACGCATCGGGAGGTAACGGCGAGACAGAAGGTGCGATTTTCGTCTC 721129
                           v   iv      v v vvi viiii           ii i i iiv  i 
C rnd-4_family-      11707 ATGCGGGCATCGCGTGCAGAAAATAAGACAGAAGGTATGGTCTCTCTCCC 11658

  contig10_pilo     721130 ACTGTAAGCCTTGACCATGATTCTCACGCACAGCGCAGCTTGATTGCAAG 721179
                           - i  i   i   iv?ivi          ivi v i    i  i  i  i
C rnd-4_family-      11657 -CCGTGAGCTTTGGGNGAAATTCTCACGCGAGGAGTAGCTCGACTGTAAA 11609

  contig10_pilo     721180 TTGGACCACTGCGTCTTTAGCTCTTTTCACCCCAATCAAGGGAAGCACTC 721229
                           iv vii ii     i  i   v       i  vi v iv          i
C rnd-4_family-      11608 CAGTGTCGTTGCGTTTTCAGCGCTTTTCATCCAGAGCGCGGGAAGCACTT 11559

  contig10_pilo     721230 GTGCGAGGCGTCATGTCGGCATCAGTTCGTCATGACGTTGGTGGTCGCGC 721279
                               i? v v  i         iv      vvi vi  v  iiiiivii 
C rnd-4_family-      11558 GTGCANGTCTTCGTGTCGGCATTCGTTCGTATCGCTGTGGGCAACTCTAC 11509

  contig10_pilo     721280 GACTGAGATGCTGACGTCTTTGCCAGACCCGATAAGATCGAAAAGCTCGA 721329
                             i vvviiv    i vv i vi   i     i i   v      i v  
C rnd-4_family-      11508 GATTTCCGCCCTGATGAATCTTTCAGGCCCGACAGGATAGAAAAGTTGGA 11459

  contig10_pilo     721330 CATCGTGAATGAGAAATCCACTTATACTTGCCTGGCCTGCGGTGCACTTG 721379
                              i     i           v    vi   v vv       i   vvi 
C rnd-4_family-      11458 CATTGTGAACGAGAAATCCACGTATAACTGCATCCCCTGCGGCGCAAACG 11409

  contig10_pilo     721380 ATATGGGTTCGCCGTCGAAGACCAATATTCCAAATGTACCCGTCACGCGC 721429
                              i   i           i   ivi     v  i  v  i     vivi
C rnd-4_family-      11408 ATACGGGCTCGCCGTCGAAAACCGTCATTCCCAACGTCCCTGTCACTTCT 11359

  contig10_pilo     721430 ATTTCGCCGGTGTTGGGCCTGCATCGTGTTTAGCTGTGAATGGATCTGTT 721479
                            vvi       i v   -v iii i  vi ii  iv vivi v  v  ii
C rnd-4_family-      11358 AGGCCGCCGGTATAGGG-ATATGTTGTCCTCGGCCTTCGCCGTATATGCC 11310

  contig10_pilo     721480 CGGACAGTGCCCGTTAACTTGCATGGACGCCGACCGCGCCACGCACGTGG 721529
                           vv ii      ivvv v    i  ivii ii v ii    i v i---i 
C rnd-4_family-      11309 GTGGTAGTGCCTCGAACCTTGTATATGTGTTGTCTACGCCGCTCG---AG 11263

  contig10_pilo     721530 AGATGCTGTGT-CTGCC------TGAAAC----TATCGCTGAAGGATACG 721568
                                i  i i- i v ------i    i----i  ii iiv vv  i- 
C rnd-4_family-      11262 AGATGTTGCGCGCCGACGATGAACGAAATCAGACATTACCATATCATG-G 11214

  contig10_pilo     721569 ATCAGTCGTGTG---TCACAGCCACCATCGATCTGCCGATCTT-CCTGGC 721614
                              - i  vv  ---           i              ii-   --i
C rnd-4_family-      11213 ATC-GCCGGTTGACCTCACAGCCACCGTCGATCTGCCGATCCCGCCT--T 11167

  contig10_pilo     721615 TTTGCACGTGTGCAATTCATTGCCGCATCTGGAGTGGC-CTCCCGGATAC 721663
                           i      -------     ii ----  ii        -  vi i  iiv
C rnd-4_family-      11166 CTTGCAC-------ATTCACCG----ATTCGGAGTGGCTCTATCAGACGA 11128

  contig10_pilo     721664 ATTG--CGTGCATGGTCTCCCGGTATATTTTACACATTAATTATATTTGT 721711
                               --    iv v -  i vv i  i              i      v 
C rnd-4_family-      11127 ATTGCTCGTGTTTCG-CTTCATGCATGTTTTACACATTAATCATATTTTT 11079

  contig10_pilo     721712 AAACTATAGTCGACGCACTACA---AGTCGCGATC--GCTTGGGT-GAAC 721755
                             ivi  -  i  -ii   iv --- iv     v -- v i   i-   v
C rnd-4_family-      11078 AAGGCAT-GTTGA-ATACTGGATGGAAACGCGAACAGGATCGGGCGGAAG 11031

  contig10_pilo     721756 ATATTAGATCGTCACAAAATAAGA----TGGGCAA-ACTTTGAACGAGAG 721800
                           i     v  v vi   iv   i i----  iv   - ii    i  -  i
C rnd-4_family-      11030 GTATTACATGGGTACAGTATAGGGCATTTGATCAACATCTTGAGCG-GAA 10982

  contig10_pilo     721801 CAGTAAGTACTACTTCATTACAGGATTTGCGCACTTTGGGCGCGAGGTAT 721850
                             v vvii i i iv     v   v ------------ i  v i ii i
C rnd-4_family-      10981 CACTTTACATTGCCACATTAGAGGCT------------GACGGGGGACAC 10944

  contig10_pilo     721851 -CGAGTGGGGTTGTATAGAGAC-CCATCAACTAGGCGTGAGATCACGTGG 721898
                           -  v   vii v  i v i   -   v iiv  v i   viv   -----
C rnd-4_family-      10943 GCGTGTGTAATAGTGTTGGGACGCCAGCGGATACGTGTGTATTCA----- 10899

  contig10_pilo     721899 ACATTTCCCAGGTGGTTCCCTCTCGAGGGTTCCAGTGGTCGAATCGATTG 721948
                           ----           v   v  v          iv vv     v  i i 
C rnd-4_family-      10898 ----TTCCCAGGTGGATCCATCGCGAGGGTTCCGTTCCTCGAAGCGGTCG 10853

  contig10_pilo     721949 TGGTGGGCGTCCCAGCGCTCGTTA------GTCCACGTGAACTTGCCGTC 721992
                              vviv   vvi-v      iv ------ i   v v            
C rnd-4_family-      10852 TGGATACCGTGAT-CCGCTCGCGAAAAGCGGCCCAGGAGAACTTGCCGTC 10804

  contig10_pilo     721993 GCTACGGCGCACGACGCCGGTGCTCCAGAAGCATGACCCCGCCCTGATTG 722042
                            vvi     vii  ii vv          vi  v     v  i     i 
C rnd-4_family-      10803 GGAGCGGCGAGTGATACGCGTGCTCCAGACACAGGACCCGGCTCTGATCG 10754

  contig10_pilo     722043 CAAACGATTCAAGGCTGGGAATCAT-TGAAACATACGGTATGGCGA 722087
                            i      i          i -   -i  i  iv i  v       
C rnd-4_family-      10753 CGAACGATCCAAGGCTGGGGA-CATGCGAGACGAATGGGATGGCGA 10709

Matrix = 20p53g.matrix
Kimura (with divCpGMod) = 38.74
Transitions / transversions = 1.42 (643/452)
Gap_init rate = 0.05 (170 / 3422), avg. gap size = 1.42 (241 / 170)

 
 

 
   +    3691    5.2  0.4  0.0  contig10_pilon   722168  722665 (1130014) +  rnd-4_family-5204  Unknown              1    500     (0)   184     
 
 ANNOTATION EVIDENCE: 
  3691   5.22 0.40 0.00  contig10_pilon   722168  722665   1130014 +  rnd-4_family-5204  Unknown              1    500       0      
3691 5.22 0.40 0.00 contig10_pilon 722168 722665 (1130014) rnd-4_family-5204#Unknown 1 500 (0) m_b696s001i4

  contig10_pilo     722168 AAGGGGGCACCGGGATCAAAATCCCGGAACCAAACCTAGGTAGGTAGGTT 722217
                                                                            i
  rnd-4_family-          1 AAGGGGGCACCGGGATCAAAATCCCGGAACCAAACCTAGGTAGGTAGGTC 50

  contig10_pilo     722218 GCAGCCCGGAGGGCTGCGAACGTAACTGGAGTCCCATATACTCGGCTTGC 722267
                                                                             
  rnd-4_family-         51 GCAGCCCGGAGGGCTGCGAACGTAACTGGAGTCCCATATACTCGGCTTGC 100

  contig10_pilo     722268 CGAGCCGCTCTGGACGAATTTTTTCATCAAAATGCACCT-TTCTGCACTA 722316
                                           i      ?               -          
  rnd-4_family-        101 CGAGCCGCTCTGGACGGATTTTTNCATCAAAATGCACCTCTTCTGCACTA 150

  contig10_pilo     722317 TTTTTCCTGAAAGAGAGCCTCAAAACAGGTCAATACGTCAGTCCCGGCTG 722366
                             iiiv            ?        i   v                  
  rnd-4_family-        151 TTCCCACTGAAAGAGAGCNTCAAAACAAGTCTATACGTCAGTCCCGGCTG 200

  contig10_pilo     722367 TCTGAACCACATCCAGGCCCTTTCCATGAGCTGGCGTGGGTAGAGTACAC 722416
                               ?      i              ? i     v               
  rnd-4_family-        201 TCTGNACCACACCCAGGCCCTTTCCANGGGCTGGGGTGGGTAGAGTACAC 250

  contig10_pilo     722417 TGAAAACTAATGAAAGATGTTGTATTGCAACAAAGTATATTAGTTTAGAG 722466
                                 ?         ii    ?                ivv v      
  rnd-4_family-        251 TGAAAANTAATGAAAGGCGTTGNATTGCAACAAAGTATACGTGATTAGAG 300

  contig10_pilo     722467 TGCCTTTTAAGCTTTTCAACCTATCTTACATTGATCACAAGGAGTTTCTT 722516
                           i      i       ?      v                           
  rnd-4_family-        301 CGCCTTTCAAGCTTTNCAACCTCTCTTACATTGATCACAAGGAGTTTCTT 350

  contig10_pilo     722517 TACTGAAGTCAGAACTAGTTTGCTTCCGGCTCCGCGGAAGGCCTTCCCAA 722566
                                ?  i i    i                       i        i 
  rnd-4_family-        351 TACTGNAGCCGGAACCAGTTTGCTTCCGGCTCCGCGGAAAGCCTTCCCGA 400

  contig10_pilo     722567 AATGGGAATATCTTCCCACGGTAGGTAGGTAGGTCGGCCTCGCCAGCTTC 722616
                                v                                            
  rnd-4_family-        401 AATGGCAATATCTTCCCACGGTAGGTAGGTAGGTCGGCCTCGCCAGCTTC 450

  contig10_pilo     722617 CCAGCGTAAACCGACCC-GGTTCCGTCATTTTGCTTCCGGTGCCCCCTTG 722665
                                            -                                
  rnd-4_family-        451 CCAGCGTAAACCGACCCAGGTTCCGTCATTTTGCTTCCGGTGCCCCCTTG 500

Matrix = 20p53g.matrix
Kimura (with divCpGMod) = 4.34
Transitions / transversions = 2.25 (18/8)
Gap_init rate = 0.00 (2 / 497), avg. gap size = 1.00 (2 / 2)

 
 
 
 
  +    4354   32.2  1.1  0.9  contig10_pilon   722669  724504 (1128175) C  rnd-4_family-1113  LTR/Gypsy      (17099)  10634    8794   183     
 
 ANNOTATION EVIDENCE: 
  4354  32.25 1.14 0.87  contig10_pilon   722669  724504   1128175 C  rnd-4_family-1113  LTR/Gypsy         8794  10634   17099      
4354 32.25 1.14 0.87 contig10_pilon 722669 724504 (1128175) C rnd-4_family-1113#LTR/Gypsy (17099) 10634 8794 m_b696s001i5

  contig10_pilo     722669 GGAACATGGCCGTATGCTGCAGAGATGAGCATGCTAATAGGGGCCATGTC 722718
                                i  v     i  v  v    v      iiii  vvvv v ii i 
C rnd-4_family-      10634 GGAACGTGTCCGTACGCGGCCGAGAGGAGCATATCGATTCCTGACGCGCC 10585

  contig10_pilo     722719 GTAGCCACTCGAGAGCCACATGGTGTTGACTTCGAGATGACGCAGAGACC 722768
                             i  i vii iivv  i  i  i   v     i  i        v  i 
C rnd-4_family-      10584 GTGGCTAACTGGACCCCGCACGGCGTTCACTTCAAGGTGACGCAGTGATC 10535

  contig10_pilo     722769 GGTGTCCGCTCAGCGCGAGAGCGTATTCGTGATTGGGGTGGTCCCAGTCG 722818
                            i  v  vivv  i  vv v     v    ivvi    i           
C rnd-4_family-      10534 GATGACCCTGGAGTGCCTGCGCGTAGTCGTACGCGGGGCGGTCCCAGTCG 10485

  contig10_pilo     722819 CACAGGGTGATTTCCTTGTGTGAAGGGCTCTGACGTCGAGCGAGGCTGAG 722868
                             v ii   vvv        vi vv    i vi      i    i     
C rnd-4_family-      10484 CAAAAAGTGTAGTCCTTGTGGAACTGGCTTTCGCGTCGAACGAGACTGAG 10435

  contig10_pilo     722869 GCATTCGAGACGTGGGAGCTTGTCTCGAAGCTGCAT-CATGAAAT-CGAG 722916
                           v vviv     v   i  v     v    --  vi - iv i   -v  v
C rnd-4_family-      10434 CCCGCGGAGACTTGGAAGGTTGTCGCGAA--TGAGTGCGAGGAATGAGAC 10387

  contig10_pilo     722917 GGGGACGCGATCGAGGCCTTCGAGGACAAGCACCGACAGGGCTGGGTAAT 722966
                           iv          v iv  i     i  i  iiiv     vv v  i  i 
C rnd-4_family-      10386 ATGGACGCGATCCAACCCCTCGAGAACGAGTGTGGACAGCTCGGGATAGT 10337

  contig10_pilo     722967 GCTCAGCCAGAACTTCGACGAGCCCGGTCAAATGCTCCTGATGCAGTCCC 723016
                            vvii  i i   v i      vi       i vvi    ivi  v    
C rnd-4_family-      10336 GGGTGGCTAAAACATTGACGAGGTCGGTCAAGTCGCCCTGGAACATTCCC 10287

  contig10_pilo     723017 ACGCGAGAGTGCAGCTTCAGTTTGCGAAGACGACCGGGGGCCGCGGTTCT 723066
                                      i              i     i  i  vvi v  v   v
C rnd-4_family-      10286 ACGCGAGAGTGTAGCTTCAGTTTGCGGAGACGGCCAGGCTTCCCGCTTCG 10237

  contig10_pilo     723067 CCGGGCACAGACTGCGAGCCAGCCAACCAAACCCTCGGTGTACCTCGTTG 723116
                           vv     v vv iv i       vi     iv v  i   v    i vi 
C rnd-4_family-      10236 GAGGGCAGATCCCTCAAGCCAGCGGACCAAGGCGTCAGTGAACCTTGACG 10187

  contig10_pilo     723117 TACCGTCGATATGCAGACACTCAACGCTGAGGATGTCAGGCATGTCGTTG 723166
                            i  v vi         vi   i   i  v   v   vi  iv    vi 
C rnd-4_family-      10186 TGCCCTGAATATGCAGAAGCTCGACGTTGTGGAGGTCTAGCGGGTCGGCG 10137

  contig10_pilo     723167 GCGAGGTCTGGGTAGCGTATGACAGTTTCGTATATGTCCAGGGCGTAGGC 723216
                           ivi i   i  i  v  i iv  viii     v     v  vv    v  
C rnd-4_family-      10136 AGAAAGTCCGGATACCGCACCACCACCTCGTAGATGTCGAGTCCGTACGC 10087

  contig10_pilo     723217 AAACAGCTGTCGCAGAGCCGGGAATAGGACCACGACGGCCCATTGCTGGC 723266
                           iv v vi        v  v     i vviv   v i  v   i  v  i 
C rnd-4_family-      10086 GTAAATTTGTCGCAGCGCGGGGAACATTGACACCATGGACCACTGGTGAC 10037

  contig10_pilo     723267 GATGCGTGTAATGCTCGCCGTGGAACCCGAGGGCGAGGTAGGTAACGTTC 723316
                                     i  i                v   v        i i   v
C rnd-4_family-      10036 GATGCGTGTAGTGTTCGCCGTGGAACCCGATGGCCAGGTAGGTGATGTTG 9987

  contig10_pilo     723317 GTGAGGCGAAAGGAGCTGTATCTCTCGATCGTCATGGCAGGATAGATCGC 723366
                            iv  i  v ii        iv  v    viiiiv   i   v v    v
C rnd-4_family-       9986 GCCAGACGCAGAGAGCTGTACGTCGCGATGACTGAGGCGGGAAACATCGA 9937

  contig10_pilo     723367 GTAGCGCTCGATGGTCATGCGCGTGATGGCCGGGTGTCCGCGGAGATGTT 723416
                           i     i    i   v vi  v       vv     i  v  i     iv
C rnd-4_family-       9936 ATAGCGTTCGACGGTGAGACGAGTGATGGAGGGGTGCCCCCGAAGATGCA 9887

  contig10_pilo     723417 CGACGAGTCTATGCGT-CATGAGTAGTCCGATGAACCGCACGTGGTTGAC 723465
                           vi v   i  v  i  -v   v -  v   v   iv        v i   
C rnd-4_family-       9886 GAAGGAGCCTCTGTGTGAATGCG-AGACCGCTGAGGCGCACGTGTTCGAC 9838

  contig10_pilo     723466 GTTGCCCAGATGAGCAAGCAGCGCAATCACGCTATTGTAGAACGGCCCAA 723515
                            vi  iv  i     i    vv v   v i   i     i  i   i i 
C rnd-4_family-       9837 GACGCTGAGGTGAGCGAGCATGGAAATGATGCTGTTGTAAAATGGCTCGA 9788

  contig10_pilo     723516 ATAT-CTCGTCGTCGAGTGGCGAGGGCATGATGTCGAATCTGTGAGTGAA 723564
                            v i- v i -     v  vvvv     vi vv          vv  i  
C rnd-4_family-       9787 AGACGCGCAT-GTCGATTGCGTTGGGCAGAAGTTCGAATCTGTCTGTAAA 9739

  contig10_pilo     723565 CGGCGGCCAGAGGGTCTCCCATTGATTCTCGAAGCTGACGAACCGAGCCC 723614
                                                   i     i       iiv v   i   
C rnd-4_family-       9738 CGGCGGCCAGAGGGTCTCCCATTGGTTCTCAAAGCTGATATAGCGAACCC 9689

  contig10_pilo     723615 AGCGGCAGTTGTTCACGTTCTGCACGAAGAACGTCATCGCGCTCGTCGC- 723663
                               i  i  v i vi  ivi vi iv  ivvv    vii   v    i-
C rnd-4_family-       9688 AGCGACAATTTTCCTTGTCGCGAGCATAGGTGCTCATGATGCTGGTCGTA 9639

  contig10_pilo     723664 CTCAGTCCTCTCCTCGACCATGCTCTTGACGATGTCCATGAATAACGCAT 723713
                           v  v  -  v vi v  i   i  i i  vi  v  v     v i v i 
C rnd-4_family-       9638 ATCCGT-CTGTGTTGGATCATACTTTCGAGAATCTCAATGAAGAGCTCGT 9590

  contig10_pilo     723714 CGCGGCCGTAGGGAGGTGGTCGGTGGATGCACTCCATGTTGTAGTCAATC 723763
                               vvv viv    ii          v vv        i         v
C rnd-4_family-       9589 CGCGCGAGGGCGGAGACGGTCGGTGGAGGATCTCCATGTCGTAGTCAATG 9540

  contig10_pilo     723764 CGGAAGAAGGCTTTCTGTGCTCATCTCAGCCGAGTTACCATCCAGGCGTG 723813
                             i        v     iiiiiiiv      i    iv i viv i  ii
C rnd-4_family-       9539 CGAAAGAAGGCATTCTGCATCTGCATCAGCCAAGTTGACGTATCGACGCA 9490

  contig10_pilo     723814 GTTTACGTACCTCACTCACTCTCCTCGAGCATATGTCGCGGAAACCTCGA 723863
                            v     i     v vv ii vv vi       vv i   i  v      
C rnd-4_family-       9489 GGTTACGCACCTCCCGAATCCGACGTGAGCATAACTTGCGAAATCCTCGA 9440

  contig10_pilo     723864 TTCGCTGCAGCCAATC-TGCGCACAGCGTAGTCGGGGACATGTGAGACGA 723912
                             i -   v v   v -    v iivi i    i  v   iii    iv 
C rnd-4_family-       9439 TTTG-TGCTGGCAAGCATGCGGATGTTGCAGTCAGGTACACACGAGATTA 9391

  contig10_pilo     723913 TCTCGTGGAGGACATCGAGCGGGAGCTGGTCGATGAAAATGGTTTGAAAC 723962
                                v    v iv  i        v v         v v v i  i   
C rnd-4_family-       9390 TCTCGAGGAGTATCTCAAGCGGGAGATCGTCGATGAACAGGCTCTGGAAC 9341

  contig10_pilo     723963 TCGGGGATTGGTTTCTTCTTGATGACAGCAAAGCTGCGAGCTGTGT-CCG 724011
                           vv     i   v v  ii ?      v vi      ii      i -  i
C rnd-4_family-       9340 GAGGGGACTGGGTGCTCTTNGATGACCGAGAAGCTGTAAGCTGTATGCCA 9291

  contig10_pilo     724012 --TGGGATCAGCCAACGGGATAAATTGCACACGGACGGA---CAATA-CG 724055
                           --      v  viivi v i vi i--  i-  i v   --- i   - v
C rnd-4_family-       9290 TCTGGGATGAGATGCTGCGGTCGAC--CAT-CGAAGGGATCGCGATAGCT 9244

  contig10_pilo     724056 TACCCAGTCGCCTAATTATGTCTGTGAACAGGTAGTCAACCGAGATTGCA 724105
                                  v    vi  v     v     vvv   vvii v        vi
C rnd-4_family-       9243 TACCCAGGCGCCGGATAATGTCAGTGAAATCGTATGTGAACGAGATTGAG 9194

  contig10_pilo     724106 AAGCGTTTGTAGAGTCCAGCCGTGAAGTCTTCGTCAACGACGACTTCGAT 724155
                              vvi  i     ii iv       v iiivv  i i v   iii    
C rnd-4_family-       9193 AAGGCCTTATAGAGCTCGTCCGTGAACTTCCGTTCGATGTCGATCCCGAT 9144

  contig10_pilo     724156 TATGTCTGTGCGATACACGATGCGCGCGAATGGAAGGACATCTTCGACAG 724205
                           iv    ii v i    vi  ii  v  i  v  v  i  iiv     vi 
C rnd-4_family-       9143 CTTGTCCATCCAATACCTGACACGAGCAAAGGGCAGAACGCATTCGAGGG 9094

  contig10_pilo     724206 CAGATAGGAACGTCAGGTATTGATCCAAGACGTCTTCGTAGAGCTACACA 724255
                            i vi       iv v   vi v     ii      i   viv    iv 
C rnd-4_family-       9093 CGGCCAGGAACGCAATGTAGCGTTCCAAAGCGTCTTTGTACGTCTACGGA 9044

  contig10_pilo     724256 GTGTCACCATCTAGAACGACAGC------ACGAGGCCGAGCGCACCTCCC 724299
                           i i  ----  vi     - i  ------i     iiiii  i   vivv
C rnd-4_family-       9043 ATATC----TCAGGAACG-CGGCGGGCGAGCGAGGTTAGACGTACCATGA 8999

  contig10_pilo     724300 GGGAGCGAGGCCTGATCATCCTGGGAAGGCAGCTCTGCGTAG-ATTGAAT 724348
                                     i    i   v ii  i     v i   i  i - i   iv
C rnd-4_family-       8998 GGGAGCGAGGTCTGACCATGCCAGGGAGGCATCCCTGTGTGGTACTGAGG 8949

  contig10_pilo     724349 GTCAGTGGTGT-GTTGTGAGCGCGGGACTGACGGACCATGTGGAAGCCTG 724397
                            i i i i v -  i  vv i  ivvv --   v    v   i     v 
C rnd-4_family-       8948 GCCGGCGATCTCGTCGTTCGTGCATTCC--ACGTACCAGGTGAAAGCCAG 8901

  contig10_pilo     724398 TGACCATCTCTATGAAATCGAGCGTGTTTGGATGCCTTGACGTCCATCGC 724447
                           i    v    v     i     v v  i   iv  iii  vvv    i  
C rnd-4_family-       8900 CGACCTTCTCGATGAAGTCGAGGGAGTCTGGGAGCTCCGAACGCCATTGC 8851

  contig10_pilo     724448 AGTGGGGTCATCCGCGTTACGAAGGAACAGACGACGGAGGAGCTGTTCGT 724497
                           v i  i  v vi     i   v  viv      i       v        
C rnd-4_family-       8850 TGCGGAGTAAGTCGCGTCACGTAGCGCCAGACGGCGGAGGACCTGTTCGT 8801

  contig10_pilo     724498 GGTGTAT 724504
                              i   
C rnd-4_family-       8800 GGTATAT 8794

Matrix = 20p53g.matrix
Kimura (with divCpGMod) = 37.94
Transitions / transversions = 1.05 (300/287)
Gap_init rate = 0.02 (29 / 1835), avg. gap size = 1.28 (37 / 29)

 
 

 
   +      14   15.4  6.7  2.1  contig10_pilon   724536  724580 (1128099) +  (TGAGA)n           Simple_repeat        1     47     (0)   185     
 
 ANNOTATION EVIDENCE: 
    14  15.38 6.67 2.13  contig10_pilon   724536  724580   1128099 +  (TGAGA)n           Simple_repeat        1     47       0      
14 15.38 6.67 2.13 contig10_pilon 724536 724580 (1128099) (TGAGA)n#Simple_repeat 1 47 (0) m_b696s252i2

  contig10_pilo     724536 TGA-ATGAGAGGATGATGATTTG-GTTGAGGTGAGATGAGGT-AGATG 724580
                              -      v  -     vv  - v    i         i -     
  (TGAGA)n#Simp          1 TGAGATGAGATGA-GATGAGATGAGATGAGATGAGATGAGATGAGATG 47

Matrix = Unknown
Transitions / transversions = 0.50 (2/4)
Gap_init rate = 0.09 (4 / 44), avg. gap size = 1.00 (4 / 4)

 
 
 
 
  +     514   35.1  7.9  1.8  contig10_pilon   724644  725113 (1127566) C  rnd-4_family-1113  LTR/Gypsy      (19101)   8632    8135   183     
 
 ANNOTATION EVIDENCE: 
   514  35.11 7.87 1.81  contig10_pilon   724644  725113   1127566 C  rnd-4_family-1113  LTR/Gypsy         8135   8632   19101      
514 35.11 7.87 1.81 contig10_pilon 724644 725113 (1127566) C rnd-4_family-1113#LTR/Gypsy (19101) 8632 8135 m_b696s001i6

  contig10_pilo     724644 AGCGTAAGGCACATGGGCAGTTCAAGGGGACAGGCACGC-CTAGAAACGT 724692
                              iii v   v i  i vi  ii i v           - i  vv  v 
C rnd-4_family-       8632 AGCACGACGCAAACGGACCATTTGAAGCGACAGGCACGCACCAGCTACCT 8583

  contig10_pilo     724693 CATGGATTAACAAT-CCACAGCGTTAG--GCGGGGTGTTTCTGGTG---- 724735
                           v     v v ivi -i  v  v i i --  ii       vi  ? ----
C rnd-4_family-       8582 AATGGAATCATTGTGTCAGAGGGCTGGCTGCAAGGTGTTTGCGGNGACAG 8533

  contig10_pilo     724736 -------TAGCAGAC-CCGCGTGGGTGCCCGTTGACTCGCCTGTG--TTG 724775
                           -------ii    i - i ii   v    - i        v  v --   
C rnd-4_family-       8532 GTTCGAGCGGCAGGCACTGTATGGTTGCC-GCTGACTCGCGTGGGCCTTG 8484

  contig10_pilo     724776 CTCAGCGAGCGTGGGGCCGTCAAAAATGTGATTTTAGGCACTGGTAGGCA 724825
                            v             viv     v  v i   i  i   i    - v  -
C rnd-4_family-       8483 CGCAGCGAGCGTGGGTTGGTCAACAAGGCGATCTTGGGCGCTGG-ACGC- 8436

  contig10_pilo     724826 CTGGATGG---CGGCAC---ATAACCGTGTGTGAGCAGCT--CGTCTGAG 724867
                           --  i   ---v     ---     i   iii iii iii--i  vi  i
C rnd-4_family-       8435 --GGGTGGGGAAGGCACGGCATAACTGTGCACGGATAATCACTGTGCGAA 8388

  contig10_pilo     724868 GGGTTGCAGTAATGAGATCTAGCGGTCGATCGTGGGAGCCGCACACGCAC 724917
                           i i ---ii   i     vii i    - i    i  vvv v iv iv  
C rnd-4_family-       8387 AGAT---GATAACGAGATGCGGTGGTC-ACCGTGAGACGGGGATCCAGAC 8342

  contig10_pilo     724918 AGTTGGAGATGAAATAATGGTGTTTCTGACGGATCGAGACCTGTGTGAGT 724967
                                  vvvi   ii ivi iv i  ii vv vii   v     i  i 
C rnd-4_family-       8341 AGTTGGACCGAAAACGACCATAATCCTAGCCCAATAAGAGCTGTGCGAAT 8292

  contig10_pilo     724968 CCC-----------AGCAAACATTAGGTACGAAGAGGGGTGCTTACAATC 725006
                            v -----------      ivii ii  i  viv  i  v      i  
C rnd-4_family-       8291 CGCCGTCCGCTTATAGCAAATCCCAAATATGATACGGAGTCCTTACAGTC 8242

  contig10_pilo     725007 AGTCTGTAGCACGTCAGGCCTTTCTCTGAGTTCCGAACAGGTCACGTTCC 725056
                             v  i v        ivv viivivi      i iv i           
C rnd-4_family-       8241 AGACTATCGCACGTCAACGCGCCGCGCGAGTTCTGGTCGGGTCACGTTCC 8192

  contig10_pilo     725057 ATTGGCGCGTAAGCAGTTCGACGTGGAGCACGCTCGGGCGTAGAGCAAGG 725106
                           i i    v ii  i  i  ii  v v    i  v    v vv   i vv 
C rnd-4_family-       8191 GTCGGCGGGCGAGTAGCTCAGCGAGCAGCATGCGCGGGGGACGAGTATCG 8142

  contig10_pilo     725107 ACGTGGT 725113
                            v   i 
C rnd-4_family-       8141 AAGTGAT 8135

Matrix = 20p53g.matrix
Kimura (with divCpGMod) = 41.69
Transitions / transversions = 1.53 (98/64)
Gap_init rate = 0.04 (20 / 469), avg. gap size = 2.30 (46 / 20)

 
 

   +     957   31.4  1.8  1.6  contig10_pilon   725510  725953 (1126726) C  rnd-4_family-1113  LTR/Gypsy      (19957)   7776    7332   183     
 
 ANNOTATION EVIDENCE: 
   957  31.35 1.80 1.57  contig10_pilon   725510  725953   1126726 C  rnd-4_family-1113  LTR/Gypsy         7332   7776   19957      
957 31.35 1.80 1.57 contig10_pilon 725510 725953 (1126726) C rnd-4_family-1113#LTR/Gypsy (19957) 7776 7332 m_b696s001i7

  contig10_pilo     725510 CTCTTCTATTGTCAGGGGTGTTCCTCCCTTTGGTTCTGAGCATATGTTTC 725559
                            i   i vv i  v  i i  i i i        vvi      i   v  
C rnd-4_family-       7776 CCCTTTTCATATCCGGAGCGTCCTTTCCTTTGGTGGCGAGCATGTGTGTC 7727

  contig10_pilo     725560 AGCTTCTTTACGTAGGGTTTGAACTGAGCGTTGATCTCGGAGTCATTGAA 725609
                                iv i  i iv   vvi   vi i   iv v  i  i   i i   
C rnd-4_family-       7726 AGCTTTGTCACATGCGGTGAAAACAAAACGTCCAACTTGGGGTCGTCGAA 7677

  contig10_pilo     725610 GAGATCGAGGATGCGCTGTGCAACGGTGTTCG--TCAGGCCGGGGCGCAA 725657
                              v        iiv   v?v  - v    v --     --  iv  iii
C rnd-4_family-       7676 GAGTTCGAGGATATTCTGANGAA-GCTGTTAGCTTCAGG--GGATCGTGG 7630

  contig10_pilo     725658 TCGACGGCAGAC---AACGCACCAAGACGATATTCGCTTCTTCGACCCAC 725704
                            -i   iv v  ---    v    i   vi vi    i  vivv      
C rnd-4_family-       7629 T-AACGAAACACGGAAACGGACCAGGACCGTCCTCGCCTCGCGCACCCAC 7581

  contig10_pilo     725705 AGCTGAGTCACAAAGGTGTGTCTGGACAGATTCTTTGCTGCGTCGTAGAG 725754
                            i         v iv  i  iv v  v  i     v  v   i       
C rnd-4_family-       7580 AACTGAGTCACCAGTGTATGCATTGAGAGGTTCTTGGCGGCGCCGTAGAG 7531

  contig10_pilo     725755 ACGGTCTACGAATTCAGC-CGCAGT-ATCACAAGTGTCGACGGGTTCCCA 725802
                                 i     --   i-   v  - v  vvv        v    i   
C rnd-4_family-       7530 ACGGTCCACGAA--CAGTGCGCCGTCAGCAGCTGTGTCGACCGGTTTCCA 7483

  contig10_pilo     725803 GAATGGCGAGTCCCAGTAGAAGATTAGCTTTCGCGCCGCTTGGAAATTCT 725852
                              i        vi  v vv v  i  v iv  i  vv i v   v  iv
C rnd-4_family-       7482 GAACGGCGAGTCGTAGAACCATATCAGGTCGCGTGCGTCCTCGAACTTTG 7433

  contig10_pilo     725853 CTGTGACGCTGCGCTGCGCCGCGTCAGCCCCGAACTA-TCGTTGTACTCT 725901
                            ivv    vi       ivi        vviviv i -     - vivi 
C rnd-4_family-       7432 CCCAGACGGCGCGCTGCAATGCGTCAGCAGTTGTCCATTCGTT-TCTGTT 7384

  contig10_pilo     725902 GTGAAAGACCCACCGAGTGCTTGGCAGCGATGTACACATACTTGTGCCTA 725951
                             iv v    v       v      v i ii   v  i i      ii  
C rnd-4_family-       7383 GTACACGACCGACCGAGTCCTTGGCCGTGGCGTAAACGTGCTTGTGTTTA 7334

  contig10_pilo     725952 AT 725953
                             
C rnd-4_family-       7333 AT 7332

Matrix = 20p53g.matrix
Kimura (with divCpGMod) = 37.16
Transitions / transversions = 0.99 (68/69)
Gap_init rate = 0.03 (12 / 443), avg. gap size = 1.25 (15 / 12)

 
 
 
  +    3486   32.5  2.4  6.3  contig10_pilon   726155  728110 (1124569) C  rnd-4_family-1113  LTR/Gypsy      (20599)   7134    5251   183     
 
 ANNOTATION EVIDENCE: 
  3486  32.50 2.35 6.26  contig10_pilon   726155  728110   1124569 C  rnd-4_family-1113  LTR/Gypsy         5251   7134   20599      
3486 32.50 2.35 6.26 contig10_pilon 726155 728110 (1124569) C rnd-4_family-1113#LTR/Gypsy (20599) 7134 5251 m_b696s001i8

  contig10_pilo     726155 CACTGCGTTTTATATGCCTGTGACGGA--GTCGATATTTATTAAATATTC 726202
                            i   v i     i  ii -i i   i--  i    i vi---     v 
C rnd-4_family-       7134 CGCTGGGCTTTATGTGTTT-CGGCGGGCGGTTGATACTAG---AATATGC 7089

  contig10_pilo     726203 ATATATTGTAAAGTGGGTGAGTCAACGATGGAGTGCGGAAATACAGGGCA 726252
                             i      iii  vvviii i ivv  iii iivv v          vi
C rnd-4_family-       7088 ATGTATTGTGGGGTTCTCAGGCCGCGGACAAAACTGGTAAATACAGGGGG 7039

  contig10_pilo     726253 TCGCCCGCGAAAGGGGTCATTAAAACGTGGTTACATGTTTTGAGTCAAAT 726302
                            -i           i             iii  vv i   i      ---
C rnd-4_family-       7038 T-ACCCGCGAAAGGAGTCATTAAAACGTAACTAGTTATTTCGAGTCA--- 6993

  contig10_pilo     726303 TACGTTGCGGTAGCTATTAGTTTCCTGAGAGCTCGAGGTCGTCGATACCG 726352
                           ---------------------   vv  -  v ii  i i       ii 
C rnd-4_family-       6992 ---------------------TTCGAGA-AGATTAAGATTGTCGATATTG 6965

  contig10_pilo     726353 TTCTGGACCCTACGGACATTCTCCGCCATTTCTACATCACCGCCGCTGAC 726402
                           vv  ivi  i  v ii vv    i        v  i   v ?  ii v  
C rnd-4_family-       6964 AGCTACGCCTTAGGAGCCGTCTCTGCCATTTCGACGTCAGCNCCATTCAC 6915

  contig10_pilo     726403 TGAACCGGTCAGATATCA-GAATTGGTGTGGCACGTGATCATGC---TTA 726448
                           v vi  v    vi iivi-   i -- v  i        vv   ---  i
C rnd-4_family-       6914 GGCGCCTGTCACGTGCGGTGAACT--TCTGACACGTGATGCTGCAGATTG 6867

  contig10_pilo     726449 AGTCAGCATACCTTGTTGGGTCGACAAGTTGGTTATGTCGGCGAGGACGC 726498
                           v  v v i        iv ---------     i v   iv    v    
C rnd-4_family-       6866 TGTAATCGTACCTTGTCTG---------TTGGTCAGGTCACCGAGCACGC 6826

  contig10_pilo     726499 GACGAGTGATGGTTCCGGCCTCTGGGATCCTCTTGCGTTTCAGCTTCTGG 726548
                            i  i     v  i    ii  i v   i              i      
C rnd-4_family-       6825 GGCGGGTGATCGTCCCGGTTTCCGCGATTCTCTTGCGTTTCAGTTTCTGG 6776

  contig10_pilo     726549 CTCGAGTGTGCCTGAACCGCCGGCTCTGCAATGGCGCGTGCACGGCATGC 726598
                           iii iiivv i  vvii         v vv  v       iv v i vi 
C rnd-4_family-       6775 TCTGGACCAGTCTCCGTCGCCGGCTCAGGCATCGCGCGTGTTCTGTAGAC 6726

  contig10_pilo     726599 CTGT-TGAATGTCAGTTTACACGATGGAAGCGGTCTGTGGCTCACATGCG 726647
                              i-i ii         iii iiiiv v  i iv i vivvv  v vi 
C rnd-4_family-       6725 CTGCGCGGGTGTCAGTTTGTGCAGCATATGCAGCGTATCAAGGACCTTTG 6676

  contig10_pilo     726648 AAAGTGAATTGTCCATGCTGACAATGGAACGGAGCACCAGTTTCGCGAAC 726697
                            v iiiv   i    v   v  i     i  v     v           v
C rnd-4_family-       6675 ACAACACATTATCCAGGCTTACGATGGAGCGCAGCACAAGTTTCGCGAAG 6626

  contig10_pilo     726698 AGCTGTTCCCTGTCCGCGTGAAATAAGACGGGGCTGAAATGCACTTCGAC 726747
                             vviv   i v    v   i    iv      vv         i     
C rnd-4_family-       6625 AGGAAATCCTTCTCCGAGTGGAATAGCACGGGGGAGAAATGCACCTCGAC 6576

  contig10_pilo     726748 GATGTCTCCTCGCCGGAAAGAGCGCGGCGACGTCGTTTGGCTGCCGCCTT 726797
                              i  v     vi                v  v vvi i   ii  vi 
C rnd-4_family-       6575 GATATCGCCTCGATGGAAAGAGCGCGGCGAAGTAGGACGACTGTTGCGCT 6526

  contig10_pilo     726798 TGGTCAGCATTTCCACTGTGCACCGCGTGTATCCCACGCACTATCCCAAT 726847
                           i   i  vv ii  i vvi   iv viiiii  i       i      iv
C rnd-4_family-       6525 CGGTTAGACTCCCCGCGTCGCATAGAACACGTCTCACGCACCATCCCAGA 6476

  contig10_pilo     726848 CCAGGCCGGTAATGCAGGTGCTGCACCGAATTGTCGTCGGTGCGCACCAG 726897
                           iii     i     iii  i       ii   v  v      i      i
C rnd-4_family-       6475 TTGGGCCGATAATGTGAGTACTGCACCAGATTTTCCTCGGTGTGCACCAA 6426

  contig10_pilo     726898 GTCCTTCTGTCTCGCCGCAGTGCGGAGGTCACGGAGAGGGTCCATAATAT 726947
                                 v v         i     i     ii    v  i     i  i 
C rnd-4_family-       6425 GTCCTTGTTTCTCGCCGCGGTGCGAAGGTCGTGGAGCGGATCCATGATGT 6376

  contig10_pilo     726948 GGGTAATGGGTACCGCGACTTGAGGTTGGGTCTGATCTATTGGCGAGAAC 726997
                            v iv v     ii  i     v v     vv  i  vvvi    v    
C rnd-4_family-       6375 GCGCTAGGGGTATTGCAACTTGCGTTTGGGAGTGGTCGCGCGGCGTGAAC 6326

  contig10_pilo     726998 ATTCGGTTGGAGAATGTAATCGGGCGATTGATATTGGTGATGCACGCATG 727047
                                         v  i  v  i  vviii i   v       v  i  
C rnd-4_family-       6325 ATTCGGTTGGAGAAGGTGATAGGACGTGCAGTGTTGCTGATGCAGGCGTG 6276

  contig10_pilo     727048 CGTATCGACGGGGAACGGAGCCAGGGACGATACAAGGTCCAAAATGTCGA 727097
                           v      i i ii  v  i    v       v i  v     i     ii
C rnd-4_family-       6275 GGTATCGGCAGAAAAAGGGGCCATGGACGATTCGAGTTCCAAGATGTCAG 6226

  contig10_pilo     727098 CGACTGCCTGGATGTTGCGCGCGTACGAGTCCGAGTCAGACGCCGTAATA 727147
                            v  i   i       vi  i             v vv     v ii  i
C rnd-4_family-       6225 CTACCGCCCGGATGTTCTGCACGTACGAGTCCGATTGCGACGCGGCGATG 6176

  contig10_pilo     727148 CTAACTTTCTGCTGAAGGTATGCGATAGTGGAGGGACGACTGTCATGGGA 727197
                             v i i i           vi i  iv    i ii        ii vvv
C rnd-4_family-       6175 CTTATTCTTTGCTGAAGGTAGACAATGTTGGAAGAGCGACTGTCGCGTTC 6126

  contig10_pilo     727198 CAGTCAGC-GACGACAAGGGATTTTTGAGATCCACGCACGTAAACCTATG 727246
                            i      -     -iv  v  viii v i vi        i    v   
C rnd-4_family-       6125 CGGTCAGCAGACGA-GTGGCATGCCCGTGGTGTACGCACGTGAACCGATG 6077

  contig10_pilo     727247 CAGAAAGGGGGGTAGGCTCACGTCGTCGACGATACCGATCAACCGGAACG 727296
                           vv    i  v  i   i v     v     i  i  i  v ii vi   i
C rnd-4_family-       6076 GTGAAAAGGTGGCAGGTTAACGTCTTCGACAATGCCAATGAGTCTAAACA 6027

  contig10_pilo     727297 TGATTTCGCTGCCGTCCTC---TGCATT--CGCTGCG-TCGAGGAGTCGG 727340
                           ii v    i  i v   i ---  iv  --  ii   -    v   i v 
C rnd-4_family-       6026 CAAGTTCGTTGTCCTCCCCGTTTGTCTTGCCGTCGCGCTCGACGAGCCTG 5977

  contig10_pilo     727341 TAAGATGTTGGAGATCCAAAGGGAACACAGTTGGCCTTGACGGTGCCGTC 727390
                               vi  i  i  v   viv         viv  v  i i vi     i
C rnd-4_family-       5976 TAAGTCGTCGGGGAGCCATGCGGAACACAGGCCGCATTAATGTCGCCGTT 5927

  contig10_pilo     727391 GATGTAGGCCTATGTGAAAGTGAACACCGAAAGTTCAATGGAACAGCGGT 727440
                             v   i    ii vvi      ?    v vi iv vvi vi  ii  vi
C rnd-4_family-       5926 GAGGTAAGCCTGCGACGAAGTGANCACCCACGGCGCTCCGCGACGACGCC 5877

  contig10_pilo     727441 GACTG------ACCTGGTACCAGTGAGCAGGTAGCATTGTGCTCTCTGGA 727484
                            i  i------     i     v  v  v  i        vii vii   
C rnd-4_family-       5876 GGCTAGCACACACCTGATACCATTGCGCCGGCAGCATTGTTTCCGTCGGA 5827

  contig10_pilo     727485 TC--CCCGCCTTCAAGCGCTTTGAACATGTCTTCAACACGTTGATCTCCA 727532
                             --v i  --  v v   i   v v v      i  i     iv   v 
C rnd-4_family-       5826 TCGTGCTGC--TCCATCGCCTTGTAAAAGTCTTCGACGCGTTGGGCTCGA 5779

  contig10_pilo     727533 TGGTTGT--TCAGCGAAAGTGTGCGCGGCATTGTGAGCAACGTACTCGCG 727580
                            -  i  --     vi?v i iiviviv i   i   iv  vi   i  i
C rnd-4_family-       5778 T-GTCGTCATCAGCCGNCGCGCAGAGACCGTTGCGAGTTACTCACTTGCA 5730

  contig10_pilo     727581 GTCTTGAAGTCGTAGTCAGTCGATGCCATGGTGCGTGTCTGTGTGTCGGG 727630
                            ii i   i  i   iiiivv ?      vv    v  vi i i  iv v
C rnd-4_family-       5729 GCTTCGAAATCATAGCTGAGGGNTGCCATCCTGCGAGTACGCGCGTTTGC 5680

  contig10_pilo     727631 AGGGAGAGAAGGGGGGGGTATGTGAGTTTTTGGTCGCGCGTCAACGGTGA 727680
                           v   v i ---------------------i   iv   ------   i  
C rnd-4_family-       5679 TGGGCGGG---------------------CTGGCAGCG------CGGCGA 5657

  contig10_pilo     727681 ATTGGTGTAAGGTATTTCAGGCTACCCTGCAAGCGGCAAGTCAGCCGAAT 727730
                             iv iivvv        vivv i-i   iii           v vivii
C rnd-4_family-       5656 ATCCGCAGCCGGTATTTCTATGTG-TCTGTGGGCGGCAAGTCATCGACGC 5608

  contig10_pilo     727731 CGCGGCGGTTTGCCGGTGCCCAAATCAGGTAATCGCTGCATGGACG-GCA 727779
                              iiiv i ivv iv    ?v vi i i i i--     i   v -  i
C rnd-4_family-       5607 CGCAATCGCTCCGCATTGCCNTATCCGGATGAC--CTGCACGGAGGCGCG 5560

  contig10_pilo     727780 ACTTGGTCGTCGCGGGACGTGCTCA-GTCACGTG----TACTAAGAAAT- 727823
                             i  iv -     i ii iii   -i  i    ----?v  iv viv -
C rnd-4_family-       5559 ACCTGAAC-TCGCGAGGTGCATTCACATCGCGTGCTCCNTCTGTGCGTTT 5511

  contig10_pilo     727824 ---AATTTGAAACTCAATGCGATGCGGGAGGCGGTAGACGCAGCTCCGTT 727870
                           ---            ivi     i     v     - i   i i  ivi 
C rnd-4_family-       5510 GGGAATTTGAAACTCGCCGCGATACGGGACGCGGT-GGCGCGGTTCTTCT 5462

  contig10_pilo     727871 GCTCATGATGGTGCCTTGTGGGAAAGCGACGCCTTGTGGGGGAAGTTTCA 727920
                             ----   v      i       --------- i  -------vi  vi
C rnd-4_family-       5461 GC----GATTGTGCCTCGTGGGAA---------TCGT-------TCTTGG 5432

  contig10_pilo     727921 GGACGCTGTGATCATGTTCGGGACGGTGCATTGCTGCGGTG-CCACAACC 727969
                             ? i  ---v   i  iv ii      i  i -v      -i  ii  i
C rnd-4_family-       5431 GGNCACT---CTCACGTCGGAAACGGTGTATCG-GGCGGTGATCATGACT 5386

  contig10_pilo     727970 GATACAGCGGCAAACCTCATGGGTTGGAAATTCGGGCGTGTCAGGGGCGA 728019
                           v  iv     viv vvv iiv           ---------------?  
C rnd-4_family-       5385 CATGGAGCGGGGTAAGACGCCGGTTGGAAATT---------------NGA 5351

  contig10_pilo     728020 TTACCGT---CATTGT--TTGTCGATCTCTGTTATG----GATGCAGGCT 728060
                               i  ---    v --               -  ----   ivii vi
C rnd-4_family-       5350 TTACTGTACACATTCTGGTTGTCGATCTCTGTT-TGTCGAGATAAGAGGC 5302

  contig10_pilo     728061 GTGCCTATGATTTTCATACCATATCCGTCAA-GTCGTCGTTC-CCGGGAT 728108
                                        v i-    v? v    i - ii       -i v    
C rnd-4_family-       5301 GTGCCTATGATTTGCG-ACCAANTGCGTCGATGCTGTCGTTCATCCGGAT 5253

  contig10_pilo     728109 TT 728110
                             
C rnd-4_family-       5252 TT 5251

Matrix = 20p53g.matrix
Kimura (with divCpGMod) = 37.05
Transitions / transversions = 1.44 (352/244)
Gap_init rate = 0.07 (140 / 1955), avg. gap size = 1.17 (164 / 140)

 
 

   +    5200   31.9  2.5  4.1  contig10_pilon   728745  731394 (1121285) C  rnd-4_family-1113  LTR/Gypsy      (23146)   4587    1980   183     
 
 ANNOTATION EVIDENCE: 
  5200  31.89 2.49 4.14  contig10_pilon   728745  731394   1121285 C  rnd-4_family-1113  LTR/Gypsy         1980   4587   23146      
5200 31.89 2.49 4.14 contig10_pilon 728745 731394 (1121285) C rnd-4_family-1113#LTR/Gypsy (23146) 4587 1980 m_b696s001i9

  contig10_pilo     728745 GGCCCTGAGACAAACT-GACGAGTGCAGGTCGCCTGTCTATGCTAGCCGC 728793
                            i  ii i i i    -i   i iv   vv i     vv vv  i ivv 
C rnd-4_family-       4587 GACCTCGGGGCGAACTCAACGGGCCCAGCGCACCTGTGGAATCTGGTGCC 4538

  contig10_pilo     728794 ATGACGAGGACTATGGAAGAGTGAGCATATGGTGCGACCGATGTTAACCA 728843
                           ii      i vv     ?    i   v iii    iv v viii --   
C rnd-4_family-       4537 GCGACGAGAAAGATGGANGAGTAAGCTTGCAGTGCATCAGCCACT--CCA 4490

  contig10_pilo     728844 TTC----GCTGATC-TATCGCGCGTTCCAATACAGCCTATCCGATGAGAG 728888
                           ? i----       -iii v   vv v  v v    i i  v  i iv  
C rnd-4_family-       4489 NTTTCGTGCTGATCGCGCCCCGCCGTGCACTTCAGCTTGTCGGACGGCAG 4440

  contig10_pilo     728889 CCCGACGATCAAGAGGTCCACCGGAAACCCCGGGTGCGCACTTCGTCCG- 728937
                             v   viv    ii i vii iv  iiv  i    v vii ii   vi-
C rnd-4_family-       4439 CCAGACCGGCAAGGAGCCAGTCACAAGTGCCAGGTGAGAGTTCTGTCGAT 4390

  contig10_pilo     728938 GCATCGCGTTTTG-GCTAACGTCA--ATCTTAGGAAGAAGCGCCGCCTCC 728984
                            ivi   vi  i -v  i  i iv--iiv    i        v   v   
C rnd-4_family-       4389 GTTCCGCTCTTCGCTCTGACATTTTCGCATTAGAAAGAAGCGGCGCGTCC 4340

  contig10_pilo     728985 AGACTATCAAGAAGTCTGAACGCAATGCGACGAGGACCAAGTCTGTCCTG 729034
                            ii i           v  i  v  i v   iv i       v   ii ?
C rnd-4_family-       4339 AAGCCATCAAGAAGTCAGAGCGAAACGGGACACGAACCAAGTGTGTTTTN 4290

  contig10_pilo     729035 CACCTATACTCTCGTTAGATTGTGCCACTGACCACTGCCAGGAGCCGGAA 729084
                            vv i ii  v  vivv   i     i     vi ivv      i  v  
C rnd-4_family-       4289 CCACCACGCTGTCCCGCGATCGTGCCGCTGACAGCCTGCAGGAGTCGCAA 4240

  contig10_pilo     729085 GCGGAGCGCGCGTAAGGCGTCGTGGAAGCAATTCATTGATGAGTATGTCA 729134
                           v  i  i     v               vvi     v  i     v    
C rnd-4_family-       4239 TCGAAGTGCGCGAAAGGCGTCGTGGAAGATGTTCATAGACGAGTAAGTCA 4190

  contig10_pilo     729135 C----GCC-GCTT-CCAGCG--ATATGGTGCTGAGATCCTGTCAGCGAGG 729176
                            ----  v-   i-      --  i  ii     v    iv       i 
C rnd-4_family-       4189 CCGAGGCAAGCTCACCAGCGCGATGTGACGCTGACATCCCTTCAGCGAAG 4140

  contig10_pilo     729177 CGATGGAGGCCAGTGCAGAATCTGAGTTGAGCGACATCGAGTAGGTGCCG 729226
                               v     v  i vi  i  i  v    v?  i  v    ii   v  
C rnd-4_family-       4139 CGATTGAGGCGAGCGGGGAGTCCGACTTGACNGATATGGAGTGAGTGACG 4090

  contig10_pilo     729227 CTTGTCGTCCACCTGCTGAGAGATACTCACGCTCGCGAATCAGCATGGAG 729276
                            i  i  ---- iv i     i ii  v    vi i v vi   i v  v
C rnd-4_family-       4089 CCTGCCG----CTGGTTGAGAAACGCTGACGCGTGTGCAATAGCGTCGAT 4044

  contig10_pilo     729277 CTCGCCGACGCCGCATTCGATGCTATGCCCGAACGTCTCGCACCG--GGA 729324
                           v   v ii  v  i-i  i   ---------i   -v  v  i  -- i 
C rnd-4_family-       4043 ATCGACAGCGGCGT-CTCAATG---------GACG-ATCCCATCGCCGAA 4005

  contig10_pilo     729325 GCTCTGCAACCAACTGGTAGCGATGATGAAGTTT---CGACGGGTTCGGC 729371
                            ii     vv      i  ?i vv ii  v i i---  i v i   v -
C rnd-4_family-       4004 GTCCTGCACGCAACTGATANTGTGGGCGACGCTCGCGCGGCTGATTCTG- 3956

  contig10_pilo     729372 AACAGAAAGCTCGACTACTGTCGCCGGCACTCCATCCGTGATGGAAGAAA 729421
                           -------  v   - vv     ---------i     iv ii    ----
C rnd-4_family-       3955 -------AGATCG-CGCCTGTC---------TCATCCAAGGCGGAA---- 3927

  contig10_pilo     729422 CTACGCGCATCGACGCCGGTGCCATGGAGGTTGCGCCGAAGGACACGGCA 729471
                           -------- v        ii      v  i  i   v i iv ivi  iv
C rnd-4_family-       3926 --------AGCGACGCCGACGCCATGCAGATTACGCAGGAACATCTGGTC 3885

  contig10_pilo     729472 TCTTTGCGAGAGGTTCTGGAAGGCGCGGAGAGCTCAGCGAGCCAGCCTCA 729521
                           i ivi      v i  ii  ii  v   vi  ii  iv  iv v   i i
C rnd-4_family-       3884 CCCGCGCGAGATGCTCCAGAGAGCCCGGCAAGTCCAAAGAAACCGCCCCG 3835

  contig10_pilo     729522 AGCAGGGACGACTGCT----GCGCACACTGGTGAAGGAGTTGGCGGCAG- 729566
                           v  i i i  v -   ---- v   ---     vvivv ii i ?v i -
C rnd-4_family-       3834 TGCGGAGGCGTC-GCTCAAGGGGCA---TGGTGTCACTGCCGACNCCGGG 3789

  contig10_pilo     729567 --AACGTGGGTGTTGGTGGAGTTGCGTATATCGTCGCTGACCCAGAGCAC 729614
                           --?          i i ?   i i  iviii   i       vviivii 
C rnd-4_family-       3788 GANACGTGGGTGTCGATNGAGCTACGCCCGCCGTTGCTGACCACAGTTGC 3739

  contig10_pilo     729615 AGGGATGGTCAGGAACGCCTTTCGGCTCTCGTAGACATTGTGTCCAAGAC 729664
                           v viiv v      ---   i  v  v     v  iv ii          
C rnd-4_family-       3738 TGTAGGGCTCAGGA---CCTCTCCGCGCTCGTTGATTTCATGTCCAAGAC 3692

  contig10_pilo     729665 ACCGAATCTATTGGAGCTC-TTGATGGAGCGAGCGTGCGTCCTTCACTCT 729713
                           i         i  -    i-        i i     i   ii v  -   
C rnd-4_family-       3691 GCCGAATCTACTG-AGCTTGTTGATGGAACAAGCGTACGTTTTGCA-TCT 3644

  contig10_pilo     729714 GCGAGACATCTTGAGGCTGACCAGTGGAATTC---CTGGCGCCAGTCACA 729760
                            i v viivviiv v     i---  vi ii ---  v ivvi    iv 
C rnd-4_family-       3643 GTGTGTTGGACCCACGCTGAT---TGCGACCCGCTCTCGTTGTAGTCGGA 3597

  contig10_pilo     729761 GCACGAACAAGTTCGCAAAACCAGCAGAGCCCATCAAGACCAAAGAATCT 729810
                               vii   i    vv  i  i ii  i           v     vv v
C rnd-4_family-       3596 GCACTGGCAAATTCGACAAGCCGGTGGAACCCATCAAGACGAAAGACGCG 3547

  contig10_pilo     729811 GCGCCGGTAGACGCGGTTCCTACGCAGCAAACACAGCTCGGAGACGATTT 729860
                                v  i     i   v i  iv    i   v    v  ? i   i  
C rnd-4_family-       3546 GCGCCCGTGGACGCAGTTGCCACAAAGCAGACAGAGCTGGGNGGCGACTT 3497

  contig10_pilo     729861 CCTTGTTGATGCGAAGTTCGACGGGACAAAGGACTGCCTAGACCATACCC 729910
                              iv i  i        i     v  v     i   v ii    i vv 
C rnd-4_family-       3496 CCTCCTCGACGCGAAGTTTGACGGTACCAAGGATTGCATGAACCACAAGC 3447

  contig10_pilo     729911 TCTGGGACACAACACTGAAGGCCATGTACCGGAAGGTCCGCGCGCCACCC 729960
                            v      ivii v   viv v?        vvi?  v     v     v
C rnd-4_family-       3446 TATGGGACGAGGCCCTGCGCGANATGTACCGTCGNGTGCGCGCCCCACCG 3397

  contig10_pilo     729961 CGGTACGTCAAACTTTGAGGATGATTTCTATGCCTGACTTTTCGAACAGA 730010
                                     iii  i  i i  v    v i v  i   i  v v i   
C rnd-4_family-       3396 CGGTACGTCAGGTTTCGAAGGTGTTTTCGACGACTAACTCTTGGTATAGA 3347

  contig10_pilo     730011 CTCGTTGCGTATGATGTGCAGAAGGATGGGGCTATTATAGAGCCTGCTTC 730060
                           v i  i v    i ?    vi ?   v  v vvv  v iviv    vv v
C rnd-4_family-       3346 ATTGTCGGGTATAANGTGCCAANGGAGGGCGGGTTTCTGCGCCCTGAGTG 3297

  contig10_pilo     730061 CTTCAGAAAGTGGGCCAAGCATGAGAAGTCACCGTGAGTCGACCTGTTGC 730110
                                ii  i   v       ii i  v iv         vi viv iv 
C rnd-4_family-       3296 CTTCAAGAAATGGTCCAAGCACAAAAACTTTCCGTGAGTCCGCACTTCTC 3247

  contig10_pilo     730111 TGTCTCTAATGAAGGCTATGATACTCAACGGAGGTGCAGTGTCATCATGG 730160
                             v  vi  vi v ii vv i i    --v  v vii   v  v vv vi
C rnd-4_family-       3246 TGACTGCAAGAATGATTCGGGTGCTCA--TGATGGATAGTCTCCTGTTCA 3199

  contig10_pilo     730161 AGATCATGGATACCCCCTACACAACCGTATATGTCAACCCCAGCCGTATC 730210
                             i       iv   iii v ivv  ivi  i      v   v v vi  
C rnd-4_family-       3198 AGGTCATGGACTCCCTTCAGATCTCCAAGTACGTCAACGCCACCAGGGTC 3149

  contig10_pilo     730211 AGCCACAAGAAGATCCGGATCGAGGCGGGAGGGACTGCTGTCCATCCTTT 730260
                            i          v i  iv        ?vi  v v v  v ii i  ? i
C rnd-4_family-       3148 AACCACAAGAAGCTTCGACTCGAGGCGNCGGGCAGTTCTCTTTACCCNTC 3099

  contig10_pilo     730261 CAATTTTTTAGCCCCCCAAGGATCGTCTGAGCCCGCGATGTTCTTCAGCC 730310
                                v v vi  v  v?v v v  v i  i  v ivi v         v
C rnd-4_family-       3098 CAATTATGTTACCGCCANTGCAACGACCGAACCAGTCGTCTTCTTCAGCG 3049

  contig10_pilo     730311 TTGTCGAAACCAAGGTTTCATCGCTCTTATCAGTAACGACCATGAAG--- 730357
                           ii    vv v    iiv  v  vi vi    i  i    ?v     i---
C rnd-4_family-       3048 CCGTCGTCAACAAGACGTCTTCCTTGCTATCGGTGACGANGATGAAANGC 2999

  contig10_pilo     730358 AAGGATCAGCCCGACGCACTTGAGAAGAAGCTGCTCGTAGCCAACTTATT 730407
                           ?    iv      v i i  i           vv           i v  
C rnd-4_family-       2998 NAGGACAAGCCCGTCACGCTCGAGAAGAAGCTCATCGTAGCCAACCTCTT 2949

  contig10_pilo     730408 CAGGCAGGAGTTCGAACTCCTGGTATGCAACCTTGGTTGCATATACAACG 730457
                              i        i        v  v  i     i  i v   v       
C rnd-4_family-       2948 CAGACAGGAGTTTGAACTCCTCGTCTGTAACCTCGGCTCCATCTACAACG 2899

  contig10_pilo     730458 TCCAGGACATCACGCTCCCGAGTTGGTCCAATGTCGACGACACCCGCGGT 730507
                            vvi   v                      vi     v    v       
C rnd-4_family-       2898 TGGGGGAGATCACGCTCCCGAGTTGGTCCACCGTCGAAGACAGCCGCGGT 2849

  contig10_pilo     730508 TTGACCTTCCACACGAGGGTCGCGCCGGTCGAGAAAGGTCAGTATCTCGT 730557
                           i i        i  i      ivi  v  ?     ---- i   iv  ii
C rnd-4_family-       2848 CTAACCTTCCATACAAGGGTCAAACCCGTNGAGAA----CGGTACGTCAC 2803

  contig10_pilo     730558 CATTCGACGTCTGAATATGGGGCTGATTGTCTCTAGCCACTGCGTCGCGT 730607
                            -    ?  viv viiii i ii?   ii    v    ii   i--   i
C rnd-4_family-       2802 C-TTCGNCGATGGCGCGCGAGATNGATCATCTCAAGCCGTTGCA--GCGC 2756

  contig10_pilo     730608 GA-GACGGTGATCAAGACG-GGTTTGTTCAAGGGCTCTCGTAACGCGGCT 730655
                           i -v    v  vvi iv i-i      ?ivv   i   v i i     i 
C rnd-4_family-       2755 AACCACGGGGAGAGAACCACAGTTTGTNTTTGGGTTCTGGCAGCGCGGTT 2706

  contig10_pilo     730656 AGA------AAATCTAAGACAAAGACTCCTGTTCAACTCGTGGGCCGTGA 730699
                            i ------   v v     i     i  i     i        iv    
C rnd-4_family-       2705 AAACAGCAGAAAACGAAGACGAAGACCCCCGTTCAGCTCGTGGGTGGTGA 2656

  contig10_pilo     730700 ACGAGGTAAGCCCTTTTCTGGTACTTCGATCACTTTGGGCGCGCGCGCTG 730749
                             iv   i  i vv i  ii iiii    ----------- i i     i
C rnd-4_family-       2655 ACACGGTGAGTCAGTCTCCAGCGTCTCGA-----------GTGTGCGCTA 2617

  contig10_pilo     730750 ACTGCT-TCAGTGCCGCTGTTTGATATGTCGACGTTCAACTTCAAGACCT 730798
                               i -vi   i  ?i i     i         vvvii    i -----
C rnd-4_family-       2616 ACTGTTAATAGTACCNTTATTTGACATGTCGACGGAGGGCTTCGA----- 2572

  contig10_pilo     730799 CTTTCGCAAGTC--TCGGCTTCAAGCAC-TGCCACGACCTGGAGGAGGAG 730845
                           ---  v   ?  -- vii   v   v i-    -    v       i   
C rnd-4_family-       2571 ---TCTCAANTCCGTGAACTTGAAGAATATGCC-CGACATGGAGGAAGAG 2526

  contig10_pilo     730846 CCCCCCCAGGGATCGCTGGGCGTGGTCTTCTATACCATCTCTGACAGGGG 730895
                           vii   vv      vv v  v  v        i   i          i  
C rnd-4_family-       2525 GTTCCCACGGGATCCGTTGGAGTCGTCTTCTACACCGTCTCTGACAGAGG 2476

  contig10_pilo     730896 TGGTCCAAGCAAGATCGAGTTTAACATTCACGGCTTCGGATTAATCGCCA 730945
                           i     vi   vi        i  ii i       v   v  i     v 
C rnd-4_family-       2475 CGGTCCCGGCACAATCGAGTTCAATGTCCACGGCTACGGCTTGATCGCAA 2426

  contig10_pilo     730946 CGCCGTCTGAAGATCGCTGGGCCGATTCAGATTAAATATCTCCTCGCTAA 730995
                                vvi     v  v    v            i iv    vi    vi
C rnd-4_family-       2425 CGCCGGACGAAGAGCGATGGGACGATTCAGATTAGACCTCTCGCCGCTCG 2376

  contig10_pilo     730996 TGCGCCCGTCGCTGGGATCACGATGGCGGCGATAACATATACGTGATGGA 731045
                             vviv                 i    i   ivvi v            
C rnd-4_family-       2375 TGACTGCGTCGCTGGGATCACGACGGCGACGACTTTAGATACGTGATGGA 2326

  contig10_pilo     731046 CATTATCTTAACAGTTCGCTTTTAGTTG--TGCCCAAGTTGTAATGCGC- 731092
                            iv  i v       ii  v      i --vi viv   i         -
C rnd-4_family-       2325 CGGTACCGTAACAGTCTGCATTTAGTCGGTAACGTTAGTCGTAATGCGCG 2276

  contig10_pilo     731093 ------TTTT---TGTCCGGATGTTCGAGACCTAATATTTTCATACGCTA 731133
                           ------ i  ---  i  i iv        vv v            ii  
C rnd-4_family-       2275 CCGTTGTCTTATGTGCCCAGGGGTTCGAGAAGTCATATTTTCATACATTA 2226

  contig10_pilo     731134 ATTCATTTGTTGATGTCGTGCA--TGGCAGGCGCATTGAAGCGACGAAAG 731181
                             i  v    i    v  i   --i  i--      v     i i vi  
C rnd-4_family-       2225 ATCCAATTGTCGATGACGCGCAACCGGT--GCGCATGGAAGCAATGCGAG 2178

  contig10_pilo     731182 GGGCAATTTTGGTTGTATTGGCATAGGCTGGGGAATATACAACACGCGTG 731231
                           v ii  vviviii v      v  v i  i  v ii    ------i   
C rnd-4_family-       2177 TGATAAAACGAACTTTATTGGAATCGACTAGGTAGCATAC------TGTG 2134

  contig10_pilo     731232 CCAGTCAAAAATGCCACCTCGTCTCGAAGG--AAGATGATGATGGACGCC 731279
                            ------i i i v  i?v       v   --    i--i i i     i
C rnd-4_family-       2133 C------GAGACGGCATNACGTCTCGCAGGGCAAGAC--CGGTAGACGCT 2092

  contig10_pilo     731280 GGCGCCCAGCGCCAGAGAAGTAGGAATGGATGGATGGATGAATAGCAAGC 731329
                                v   viiii  ii   v v ii  i   v      ---i v i i
C rnd-4_family-       2091 GGCGCACAGGATTGGAAGAGTCGTAGCGGGTGGTTGGATG---GGAAGGT 2045

  contig10_pilo     731330 GATGGCGACGGGTATGGGCA-CAAAGTAGGCATTGGCGTGTGGTTGTCGA 731378
                               iv v    -   v  i-   i i??  v      vvi i v     
C rnd-4_family-       2044 GATGAGGTCGGG-ATGCGCGCCAAGGCNNGCCTTGGCGGTCGATGGTCGA 1996

  contig10_pilo     731379 GAACTGCCGCACAATG 731394
                             i   ii v  i   
C rnd-4_family-       1995 GAGCTGTTGGACGATG 1980

Matrix = 20p53g.matrix
Kimura (with divCpGMod) = 37.24
Transitions / transversions = 1.28 (455/355)
Gap_init rate = 0.05 (139 / 2649), avg. gap size = 1.25 (174 / 139)

 
 
 
  +     378   20.7  0.0  0.0  contig10_pilon   731612  731693 (1120986) C  rnd-4_family-1113  LTR/Gypsy      (25927)   1806    1725   183     
 
 ANNOTATION EVIDENCE: 
   378  20.73 0.00 0.00  contig10_pilon   731612  731693   1120986 C  rnd-4_family-1113  LTR/Gypsy         1725   1806   25927      
378 20.73 0.00 0.00 contig10_pilon 731612 731693 (1120986) C rnd-4_family-1113#LTR/Gypsy (25927) 1806 1725 m_b696s001i10

  contig10_pilo     731612 GCCATCTGCGCTCAAGTCGACAGTCCGTCAACCGAACTACAGACGCGGTG 731661
                              i                  i                   i  v  i 
C rnd-4_family-       1806 GCCGTCTGCGCTCAAGTCGACAATCCGTCAACCGAACTACAGGCGGGGCG 1757

  contig10_pilo     731662 GTGGAGGGGAGGAAGAGATGCGGAGTTTGCCG 731693
                             vi  iv   i   iv? v   vvii     
C rnd-4_family-       1756 GTCAAGACGAGAAAGGCNTCCGGCTCCTGCCG 1725

Matrix = 20p53g.matrix
Kimura (with divCpGMod) = 21.79
Transitions / transversions = 1.43 (10/7)
Gap_init rate = 0.00 (0 / 81), avg. gap size = 0.0 (0 / 0)

 
 

 
 
   +     255   25.5  3.2  3.9  contig10_pilon   749523  749677 (1103002) C  rnd-4_family-310   LINE/R1          (168)    632     479   186     
 
 ANNOTATION EVIDENCE: 
   255  25.52 3.23 3.90  contig10_pilon   749523  749677   1103002 C  rnd-4_family-310   LINE/R1            479    632     168      
255 25.52 3.23 3.90 contig10_pilon 749523 749677 (1103002) C rnd-4_family-310#LINE/R1 (168) 632 479 m_b696s001i11

  contig10_pilo     749523 CGCCTGCTGCTG--GTACCCGCTCG--CCTGCTGCTGGTGC-TGCGGCGG 749567
                                       --  ?v i ?v  --i         v?  -   iv v 
C rnd-4_family-        632 CGCCTGCTGCTGCTGTNGCTGNACGGCTCTGCTGCTGCNGCCTGCATCTG 583

  contig10_pilo     749568 GCTCTGCCATCCATTCTGCGCGCCCGCAGCTGACGGATAGCCGTTCGACT 749617
                           viv    ii v i  vi-      i  v   vi  --iv  i   --i  
C rnd-4_family-        582 CTGCTGCTGTGCGTTGC-CGCGCCTGCCGCTCGCG--CTGCTGTT--GCT 538

  contig10_pilo     749618 GCCGCGGCTGCTGCTGTTGCTGTTGCTGCTGCTGCTGGTATTGCTGCTGG 749667
                               -   i vi i  i     i     i        v ii        v
C rnd-4_family-        537 GCCG-GGCCGGCGTTGCTGCTGCTGCTGTTGCTGCTGCTGCTGCTGCTGC 489

  contig10_pilo     749668 TAGAACTGCG 749677
                            i  i     
C rnd-4_family-        488 TGGAGCTGCG 479

Matrix = 20p53g.matrix
Kimura (with divCpGMod) = 28.73
Transitions / transversions = 1.53 (23/15)
Gap_init rate = 0.06 (9 / 154), avg. gap size = 1.22 (11 / 9)

 
 
 
 
 
  +     360   17.8  4.3  3.2  contig10_pilon   752853  752945 (1099734) +  rnd-4_family-331   Unknown            763    856   (542)   187     
 
 ANNOTATION EVIDENCE: 
   360  17.77 4.30 3.19  contig10_pilon   752853  752945   1099734 +  rnd-4_family-331   Unknown            763    856     542      
360 17.77 4.30 3.19 contig10_pilon 752853 752945 (1099734) rnd-4_family-331#Unknown 763 856 (542) m_b697s001i0

  contig10_pilo     752853 GATGGCGGAAAGAG---GGGGAAAAGAGGGCAACGACGCGTCCGCGCCGG 752899
                             i     i i  i---    i         ii         iv    v 
  rnd-4_family-        763 GACGGCGGGAGGAAAGTGGGGGAAAGAGGGCGGCGACGCGTCTCCGCCCG 812

  contig10_pilo     752900 TG-GTCCTGCTTTTATAGTCAAGCTTCTGCCCTCCAATCCGTTGGGG 752945
                             - i     ?      -i i  v --i  v                
  rnd-4_family-        813 TGCGCCCTGCNTTTATA-CCGAGAT--CGCGCTCCAATCCGTTGGGG 856

Matrix = 20p53g.matrix
Kimura (with divCpGMod) = 15.66
Transitions / transversions = 3.00 (12/4)
Gap_init rate = 0.05 (5 / 92), avg. gap size = 1.40 (7 / 5)

 
 

 
 
   +     255   28.7  2.7  1.8  contig10_pilon   753073  753182 (1099497) C  rnd-4_family-331   Unknown          (508)    890     780   188     
 
 ANNOTATION EVIDENCE: 
   255  28.70 2.73 1.80  contig10_pilon   753073  753182   1099497 C  rnd-4_family-331   Unknown            780    890     508      
255 28.70 2.73 1.80 contig10_pilon 753073 753182 (1099497) C rnd-4_family-331#Unknown (508) 890 780 m_b697s001i1

  contig10_pilo     753073 CACCCCAATGGAGTTTTCTCCTATGGGAGGCTTACCCCAACGAATTGGCA 753122
                                   i   v i     vviv ?ii i vi         i     vi
C rnd-4_family-        890 CACCCCAACGGATTCTTCTCAGGGGNAGGACGCACCCCAACGGATTGGAG 841

  contig10_pilo     753123 GACGTCCTCCATATAAAGTCACGCCCCCCGGCGCGATGGACACGT---CG 753169
                           vi  vi   vi      ?v  v v v v   -   i-i   i   ---  
C rnd-4_family-        840 CGCGATCTCGGTATAAANGCAGGGCGCACGG-GCGG-AGACGCGTCGCCG 793

  contig10_pilo     753170 TCCTCTTTCCCTC 753182
                           i          i 
C rnd-4_family-        792 CCCTCTTTCCCCC 780

Matrix = 20p53g.matrix
Kimura (with divCpGMod) = 33.37
Transitions / transversions = 1.21 (17/14)
Gap_init rate = 0.03 (3 / 109), avg. gap size = 1.67 (5 / 3)

 
 
 
 
 
  +      23   37.5  0.0  0.0  contig10_pilon   753975  754075 (1098604) +  (CACCAG)n          Simple_repeat        1    101     (0)   189     
 
 ANNOTATION EVIDENCE: 
    23  37.45 0.00 0.00  contig10_pilon   753975  754075   1098604 +  (CACCAG)n          Simple_repeat        1    101       0      
23 37.45 0.00 0.00 contig10_pilon 753975 754075 (1098604) (CACCAG)n#Simple_repeat 1 101 (0) m_b697s252i0

  contig10_pilo     753975 CACCAGCATCGCCAGCGGCAGCAGCACCAGCATCGCCGCCGGCTCCGGCC 754024
                                   i iv  v i   v           i iv i  i  v  i  v
  (CACCAG)n#Sim          1 CACCAGCACCAGCACCAGCACCAGCACCAGCACCAGCACCAGCACCAGCA 50

  contig10_pilo     754025 CCGGCGTCCGCTCCCGCACTGGCACCTGCGCCGGCGCCGGCACCCGCATC 754074
                             i  ii v  v  v    ii     v  i  i  i  i     v   i 
  (CACCAG)n#Sim         51 CCAGCACCAGCACCAGCACCAGCACCAGCACCAGCACCAGCACCAGCACC 100

  contig10_pilo     754075 A 754075
                            
  (CACCAG)n#Sim        101 A 101

Matrix = Unknown
Transitions / transversions = 1.64 (18/11)
Gap_init rate = 0.00 (0 / 100), avg. gap size = 0.0 (0 / 0)

 
 

 
 
   +      14   17.2  5.1  0.0  contig10_pilon   755658  755696 (1096983) +  (CGCTCG)n          Simple_repeat        1     41     (0)   190     
 
 ANNOTATION EVIDENCE: 
    14  17.22 5.13 0.00  contig10_pilon   755658  755696   1096983 +  (CGCTCG)n          Simple_repeat        1     41       0      
14 17.22 5.13 0.00 contig10_pilon 755658 755696 (1096983) (CGCTCG)n#Simple_repeat 1 41 (0) m_b697s252i1

  contig10_pilo     755658 CGCTTGCGCTCGCTCTCGCGGCCTCGAT-G-GCTCGCGCTC 755696
                               i        v      vi v  v - -          
  (CGCTCG)n#Sim          1 CGCTCGCGCTCGCGCTCGCGCTCGCGCTCGCGCTCGCGCTC 41

Matrix = Unknown
Transitions / transversions = 0.50 (2/4)
Gap_init rate = 0.05 (2 / 38), avg. gap size = 1.00 (2 / 2)

 
 
 
 
 
  +      16    8.1  0.0  0.0  contig10_pilon   761421  761446 (1091233) +  (CGGAG)n           Simple_repeat        1     26     (0)   191     
 
 ANNOTATION EVIDENCE: 
    16   8.13 0.00 0.00  contig10_pilon   761421  761446   1091233 +  (CGGAG)n           Simple_repeat        1     26       0      
16 8.13 0.00 0.00 contig10_pilon 761421 761446 (1091233) (CGGAG)n#Simple_repeat 1 26 (0) m_b697s252i2

  contig10_pilo     761421 CGGGGCGGAGCTGAGCGGAGCGGAGC 761446
                              i       v              
  (CGGAG)n#Simp          1 CGGAGCGGAGCGGAGCGGAGCGGAGC 26

Matrix = Unknown
Transitions / transversions = 1.00 (1/1)
Gap_init rate = 0.00 (0 / 25), avg. gap size = 0.0 (0 / 0)

 
 

 
 
   +      12   24.9  2.0  4.0  contig10_pilon   770142  770192 (1082487) +  (CGC)n             Simple_repeat        1     50     (0)   192     
 
 ANNOTATION EVIDENCE: 
    12  24.93 1.96 4.00  contig10_pilon   770142  770192   1082487 +  (CGC)n             Simple_repeat        1     50       0      
12 24.93 1.96 4.00 contig10_pilon 770142 770192 (1082487) (CGC)n#Simple_repeat 1 50 (0) m_b697s252i3

  contig10_pilo     770142 CGCCGCCTCGAACTCGGCCGCCGTCG-AGCCGCTCGCCTCCGGCCGCCGG 770190
                                  v viv v v       i  -v     -    v  -       v
  (CGC)n#Simple          1 CGCCGCCGCCGCCGCCGCCGCCGCCGCCGCCGC-CGCCGCC-GCCGCCGC 48

  contig10_pilo     770191 CG 770192
                             
  (CGC)n#Simple         49 CG 50

Matrix = Unknown
Transitions / transversions = 0.25 (2/8)
Gap_init rate = 0.06 (3 / 50), avg. gap size = 1.00 (3 / 3)

 
 
 
 
 
  +      15   17.7  5.0  2.4  contig10_pilon   770588  770627 (1082052) +  (CG)n              Simple_repeat        1     41     (0)   193     
 
 ANNOTATION EVIDENCE: 
    15  17.73 5.00 2.44  contig10_pilon   770588  770627   1082052 +  (CG)n              Simple_repeat        1     41       0      
15 17.73 5.00 2.44 contig10_pilon 770588 770627 (1082052) (CG)n#Simple_repeat 1 41 (0) m_b697s252i4

  contig10_pilo     770588 CGCGCGCGAGAG-GTGCGCGCGCG-GCTGCGAGCCCTCGCGC 770627
                                   v v - i         -  -   v  v v     
  (CG)n#Simple_          1 CGCGCGCGCGCGCGCGCGCGCGCGCGC-GCGCGCGCGCGCGC 41

Matrix = Unknown
Transitions / transversions = 0.20 (1/5)
Gap_init rate = 0.08 (3 / 39), avg. gap size = 1.00 (3 / 3)

 
 

 
 
   +      18   20.1  0.0  0.0  contig10_pilon   771549  771588 (1081091) +  (CGG)n             Simple_repeat        1     40     (0)   194     
 
 ANNOTATION EVIDENCE: 
    18  20.07 0.00 0.00  contig10_pilon   771549  771588   1081091 +  (CGG)n             Simple_repeat        1     40       0      
18 20.07 0.00 0.00 contig10_pilon 771549 771588 (1081091) (CGG)n#Simple_repeat 1 40 (0) m_b697s252i5
[truncated: 30,890,800 more chars]
