# Supplementary material for: Comparative Genomic Analysis of Two Monokaryons of Auricularia heimuer Hei29
Source: J Fungi (Basel). 2025 Feb 6;11(2):122. doi: 10.3390/jof11020122 (PMC11856363; doi:10.3390/jof11020122)
Supplement: Supplementary file 1 [file jof-11-00122-s001.zip › supplementary.pdf]

Comparative Genomic Analysis of Two Monokaryons of *Auricularia heimuer* Hei29

Fengli Wang [1,†], Chuang Han [1,2,†], Jiechi Zhang [1], Piqi Zhang [1], Xiaojia Zhang [1], Xin Yue [1], Yanshu Zhao [1] and Xiaodong Dai [1*]

[1.] Institute of Microbiology, Heilongjiang Academy of Sciences, Harbin 150010, Heilongjiang, China
[2.] College of Plant Protection, Northeast Agricultural University/Key Laboratory of Agricultural Microbiology of Heilongjiang Province, Harbin 150030, Heilongjiang, China
[†] These authors contributed equally to this work.
* Correspondence: heiweihlj@126.com

Supplementary

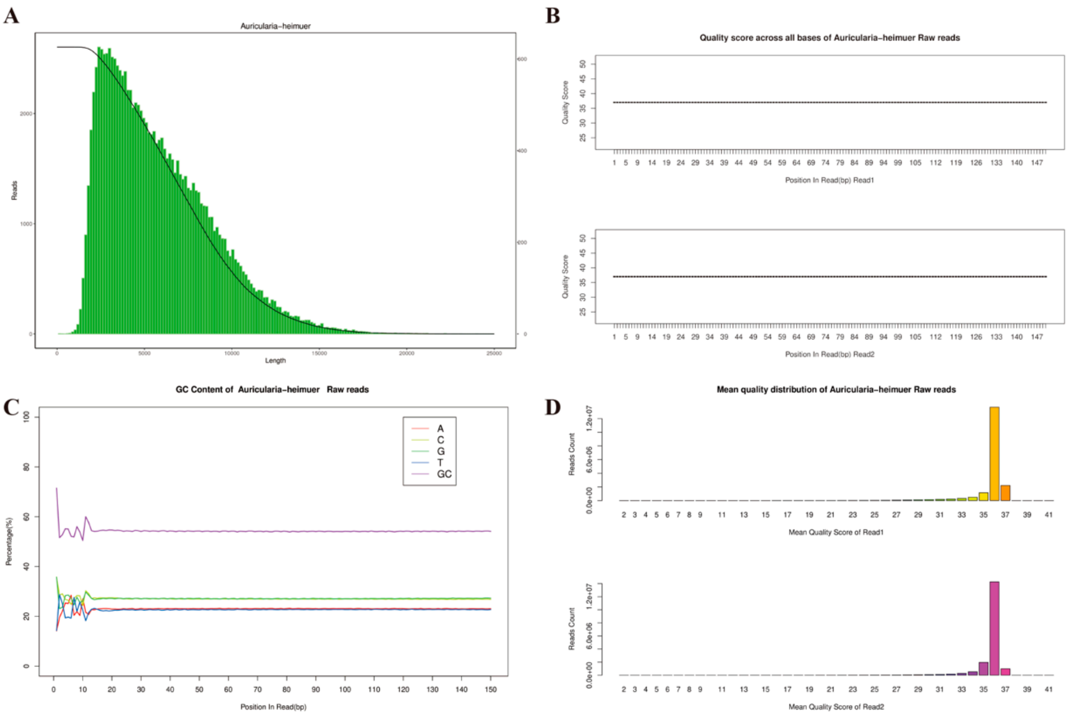

FigureS1 Quality Control of Hei29D1. A sequencing data length distribution (PacBio data); B reads sequencing quality distribution map (illumina data); C reads base type percentage statistical map (illumina data) ;D reads average quality distribution map (illumina data).

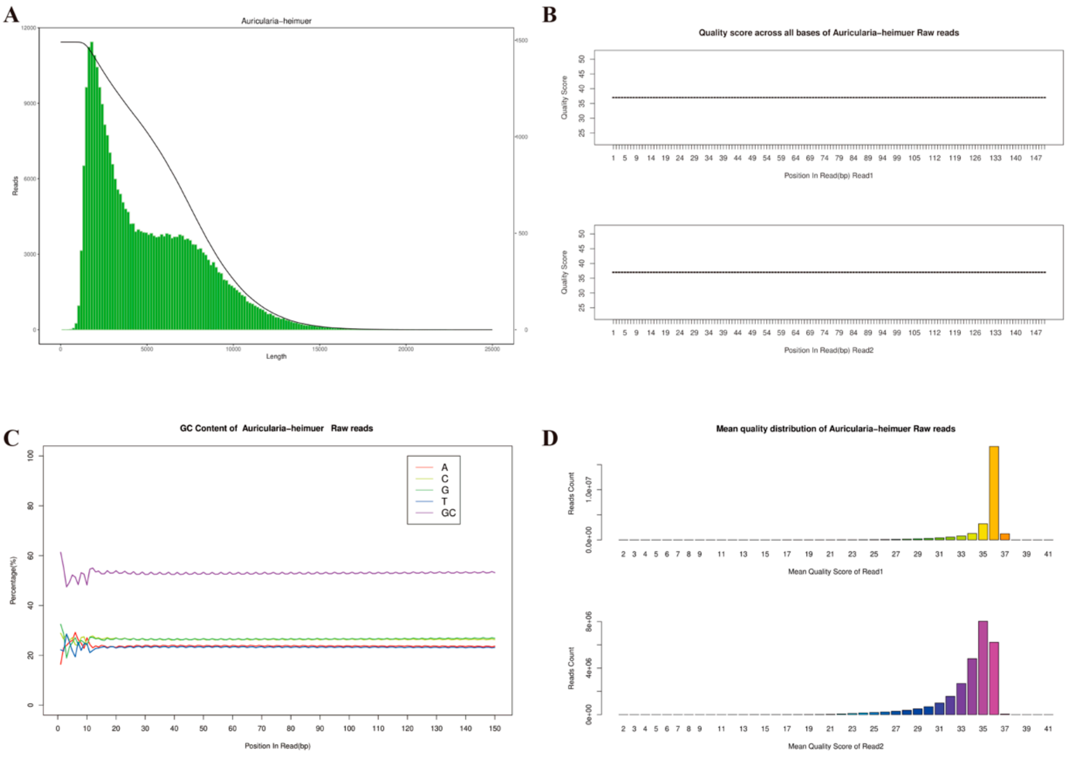

FigureS2 Quality Control of Hei29D2. A sequencing data length distribution (PacBio data); B reads sequencing quality distribution map (illumina data); C reads base type percentage statistical map (illumina data) ;D reads average quality distribution map (illumina data).

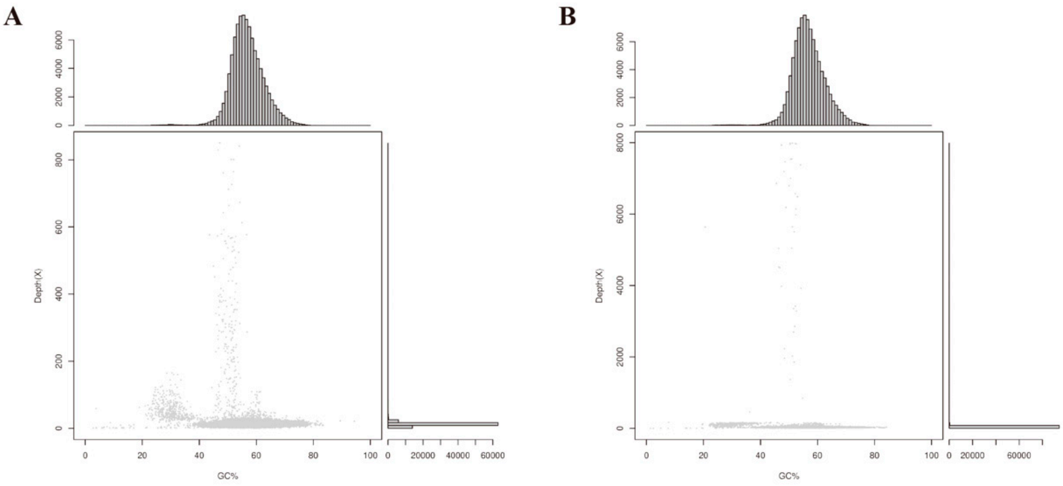

FigureS3 Statistical plot of correlation analysis between GC content and depth. A Hei29-D1; B Hei29-D2.

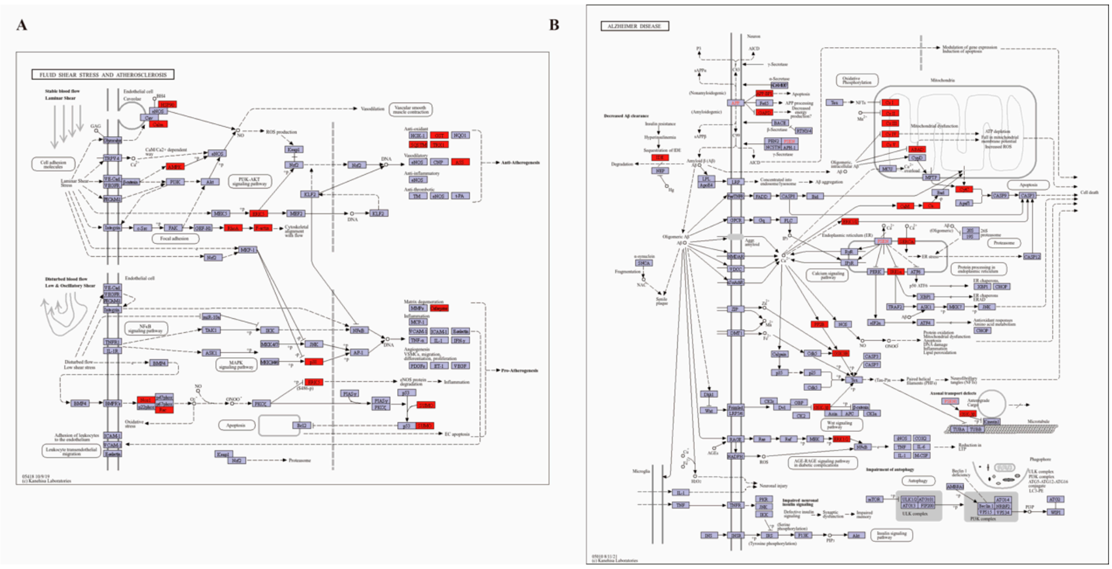

FigureS4 KEGG pathway of Hei29. **A** Fluid shear stress and atherosclerosis KEGG pathway of Hei29; **B** Alzheimer disease KEGG pathway of Hei29 (Genes that Hei29 is present in the pathway highlighted in red).

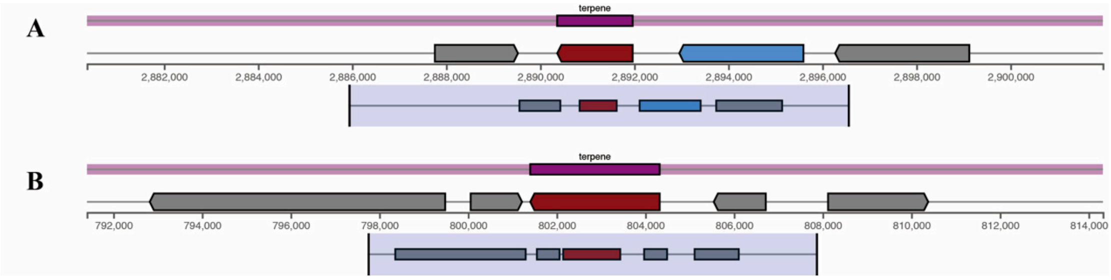

FigureS5. Phytoene synthase gene of Hei29. **A.** Terpene gene clusters in Hei29-D1 genomes (g894); **B.** Biosynthetic gene clusters in Hei29 genomes (911183).

TableS1 Clean data of Hei29

Sample	PF Reads	Clean Reads	Ratio of Reads(%)	PF Bases(bp)	Clean Bases(bp)	Ratio of Bases(%)
Hei29-D1	37442126	37090016	99.06	5616318900	5538340659	98.61
Hei29-D2	54059978	53235496	98.47	8108996700	7963489039	98.21

TableS2 GO and Gene distribution of Hei29-D1

Type	Class	GO Number	Gene Number
	Biological regulation	1235	1251
	Cellular process	1023	2807
Biological Process	Metabolic process	965	2600
	Localization	456	957
	Cellular component organization or biogenesis	356	991

	Responce to simulus	305	713
	Developmental process	304	324
	Reproductive process	86	202
	Multicellular organismal process	85	152
	Multi-organism process	53	178
	Immune system process	26	37
	Locomotion	25	34
	Growth	24	56
	Behavior	20	18
	Biological adhesion	17	115
	Detoxification	13	47
	Cell population proliferation	7	15
	Reproduction	6	13
	signaling	4	26
	Rhythmic process	4	14
	Cell killing	3	4
	Pigmentation	2	2
	Nitrogen utilization	2	4
	Cell aggregation	1	1
	Carbon utilization	1	4
Molecular Function	Catalytic activity	1513	3550
	Binding	477	3751
	Transporter activity	218	470
	Molecular function regular	57	198
	Molecular transducer activity	16	35
	Antioxidant activity	15	79
	Transcription regulator activity	13	202
	Structural molecule activity	12	216
	Translation regulator activity	10	96
	molecular carrier activity	5	21
	Cargo receptor activity	4	16
	Small molecule sensor activity	2	8
	Toxin activity	1	2
	Protein tag	1	6
	Nutrient reservoir activity	1	1
	Protein folding chaperone	1	9
Cellular Component	Protein-containing complex	471	1398
	Cell part	391	3494
	Organelle part	370	2002

	Organelle	85	2659
	Membrane part	60	1258
	Membrane	54	10062
	Synapse part	26	33
	Extracellular region part	15	152
	Synapse	10	24
	Membrane-enclosed lumen	9	160
	Cell junction	8	45
	Other organism part	5	28
	Extracellular region	2	557
	Vision part	2	15
	Necleoid	2	13

TableS3 GO and Gene distribution of Hei29-D2

Type	Class	GO Number	Gene Number
Biological Process	Metabolic process	328	2305
	Cellular process	224	1393
	Localization	73	581
	Cellular component organization or biogenesis	60	152
	Biological regulation	46	353
	Response to stimulus	17	137
	Detoxification	2	2
	Reproductive process	2	2
	Developmental process	2	3
	Multi-organism process	1	1
	Biological adhesion	1	1
	Cell killing	1	1
	Multicellular organismal process	1	1
Molecular Function	Catalytic activity	598	3002
	Binding	130	3717
	Transporter activity	44	271
	Molecular function regulator	23	63
	Antioxidant activity	8	72
	Transcription regulator activity	5	139
	Structural molecule activity	5	129
	Molecular transducer activity	4	6
	Translation regulator activity	3	36
	Molecular carrier activity	2	3
	Nutrient reservoir activity	1	3

	Small molecules sensor activity	1	8
Cellular Component	Protein-containing complex	148	445
	Organelle part	79	231
	Cell part	78	437
	Organelle	15	370
	Membrane part	9	425
	Membrane	5	268
	Extracellular region part	1	9
	extracellular region	1	56
	Membrane-enclosed lumen	1	2

TableS4 The biosynthetic gene cluster of Hei29

Gene Cluster Type	core biosynthetic gene	Location (Hei29-D1)	Location (Hei29-D2)	Gene ID (Hei29-D1)	Gene ID (Hei29-D2)
Terpene	phytoene_synt	contig1	contig2	g894	g2384
	Terpene_synth_C	contig2	contig1	g1748	g1306
	terpene_cyclase	contig8	contig6	g7708	g7843
	TRI5	contig11	contig10	g9430	g11917
	TRI5	contig12	contig3	g9719	g4021
	phytoene_synt	contig15	contig4	g11183	g6014
	Terpene_synth_C	contig16	contig13	g11460	g13273
	Terpene_synth_C	contig19	contig1	g12256	g506
NRPS-like	AMP-binding; NAD_binding_4; PP-binding; Polysacc_synt_2	contig2	contig1	g1709	g1345
	AMP-binding; NAD_binding_4; PP-binding	contig12	contig3	g9747	g4049
	AMP-binding; PP-binding; 2OG-FeII_Oxy	contig12	contig3	g9980	g4282
	AMP-binding; PP-binding; 2OG-FeII_Oxy	contig12	contig3	g9994	g4296
	AMP-binding; NAD_binding_4; PP-binding	contig14	contig1	g10753	g210
	Condensation; AMP-binding; PP-binding	contig15	contig4	g11232	g5964
	AMP-binding; NAD_binding_4; PP-binding	contig16	contig13	g11360	g13173
	AMP-binding; NAD_binding_4	contig16	contig13	g11370	g13183
	AMP-binding; NAD_binding_4; PP-binding	contig28	contig11	g13491	g12728
Indole	dmat	contig2	contig1	g1993	g1063
	dmat	contig5	contig11	g5500	g12582

dmat		contig6	contig9	g6504	g11039

TableS5 The UGPase related genes of Hei29

	Gene_ID		Start	End	Strand
	g12660	contig21	394523	397315	+
	g12657	contig21	384060	386118	+
	g12654	contig21	373597	375655	+
	g12651	contig21	363137	365192	+
	g12637	contig21	305696	308464	+
Hei29-D1	g12676	contig21	459142	460572	-
	g12669	contig21	432308	436519	-
	g12667	contig21	422717	426928	-
	g12671	contig21	441899	443639	-
	g12679	contig21	468615	470696	-
	g12635	contig21	297376	300451	-
	g13451	contig15	79158	81950	-
	g13454	contig15	90355	92410	-
	g13468	contig15	147083	149851	-
	g13437	contig15	25492	26922	+
	g13442	contig15	42425	44165	+
Hei29-D2	g13444	contig15	49545	53756	+
	g13434	contig15	15368	17449	+
	g13470	contig15	155096	158171	+
	g13943	contig24	15230	17288	-
	g13940	contig24	4767	6825	-
